# Geminal Difunctionalization of Ketones via C─S Bond Insertion of Photogenerated Donor–Donor Diazo Compounds

**DOI:** 10.1002/anie.6162809

**Published:** 2026-05-02

**Authors:** Vincent George, Aryaman Pattanaik, Daniel Maddox, Lukas M. Sigmund, Giovanna Mejia, Sara Pahlén, Simon O. Angerer, Maria Schmoll, Lisa Marie Schneider, Nidhal Selmi, Mikhail Kabeshov, Giulia Bergonzini, Julia Rehbein, Burkhard König

**Affiliations:** ^1^ Faculty of Chemistry and Pharmacy University of Regensburg Regensburg Germany; ^2^ Compound Synthesis and Management, Discovery Sciences BioPharmaceuticals R&D AstraZeneca Mölndal Sweden; ^3^ Molecular AI, Discovery Sciences BioPharmaceuticals R&D AstraZeneca Mölndal Sweden

**Keywords:** chemical library, diazo compounds, difunctionalization, flow chemistry, photochemistry

## Abstract

Geminal difunctionalization of carbonyl‐derived building blocks represents a versatile strategy for the rapid generation of sp^3^‐rich molecular architectures. In this context, diazo compounds provide a powerful platform for installing two distinct functional groups, yet the reaction space for carbonyl‐derived donor–donor diazo systems remains underdeveloped. Here, we report a metal‐free migratory insertion of diazo compounds into C─S bonds of sulfonyl cyanides, enabling the simultaneous installation of sulfone and nitrile functionalities at a single carbon center. Key to this transformation is the in situ generation of highly reactive diazo intermediates via photochemical decomposition of bench‐stable oxadiazolines derived from ketones. This substantially expands the accessible coupling partner space, previously limited to aldehydes or boronic acids. The reaction exhibits broad functional group, water, and air tolerance, delivers high yields, and provides excellent diastereoselectivity in constrained cyclic systems. Compatibility with both batch and continuous‐flow processing, as well as its application to a realistic medicinal chemistry combinatorial library synthesis, highlights the practical utility of the method.

## Graphical abstract

Geminal difunctionalization of readily available building blocks provides rapid access to molecular complexity. This work introduces a strategy for installing two distinct functional groups on a carbonyl carbon by transforming it into a donor–donor diazo compound, enabling both batch and flow applications with broad solvent compatibility.

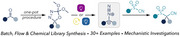

## 1 Introduction

**FIGURE 1:**
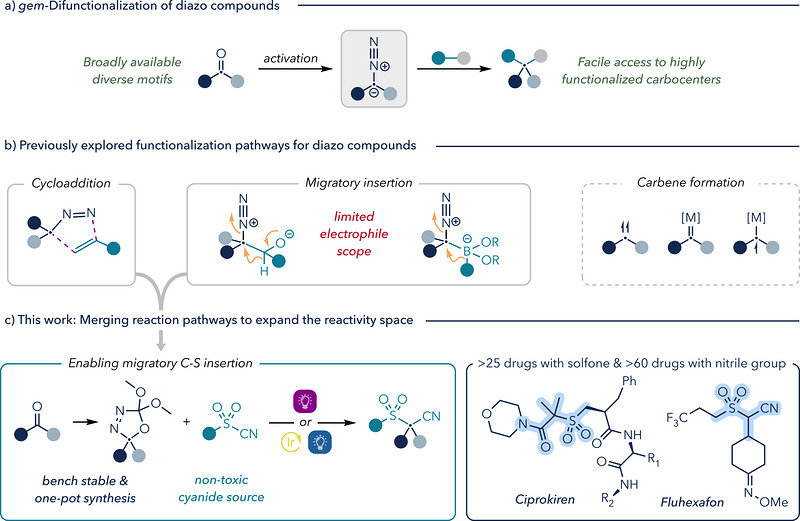
Diazo compound reaction modes and this work.

Ketones are among the most ubiquitous functional groups in organic chemistry. They are central motifs in biologically active molecules and functional materials and serve as fundamental synthetic building blocks, which makes them attractive platforms for diversification [1]. An especially appealing strategy is the direct introduction of two distinct functional groups at the carbonyl center, which not only increases molecular complexity but also facilitates the generation of sp^3^‐rich architectures [2]. One strategy to enable such transformations is to activate the carbonyl moiety, for example, by converting it into a diazo derivative (Figure 1a).

Diazo compounds are versatile and powerful reagents in modern synthetic chemistry, owing to their diverse reactivity. Among these, geminal difunctionalization—where two distinct functional groups are introduced onto the diazo carbon–has gained increasing attention as a powerful strategy for constructing highly functionalized scaffolds (Figure 1a). While significant progress has been made in the functionalization of stabilized diazo compounds [3, 4, 5, 6], such as diazo acetates, commonly via free or metal carbene moieties, the corresponding transformations of unstabilized diazo compounds, e.g., bearing two alkyl substituents, remain comparatively underexplored.

Donor–donor diazo compounds differ fundamentally from their stabilized counterparts, exhibiting a unique nucleophilic character. This reactivity has enabled a range of transformations, including (3+2) cycloadditions with electron‐deficient olefins [7, 8, 9, 10, 11], formal migratory insertions with electrophiles, and the generation of dialkylcarbenes [10, 12] and metalloradical intermediates [13] (Figure 1b). Of particular interest are migratory bond insertion reactions, which proceed via nucleophilic addition of the diazo carbon to an electrophile, followed by 1,2‐migration and nitrogen extrusion to form the final product. This mechanistic paradigm is exemplified by the Büchner–Curtius–Schlotterbeck (BCS) reaction, in which diazo compounds undergo addition to aldehydes followed by a 1,2‐hydride shift, ultimately yielding ketones [14, 15, 16]. Building on this logic, Barluenga, Valdés, and coworkers have developed a series of protocols involving boron‐based electrophiles to effect formal B–C [17, 18, 19, 20] insertions, which were later expanded by Wang and colleagues to include B─B and B─Si bond insertions [21]. Despite these advances, the overall scope of electrophiles in *gem*‐difunctionalization remains limited.

To address this gap, we aimed to identify new electrophilic partners that would enable the installation of two high‐value functional groups. While BCS‐type chemistry provides an elegant framework, a notable limitation is the inherent potential for divergent reaction pathways from the intermediate [22]. We therefore sought an electrophile that would ensure a well‐defined intermediate, such as one formed during a cycloaddition, while still enabling a subsequent 1,2‐migration to generate the desired product.

Sulfonyl cyanides emerged as a particularly attractive class of electrophiles for this purpose. These non‐toxic electrophilic reagents are compatible with cycloaddition chemistry and are well established for 1,2‐difunctionalization of alkenes [23, 24, 25, 26, 27, 28]. Importantly, sulfonyl cyanides incorporate two high‐value groups: sulfones and nitriles. Sulfones serve as bioisosteric motifs in pharmaceuticals and can be found in over 25 currently approved drugs (Figure 1c) and in agrochemical applications, while nitriles are even more prevalent in the pharmaceutical industry and provide versatile handles for downstream transformations into amides, amines, and heterocycles [29]. Despite this, to the best of our knowledge, their use in *gem*‐difunctionalizations has not been reported previously.

A critical challenge in developing new transformations involving donor–donor diazo compounds lies in their safe handling, as they are generally unstable, toxic, and potentially explosive. Consequently, in situ generation of the reactive species is preferred, making precursor selection crucial. Tosylhydrazones are commonly used diazo precursors, requiring a base and heating or irradiation to cleave the tosyl group after deprotonation [30, 31]. However, this approach introduces an inherent drawback: the deprotonated tosylhydrazone can act as a competing nucleophile, potentially leading to undesired side reactions [32]. To overcome this limitation, we turned to dimethoxy‐1,3,4‐oxadiazolines, which enable mild photochemical diazo generation without competing nucleophilicity. Analogous to tosylhydrazones, this precursor can be synthesized in an operationally simple one‐pot procedure from ketones, with high functional group tolerance and scalability to multi‐gram quantities [10]. Although various substitution patterns have been used, Gryko and coworkers have recently demonstrated that oxadiazolines can be efficiently converted to diazo compounds under both direct and photosensitized excitation [10]. The flexibility and efficiency of this approach inspired us to explore its application in *gem*‐difunctionalization.

## 2 Results and Discussion

We started our investigation of the proposed formal C─S bond insertion using 5,5‐cyclo‐hexylidene‐2,2‐dimethoxy oxadiazoline (**1a**) as a model diazo compound in combination with commercially available tosyl cyanide (**2a**) as nitrile source. We were delighted to observe the formation of the desired product **3a** in good yields during our initial screening, and, after optimizing the reaction conditions, we obtained **3a** in 97% GC yield (Table 1 and Supporting Information for details). Notably, however, the reaction exhibited a relatively low sensitivity to solvent effects, proceeding efficiently in ethyl acetate, mesitylene, tert‐amyl alcohol, DMSO, and water or even without solvent. In certain solvents, such as *tert*‐amyl alcohol or mesitylene, product crystallization was observed directly from the reaction mixture, allowing for straightforward isolation at the expense of a moderate decrease in yield.

**TABLE 1:** Optimization of the reaction conditions.

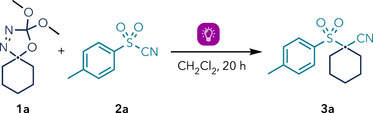		
Entry	Variation of standard conditions^a^	Yield^b^ 3a
1	None	97 (94)^c^
2	EtOAc as solvent	80
3	Mesitylene as solvent	81
4	*^t^* AmOH as solvent	81
5	DMSO as solvent	59
6	450 nm, Ir[dF(CF_3_)ppy]_2_(dtbpy)PF_6_	95^d^
7	110 °C, dark, mesitylene as solvent	Traces
8	Cyclohexane tosylhydrazone + Cs_2_CO_3_ instead of **1a**	n.d.
9	Ethyl diazoacetate instead of **1a**, dark	n.r.

^a^ Standard conditions: **1a** (0.3 mmol, 1.5 equiv), **2a** (0.2 mmol, 1.0 equiv), dry CH_2_Cl_2_ (2 mL), N_2_ atmosphere, 365 nm LED (0.6 W), 25°C, 20 h.

^b^ Yield determined by GC‐FID with toluene as internal standard.

^c^ Isolated yield.

^d^ Yield determined by ^1^H‐NMR with ethylene carbonate as internal standard. n.d. = not detected; n.r. = no reaction.

As described by Gryko and coworkers, dimethoxy oxadiazolines release the active species not only after direct excitation with near‐UV irradiation but also after photosensitization with catalytic Ir[dF(CF3)ppy]2(dtbpy)PF6 under 450 nm irradiation [10]. In our system, using 0.5 mol% catalyst gave yields comparable to those from direct excitation. However, heating the mixture to 110°C without irradiation yields the product only in trace amounts, making a photochemical approach crucial.

As mentioned above, tosylhydrazones were not suitable diazo precursors for the desired transformation. Exchanging **1a** for cyclohexane tosylhydrazone and cesium carbonate leads to the exclusive nucleophilic attack on the nitrile and major formation of *N*‐cyano tosylhydrazone, precluding the desired transformation (see Supporting Information for details). Furthermore, stabilized diazo compounds (such as ethyl diazoacetate) lack the requisite nucleophilicity to engage in productive reactions, underscoring the necessity of donor–donor moieties for this transformation.

**FIGURE 2:**
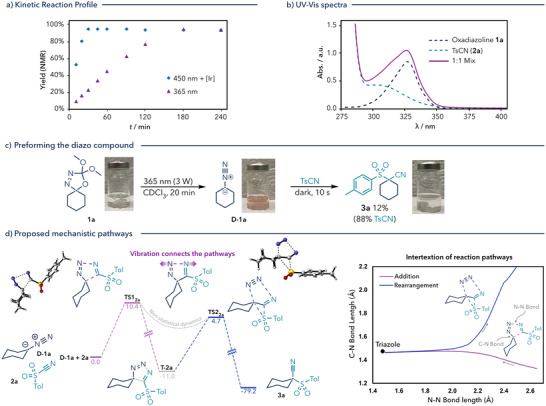
Mechanistic investigations. (a) Kinetic profile of the reaction with and without photosensitizer followed by ^1^H NMR spectroscopy. Conditions: **1a** (300 µmol, 1.5 equiv), **2a** (200 µmol, 1.0 equiv), CDCl_3_ (2 mL), [Ir] = Ir[dF(CF_3_)ppy]_2_(dtbpy)PF_6_ (0.5 mol%), ethylene carbonate as internal standard. (b) UV–vis spectrum in CH_2_Cl_2_ of oxadiazoline **1a**, tosyl cyanide (**2a**), and a 1:1 mixture of them. (c) Pre‐formation of diazo compound (pink) followed by addition of tosyl cyanide in the dark leads to rapid discoloration and product formation. (d) Reaction profile calculated in (CPCM:CH_2_Cl_2_)B3LYP‐D3BJ/def2SVP level of theory. All the free energies are in kcal mol^−1^.

After exploring tolerated reaction conditions, we started the mechanistic investigation by measuring the kinetic profile (Figure 2a). Upon irradiation with a 365 nm LED, the reaction proceeds linearly for approximately 90 min, reaching a 65% yield, after which the rate slightly decreases before achieving full conversion with nearly quantitative yield after 3 h. In contrast, employing a photosensitized system dramatically alters the kinetic behavior: full conversion is achieved within 30 min using only 0.5 mol% of the Ir‐based photocatalyst. Despite the significantly enhanced reaction rate under sensitized conditions, we opted to proceed with direct excitation in the absence of the catalyst to maintain a simpler reaction system.

Given the established reactivity of tosyl cyanide (**2a**) not only in electrophilic but also radical nitrile transfers [27], we sought to exclude the possibility of a photoinitiated radical reaction pathway. To obtain a clearer picture, we pre‐formed the presumed cyclohexane diazo intermediate by irradiating oxadiazoline **1a** (in the absence of tosyl cyanide) with a 3 W, 365 nm LED for 20 min. This treatment yielded a pink solution, a color characteristic of dialkyl diazo compounds (Figure 2c) [9, 33]. Subsequent addition of tosyl cyanide to the diazo‐containing solution, without further irradiation, led to rapid discoloration and gas evolution, likely nitrogen release. ^1^H‐NMR analysis revealed 12% conversion of tosyl cyanide to the target product, with 88% remaining unreacted. Although the low conversion suggests the diazo compound is present at low concentration under these conditions, either due to slow formation by direct excitation or decomposition of the diazo species at 25 °C, it demonstrates that the diazo compound is indeed the reactive species in this reaction. Photochemical activation appears necessary only for its generation.

The computational investigation of the reaction profile, performed at the B3LYP‐D3BJ/def2‐SVP (see SI for technical details) level of theory with CPCM (CH_2_Cl_2_) solvation, begins with the diazo compound **D‐1a** and the cyanide source **2a** as the photoinitiation, leading to the formation of **D‐1a,** which was found to have no experimentally detectable influence on the subsequent formation of the final product **3a**.

The overall mechanism comprises two elementary steps (Figure 2d) and commences with a (3+2) cycloaddition between **2a** and **D‐1a** via **TS1**. This step proceeds via a low activation barrier of 10.4 kcal·mol^−^^1^, leading to the triazole intermediate **T‐2a**, which is formed in a downhill process (Δ_R_ *G*  = −11.0 kcal·mol^−^^1^). The subsequent step involves loss of N_2_ accompanied by migration of the sulfonyl group, for which a single transition state, **TS2,** could be located. The associated barrier of 15.7 kcal·mol^−^^1^ corresponds to a half‐life of **T‐2a** of approximately 0.04 s at 298 K, and is in excellent agreement with the experimentally observed rapid product formation. Moreover, NMR spectroscopy at 248 K confirmed the existence of an intermediate involved in the reaction process. The overall transformation is strongly exergonic (Δ_R_ *G* = −79.2 kcal·mol^−^^1^), which can be attributed in part to the inherent instability of **D‐1a** and the release of dinitrogen.

An intriguing feature emerged from the analysis of the imaginary normal modes of **TS1** and **TS2**, which describe the respective reaction paths. In both transition states, the N–N stretching motion (Figure 2) contributes significantly to the reaction coordinate, suggesting that—given the topology of the potential energy surface—fractions of the reacting ensemble may deviate from the minimum energy path and proceed directly to the product under non‐statistical dynamic conditions. This implies that the intermediate **T‐2a** may not fully equilibrate thermally. Instead, due to the coupling of the N–N‐stretching modes associated with both elementary steps, the reaction proceeds via an effectively lower barrier to form **3a**.

**SCHEME 1:**
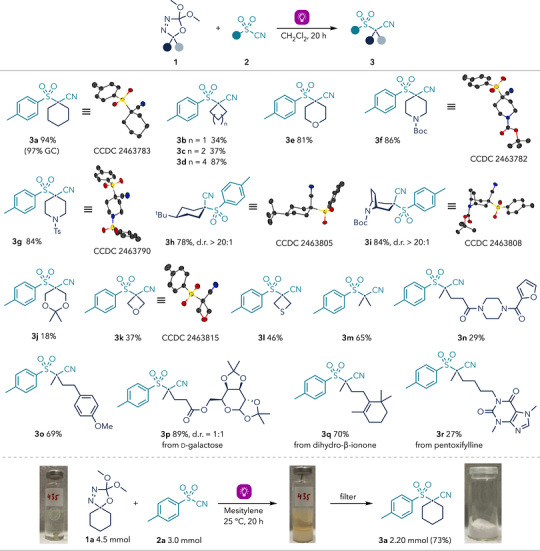
Substrate scope of oxadiazolines in the formal bond insertion. Reaction conditions: 1 (0.3 mmol, 1.5 equiv), 2 (0.2 mmol, 1.0 equiv) in CH_2_Cl_2_ (2 mL, 0.1 m), irradiation with a 365 nm LED (0.6 W) for 20 h at 25°C under N_2_ atmosphere. Yields of isolated products are given unless stated otherwise [34] GC‐yield was determined with toluene as an internal standard. Scale‐up: Reaction performed in mesitylene (7.5 mL, 0.4 m) and open to air.

With optimized conditions in hand, we investigated the scope of the discovered reaction. We began by exploring the range of oxadiazolines applicable with commercially available tosyl cyanide **2a** as a model substrate (Scheme 1). Varying the ring size of the ketone precursor from four to seven showed a decrease in yield for four and five‐membered rings (**3b** and **3c**), though a very good yield was obtained for an increased ring size (**3d**). When employing six‐membered heterocycles, the products can be isolated in very good yields for tetrahydropyran (**3e**), as well as Boc and tosyl‐protected piperidine (**3f** and **3g**). When adding a conformational anchor, such as a *tert*‐butyl group to cyclohexane, good yields and excellent diastereoselectivity can be observed (**3h**). Equally good selectivity was achieved when a bridged piperidine system (**3i**) was employed with negligible loss in yield compared to the non‐bridged analogue. Both configurations are supported by single‐crystal x‐ray structures. A 1,3‐dioxane could also be employed, but with a noticeably lower yield, which is in alignment with observations by the Gryko group (**3j**) [10]. Moving from six‐membered ring systems back to smaller rings, oxetane and thietane rings were successfully installed with moderate yields (**3k** and **3l**).

Importantly, this reaction not only promotes the installation of ring structures, but also of non‐cyclic moieties. *Gem*‐dimethyl groups can easily be incorporated with the oxadiazoline generated from acetone in good yields (**3m**), with similar yields for more complex nonsymmetric substrates, including an anisole motif (**3o**), a protected d‐galactose (**3p**), and double bonds in dihydro‐β‐ionone (**3q**), all in good yields. When further including late‐stage functionalization involving a piperazine and furane ring (**3n**), or a pentoxifylline‐analogue (**3r**), the products can still be generated, however, in diminished yields of 29% and 27%, respectively, due to the formation of multiple side products. We also leveraged the broad solvent compatibility and concentration independence to scale the reaction between **1a** and **2a** up to 3 mmol in mesitylene as the solvent. After 20 h of irradiation, the precipitate was simply filtered off and washed to give the desired product in 73% yield (Scheme 1, lower part). To avoid pressure buildup, the reaction was kept open to air, with no noticeable formation of byproducts.

**SCHEME 2:**
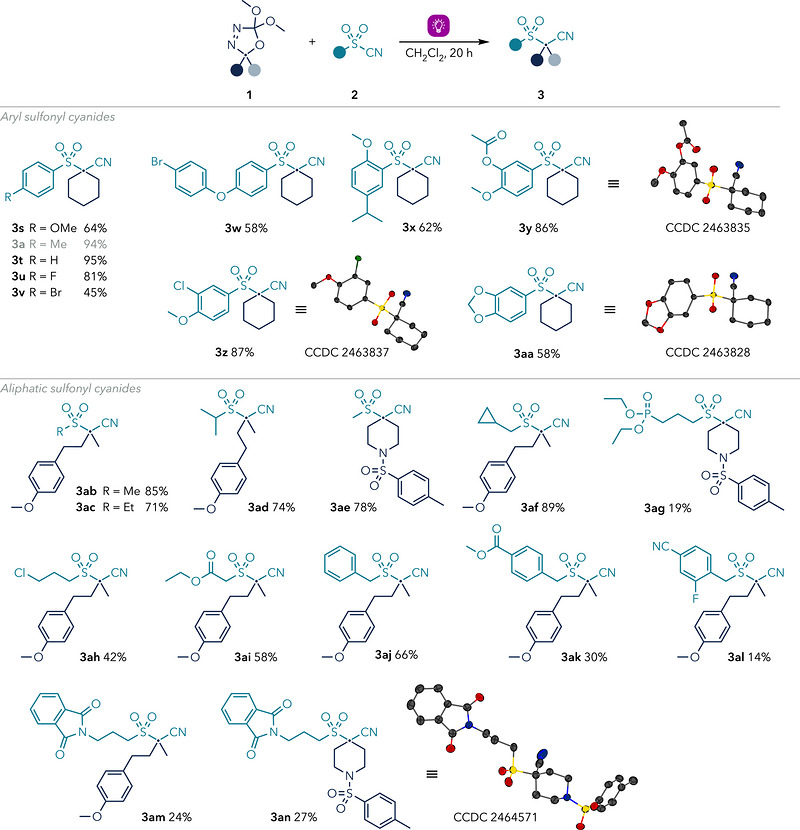
Substrate scope of sulfonyl cyanides in the formal bond insertion. Reaction conditions: **1** (0.3 mmol, 1.5 equiv), **2** (0.2 mmol, 1.0 equiv) in CH_2_Cl_2_ (2 mL, 0.1 m), irradiation with a 365 nm LED (0.6 W) for 20 h at 25°C under N_2_ atmosphere. Yields of isolated products are given unless stated otherwise [34].

Following the successful functionalization of diverse motifs with tosyl cyanide (**2a**), we next investigated the scope of sulfonyl cyanides compatible with the reaction conditions (Scheme 2). We began by examining aryl‐substituted sulfonyl cyanides, where para‐substituted, electronically neutral groups such as hydrogen and fluorine (**3t** and **3u**) furnished the desired products in excellent yields of 81% and 95%, respectively. Electron‐donating (‐OMe, **3s**) and electron‐withdrawing (‐Br, **3v**) groups were also tolerated, albeit with somewhat diminished yields of 64% and 45%, respectively.

Encouraged by these results, we extended the study to more complex anisole derivatives (**3w**‐**3aa**). A biaryl ether was successfully incorporated into product **3w** in 58% yield, and aryl systems bearing two electron‐donating substituents yielded **3x** and **3aa** without further loss in yield. Notably, aryl substrates bearing both electron‐donating and electron‐withdrawing groups led to improved yields of 86% (**3y**) and 87% (**3z**), supporting the observation that electronically neutral sulfonyl cyanides provide optimal results.

To further expand the substrate scope, we sought to incorporate pharmaceutically relevant heterocycles. However, this was limited by the synthetic accessibility of the corresponding sulfonyl cyanides (starting material synthesis). The standard oxidation of thiocyanates with trifluoroperacetic acid was incompatible with sensitive heterocycles, and a reported alternative using sulfinates and cyanogen chloride was avoided due to safety concerns [35].

We next evaluated whether aliphatic sulfonyl cyanides would undergo similar reactivity. Incorporation of small aliphatic sulfonyl groups such as methyl (**3ab** and **3ae**), ethyl (**3ac**), isopropyl (**3ad**), and cyclopropylmethyl (**3af**) proceeded efficiently, affording products in yields ranging from 71% to 89% in acyclic and cyclic substrates. In contrast, functionalized aliphatic chains containing diethyl phosphonate (**3ag**), chloro (**3ah**), ester (**3ai**), or phthalimide (**3am** and **3an**) substituents led to reduced yields (24%–58%). A similar trend was observed for benzylic sulfonyl cyanides: the unsubstituted benzyl derivative **3aj** was obtained in 66% yield, while arene‐substituted analogues (**3ak**, **3al**) suffered from a notable yield decrease.

**SCHEME 3:**
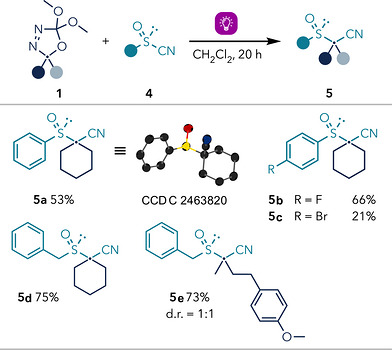
Substrate scope of sulfinyl cyanides in the formal bond insertion. Reaction conditions: **1** (0.3 mmol, 1.5 equiv), **4** (0.2 mmol, 1.0 equiv) in CH_2_Cl_2_ (2 mL, 0.1 m), irradiation with a 365 nm LED (0.6 W) for 20 h at 25°C under N_2_ atmosphere. Yields of isolated products are given unless stated otherwise [34].

The oxidation protocol of thiocyanates with trifluoroperacetic acid for the synthesis of sulfonyl cyanides **2** was modified to yield the challenging‐to‐obtain sulfinyl cyanides **4** (partial oxidation) by reducing reaction temperatures and times to limit overoxidation [36, 37, 38]. These species proved to be valuable substrates, enabling access to sulfoxide‐containing products (Scheme 3). This tolerated both aryl groups with electron‐neutral (‐H, **5a**; ‐F, **5b**), and poor (‐Br, **5c**) substituents, with yields up to 66%, as well as an aliphatic substituent yielding 75% of **5d**. We also investigated whether the sulfur‐centered stereocenter could induce diastereoselectivity in reactions with unsymmetrical oxadiazolines. While product **5e** was obtained in 73% yield, no diastereomeric preference was observed.

Over the past decade, continuous‐flow technology has emerged as a powerful tool across various branches of chemistry, largely due to its enhanced control over reaction parameters, improved scalability, and increased safety [39, 40, 41]. It is a key pillar in the synthesis of active pharmaceutical ingredients in automated and modular systems [42]. In the context of photochemistry, the typically small reactor volumes enhance irradiation efficiency while improving safety for handling of unstable or potentially explosive intermediates. These advantages have led to the widespread use of flow chemistry for the generation of diazo compounds from hydrazones [9] and oxadiazolines [43]. To further broaden the condition space of the herein presented reaction toward protocols highly important to the pharmaceutical industry, we sought to explore the application of this transformation under continuous‐flow conditions.

We started our investigation by again utilizing the broad solvent compatibility and switched from dichloromethane to ethyl acetate (Table 1), as it offers drastically lower safety, health, and environmental (SHE) impact at the cost of a minimal decrease in yield. Excitingly, the reaction behaved similarly to batch conditions, allowing us to exploit previously observed kinetics to shorten residence time and improve efficiency. Using 0.5 mol% Ir‐catalyst and 450 nm irradiation, full conversion was achieved within 64 min, with minimal yield increase beyond 48 min. Increasing the oxadiazoline excess to 2.0 equiv improved the yield, affording 88% of **3a** under optimized conditions (LCMS yield; see Supporting Information).

**FIGURE 3:**
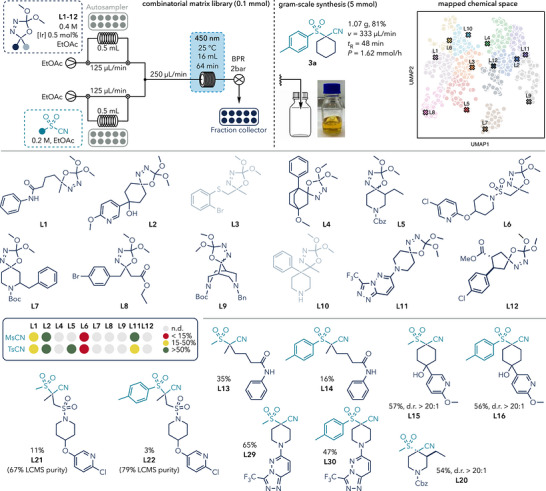
Application of the C─S bond insertion to construct a 10x2 combinatorial matrix library under flow‐chemical conditions. *v*  =  flow rate, *t*_R_  =  residence time, *P*  =  productivity, n.d. = not detected. Reaction conditions: **L1‐12** (0.2 mmol, 2.0 equiv), MsCN or TsCN (0.1 mmol, 1.0 equiv). Collection: 3 mL pre‐ and 8 mL post‐slug. Isolations were performed on a 50 µmol scale, and those yields were reported.

The successful transfer of the optimized batch synthesis to flow reactor conditions inspired us to utilize the discovered reaction for synthesizing a diverse compound library from drug‐like diazo precursors in flow, a typical scenario in the DMTA cycle for drug development (Figure 3). To select a representative set of compounds, we designed a 2D chemical space map of publicly available ketones (starting materials for the diazo precursor synthesis) available from the AstraZeneca compound collection, as presented by Glorius and coworkers [44, 45, 46, 47]. The generated chemical space was divided into 12 clusters from which the molecules with the highest quantitative estimate of drug‐likeness (QED) were selected. This approach allowed us to ensure chemical diversity while focusing on pharmaceutically relevant compounds at the same time. Out of the 12 compounds selected, we successfully obtained 10 oxadiazoline precursors. The synthesis of oxadiazolines **L3** and **L10** was unsuccessful, likely due to the presence of functional groups incompatible with oxidation by hypervalent iodine reagents. The successfully obtained structures include, for example, nitrogen‐rich aromatic systems (**L2**, **L6**, and **L11**) or piperidine‐containing scaffolds (**L5**‐**L7** and **L11**). In a combinatorial matrix library, these were paired with tosyl and mesyl cyanide as representative electrophiles.

We were delighted to obtain products containing a phenyl amide (**L13** and **L14**) in acceptable yields for standard medicinal chemistry synthesis (>10%), a pyridine and free alcohol in (**L15** and **L16**) in good yields of above 50%, and excellent diastereoselectivity. This result is particularly noteworthy, as it highlights a distinct advantage of diazo compounds over free carbenes, which can favor O–H insertion as a reaction pathway [12].

Consistent with previous batch experiments, substrates bearing piperidine rings also furnished the desired products (**L20**, **L29**, and **L30**) in good yields. Remarkably, for the α‐ethyl‐substituted substrate, we observed excellent diastereoselectivity in **L20**.

Unexpectedly, no product formation was observed for the β‐benzyl‐substituted substrate **L7**, as determined by LC‐MS analysis. Substrates containing highly sterically hindered ring systems (**L4** and **L9**) and those with electron‐withdrawing groups in close proximity to the reactive site (**L6** and **L8**) also proved unreactive or led to negligible product formation. Additionally, a substituted cyclopentane substrate (**L12**) failed to yield detectable products, mirroring the low efficiency observed with the corresponding unsubstituted analog.

To further demonstrate the scalability and practicality of the developed method, we leveraged one of the biggest advantages of flow chemistry over batch and performed a scaled‐up reaction. Using the previously optimized conditions and reducing the residence time to 48 min resulted in the formation of 1.07 g (81%) of **3a** over 2.5 h, corresponding to 1.62 mmol/h.

## 3 Conclusion

We have developed a new strategy for the *gem*‐difunctionalization of ketones via photogenerated unstabilized diazo compounds using sulfonyl cyanides, enabled by a migratory 1,2‐sulfone shift. This transformation proceeds under mild conditions and is compatible with various solvents, air, and moisture, thereby making it operationally simple and broadly applicable. It exhibits broad functional group tolerance, enabling the incorporation of a wide array of structural motifs, including small rings, bridged bicyclic systems, and both aromatic and aliphatic sulfones, with excellent diastereoselectivity in cyclic frameworks. Moreover, the identification and rationalization of certain functional group limitations provide a clear reactivity profile, allowing the reaction to be applied predictively and selectively to suitable substrates.

Beyond sulfonyl cyanides, we have shown that sulfinyl cyanides undergo analogous reactivity, thereby expanding the scope and modularity of this methodology. To demonstrate its synthetic potential, we designed and carried out a chemical library synthesis in continuous flow, guided by a chemical space map to enable rational substrate selection and maximize structural diversity. This continuous‐flow implementation also demonstrates the reaction's scalability, including gram‐scale synthesis under operationally simple conditions.

The mechanism of the transformation was investigated using DFT calculations, supported by low‐temperature NMR experiments, revealing key features of a merged cycloaddition‐1,2‐migration reaction pathway.

This work establishes a new disconnection strategy for ketone functionalization that leverages the unique reactivity of unstabilized diazo intermediates. By strategically employing an operationally simple diazo generation protocol, we establish a platform for selective geminal difunctionalization, thereby expanding the synthetic toolbox for the rapid assembly of complex, densely functionalized molecules. We anticipate that this strategy will inspire further exploration of electrophilic partners and facilitate applications in late‐stage functionalization, medicinal chemistry, and diversity‐oriented synthesis.

## Author Contributions

**Vincent George** and **Burkhard König** conceptualized the project. **Vincent George**, **Daniel Maddox**, **Giovanna Mejia**, **Simon O. Angerer**, **Maria Schmoll**, and **Lisa Marie Schneider** conducted experimental investigations. **Aryaman Pattanaik** and **Lukas M. Sigmund** conducted the computational work. The manuscript was written with input from all authors, and all authors agreed with it.

## Conflicts of Interest

The authors declare no conflicts of interest.

## Supporting information




**Supporting File**: anie72356‐sup‐0001‐SuppMat.pdf.

## Data Availability

Raw analytical data are available free of charge at the Radar4Chem repository under https://www.radar‐service.eu/radar/de/dataset/aavfxp6aajpvqgkb.
