# Supplementary material for: Geminal Difunctionalization of Ketones via C─S Bond Insertion of Photogenerated Donor–Donor Diazo Compounds
Source: Angew Chem Int Ed Engl. 2026 May 2;65(25):e6162809. doi: 10.1002/anie.6162809 (PMC13266950; doi:10.1002/anie.6162809)
Supplement: Supplementary file 1 — Supporting File: anie72356‐sup‐0001‐SuppMat.pdf. [file ANIE-65-e6162809-s001.pdf]

Supporting Information

Geminal Difunctionalization of Ketones via C–S Bond Insertion of Photogenerated Donor–Donor Diazo Compounds

Vincent George[1,2], Aryaman Pattanaik[1], Daniel Maddox[2], Lukas M. Sigmund[3], Giovanna Mejia[2], Sara Pahlén[2], Simon Angerer[1], Maria Schmoll[1], Lisa Marie Schneider[1], Nidhal Selmi[2], Mikhail Kabeshov[3], Giulia Bergonzini[2], Julia Rehbein[1], Burkhard König[1]*

[1] Faculty of Chemistry and Pharmacy, University Regensburg, 93040 Regensburg, Germany
[2] Compound Synthesis and Management, Discovery Sciences, BioPharmaceuticals R&D, AstraZeneca, Gothenburg, 431 83 Mölndal, Sweden
[3] Molecular AI, Discovery Sciences, BioPharmaceuticals R&D, AstraZeneca, 431 83 Mölndal, Sweden.

*burkhard.koenig@ur.de

Table of Contents

1 General Information 3

2 Optimization & Control Experiments 5

2.1 Optimization 5

2.2 Control Experiments 7

2.3 Reaction Kinetics 8

2.4 Flow Optimization 10

3 Mechanistic Investigations 12

3.1 UV-Vis 12

3.2 Pre-Forming Experiments 12

3.3 Computational Studies 14

3.4 Optimized coordinates 14

4 Chemical Space Map 20

5 Synthesis of starting materials 21

5.1 Ketones 22

5.2 Oxadiazolines 24

5.3 Aryl sulfonyl and sulfinyl cyanides 33

5.4 Alkyl sulfonyl and sulfinyl cyanides 40

6 Synthesis of Products 46

6.1 Oxadiazoline Scope 46

6.2 Aryl sulfonyl cyanide scope 52

6.3 Aliphatic sulfonyl cyanide scope 55

6.4 Sulfinyl cyanide scope 59

6.5 Matrix Library Scope 61

7 X-ray crystal structures 64

8 Literature 76

9 NMR Spectra 78

1 General Information

Reagents and solvents: Commercially available starting materials and solvents were used without further purification. Reactions were carried out in dry solvents under nitrogen atmosphere unless otherwise stated.

NMR spectroscopy: All NMR spectra were recorded at room temperature using a Bruker Avance 300 (300 MHz for ^{1}H, 75 MHz for ^{13}C), a Bruker Avance 400 (400 MHz for ^{1}H, 101 MHz for ^{13}C), or a Bruker Avance 600 (600 MHz for ^{1}H, 150 MHz for ^{13}C) NMR spectrometer. All chemical shifts are reported in δ-scale as parts per million [ppm] (multiplicity, coupling constant *J*, number of protons) relative to the solvent residual peaks as the internal standard. Coupling constants *J* are given in Hertz [Hz]. Abbreviations used for signal multiplicity: ^{1}H-NMR: br = broad, s = singlet, d = doublet, t = triplet, q = quartet, dd = doublet of doublets, dt = doublet of triplets, and m = multiplet.

Mass spectrometry: High resolution mass spectra (HRMS) were obtained from the central analytic mass spectrometry facilities of the Faculty of Chemistry and Pharmacy, Regensburg University, and are reported according to the IUPAC recommendations 2013. All mass spectra were recorded on a Finnigan MAT 95, Thermo Quest Finnigan TSQ 7000, Finnigan MATSSQ 710 A or an Agilent Q-TOF 6540 UHD instrument.

UV-Vis spectroscopy: UV–Vis measurements were performed with a Varian Cary 100 UV/Vis spectrophotometer.

X-Ray diffraction: X-Ray analysis was performed by the crystallography department of the University of Regensburg. The structures were solved by Birgit Hischa (University of Regensburg) and Sabine Stempfhuber (University of Regensburg).

Gas chromatography: GC measurements were performed on a GC 7890 from Agilent Technologies. Data acquisition and evaluation was done with Agilent ChemStation Rev.C.01.04.

Thin layer chromatography: Analytical TLC was performed on silica gel coated alumina plates (MN TLC sheets ALUGRAM® Xtra SIL G/UV254). Visualization was done by UV light (254 or 366 nm). If necessary, potassium permanganate was used for chemical staining.

Flash column chromatography: Purification by column chromatography was performed with silica gel 60 M (40-63 µm, 230-440 mesh, Merck) on a Biotage® Selekt or Biotage® Selekt Enkel device.

LED Setup: Photochemical reactions were performed with 365 nm LEDs (SSC VIOSYS UV 365nm CUN66A1B (*λ* = 365 nm (± 15 nm), 3.6 V, 700 mA, 600 µW optical power). The sample was irradiated with a LED through the vial's plane bottom side and cooled from the side using custom-made aluminum cooling blocks connected to a thermostat (Figure S1).

Flow Setup: Syrris ASIA system equipped with 2 syringe pumps, automated reagent injector module (AutoRIM) with total of 2 automated injectors, 1 coil reactor (16 mL) for photochemistry with CryoController module for temperature control, backpressure regulator (BPR) and an automated collector. Temperature was kept at 25.0±0.1 °C at 20 W LED power and 25.0±1.0 °C at 40 W LED power.

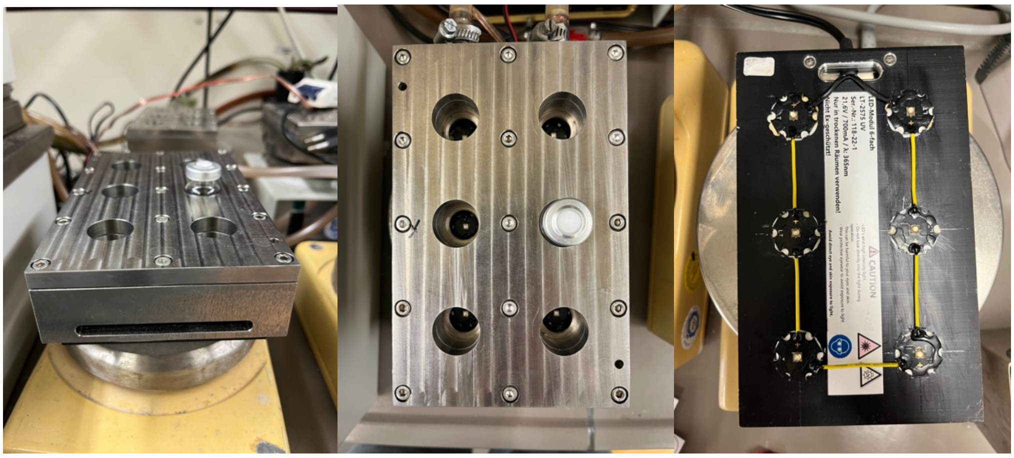

Figure S1. Photochemical set-up for regular-scale reactions.

2 Optimization & Control Experiments

2.1 Optimization

2.1.1 Solvent Screen

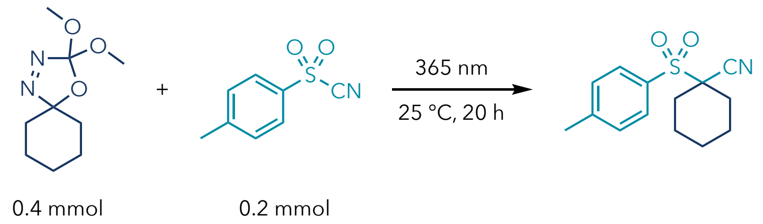

A 5 mL crimp vial equipped with tosyl cyanide (0.2 mmol, 1.0 eq.) and oxadiazoline (0.4 mmol, 2.0 eq.) was evacuated and filled back with N_2 three times. Then, dry solvent (2 mL) was added and the mixture irradiated with 365 nm for 20 h. Then, PhMe (20 µL) was added by syringe and the mixture analyzed by GC-FID.

When no solvent or water was used, workup with CH_2Cl_2 was performed. When mesitylene was used, EtOAc (1 mL) was added to dissolve the precipitate. When PhMe was used, EtOAc (0.5 mL) was added to dissolve the precipitate. When iPrOH or tert-amyl alcohol (TAA) were used, CH_2Cl_2 (2 mL) was added to dissolve the precipitate.

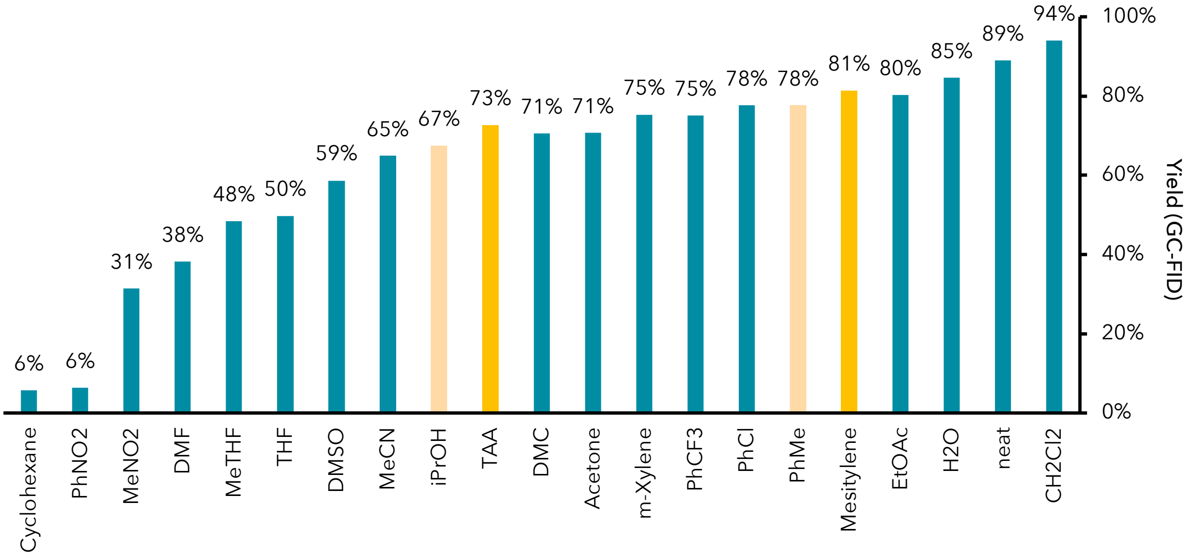

Figure S2. Screening of solvents for the formal C-S bond insertion reaction. Yields determined with PhMe as internal standard. In mesitylene and *tert*-amyl alcohol (TAA), major formation of precipitate was observed. In *iso*-propanol and toluene, small amounts of precipitate was observed.

2.1.2 Concentration Screen

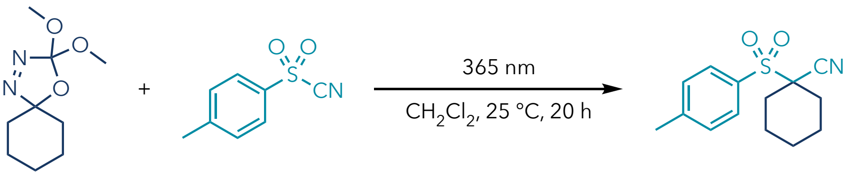

A 5 mL crimp vial equipped with tosyl cyanide (0.2 mmol, 1.0 eq.) and oxadiazoline (0.4 mmol, 2.0 eq.) was evacuated and filled back with N_2 three times. Then, dry CH_2Cl_2 was added and the mixture irradiated with 365 nm for 20 h. Then, the volume was brought to 2 mL with CH_2Cl_2, PhMe (20 µL) added by syringe and the mixture analyzed by GC-FID.

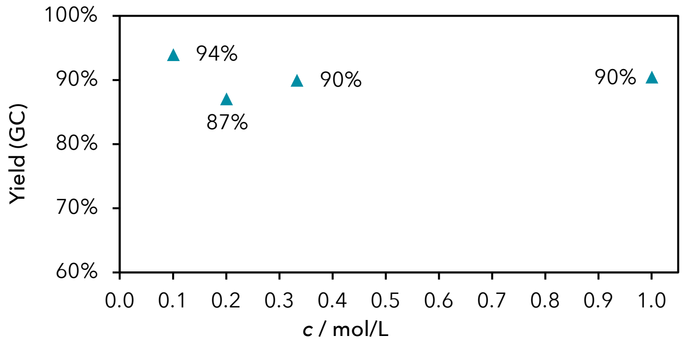

Figure S3. Screening of different concentrations. Yields determined with PhMe as internal standard.

2.1.3 Equivalent Screen

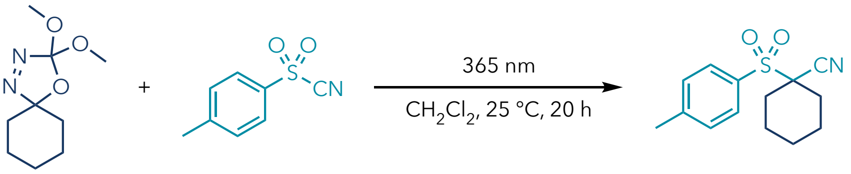

A 5 mL crimp vial equipped with tosyl cyanide (0.2 mmol, 1.0 eq.) and oxadiazoline was evacuated and filled back with N_2 three times. Then, dry CH_2Cl_2 (2 mL) was added and the mixture irradiated with 365 nm for 20 h. Then, PhMe (20 µL) was added by syringe and the mixture analyzed by GC-FID.

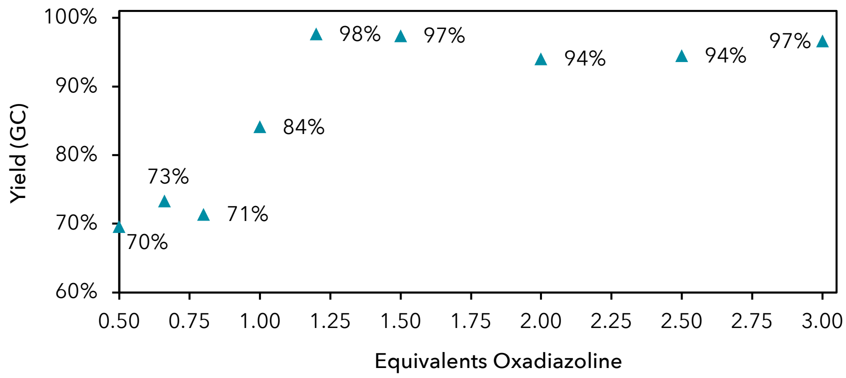

Figure S4. Optimization of oxadiazoline equivalents. Yields determined with PhMe as internal standard.

2.2 Control Experiments

2.2.1 Tosylhydrazones as diazo precursor

Tosylhydrazones are commonly used diazo precursors. However, the under basic conditions required for the diazo formation the competing nucleophilicity does lead to side reactions such as substitution at nitrogen. As this reaction takes place without irradiation, tosylhydrazones also undergo this reaction even before the mixture can be irradiated and can therefore not be used as precursors for this reaction under batch conditions.

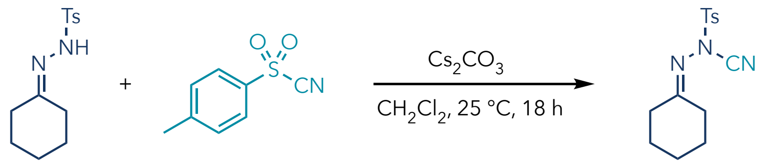

Procedure: Tosyl cyanide (0.2 mmol, 1.0 eq.), cyclohexane tosylhydrazone (0.24 mmol, 1.2 eq.) and cesium carbonate (0.3 mmol, 1.5 eq.) were dissolved in CH_2Cl_2 (2 mL) and stirred for 18 h. The reaction was quenched by addition of water, the phases separated and the aqueous phase washed with CH_2Cl_2 (2x 10 mL). The organic phases were combined, dried over Na_2SO_4 and mixture purified by flash column chromatography (PE/EtOAc 0% → 15%). The product was obtained as colorless oil (54.2 mg, 186 μmol, 93%).

^{1}H NMR (400 MHz, $CDCl_3$) δ = 7.97 – 7.82 (m, 2H), 7.42 (d, *J*=8.3, 2H), 2.62 (t, *J*=6.6, 2H), 2.48 (s, 3H), 2.41 – 2.32 (m, 2H), 1.78 (dtt, *J*=8.4, 6.1, 2.4, 4H), 1.70 – 1.61 (m, 2H).
^{13}C NMR (101 MHz, $CDCl_3$) δ = 184.8, 146.8, 131.6, 130.2, 129.5, 108.6, 35.6, 30.6, 27.2, 26.3, 25.2, 22.0.
The analytical data are in accordance with those reported in the literature.[1]

2.2.2 Stabilized diazo compounds

Diazoacetates are commonly used commercially available stabilized diazo compounds. However, when employed under similar reaction conditions to the optimized procedure, no formation of the desired compound can be observed with most of tosyl cyanide left unreacted (GC/GC-MS). This could be due to the lacking nucleophilic character of stabilized diazo compounds.

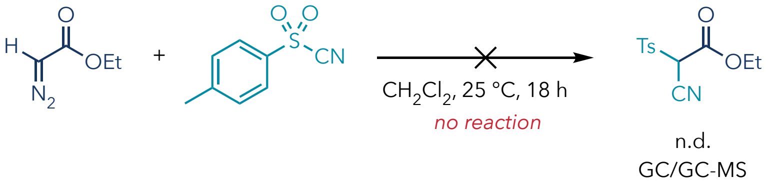

Procedure: A 5 mL crimp vial equipped with tosyl cyanide (0.2 mmol, 1.0 eq.) and ethyl diazoacetate (0.24 mmol, 1.2 eq.). Then, CH_2Cl_2 (2 mL) was added and the mixture stirred at 25 °C for 20 h. Then, the mixture analyzed by GC/GC-MS. No product formation was observed.

2.2.3 Thermal reaction

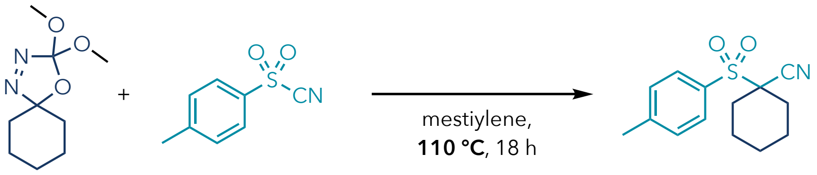

A 5 mL crimp vial equipped with tosyl cyanide (0.2 mmol, 1.0 eq.) and oxadiazoline (0.3 mmol, 1.5 eq.) was evacuated and filled back with N_2 three times. Then, mesitylene (2 mL) was added and the mixture heated to 110 °C for 20 h. No precipitate was observed. Then, PhMe (20 µL) was added by syringe and the mixture analyzed by GC-FID.

2.3 Reaction Kinetics

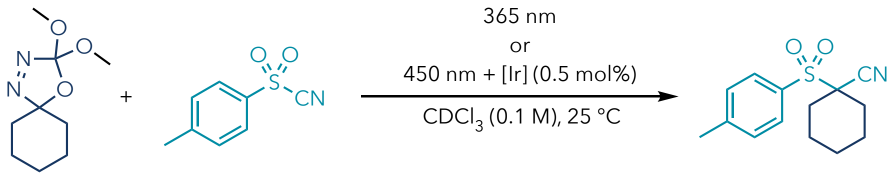

A 5 mL crimp vial equipped with tosyl cyanide (0.2 mmol, 1.0 eq.), oxadiazoline **1a** (0.3 mmol, 1.5 eq.) and ethylene carbonate was evacuated and filled back with N_2 three times. Then, $CDCl_3$ (2 mL) was added by syringe and the solutions stirred at either 365 nm or 450 nm irradiation. 100 µL samples were taken, diluted with 500 µL $CDCl_3$ and analyzed by quantitative ^{1}H-NMR spectroscopy (t1 = 30 s).

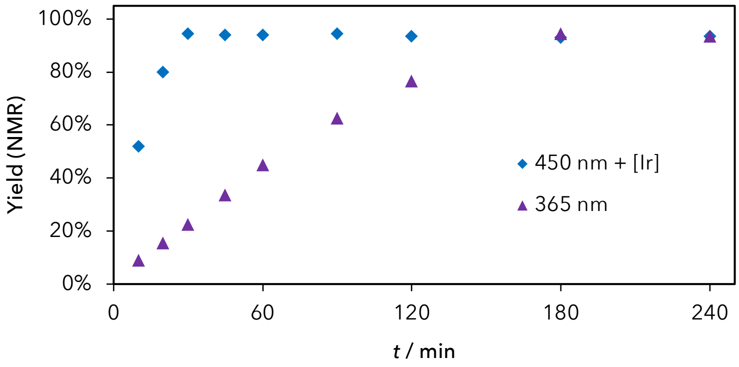

Figure S5. Reaction kinetics under direct excitation with 365 nm irradiation and sensitized with $Ir[dF(CF_3)ppy]_2(dtbpy)PF_6$ (0.5 mol%) and 450 nm irradiation. Yield determined with ethylene carbonate as internal standard.

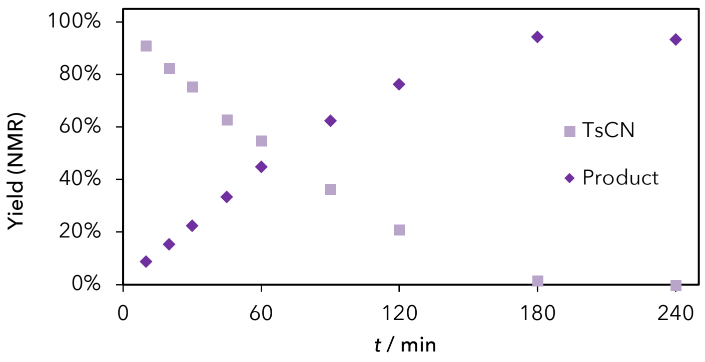

Figure S6. Reaction kinetics under direct excitation with 365 nm irradiation. Yield determined with ethylene carbonate as internal standard.

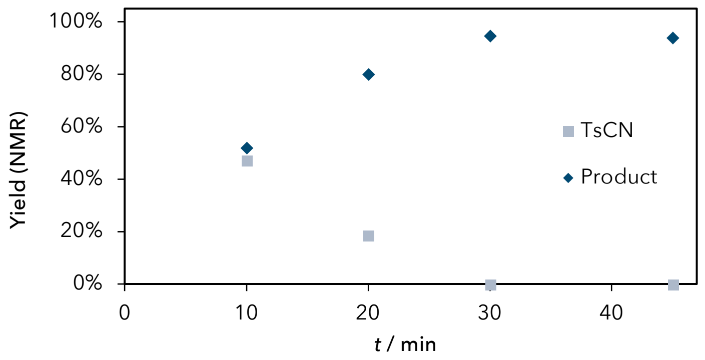

Figure S7. Reaction kinetics sensitized with Ir[dF(CF_3)ppy]$_2$(dtbpy)PF_6 (0.5 mol%) and 450 nm irradiation. Yield determined with ethylene carbonate as internal standard.

2.4 Flow Optimization

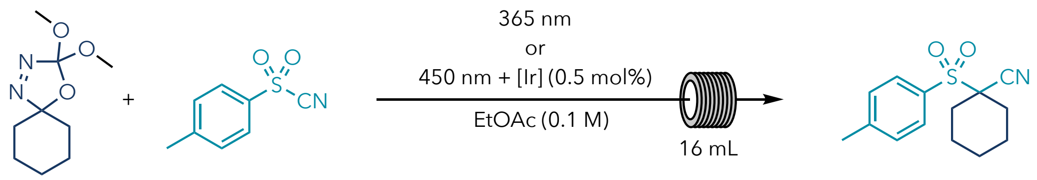

Two stock solutions were prepared in EtOAc: Solution A: Oxadiazoline (0.3, 1.5 eq. or 0.4 M, 2.0 eq.) and $Ir[dF(CF_3)ppy]_2(dtbpy)PF_6$ (0.5 mol%), Solution B: Tosyl cyanide (0.2 M, 1.0 eq.). These were pumped through a 16 mL coiled tube photoreactor at 25 °C and a reaction scale of 0.1 mmol. To the collected crude product, meta-terphenyl (10 µmol, 10 mol%) was added as internal standard, the solvent evaporated in vacuo, the mixture re-dissolved in DMSO and then analyzed by HPLC-MS.

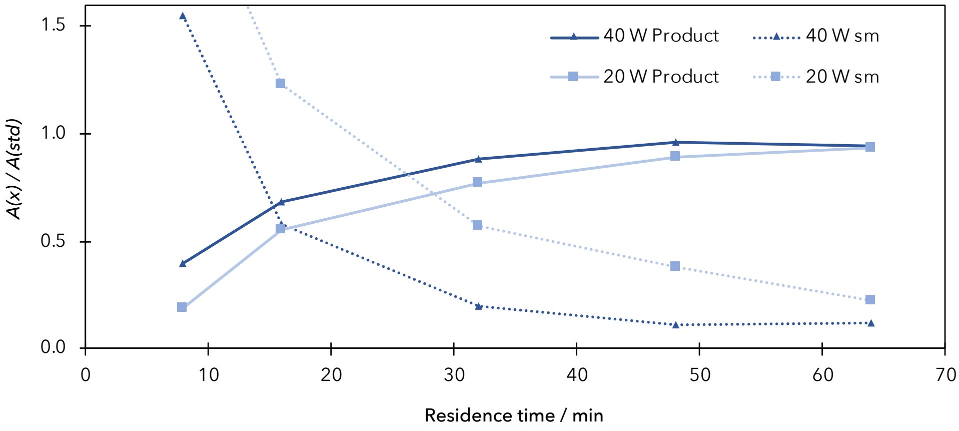

Figure S8. Optimization of photoreactor power. Reaction conditions: Oxadiazoline (1.5 eq.), TsCN (1.0 eq.), 450 nm LEDs. sm = Area of tosyl cyanide and oxadiazoline combined due to overlapping signals.

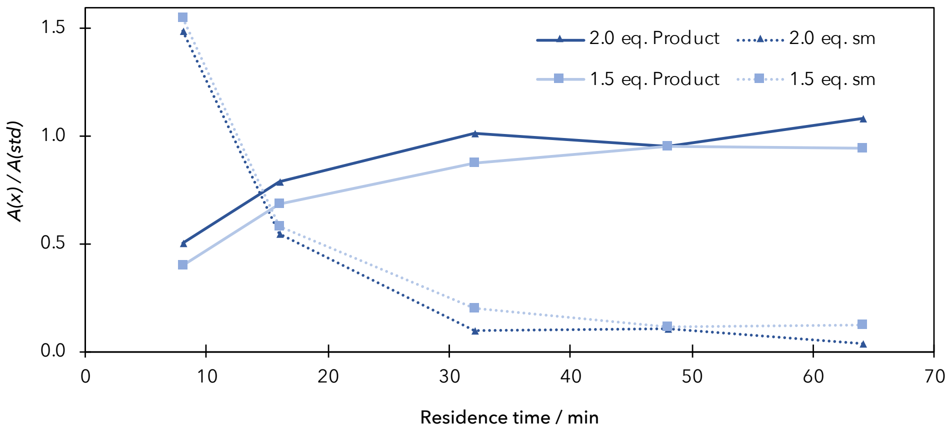

Figure S9. Optimization of oxadiazoline excess. Reaction conditions: Oxadiazoline, TsCN (1.0 eq.), 450 nm LEDs, 40 W. sm = Area of tosyl cyanide and oxadiazoline combined due to overlapping signals.

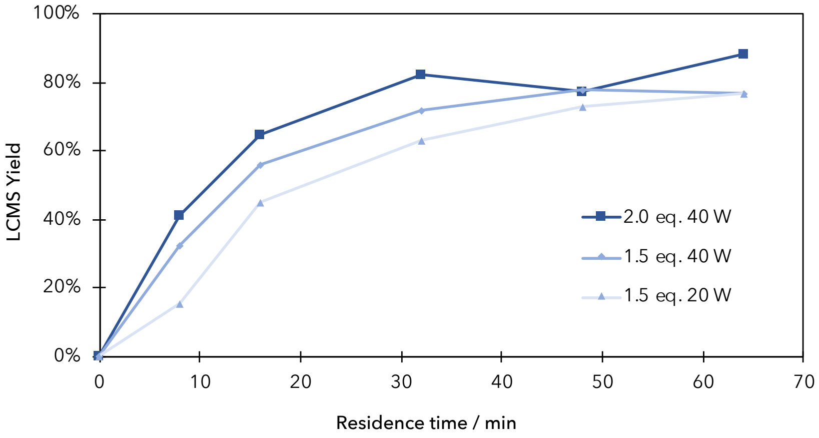

Figure S10. Yield comparison with optimized conditions. Reaction conditions: Oxadiazoline, TsCN (1.0 eq.), 450 nm LEDs. Yields determined with meta-terphenyl as internal standard.

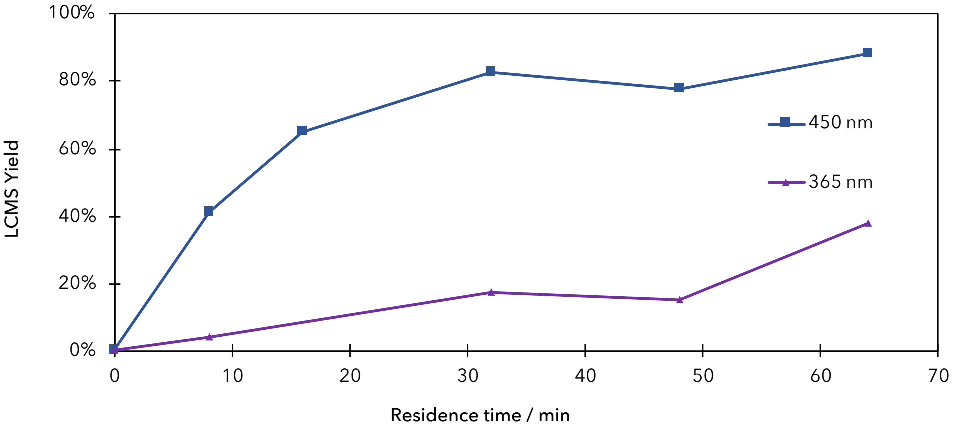

Figure S11. Comparison of sensitized and unsensitized reaction conditions. Reaction conditions: Oxadiazoline (2.0 eq.), TsCN (1.0 eq.), 450 nm or 365 nm LEDs, 40 W. Yields determined with meta-terphenyl as internal standard.

3 Mechanistic Investigations

3.1 UV-Vis

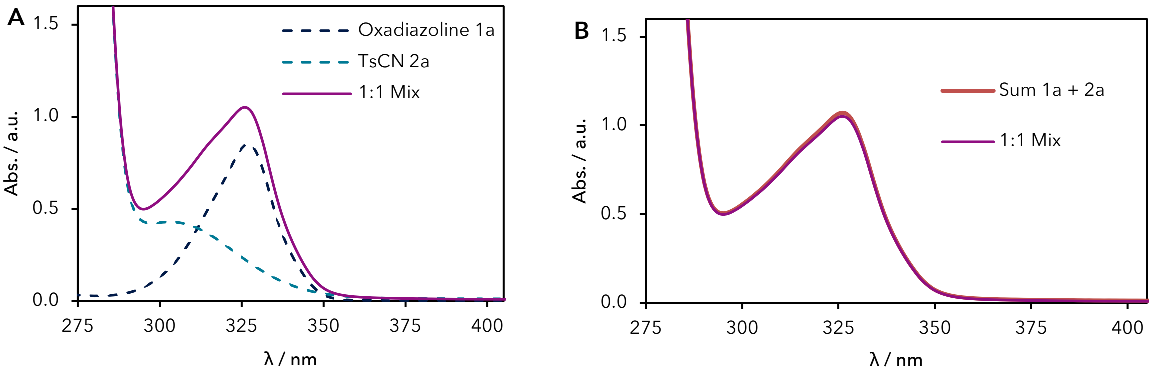

Figure S12. A) UV-Vis spectra of Oxadiazoline 1a, TsCN (2a) and a 1:1 mixture of them. Concentration 0.01 M, CH_2Cl_2 as solvent. **B)** Sum of both solo measurements for **1a** and **2a** compared to a 1:1 mixture of the compounds that is also depicted in A).

3.2 Pre-Forming Experiments

To further elucidate the reaction mechanism, a solution of oxadiazoline **1a** (0.3 mmol, 1.5 mL $CDCl_3$) was irradiated with a 365 nm LED (3 W) for 20 min, affording the cyclohexane diazo compound **D-1a** (Figure S13). Upon addition of a solution of tosyl cyanide (**2a**, 0.2 mmol, 0.5 mL $CDCl_3$) to the resulting characteristic pink diazo solution, rapid discoloration and gas evolution were observed. Analysis by ^{1}H NMR spectroscopy indicated ca. 12% conversion of **2a** to the product **3a**, confirming that light is not required for the subsequent bond insertion step.

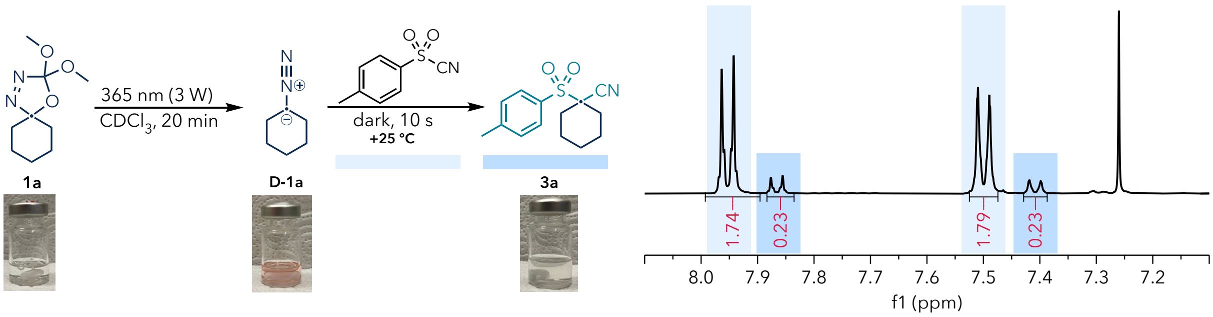

Figure S13. Pre-forming experiment with pictures highlighting the pink color of the diazo compound. Right: Crude 1H-NMR spectrum with highlighted signals corresponding to tosyl cyanide (**2a)** and the product **3a**.

In a complementary experiment, the pre-formed diazo solution **D-1a** was cooled to –25 °C using an *o*-xylene/dry ice bath prior to the dropwise addition of tosyl cyanide (**2a**, 0.2 mmol, 0.5 mL $CDCl_3$), which resulted in an immediate discoloration of the solution. The mixture was then transferred using a pre-cooled syringe into a cooled NMR tube. A ^{1}H-NMR spectrum was acquired at –25 °C (Figure S14A), indicating the conversion of tosyl cyanide **2a** to the desired product **3a**, although additional peaks were observed (highlighted in pink). Upon warming the sample to +25 °C, the new signals disappeared, while the product integral increased, aligning with the sum of **3a** and the unidentified species (Figure S14B). Subsequent re-cooling to –25 °C did not regenerate these

signals, suggesting that they correspond to a transient intermediate that undergoes irreversible decomposition to form **3a**. We attribute these resonances to the elusive triazole **T-2a**, which thermally decomposes under the reaction conditions.

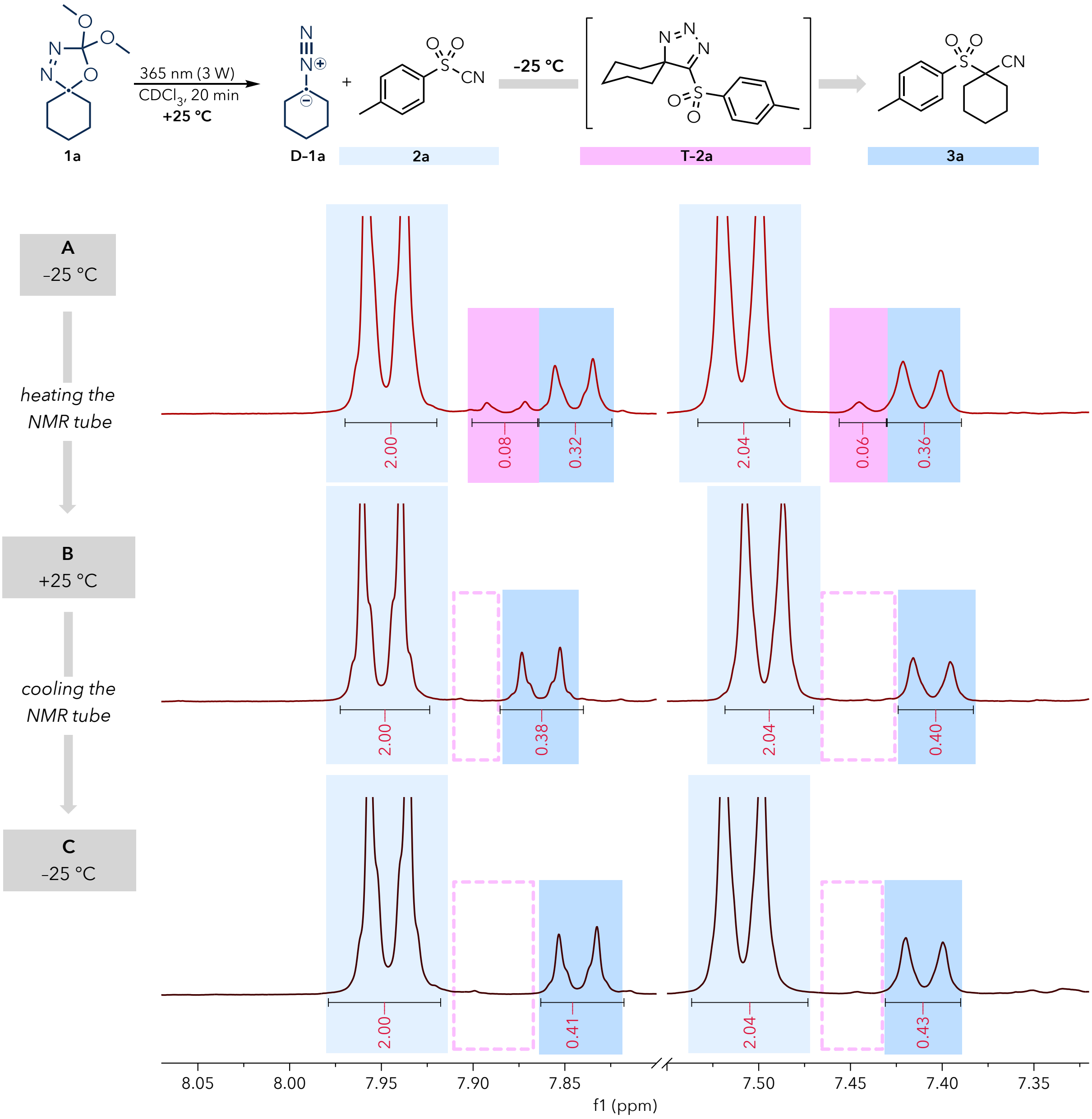

Figure S14. Crude ^{1}H-NMR spectra of the reaction between pre-formed diazo compound **D-1a** and tosyl cyanide **2a** at –25 °C. **A)** ^{1}H-NMR measured at –25 °C. **B)** ^{1}H-NMR after letting the NMR tube heat up to +25 °C. **C)** ^{1}H-NMR after cooling the NMR tube back down to –25 °C.

To substantiate this assignment, DFT calculations of ^{1}H NMR chemical shifts were performed at the (CPCM:Chloroform)B3LYP-D3BJ/PCSSEG-2//(CPCM:CH2Cl2)B3LYP-D3BJ/def2-SVP level of theory (Figure S15). With each progressive step in the reaction, the sulphonyl group becomes less electrophilic as initially it was directly bonded to a *sp*-carbon in cyanide group, then *sp*^{2}-carbon and then finally *sp*^{3}-carbon. Consequently, this is reflected in the DFT-predicted-^{1}H chemical shifts of the aromatic-H of the tolyl group, where the aromatic-H of the triazole intermediate have chemical shifts in between that of Tosyl-CN **2a** and product **3a**. This qualitative

difference in shifts is also seen in NMR spectra of **2a** and **3a** ($\Delta\delta$ ~ 0.4-0.5 vs theoretical). Very faint aromatic protons (confirmed by *J* value in Hz) in between the identified peaks of **2a** and **3a** are further evidence for formation of the proposed triazole-intermediate.

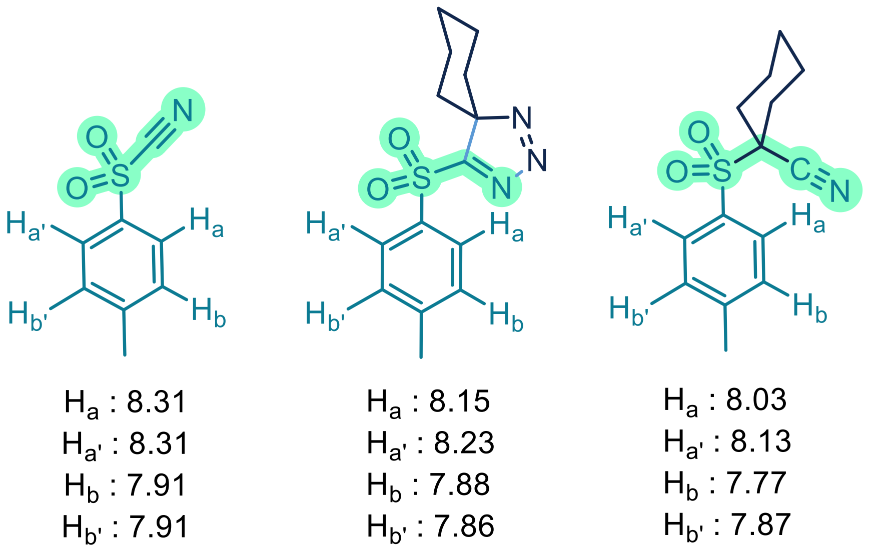

Figure S15. DFT Predicted 1HNMR shifts. Method: (CPCM:Chloroform)B3LYP-D3BJ/PCSSEG-2//(CPCM:CH2Cl2)B3LYP-D3BJ/def2-SVP.

3.3 Computational Studies

Computational studies were performed with the ORCA 5.0.4 program package.[2,3] Geometry optimizations employed the B3LYP-D3BJ functional with the def2-TZVP basis set.[4-7] Solvent effects were included via the CPCM implicit solvation model with dichloromethane (CH_2Cl_2) as the dielectric medium.[8] Transition states were characterized by a single imaginary vibrational frequency and further validated through intrinsic reaction coordinate (IRC) calculations. All reactants and intermediates were confirmed as true minima by the absence of imaginary frequencies.

3.4 Optimized coordinates

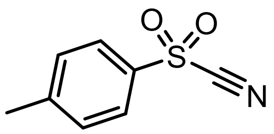

2a

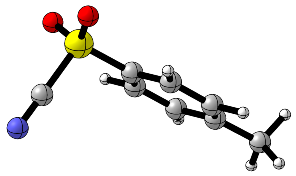

Charge:0, Multiplicity:1

[(CPCM:CH_2Cl_2)B3LYP-D3BJ/def2-TZVP]G_{298K}: -912.22980138

[(CPCM:CH_2Cl_2)B3LYP-D3BJ/def2-TZVP]H_{298K}: -912.18036046

C	1.87540908505474	0.78726557672996	-0.32900245440228
N	1.52006209237591	1.86760580043156	-0.50663637933339
S	2.41387567430576	-0.88132482591886	-0.04751706231352

O	2.38649989894072	-1.04197988975402	1.37815703695950
O	1.58512018335696	-1.68449524987885	-0.90026433566313
C	4.05266847769136	-0.83469555456450	-0.63709560347233
C	4.29968458610223	-1.11898157687943	-1.97896399575175
C	5.08214282296668	-0.50006040104196	0.24092574649286
C	5.60418341589266	-1.07175641511193	-2.43806505662417
H	3.48659859684111	-1.37350171029130	-2.64386268176371
C	6.37847619163893	-0.46006357174830	-0.24145561795134
H	4.86878564477067	-0.28041780054465	1.27735510397013
C	6.66014698415133	-0.74482859655497	-1.58143115754186
H	5.80886755325963	-1.29101684712391	-3.47816255090881
H	7.18706179822966	-0.20277884335514	0.43068963871563
C	8.07285707276158	-0.72447618718288	-2.08512119316820
H	8.68158464626361	-0.01442653308345	-1.52527992535262
H	8.52694134376410	-1.71290320084316	-1.96742353971654
H	8.11085706163232	-0.46934350328420	-3.14413057217446

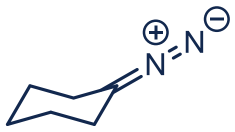

D-1a

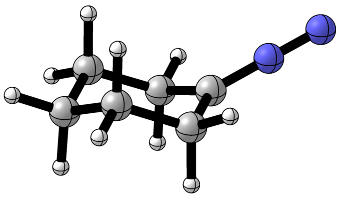

Charge:0, Multiplicity:1

[(CPCM:CH_2Cl_2)B3LYP-D3BJ/def2-TZVP]G_{298K}: -343.93065583

[(CPCM:CH_2Cl_2)B3LYP-D3BJ/def2-TZVP]H_{298K}: -343.89079093

C	-2.84212183638139	-1.43226458363467	-0.02153020504325
C	-2.43354965367637	-0.67898165769274	1.24736593714008
C	-0.97494456748765	-0.20574269013188	1.17322401262346
C	-1.19765329846536	-0.09773283591783	-1.41234051528286
C	-2.65074537741475	-0.57449048419118	-1.27521929518749
H	-0.72566888069158	0.43201353406109	2.02208024439377
H	-3.08542064390062	0.19080094539653	1.37671377611058
H	-2.56427217570210	-1.31584605269901	2.12588480225565
H	-2.23720890585214	-2.34212296449143	-0.11117286685561
H	-3.88407143091821	-1.75218612970975	0.05481878288554
H	-1.09237080292268	0.60951735003890	-2.23578277447159
H	-0.56243378961973	-0.96309384048803	-1.64218850529019
H	-3.31010079895345	0.29760690815870	-1.22049089099672
H	-2.93290592271543	-1.13861037680584	-2.16778575282597
H	-0.31562067817977	-1.08237214683862	1.21977690341795
C	-0.73426634311309	0.52644341260457	-0.12148235736963
N	-0.18318114558881	1.68207261387659	-0.12038206736058
N	0.31170225158315	2.71548899846460	-0.11865922814314

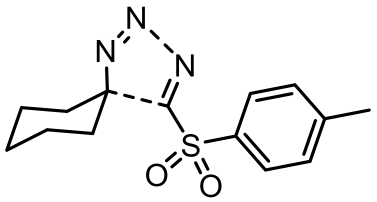

TS1$_{2a}$

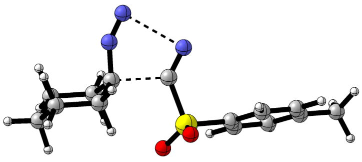

Charge:0, Multiplicity:1

[(CPCM:CH_2Cl_2)B3LYP-D3BJ/def2-TZVP]G_{298K}: -1256.13461416

[(CPCM:CH_2Cl_2)B3LYP-D3BJ/def2-TZVP]H_{298K}: -1256.06914474

C	-2.91313826646482	-1.22104028579840	-0.12569772322664
C	-2.38922719650576	-0.64954879720993	1.19279938996633
C	-0.86769737924728	-0.45319540703731	1.15695930595938
C	-0.99390934871659	-0.15159732564039	-1.38278897480136
C	-2.51356474519092	-0.34853723926541	-1.31707100229473
H	-0.50230768601434	0.01754078905077	2.06984210185040
H	-2.86903430695984	0.31380187399164	1.39348699581058
H	-2.64122104470802	-1.31219153855597	2.02355349859253
H	-2.50894178741551	-2.22923483933600	-0.26702769420048
H	-4.00026695069826	-1.31775171611448	-0.08304409382040
H	-0.71190678825866	0.52904348574273	-2.18688062182783
H	-0.50338778229378	-1.10650016844627	-1.56716197593805
H	-3.00150913369406	0.62847642522572	-1.23824854528888
H	-2.85278716179410	-0.80079343700417	-2.25168478295414
H	-0.37817575418844	-1.42426030701015	1.06942211631777
C	-0.44078680608262	0.35506465625918	-0.05659978243658
N	-0.60890351905245	1.70109925466946	0.09081816685927
N	-0.19301690750099	2.74616757201899	0.11965414910267
C	1.59519595857358	0.59138810195923	-0.14947577691944
N	2.06119379461486	1.68168695734556	-0.19252379337832
S	2.32373150864622	-1.04631475294679	-0.03499807678049
O	2.38286332536896	-1.35038499473110	1.37741610415793
O	1.60411567839643	-1.93898557699985	-0.91411495397437
C	3.95498051219760	-0.81494169905215	-0.65655515211805
C	4.20298097421694	-1.02127042823463	-2.00967375876699
C	4.96382865162698	-0.40714043049984	0.21052017216613
C	5.48792404584672	-0.82567316464051	-2.49279005123542
H	3.40564299627476	-1.33326260796767	-2.66951109387517
C	6.24147112290452	-0.21716912464346	-0.29168391983154
H	4.75090585816525	-0.24546227289855	1.25785940322249
C	6.52413736012914	-0.42411729566332	-1.64536645327353
H	5.69018399431780	-0.98629480776101	-3.54445509658531
H	7.03334572010585	0.09837311614709	0.37621444892257
C	7.91911480592599	-0.24515260084106	-2.17221795100862
H	7.91064074360386	0.03044248746409	-3.22693002508610
H	8.45811719420241	0.51871756613206	-1.61124386344790
H	8.48086031966851	-1.17932046770811	-2.07925468985771

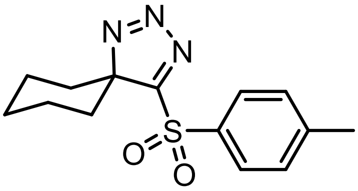

T$_{2a}$

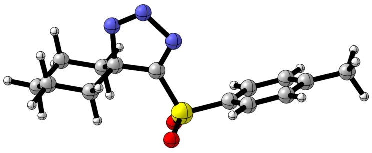

Charge:0, Multiplicity:1

[(CPCM:CH_2Cl_2)B3LYP-D3BJ/def2-TZVP]G_{298K}: -1256.16458841

[(CPCM:CH_2Cl_2)B3LYP-D3BJ/def2-TZVP]H_{298K}: -1256.10073493

C	-3.13792940195934	-1.15447799221409	0.01489083171613
C	-2.57512049666589	-0.46239782179690	1.25572746695301
C	-1.05399752969863	-0.34515249245509	1.19443655788478
C	-1.18814218693098	-0.31524910312971	-1.35604962183494
C	-2.70761984908952	-0.43033058846184	-1.26035694562087
H	-0.66149609737131	0.19597823584499	2.05627355766976
H	-3.01631447829500	0.53265843004636	1.35379890398595
H	-2.84094751384903	-1.01985206944413	2.15658371363606
H	-2.77828420477335	-2.18874530407173	-0.01712618038143
H	-4.22769301244758	-1.19666762198374	0.07179412972817
H	-0.88609370067184	0.24784688358464	-2.24014432232161
H	-0.73937278692701	-1.30618397726818	-1.42838378303505
H	-3.15638952843415	0.56575472620132	-1.28623486100547
H	-3.06716749062251	-0.96581723109268	-2.14190232312964
H	-0.60112682805682	-1.33784073233492	1.19718688233780
C	-0.57945289599751	0.35927422023909	-0.09973687012338
N	-0.97236284808602	1.77366419514781	-0.07446322095099
N	0.00534959644701	2.51982927115311	-0.13973688983456
C	0.90622935992685	0.53215218318438	-0.18152041727386
N	1.26337866825606	1.75159748414466	-0.21257117573639
S	2.11256218803256	-0.78767795036883	-0.19411146377366
O	2.10642093722441	-1.33378440298595	1.14431694383370
O	1.77351593798791	-1.64224374517098	-1.30912505058437
C	3.64614134446679	0.00549438988962	-0.50450007542367
C	4.06740787552421	0.17912853179107	-1.81923645364727
C	4.41125735999728	0.45680499271456	0.56604461450435
C	5.27814989855966	0.80863307946767	-2.05654209253924
H	3.45827619700213	-0.17482966102738	-2.63886353216897
C	5.61914397392748	1.08402506243148	0.30775404339052
H	4.06572191962923	0.31776879027466	1.58055835990572
C	6.07147764870263	1.26817435237283	-1.00158417261735
H	5.61389456821252	0.94763476999074	-3.07651327889221
H	6.22075787215003	1.43906748438734	1.13500396988602
C	7.39559127664820	1.92292063087110	-1.26930898444201
H	7.65176072680600	2.63209849759869	-0.48205085593511
H	8.18847900420043	1.17007804265382	-1.30606551053703
H	7.39215149617508	2.44291243981626	-2.22747989362285

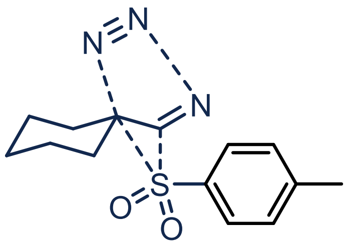

$TS2_{2a}$

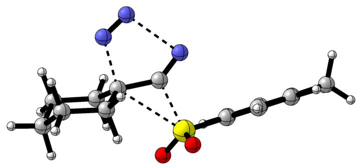

Charge:0, Multiplicity:1

[(CPCM:CH_2Cl_2)B3LYP-D3BJ/def2-TZVP]G_{298K}: -1256.16458841

[(CPCM:CH_2Cl_2)B3LYP-D3BJ/def2-TZVP]H_{298K}: -1256.10073493

C	-2.98923576995570	-1.20972436265661	-0.18546111782336
C	-2.54086690685752	-0.63853883305022	1.15798553953931
C	-1.02592014888502	-0.41425292494784	1.20121803106798
C	-1.04209276914780	-0.06610022286101	-1.32967191101747
C	-2.55450202459065	-0.30571007052646	-1.33676998372332
H	-0.71024704906205	0.08214416090339	2.11958304302056
H	-3.04685086966976	0.31290443748634	1.34163173262619
H	-2.81374095082429	-1.30910740929555	1.97407613359998
H	-2.55764826982626	-2.20688675381955	-0.32118669304507
H	-4.07471715976719	-1.32785251061897	-0.19510549813829
H	-0.73649139142182	0.65926762300831	-2.08569654239197
H	-0.51983884865384	-1.00232256675108	-1.56740479885352
H	-3.06728281167968	0.65716270817805	-1.26240503293803
H	-2.83174996786555	-0.74262067936773	-2.29730558870461
H	-0.52361966476998	-1.38922780302211	1.18754639327730
C	-0.51349402321702	0.34108304074613	0.00932428727846
N	-1.24712461637034	2.09084896440444	0.25245604125257
N	-0.53104699068015	2.94629908488673	0.32692586784104
C	0.91180902693993	0.79829197717750	0.05038498668834
N	1.52142855765903	1.83456989924809	0.14933330103027
S	1.99014250363643	-0.88177568919646	-0.01377168116808
O	2.09538590479767	-1.31664470491132	1.36675655233860
O	1.49913615328941	-1.82304571939038	-1.00690497131095
C	3.55291841146744	-0.25935113018236	-0.54590162087332
C	3.84184176868979	-0.22498678069052	-1.90387188367139
C	4.46142253529082	0.20180806761908	0.40200496324021
C	5.07131096274135	0.27246739820547	-2.31406409052192
H	3.12345084043022	-0.58525338024936	-2.62673844286547
C	5.68262644997170	0.69314173385341	-0.02732259411587
H	4.21557218684901	0.16994715189749	1.45367683587396
C	6.00553070077275	0.74070205847599	-1.38809063097489
H	5.30847870429906	0.29590691404282	-3.37030522638396
H	6.39842069766427	1.04805047212701	0.70410325164258
C	7.32789750339601	1.29600030824835	-1.83438908770373
H	7.38173928759616	2.36819076114059	-1.62810920342454
H	8.15098052682451	0.82296104414439	-1.29492843022535
H	7.48051856092897	1.14710396574396	-2.90265423044221

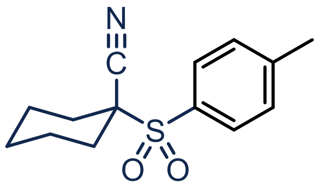

3a

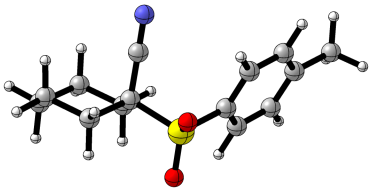

Charge:0, Multiplicity:1

[(CPCM:CH_2Cl_2)B3LYP-D3BJ/def2-TZVP]G_{298K}: -1146.74704393

[(CPCM:CH_2Cl_2)B3LYP-D3BJ/def2-TZVP]H_{298K}: -1146.68578711

C	-2.35131163170710	-0.39066335102471	-0.68974808715280
C	-2.04583395436528	-0.45295857644973	0.80621403940342
C	-0.71059667986532	-1.14736798708914	1.08226749975441
C	0.12608215350577	-0.40053432413202	-1.20500409900711
C	-1.21751070173168	0.28665110796856	-1.45847776469001
H	-0.48246772769035	-1.14794751354715	2.14711104762273
H	-2.02359033238176	0.55920616982151	1.22153550164402
H	-2.83148215786891	-0.99445565581976	1.33642184279109
H	-2.48967530524785	-1.40747177052515	-1.07229199413152
H	-3.28817083644744	0.14462337702992	-0.85749844257108
H	0.92773669566879	0.11747943941342	-1.73027548651600
H	0.09169712849712	-1.42869805585480	-1.56836999689604
H	-1.15013745075893	1.33912213798560	-1.16654413659734
H	-1.41726820472564	0.26938886717740	-2.53146380652639
H	-0.75575442992960	-2.18415479298353	0.74441915793514
C	0.43875969354744	-0.46612209013912	0.30324284919521
C	0.70077870414255	0.86500165716726	0.83582520976571
N	0.89806651441170	1.92148662264152	1.24560111445032
S	1.97036986941270	-1.47814042976423	0.60832581043179
O	2.23176727922823	-1.42655556738810	2.03152573825737
O	1.73554041576307	-2.75995142442151	-0.02239653380651
C	3.26573298601293	-0.64513559776584	-0.24580288499849
C	3.54461843110143	-0.98478260217324	-1.56633761493562
C	3.98098219766813	0.35618295249035	0.40563460399514
C	4.55167925241269	-0.30875194004054	-2.23666746079753
H	2.98538350077585	-1.76897665842062	-2.05617471006933
C	4.98303491955901	1.02122489111054	-0.28187738630699
H	3.75961698864440	0.60464915825664	1.43334304239106
C	5.28391655296800	0.70169452738539	-1.60864589822319
H	4.77337981854657	-0.56869451791226	-3.26417483866307
H	5.54216430809633	1.80179504220902	0.21880000553906
C	6.39123147845617	1.41082298566388	-2.33351078281036
H	6.47506627725004	2.44786944853870	-2.00719348824361
H	7.34949382125091	0.92432809825684	-2.12918258727861
H	6.23402642579997	1.39079537233489	-3.41191346295482

N≡N

N_2

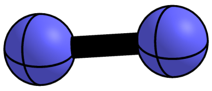

Charge:0, Multiplicity:1
[(CPCM:CH_2Cl_2)B3LYP-D3BJ/def2-TZVP]G_{298K}: -109.53594220
[(CPCM:CH_2Cl_2)B3LYP-D3BJ/def2-TZVP]H_{298K}: -109.51355821

N	-2.94764183641856	3.10607624208493	0.01689753470581
N	-2.68585916358144	4.16048075791507	0.11333046529419

4 Chemical Space Map

All cheminformatics workflows were implemented in Python 3.12.3 utilizing RDKit 2023.09.6.[9] For dimensionality reduction and clustering umap 0.5.6[10] and sklearn 1.4.2[11], respectively, were used. For data plotting seaborn 0.13.2[12] and matplotlib 3.7.5[13] were used.
To select the ketones for the generation of the compound library, the AstraZeneca molecule collection was used as a database, restricted to publicly known molecules only. The following filtering steps were taken:

- Compounds that matched the SMARTS patterns listed in **Table S1** were removed.
- The set of molecules was restricted to compounds that had exactly one keto group (`[#6]-[#6](=O)-[#6]`).
- Duplicates were removed using SMILES strings, both with and without considering stereochemistry (asymmetric atoms).
- Compound availability (amount and location) was considered.

Table S1. Functional groups excluded from chemical space map.

**Name**	**SMARTS**
Aldehyde	`[#6]-[#6h1](=O)`
Michael acceptor	`[#6]=[#6]-[#6](=O)`
Benzylic ketone	`c-[#6](=O)-[#6]`
Phenol	`c-[#8h1]`
Thiol	`[#16h1]`
Nitro	`[#7+](-[#8-])=[#8]`
Primary amine	`[#7h2]`
Primary alcohol	`[#6h2]-[#8h1]`
Secondary alcohol	`[#6h1]-[#8h1]`

dicarbonyl	`[#6](=[#8])-[#6](=[#8])`
Sulfoxide carbonyl	`[#16v4](=[#8])-[#6](=[#8])`
Sulfone carbonyl	`[#16](=[#8])(=[#8])-[#6](=[#8])`
CF_3 carbonyl	`[#6](-[#9])(-[#9])(-[#9])-[#6](=[#8])`

These filtering steps resulted in a set of 876 compounds. The Morgan fingerprint was calculated using RDKit's GetMorganGenerator with `radius=3` and `fpSize=2048` for all compounds. The resulting features were utilized for UMAP dimensionality reduction into 2D, followed by *k*-means clustering (using the 2D UMAP embeddings) to yield `12` clusters. All default settings were applied for both the dimensionality reduction and the clustering algorithm.

The quantitative estimation of drug likeness (QED) of all 876 molecules was calculated with RDKit using the default settings. For each *k*-means cluster, the compounds were ranked by the QED score in ascending order, and the molecule with the highest score was selected for experimental testing. In case the oxadiazoline **1** could not form e.g. due to side-reactions with the hypervalent iodine reagent PIDA, the molecule with the second highest QED score (for the given cluster) was selected. This was repeated until a compound was successfully selected. The final selection of molecules is illustrated in the main article in Figure 3.

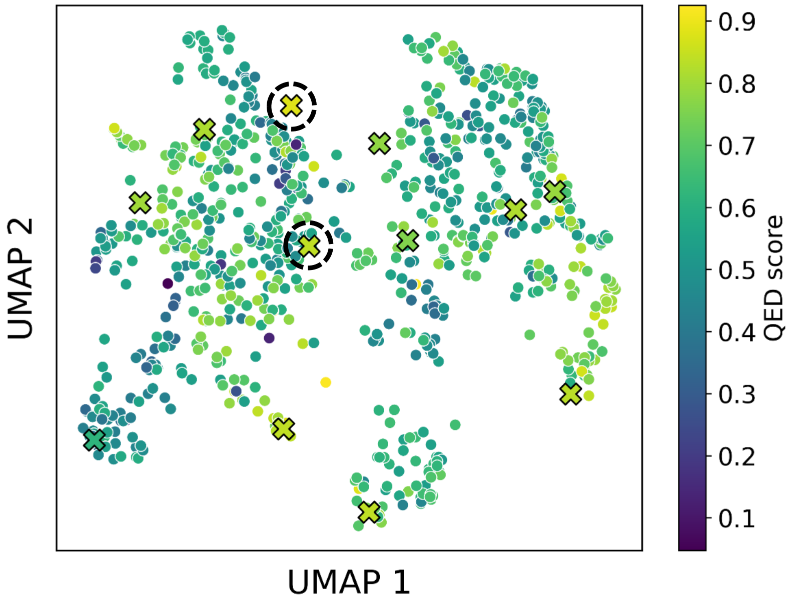

Figure S16. Chemical space of the 876 ketones selected from the AstraZeneca compound collection as obtained with the dimensionality reduction procedure described above. The analogous plot with the cluster labels defining the hue color is given in the main article in Figure 3. The X markers represent the compounds selected for experimental testing. The two circled compounds could not be tested as the corresponding oxadiazolines could not be observed (HPLC-MS) or purified.

5 Synthesis of starting materials

5.1 Ketones

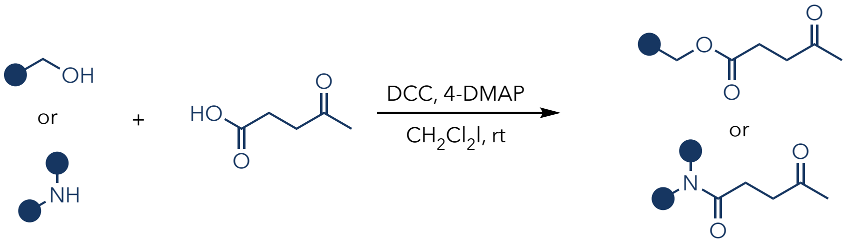

General Procedure A: Synthesis of Ketones

Keton were synthesized according to a modified literature procedure.[14]

To a solution of the corresponding alcohol or amine (1.0 equiv.), levulinic acid (1.2 equiv.) in CH_2Cl_2 (c= 0.2 M for alcohol or amine) was added *N*,*N*'-dicyclohexylcarbodiimide (1.3 equiv.) and 4-dimethylaminopyridine (0.1 equiv.) and the mixture stirred for 1-16 h at ambient temperature. Once full conversion was confirmed by TLC, the precipitate of urea was filtered off and the filtrate concentrated in vacuo. The product purified by silica flash column chromatography (PE/EtOAc) or used directly without further purification.

1-(4-(Furan-2-carbonyl)piperazin-1-yl)pentane-1,4-dione (S1a).

Following general procedure A described above (10 mmol scale), the product was obtained as a white solid (2.71 g, 9.74 mmol, 97%). Purification was achieved by silica column chromatography (EtOAc/MeOH 0 → 20%).

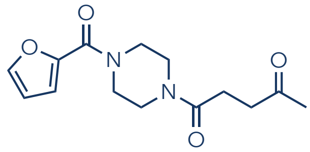

^{1}H NMR (400 MHz, $CDCl_3$) δ = 7.49 (dd, *J*=1.8, 0.9, 1H), 7.05 (dd, *J*=3.5, 0.9, 1H), 6.50 (dd, *J*=3.5, 1.8, 1H), 3.82 (d, *J*=16.0, 4H), 3.68 (dd, *J*=6.6, 3.9, 2H), 3.61 (dd, *J*=6.4, 4.2, 2H), 2.81 (t, *J*=6.3, 2H), 2.62 (dd, *J*=7.6, 5.0, 2H), 2.22 (s, 3H).

^{13}C NMR (101 MHz, $CDCl_3$) δ = 207.8, 170.5, 159.3, 147.8, 144.1, 117.3, 111.6, 45.4, 42.0, 38.1, 30.3, 27.0.

HRMS (ESI): Calcd. for $C_{14}H_{18}N_2O_4$ $[M+H]^+$: 279.1339, Found: 279.1342.

((5*R*,5a*S*,8a*S*,8b*R*)-2,2,7,7-Tetramethyltetrahydro-5*H*-bis([1,3]dioxolo)[4,5-*b*:4',5'-*d*]pyran-5-yl)methyl 4-oxopentanoate (S1b).

Following general procedure A described above (6.15 mmol scale), the product was obtained as a white solid (2.17 g, 6.04 mmol, 98%) and was used without further purification.

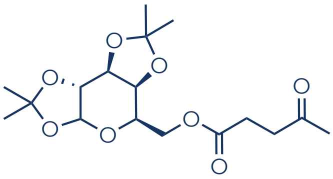

^{1}H NMR (400 MHz, $CDCl_3$) δ = 5.52 (d, *J*=5.0, 1H), 4.60 (dd, *J*=7.9, 2.5, 1H), 4.31 (dd, *J*=5.0, 2.5, 1H), 4.27 (dd, *J*=11.5, 4.8, 1H), 4.22 (dd, *J*=7.4, 1.6, 1H), 4.18 (dd, *J*=11.1, 7.2, 1H), 2.74 (dd, *J*=7.8, 6.3, 2H), 2.61 (dd, *J*=6.8, 5.5, 2H), 2.18 (s, 3H), 1.50 (s, 3H), 1.43 (s, 3H), 1.32 (s, 3H), 1.32 (s, 3H).

^{13}C NMR (101 MHz, $CDCl_3$) δ = 206.6, 172.7, 109.7, 108.9, 96.4, 71.1, 70.8, 70.6, 66.0, 63.7, 38.1, 30.0, 28.1, 26.1, 26.1, 25.1, 24.6.

HRMS (ESI): Calcd. for $C_{17}H_{26}O_8$ $[M+H]^+$: 359.1700 , Found: 359.1713.

1-Tosylpiperidin-4-one (S1c).

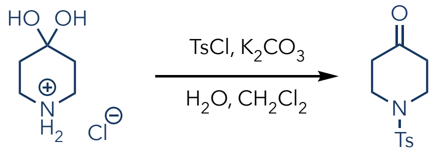

Piperidinone **S1c** was synthesized according to a modified literature procedure.[15]

In a round bottom flask, piperidine-4,4-diol hydrochloride (7.68 g, 50.0 mmol, 1.0 eq.) was mixed with 50 mL water. K_2CO_3 (16.6 g, 120 mmol, 2.4 eq.) was then added portionwise followed by CH_2Cl_2 (50 mL). A solution of *p*-toluenesulfonyl chloride (10.0 g, 52.5 mmol, 1.05 eq.) in CH_2Cl_2 (25 mL) was then added over a period of 30 minutes. The reaction mixture was allowed to stir at room temperature for 16 hours. The organic and aqueous phases were separated and the aqueous phase was then extracted with CH_2Cl_2 two times. The combined organic extracts were washed with saturated aqueous solution of $NaHCO_3$, dried over Na_2SO_4, filtered and concentrated in vacuo to provide 1-tosylpiperidin-4-one as an off-white solid (12.6 g, 49.5 mmol, 99%) that was used without further purification.

^{1}H NMR (400 MHz, $CDCl_3$) δ = 7.71 – 7.63 (m, 2H), 7.37 – 7.30 (m, 2H), 3.37 (t, *J*=6.3, 4H), 2.52 (t, *J*=6.3, 4H), 2.43 (s, 3H).
^{13}C NMR (101 MHz, $CDCl_3$) δ = 205.7, 144.3, 133.5, 130.0, 127.7, 46.0, 40.8, 21.7.
The analytical data are in accordance with those reported in the literature.[15]

5.2 Oxadiazolines

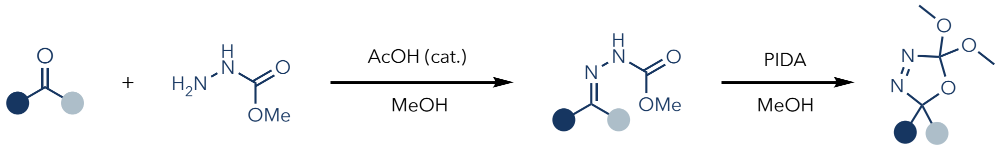

General Procedure B: Synthesis of oxadiazolines (1).
Oxadiazolines were synthesized according to a modified literature procedure.[16]
A solution of the corresponding ketone (1.0 equiv.), methyl hydrazinocarboxylate (1.1 equiv.) and acetic acid (0.03 equiv.) in methanol (c = 1.0 M for ketone) was refluxed for 1 h (or stirred for 16 h at ambient temperature). After cooling the reaction mixture, (diacetoxyiodo)benzene (PIDA, 1.0 equiv.) was added portionwise over 10 minutes at 0 °C. The cooling bath was removed, and the reaction mixture was stirred for 1 h at ambient temperature. After that time, the solvent was removed directly and the mixture purified by silica flash column chromatography (PE/EtOAc).

3,3-Dimethoxy-4-oxa-1,2-diazaspiro[4.5]dec-1-ene (1a).
Following general procedure B described above (100 mmol scale), the product was obtained as a colorless oil (12.5 g, 62.4 mmol, 62%). Purification was achieved by silica column chromatography (PE/EtOAc 0 → 5%).

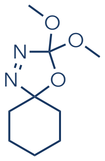

^{1}H NMR (400 MHz, $CDCl_3$) δ = 3.47 (s, 6H), 1.98 (ddd, *J*=13.1, 10.1, 4.7, 2H), 1.93 – 1.81 (m, 2H), 1.74 – 1.60 (m, 3H), 1.59 – 1.45 (m, 3H).
^{13}C NMR (101 MHz, $CDCl_3$) δ = 136.3, 121.2, 52.1, 33.8, 24.9, 22.9.
The analytical data are in accordance with those reported in the literature.[16]

7,7-Dimethoxy-8-oxa-5,6-diazaspiro[3.4]oct-5-ene (1b).
Following general procedure B described above (10 mmol scale), the product was obtained as a yellow oil (521 mg, 3.03 mmol, 30%). Purification was achieved by silica column chromatography (PE/EtOAc 0 → 5%).

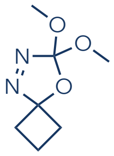

^{1}H NMR (400 MHz, $CDCl_3$) δ = 3.28 (s, 6H), 2.66 – 2.37 (m, 4H), 2.27 – 2.12 (m, 1H), 1.92 (dtt, *J*=11.3, 10.2, 3.2, 1H).
^{13}C NMR (101 MHz, $CDCl_3$) δ = 138.3, 117.9, 51.8, 32.8, 11.7.
HRMS (ESI): Calcd. for $C_7H_{12}N_2O_3$ $[M+H]^+$: 173.0921, Found: 173.0923.

3,3-Dimethoxy-4-oxa-1,2-diazaspiro[4.4]non-1-ene (1c).
Following general procedure B described above (20 mmol scale), the product was obtained as a colorless oil (2.04 g, 11.0 mmol, 55%). Purification was achieved by silica column chromatography (PE/EtOAc 0 → 5%).

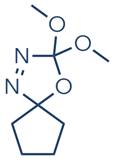

^{1}H NMR (400 MHz, $CDCl_3$) δ = 3.40 (s, 6H), 2.26 – 2.09 (m, 2H), 2.01 – 1.84 (m, 4H), 1.84 – 1.73 (m, 2H).
^{13}C NMR (101 MHz, $CDCl_3$) δ = 137.1, 128.1, 51.9, 35.6, 25.2.
HRMS (ESI): Calcd. for $C_8H_{14}N_2O_3$ $[M+H]^+$: 187.1077, Found: 187.1078.

3,3-Dimethoxy-4-oxa-1,2-diazaspiro[4.6]undec-1-ene (1d).

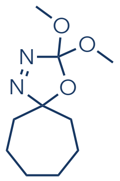

Following general procedure B described above (20 mmol scale), the product was obtained as a colorless oil (1.06 g, 4.90 mmol, 25%). Purification was achieved by silica column chromatography (PE/EtOAc 0 → 5%).

^{1}H NMR (400 MHz, $CDCl_3$) δ = 3.62 – 3.23 (m, 6H), 2.13 – 1.95 (m, 2H), 1.82 – 1.58 (m, 10H).
^{13}C NMR (101 MHz, $CDCl_3$) δ = 136.8, 124.8, 52.0, 36.4, 29.5, 23.1.
HRMS (ESI): Calcd. for $C_{10}H_{18}N_2O_3$ $[M+H]^+$: 215.1390, Found: 215.1392.

3,3-Dimethoxy-4,8-dioxa-1,2-diazaspiro[4.5]dec-1-ene (1e).

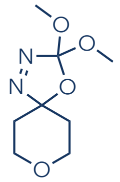

Following general procedure B described above (10 mmol scale), the product was obtained as a colorless oil (1.04 g, 5.16 mmol, 52%). Purification was achieved by silica column chromatography (PE/EtOAc 0 → 25%).

^{1}H NMR (400 MHz, $CDCl_3$) δ = 4.06 (ddd, J=11.6, 5.8, 4.4, 2H), 3.87 – 3.78 (m, 2H), 3.49 (s, 6H), 2.25 – 2.13 (m, 2H), 1.65 (dddd, J=13.6, 5.0, 3.2, 1.5, 2H).
^{13}C NMR (101 MHz, $CDCl_3$) δ = 136.9, 117.9, 64.9, 52.2, 34.0.
The analytical data are in accordance with those reported in the literature.[16]

***tert*-Butyl 3,3-dimethoxy-4-oxa-1,2,8-triazaspiro[4.5]dec-1-ene-8-carboxylate (1f).**

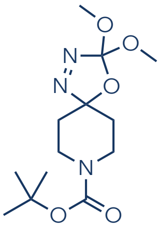

Following general procedure B described above (10 mmol scale), the product was obtained as a white solid (1.68 g, 5.58 mmol, 56%). Purification was achieved by silica column chromatography (PE/EtOAc 0 → 20%).

^{1}H NMR (400 MHz, $CDCl_3$) δ = 4.07 – 3.85 (m, 2H), 3.49 (s, 6H), 3.40 (ddd, J=13.4, 9.6, 3.7, 2H), 2.09 (ddd, J=13.7, 9.4, 4.5, 2H), 1.63 – 1.54 (m, 2H), 1.48 (s, 9H).
^{13}C NMR (101 MHz, $CDCl_3$) δ = 154.7, 137.0, 118.8, 80.3, 52.3, 41.2, 33.3, 28.5.
The analytical data are in accordance with those reported in the literature.[16]

3,3-Dimethoxy-8-tosyl-4-oxa-1,2,8-triazaspiro[4.5]dec-1-ene (1g).

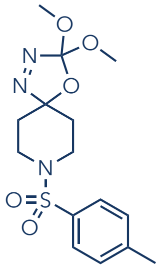

Following general procedure B described above (50 mmol scale), the product was obtained as a white solid (4.10 g, 11.5 mmol, 23%). Purification was achieved by flash column chromatography (CH_2Cl_2/MeOH 0% → 10%).
Structure was confirmed by single-crystal X-ray diffraction.

^{1}H NMR (300 MHz, $CDCl_3$) δ = 7.70 – 7.63 (m, 2H), 7.38 – 7.30 (m, 2H), 3.62 (dt, J=11.4, 5.3, 2H), 3.44 (s, 6H), 3.06 – 2.89 (m, 2H), 2.45 (s, 3H), 2.26 (ddd, J=14.3, 9.7, 4.4, 2H), 1.77 – 1.64 (m, 2H).
^{13}C NMR (101 MHz, $CDCl_3$) δ = 144.1, 137.2, 133.2, 130.0, 127.8, 117.4, 52.4, 43.5, 33.0, 21.7.
The analytical data are in accordance with those reported in the literature.[16]

8-(*tert*-Butyl)-3,3-dimethoxy-4-oxa-1,2-diazaspiro[4.5]dec-1-ene (1h).

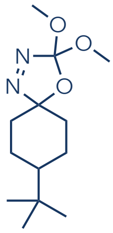

Following general procedure B described above (10 mmol scale), the product was obtained as a colorless oil (937 mg, 3.66 mmol, 37%, d.r. = 1.3-1.5). Purification was achieved by silica column chromatography (PE/EtOAc 0 → 5%).

^{1}H NMR (400 MHz, $CDCl_3$) δ = 3.52 – 3.42 (m, 6H), 2.16 (td, *J*=13.5, 4.7, 1H), 1.94 – 1.43 (m, 6H), 1.41 – 1.12 (m, 2H), 0.95 – 0.87 (m, 9H).
^{13}C NMR (101 MHz, $CDCl_3$) δ = 136.6, 121.2, 121.1, 52.1, 52.1, 47.3, 46.8, 35.7, 33.4, 32.6, 27.8, 27.7, 25.0, 23.5.
HRMS (ESI): Calcd. for $C_{13}H_{24}N_2O_3$ $[M+H]^+$: 279.1679, Found: 279.1682.

***tert*-Butyl 5',5'-dimethoxy-5'*H*-3-azaspiro[bicyclo[3.2.1]octane-8,2'-[1,3,4]oxadiazole]-3-carboxylate (1i).**

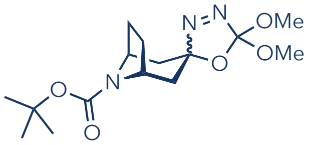

Following general procedure B described above (10 mmol scale), the product was obtained as a white solid (1.38 mg, 4.22 mmol, 42%). Purification was achieved by silica column chromatography (PE/EtOAc 0 → 25%).

^{1}H NMR (400 MHz, $CDCl_3$) δ = 4.42 – 4.19 (m, 2H), 3.44 (s, 6H), 2.58 (d, *J*=33.4, 1H), 2.42 – 1.72 (m, 5H), 1.45 (d, *J*=4.8, 9H), 1.39 (dt, *J*=13.7, 1.8, 2H).
^{13}C NMR (101 MHz, $CDCl_3$) δ = 153.3, 138.7, 135.3, 118.8, 118.8, 79.9, 79.9, 52.3, 52.2, 51.7, 40.2, 39.3, 37.7, 37.0, 28.9, 28.5, 28.5, 28.1, 27.8, 27.1.
The analytical data are in accordance with those reported in the literature.[16]

3,3-Dimethoxy-8,8-dimethyl-4,7,9-trioxa-1,2-diazaspiro[4.5]dec-1-ene (1j).

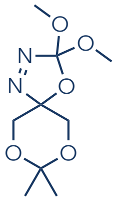

Following general procedure B described above (5 mmol scale), the product was obtained as a colorless oil (370 mg, 1.59 mmol, 32%). Purification was achieved by silica column chromatography (PE/EtOAc 0 → 25%).

^{1}H NMR (400 MHz, $CDCl_3$) δ = 4.14 (d, *J*=12.6, 2H), 3.73 (d, *J*=12.6, 2H), 3.49 (s, 6H), 1.57 – 1.54 (m, 3H), 1.52 – 1.48 (m, 3H).
^{13}C NMR (101 MHz, $CDCl_3$) δ = 137.7, 115.9, 99.7, 62.9, 52.4, 24.0, 23.0.
The analytical data are in accordance with those reported in the literature.[16]

7,7-Dimethoxy-2,8-dioxa-5,6-diazaspiro[3.4]oct-5-ene (1k).

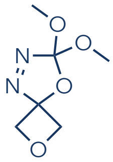

Following general procedure B described above (10 mmol scale), the product was obtained as a colorless oil (421 mg, 2.42 mmol, 24%). Purification was achieved by silica column chromatography (PE/EtOAc 0 → 25%).

^{1}H NMR (400 MHz, $CDCl_3$) δ = 5.12 – 5.03 (m, 2H), 5.01 – 4.88 (m, 2H), 3.39 (s, 6H).
^{13}C NMR (101 MHz, $CDCl_3$) δ = 139.1, 114.6, 78.1, 52.3.
The analytical data are in accordance with those reported in the literature.[16]

7,7-Dimethoxy-8-oxa-2-thia-5,6-diazaspiro[3.4]oct-5-ene (1l).
Following general procedure B described above (10 mmol scale), the product was obtained as a colorless oil (320 mg, 1.68 mmol, 17%). Purification was achieved by silica column chromatography (PE/EtOAc 0 → 25%).

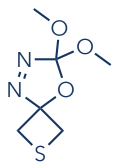

^{1}H NMR (400 MHz, $CDCl_3$) δ = 3.87 – 3.82 (m, 2H), 3.44 – 3.39 (m, 2H), 3.38 (s, 6H).
^{13}C NMR (101 MHz, $CDCl_3$) δ = 139.7, 116.5, 52.3, 36.4.
HRMS (ESI): Calcd. for $C_6H_{10}N_2O_3S$ $[M+H]^+$: 191.0485, Found: 191.0486.

2,2-dimethoxy-5,5-dimethyl-2,5-dihydro-1,3,4-oxadiazole (1m).
Following general procedure B described above (20 mmol scale), the product was obtained as a colorless oil (938 mg, 5.86 mmol, 29%). Purification was achieved by silica column chromatography (PE/Et_2O 0 → 10%).

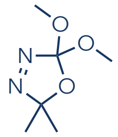

^{1}H NMR (400 MHz, $CDCl_3$) δ = 3.39 (s, 6H), 1.47 (s, 6H).
^{13}C NMR (101 MHz, $CDCl_3$) δ = 137.2, 119.1, 51.8, 24.0.
HRMS (ESI): Calcd. for $C_6H_{12}N_2O_3$ $[M+H]^+$: 161.0921, Found: 161.0922.

3-(5,5-Dimethoxy-2-methyl-2,5-dihydro-1,3,4-oxadiazol-2-yl)-1-(4-(furan-2-carbonyl)piperazin-1-yl)propan-1-one (1n).
Following general procedure B described above (10 mmol scale), the product was obtained as a yellow oil (241 mg, 637 μmol, 6%). Purification was achieved by silica column chromatography (PE/EtOAc 40 → 100%).

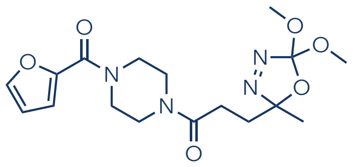

^{1}H NMR (400 MHz, $CDCl_3$) δ = 7.48 (dd, *J*=1.8, 0.8, 1H), 7.04 (dd, *J*=3.5, 0.9, 1H), 6.49 (dd, *J*=3.5, 1.7, 1H), 3.88 – 3.41 (m, 14H), 2.43 – 2.12 (m, 4H), 1.53 (s, 3H).
^{13}C NMR (101 MHz, $CDCl_3$) δ = 170.3, 159.3, 147.7, 144.1, 137.4, 121.3, 117.3, 111.6, 52.4, 51.9, 45.3, 41.8, 32.5, 27.0, 22.9.
HRMS (ESI): Calcd. for $C_{17}H_{24}N_4O_6$ $[M+Na]^+$: 403.1588, Found: 403.1592.

2,2-Dimethoxy-5-(4-methoxyphenethyl)-5-methyl-2,5-dihydro-1,3,4-oxadiazole (1o).
Following general procedure B described above (10 mmol scale), the product was obtained as a colorless oil (777 mg, 2.77 mmol, 28%). Purification was achieved by silica column chromatography (PE/EtOAc 0 → 10%).

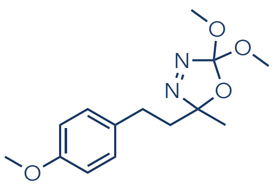

^{1}H NMR (400 MHz, $CDCl_3$) δ = 7.12 – 7.05 (m, 2H), 6.87 – 6.78 (m, 2H), 3.78 (s, 3H), 3.56 (s, 3H), 3.50 (s, 3H), 2.69 (ddd, *J*=13.9, 12.0, 5.4, 1H), 2.51 (ddd, *J*=13.8, 11.9, 5.3, 1H), 2.23 – 2.02 (m, 2H), 1.56 (s, 3H).
^{13}C NMR (101 MHz, $CDCl_3$) δ = 158.2, 137.3, 133.1, 129.3, 121.5, 114.1, 55.4, 52.2, 52.0, 39.7, 28.9, 22.6.
HRMS (ESI): Calcd. for $C_{14}H_{20}N_2O_4$ $[M+H]^+$: 281.1496, Found: 281.1492.

((5*R*,5a*S*,8a*S*,8b*R*)-2,2,7,7-Tetramethyltetrahydro-5*H*-bis([1,3]dioxolo)[4,5-*b*:4',5'-*d*]pyran-5-yl)methyl 3-(5,5-dimethoxy-2-methyl-2,5-dihydro-1,3,4-oxadiazol-2-yl)propanoate (1p).

Following general procedure B described above (6.15 mmol scale), the product was obtained as a colorless sticky oil (863 mg, 1.87 mmol, 30%). Purification was achieved by silica column chromatography (PE/EtOAc 20 → 25%).

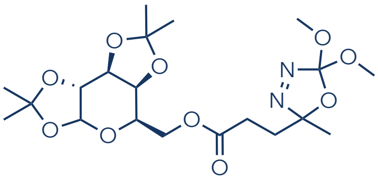

^{1}H NMR (400 MHz, $CDCl_3$) δ = 5.52 (d, *J*=5.0, 1H), 4.61 (dd, *J*=7.9, 2.5, 1H), 4.32 (dd, *J*=5.0, 2.5, 1H), 4.30 – 4.16 (m, 3H), 4.01 (ddd, *J*=7.6, 5.1, 2.5, 1H), 3.54 (s, 3H), 3.47 (s, 3H), 2.50 – 2.39 (m, 1H), 2.36 – 2.15 (m, 3H), 1.51 (s, 3H), 1.50 (s, 3H), 1.44 (s, 3H), 1.33 (s, 3H), 1.32 (s, 3H).

^{13}C NMR (101 MHz, $CDCl_3$) δ = 172.5, 137.5, 120.8, 109.8, 108.9, 96.4, 71.2, 70.8, 70.6, 66.0, 66.0, 63.8, 52.3, 52.0, 32.5, 32.4, 28.5, 28.4, 26.2, 26.1, 25.1, 24.6, 22.6, 22.6.

HRMS (ESI): Calcd. for $C_{20}H_{32}N_2O_{10}$ $[M+Na]^+$: 483.1949, Found: 483.1955.

2,2-Dimethoxy-5-methyl-5-(2-(2,6,6-trimethylcyclohex-1-en-1-yl)ethyl)-2,5-dihydro-1,3,4-oxadiazole (1q).

Following general procedure B described above (20 mmol scale), the product was obtained as a colorless oil (2.69 g, 9.08 mmol, 45%). Purification was achieved by silica column chromatography (PE/EtOAc 0 → 5%).

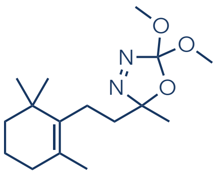

^{1}H NMR (400 MHz, $CDCl_3$) δ = 3.53 (s, 3H), 3.48 (s, 3H), 2.16 – 2.09 (m, 1H), 1.98 – 1.81 (m, 5H), 1.63 – 1.49 (m, 8H), 1.44 – 1.36 (m, 2H), 0.96 (s, 6H).

^{13}C NMR (101 MHz, $CDCl_3$) δ = 137.0, 135.8, 128.2, 122.0, 52.1, 52.0, 39.9, 37.7, 35.2, 32.9, 28.6, 28.6, 22.2, 22.2, 19.8, 19.6.

HRMS (ESI): Calcd. for $C_{16}H_{28}N_2O_3$ $[M+H]^+$: 297.2173, Found: 297.2177.

1-(4-(5,5-Dimethoxy-2-methyl-2,5-dihydro-1,3,4-oxadiazol-2-yl)butyl)-3,7-dimethyl-3,7-dihydro-1*H*-purine-2,6-dione (1r).

Following general procedure B described above (20 mmol scale), the product was obtained as a yellow sticky oil (2.34 g, 6.15 mmol, 31%). Purification was achieved by silica column chromatography (DCM/MeOH 0 → 20%).

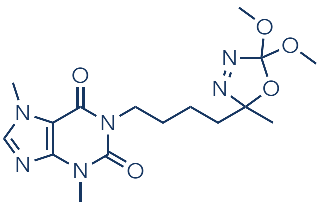

^{1}H NMR (400 MHz, $CDCl_3$) δ = 7.50 (d, *J*=0.6, 1H), 4.02 – 3.97 (m, 5H), 3.56 (s, 3H), 3.50 (s, 3H), 3.46 (s, 3H), 1.95 (ddd, *J*=13.9, 11.9, 4.9, 1H), 1.82 (ddd, *J*=13.9, 11.6, 5.0, 1H), 1.72 – 1.63 (m, 2H), 1.49 (s, 4H), 1.44 – 1.33 (m, 1H).

^{13}C NMR (101 MHz, $CDCl_3$) δ = 155.3, 151.5, 148.9, 141.5, 137.1, 121.7, 107.8, 52.2, 51.9, 41.1, 37.2, 33.7, 29.8, 28.0, 22.3, 21.0.

HRMS (ESI): Calcd. for $C_{16}H_{24}N_6O_5$ $[M+H]^+$: 381.1881, Found: 381.1882.

4-(5,5-Dimethoxy-2-methyl-2,5-dihydro-1,3,4-oxadiazol-2-yl)-*N*-phenylbutanamide (L1).
Following general procedure B described above (5 mmol scale), the product was obtained as a yellow oil (709 mg, 2.31 mmol, 46%). Purification was achieved by silica column chromatography (Hept/EtOAc 0 → 15%).

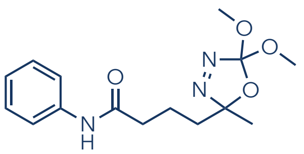

^{1}H NMR (500 MHz, $CDCl_3$) δ = 7.54 – 7.45 (m, 2H), 7.36 – 7.27 (m, 3H), 7.09 (t, *J*=7.4, 1H), 3.55 (s, 3H), 3.46 (s, 3H), 2.38 (t, *J*=7.1, 2H), 2.03 – 1.96 (m, 1H), 1.94 – 1.84 (m, 2H), 1.81 – 1.73 (m, 1H), 1.52 (s, 3H).
^{13}C NMR (126 MHz, $CDCl_3$) δ = 170.6, 137.9, 137.3, 129.1, 124.4, 121.7, 119.9, 52.4, 51.9, 37.1, 36.5, 22.3, 19.7.
MS (ESI): Calcd. for $C_{15}H_{21}N_3O_4$ $[M-H]^-$: 306.2, Found: 306.3.

3,3-Dimethoxy-8-(6-methoxypyridin-3-yl)-4-oxa-1,2-diazaspiro[4.5]dec-1-en-8-ol (L2).
Following general procedure B described above (5 mmol scale), the product was obtained as a white solid (931 mg, 2.31 mmol, 46%, mixture of diastereomers). Purification was achieved by silica column chromatography (Hept/EtOAc 20 → 30%).

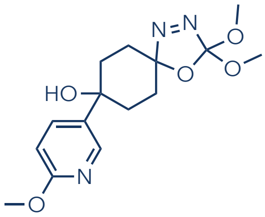

^{1}H NMR (500 MHz, $CDCl_3$) δ = 8.42 (dd, *J*=2.6, 0.8, 0.3H), 8.36 (dd, *J*=2.7, 0.7, 0.7H), 7.84 (dd, *J*=8.8, 2.6, 0.3H), 7.78 (dd, *J*=8.7, 2.7, 0.7H), 6.80 (dd, *J*=8.8, 0.8, 0.3H), 6.78 (dd, *J*=8.7, 0.7, 0.7H), 3.99 (s, 0.8H), 3.98 (s, 2.2H), 3.53 (s, 4.4H), 3.52 (s, 1.6H), 2.86 – 2.75 (m, 1.5H), 2.58 (td, *J*=13.9, 4.3, 0.5H), 2.41 (td, *J*=13.3, 4.3, 0.5H), 2.15 (td, *J*=13.8, 4.3, 1.5H), 2.05 – 1.95 (m, 2H), 1.75 – 1.63 (m, 0.5H), 1.53 – 1.40 (m, 1.5H).
^{13}C NMR (126 MHz, $CDCl_3$) δ = 171.3, 163.4, 163.4, 143.1, 142.9, 136.8, 136.6, 136.6, 136.4, 136.2, 136.0, 120.2, 119.9, 110.8, 110.7, 71.0, 70.6, 60.5, 54.0, 54.0, 52.2, 52.2, 36.2, 34.9, 30.8, 28.8, 21.2, 14.3.
HRMS (ESI): Calcd. for $C_{15}H_{21}N_3O_5$ $[M+H]^+$: 324.1559, Found: 324.1552.

4,5',5'-Trimethoxy-1-phenyl-5'*H*-spiro[bicyclo[2.2.2]octane-2,2'-[1,3,4]oxadiazole] (L4).
Following general procedure B described above (3 mmol scale), the product was obtained as a white solid (845 mg, 2.54 mmol, 85%). Purification was achieved by silica column chromatography (Hept/EtOAc 0 → 15%).

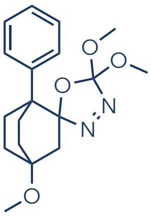

^{1}H NMR (500 MHz, $CDCl_3$) δ = 7.28 – 7.22 (m, 2H), 7.23 – 7.17 (m, 2H), 7.17 – 7.12 (m, 1H), 3.43 (s, 3H), 3.25 (s, 3H), 3.17 – 3.10 (m, 1H), 2.77 – 2.67 (m, 1H), 2.61 (s, 3H), 2.17 – 2.06 (m, 2H), 1.98 – 1.87 (m, 5H), 1.83 – 1.75 (m, 1H).
^{13}C NMR (126 MHz, $CDCl_3$) δ = 140.3, 136.3, 128.1, 127.7, 127.1, 122.9, 73.3, 52.0, 51.3, 49.7, 41.6, 40.6, 29.7, 29.1, 28.0.
HRMS (ESI): Calcd. for $C_{18}H_{24}N_2O_4$ $[M+H]^+$: 333.1814, Found: 333.1758.

Benzyl 6-ethyl-3,3-dimethoxy-4-oxa-1,2,8-triazaspiro[4.5]dec-1-ene-8-carboxylate (L5).
Following general procedure B described above (5 mmol scale), the product was obtained as a colorless oil (621 mg, 1.71 mmol, 34%, mixture of diasteromers). Purification was achieved by silica column chromatography (Hept/EtOAc 0 → 10%).

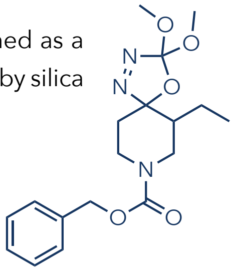

^{1}H NMR (500 MHz, $CDCl_3$) δ = 7.50 – 7.27 (m, 5H), 6.05 – 4.70 (m, 2H), 4.47 – 2.92 (m, 9H), 2.72 – 0.40 (m, 9H).

^{13}**C NMR** (126 MHz, $CDCl_3$) δ = 155.44, 155.33, 136.94, 136.73, 136.27, 128.67, 128.24, 128.06, 121.64, 121.52, 67.50, 65.49, 52.40, 52.36, 52.08, 52.01, 45.46, 44.83, 44.65, 41.62, 41.08, 33.59, 32.65, 18.91, 18.85, 11.96, 11.69.
HRMS (ESI): Calcd. for $C_{18}H_{25}N_3O_5$ $[M+H]^+$: 364.1872, Found: 364.1902.
LC-MS (Peak 5):

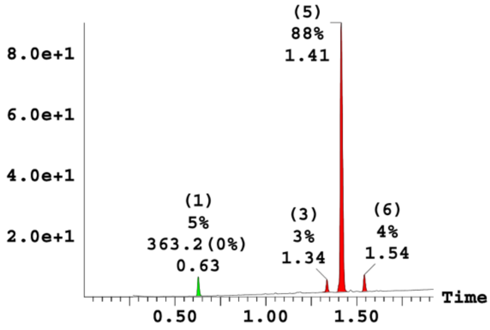

2-(((4-((5-Chloropyridin-2-yl)oxy)piperidin-1-yl)sulfonyl)methyl)-5,5-dimethoxy-2-methyl-2,5-dihydro-1,3,4-oxadiazole (L6).

Following general procedure B described above (3 mmol scale), the product was obtained as a white solid (667 mg, 1.53 mmol, 51%). Purification was achieved by silica column chromatography (Hept/EtOAc 0 → 25%).

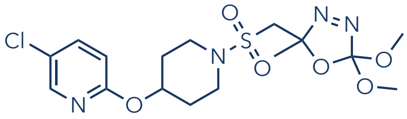

^{1}**H NMR** (500 MHz, $CDCl_3$) δ = 8.05 (dd, *J*=2.7, 0.7, 1H), 7.53 (dd, *J*=8.8, 2.7, 1H), 6.67 (dd, *J*=8.8, 0.7, 1H), 5.19 (tt, *J*=6.9, 3.4, 1H), 3.62 (s, 3H), 3.54 – 3.47 (m, 3H), 3.43 (s, 3H), 3.33 (tdd, *J*=9.9, 3.4, 1.6, 2H), 2.97 (d, *J*=14.5, 1H), 2.09 – 2.00 (m, 2H), 1.91 (dtd, *J*=13.6, 7.3, 3.6, 2H), 1.80 (s, 3H).
^{13}**C NMR** (126 MHz, $CDCl_3$) δ = 161.2, 145.1, 139.4, 139.0, 124.3, 116.6, 112.7, 69.3, 54.7, 52.9, 52.3, 42.7, 42.7, 30.5, 21.7.
HRMS (ESI): Calcd. for $C_{16}H_{23}ClN_4O_6S$ $[M+H]^+$: 435.1105, Found: 435.1081.

tert-Butyl 7-benzyl-3,3-dimethoxy-4-oxa-1,2,8-triazaspiro[4.5]dec-1-ene-8-carboxylate (L7).

Following general procedure B described above (5 mmol scale), the product was obtained as a colorless oil (1.33 g, 3.40 mmol, 68%, mixture of diasteromers). Purification was achieved by silica column chromatography (Hept/EtOAc 0 → 15%).

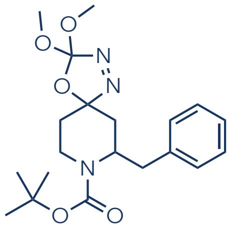

^{1}H NMR (500 MHz, $CDCl_3$) δ = 7.30 – 7.25 (m, 2H), 7.19 (dtd, *J*=8.5, 6.6, 4.3, 3H), 4.82 – 4.52 (m, 1H), 4.27 (d, *J*=14.2, 1H), 3.57 – 3.39 (m, 6H), 3.28 – 3.02 (m, 2H), 2.96 – 2.79 (m, 0.5H), 2.49 (dd, *J*=14.2, 6.8, 0.5H), 2.37 – 2.24 (m, 0.5H), 2.02 – 1.93 (m, 1H), 1.72 (dq, *J*=13.4, 2.5, 0.5H), 1.68 – 1.27 (m, 11H).

^{13}C NMR (126 MHz, $CDCl_3$) δ = 171.3, 154.6, 154.4, 138.8, 138.4, 135.8, 129.6, 129.5, 128.6, 128.5, 126.5, 126.5, 118.9, 118.8, 80.2, 80.1, 60.5, 52.5, 52.4, 52.2, 52.1, 38.7, 34.6, 32.4, 28.4, 28.3, 21.2, 14.3.

HRMS (ESI): Calcd. for $C_{20}H_{29}N_3O_5$ $[M+H]^+$: 392.2185, Found: 392.2206.

Ethyl 2-(2-(4-bromobenzyl)-5,5-dimethoxy-2,5-dihydro-1,3,4-oxadiazol-2-yl)acetate (L8).

Following general procedure B described above (5 mmol scale), the product was obtained as a colorless oil (896 mg, 2.31 mmol, 46%). Purification was achieved by silica column chromatography (Hept/EtOAc 0 → 10%).

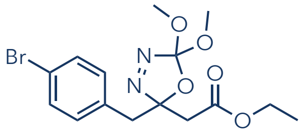

^{1}H NMR (500 MHz, $CDCl_3$) δ = 7.45 – 7.38 (m, 2H), 7.21 – 7.12 (m, 2H), 4.20 – 4.13 (m, 2H), 3.49 (s, 3H), 3.37 (s, 3H), 3.34 (d, *J*=14.2, 1H), 3.28 (d, *J*=14.2, 1H), 2.75 (d, *J*=15.0, 1H), 2.64 (d, *J*=15.1, 1H), 1.27 (t, *J*=7.1, 3H).

^{13}C NMR (126 MHz, $CDCl_3$) δ = 168.0, 138.1, 133.0, 132.7, 131.6, 121.6, 119.3, 61.3, 52.4, 52.3, 41.2, 40.0, 14.2.

HRMS (ESI): Calcd. for $C_{15}H_{19}BrN_2O_5$ $[M+H]^+$: 387.0555, Found: 387.0571.

tert-Butyl 3-benzyl-5',5'-dimethoxy-5'*H*-3,7-diazaspiro[bicyclo[3.3.1]nonane-9,2'-[1,3,4]oxadiazole]-7-carboxylate (L9).

Following general procedure B described above (5 mmol scale), the product was obtained as a colorless oil (320 mg, 740 μmol, 15%, mixture of diasteromers). Purification was achieved by silica column chromatography (Hept/EtOAc 0 → 15%).

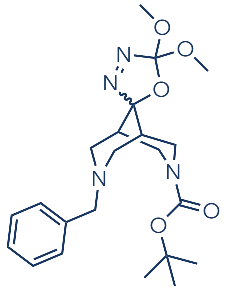

^{1}H NMR (500 MHz, $CDCl_3$) δ = 7.44 – 7.29 (m, 4H), 7.30 – 7.20 (m, 1H), 4.37 (dd, *J*=55.9, 13.2, 1H), 4.27 – 4.06 (m, 1H), 3.96 – 3.76 (m, 1H), 3.70 – 3.30 (m, 9H), 3.26 – 2.92 (m, 3H), 2.62 (s, 1H), 1.57 (d, *J*=8.0, 11H).

^{13}C NMR (126 MHz, $CDCl_3$) δ = 154.9, 138.6, 138.3, 136.1, 128.7, 128.4, 127.1, 119.9, 79.6, 62.8, 62.5, 56.2, 56.0, 55.6, 52.3, 47.4, 46.7, 45.8, 38.4, 32.0, 29.2, 28.8, 28.8, 22.8, 14.3.

HRMS (ESI): Calcd. for $C_{22}H_{32}N_4O_5$ $[M+H]^+$: 433.2451, Found: 433.2489.

3,3-Dimethoxy-8-(3-(trifluoromethyl)-[1,2,4]triazolo[4,3-*b*]pyridazin-6-yl)-4-oxa-1,2,8-triazaspiro[4.5]dec-1-ene (L11).

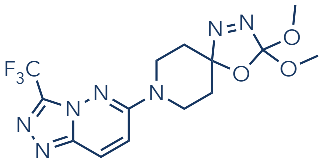

Following general procedure B described above (5 mmol scale), the product was obtained as a faint yellow solid (480 mg, 1.24 mmol, 25%). The product crystalized from the reaction medium and was filtered off, washed with cold methanol and used directly without further purification.

^{1}H NMR (500 MHz, $CDCl_3$) δ = 7.98 (d, *J*=10.2, 1H), 7.19 (d, *J*=10.2, 1H), 4.19 – 4.10 (m, 2H), 3.67 (ddd, *J*=13.4, 9.6, 3.5, 2H), 3.50 (s, 6H), 2.26 (ddd, *J*=13.9, 9.7, 4.3, 2H), 1.76 (dddd, *J*=13.7, 5.1, 3.4, 1.3, 2H).
^{13}C NMR (126 MHz, $CDCl_3$) δ = 155.14, 143.95, 139.12 (q, *J*=40.9), 137.30, 124.84, 118.51 (q, *J*=270.1), 117.91, 115.45, 52.31, 43.42, 32.56.
^{19}F NMR (471 MHz, $CDCl_3$) δ = –64.47.
HRMS (ESI): Calcd. for $C_{14}H_{16}F_3N_7O_3$ $[M+H]^+$: 388.1345, Found: 388.1379.

Methyl 8-(4-chlorophenyl)-3,3-dimethoxy-4-oxa-1,2-diazaspiro[4.4]non-1-ene-7-carboxylate (L12).

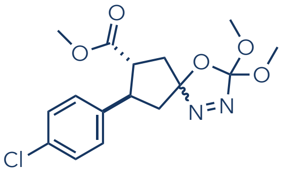

Following general procedure B described above (5 mmol scale), the product was obtained as a colorless oil (1.40 g, 3.94 mmol, 79%, mixture of diasteromers). Purification was achieved by silica column chromatography (Hept/EtOAc 0 → 10%).

^{1}H NMR (500 MHz, $CDCl_3$) δ = 7.35 – 7.22 (m, 4H), 3.83 – 3.67 (m, 1H), 3.64 – 3.60 (m, 3H), 3.51 – 3.47 (m, 3H), 3.49 – 3.41 (m, 3H), 3.30 (td, *J*=10.6, 8.5, 0.5H), 3.21 (td, *J*=11.1, 7.9, 0.5H), 2.77 – 2.61 (m, 1.5H), 2.45 (ddd, *J*=14.2, 11.5, 0.8, 0.5H), 2.35 (ddd, *J*=14.2, 10.4, 1.1, 0.5H), 2.24 (dddd, *J*=13.9, 12.2, 8.0, 1.1, 0.5H), 2.14 – 2.02 (m, 0.5H).
^{13}C NMR (126 MHz, $CDCl_3$) δ 173.4, 173.3, 139.7, 139.5, 138.0, 137.9, 133.1, 133.1, 129.0, 128.8, 128.8, 124.7, 124.6, 52.3, 52.2, 52.2, 52.2, 51.1, 50.7, 47.6, 47.3, 43.2, 42.8, 39.3, 39.3.
HRMS (ESI): Calcd. for $C_{16}H_{19}ClN_2O_5$ $[M+H]^+$: 355.1031, Found: 355.1031.

5.3 Aryl sulfonyl and sulfinyl cyanides

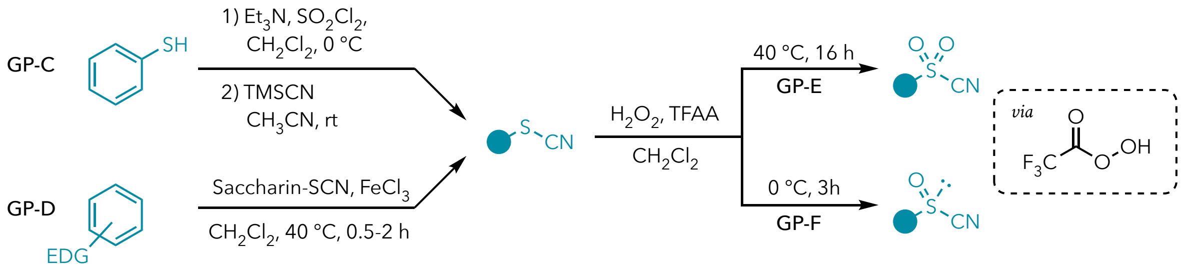

Aryl sulfonyl cyanides were synthesized according to modified literature procedures.

General Procedure C: Synthesis of aryl thiocyanates (S2).[17,18]
To a solution of thiophenol (10 mmol, 1.0 equiv.) in dichloromethane (16 mL) was added trimethylamine (0.1 mL) at 0 °C under N_2 atmosphere. The mixture was stirred at 0 °C for 5 min, then SO_2Cl_2 (11 mmol, 1.1 equiv.) was added dropwise. The reaction mixture was stirred at 0 °C for 30 min until all the HCl gas had evolved. Then dichloromethane was removed by evaporation and replaced with acetonitrile (16 mL) followed by the slow addition of TMSCN (11 mmol, 1.1 equiv.). The resulting brown solution was stirred at 25 °C for 5 h and concentrated. The residue was purified by column chromatography on silica gel to afford aryl thiocyanate **S2**.

General Procedure D: Synthesis of aryl thiocyanates (S2).[19]
To a solution of *N*-thiocyanatosaccharin (481 mg, 2.00 mmol, 1.0 eq.) and $FeCl_3$ (8.1 mg, 0.05 mmol, 2.5 mol%) in dry dichloromethane (10 mL) under nitrogen atmosphere was added the arene (2.00 mmol, 1.0 eq.). The reaction mixture was stirred at 40 °C for 8 h. After cooling to room temperature, the reaction mixture was diluted with dichloromethane (20 mL) and washed with water (20 mL). The aqueous layer was extracted with dichloromethane (20 mL), and the combined organic layers were washed with brine (40 mL). The organic phase was dried (Na_2SO_4), filtered, and concentrated *in vacuo*. The residue was purified by column chromatography on silica gel to afford aryl thiocyanate **S2**.

General Procedure E: Synthesis of aryl sulfonyl cyanides (2).[20]
Hydrogen peroxide (50 wt.% solution in water, 10 eq.) was added dropwise at 0 °C to a solution of trifluoroacetic anhydride (10 eq.) in dichloromethane (0.5 M) in a two-necked round bottom flask equipped with a condenser and a glass cap. After being stirred for 40 minutes at 0 °C, the thiocyanate (1.0 eq.) was added dropwise without any dilution, and the reaction mixture was stirred at 40 °C for 16 hours. At 0 °C, the reaction was quenched with water. The aqueous layer was extracted with dichloromethane, and the combined organic layers were washed with water and brine, dried over Na_2SO_4 and concentrated under reduced pressure. The obtained crude sulfonyl cyanides were pure enough and were used directly without purification. These compounds can be stored at –20 °C for months.

General Procedure F: Synthesis of aryl sulfinyl cyanides (4).[21]
Hydrogen peroxide (50 wt.% solution in water, 10 eq.) was added dropwise at 0 °C to a solution of trifluoroacetic anhydride (10 eq.) in dichloromethane (0.5 M) in a two-necked round bottom flask equipped with a condenser and a glass cap. After being stirred for 40 minutes at 0 °C, the thiocyanate (1.0 eq.) was added dropwise without any dilution, and the reaction mixture was stirred at 0 °C for 3 hours. At 0 °C, the reaction was quenched with water. The aqueous layer was extracted with dichloromethane, and the combined organic layers were washed with water and brine, dried over Na_2SO_4 and concentrated under reduced pressure. The obtained crude sulfonyl cyanides were pure enough and were used directly without purification.

Thiocyanatobenzene (S2b).

Following general procedure C described above (10 mmol scale), the product was obtained as a colorless oil (621 mg, 4.55 mmol, 46%). Purification was achieved by silica column chromatography (PE).

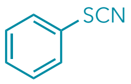

^{1}H NMR (400 MHz, $CDCl_3$) δ = 7.59 – 7.49 (m, 2H), 7.48 – 7.38 (m, 3H).
^{13}C NMR (101 MHz, $CDCl_3$) δ = 130.4, 130.2, 129.7, 124.6, 110.7.33
HRMS (EI): Calcd. for C_7H_5NS $[M]^+$: 135.01372, Found: 135.01341.

Benzenesulfonyl cyanide (2b).

Following general procedure E described above (1.5 mmol scale), the product was obtained as a colorless oil (230 mg, 1.38 µmol, 93%) and used without further purification.

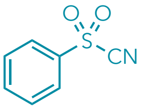

^{1}H NMR (400 MHz, $CDCl_3$) δ = 8.14 – 8.06 (m, 2H), 7.93 – 7.83 (m, 1H), 7.80 – 7.67 (m, 2H).
^{13}C NMR (101 MHz, $CDCl_3$) δ = 137.5, 137.2, 130.5, 129.2, 114.1.

Benzenesulfinyl cyanide (4b).

Following general procedure F described above (1.5 mmol scale), the product was obtained as a colorless oil (210 mg, 1.39 µmol, 94%) and used without further purification.

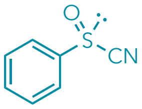

^{1}H NMR (300 MHz, $CDCl_3$) δ = 7.95 – 7.87 (m, 2H), 7.78 – 7.63 (m, 3H).
^{13}C NMR (75 MHz, $CDCl_3$) δ = 139.0, 134.2, 130.6, 125.7, 116.0.
HRMS (EI): Calcd. for C_7H_5NOS $[M]^+$: 151.00864, Found: 151.00840.

1-Fluoro-4-thiocyanatobenzene (S2c).

Following general procedure C described above (10 mmol scale), the product was obtained as a faint yellow oil (1.50 g, 9.81 mmol, 83%). Purification was achieved by silica column chromatography (PE).

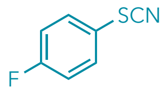

^{1}H NMR (400 MHz, $CDCl_3$) δ = 7.63 – 7.51 (m, 2H), 7.21 – 7.10 (m, 2H).
^{13}C NMR (101 MHz, $CDCl_3$) δ = 163.8 (d, *J*=251.9), 133.4 (d, *J*=8.7), 119.4 (d, *J*=3.5), 117.8 (d, *J*=22.5), 110.7.
^{19}F NMR (376 MHz, $CDCl_3$) δ = -110.1.
The analytical data are in accordance with those reported in the literature.[22]

4-Fluorobenzenesulfonyl cyanide (2c).

Following general procedure E described above (2.0 mmol scale), the product was obtained as a yellow oil (201 mg, 1.09 mmol, 54%) and used without further purification.

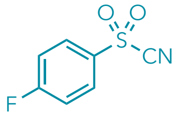

^{1}H NMR (400 MHz, $CDCl_3$) δ = 8.19 – 8.09 (m, 2H), 7.47 – 7.34 (m, 2H).
^{13}C NMR (101 MHz, $CDCl_3$) δ = 168.0 (d, *J*=263.1), 133.4 (d, *J*=3.0), 132.6 (d, *J*=10.8), 118.2 (d, *J*=23.0), 114.0.
^{19}F NMR (376 MHz, $CDCl_3$) δ = -96.3.
HRMS (EI): Calcd. for $C_7H_4NO_2FS$ $[M]^+$: 184.99413, Found: 184.99387.

4-Fluorobenzenesulfinyl cyanide (4c).

Following general procedure F described above (2.0 mmol scale), the product was obtained as a colorless oil (163 mg, 1.64 mmol, 82%) and used without further purification.

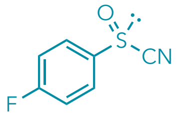

^{1}H NMR (400 MHz, $CDCl_3$) δ = 7.93 (dd, *J*=8.8, 4.8, 2H), 7.42 – 7.34 (m, 2H).
^{13}C NMR (101 MHz, $CDCl_3$) δ = 166.0 (d, *J*=257.1), 134.6 (d, *J*=3.0), 128.5 (d, *J*=9.5), 118.0 (d, *J*=23.0), 115.7.
^{19}F NMR (376 MHz, $CDCl_3$) δ = -102.6.
HRMS (EI): Calcd. for C_7H_4FNOS $[M]^+$: 168.99921, Found: 168.99959.

1-Bromo-4-thiocyanatobenzene (S2d).

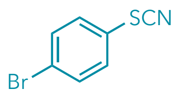

Following general procedure C described above (10 mmol scale), the product was obtained as a yellow oil (2.04 g, 9.54 mmol, 95%). Purification was achieved by silica column chromatography (PE).

^{1}H NMR (400 MHz, $CDCl_3$) δ = 7.59 – 7.51 (m, 2H), 7.44 – 7.34 (m, 2H).
^{13}C NMR (101 MHz, $CDCl_3$) δ = 133.5, 131.6, 124.2, 123.6, 109.9.
The analytical data are in accordance with those reported in the literature.[22]

4-Bromobenzenesulfonyl cyanide (2d).

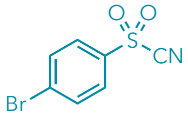

Following general procedure E described above (2.0 mmol scale), the product was obtained as a white solid (235 mg, 955 μmol, 48%) and used without further purification.

^{1}H NMR (400 MHz, $CDCl_3$) δ = 7.98 – 7.91 (m, 2H), 7.91 – 7.84 (m, 2H).
^{13}C NMR (101 MHz, $CDCl_3$) δ = 136.2, 134.0, 133.6, 130.5, 113.8.
HRMS (EI): Calcd. for $C_7H_4BrNO_2S$ $[M]^+$: 244.91406, Found: 244.91363.

4-Bromobenzenesulfinyl cyanide (4d).

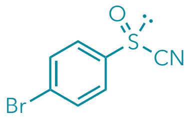

Following general procedure F described above (2.0 mmol scale), the product was obtained as a white solid (424 mg, 1.84 mmol, 92%) and used without further purification.

^{1}H NMR (400 MHz, $CDCl_3$) δ = 7.81 (d, *J*=8.6, 2H), 7.75 (d, *J*=8.6, 2H).
^{13}C NMR (101 MHz, $CDCl_3$) δ = 137.9, 133.8, 129.3, 127.0, 115.6.
HRMS (EI): Calcd. for C_7H_4BrNOS $[M]^+$: 228.91917, Found: 228.91915.

1-Methoxy-4-thiocyanatobenzene (S2e).

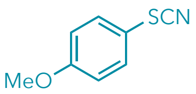

Following general procedure C described above (10 mmol scale), the product was obtained as a yellow oil (1.57 g, 9.52 mmol, 95%). Purification was achieved by silica column chromatography (PE/EtOAc 0% → 5%).

^{1}H NMR (400 MHz, $CDCl_3$) δ = 7.58 – 7.43 (m, 2H), 7.04 – 6.83 (m, 2H), 3.83 (s, 3H).
^{13}C NMR (101 MHz, $CDCl_3$) δ = 161.4, 133.9, 116.0, 113.9, 111.7, 55.6.
The analytical data are in accordance with those reported in the literature.[22]

4-Methoxybenzenesulfonyl cyanide (2e).

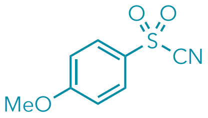

Following general procedure E described above stirred at 25 °C instead of 40 °C (2.0 mmol scale), the product was obtained as a yellow oil (58.0 mg, 294 µmol, 15%) and used without further purification.

^{1}H NMR (400 MHz, $CDCl_3$) δ = 8.05 – 7.96 (m, 2H), 7.18 – 7.07 (m, 2H), 3.96 (s, 3H).
^{13}C NMR (101 MHz, $CDCl_3$) δ = 166.7, 131.9, 128.3, 115.7, 114.6, 56.3.
The analytical data are in accordance with those reported in the literature.[17]

5-Thiocyanatobenzo[*d*][1,3]dioxole (S2f).

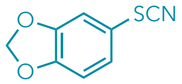

Following general procedure D described above (5.0 mmol scale), the product was obtained as a colorless oil (785 mg, 4.38 mmol, 88%). Purification was achieved by silica column chromatography (PE/EtOAc 0% → 10%).

^{1}H NMR (400 MHz, $CDCl_3$) δ = 7.06 (dd, *J*=8.0, 2.0, 1H), 7.04 (dd, *J*=2.0, 0.4, 1H), 6.84 (dd, *J*=8.0, 0.4, 1H), 6.04 (s, 2H).
^{13}C NMR (101 MHz, $CDCl_3$) δ = 149.9, 149.2, 126.7, 115.2, 112.2, 111.4, 109.7, 102.3.
HRMS (EI): Calcd. for $C_8H_5NO_2S$ $[M]^+$: 179.00355, Found: 179.00369.

Benzo[*d*][1,3]dioxole-5-sulfonyl cyanide (2f).

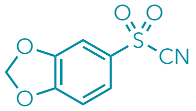

Following general procedure E described above stirred at 25 °C instead of 40 °C (2.0 mmol scale), the product was obtained as a colorless oil (47.0 mg, 223 µmol, 11%) and used without further purification.

^{1}H NMR (400 MHz, $CDCl_3$) δ = 7.67 (dd, *J*=8.3, 2.0, 1H), 7.40 (d, *J*=2.0, 1H), 7.04 (d, *J*=8.3, 1H), 6.21 (s, 2H).
^{13}C NMR (101 MHz, $CDCl_3$) δ = 155.5, 149.4, 129.8, 126.9, 114.3, 109.5, 108.4, 103.6.
HRMS (ESI): Calcd. for $C_8H_5NO_4S$ $[M\text{-}CN]^+$: 184.9904, Found: 184.9902.

2-Chloro-1-methoxy-4-thiocyanatobenzene (S2g).

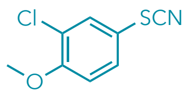

Following general procedure D described above (5.0 mmol scale), the product was obtained as a yellow oil (631 mg, 3.16 mmol, 63%). Purification was achieved by silica column chromatography (PE/EtOAc 0% → 10%).

^{1}H NMR (400 MHz, $CDCl_3$) δ = 7.60 (d, *J*=2.4, 1H), 7.46 (dd, *J*=8.7, 2.4, 1H), 6.97 (d, *J*=8.7, 1H), 3.93 (s, 3H).
^{13}C NMR (101 MHz, $CDCl_3$) δ = 157.0, 133.6, 131.9, 124.4, 114.8, 113.3, 110.9, 56.6.
HRMS (EI): Calcd. for C_8H_6ClNOS $[M]^+$: 198.98531, Found: 198.98535.

3-Chloro-4-methoxybenzenesulfonyl cyanide (2g).

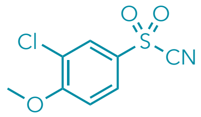

Following general procedure E described above stirred at 25 °C instead of 40 °C (2.0 mmol scale), the product was obtained as a yellow oil (409 mg, 1.77 mmol, 88%) and used without further purification.

^{1}H NMR (400 MHz, $CDCl_3$) δ = 8.06 (d, *J*=2.4, 1H), 7.97 (dd, *J*=8.8, 2.4, 1H), 7.16 (d, *J*=8.9, 1H), 4.06 (s, 3H).
^{13}C NMR (101 MHz, $CDCl_3$) δ = 162.2, 131.1, 130.3, 128.9, 125.2, 114.2, 112.7, 57.2.
HRMS (EI): Calcd. for $C_8H_6ClNO_3S$ $[M]^+$: 230.97514, Found: 230.97485.

1-Bromo-4-(4-thiocyanatophenoxy)benzene (S2h).

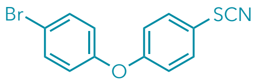

Following general procedure D described above (5.0 mmol scale), the product was obtained as a colorless oil (956 mg, 3.12 mmol, 62%). Purification was achieved by silica column chromatography (PE/EtOAc 0% → 10%).

^{1}H NMR (400 MHz, $CDCl_3$) δ = 7.57 – 7.44 (m, 4H), 7.07 – 6.98 (m, 2H), 6.96 – 6.87 (m, 2H).
^{13}C NMR (101 MHz, $CDCl_3$) δ = 159.1, 155.1, 133.5, 133.2, 121.6, 120.0, 117.5, 117.3, 111.1.
HRMS (EI): Calcd. for $C_{13}H_8BrNOS$ $[M]^+$: 304.95045, Found: 304.94981.

4-(4-Bromophenoxy)benzenesulfonyl cyanide (2h).

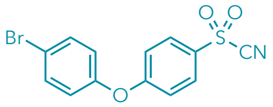

Following general procedure E described above stirred at 25 °C instead of 40 °C (2.0 mmol scale), the product was obtained as a faint yellow solid (450 mg, 1.33 mmol, 67%) and used without further purification.

^{1}H NMR (400 MHz, $CDCl_3$) δ = 8.06 (d, *J*=2.4, 1H), 7.97 (dd, *J*=8.8, 2.4, 1H), 7.16 (d, *J*=8.9, 1H), 4.06 (s, 3H).
^{13}C NMR (101 MHz, $CDCl_3$) δ = 162.2, 131.1, 130.3, 128.9, 125.2, 114.2, 112.7, 57.2.
HRMS (ESI): Calcd. for $C_{13}H_8BrNO_3S$ $[M-CN]^+$: 310.9378, Found: 310.9369.

4-Isopropyl-1-methoxy-2-thiocyanatobenzene (S2i).

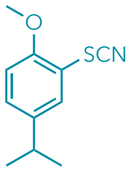

Following general procedure D described above (5.0 mmol scale), the product was obtained as a yellow oil (640 mg, 3.09 mmol, 62%). Purification was achieved by silica column chromatography (PE/EtOAc 0% → 10%).

^{1}H NMR (400 MHz, $CDCl_3$) δ = 7.40 (dd, *J*=2.1, 0.6, 1H), 7.21 (ddd, *J*=8.4, 2.2, 0.6, 1H), 6.86 (d, *J*=8.4, 1H), 3.89 (s, 3H), 2.90 (hept, *J*=7.0, 1H), 1.24 (d, *J*=6.9, 6H).
^{13}C NMR (101 MHz, $CDCl_3$) δ = 154.8, 143.1, 128.5, 128.2, 112.6, 111.6, 110.8, 56.4, 33.6, 24.2.
HRMS (EI): Calcd. for $C_{11}H_{13}NOS$ $[M]^+$: 207.07124, Found: 207.07119.
The analytical data are in accordance with those reported in the literature.[23]

5-Isopropyl-2-methoxybenzenesulfonyl cyanide (2i).

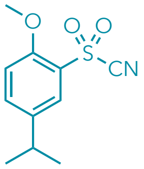

Following general procedure E described above although stirred at 25 °C instead of 40 °C (2.0 mmol scale), the product was obtained as a faint yellow solid (310 mg, 1.30 mmol, 65%) and used without further purification.

^{1}H NMR (400 MHz, $CDCl_3$) δ = 7.76 (d, *J*=2.5, 1H), 7.62 (ddd, *J*=8.6, 2.3, 0.6, 1H), 7.08 (d, *J*=8.6, 1H), 4.06 (s, 3H), 2.96 (hept, *J*=6.9, 1H), 1.26 (d, *J*=7.0, 6H).
^{13}C NMR (101 MHz, $CDCl_3$) δ = 157.1, 142.2, 137.2, 128.0, 124.7, 114.2, 113.3, 56.9, 33.4, 23.9.
HRMS (EI): Calcd. for $C_{11}H_{13}NO_3S$ $[M]^+$: 239.06107, Found: 239.06129.

2-Methoxy-5-thiocyanatophenyl acetate (S2j).

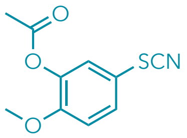

Following general procedure D described above (5.0 mmol scale), the product was obtained as a white solid (835 mg, 3.74 mmol, 75%). Purification was achieved by silica column chromatography (PE/EtOAc 0% → 10%).

^{1}H NMR (400 MHz, $CDCl_3$) δ = 7.42 (dd, *J*=8.7, 2.4, 1H), 7.28 (d, *J*=2.4, 1H), 7.00 (d, *J*=8.7, 1H), 3.86 (s, 3H), 2.32 (s, 3H).
^{13}C NMR (101 MHz, $CDCl_3$) δ = 168.5, 153.2, 140.8, 131.0, 126.6, 114.1, 113.8, 111.0, 56.3, 20.6.
HRMS (EI): Calcd. for $C_{10}H_9NO_3S$ $[M]^+$: 223.02977, Found: 223.02942.

5-(Cyanosulfonyl)-2-methoxyphenyl acetate (2j).

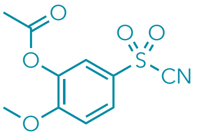

Following general procedure E described above although stirred at 25 °C instead of 40 °C (2.0 mmol scale), an approx. 3:2 mixture of desired and de-acetylated compound was obtained (colorless oil, 287 mg). The desired product was purified by flash column chromatography (PE/EtOAc 0% → 30%) to yield a white solid (99.0 mg, 388 µmol, 19%).

^{1}H NMR (400 MHz, $CDCl_3$) δ = 7.96 (dd, *J*=8.8, 2.4, 1H), 7.75 (d, *J*=2.4, 1H), 7.19 (d, *J*=8.9, 1H), 3.99 (s, 3H), 2.35 (s, 3H).
^{13}C NMR (101 MHz, $CDCl_3$) δ = 168.0, 158.8, 140.7, 129.7, 128.2, 124.2, 114.3, 113.2, 56.9, 20.6.
HRMS (EI): Calcd. for $C_{10}H_9NO_5S$ $[M]^+$: 255.01959, Found: 255.01925.

5.4 Alkyl sulfonyl and sulfinyl cyanides

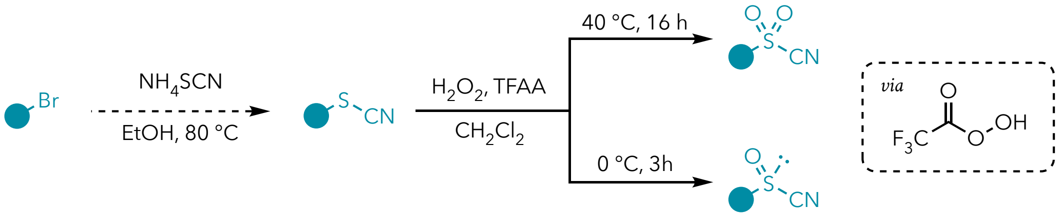

Aryl sulfonyl and sulfinyl cyanides were synthesized according to modified literature procedures.[17,24]

General Procedure G: Synthesis of alkyl thiocyanates (S2).
To a solution of ammonium thiocyanate (1.2 equiv.) in EtOH was added alkyl halide (1.0 equiv.). The reaction mixture was heated to reflux for 16 hours. Then, the reaction was allowed to reach room temperature, diluted with water and extracted with DCM. The combined organic extracts were washed with brine, dried over Na_2SO_4 and concentrated under reduced pressure. The obtained crude product was pure enough and was used directly without further purification.

General Procedure E: Synthesis of alkyl sulfonyl cyanides (2).
Hydrogen peroxide (50 wt.% solution in water, 10 eq.) was added dropwise at 0 °C to a solution of trifluoroacetic anhydride (10 eq.) in dichloromethane (0.5 M) in a two-necked round bottom flask equipped with a condenser and a glass cap. After being stirred for 40 minutes at 0 °C, the thiocyanate (1.0 eq.) was added dropwise without any dilution, and the reaction mixture was stirred at 40 °C for 16 hours. At 0 °C, the reaction was quenched with water. The aqueous layer was extracted with dichloromethane, and the combined organic layers were washed with water and brine, dried over Na_2SO_4 and concentrated under reduced pressure. The obtained crude sulfonyl cyanides were pure enough and were used directly without purification. These compounds can be stored at –20 °C under nitrogen atmosphere for months.

General Procedure F: Synthesis of alkyl sulfinyl cyanides (4).
Hydrogen peroxide (50 wt.% solution in water, 10 eq.) was added dropwise at 0 °C to a solution of trifluoroacetic anhydride (10 eq.) in dichloromethane (0.5 M) in a two-necked round bottom flask equipped with a condenser and a glass cap. After being stirred for 40 minutes at 0 °C, the thiocyanate (1.0 eq.) was added dropwise without any dilution, and the reaction mixture was stirred at 0 °C for 3 hours. At 0 °C, the reaction was quenched with water. The aqueous layer was extracted with dichloromethane, and the combined organic layers were washed with water and brine, dried over Na_2SO_4 and concentrated under reduced pressure. The obtained crude sulfonyl cyanides were pure enough and were used directly without purification.

Phenylmethanesulfonyl cyanide (2k).

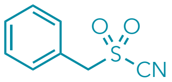

Following general procedure E described above starting from benzyl thiocyanate (10 mmol scale), the product was obtained as a white solid (1.38 g, 7.60 mmol, 76%).

^{1}H NMR (400 MHz, $CDCl_3$) δ = 7.60 – 7.37 (m, 5H), 4.61 (s, 2H).
^{13}C NMR (101 MHz, $CDCl_3$) δ = 131.6, 130.9, 129.8, 123.5, 111.9, 64.8.
The analytical data are in accordance with those reported in the literature.[21]

Phenylmethanesulfinyl cyanide (4e).

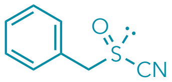

Following general procedure F described above starting from benzyl thiocyanate (5 mmol scale), the product was obtained as a white solid (693 mg, 4.20 mmol, 84%, contains impurity of sulfonyl cyanide).
^{1}H NMR (400 MHz, $CDCl_3$) δ = 7.50 – 7.27 (m, 5H), 4.54 (d, *J*=13.0, 1H), 4.44 (d, *J*=12.8, 1H).
^{13}C NMR (101 MHz, $CDCl_3$) δ = 130.8, 130.2, 129.5, 126.2, 111.8, 61.7.

Methanesulfonyl cyanide (2l).

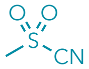

Following general procedure E described above starting from methyl thiocyanate (10 mmol scale), the product was obtained as a colorless oil (230 mg, 2.19 mmol, 22%).

^{1}H NMR (400 MHz, $CDCl_3$) δ = 3.43 (s, 3H).
^{13}C NMR (101 MHz, $CDCl_3$) δ = 113.3, 46.6.
The analytical data are in accordance with those reported in the literature.[24]

Ethanesulfonyl cyanide (2m).

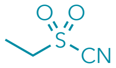

Following general procedure E described above starting from ethyl thiocyanate (10 mmol scale), the product was obtained as a colorless oil (946 mg, 7.94 mmol, 79%).

^{1}H NMR (400 MHz, $CDCl_3$) δ = 3.43 (q, *J*=7.4, 2H), 1.60 (t, *J*=7.4, 3H).
^{13}C NMR (101 MHz, $CDCl_3$) δ = 112.3, 53.3, 7.2.
The analytical data are in accordance with those reported in the literature.[24]

2-Thiocyanatopropane (S2n).

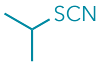

This step was carried out according to a modified literature procedure.[25]
Potassium thiocyanate (14.6 g, 150 mmol, 1.5 eq.) was dispersed in ethanol (50 mL). The mixture was heated to 80 °C followed by slow addition of isopropyl bromide (9.4 mL, 100 mmol, 1.0 eq.) over 20 min. After the mixture was refluxed for 20 h, the solution was cooled down to ambient temperature, the precipitate filtered off and washed with ethanol. After removal of the majority of ethanol *in vacuo*, the mixture was diluted with water, extracted with ethyl acetate (3x), the combined organic phases were dried over Na_2SO_4, and the solvent removed in vacuo. The resulting colorless crude mixture was then purified by distillation to obtain the product as colorless oil (4.16 g, 41.1 mmol, 41%).

$\boldsymbol{T_B}$ = 145-151 °C
^{1}H NMR (400 MHz, $CDCl_3$) δ = 3.48 (hept, *J*=6.7, 1H), 1.49 (d, *J*=6.7, 6H).
^{13}C NMR (101 MHz, $CDCl_3$) δ = 111.6, 40.7, 23.7.
The analytical data are in accordance with those reported in the literature.[17]

Propane-2-sulfonyl cyanide (2n).

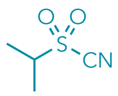

Following general procedure described above starting from 2-thiocyanatopropane (10 mmol scale), the product was obtained as a colorless oil (1.13 g, 8.45 mmol, 84%).

^{1}H NMR (400 MHz, $CDCl_3$) δ = 3.44 (hept, *J*=6.9, 1H), 1.57 (d, *J*=6.9, 6H).
^{13}C NMR (101 MHz, $CDCl_3$) δ = 111.5, 59.2, 15.1.
The analytical data are in accordance with those reported in the literature.[17]

Ethyl 2-thiocyanatoacetate (S2o).

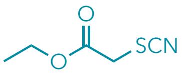

Following general procedure described above starting from ethyl 2-bromoacetate (10 mmol scale), the crude product was obtained as a yellow oil (1.45 g) and the purified by vacuum distillation to obtain the product as colorless oil (719 mg, 4.95 mmol, 50%).

T_B = 120 °C (10 mbar)
^{1}H NMR (400 MHz, $CDCl_3$) δ = 4.29 (q, *J*=7.2, 2H), 3.77 (s, 2H), 1.33 (t, *J*=7.2, 3H).
^{13}C NMR (101 MHz, $CDCl_3$) δ = 166.3, 110.8, 63.1, 35.2, 14.2.
The analytical data are in accordance with those reported in the literature.[24]

Ethyl 2-(cyanosulfonyl)acetate (2o).

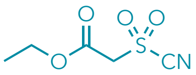

Following general procedure described above starting from ethyl 2-thiocyanatoacetate (10 mmol scale), the product was obtained as a colorless oil (402 mg, 2.27 mmol, 23%).

^{1}H NMR (400 MHz, $CDCl_3$) δ 4.39 (q, *J* = 7.2 Hz, 2H), 4.36 (s, 2H), 1.38 (t, *J* = 7.2 Hz, 3H).
^{13}C NMR (101 MHz, $CDCl_3$) δ = 159.3, 111.9, 67.2, 64.1, 61.3, 13.8.
The analytical data are in accordance with those reported in the literature.[24]

1-Chloro-3-thiocyanatopropane (S2p).

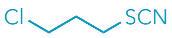

Following general procedure described above starting from 1-bromo-3-chloropropane (10 mmol scale), the crude product was obtained as a colorless oil (1.30 g) with small impurities. The product was purified by distillation to obtain a colorless oil (373 mg, 2.75 mmol, 28%).

T_B = 93 °C (10 mbar)
^{1}H NMR (400 MHz, $CDCl_3$) δ = 3.77 – 3.65 (m, 2H), 3.13 (t, *J*=6.8, 2H), 2.36 – 2.24 (m, 2H).
^{13}C NMR (101 MHz, $CDCl_3$) δ = 111.7, 42.0, 32.1, 31.0.
The analytical data are in accordance with those reported in the literature.[26]

3-Chloropropane-1-sulfonyl cyanide (2p).

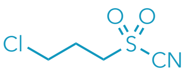

Following general procedure described above starting from 1-chloro-3-thiocyanatopropane (2 mmol scale), the product was obtained as a colorless oil (204 mg, 1.22 mmol, 61%).

^{1}H NMR (400 MHz, $CDCl_3$) δ = 3.76 – 3.72 (m, 2H), 3.67 – 3.58 (m, 2H), 2.54 – 2.40 (m, 2H).
^{13}C NMR (101 MHz, $CDCl_3$) δ = 112.4, 55.9, 41.4, 25.6.
HRMS (ESI): Calcd. for $C_4H_6ClNO_2S$ $[M+H]^+$: 167.9881, Found: 167.9884.

(Thiocyanatomethyl)cyclopropane (S2q).

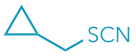

Following general procedure described above starting from 1-bromo-3-chloropropane (10 mmol scale), the product was obtained as a colorless oil (1.09 g, 9.67 mmol, 94%) and directly used without further purification.

^{1}H NMR (400 MHz, $CDCl_3$) δ = 2.93 (d, *J*=7.4, 2H), 1.28 – 1.16 (m, 1H), 0.83 – 0.67 (m, 2H), 0.46 – 0.33 (m, 2H).
^{13}C NMR (101 MHz, $CDCl_3$) δ = 112.7, 40.4, 11.6, 6.2.
The analytical data are in accordance with those reported in the literature.[24]

3-Chloropropane-1-sulfonyl cyanide (2q).

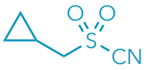

Following general procedure described above starting from (thiocyanatomethyl)cyclopropane (10 mmol scale), the product was obtained as a colorless oil (1.25 g, 8.60 mmol, 86%).

^{1}H NMR (400 MHz, $CDCl_3$) δ = 3.33 (d, *J*=7.3, 2H), 1.34 – 1.22 (m, 1H), 0.98 – 0.88 (m, 2H), 0.66 – 0.54 (m, 2H).
^{13}C NMR (101 MHz, $CDCl_3$) δ = 113.3, 63.6, 4.9, 3.9.
The analytical data are in accordance with those reported in the literature.[24]

Methyl 4-(thiocyanatomethyl)benzoate (S2r).

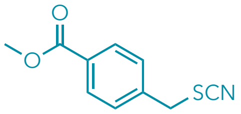

Following general procedure described above (10 mmol scale), the product was obtained as a colorless oil (2.02 g, 9.73 mmol, 97%) and directly used without further purification.

^{1}H NMR (400 MHz, $CDCl_3$) δ = 8.15 – 8.03 (m, 2H), 7.50 – 7.39 (m, 2H), 4.19 (s, 2H), 3.94 (s, 3H).
^{13}C NMR (101 MHz, $CDCl_3$) δ = 166.5, 139.4, 130.8, 130.5, 129.1, 111.5, 52.4, 37.8.
The analytical data are in accordance with those reported in the literature.[27]

Methyl 4-((cyanosulfonyl)methyl)benzoate (2r).

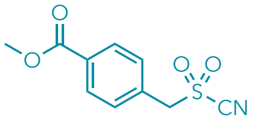

Following general procedure described above (10 mmol scale), the product was obtained as a white solid (1.83 g, 7.66 mmol, 77%) and directly used without further purification.

^{1}H NMR (400 MHz, $CDCl_3$) δ = 8.20 – 8.13 (m, 2H), 7.58 – 7.50 (m, 2H), 4.67 (s, 2H), 3.95 (s, 3H).
^{13}C NMR (101 MHz, $CDCl_3$) δ = 166.1, 132.6, 131.6, 130.8, 128.1, 111.6, 64.3, 52.7.
HRMS (ESI): Calcd. for $C_{10}H_9NO_4S$ $[M+H]^+$: 240.0330, Found: 240.0325.

3-Fluoro-4-(thiocyanatomethyl)benzonitrile (S2s).

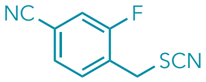

Following general procedure described above (1.66 mmol scale), the product was obtained as a white solid (319 mg, 1.66 mmol, quant.) and directly used without further purification.

^{1}H NMR (400 MHz, $CDCl_3$) δ = 7.58 – 7.49 (m, 2H), 7.49 – 7.39 (m, 1H), 4.18 (d, *J*=1.3, 2H).
^{13}C NMR (101 MHz, $CDCl_3$) δ = 160.2 (d, *J*=253.2), 132.1 (d, *J*=3.5), 128.9 (d, *J*=4.3), 128.1 (d, *J*=14.7), 119.8 (d, *J*=24.7), 117.0 (d, *J*=3.0), 114.8 (d, *J*=9.5), 110.8, 31.1 (d, *J*=3.5).
^{19}F NMR (376 MHz, $CDCl_3$) δ = -113.4.
HRMS (EI): Calcd. for $C_9H_5N_2FS$ $[M]^+$: 192.01520, Found: 192.01560.

(4-Cyano-2-fluorophenyl)methanesulfonyl cyanide (2s).

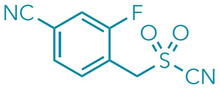

Following general procedure described above (1.66 mmol scale), the product was obtained as a white solid (267 mg, 1.19 mmol, 72%) and directly used without further purification.

^{1}H NMR (400 MHz, $CDCl_3$) δ = 7.73 – 7.50 (m, 3H), 4.77 (s, 2H).
^{13}C NMR (101 MHz, $CDCl_3$) δ = 161.19 (d, *J*=256.2), 134.06 (d, *J*=2.6), 129.17 (d, *J*=4.3), 120.42 (d, *J*=24.7), 117.19 (d, *J*=14.7), 116.73 (d, *J*=42.5), 111.58, 57.60 (d, *J*=2.2).
^{19}F NMR (376 MHz, $CDCl_3$) δ = -111.02.
HRMS (ESI): Calcd. for $C_9H_5FN_2O_2S$ $[M\text{-}CN+2H]^+$: 200.0177, Found: 200.0179.

Diethyl (3-thiocyanatopropyl)phosphonate (S2t).

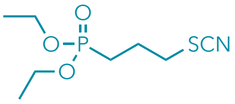

Following general procedure described above starting from diethyl (3-bromopropyl)phosphonate (6 mmol scale), the product was obtained as a colorless oil and directly used without further purification.

^{1}H NMR (400 MHz, $CDCl_3$) δ = 4.21 – 4.01 (m, 4H), 3.07 (t, *J*=7.1, 2H), 2.24 – 2.07 (m, 2H), 1.97 – 1.84 (m, 3H), 1.34 (t, *J*=7.1, 6H).
^{13}C NMR (101 MHz, $CDCl_3$) δ = 111.69, 61.90 (d, *J*=6.5), 34.04 (d, *J*=14.7), 23.81 (d, *J*=143.1), 23.34 (d, *J*=4.8), 16.48 (d, *J*=6.1).
^{31}P NMR ($CDCl_3$, 162 MHz): δ = 30.17.

Diethyl (3-(cyanosulfonyl)propyl)phosphonate (2t).

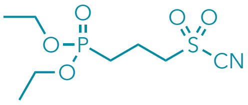

Following general procedure described above starting from diethyl (3-thiocyanatopropyl)phosphonate (5 mmol scale), the product was obtained as a colorless oil (1.12 g, 4.16 mmol, 83%).

^{1}H NMR (400 MHz, $CDCl_3$) δ = 4.22 – 4.07 (m, 4H), 3.87 – 3.31 (m, 2H), 2.51 – 2.12 (m, 2H), 2.00 (dt, *J*=18.6, 7.3, 2H), 1.36 (t, *J*=7.1, 6H).
^{13}C NMR (101 MHz, $CDCl_3$) δ = 112.5, 62.9 (d, *J*=6.9), 57.8 (d, *J*=11.3), 23.4 (d, *J*=144.4), 16.5, 16.5, 16.4.
^{31}P NMR (162 MHz, $CDCl_3$) δ = 29.04.
HRMS (ESI): Calcd. for $C_8H_{16}NO_5PS$ $[M+H]^+$: 270.0560, Found: 270.0566.

2-(3-Thiocyanatopropyl)isoindoline-1,3-dione (S2u).

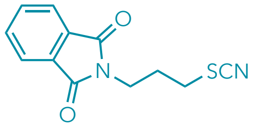

Following general procedure described above starting from 2-(3-bromopropyl)isoindoline-1,3-dione (10 mmol scale), the product was obtained as a white solid (2.43 g, 9.88 mmol, 99%) and directly used without further purification.

^{1}H NMR (400 MHz, $CDCl_3$) δ = 7.87 – 7.78 (m, 2H), 7.76 – 7.68 (m, 2H), 3.84 (t, *J*=6.4, 2H), 2.97 (t, *J*=7.2, 2H), 2.25 – 2.13 (m, 2H).

^{13}C NMR (101 MHz, $CDCl_3$) δ = 168.3, 134.3, 131.9, 123.5, 111.9, 35.9, 31.6, 29.3.

HRMS (ESI): Calcd. for $C_{12}H_{10}N_2O_2S$ $[M+H]^+$: 247.0536, Found: 247.0537.

3-(1,3-Dioxoisoindolin-2-yl)propane-1-sulfonyl cyanide (2u).

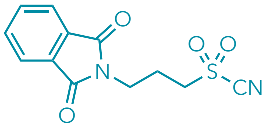

Following general procedure described above starting from 2-(3-thiocyanatopropyl)isoindoline-1,3-dione (10 mmol scale), the product was obtained as a white solid (2.07 g, 7.44 mmol, 74%).

^{1}H NMR (400 MHz, $CDCl_3$) δ = 7.88 (dd, *J*=5.5, 3.0, 2H), 7.76 (dd, *J*=5.5, 3.0, 2H), 3.91 (t, *J*=6.4, 2H), 3.65 – 3.46 (m, 2H), 2.53 – 2.24 (m, 2H).

^{13}C NMR (101 MHz, $CDCl_3$) δ = 168.1, 134.5, 131.7, 123.7, 112.3, 56.3, 35.5, 22.2.

HRMS (ESI): Calcd. for $C_{12}H_{10}N_2O_4S$ $[M+H]^+$: 279.0434, Found: 279.0434.

6 Synthesis of Products

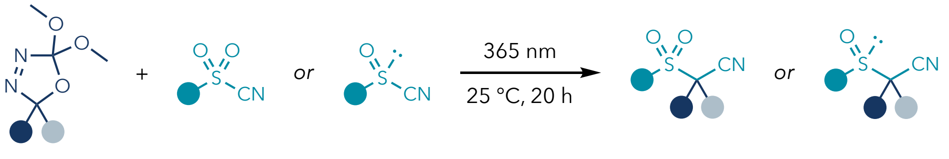

General procedure G: Photochemical C-S bond insertion

To a 5 mL snap vial with a magnetic stirring bar, oxadiazoline (**1**, 300 µmol, 1.2 eq.) and sulfonyl cyanide (**2**, 200 µmol, 1.0 eq.) or sulfinyl cyanide (**4**, 200 µmol, 1.0 eq.) were added. The vial was evacuated and back filled with N2 for three times. Then dry CH_2Cl_2 (2 mL) was added by syringe, and the mixture was irradiated for 20 h with a 365 nm LED at 25 °C. The reaction was quenched by addition of water, the phases separated and the aqueous phase washed with CH_2Cl_2 (2x). The combined organic phases were dried over Na_2SO4, the solvent removed *in vacuo* and the residue purified by silica gel column chromatography to give the desired product.

6.1 Oxadiazoline Scope

1-Tosylcyclohexane-1-carbonitrile (3a).

Following general procedure G described above, the product was obtained as a white solid (49.3 mg, 187 µmol, 94%). Purification was achieved by silica column chromatography (PE/Acetone 0% → 10%).
Structure was confirmed by single-crystal X-ray diffraction.

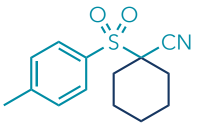

^{1}H NMR (300 MHz, $CDCl_3$) δ = 8.00 – 7.68 (m, 2H), 7.52 – 7.30 (m, 2H), 2.48 (s, 3H), 2.17 – 2.04 (m, 2H), 2.00 – 1.83 (m, 4H), 1.83 – 1.71 (m, 1H), 1.63 – 1.47 (m, 2H), 1.34 – 1.14 (m, 1H).
^{13}C NMR (101 MHz, $CDCl_3$) δ = 146.49, 130.85, 130.81, 130.03, 117.09, 64.45, 29.22, 24.36, 22.60, 21.90.
HRMS (ESI): Calcd. for $C_{14}H_{17}NO_2S$ $[M+H]^+$: 264.1053, Found: 264.1049.

1-Tosylcyclobutane-1-carbonitrile (3b).

Following general procedure G described above, the product was obtained as a white solid (9.5 mg, 40.4 µmol, 20%). Purification was achieved by silica column chromatography (PE/Acetone 0% → 10%).

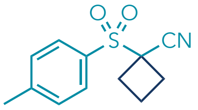

^{1}H NMR (400 MHz, $CDCl_3$) δ = 7.91 – 7.83 (m, 2H), 7.47 – 7.37 (m, 2H), 3.06 – 2.93 (m, 2H), 2.61 – 2.51 (m, 2H), 2.48 (s, 3H), 2.34 – 2.20 (m, 2H).
^{13}C NMR (101 MHz, $CDCl_3$) δ 146.61, 131.49, 130.31, 130.05, 117.93, 57.78, 28.91, 21.93, 16.08.
HRMS (ESI): Calcd. for $C_{12}H_{13}NO_2S$ $[M+H]^+$: 236.0740, Found: 236.0741.

1-Tosylcyclopentane-1-carbonitrile (3c).

Following general procedure G described above, the product was obtained as a white solid (20.5 mg, 74.0 µmol, 37%). Purification was achieved by silica column chromatography (PE/Acetone 0% → 10%).

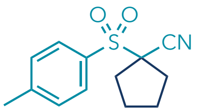

^{1}H NMR (400 MHz, $CDCl_3$) δ = 7.99 – 7.81 (m, 2H), 7.50 – 7.35 (m, 2H), 2.57 – 2.46 (m, 5H), 2.23 – 2.13 (m, 2H), 2.00 – 1.84 (m, 4H).

^{13}C NMR (101 MHz, $CDCl_3$) δ = 146.5, 132.5, 130.3, 130.2, 119.2, 66.7, 35.0, 25.6, 21.9.

HRMS (ESI): Calcd. for $C_{13}H_{15}NO_2S$ $[M+H]^+$: 250.0896, Found: 250.0901.

1-Tosylcycloheptane-1-carbonitrile (3d).

Following general procedure G described above, the product was obtained as a white solid (20.5 mg, 74.0 µmol, 37%). Purification was achieved by silica column chromatography (PE/Acetone 0% → 10%).

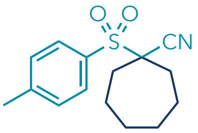

^{1}H NMR (400 MHz, $CDCl_3$) δ = 7.89 (d, *J*=8.4, 2H), 7.44 – 7.36 (m, 2H), 2.48 (s, 3H), 2.25 – 2.11 (m, 4H), 1.93 – 1.84 (m, 2H), 1.73 – 1.54 (m, 7H).

^{13}C NMR (101 MHz, $CDCl_3$) δ = 146.4, 131.2, 131.0, 130.0, 118.2, 66.8, 32.2, 27.4, 23.4, 21.9.

HRMS (ESI): Calcd. for $C_{15}H_{19}NO_2S$ $[M+H]^+$: 278.1209, Found: 278.1211.

4-Tosyltetrahydro-2*H*-pyran-4-carbonitrile (3e).

Following general procedure G described above, the product was obtained as a white solid (43.0 mg, 162 µmol, 81%). Purification was achieved by silica column chromatography (PE/Acetone 0% → 20%).

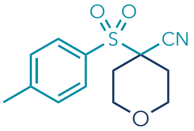

^{1}H NMR (400 MHz, $CDCl_3$) δ = 7.94 – 7.80 (m, 2H), 7.49 – 7.37 (m, 2H), 4.18 – 4.01 (m, 2H), 3.58 (ddd, *J*=12.4, 12.4, 2.1, 2H), 2.48 (s, 3H), 2.28 (ddd, *J*=13.6, 12.3, 4.9, 2H), 1.95 – 1.87 (m, 2H).

^{13}C NMR (101 MHz, $CDCl_3$) δ = 147.0, 130.8, 130.2, 130.2, 116.4, 64.0, 61.9, 29.0, 21.9.

HRMS (ESI): Calcd. for $C_{13}H_{15}NO_3S$ $[M+H]^+$: 266.0845, Found: 266.0848.

***tert*-Butyl 4-cyano-4-tosylpiperidine-1-carboxylate (3f).**

Following general procedure G described above, the product was obtained as a white solid (62.6 mg, 171 µmol, 86%). Purification was achieved by silica column chromatography (PE/Acetone 0% → 20%).
Structure was confirmed by single-crystal X-ray diffraction.

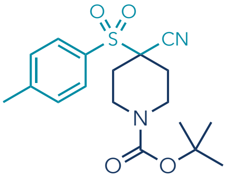

^{1}H NMR (400 MHz, $CDCl_3$) δ = 7.93 – 7.80 (m, 2H), 7.42 (d, *J*=8.1, 2H), 4.29 (s, 2H), 2.88 (s, 2H), 2.47 (s, 3H), 2.08 (td, *J*=12.8, 4.5, 2H), 2.02 (s, 2H), 1.45 (s, 9H).

^{13}C NMR (101 MHz, $CDCl_3$) δ = 154.03, 146.99, 130.80, 130.27, 130.23, 116.11, 80.81, 62.96, 40.62, 28.71, 28.40, 21.90.

HRMS (ESI): Calcd. for $C_{18}H_{24}N_2O_4S$ $[M+H]^+$: 365.1530, Found: 365.1529.

1,4-Ditosylpiperidine-4-carbonitrile (3g).

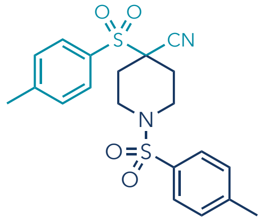

Following general procedure G described above, the product was obtained as a white solid (73.7 mg, 176 μmol, 88%). Purification was achieved by silica column chromatography (PE/Acetone 0% → 20%).
Structure was confirmed by single-crystal X-ray diffraction.

^{1}H NMR (400 MHz, $CDCl_3$) δ = 7.90 – 7.77 (m, 2H), 7.63 – 7.59 (m, 2H), 7.46 – 7.38 (m, 2H), 7.34 – 7.30 (m, 2H), 4.11 – 3.87 (m, 2H), 2.52 – 2.42 (m, 8H), 2.33 – 2.23 (m, 2H), 2.12 – 2.04 (m, 2H).
^{13}C NMR (101 MHz, $CDCl_3$) δ = 147.3, 144.5, 132.9, 130.8, 130.3, 130.2, 130.1, 127.6, 115.7, 62.0, 42.6, 28.7, 21.9, 21.7.
HRMS (ESI): Calcd. for $C_{20}H_{22}N_2O_4S_2$ [M+H]$^+$: 419.1098, Found: 419.1098.

4-(*tert*-Butyl)-1-tosylcyclohexane-1-carbonitrile (3h).

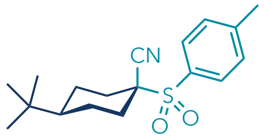

Following general procedure G described above, the product was obtained as a white solid (49.6 mg, 155 μmol, 78%, d.r. >20:1). Purification was achieved by silica column chromatography (PE/Acetone 0% → 20%).
Structure was confirmed by single-crystal X-ray diffraction.

^{1}H NMR (400 MHz, $CDCl_3$) δ = 7.92 – 7.82 (m, 2H), 7.44 – 7.36 (m, 2H), 2.48 (s, 3H), 2.19 – 2.11 (m, 2H), 2.00 – 1.90 (m, 4H), 1.38 – 1.23 (m, 2H), 1.12 – 1.00 (m, 1H), 0.86 (s, 9H).
^{13}C NMR (101 MHz, $CDCl_3$) δ = 146.5, 131.0, 130.8, 130.0, 117.1, 64.4, 46.6, 32.4, 29.7, 27.4, 23.7, 21.9.
HRMS (ESI): Calcd. for $C_{18}H_{25}NO_2S$ [M+H]$^+$: 320.1679, Found: 320.1681.

***tert*-Butyl 8-cyano-8-tosyl-3-azabicyclo[3.2.1]octane-3-carboxylate (3i).**

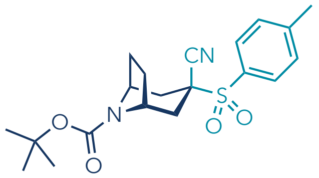

Following general procedure G described above, the product was obtained as a white solid (65.9 mg, 168 μmol, 84%, d.r. >20:1). Purification was achieved by silica column chromatography (PE/Acetone 0% → 20%).
Structure was confirmed by single-crystal X-ray diffraction.

^{1}H NMR (400 MHz, $CDCl_3$) δ = 7.83 (d, *J*=8.4, 2H), 7.45 – 7.37 (m, 2H), 4.35 (d, *J*=8.3, 2H), 2.56 (dd, *J*=13.8, 3.7, 2H), 2.47 (s, 3H), 2.18 – 1.93 (m, 5H), 1.76 (d, *J*=13.1, 1H), 1.49 (s, 9H).
^{13}C NMR (101 MHz, $CDCl_3$) δ = 153.1, 146.7, 131.0, 130.3, 130.0, 120.0, 80.7, 58.1, 52.5, 51.9, 32.9, 32.2, 28.4, 27.6, 26.5, 21.8.
HRMS (ESI): Calcd. for $C_{20}H_{26}N_2O_4S$ [M+Na]$^+$: 413.1505, Found: 413.1508.

2,2-Dimethyl-5-tosyl-1,3-dioxane-5-carbonitrile (3j).

Following general procedure G described above, the product was obtained as a white solid (10.4 mg, 35.2 µmol, 18%). Purification was achieved by silica column chromatography (PE/Acetone 0% → 20%).

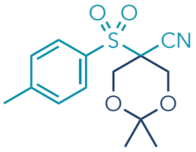

^{1}H NMR (400 MHz, $CDCl_3$) δ = 7.91 – 7.83 (m, 2H), 7.49 – 7.41 (m, 2H), 4.47 (dt, *J*=11.7, 1.0, 2H), 4.05 (dt, *J*=11.8, 1.0, 2H), 2.50 (s, 3H), 1.47 (s, 3H), 1.43 (s, 3H).
^{13}C NMR (101 MHz, $CDCl_3$) δ = 147.5, 131.1, 130.5, 130.3, 115.8, 99.9, 61.3, 59.9, 27.4, 22.0, 19.2.
HRMS (ESI): Calcd. for $C_{14}H_{17}NO_4S$ $[M+H]^+$: 296.0951, Found: 296.0956.

3-Tosyloxetane-3-carbonitrile (3k).

Following general procedure G described above, the product was obtained as a white solid (17.5 mg, 73.7 µmol, 37%). Purification was achieved by silica column chromatography (PE/Acetone 0% → 20%).
Structure was confirmed by single-crystal X-ray diffraction.

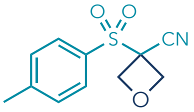

^{1}H NMR (400 MHz, $CDCl_3$) δ = 7.89 (d, *J*=8.4, 2H), 7.45 (dt, *J*=8.0, 0.7, 2H), 5.15 (dd, *J*=7.2, 0.8, 2H), 4.92 (dd, *J*=7.2, 0.8, 2H), 2.49 (s, 3H).
^{13}C NMR (101 MHz, $CDCl_3$) δ = 147.7, 130.9, 130.7, 130.0, 114.8, 73.5, 57.9, 22.0.
HRMS (ESI): Calcd. for $C_{11}H_{11}NO_3S$ $[M+H]^+$: 238.0532, Found: 238.0532.

3-Tosylthietane-3-carbonitrile (3l).

Following general procedure G described above, the product was obtained as a white solid (23.3 mg, 92.0 µmol, 46%). Purification was achieved by silica column chromatography (PE/Acetone 0% → 20%).

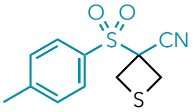

^{1}H NMR (400 MHz, $CDCl_3$) δ = 7.99 – 7.79 (m, 2H), 7.53 – 7.38 (m, 2H), 4.24 – 4.04 (m, 2H), 3.30 – 3.17 (m, 2H), 2.49 (s, 3H).
^{13}C NMR (101 MHz, $CDCl_3$) δ = 147.5, 130.6, 130.4, 129.9, 116.8, 61.7, 28.5, 22.0.
HRMS (ESI): Calcd. for $C_{11}H_{11}NO_2S_2$ $[M+H]^+$: 254.0304, Found: 254.0303.

2-Methyl-2-tosylpropanenitrile (3m).

Following general procedure G described above, the product was obtained as a white solid (29.0 mg, 130 µmol, 65%). Purification was achieved by silica column chromatography (PE/Acetone 0% → 20%).

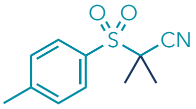

^{1}H NMR (400 MHz, $CDCl_3$) δ = 8.06 – 7.66 (m, 2H), 7.56 – 7.27 (m, 2H), 2.49 (s, 3H), 1.70 (s, 6H).
^{13}C NMR (101 MHz, $CDCl_3$) δ = 146.7, 130.9, 130.6, 130.1, 118.4, 57.7, 21.9, 21.3.
HRMS (ESI): Calcd. for $C_{11}H_{13}NO_2S$ $[M+H]^+$: 224.0740, Found: 224.0743.

5-(4-(Furan-2-carbonyl)piperazin-1-yl)-2-methyl-5-oxo-2-tosylpentanenitrile (3n).

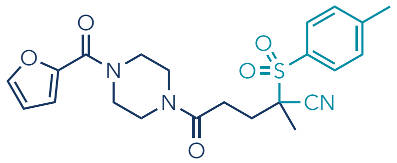

Following general procedure G described above, the product was obtained as a white solid (28.6 mg, 64.5 µmol, 32%). Purification was achieved by Biotage® Sfär KP-Amino 11g column chromatography (PE/EtOAc 35% → 65%).

^{1}H NMR (400 MHz, $CDCl_3$) δ = 7.94 – 7.85 (m, 2H), 7.50 (dd, *J*=1.8, 0.9, 1H), 7.47 – 7.39 (m, 2H), 7.07 (dd, *J*=3.5, 0.8, 1H), 6.50 (dd, *J*=3.5, 1.7, 1H), 3.83 (s, 4H), 3.70 (dd, *J*=7.3, 4.0, 2H), 3.62 – 3.51 (m, 2H), 2.77 (ddd, *J*=16.1, 10.7, 5.5, 1H), 2.67 (ddd, *J*=16.0, 10.1, 5.5, 1H), 2.54 – 2.45 (m, 4H), 2.39 (ddd, *J*=14.7, 10.7, 5.6, 1H), 1.63 (s, 3H).

^{13}C NMR (101 MHz, $CDCl_3$) δ = 169.1, 159.3, 147.7, 146.9, 144.1, 130.9, 130.3, 117.4, 117.4, 111.7, 61.3, 45.4, 42.0, 29.4, 29.0, 21.9, 20.6.

HRMS (ESI): Calcd. for $C_{22}H_{25}N_3O_5S$ $[M+H]^+$: 444.1588, Found: 444.1594.

4-(4-Methoxyphenyl)-2-methyl-2-tosylbutanenitrile (3o).

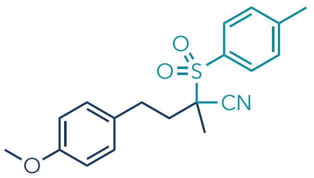

Following general procedure G described above, the product was obtained as a white solid (47.6 mg, 138 µmol, 69%). Purification was achieved by silica column chromatography (PE/Acetone 0% → 10%).

^{1}H NMR (400 MHz, $CDCl_3$) δ = 7.93 – 7.84 (m, 2H), 7.45 – 7.37 (m, 2H), 7.14 – 7.05 (m, 2H), 6.88 – 6.80 (m, 2H), 3.79 (s, 3H), 2.86 – 2.72 (m, 2H), 2.49 (s, 3H), 2.34 (ddd, *J*=13.7, 10.7, 6.2, 1H), 2.15 (ddd, *J*=13.7, 11.0, 6.6, 1H), 1.69 (s, 3H).

^{13}C NMR (101 MHz, $CDCl_3$) δ = 158.5, 146.7, 131.3, 130.9, 130.8, 130.1, 129.4, 117.5, 114.2, 62.1, 55.4, 35.0, 30.4, 21.9, 19.0.

HRMS (ESI): Calcd. for $C_{19}H_{21}NO_3S$ $[M+H]^+$: 344.1315, Found: 344.1321.

((5*R*,5a*S*,8a*S*,8b*R*)-2,2,7,7-tetramethyltetrahydro-5*H*-bis([1,3]dioxolo)[4,5-*b*:4',5'-*d*]pyran-5-yl)methyl 4-cyano-4-tosylpentanoate (3p).

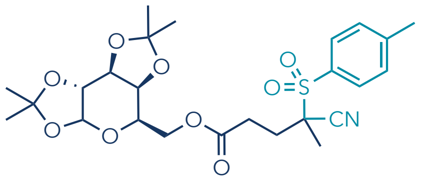

Following general procedure G described above, the product was obtained as a colorless sticky oil (93.2 mg, 178 µmol, 89%). Purification was achieved by silica column chromatography (PE/Acetone 0% → 20%).

^{1}H NMR (400 MHz, $CDCl_3$) δ = 7.98 – 7.69 (m, 2H), 7.50 – 7.29 (m, 2H), 5.51 (dd, *J*=5.0, 2.8, 1H), 4.60 (dd, *J*=7.8, 2.4, 1H), 4.31 (ddd, *J*=5.0, 2.6, 1.7, 1H), 4.29 – 4.15 (m, 3H), 4.00 (ddt, *J*=7.3, 5.4, 1.9, 1H), 2.73 – 2.51 (m, 2H), 2.47 (s, 3H), 2.46 – 2.35 (m, 1H), 2.24 (dddd, *J*=14.2, 10.0, 6.2, 1.7, 1H), 1.63 (d, *J*=3.3, 3H), 1.50 (s, 3H), 1.43 (s, 3H), 1.32 (d, *J*=2.5, 6H), 0.89 – 0.79 (m, 1H).

^{13}C NMR (101 MHz, $CDCl_3$) δ = 171.2, 171.2, 146.9, 130.9, 130.9, 130.6, 130.6, 130.2, 117.1, 109.8, 109.8, 108.9, 108.9, 96.3, 71.1, 70.8, 70.5, 65.9, 65.8, 64.1, 64.1, 61.3, 61.2, 29.8, 29.8, 28.7, 28.7, 26.1, 26.0, 25.0, 24.6, 21.9, 19.1, 19.0.

HRMS (ESI): Calcd. for $C_{25}H_{33}NO_9S$ $[M+H]^+$: 524.1949, Found: 524.1953.

2-Methyl-2-tosyl-4-(2,6,6-trimethylcyclohex-1-en-1-yl)butanenitrile (3q).

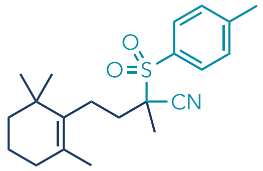

Following general procedure G described above, the product was obtained as a white solid (50.2 mg, 140 µmol, 70%). Purification was achieved by silica column chromatography (PE/Acetone 0% → 20%).

^{1}H NMR (400 MHz, $CDCl_3$) δ = 7.96 – 7.85 (m, 2H), 7.47 – 7.39 (m, 2H), 2.50 (s, 3H), 2.32 – 2.10 (m, 3H), 2.03 – 1.88 (m, 3H), 1.67 (s, 3H), 1.62 – 1.54 (m, 5H), 1.46 – 1.41 (m, 2H), 1.00 (s, 3H), 0.97 (s, 3H), 0.95 – 0.83 (m, 1H).
^{13}C NMR (101 MHz, $CDCl_3$) δ = 146.5, 134.6, 131.0, 130.9, 130.0, 129.5, 117.6, 62.2, 39.7, 35.1, 32.9, 32.5, 28.7, 28.5, 23.8, 21.9, 19.8, 19.4, 18.9.
HRMS (ESI): Calcd. for $C_{21}H_{29}NO_2S$ $[M+H]^+$: 360.1992, Found: 360.1998.

6-(3,7-Dimethyl-2,6-dioxo-2,3,6,7-tetrahydro-1*H*-purin-1-yl)-2-methyl-2-tosylhexanenitrile (3r).

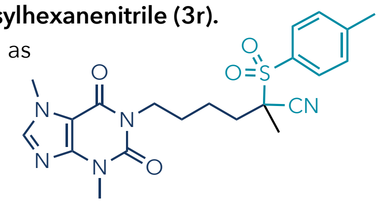

Following general procedure G described above, the product was obtained as a white solid (28.6 mg, 64.5 µmol, 32%). Purification was achieved by silica column chromatography (PE/EtOAc 0% → 65%).

^{1}H NMR (400 MHz, $CDCl_3$) δ = 7.96 – 7.68 (m, 2H), 7.54 – 7.46 (m, 1H), 7.46 – 7.28 (m, 2H), 4.00 (ddd, *J*=8.2, 6.5, 3.3, 2H), 3.97 – 3.93 (m, 3H), 3.55 (d, *J*=1.3, 3H), 2.47 (s, 3H), 2.12 – 1.98 (m, 1H), 1.99 – 1.86 (m, 1H), 1.78 – 1.52 (m, 7H).
^{13}C NMR (101 MHz, $CDCl_3$) δ = 155.3, 151.5, 148.9, 146.6, 141.6, 130.9, 130.8, 130.1, 117.6, 107.7, 62.2, 40.7, 33.7, 32.7, 29.8, 27.8, 22.4, 21.9, 18.6.
HRMS (ESI): Calcd. for $C_{21}H_{25}N_5O_4S$ $[M+H]^+$: 444.1700, Found: 444.1709.

6.2 Aryl sulfonyl cyanide scope

1-((4-Methoxyphenyl)sulfonyl)cyclohexane-1-carbonitrile (3s).

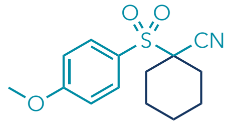

Following general procedure G described above, the product was obtained as a colorless oil (34.9 mg, 125 µmol, 64%). Purification was achieved by silica column chromatography (PE/Acetone 0% → 10%).

^{1}H NMR (400 MHz, $CDCl_3$) δ = 8.05 – 7.71 (m, 2H), 7.12 – 6.92 (m, 2H), 3.90 (s, 3H), 2.19 – 2.02 (m, 2H), 1.98 – 1.83 (m, 4H), 1.81 – 1.70 (m, 1H), 1.61 – 1.45 (m, 2H), 1.29 – 1.17 (m, 1H).
^{13}C NMR (101 MHz, $CDCl_3$) δ = 164.9, 133.0, 125.0, 117.2, 114.6, 64.6, 55.9, 29.3, 24.4, 22.6.
HRMS (ESI): Calcd. for $C_{14}H_{17}NO_3S$ $[M+H]^+$: 280.1002, Found: 280.1005.

1-(Phenylsulfonyl)cyclohexane-1-carbonitrile (3t).

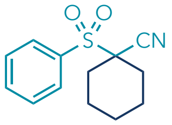

Following general procedure G described above, the product was obtained as a white solid (47.3 mg, 190 µmol, 95%). Purification was achieved by silica column chromatography (PE/Acetone 0% → 10%).

^{1}H NMR (400 MHz, $CDCl_3$) δ = 8.06 – 7.88 (m, 2H), 7.83 – 7.67 (m, 1H), 7.69 – 7.50 (m, 2H), 2.18 – 2.04 (m, 2H), 2.02 – 1.82 (m, 4H), 1.81 – 1.70 (m, 1H), 1.61 – 1.48 (m, 2H), 1.30 – 1.17 (m, 1H).
^{13}C NMR (101 MHz, $CDCl_3$) δ = 135.1, 133.9, 130.9, 129.4, 117.0, 64.5, 29.2, 24.3, 22.6.
HRMS (ESI): Calcd. for $C_{13}H_{15}NO_2S$ $[M+H]^+$: 250.0896, Found: 250.0904.

1-((4-Fluorophenyl)sulfonyl)cyclohexane-1-carbonitrile (3u).

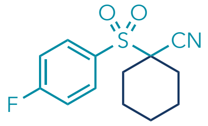

Following general procedure G described above, the product was obtained as a white solid (43.1 mg, 161 µmol, 81%). Purification was achieved by silica column chromatography (PE/Acetone 0% → 10%).

^{1}H NMR (400 MHz, $CDCl_3$) δ = 8.10 – 7.90 (m, 2H), 7.45 – 7.26 (m, 2H), 2.19 – 2.04 (m, 2H), 2.04 – 1.83 (m, 4H), 1.82 – 1.73 (m, 1H), 1.61 – 1.46 (m, 2H), 1.31 – 1.17 (m, 1H).
^{13}C NMR (101 MHz, $CDCl_3$) δ = 166.8 (d, *J*=258.8), 133.8 (d, *J*=10.0), 129.8 (d, *J*=3.5), 116.9, 116.9 (d, *J*=23.0), 64.6, 29.2, 24.3, 22.6.
^{19}F NMR (376 MHz, $CDCl_3$) δ = -101.35.
HRMS (ESI): Calcd. for $C_{13}H_{14}FNO_2S$ $[M+H]^+$: 268.0802, Found: 268.0805.

1-((4-Bromophenyl)sulfonyl)cyclohexane-1-carbonitrile (3v).

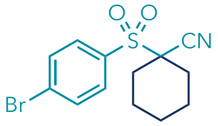

Following general procedure G described above, the product was obtained as a colorless oil (29.4 mg, 89.5 µmol, 45%). Purification was achieved by silica column chromatography (PE/Acetone 0% → 10%).

^{1}H NMR (400 MHz, $CDCl_3$) δ = 8.00 – 7.79 (m, 2H), 7.79 – 7.67 (m, 2H), 2.21 – 2.02 (m, 2H), 2.04 – 1.83 (m, 4H), 1.78 (dddd, *J*=16.2, 5.3, 2.7, 1.3, 1H), 1.68 – 1.47 (m, 2H), 1.33 – 1.16 (m, 1H).
^{13}C NMR (101 MHz, $CDCl_3$) δ = 132.8, 132.8, 132.3, 131.0, 116.8, 64.6, 29.1, 24.3, 22.6.
HRMS (ESI): Calcd. for $C_{13}H_{14}BrNO_2S$ $[M+H]^+$: 328.0001, Found: 328.0002.

1-((4-(4-Bromophenoxy)phenyl)sulfonyl)cyclohexane-1-carbonitrile (3w).

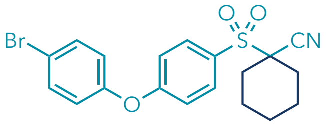

Following general procedure G described above, the product was obtained as a colorless oil (48.4 mg, 115 µmol, 58%). Purification was achieved by silica column chromatography (PE/Acetone 0% → 20%).

^{1}H NMR (400 MHz, $CDCl_3$) δ = 8.00 – 7.69 (m, 2H), 7.66 – 7.30 (m, 2H), 7.19 – 7.02 (m, 2H), 7.05 – 6.91 (m, 2H), 2.22 – 2.04 (m, 2H), 2.01 – 1.85 (m, 4H), 1.81 – 1.74 (m, 1H), 1.62 – 1.52 (m, 2H), 1.30 – 1.18 (m, 1H).
^{13}C NMR (101 MHz, $CDCl_3$) δ = 163.3, 153.7, 133.5, 133.3, 127.3, 122.5, 118.5, 117.5, 117.1, 64.6, 29.2, 24.4, 22.6.

1-((5-Isopropyl-2-methoxyphenyl)sulfonyl)cyclohexane-1-carbonitrile (3x).

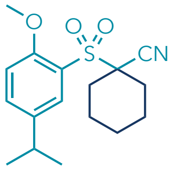

Following general procedure G described above, the product was obtained as a colorless oil (40.0 mg, 124 µmol, 62%). Purification was achieved by silica column chromatography (PE/Acetone 0% → 15%).

^{1}H NMR (400 MHz, $CDCl_3$) δ = 7.86 – 7.67 (m, 1H), 7.50 (ddd, *J*=8.5, 2.4, 0.6, 1H), 7.00 (d, *J*=8.6, 1H), 3.91 (s, 3H), 2.92 (hept, *J*=6.9, 1H), 2.20 – 2.08 (m, 2H), 2.02 – 1.83 (m, 4H), 1.79 – 1.72 (m, 1H), 1.65 – 1.52 (m, 2H), 1.27 – 1.18 (m, 7H).
^{13}C NMR (101 MHz, $CDCl_3$) δ = 157.2, 141.5, 135.1, 131.8, 121.9, 117.0, 113.0, 65.4, 56.2, 33.2, 29.5, 24.4, 23.9, 22.6.
HRMS (ESI): Calcd. for $C_{17}H_{23}NO_3S$ $[M+H]^+$: 322.1471, Found: 322.1475.

5-((1-Cyanocyclohexyl)sulfonyl)-2-methoxyphenyl acetate (3y).

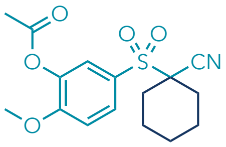

Following general procedure G described above, the product was obtained as a white solid (58.2 mg, 173 µmol, 86%). Purification was achieved by silica column chromatography (PE/Acetone 0% → 20%).

^{1}H NMR (400 MHz, $CDCl_3$) δ = 7.87 (dd, *J*=8.8, 2.3, 1H), 7.61 (d, *J*=2.3, 1H), 7.13 (d, *J*=8.8, 1H), 3.92 (s, 3H), 2.32 (s, 3H), 2.17 – 2.05 (m, 2H), 1.97 – 1.82 (m, 4H), 1.79 – 1.70 (m, 1H), 1.61 – 1.46 (m, 2H), 1.27 – 1.15 (m, 1H).
^{13}C NMR (101 MHz, $CDCl_3$) δ = 168.2, 156.9, 139.8, 130.7, 125.5, 125.0, 117.0, 112.4, 64.7, 56.5, 29.2, 24.3, 22.6, 20.6.
HRMS (ESI): Calcd. for $C_{16}H_{19}NO_5S$ $[M+H]^+$: 338.1057, Found: 338.1060.

1-((3-Chloro-4-methoxyphenyl)sulfonyl)cyclohexane-1-carbonitrile (3z).

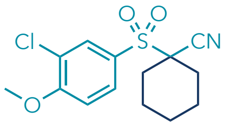

Following general procedure G described above, the product was obtained as a colorless oil (54.4 mg, 173 µmol, 87%). Purification was achieved by silica column chromatography (PE/Acetone 0% → 15%).

^{1}H NMR (400 MHz, $CDCl_3$) δ = 7.92 (d, *J*=2.3, 1H), 7.88 (dd, *J*=8.7, 2.3, 1H), 7.10 (d, *J*=8.7, 1H), 4.00 (s, 3H), 2.18 – 2.04 (m, 2H), 2.01 – 1.80 (m, 4H), 1.77 (dqd, *J*=13.5, 2.6, 1.4, 1H), 1.60 – 1.47 (m, 2H), 1.29 – 1.18 (m, 1H).
^{13}C NMR (101 MHz, $CDCl_3$) δ = 160.4, 132.3, 131.5, 125.7, 123.8, 116.9, 111.9, 64.7, 56.8, 29.2, 24.3, 22.6.
HRMS (ESI): Calcd. for $C_{14}H_{16}ClNO_3S$ $[M+H]^+$: 314.0615, Found: 314.0615.

1-(Benzo[*d*][1,3]dioxol-5-ylsulfonyl)cyclohexane-1-carbonitrile (3aa).

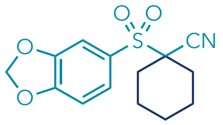

Following general procedure G described above, the product was obtained as a white solid (34.3 mg, 117 µmol, 58%). Purification was achieved by silica column chromatography (PE/Acetone 0% → 15%).

^{1}H NMR (400 MHz, $CDCl_3$) δ = 7.55 (dd, *J*=8.3, 1.9, 1H), 7.36 (d, *J*=1.9, 1H), 6.97 (d, *J*=8.3, 1H), 6.13 (s, 2H), 2.21 – 2.05 (m, 2H), 1.98 – 1.82 (m, 4H), 1.81 – 1.73 (m, 1H), 1.60 – 1.46 (m, 2H), 1.23 (qt, *J*=13.3, 3.8, 1H).
^{13}C NMR (101 MHz, $CDCl_3$) δ = 153.6, 148.5, 127.3, 126.7, 117.1, 110.4, 108.7, 102.9, 64.7, 29.3, 24.4, 22.6.
HRMS (ESI): Calcd. for $C_{14}H_{15}NO_4S$ [M+H]$^+$: 294.0795, Found: 294.0797.

6.3 Aliphatic sulfonyl cyanide scope

4-(4-Methoxyphenyl)-2-methyl-2-(methylsulfonyl)butanenitrile (3ab).
Following general procedure G described above, the product was obtained as a colorless oil (45.3 mg, 169 µmol, 85%). Purification was achieved by silica column chromatography (PE/Acetone 0% → 20%).

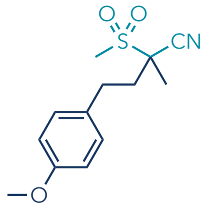

^{1}H NMR (400 MHz, $CDCl_3$) δ = 7.19 – 7.05 (m, 2H), 6.91 – 6.79 (m, 2H), 3.79 (s, 3H), 3.13 (s, 3H), 2.94 – 2.77 (m, 2H), 2.40 (ddd, *J*=13.7, 11.2, 5.4, 1H), 2.15 (ddd, *J*=13.8, 11.6, 6.0, 1H), 1.79 (s, 3H).
^{13}C NMR (101 MHz, $CDCl_3$) δ = 158.6, 131.0, 129.5, 117.4, 114.3, 60.8, 55.4, 36.5, 34.7, 30.3, 18.4.
HRMS (ESI): Calcd. for $C_{13}H_{17}NO_3S$ $[M+H]^+$: 268.1002, Found: 268.1005.

2-(Ethylsulfonyl)-4-(4-methoxyphenyl)-2-methylbutanenitrile (3ac).
Following general procedure G described above, the product was obtained as a colorless oil (40.0 mg, 142 µmol, 71%). Purification was achieved by silica column chromatography (PE/Acetone 0% → 20%).

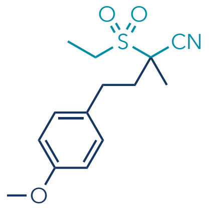

^{1}H NMR (400 MHz, $CDCl_3$) δ = 7.17 – 7.10 (m, 2H), 6.90 – 6.81 (m, 2H), 3.79 (s, 3H), 3.40 – 3.19 (m, 2H), 2.95 – 2.75 (m, 2H), 2.41 (ddd, *J*=13.8, 11.3, 5.4, 1H), 2.15 (ddd, *J*=13.8, 11.7, 6.0, 1H), 1.79 (s, 3H), 1.53 (t, *J*=7.5, 3H).
^{13}C NMR (101 MHz, $CDCl_3$) δ = 158.6, 131.1, 129.5, 117.5, 114.3, 60.3, 55.4, 43.6, 34.9, 30.2, 18.6, 5.7.
HRMS (ESI): Calcd. for $C_{14}H_{19}NO_3S$ $[M+H]^+$: 282.1158, Found: 282.1162.

2-(Isopropylsulfonyl)-4-(4-methoxyphenyl)-2-methylbutanenitrile (3ad).
Following general procedure G described above, the product was obtained as a colorless oil (40.0 mg, 142 µmol, 71%). Purification was achieved by silica column chromatography (PE/Acetone 0% → 20%).

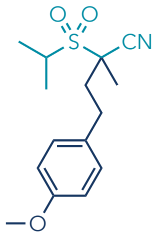

^{1}H NMR (400 MHz, $CDCl_3$) δ = 7.20 – 7.04 (m, 2H), 6.93 – 6.75 (m, 2H), 3.79 (s, 3H), 3.65 (hept, *J*=6.9, 1H), 2.97 – 2.72 (m, 2H), 2.41 (ddd, *J*=13.7, 11.7, 5.0, 1H), 2.15 (ddd, *J*=13.8, 12.0, 5.7, 1H), 1.79 (s, 3H), 1.55 (d, *J*=6.9, 3H), 1.55 (d, *J*=6.9, 3H).
^{13}C NMR (101 MHz, $CDCl_3$) δ = 158.6, 131.2, 129.5, 117.9, 114.3, 59.7, 55.4, 53.4, 35.6, 30.1, 19.3, 17.1, 17.0.
HRMS (ESI): Calcd. for $C_{15}H_{21}NO_3S$ $[M+H]^+$: 296.1315, Found: 296.1320.

4-(Methylsulfonyl)-1-tosylpiperidine-4-carbonitrile (3ae).
Following general procedure G described above, the product was obtained as a white solid (53.1 mg, 155 µmol, 78%). Purification was achieved by silica column chromatography (PE/Acetone 10% → 30%).

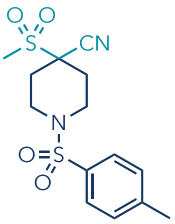

^{1}H NMR (400 MHz, $CDCl_3$) δ = 7.70 – 7.54 (m, 2H), 7.45 – 7.29 (m, 2H), 4.13 – 3.89 (m, 2H), 3.08 (s, 3H), 2.57 (ddd, *J*=12.8, 10.9, 4.0, 2H), 2.45 (s, 3H), 2.36 – 2.20 (m, 4H).
^{13}C NMR (101 MHz, $CDCl_3$) δ = 143.5, 131.7, 129.1, 126.5, 114.5, 59.5, 41.4, 35.3, 27.0, 20.6.
HRMS (ESI): Calcd. for $C_{14}H_{18}N_2O_4S_2$ $[M+H]^+$: 343.0781, Found: 343.0788.

2-((Cyclopropylmethyl)sulfonyl)-4-(4-methoxyphenyl)-2-methylbutanenitrile (3af).

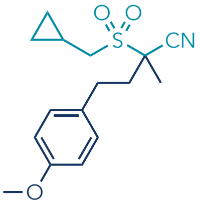

Following general procedure G described above, the product was obtained as a colorless oil (9.4 mg, 31 μmol, 15%). Purification was achieved by silica column chromatography (PE/Acetone 0% → 20%).

^{1}H NMR (400 MHz, $CDCl_3$) δ = 7.19 – 7.10 (m, 2H), 6.90 – 6.82 (m, 2H), 3.80 (s, 3H), 3.22 (d, *J*=7.2, 2H), 2.95 – 2.76 (m, 2H), 2.41 (ddd, *J*=13.8, 11.4, 5.4, 1H), 2.14 (ddd, *J*=13.8, 11.7, 6.0, 1H), 1.79 (s, 3H), 1.35 – 1.25 (m, 1H), 0.92 – 0.71 (m, 2H), 0.61 – 0.40 (m, 2H).
^{13}C NMR (101 MHz, $CDCl_3$) δ = 158.6, 131.2, 129.5, 117.6, 114.4, 60.5, 55.4, 54.9, 35.0, 30.3, 18.5, 5.3, 3.6.
HRMS (ESI): Calcd. for $C_{16}H_{21}NO_3S$ $[M+H]^+$: 308.1315, Found: 308.1317.

Diethyl (3-((4-cyano-1-tosylpiperidin-4-yl)sulfonyl)propyl)phosphonate (3ag).

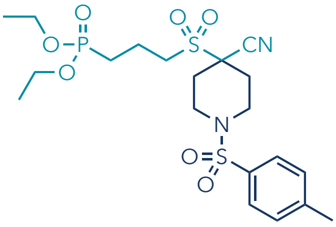

Following general procedure G described above, the product was obtained as a white solid (19.0 mg, 37.5 μmol, 19%). Purification was achieved by silica column chromatography (PE/Acetone 10% → 30%).

^{1}H NMR (400 MHz, $CDCl_3$) δ = 7.70 – 7.59 (m, 2H), 7.42 – 7.30 (m, 2H), 4.43 – 3.71 (m, 6H), 3.62 – 3.20 (m, 2H), 2.66 – 2.48 (m, 2H), 2.45 (s, 3H), 2.38 – 2.17 (m, 5H), 1.94 (dt, *J*=18.2, 7.3, 3H), 1.32 (t, *J*=7.1, 6H).
^{13}C NMR (101 MHz, $CDCl_3$) δ = 144.64, 132.87, 130.26, 127.64, 115.59, 62.14 (d, *J*=6.5), 60.61, 48.58 (d, *J*=10.8), 42.48, 28.17, 24.16 (d, *J*=143.1), 21.72, 16.59 (d, *J*=6.1), 15.20 (d, *J*=5.2).
HRMS (ESI): Calcd. for $C_{20}H_{31}N_2O_7PS_2$ $[M+H]^+$: 507.1383, Found: 507.1391.

2-((3-Chloropropyl)sulfonyl)-4-(4-methoxyphenyl)-2-methylbutanenitrile (3ah).

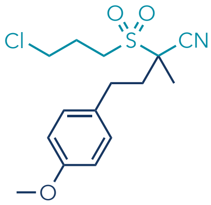

Following general procedure G described above, the product was obtained as a colorless oil (27.9 mg, 84.6 μmol, 42%). Purification was achieved by silica column chromatography (PE/Acetone 0% → 20%).

^{1}H NMR (400 MHz, $CDCl_3$) δ = 7.18 – 7.11 (m, 2H), 6.89 – 6.83 (m, 2H), 3.80 (s, 3H), 3.74 (t, *J*=6.1, 2H), 3.56 – 3.35 (m, 2H), 2.98 – 2.76 (m, 2H), 2.63 – 2.32 (m, 3H), 2.16 (ddd, *J*=13.7, 11.7, 5.9, 1H), 1.81 (s, 3H).
^{13}C NMR (101 MHz, $CDCl_3$) δ = 158.7, 131.0, 129.5, 117.3, 114.4, 61.0, 55.5, 46.3, 42.9, 34.8, 30.3, 24.6, 18.5.
HRMS (ESI): Calcd. for $C_{15}H_{20}ClNO_3S$ $[M+H]^+$: 330.0925, Found: 330.0929.

Ethyl 2-((2-cyano-4-(4-methoxyphenyl)butan-2-yl)sulfonyl)acetate (3ai).

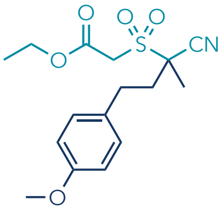

Following general procedure G described above, the product was obtained as a colorless oil (12.3 mg, 36.2 μmol, 18%). Purification was achieved by silica column chromatography (PE/Acetone 0% → 20%).

^{1}H NMR (400 MHz, $CDCl_3$) δ = 7.17 – 7.10 (m, 2H), 6.89 – 6.82 (m, 2H), 4.32 (q, *J*=7.2, 2H), 4.26 (s, 2H), 3.80 (s, 3H), 2.86 (s, 2H), 2.50 (ddd, *J*=13.8, 11.5, 5.2, 1H), 2.16 (ddd, *J*=13.8, 11.7, 5.7, 1H), 1.84 (s, 3H), 1.33 (t, *J*=7.2, 3H).
^{13}C NMR (101 MHz, $CDCl_3$) δ = 161.3, 158.7, 130.9, 129.5, 116.8, 114.4, 63.5, 62.7, 55.4, 55.2, 34.9, 30.3, 18.8, 14.0.
HRMS (ESI): Calcd. for $C_{16}H_{21}NO_5S$ $[M+H]^+$: 340.1213, Found: 340.1214.

2-(Benzylsulfonyl)-4-(4-methoxyphenyl)-2-methylbutanenitrile (3aj).

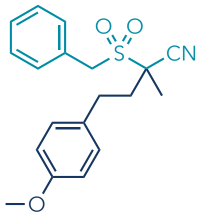

Following general procedure G described above, the product was obtained as a colorless oil (45.4 mg, 132 μmol, 66%). Purification was achieved by silica column chromatography (PE/Acetone 0% → 20%).

^{1}H NMR (400 MHz, $CDCl_3$) δ = 7.54 – 7.45 (m, 2H), 7.46 – 7.40 (m, 3H), 7.14 – 7.07 (m, 2H), 6.88 – 6.83 (m, 2H), 4.54 (s, 2H), 3.79 (s, 3H), 2.82 (qt, *J*=13.6, 7.1, 2H), 2.37 (ddd, *J*=13.8, 11.4, 5.4, 1H), 2.13 (ddd, *J*=13.8, 11.6, 5.9, 1H), 1.74 (s, 3H).
^{13}C NMR (101 MHz, $CDCl_3$) δ = 158.6, 131.5, 131.1, 129.7, 129.5, 129.2, 125.4, 117.5, 114.3, 61.0, 55.7, 55.4, 35.2, 30.2, 18.8.
HRMS (ESI): Calcd. for $C_{19}H_{21}NO_3S$ $[M+H]^+$: 344.1315, Found: 344.1320.

Methyl 4-(((2-cyano-4-(4-methoxyphenyl)butan-2-yl)sulfonyl)methyl)benzoate (3ak).

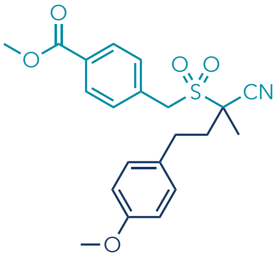

Following general procedure G described above, the product was obtained as a white solid (23.7 mg, 59.0 μmol, 30%). Purification was achieved by silica column chromatography (PE/Acetone 5% → 25%).

^{1}H NMR (400 MHz, $CDCl_3$) δ = 8.09 (d, *J*=8.5, 2H), 7.66 – 7.48 (m, 2H), 7.15 – 7.07 (m, 2H), 6.88 6.81 (m, 2H), 4.58 (s, 2H), 3.93 (s, 3H), 3.79 (s, 3H), 2.92 – 2.75 (m, 2H), 2.36 (ddd, *J*=13.8, 11.2, 5.5, 1H), 2.14 (ddd, *J*=13.8, 11.6, 6.1, 1H), 1.76 (s, 3H).
^{13}C NMR (101 MHz, $CDCl_3$) δ = 166.5, 158.7, 131.6, 131.4, 130.9, 130.4, 130.3, 129.5, 117.4, 114.4, 61.3, 55.4, 55.4, 52.5, 35.2, 30.3, 18.9.
HRMS (ESI): Calcd. for $C_{21}H_{23}NO_5S$ $[M+H]^+$: 402.1370, Found: 402.1374.

4-(((2-Cyano-4-(4-methoxyphenyl)butan-2-yl)sulfonyl)methyl)-3-fluorobenzonitrile (3al).

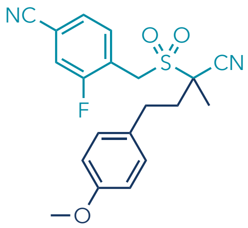

Following general procedure G described above, the product was obtained as a white solid (10.9 mg, 28.2 μmol, 14%). Purification was achieved by silica column chromatography (PE/Acetone 5% → 25%).

^{1}H NMR (400 MHz, $CDCl_3$) δ = 7.67 (t, *J*=7.5, 1H), 7.54 (dd, *J*=8.0, 1.7, 1H), 7.47 (dd, *J*=8.9, 1.6, 1H), 7.18 – 7.10 (m, 2H), 6.89 – 6.84 (m, 2H), 4.64 (s, 2H), 3.80 (s, 3H), 2.96 – 2.78 (m, 2H), 2.46 (ddd, *J*=13.7, 11.2, 5.4, 1H), 2.20 (ddd, *J*=13.7, 11.5, 5.9, 1H), 1.85 (s, 3H).
^{13}C NMR (101 MHz, $CDCl_3$) δ = 161.1 (d, *J*=254.0), 158.8, 134.4 (d, *J*=3.0), 130.7, 129.5, 128.7 (d, *J*=4.3), 119.9 (d, *J*=25.1), 119.2 (d, *J*=14.7), 116.9 (d, *J*=9.5), 115.5 (d, *J*=10.0), 114.4, 61.5, 55.5, 48.3 (d, *J*=2.2), 35.0, 30.3, 18.8.
^{19}F NMR (376 MHz, $CDCl_3$) δ = -112.6.
HRMS (ESI): Calcd. for $C_{20}H_{19}FN_2O_3S$ $[M+H]^+$: 387.1173, Found: 387.1181.

2-((3-(1,3-Dioxoisoindolin-2-yl)propyl)sulfonyl)-4-(4-methoxyphenyl)-2-methylbutanenitrile (3am).

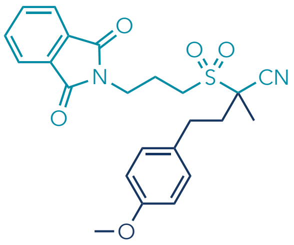

Following general procedure G described above, the product was obtained as a white solid (14.0 mg, 31.8 μmol, 16%). Purification was achieved by silica column chromatography (PE/Acetone 0% → 25%).

^{1}H NMR (400 MHz, $CDCl_3$) δ = 7.86 (dd, *J*=5.5, 3.0, 2H), 7.79 – 7.67 (m, 2H), 7.19 – 7.09 (m, 2H), 6.87 – 6.82 (m, 2H), 3.89 (t, *J*=6.6, 2H), 3.81 – 3.76 (m, 4H), 3.46 – 3.22 (m, 2H), 2.93 – 2.75 (m, 2H), 2.42 – 2.34 (m, 2H), 2.12 (ddd, *J*=13.7, 11.7, 5.8, 1H), 1.77 (s, 3H).

^{13}C NMR (101 MHz, $CDCl_3$) δ = 168.3, 158.6, 134.4, 132.0, 131.0, 129.5, 123.7, 117.3, 114.3, 60.8, 55.4, 46.8, 36.6, 34.7, 30.3, 21.2, 18.7.

HRMS (ESI): Calcd. for $C_{23}H_{24}N_2O_5S$ [M+H]$^+$: 441.1479, Found: 441.1477.

4-((3-(1,3-Dioxoisoindolin-2-yl)propyl)sulfonyl)-1-tosylpiperidine-4-carbonitrile (3an).

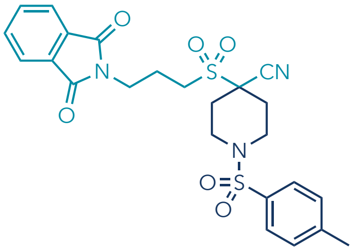

Following general procedure G described above, the product was obtained as a white solid (27.6 mg, 53.5 μmol, 27%). Purification was achieved by silica column chromatography (PE/Acetone 10% → 40%) followed by silica column chromatography (PE/EtOAc 25% → 50%).
Structure was confirmed by single-crystal X-ray diffraction.

^{1}H NMR (400 MHz, $CDCl_3$) δ = 7.85 (dd, *J*=5.5, 3.1, 2H), 7.73 (dd, *J*=5.5, 3.0, 2H), 7.68 – 7.57 (m, 2H), 7.35 (d, *J*=8.3, 2H), 4.14 – 3.91 (m, 2H), 3.87 (t, *J*=6.5, 2H), 3.39 – 3.19 (m, 2H), 2.62 – 2.48 (m, 2H), 2.45 (s, 3H), 2.42 – 2.16 (m, 6H).

^{13}C NMR (400 MHz, $CDCl_3$): δ = 168.4, 144.6, 134.4, 132.8, 131.9, 130.2, 127.6, 123.7, 115.5, 60.6, 46.9, 42.5, 36.5, 28.2, 21.7, 20.9.

HRMS (ESI): Calcd. for $C_{24}H_{25}N_3O_6S_2$ [M+H]$^+$: 516.1258, Found: 516.1264.

6.4 Sulfinyl cyanide scope

1-(Phenylsulfonyl)cyclohexane-1-carbonitrile (5a).

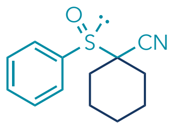

Following general procedure G described above, the product was obtained as a white solid (24.7 mg, 106 µmol, 53%). Purification was achieved by silica column chromatography (PE/Acetone 0% → 10%).
Structure was confirmed by single-crystal X-ray diffraction.

^{1}H NMR (400 MHz, $CDCl_3$) δ = 7.86 – 7.65 (m, 2H), 7.68 – 7.45 (m, 3H), 1.98 – 1.80 (m, 5H), 1.80 – 1.72 (m, 2H), 1.67 – 1.48 (m, 2H), 1.30 – 1.16 (m, 1H).
^{13}C NMR (101 MHz, $CDCl_3$) δ = 138.2, 132.7, 129.1, 126.1, 117.6, 61.6, 30.6, 27.3, 24.5, 23.2, 22.9.
HRMS (ESI): Calcd. for $C_{13}H_{15}NOS$ $[M+H]^+$: 234.0947, Found: 234.0947.

1-((4-Fluorophenyl)sulfinyl)cyclohexane-1-carbonitrile (5b).

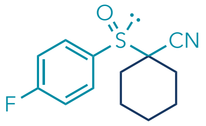

Following general procedure G described above, the product was obtained as a white solid (33.2 mg, 132 µmol, 66%). Purification was achieved by silica column chromatography (PE/Acetone 0% → 10%).

^{1}H NMR (400 MHz, $CDCl_3$) δ = 7.80 – 7.65 (m, 2H), 7.34 – 7.21 (m, 2H), 2.17 – 1.67 (m, 7H), 1.67 – 1.46 (m, 2H), 1.23 (qt, *J*=12.8, 3.7, 1H).
^{13}C NMR (101 MHz, $CDCl_3$) δ = 165.5 (d, *J*=254.0), 133.7 (d, *J*=3.0), 128.5 (d, *J*=9.1), 117.5, 116.6 (d, *J*=22.5), 61.8, 30.5, 27.3, 24.5, 23.1, 22.9.
^{19}F NMR (376 MHz, $CDCl_3$) δ = -106.3.
HRMS (ESI): Calcd. for $C_{13}H_{14}FNOS$ $[M+H]^+$: 252.0853, Found: 252.0855.

1-((4-Bromophenyl)sulfinyl)cyclohexane-1-carbonitrile (5c).

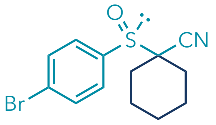

Following general procedure G described above, the product was obtained as a white solid (13.2 mg, 42.2 µmol, 21%). Purification was achieved by silica column chromatography (PE/Acetone 0% → 10%).

^{1}H NMR (400 MHz, $CDCl_3$) δ = 7.72 (d, *J*=8.5, 2H), 7.62 (d, *J*=8.6, 2H), 2.00 – 1.71 (m, 6H), 1.73 – 1.43 (m, 3H), 1.25 (ttd, *J*=12.8, 7.5, 3.8, 1H).
^{13}C NMR (101 MHz, $CDCl_3$) δ = 137.4, 132.4, 127.7, 127.6, 117.5, 61.8, 30.6, 27.1, 24.5, 23.2, 22.9.

1-(Benzylsulfinyl)cyclohexane-1-carbonitrile (5d).

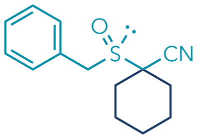

Following general procedure G described above, the product was obtained as a white solid (36.9 mg, 149 µmol, 75%). Purification was achieved by silica column chromatography (PE/Acetone 0% → 15%).

^{1}H NMR (400 MHz, $CDCl_3$) δ = 7.51 – 7.30 (m, 5H), 4.17 (d, *J*=12.8, 1H), 4.07 (d, *J*=12.8, 1H), 2.33 – 2.23 (m, 1H), 2.04 – 1.62 (m, 8H), 1.28 (qt, *J*=12.4, 3.8, 1H).
^{13}C NMR (101 MHz, $CDCl_3$) δ = 130.4, 130.0, 129.2, 128.8, 118.0, 58.9, 56.1, 30.7, 28.4, 24.6, 23.0, 22.8.
HRMS (ESI): Calcd. for $C_{14}H_{17}NOS$ $[M+H]^+$: 248.1104, Found: 248.1107.

2-(Benzylsulfinyl)-4-(4-methoxyphenyl)-2-methylbutanenitrile (5e).

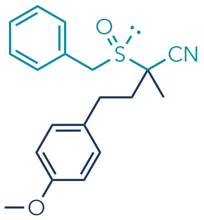

Following general procedure G described above, the product was obtained as a colorless oil (47.6 mg, 145 µmol, 73%, d.r. = 1:1). Purification was achieved by silica column chromatography (PE/Acetone 0% → 20%).

^{1}H NMR (400 MHz, $CDCl_3$) δ = 7.52 – 7.29 (m, 5H), 7.20 – 7.05 (m, 2H), 6.91 – 6.79 (m, 2H), 4.29 – 4.16 (m, 1H), 4.12 (d, *J*=12.8, 0.5H), 4.00 (d, *J*=12.8, 0.5H), 3.79 (s, 1.5H), 3.78 (s, 1.5H), 2.99 – 2.77 (m, 2H), 2.41 (ddd, *J*=13.8, 10.0, 6.9, 0.5H), 2.20 – 2.08 (m, 1H), 2.00 (ddd, *J*=14.0, 11.8, 5.6, 0.5H), 1.72 (s, 1.5H), 1.56 (s, 1.5H).

^{13}C NMR (101 MHz, $CDCl_3$) δ = 158.5, 131.5, 131.3, 130.4, 130.4, 129.9, 129.8, 129.4, 129.4, 129.2, 128.9, 118.5, 118.1, 114.2, 56.8, 56.5, 56.3, 55.9, 55.3, 37.1, 35.2, 30.4, 29.8, 18.9, 16.3.

HRMS (ESI): Calcd. for $C_{19}H_{21}NO_2S$ $[M+H]^+$: 328.1366, Found: 328.1374.

6.5 Matrix Library Scope

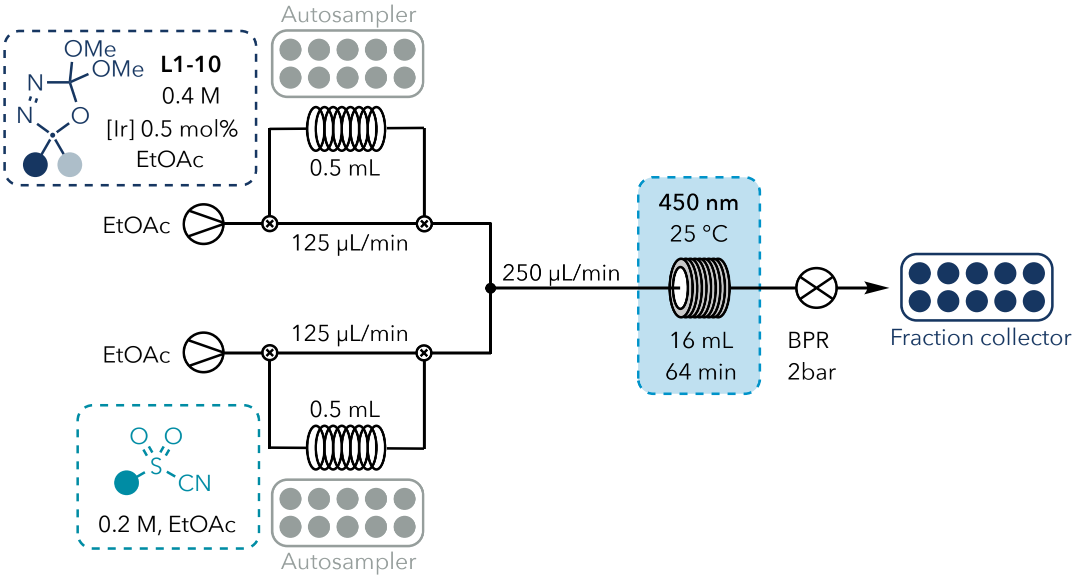

General Procedure H:

Two stock solutions were prepared in EtOAc: Solution A: Oxadiazoline (0.4 M, 2.0 eq.) and $Ir[dF(CF_3)ppy]_2(dtbpy)PF_6$ (0.5 mol%), Solution B: Tosyl cyanide (0.2 M, 1.0 eq.). These were pumped with 250 µL/min through a 16 mL coiled tube photoreactor at 25 °C and irradiated with 450 nm LEDs (40 W). The solvent was removed in vacuo, the mixture redissolved in DMSO and the products purified by preparative HPLC at 50 µmol scale.

5-Cyano-5-(methylsulfonyl)-*N*-phenylhexanamide (L13).

Following general procedure H described above, the product was obtained (5.20 mg, 17.7 µmol, 35%).

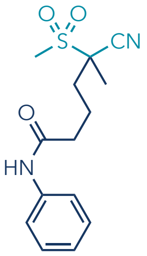

^{1}H NMR (500 MHz, DMSO) δ = 9.94 (s, 1H), 7.63 – 7.50 (m, 2H), 7.29 (dd, *J*=8.7, 7.4, 2H), 7.03 (tt, *J*=7.4, 1.2, 1H), 3.28 (s, 3H), 2.45 (td, *J*=6.8, 2.8, 2H), 2.23 – 2.11 (m, 1H), 1.92 – 1.74 (m, 3H), 1.72 (s, 3H).
^{13}C NMR (126 MHz, DMSO) δ = 170.3, 139.2, 128.7, 123.1, 119.1, 117.5, 60.8, 36.1, 35.3, 31.3, 20.4, 18.0.
HRMS (ESI): Calcd. for $C_{14}H_{18}N_2O_3S$ $[M+H]^+$: 295.1116, Found: 295.1136.

5-Cyano-*N*-phenyl-5-tosylhexanamide (L14).

Following general procedure H described above, the product was obtained (2.90 mg, 7.83 µmol, 16%).

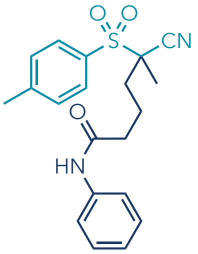

^{1}H NMR (500 MHz, DMSO) δ = 9.91 (s, 1H), 7.89 – 7.77 (m, 2H), 7.63 – 7.49 (m, 4H), 7.29 (dd, *J*=8.7, 7.4, 2H), 7.03 (tt, *J*=7.4, 1.2, 1H), 2.44 (s, 3H), 2.40 (t, *J*=7.0, 2H), 2.02 (td, *J*=12.9, 4.7, 1H), 1.91 – 1.78 (m, 2H), 1.77 – 1.65 (m, 1H), 1.61 (s, 3H).
^{13}C NMR (126 MHz, DMSO) δ = 170.2, 146.7, 139.1, 130.5, 130.3, 130.0, 128.7, 123.1, 119.1, 117.4, 62.0, 35.4, 31.8, 21.2, 20.5, 18.4.
HRMS (ESI): Calcd. for $C_{20}H_{22}N_2O_3S$ $[M+H]^+$: 371.1429, Found: 371.1432.

4-Hydroxy-4-(6-methoxypyridin-3-yl)-1-(methylsulfonyl)cyclohexane-1-carbonitrile (L15).

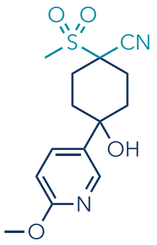

Following general procedure H described above, the product was obtained (8.90 mg, 28.7 µmol, 57%, d.r. > 20:1).

^{1}H NMR (500 MHz, DMSO) δ = 8.26 (dd, *J*=2.7, 0.9, 1H), 7.77 (dd, *J*=8.7, 2.6, 1H), 6.81 (dd, *J*=8.7, 0.8, 1H), 5.35 (s, 1H), 3.84 (s, 3H), 3.32 (s, 3H), 2.34 (td, *J*=13.1, 4.2, 2H), 2.21 (dp, *J*=13.1, 2.6, 2H), 1.97 (ddt, *J*=14.7, 5.1, 2.6, 2H), 1.90 (td, *J*=13.7, 3.9, 2H).
^{13}C NMR (126 MHz, DMSO) δ = 162.6, 143.2, 136.8, 136.3, 116.9, 109.8, 68.0, 61.8, 53.1, 36.2, 34.2, 23.9.
HRMS (ESI): Calcd. for $C_{14}H_{18}N_2O_4S$ $[M+H]^+$: 311.1065, Found: 311.1041.

4-(Methylsulfonyl)-1-(3-(trifluoromethyl)-[1,2,4]triazolo[4,3-*b*]pyridazin-6-yl)piperidine-4-carbonitrile (L29).

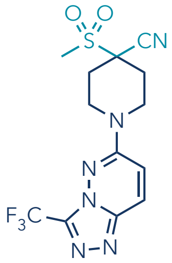

Following general procedure H described above, the product was obtained (12.2 mg, 32.6 µmol, 65%).

^{1}H NMR (500 MHz, DMSO) δ = 8.32 (d, *J*=10.4, 1H), 7.67 (d, *J*=10.4, 1H), 4.53 (dt, *J*=14.2, 2.4, 2H), 3.34 (s, 3H), 3.18 (ddd, *J*=14.9, 12.5, 2.6, 2H), 2.48 – 2.39 (m, 2H), 2.12 – 2.03 (m, 2H).
^{13}C NMR (126 MHz, DMSO) δ = 155.3, 144.3, 137.3 (q, *J*=40.0), 124.6, 118.6 (q, *J*=269.3), 117.0, 116.2, 61.2, 42.1, 36.2, 27.1.
^{19}F NMR (471 MHz, DMSO) δ = -63.29.
HRMS (ESI): Calcd. for $C_{13}H_{13}F_3N_6O_2S$ $[M+H]^+$: 375.0851, Found: 375.0834.

4-Hydroxy-4-(6-methoxypyridin-3-yl)-1-tosylcyclohexane-1-carbonitrile (L16).

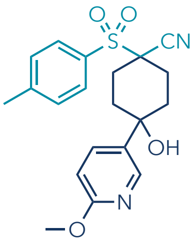

Following general procedure H described above, the product was obtained (8.90 mg, 28.7 µmol, 57%, d.r. > 20:1).

^{1}H NMR (500 MHz, DMSO) δ = 8.21 (dd, *J*=2.6, 0.8, 1H), 7.92 – 7.84 (m, 2H), 7.72 (dd, *J*=8.7, 2.6, 1H), 7.65 – 7.55 (m, 2H), 6.78 (dd, *J*=8.7, 0.8, 1H), 5.32 (s, 1H), 3.82 (s, 3H), 2.47 (s, 3H), 2.38 (td, *J*=13.0, 4.0, 2H), 2.01 – 1.87 (m, 4H), 1.81 (td, *J*=14.1, 4.0, 2H).
^{13}C NMR (126 MHz, DMSO) δ = 162.6, 146.8, 143.1, 136.7, 136.2, 130.4, 130.3, 130.0, 116.6, 109.8, 68.0, 63.3, 53.1, 34.3, 24.7, 21.3.
HRMS (ESI): Calcd. for $C_{14}H_{18}N_2O_4S$ $[M+H]^+$: 311.1065, Found: 311.1041.

Benzyl-4-cyano-3-ethyl-4-tosylpiperidine-1-carboxylate (L20).

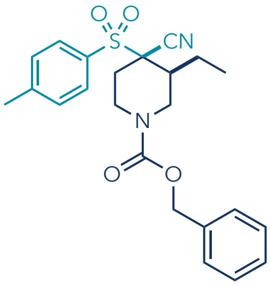

Following general procedure H described above, the product was obtained (11.6 mg, 27.2 μmol, 54%, d.r. > 20:1). Diastereomer was identified by 2D-NOESY NMR spectroscopy.

^{1}H NMR (500 MHz, DMSO) δ = 7.91 – 7.70 (m, 2H), 7.66 – 7.48 (m, 2H), 7.44 – 7.26 (m, 5H), 5.15 – 5.01 (m, 2H), 4.37 – 4.14 (m, 1H), 4.04 (ddt, *J*=14.1, 4.6, 2.7, 1H), 2.87 (s, 1H), 2.63 (d, *J*=44.6, 1H), 2.46 (s, 3H), 2.23 (dqd, *J*=15.1, 7.6, 2.8, 1H), 2.02 (tdd, *J*=10.7, 4.3, 2.7, 1H), 1.88 (td, *J*=12.8, 4.7, 1H), 1.60 (dt, *J*=13.4, 2.8, 1H), 1.31 (ddt, *J*=17.2, 14.0, 6.9, 1H), 0.96 (t, *J*=7.8, 3H).

^{13}C NMR (126 MHz, DMSO) δ = 153.9, 146.9, 136.6, 130.8, 130.5, 130.1, 128.4, 127.9, 127.5, 127.5, 115.0, 66.6, 66.4, 44.1, 40.4, 31.4, 21.6, 21.2, 11.1.

HRMS (ESI): Calcd. for $C_{23}H_{26}N_2O_4S$ $[M+H]^+$: 427.1691, Found: 427.1707.

3-((4-((6-Chloropyridin-3-yl)oxy)piperidin-1-yl)sulfonyl)-2-methyl-2-tosylpropanenitrile (L22).

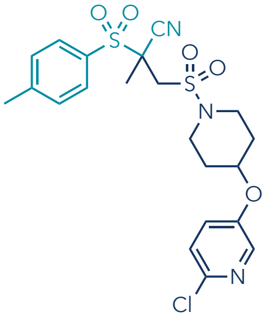

Following general procedure H described above, the product was obtained (0.8 mg, 1.64 μmol, 3% yield, 79% LCMS purity).

^{1}H NMR (500 MHz, DMSO) δ = 8.21 (d, *J*=2.8, 1H), 7.97 – 7.87 (m, 2H), 7.82 (dt, *J*=8.8, 2.6, 1H), 7.66 – 7.58 (m, 2H), 6.88 (d, *J*=8.9, 1H), 5.17 – 5.09 (m, 1H), 3.67 – 3.64 (m, 1H), 3.54 – 3.48 (m, 2H), 3.30 – 3.22 (m, 3H), 2.48 (s, 3H), 2.05 – 1.99 (m, 2H), 1.82 (s, 3H), 1.75 – 1.67 (m, 2H).

^{13}C NMR (126 MHz, DMSO) δ = 161.0, 147.5, 144.9, 139.3, 131.1, 130.5, 128.8, 123.4, 115.9, 112.9, 69.5, 58.8, 49.3, 42.8, 30.0, 21.3, 18.9.

HRMS (ESI): Calcd. for $C_{21}H_{24}ClN_3O_5S_2$ $[M+H]^+$: 498.0924, Found: 498.0947.

4-Tosyl-1-(3-(trifluoromethyl)-[1,2,4]triazolo[4,3-*b*]pyridazin-6-yl)piperidine-4-carbonitrile (L30).

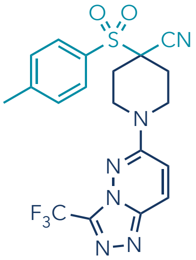

Following general procedure H described above, the product was obtained (10.5 mg, 23.3 μmol, 47%).

^{1}H NMR (500 MHz, DMSO) δ = 8.31 (d, *J*=10.2, 1H), 7.91 – 7.81 (m, 2H), 7.63 – 7.57 (m, 3H), 4.58 – 4.38 (m, 2H), 3.08 (ddd, *J*=14.7, 11.7, 3.4, 2H), 2.47 (s, 3H), 2.20 – 2.08 (m, 4H).

^{13}C NMR (126 MHz, DMSO) δ 155.3, 147.1, 144.3, 137.3 (q, *J* = 40.0 Hz), 130.5, 130.4, 129.5, 124.6, 118.6 (q, *J* = 269.6 Hz), 117.0, 116.1, 62.5, 42.2, 27.7, 21.3.

^{19}F NMR (471 MHz, DMSO) δ = -63.3.

HRMS (ESI): Calcd. for $C_{19}H_{17}F_3N_6O_2S$ $[M+H]^+$: 451.1164, Found: 451.1178.

7 X-ray crystal structures

1-Tosylcyclohexane-1-carbonitrile (3a).

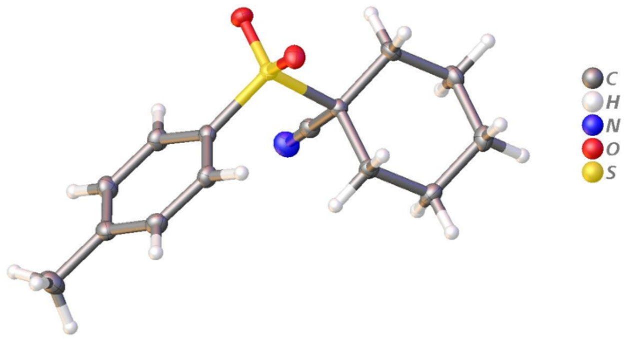

Sample preparation. In a 5 mL glass vial, purified compound **3a** was layered with 1 mL petroleum ether. Then CH_2Cl_2 was slowly added until the sample was completely dissolved. The vial was sealed with a plastic cap with a small hole to allow CH_2Cl_2 to evaporate. The sample was kept in the refrigerator until a crystal reached a sufficient size.

Crystal Data. Single clear colourless prism-shaped crystals of **3a** were used as supplied. A suitable crystal with dimensions 0.25 × 0.18 × 0.13 mm^3 was selected and mounted on a XtaLAB Synergy R, DW system, HyPix-Arc 150 diffractometer. The crystal was kept at a steady T = 123.00(10) K during data collection. The structure was solved with the ShelXT 2018/2[28] solution program using dual methods and by using Olex2 1.5-alpha as the graphical interface.[29] The model was refined with ShelXL 2018/3[30] using full matrix least squares minimisation on $\boldsymbol{F}^2$.

**Compound**	**3a**
Formula	$C_{14}H_{17}NO_2S$
$D_{calc.}$/ g cm^{-3}	1.336
μ/mm^{-1}	2.145
Formula Weight	263.34
Colour	clear colourless
Shape	prism-shaped
Size/mm^3	0.25×0.18×0.13
T/K	123.00(10)
Crystal System	monoclinic
Space Group	$P2_1/c$
a/Å	14.3813(3)
b/Å	5.99980(10)
c/Å	15.9106(3)
α/°	90
β/°	107.531(2)
γ/°	90
V/Å^3	1309.08(4)
Z	4
Z'	1
Wavelength/Å	1.54184
Radiation type	Cu K_α
Θ_{min}/°	3.223
Θ_{max}/°	75.110
Measured Refl's.	26166
Indep't Refl's	2665
Refl's I≥2 σ(I)	2641
R_{int}	0.0128
Parameters	164
Restraints	0
Largest Peak	0.295
Deepest Hole	-0.344
GooF	1.066
wR_2 (all data)	0.0751
wR_2	0.0750
R_1 (all data)	0.0284
R_1	0.0282

tert-Butyl 4-cyano-4-tosylpiperidine-1-carboxylate (3f).

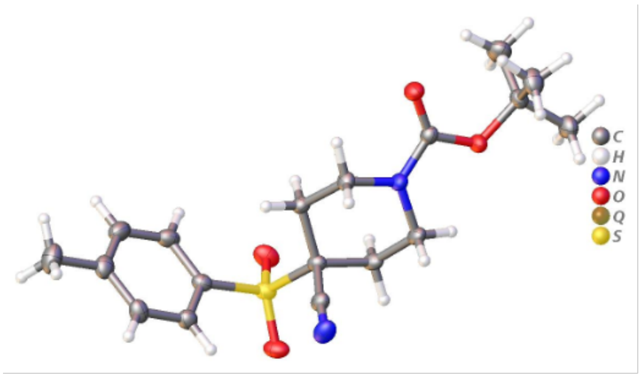

Sample preparation. In a 5 mL glass vial, the purified nitrile **3f** was layered with 1 mL petroleum ether. Then CH_2Cl_2 was slowly added until the sample was completely dissolved. The vial was sealed with a plastic cap with a small hole to allow CH_2Cl_2 to evaporate. The sample was kept in the refrigerator until a crystal reached a sufficient size.

Experimental. Single clear yellow plate-shaped crystals of **3f** were used as supplied. A suitable crystal with dimensions 0.22 × 0.04 × 0.02 mm^3 was selected and mounted on a XtaLAB Synergy R, DW system, HyPix-Arc 150 diffractometer. The crystal was kept at a steady T = 123.00(10) K during data collection. The structure was solved with the ShelXT 2018/2[28] solution program using dual methods and by using Olex2 1.5-alpha as the graphical interface.[29] The model was refined with ShelXL 2018/3[30] using full matrix least squares minimisation on $\boldsymbol{F^2}$.

**Compound**	**3f**
Formula	$C_{18}H_{24}N_2O_4S$
$D_{calc.}$/ g cm^{-3}	1.306
μ/mm^{-1}	1.762
Formula Weight	364.45
Colour	clear yellow
Shape	plate-shaped
Size/mm^3	0.22×0.04×0.02
T/K	123.00(10)
Crystal System	monoclinic
Space Group	$C2/c$
a/Å	29.1634(3)
b/Å	6.18070(10)
c/Å	21.1252(2)
α/°	90
β/°	103.1660(10)
γ/°	90
V/Å^3	3707.73(8)
Z	8
Z'	1
Wavelength/Å	1.54184
Radiation type	Cu K_α
Θ_{min}/°	3.112
Θ_{max}/°	75.350
Measured Refl's.	55011
Indep't Refl's	3812
Refl's I≥2 σ(I)	3568
R_{int}	0.0310
Parameters	230
Restraints	0
Largest Peak	0.426
Deepest Hole	-0.433
GooF	1.063
wR_2 (all data)	0.0894
wR_2	0.0882
R_1 (all data)	0.0345
R_1	0.0326

1,4-Ditosylpiperidine-4-carbonitrile (3g).

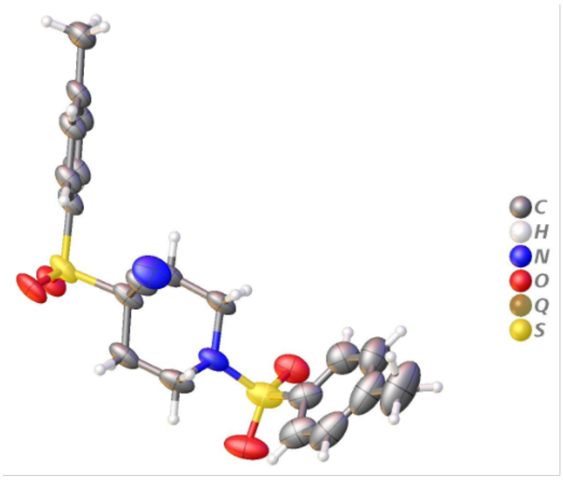

Compound	3g
Formula	$C_{20.5}H_{23}ClN_2O_4S_2$
$D_{calc.}$/ g cm^{-3}	1.392
μ/mm^{-1}	3.563
Formula Weight	461.007
Colour	colourless
Shape	needle-shaped
Size/mm^3	0.23×0.05×0.02
T/K	123.00(10)
Crystal System	monoclinic
Space Group	$P2_1/c$
a/Å	15.1621(6)
b/Å	6.0482(2)
c/Å	24.6467(10)
α/°	90
β/°	103.192(4)
γ/°	90
V/Å^3	2200.54(15)
Z	4
Z'	1
Wavelength/Å	1.54184
Radiation type	Cu K_α
Θ_{min}/°	2.99
Θ_{max}/°	73.05
Measured Refl's.	40152
Indep't Refl's	4321
Refl's I≥2 σ(I)	3843
R_{int}	0.0371
Parameters	255
Restraints	0
Largest Peak	1.1133
Deepest Hole	-0.6202
GooF	1.0370
wR_2 (all data)	0.1913
wR_2	0.1880
R_1 (all data)	0.0812
R_1	0.0756

Sample preparation. In a 5 mL glass vial, the purified nitrile **3g** was layered with 1 mL petroleum ether. Then CH_2Cl_2 was slowly added until the sample was completely dissolved. The vial was sealed with a plastic cap with a small hole to allow CH_2Cl_2 to evaporate. The sample was kept in the refrigerator until a crystal reached a sufficient size.

Experimental. Single colourless needle-shaped crystals of **3g** were used as supplied. A suitable crystal with dimensions 0.23 × 0.05 × 0.02 mm^3 was selected and mounted on a XtaLAB Synergy R, DW system, HyPix-Arc 150 diffractometer. The crystal was kept at a steady *T* = 123.00(10) K during data collection. The structure was solved with the ShelXT 2018/2[28] solution program using dual methods and by using Olex2 1.5-alpha as the graphical interface.[29] The model was refined with ShelXL 2018/3[30] using full matrix least squares minimisation on $\boldsymbol{F^2}$.

4-(*tert*-Butyl)-1-tosylcyclohexane-1-carbonitrile (3h).

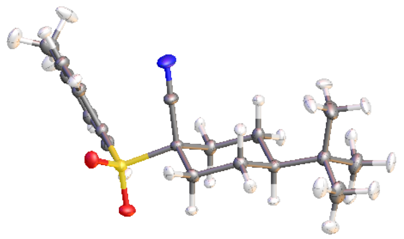

Compound	3h
Formula	$C_{18}H_{25}NO_2S$
$D_{calc.}$/ g cm^{-3}	1.229
μ/mm^{-1}	1.711
Formula Weight	319.471
Colour	clear colourless
Shape	needle-shaped
Size/mm^3	0.20×0.09×0.08
T/K	123.00(10)
Crystal System	monoclinic
Space Group	$P2_1/n$
a/Å	10.7133(2)
b/Å	6.2857(1)
c/Å	26.0530(4)
α/°	90
β/°	100.215(2)
γ/°	90
V/Å^3	1726.62(5)
Z	4
Z'	1
Wavelength/Å	1.54184
Radiation type	Cu K[illegible]
Θ_{min}/°	3.45
Θ_{max}/°	73.03
Measured Refl's.	33753
Indep't Refl's	3428
Refl's I≥2 σ(I)	3325
R_{int}	0.0170
Parameters	424
Restraints	0
Largest Peak	0.1597
Deepest Hole	-0.1400
GooF	1.1945
wR_2 (all data)	0.0255
wR_2	0.0253
R_1 (all data)	0.0114
R_1	0.0108

Sample preparation. In a 5 mL glass vial, the purified nitrile **3h** was layered with 1 mL petroleum ether. Then CH_2Cl_2 was slowly added until the sample was completely dissolved. The vial was sealed with a plastic cap with a small hole to allow CH_2Cl_2 to evaporate. The sample was kept in the refrigerator until a crystal reached a sufficient size.

Experimental. Single clear colourless needle-shaped crystals of **3h** were used as supplied. A suitable crystal with dimensions 0.20 × 0.09 × 0.08 mm^3 was selected and mounted on a MITIGEN holder inert oil on a XtaLAB Synergy R, DW system, HyPix-Arc 150 diffractometer. The crystal was kept at a steady T = 123.00(10) K during data collection. The structure was solved with the ShelXT 2018/2[28] solution program using dual methods and by using Olex2 1.5-alpha as the graphical interface.[29] The model was refined with ShelXL 2018/3[30] using full matrix least squares minimisation on $\boldsymbol{F}^2$.

tert-butyl 3-cyano-3-tosyl-8-azabicyclo[3.2.1]octane-8-carboxylate (3i).

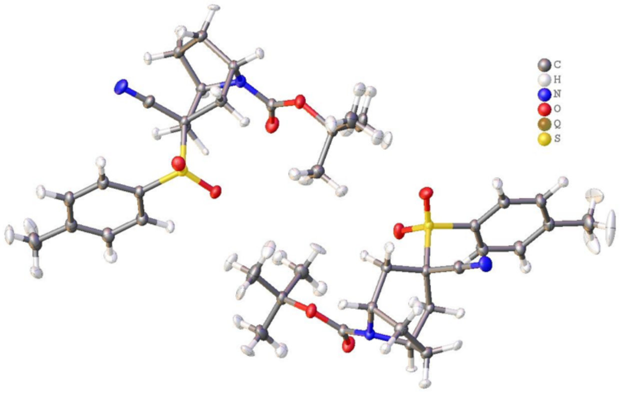

Sample preparation. In a 5 mL glass vial, the purified nitrile **3i** was layered with 1 mL petroleum ether. Then CH_2Cl_2 was slowly added until the sample was completely dissolved. The vial was sealed with a plastic cap with a small hole to allow CH_2Cl_2 to evaporate. The sample was kept in the refrigerator until a crystal reached a sufficient size.

Experimental. Single clear colourless prism-shaped crystals of **3i** were used as supplied. A suitable crystal with dimensions 0.14 × 0.13 × 0.07 mm^3 was selected and mounted on a MITIGEN holder inert oil on a XtaLAB Synergy R, DW system, HyPix-Arc 150 diffractometer. The crystal was kept at a steady T = 123.00(10) K during data collection. The structure was solved with the ShelXT 2018/2[28] solution program using dual methods and by using Olex2 1.5-alpha as the graphical interface.[29] The model was refined with ShelXL 2018/3[30] using full matrix least squares minimisation on $\boldsymbol{F^2}$.

**Compound**	**3i**
Formula	$C_{20}H_{26}N_2O_4S$
$D_{calc.}$/ g cm^{-3}	1.311
μ/mm^{-1}	1.688
Formula Weight	390.507
Colour	clear colourless
Shape	prism-shaped
Size/mm^3	0.14×0.13×0.07
T/K	123.00(10)
Crystal System	orthorhombic
Space Group	-0.014(2)
a/Å	-0.014(2)
b/Å	$Pca2_1$
c/Å	35.7699(5)
α/°	6.4182(1)
β/°	17.2309(2)
γ/°	90
V/Å^3	90
Z	90
Z'	3955.84(10)
Wavelength/Å	8
Radiation type	2
Θ_{min}/°	1.54184
Θ_{max}/°	Cu K[illegible]
Measured Refl's.	2.47
Indep't Refl's	73.00
Refl's I≥2 σ(I)	45727
R_{int}	7373
Parameters	7266
Restraints	0.0166
Largest Peak	955
Deepest Hole	1
GooF	0.0791
wR_2 (all data)	-0.0783
wR_2	1.0762
R_1 (all data)	0.0407
R_1	0.0406

3-Tosyloxetane-3-carbonitrile (3k).

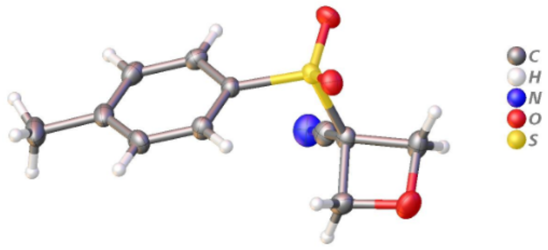

**Compound**	**3k**
Formula	$C_{11}H_{11}NO_3S$
$D_{calc.}$/ g cm^{-3}	1.417
μ/mm^{-1}	2.537
Formula Weight	237.281
Colour	clear yellow
Shape	prism-shaped
Size/mm^3	0.23×0.18×0.16
T/K	123.00(10)
Crystal System	triclinic
Space Group	*P*-1
a/Å	8.1516(3)
b/Å	8.4094(4)
c/Å	8.5413(4)
α/°	72.013(4)
β/°	89.046(3)
γ/°	86.837(3)
V/Å^3	556.04(4)
Z	2
Z'	1
Wavelength/Å	1.54184
Radiation type	Cu K_α
Θ_{min}/°	5.44
Θ_{max}/°	73.31
Measured Refl's.	18834
Indep't Refl's	2168
Refl's I≥2 σ(I)	2158
R_{int}	0.0215
Parameters	146
Restraints	0
Largest Peak	0.3423
Deepest Hole	-0.3147
GooF	1.0424
wR_2 (all data)	0.0811
wR_2	0.0811
R_1 (all data)	0.0303
R_1	0.0303

Sample preparation. In a 5 mL glass vial, the purified nitrile **3k** was layered with 1 mL petroleum ether. Then CH_2Cl_2 was slowly added until the sample was completely dissolved. The vial was sealed with a plastic cap with a small hole to allow CH_2Cl_2 to evaporate. The sample was kept in the refrigerator until a crystal reached a sufficient size.

Experimental. Single clear yellow prism-shaped crystals of **3k** were used as supplied. A suitable crystal with dimensions 0.23 × 0.18 × 0.16 mm^3 was selected and mounted on a XtaLAB Synergy R, DW system, HyPix-Arc 150 diffractometer. The crystal was kept at a steady T = 123.00(10) K during data collection. The structure was solved with the ShelXT 2018/2[28] solution program using dual methods and by using Olex2 1.5-alpha as the graphical interface.[29] The model was refined with ShelXL 2018/3[30] using full matrix least squares minimisation on $\boldsymbol{F}^2$.

5-((1-Cyanocyclohexyl)sulfonyl)-2-methoxyphenyl acetate (3y).

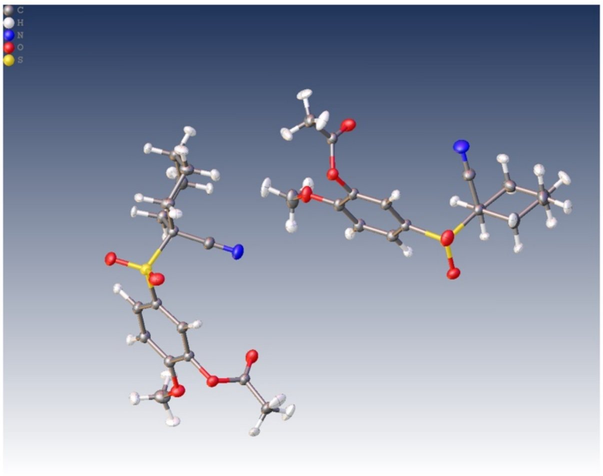

Compound	3y
Formula	$C_{16}H_{19}NO_5S$
$D_{calc.}$/ g cm^{-3}	1.377
μ/mm^{-1}	1.995
Formula Weight	337.399
Colour	clear colourless
Shape	prism-shaped
Size/mm^3	0.24×0.04×0.03
T/K	123.00(10)
Crystal System	monoclinic
Space Group	$P2_1/n$
a/Å	18.0257(3)
b/Å	6.7171(1)
c/Å	27.4070(5)
α/°	90
β/°	101.122(2)
γ/°	90
V/Å^3	3256.13(10)
Z	8
Z'	2
Wavelength/Å	1.54184
Radiation type	Cu K_a
Θ_{min}/°	2.71
Θ_{max}/°	75.85
Measured Refl's.	66786
Indep't Refl's	6698
Refl's I≥2 σ(I)	6137
R_{int}	0.0298
Parameters	757
Restraints	0
Largest Peak	0.1835
Deepest Hole	-0.2067
GooF	1.0576
wR_2 (all data)	0.0412
wR_2	0.0402
R_1 (all data)	0.0223
R_1	0.0194

Sample preparation. In a 5 mL glass vial, the purified nitrile **3y** was layered with 1 mL petroleum ether. Then CH_2Cl_2 was slowly added until the sample was completely dissolved. The vial was sealed with a plastic cap with a small hole to allow CH_2Cl_2 to evaporate. The sample was kept in the refrigerator until a crystal reached a sufficient size.

Experimental. Single clear colourless prism-shaped crystals of **3y** were used as supplied. A suitable crystal with dimensions 0.24 × 0.04 × 0.03 mm^3 was selected and mounted with inert oil on a XtaLAB Synergy R, DW system, HyPix-Arc 150 diffractometer. The crystal was kept at a steady T = 123.00(10) K during data collection. The structure was solved with the ShelXT 2018/2[28] solution program using dual methods and by using Olex2 1.5-alpha as the graphical interface.[29] The model was refined with ShelXL 2018/3[30] using full matrix least squares minimisation on F^2.

1-((3-Chloro-4-methoxyphenyl)sulfonyl)cyclohexane-1-carbonitrile (3z).

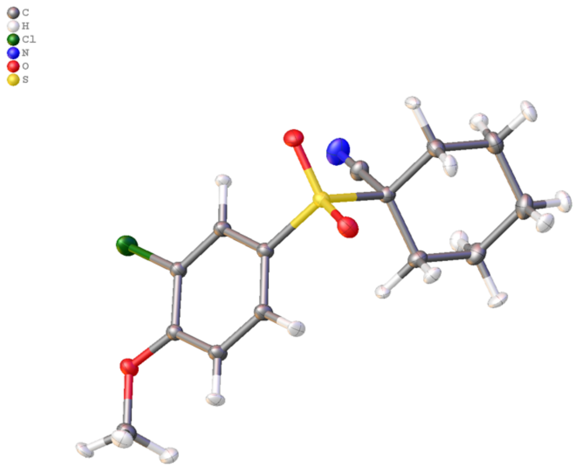

Compound	3z
Formula	$C_{14}H_{16}ClNO_3S$
$D_{calc.}$/ g cm^{-3}	1.462
μ/mm^{-1}	3.806
Formula Weight	313.807
Colour	clear colourless
Shape	prism-shaped
Size/mm^3	0.12×0.09×0.09
T/K	123.00(10)
Crystal System	orthorhombic
Space Group	*Pbca*
a/Å	11.8791(2)
b/Å	12.1244(2)
c/Å	19.7982(3)
α/°	90
β/°	90
γ/°	90
V/Å^3	2851.47(8)
Z	8
Z'	1
Wavelength/Å	1.54184
Radiation type	Cu K_a
Θ_{min}/°	4.47
Θ_{max}/°	75.13
Measured Refl's.	33524
Indep't Refl's	2919
Refl's I≥2 σ(I)	2733
R_{int}	0.0256
Parameters	325
Restraints	0
Largest Peak	0.1043
Deepest Hole	-0.0918
GooF	1.0473
wR_2 (all data)	0.0214
wR_2	0.0211
R_1 (all data)	0.0123
R_1	0.0105

Sample preparation. In a 5 mL glass vial, the purified nitrile **3z** was layered with 1 mL petroleum ether. Then CH_2Cl_2 was slowly added until the sample was completely dissolved. The vial was sealed with a plastic cap with a small hole to allow CH_2Cl_2 to evaporate. The sample was kept in the refrigerator until a crystal reached a sufficient size.

Experimental. Single clear colourless prism-shaped crystals of **3z** were used as supplied. A suitable crystal with dimensions 0.12 × 0.09 × 0.09 mm^3 was selected and mounted with inert oil on a XtaLAB Synergy R, DW system, HyPix-Arc 150 diffractometer. The crystal was kept at a steady *T* = 123.00(10) K during data collection. The structure was solved with the ShelXT 2018/2[28] solution program using dual methods and by using Olex2 1.5-alpha as the graphical interface.[29] The model was refined with ShelXL 2018/3[30] using full matrix least squares minimisation on ***F*^{2}**.

1-(Benzo[*d*][1,3]dioxol-5-ylsulfonyl)cyclohexane-1-carbonitrile (3aa).

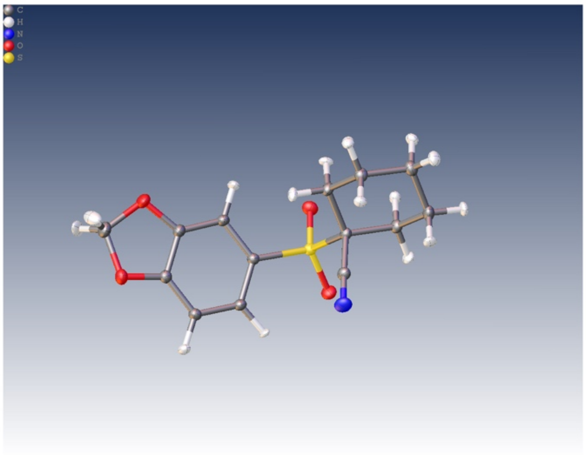

Compound	3aa
Formula	$C_{14}H_{15}NO_4S$
$D_{calc.}$/ g cm^{-3}	1.464
μ/mm^{-1}	2.294
Formula Weight	293.346
Colour	clear colourless
Shape	prism-shaped
Size/mm^3	0.17×0.10×0.07
T/K	123.00(10)
Crystal System	monoclinic
Space Group	$P2_1/c$
a/Å	9.3093(2)
b/Å	18.4996(3)
c/Å	7.8610(1)
α/°	90
β/°	100.615(2)
γ/°	90
V/Å^3	1330.64(4)
Z	4
Z'	1
Wavelength/Å	1.54184
Radiation type	Cu K_a
Θ_{min}/°	4.78
Θ_{max}/°	75.30
Measured Refl's.	21780
Indep't Refl's	2734
Refl's I≥2 σ(I)	2597
R_{int}	0.0243
Parameters	316
Restraints	0
Largest Peak	0.0845
Deepest Hole	-0.0963
GooF	1.1380
wR_2 (all data)	0.0222
wR_2	0.0220
R_1 (all data)	0.0121
R_1	0.0110

Sample preparation. In a 5 mL glass vial, the purified nitrile **3aa** was layered with 1 mL petroleum ether. Then CH_2Cl_2 was slowly added until the sample was completely dissolved. The vial was sealed with a plastic cap with a small hole to allow CH_2Cl_2 to evaporate. The sample was kept in the refrigerator until a crystal reached a sufficient size.

Experimental. Single clear colourless prism-shaped crystals of **3aa** were used as supplied. A suitable crystal with dimensions 0.17 × 0.10 × 0.07 mm^3 was selected and mounted with inert oil on a XtaLAB Synergy R, DW system, HyPix-Arc 150 diffractometer. The crystal was kept at a steady T = 123.00(10) K during data collection. The structure was solved with the ShelXT 2018/2[28] solution program using dual methods and by using Olex2 1.5-alpha as the graphical interface.[29] The model was refined with ShelXL 2018/3[30] using full matrix least squares minimisation on $\boldsymbol{F}^2$.

4-((3-(1,3-Dioxoisoindolin-2-yl)propyl)sulfonyl)-1-tosylpiperidine-4-carbonitrile (3an).

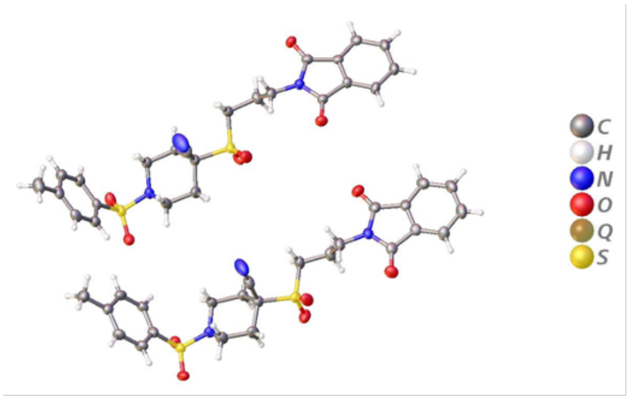

**Compound**	**3an**
Formula	$C_{24.75}H_{27.5}Cl_{1.5}N_3O_{6.5}S_2$
$D_{calc.}$/ g cm^{-3}	1.453
μ/mm^{-1}	3.575
Formula Weight	588.321
Colour	clear colourless
Shape	needle-shaped
Size/mm^3	0.30×0.05×0.02
T/K	123.00(10)
Crystal System	monoclinic
Space Group	$P2_1/n$
a/Å	22.7793(4)
b/Å	7.8406(1)
c/Å	30.7665(6)
α/°	90
β/°	101.751(2)
γ/°	90
V/Å^3	5379.84(16)
Z	8
Z'	2
Wavelength/Å	1.54184
Radiation type	Cu K_{α}
Θ_{min}/°	2.93
Θ_{max}/°	74.92
Measured Refl's.	94751
Indep't Refl's	10596
Refl's I≥2 σ(I)	9055
R_{int}	0.0434
Parameters	633
Restraints	0
Largest Peak	0.7695
Deepest Hole	-0.5907
GooF	1.0439
wR_2 (all data)	0.2114
wR_2	0.2061
R_1 (all data)	0.0799
R_1	0.0716

Sample preparation. In a 5 mL glass vial, the purified nitrile **3an** was layered with 1 mL petroleum ether. Then CH_2Cl_2 was slowly added until the sample was completely dissolved. The vial was sealed with a plastic cap with a small hole to allow CH_2Cl_2 to evaporate. The sample was kept in the refrigerator until a crystal reached a sufficient size.

Experimental. Single clear colourless needle-shaped crystals of **3an** were used as supplied. A suitable crystal with dimensions 0.30 × 0.05 × 0.02 mm^3 was selected and mounted on a XtaLAB Synergy R, DW system, HyPix-Arc 150 diffractometer. The crystal was kept at a steady T = 123.00(10) K during data collection. The structure was solved with the ShelXT 2018/2[28] solution program using dual methods and by using Olex2 1.5-alpha as the graphical interface.[29] The model was refined with ShelXL 2018/3[30] using full matrix least squares minimisation on $\boldsymbol{F^2}$.

1-(Phenylsulfinyl)cyclohexane-1-carbonitrile (5a).

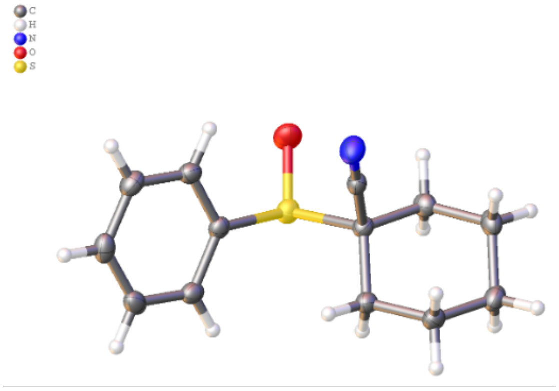

Sample preparation. In a 5 mL glass vial, the purified nitrile **5a** was layered with 1 mL petroleum ether. Then CH_2Cl_2 was slowly added until the sample was completely dissolved. The vial was sealed with a plastic cap with a small hole to allow CH_2Cl_2 to evaporate. The sample was kept in the refrigerator until a crystal reached a sufficient size.

Experimental. Single clear colourless plate-shaped crystals of **5a** were used as supplied. A suitable crystal with dimensions 0.16 × 0.04 × 0.02 mm^3 was selected and mounted on a XtaLAB Synergy R, DW system, HyPix-Arc 150 diffractometer. The crystal was kept at a steady *T* = 122.99(10) K during data collection. The structure was solved with the ShelXT 2018/2[28] solution program using dual methods and by using Olex2 1.5-alpha as the graphical interface.[29] The model was refined with ShelXL 2018/3[30] using full matrix least squares minimisation on $\boldsymbol{F}^2$.

**Compound**	**5a**
Formula	$C_{13}H_{15}NOS$
$D_{calc.}$/ g cm^{-3}	1.307
m/mm^{-1}	2.236
Formula Weight	233.32
Colour	clear colourless
Shape	plate-shaped
Size/mm^3	0.16×0.04×0.02
T/K	122.99(10)
Crystal System	monoclinic
Space Group	$P2_1/c$
a/Å	12.1407(5)
b/Å	6.1710(2)
c/Å	16.8735(7)
a/°	90
b/°	110.325(5)
g/°	90
V/Å^3	1185.46(9)
Z	4
Z'	1
Wavelength/Å	1.54184
Radiation type	Cu K_a
Q_{min}/°	3.883
Q_{max}/°	73.729
Measured Refl's.	21387
Indep't Refl's	2347
Refl's I≥2 *s*(I)	2021
R_{int}	0.0366
Parameters	145
Restraints	0
Largest Peak	0.463
Deepest Hole	-0.358
GooF	1.040
wR_2 (all data)	0.1012
wR_2	0.0969
R_1 (all data)	0.0439
R_1	0.0368

3,3-Dimethoxy-8-tosyl-4-oxa-1,2,8-triazaspiro[4.5]dec-1-ene (1g).

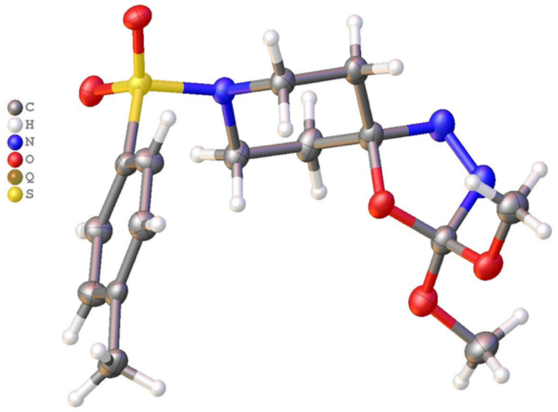

Sample preparation. In a 5 mL glass vial, the purified oxadiazoline **1g** was dissolved in 1:1 mixture of PE and EtOAc. The solvent was allowed to evaporate until a crystal reached a sufficient size.

Experimental. Single clear colourless plate-shaped crystals of **X023** were used as supplied. A suitable crystal with dimensions 0.08 × 0.03 × 0.02 mm^3 was selected and mounted on a MITIGEN holder inert oil on a XtaLAB Synergy R, DW system, HyPix-Arc 150 diffractometer. The crystal was kept at a steady T = 122.8(3) K during data collection. The structure was solved with the ShelXT 2018/2[28] solution program using dual methods and by using Olex2 1.5-alpha as the graphical interface.[29] The model was refined with ShelXL 2018/3[30] using full matrix least squares minimisation on $\boldsymbol{F^2}$.

**Compound**	**1g**
Formula	$C_{15}H_{21}N_3O_5S$
$D_{calc.}$/ g cm^{-3}	1.403
μ/mm^{-1}	1.990
Formula Weight	355.41
Colour	clear colourless
Shape	plate-shaped
Size/mm^3	0.08×0.03×0.02
T/K	122.8(3)
Crystal System	monoclinic
Space Group	$P2_1/c$
a/Å	12.2947(6)
b/Å	6.8153(3)
c/Å	20.5609(10)
α/°	90
β/°	102.448(4)
γ/°	90
V/Å^3	1682.34(14)
Z	4
Z'	1
Wavelength/Å	1.54184
Radiation type	Cu K_α
Θ_{min}/°	3.682
Θ_{max}/°	74.526
Measured Refl's.	36045
Indep't Refl's	3427
Refl's I≥2 σ(I)	3234
R_{int}	0.0322
Parameters	301
Restraints	0
Largest Peak	0.351
Deepest Hole	-0.338
GooF	1.051
wR_2 (all data)	0.0896
wR_2	0.0887
R_1 (all data)	0.0339
R_1	0.0323

8 Literature

(1) Xia, Y.; Tang, M. Umpolung of N-Tosylhydrazones: Synthesis of N-Cyano Tosylhydrazones and Their [3,3]-Sigmatropic Rearrangement to 2-Aminoimidazoles. *J. Org. Chem.* **2024**, *89* (24), 18764–18768. https://doi.org/10.1021/acs.joc.4c02580.

(2) Neese, F. The ORCA Program System. *WIREs Comput. Mol. Sci.* **2012**, *2* (1), 73–78. https://doi.org/10.1002/wcms.81.

(3) Neese, F. Software Update: The ORCA Program System—Version 5.0. *WIREs Comput. Mol. Sci.* **2022**, *12* (5), e1606. https://doi.org/10.1002/wcms.1606.

(4) Becke, A. D. Density-functional Thermochemistry. III. The Role of Exact Exchange. *J. Chem. Phys.* **1993**, *98* (7), 5648–5652. https://doi.org/10.1063/1.464913.

(5) Grimme, S.; Ehrlich, S.; Goerigk, L. Effect of the Damping Function in Dispersion Corrected Density Functional Theory. *J. Comput. Chem.* **2011**, *32* (7), 1456–1465. https://doi.org/10.1002/jcc.21759.

(6) Weigend, F. Accurate Coulomb-Fitting Basis Sets for H to Rn. *Phys. Chem. Chem. Phys.* **2006**, *8* (9), 1057–1065. https://doi.org/10.1039/B515623H.

(7) Weigend, F.; Ahlrichs, R. Balanced Basis Sets of Split Valence, Triple Zeta Valence and Quadruple Zeta Valence Quality for H to Rn: Design and Assessment of Accuracy. *Phys. Chem. Chem. Phys.* **2005**, *7* (18), 3297–3305. https://doi.org/10.1039/B508541A.

(8) Barone, V.; Cossi, M. Quantum Calculation of Molecular Energies and Energy Gradients in Solution by a Conductor Solvent Model. *J. Phys. Chem. A* **1998**, *102* (11), 1995–2001. https://doi.org/10.1021/jp9716997.

(9) RDKit: Open-Source Cheminformatics, Version 2023.09.6. https://www.rdkit.org.

(10) McInnes, L.; Healy, J.; Saul, N.; Großberger, L. UMAP: Uniform Manifold Approximation and Projection. *J. Open Source Softw.* **2018**, *3* (29), 861. https://doi.org/10.21105/joss.00861.

(11) Pedregosa, F.; Varoquaux, G.; Gramfort, A.; Michel, V.; Thirion, B.; Grisel, O.; Blondel, M.; Prettenhofer, P.; Weiss, R.; Dubourg, V.; Vanderplas, J.; Passos, A.; Cournapeau, D.; Brucher, M.; Perrot, M.; Duchesnay, É. Scikit-Learn: Machine Learning in Python. *J. Mach. Learn. Res.* **2011**, *12* (85), 2825–2830.

(12) Waskom, M. L. Seaborn: Statistical Data Visualization. *J. Open Source Softw.* **2021**, *6* (60), 3021. https://doi.org/10.21105/joss.03021.

(13) Hunter, J. D. Matplotlib: A 2D Graphics Environment. *Comput. Sci. Eng.* **2007**, *9* (3), 90–95. https://doi.org/10.1109/MCSE.2007.55.

(14) Vatèle, J.-M. 2-(Prenyloxymethyl)Benzoyl (POMB) as a New Temporary Protecting Group for Alcohols. *Tetrahedron Lett.* **2005**, *46* (13), 2299–2301. https://doi.org/10.1016/j.tetlet.2005.02.008.

(15) Jia, W.-L.; Westerveld, N.; Wong, K. M.; Morsch, T.; Hakkennes, M.; Naksomboon, K.; Fernández-Ibáñez, M. Á. Selective C–H Olefination of Indolines (C5) and Tetrahydroquinolines (C6) by Pd/S,O-Ligand Catalysis. *Org. Lett.* **2019**, *21* (23), 9339–9342. https://doi.org/10.1021/acs.orglett.9b03505.

(16) Orłowska, K.; Santiago, J. V.; Krajewski, P.; Kisiel, K.; Deperasińska, I.; Zawada, K.; Chaładaj, W.; Gryko, D. UV Light Is No Longer Required for the Photoactivation of 1,3,4-Oxadiazolines. *ACS Catal.* **2023**, *13* (3), 1964–1973. https://doi.org/10.1021/acscatal.2c05319.

(17) Huang, S.; Zeng, Z.; Zhang, N.; Qin, W.; Lan, Y.; Yan, H. Organocatalytic Asymmetric Deoxygenation of Sulfones to Access Chiral Sulfinyl Compounds. *Nat. Chem.* **2023**, *15* (2), 185–193. https://doi.org/10.1038/s41557-022-01120-x.

(18) Still, I. W. J.; Watson, I. D. G. An Efficient Synthetic Route to Aryl Thiocyanates from Arenesulfinates. *Synth. Commun.* **2001**, *31* (9), 1355–1359. https://doi.org/10.1081/SCC-100104044.

(19) Waddell, L. J. N.; Senkans, M. R.; Sutherland, A. Regioselective C–H Thiocyanation of Arenes by Iron(III) Chloride Catalysis. *J. Org. Chem.* **2023**, *88* (11), 7208–7218. https://doi.org/10.1021/acs.joc.3c00454.

(20) Pirenne, V.; Traboulsi, I.; Rouvière, L.; Lusseau, J.; Massip, S.; Bassani, D. M.; Robert, F.; Landais, Y. P-Anisaldehyde-Photosensitized Sulfonylcyanation of Chiral Cyclobutenes: Enantioselective Access to Cyclic and Acyclic Systems Bearing All-Carbon Quaternary Stereocenters. *Org. Lett.* **2020**, *22* (2), 575–579. https://doi.org/10.1021/acs.orglett.9b04345.

(21) Nagata, T.; Tamaki, A.; Kiyokawa, K.; Tsutsumi, R.; Yamanaka, M.; Minakata, S. Enantioselective Electrophilic Cyanation of Boron Enolates: Scope and Mechanistic Studies. *Chem. – Eur. J.* **2018**, *24* (64), 17027–17032. https://doi.org/10.1002/chem.201804455.

(22) Guo, W.; Tan, W.; Zhao, M.; Zheng, L.; Tao, K.; Chen, D.; Fan, X. Direct Photocatalytic S–H Bond Cyanation with Green "CN" Source. *J. Org. Chem.* **2018**, *83* (12), 6580–6588. https://doi.org/10.1021/acs.joc.8b00887.

(23) Kita, Y.; Takada, T.; Mihara, S.; Whelan, B. A.; Tohma, H. Novel and Direct Nucleophilic Sulfenylation and Thiocyanation of Phenol Ethers Using a Hypervalent Iodine(III) Reagent. *J. Org. Chem.* **1995**, *60* (22), 7144–7148. https://doi.org/10.1021/jo00127a018.

(24) Pirenne, V.; Kurtay, G.; Voci, S.; Bouffier, L.; Sojic, N.; Robert, F.; Bassani, D. M.; Landais, Y. Eosin-Mediated Alkylsulfonyl Cyanation of Olefins. *Org. Lett.* **2018**, *20* (15), 4521–4525. https://doi.org/10.1021/acs.orglett.8b01828.

(25) ISOPROPYL THIOCYANATE. *Org. Synth.* **1931**, *11*, 92. https://doi.org/10.15227/orgsyn.011.0092.

(26) Löser, R.; Fischer, S.; Hiller, A.; Köckerling, M.; Funke, U.; Maisonial, A.; Brust, P.; Steinbach, J. Use of 3-[18F]Fluoropropanesulfonyl Chloride as a Prosthetic Agent for the Radiolabelling of Amines: Investigation of Precursor Molecules, Labelling Conditions and Enzymatic Stability of the Corresponding Sulfonamides. *Beilstein J. Org. Chem.* **2013**, *9* (1), 1002–1011. https://doi.org/10.3762/bjoc.9.115.

(27) Zhang, G.; Xuan, L.; Zhao, Y.; Ding, C. Sulfuryl Fluoride Promoted Thiocyanation of Alcohols: A Practical Method for Preparing Thiocyanates. *Synlett* **2020**, *31*, 1413–1417. https://doi.org/10.1055/s-0040-1707151.

(28) Sheldrick, G. M. SHELXT – Integrated Space-Group and Crystal-Structure Determination. *Acta Crystallogr. Sect. Found. Adv.* **2015**, *71* (1), 3–8. https://doi.org/10.1107/S2053273314026370.
(29) Dolomanov, O. V.; Bourhis, L. J.; Gildea, R. J.; Howard, J. a. K.; Puschmann, H. OLEX2: A Complete Structure Solution, Refinement and Analysis Program. *J. Appl. Crystallogr.* **2009**, *42* (2), 339–341. https://doi.org/10.1107/S0021889808042726.
(30) Sheldrick, G. M. Crystal Structure Refinement with SHELXL. *Acta Crystallogr. Sect. C Struct. Chem.* **2015**, *71* (1), 3–8. https://doi.org/10.1107/S2053229614024218.

9 NMR Spectra

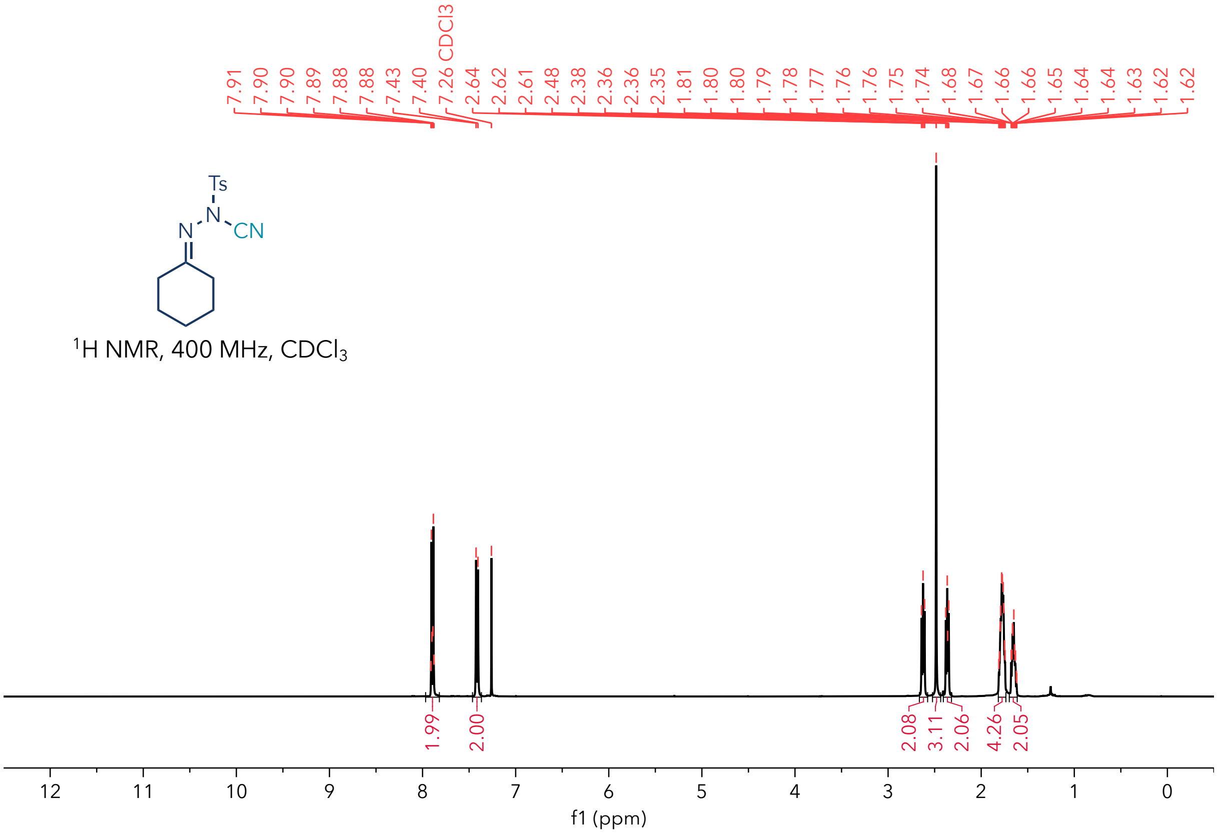

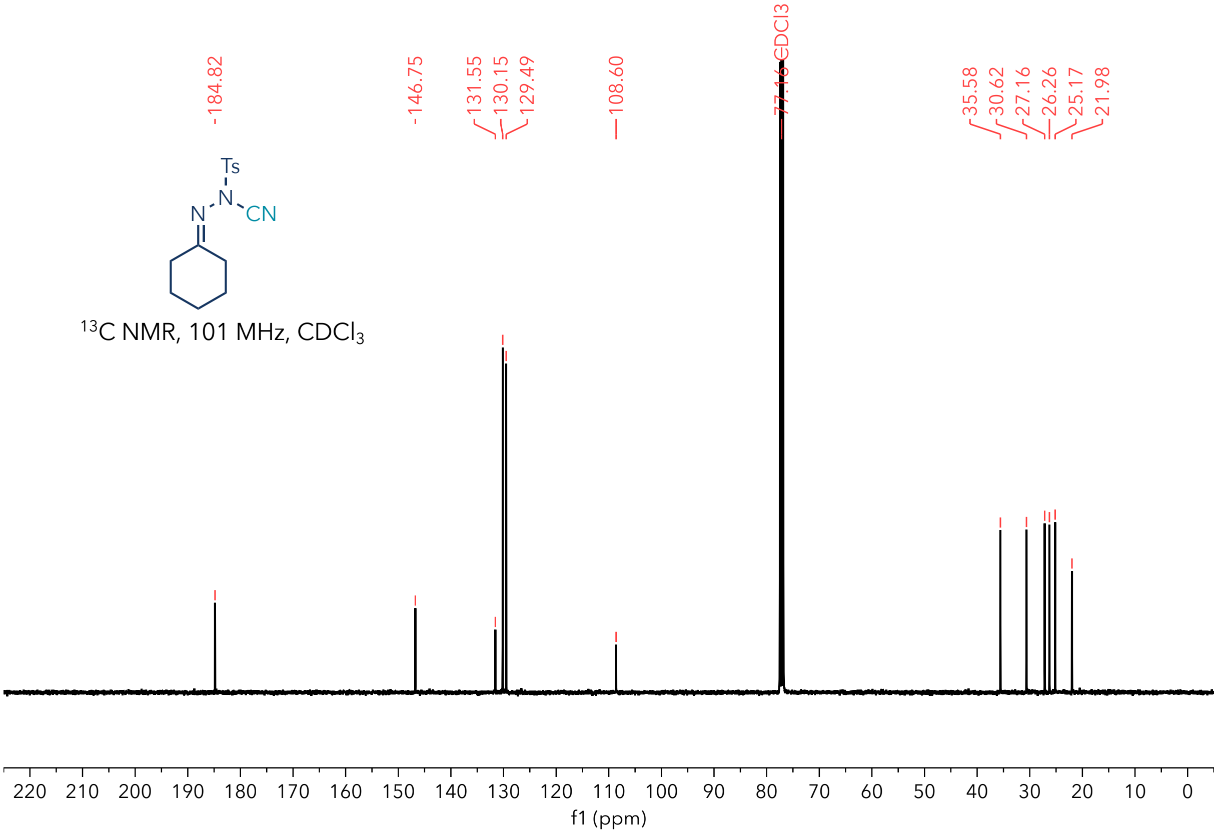

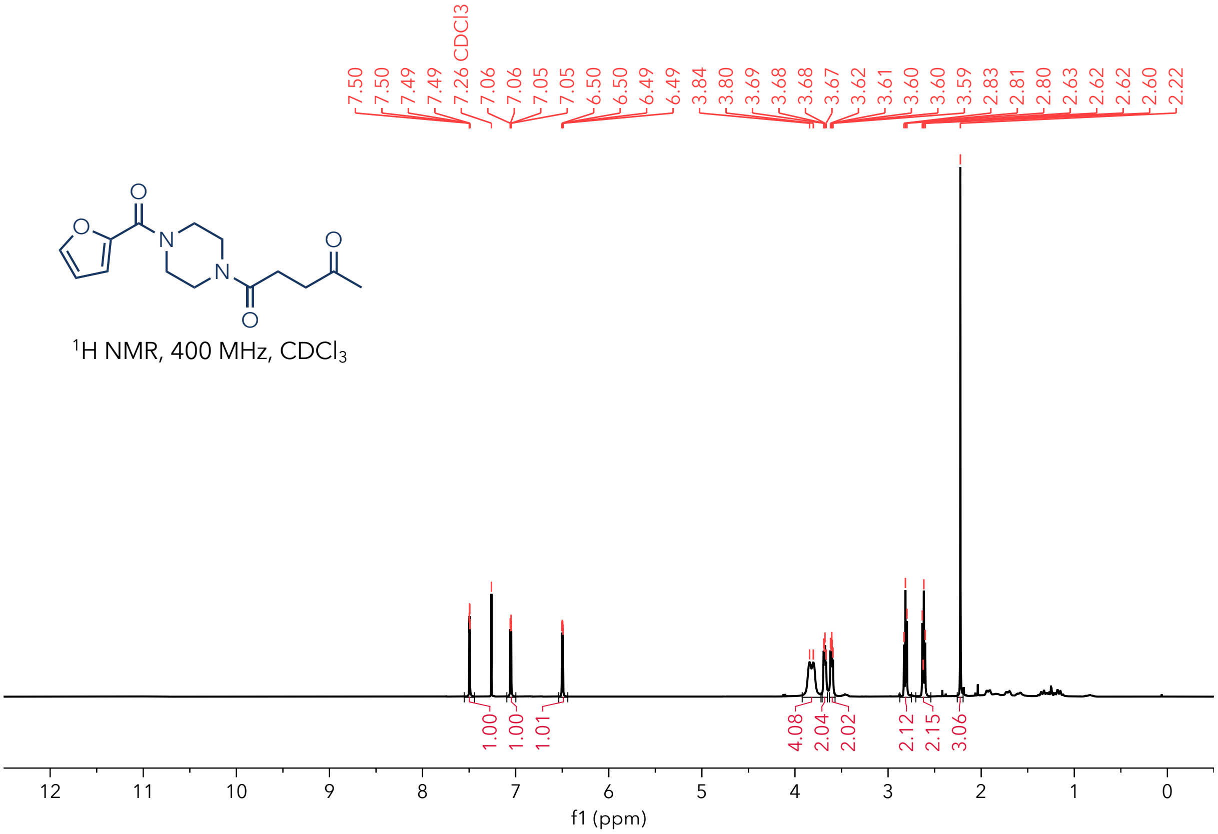
7.50 7.50 7.49 7.49 7.26 CDCl3 7.06 7.06 7.05 7.05 6.50 6.50 6.49 6.49 3.84 3.80 3.69 3.68 3.68 3.67 3.62 3.61 3.60 3.60 3.59 2.83 2.81 2.80 2.63 2.62 2.62 2.60 2.22
O
N
N
O
O
O
^{1}H NMR, 400 MHz, $CDCl_3$
1.00 1.00 1.01 4.08 2.04 2.02 2.12 2.15 3.06
12 11 10 9 8 7 6 5 4 3 2 1 0
f1 (ppm)

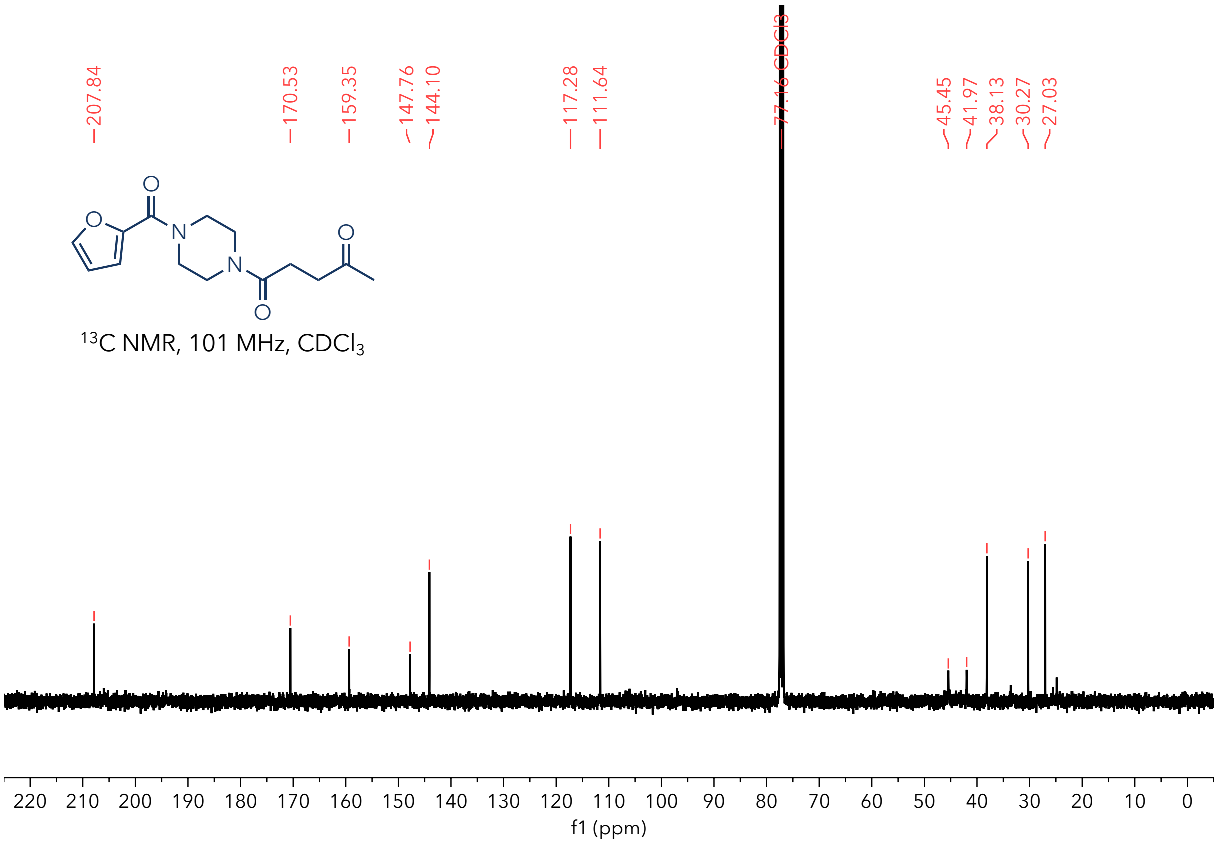
207.84 170.53 159.35 147.76 144.10 117.28 111.64 77.16 CDCl3 45.45 41.97 38.13 30.27 27.03
O
N
N
O
O
O
^{13}C NMR, 101 MHz, $CDCl_3$
220 210 200 190 180 170 160 150 140 130 120 110 100 90 80 70 60 50 40 30 20 10 0
f1 (ppm)

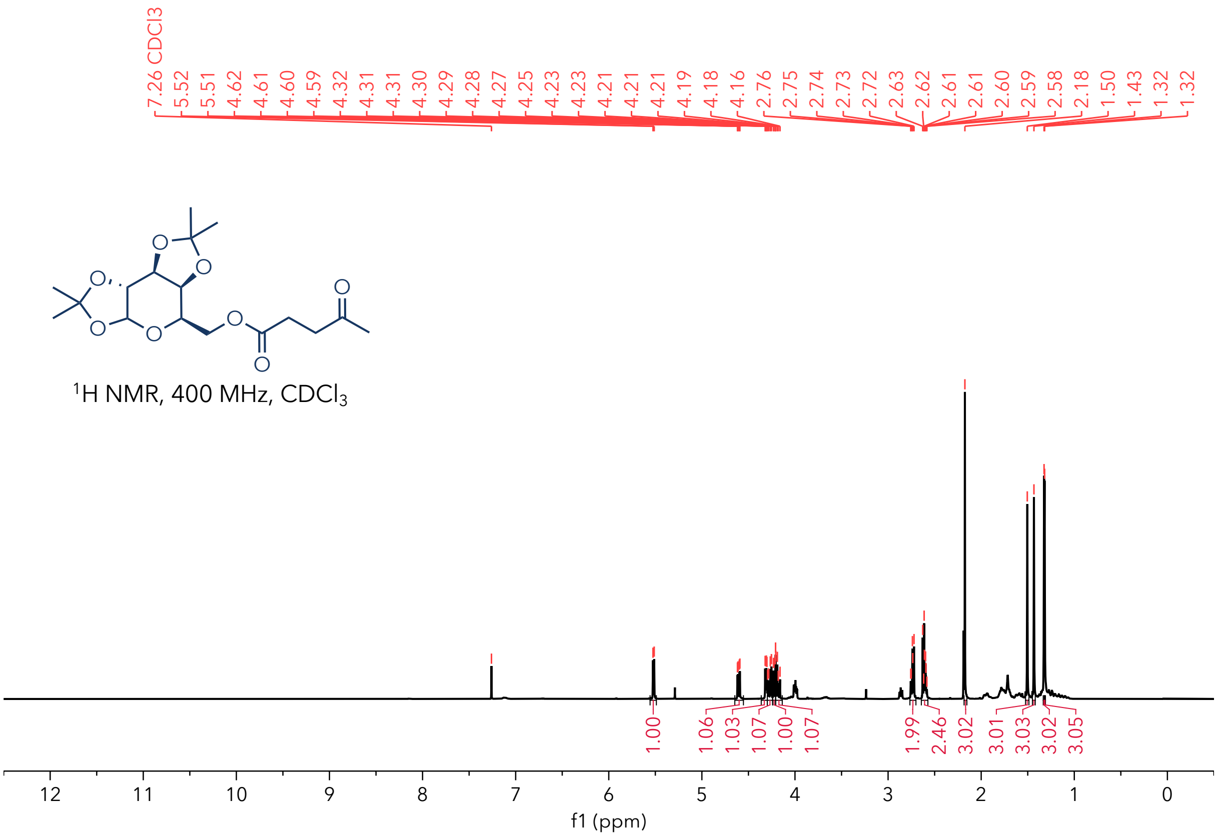

^{1}H NMR, 400 MHz, $CDCl_3$

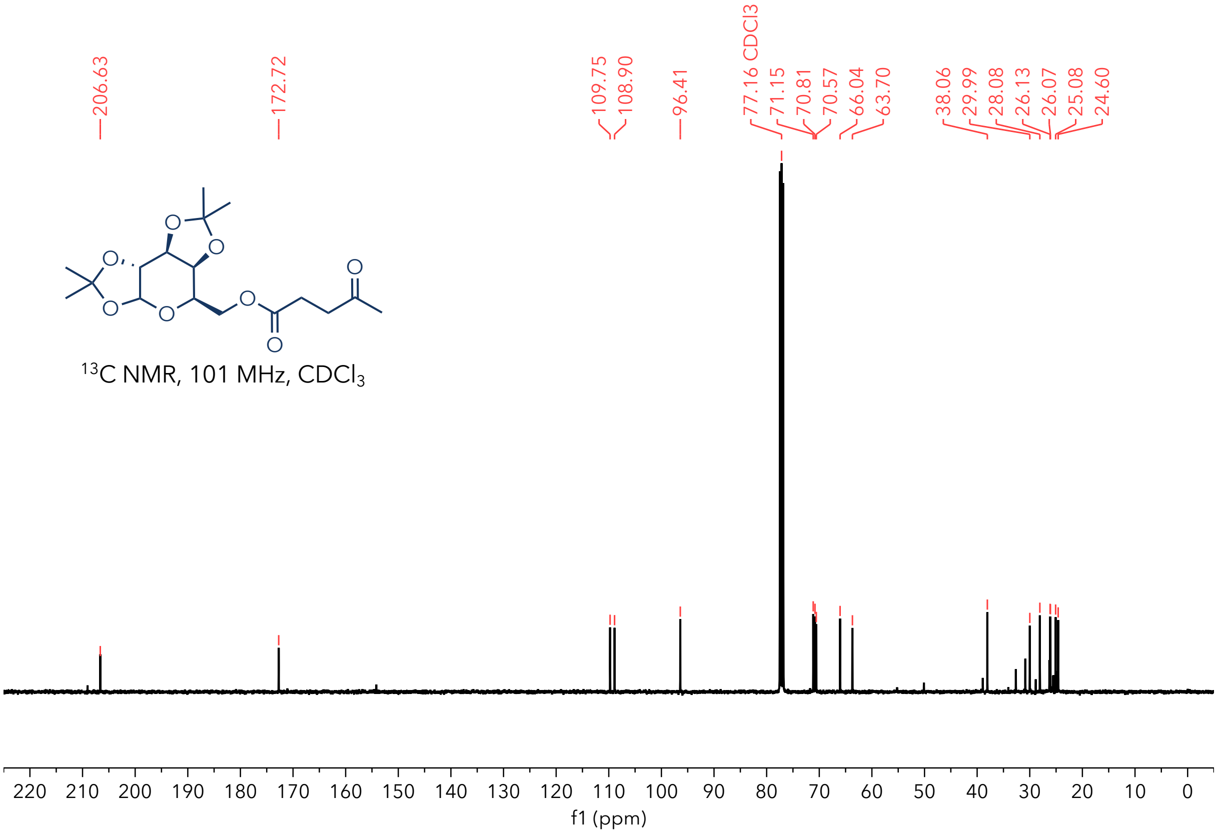

^{13}C NMR, 101 MHz, $CDCl_3$

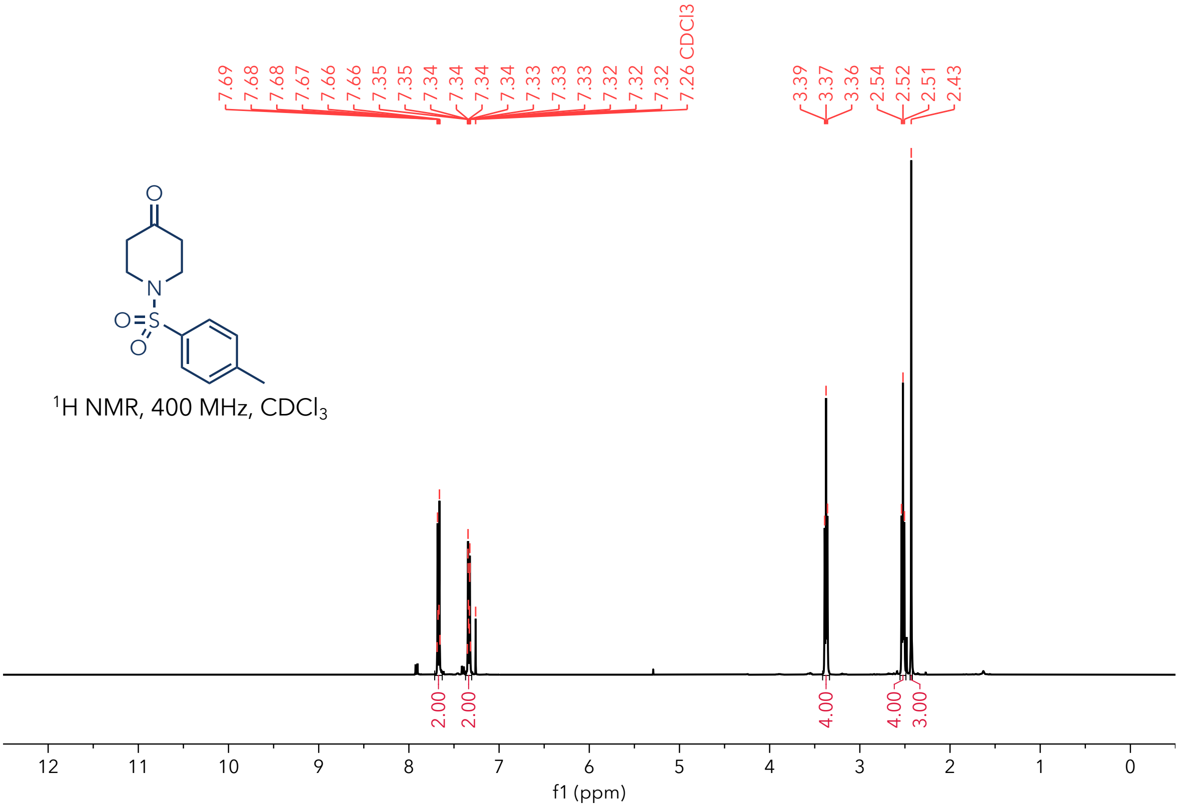

7.69
7.68
7.68
7.67
7.66
7.66
7.35
7.35
7.34
7.34
7.34
7.34
7.33
7.33
7.33
7.32
7.32
7.32
7.26 CDCl3
3.39
3.37
3.36
2.54
2.52
2.51
2.43
O
N
O=S
O
^{1}H NMR, 400 MHz, $CDCl_3$
2.00
2.00
4.00
4.00
3.00
12 11 10 9 8 7 6 5 4 3 2 1 0
f1 (ppm)

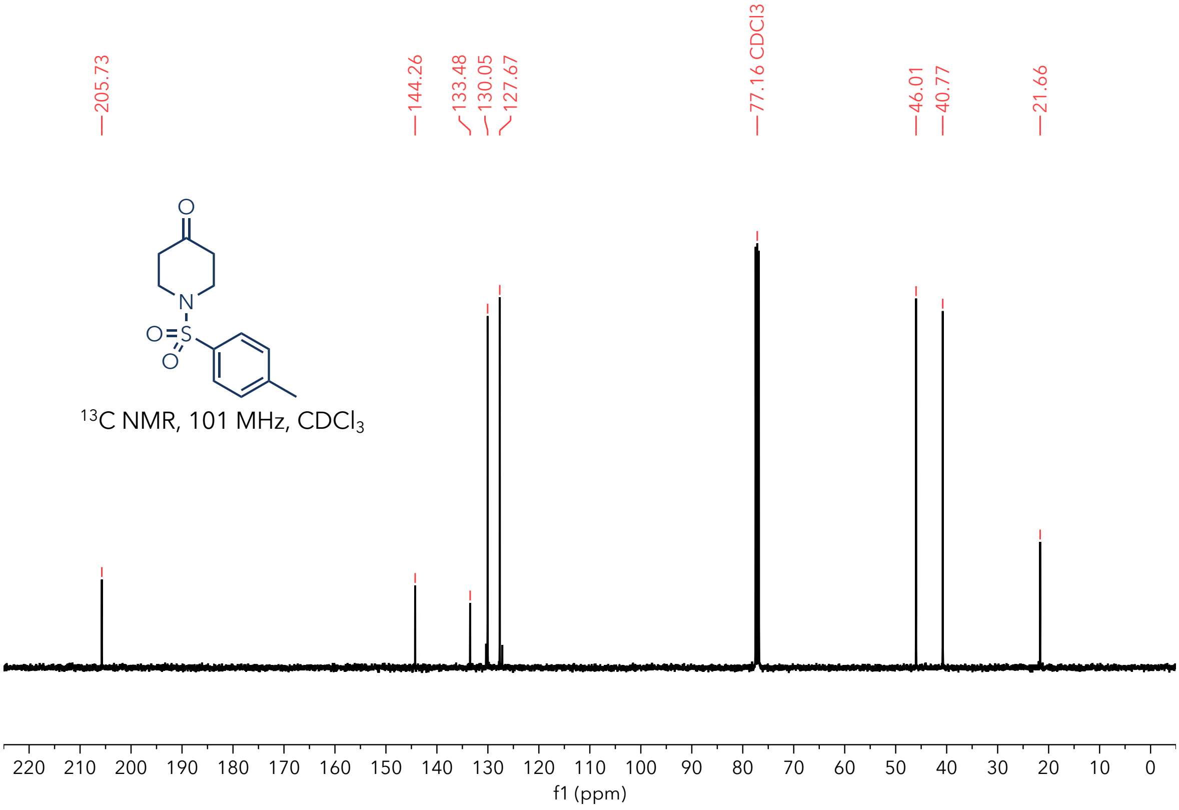

205.73
144.26
133.48
130.05
127.67
77.16 CDCl3
46.01
40.77
21.66
O
N
O=S
O
^{13}C NMR, 101 MHz, $CDCl_3$
220 210 200 190 180 170 160 150 140 130 120 110 100 90 80 70 60 50 40 30 20 10 0
f1 (ppm)

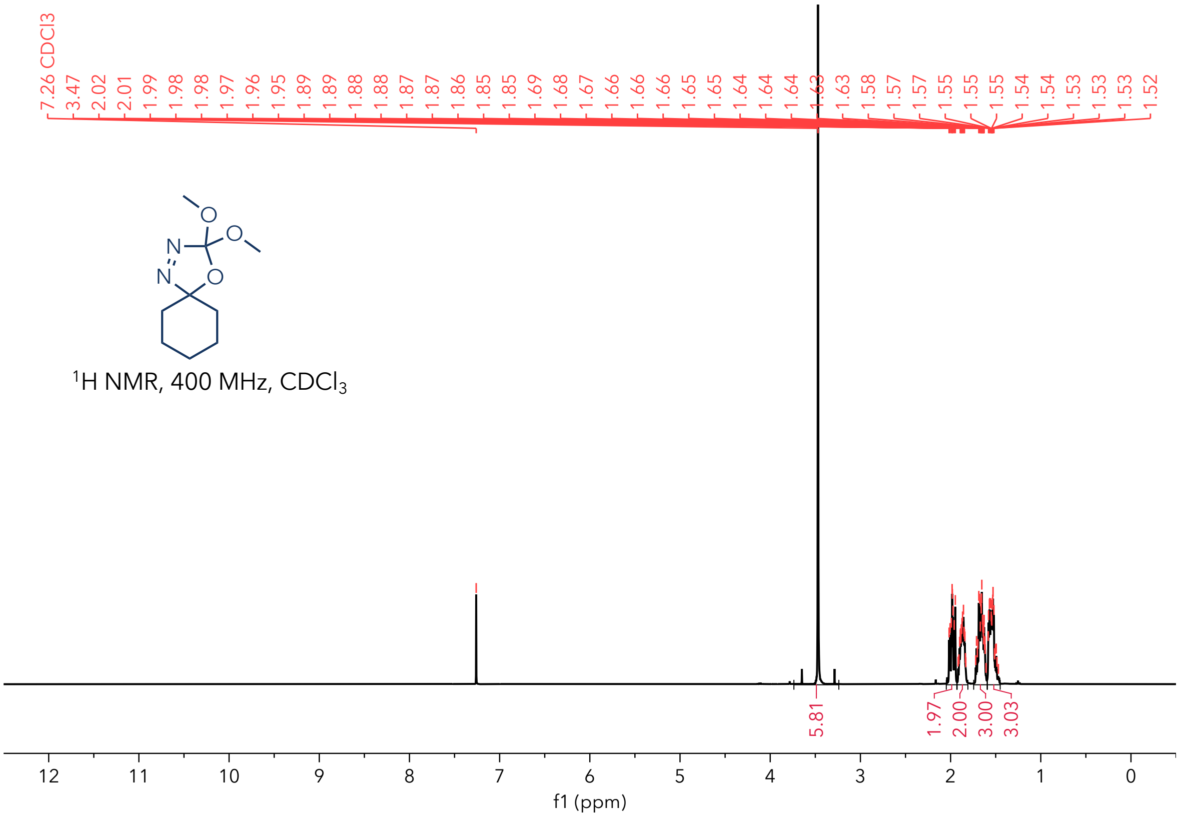
7.26 CDCl3
3.47
2.02
2.01
1.99
1.98
1.98
1.97
1.96
1.95
1.89
1.89
1.88
1.88
1.87
1.87
1.86
1.85
1.85
1.69
1.68
1.67
1.66
1.66
1.66
1.65
1.65
1.64
1.64
1.64
1.63
1.63
1.58
1.57
1.57
1.55
1.55
1.55
1.54
1.54
1.53
1.53
1.53
1.52
N
N
O
O
O
^{1}H NMR, 400 MHz, $CDCl_3$
5.81
1.97
2.00
3.00
3.03
12
11
10
9
8
7
6
5
4
3
2
1
0
f1 (ppm)

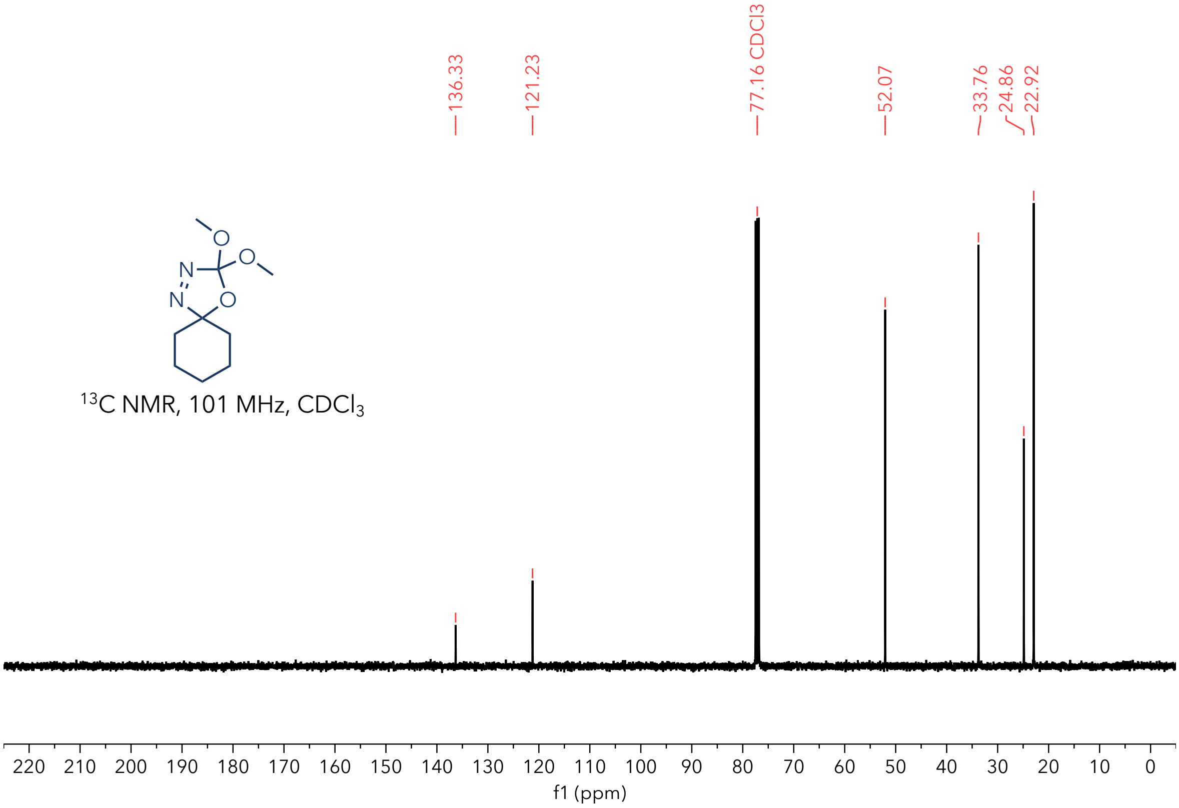
136.33
121.23
77.16 CDCl3
52.07
33.76
24.86
22.92
N
N
O
O
O
^{13}C NMR, 101 MHz, $CDCl_3$
220
210
200
190
180
170
160
150
140
130
120
110
100
90
80
70
60
50
40
30
20
10
0
f1 (ppm)

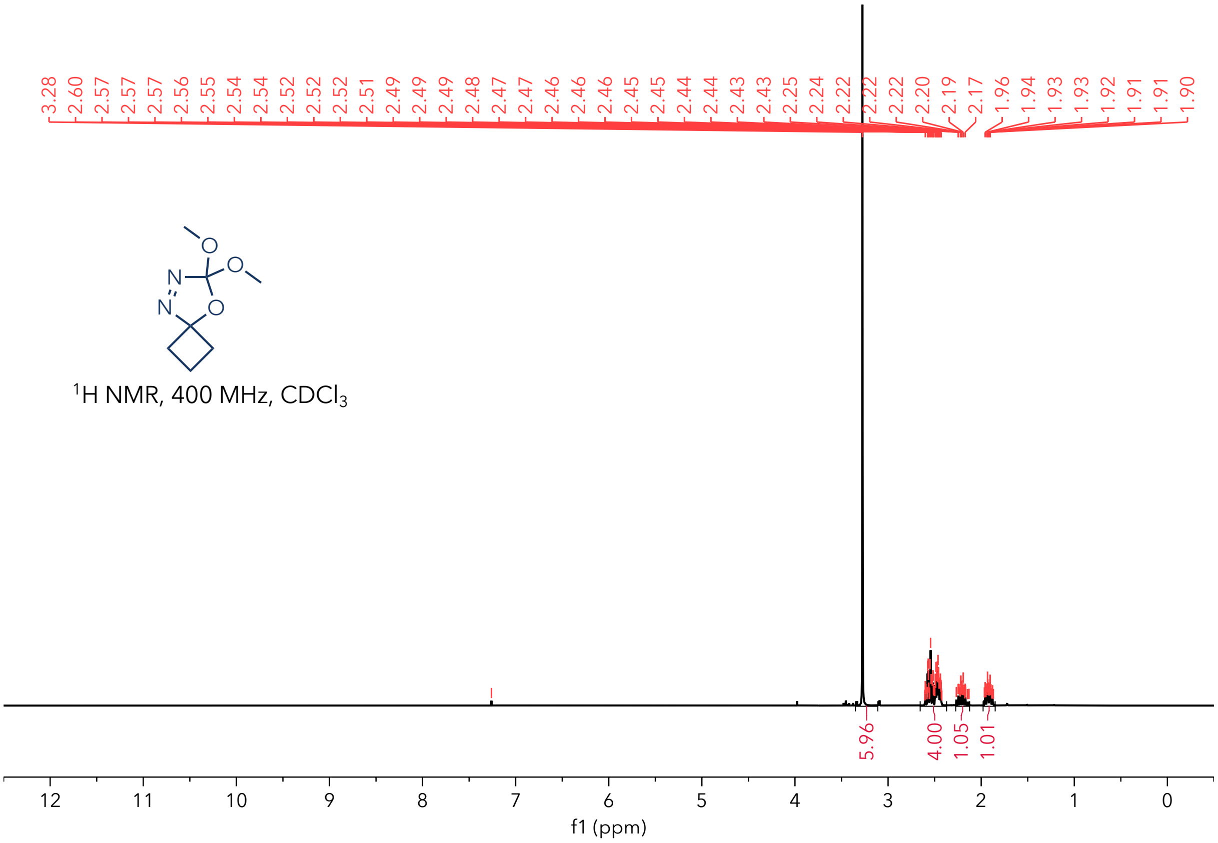
1H NMR, 400 MHz, CDCl3
f1 (ppm)

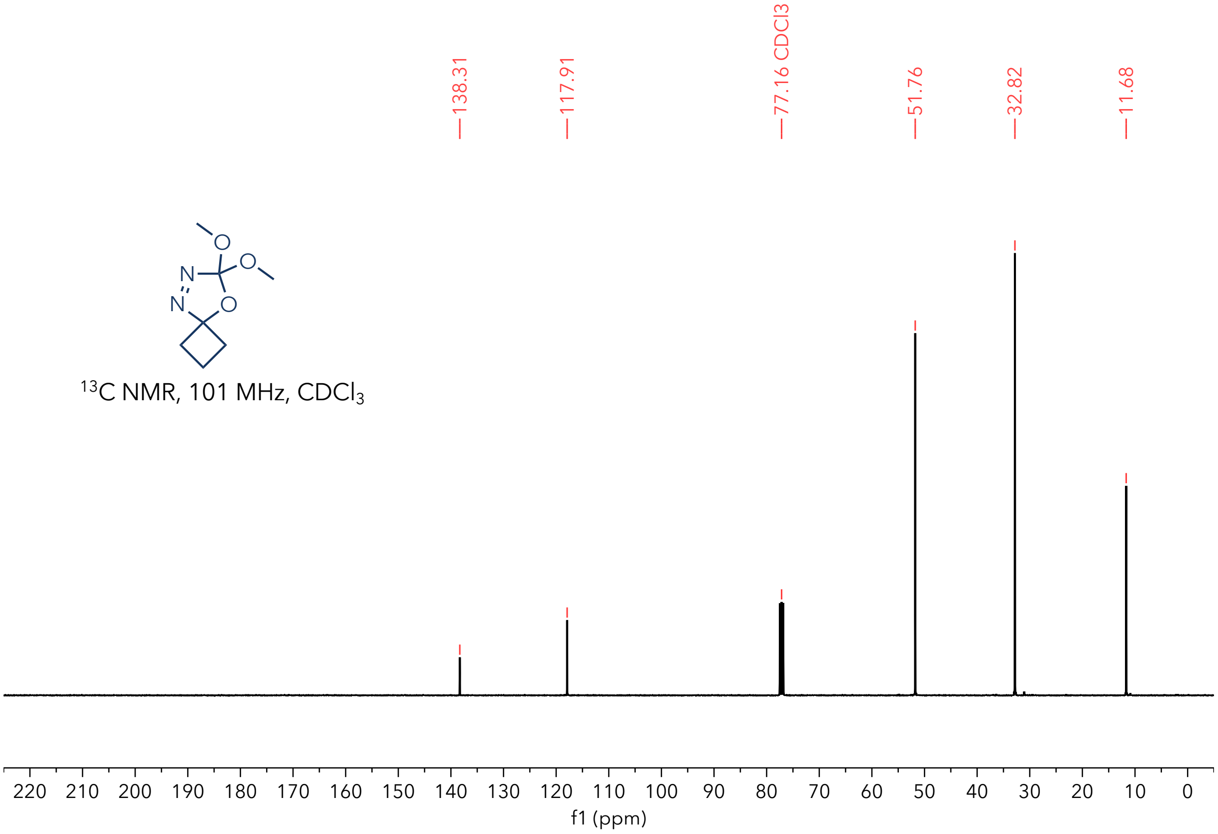
13C NMR, 101 MHz, CDCl3
f1 (ppm)

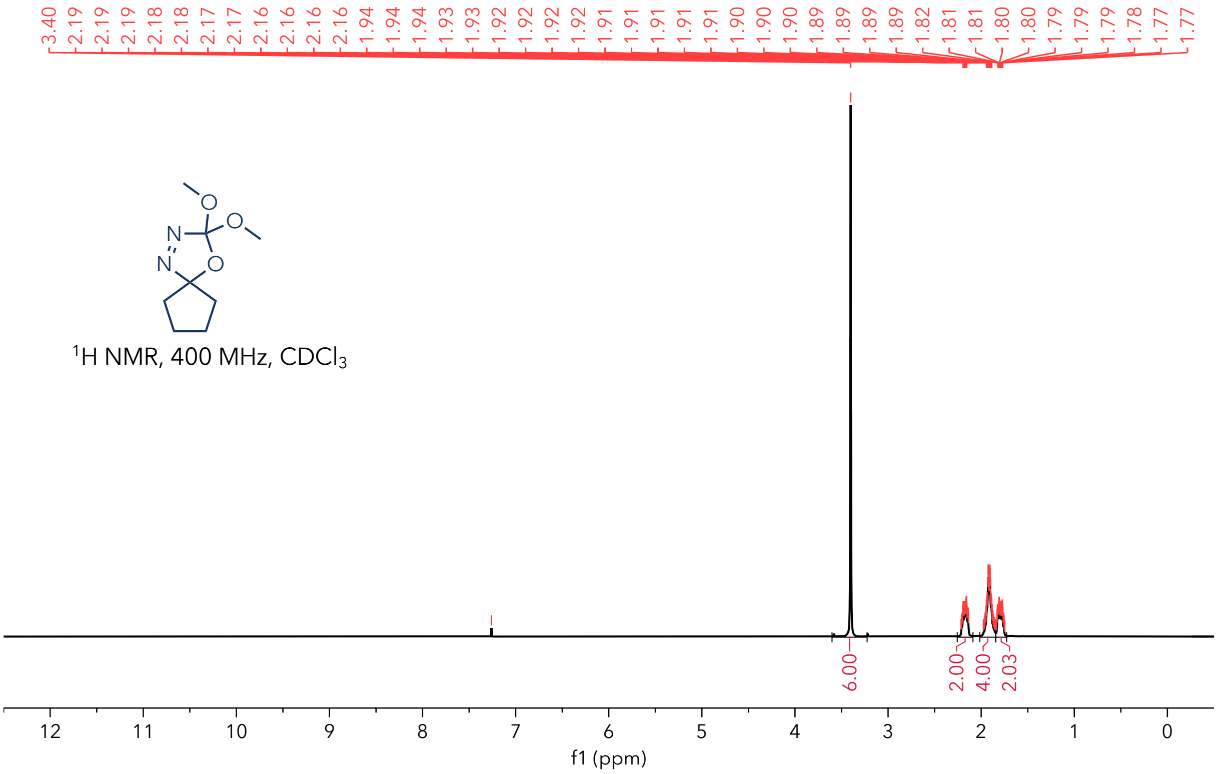

3.40
2.19
2.19
2.19
2.18
2.18
2.17
2.17
2.16
2.16
2.16
2.16
1.94
1.94
1.94
1.93
1.93
1.92
1.92
1.92
1.92
1.91
1.91
1.91
1.91
1.91
1.90
1.90
1.90
1.89
1.89
1.89
1.89
1.82
1.81
1.81
1.80
1.80
1.79
1.79
1.79
1.78
1.77
1.77
N
N
O
O
O
^{1}H NMR, 400 MHz, $CDCl_3$
6.00
2.00
4.00
2.03
f1 (ppm)

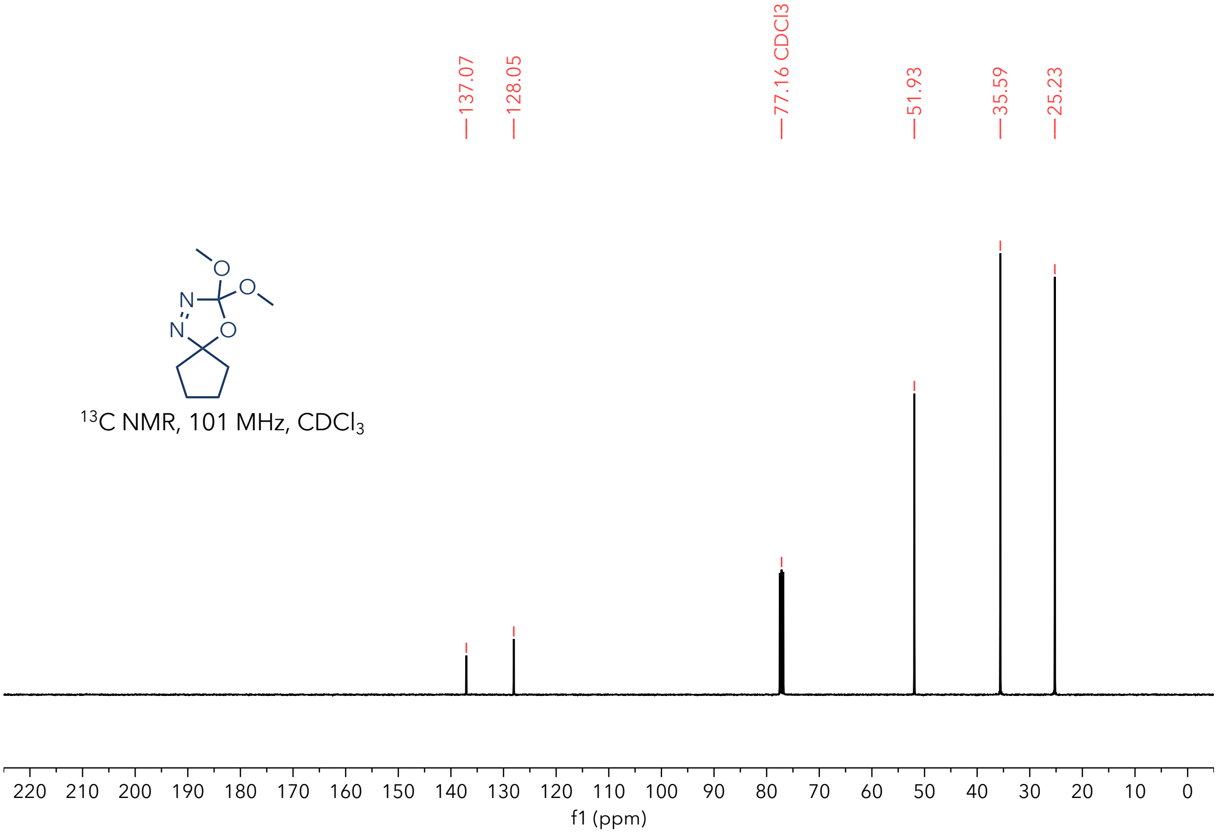

137.07
128.05
77.16 CDCl3
51.93
35.59
25.23
N
N
O
O
O
^{13}C NMR, 101 MHz, $CDCl_3$
f1 (ppm)

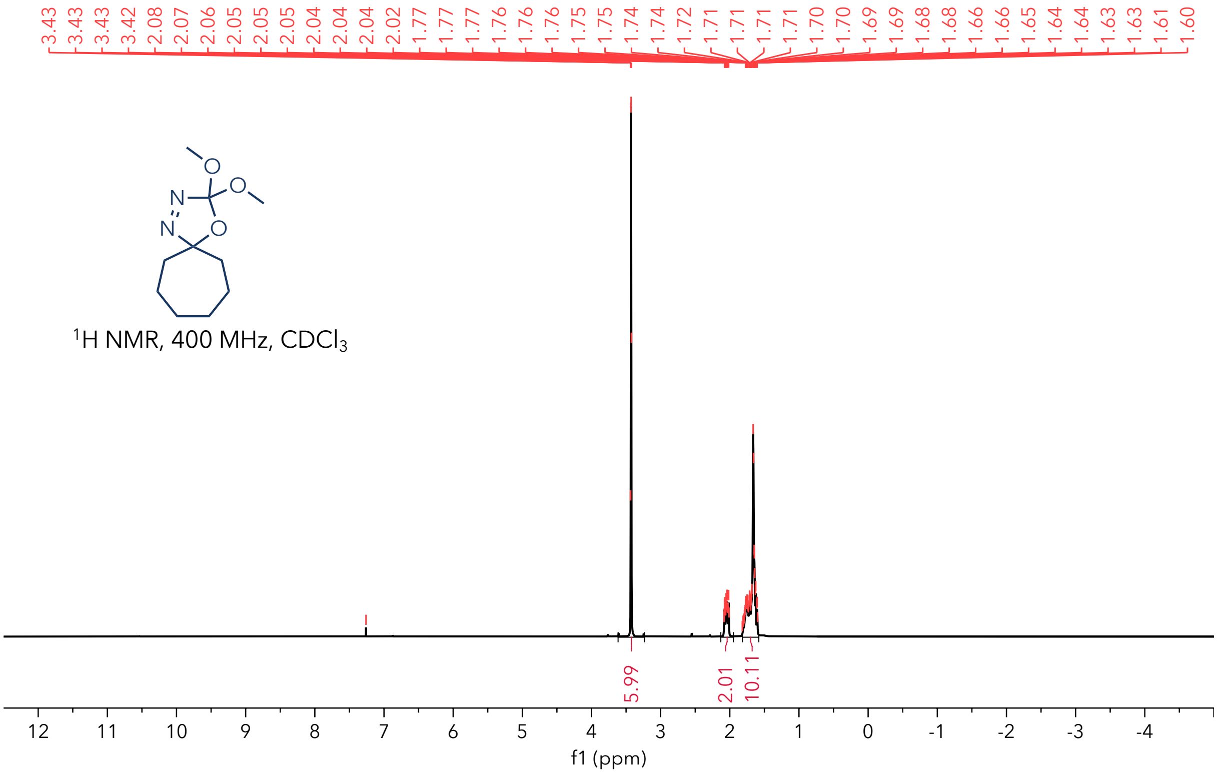

3.43 3.43 3.43 3.42 2.08 2.07 2.06 2.05 2.05 2.05 2.04 2.04 2.04 2.02 1.77 1.77 1.77 1.76 1.76 1.76 1.75 1.75 1.74 1.74 1.72 1.71 1.71 1.71 1.71 1.70 1.70 1.69 1.69 1.68 1.68 1.66 1.66 1.65 1.64 1.64 1.63 1.63 1.61 1.60
^{1}H NMR, 400 MHz, $CDCl_3$
5.99
2.01
10.11
12 11 10 9 8 7 6 5 4 3 2 1 0 -1 -2 -3 -4
f1 (ppm)

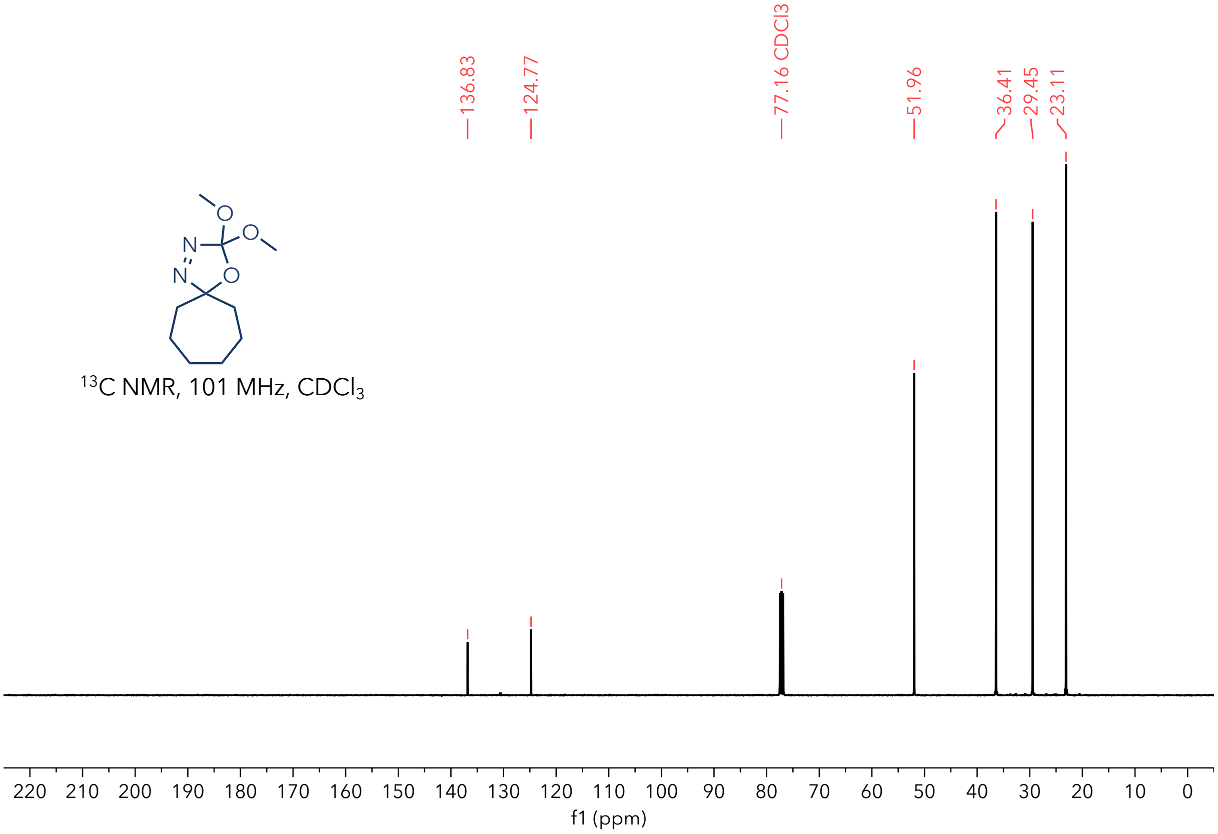

136.83
124.77
77.16 CDCl3
51.96
36.41
29.45
23.11
^{13}C NMR, 101 MHz, $CDCl_3$
220 210 200 190 180 170 160 150 140 130 120 110 100 90 80 70 60 50 40 30 20 10 0
f1 (ppm)

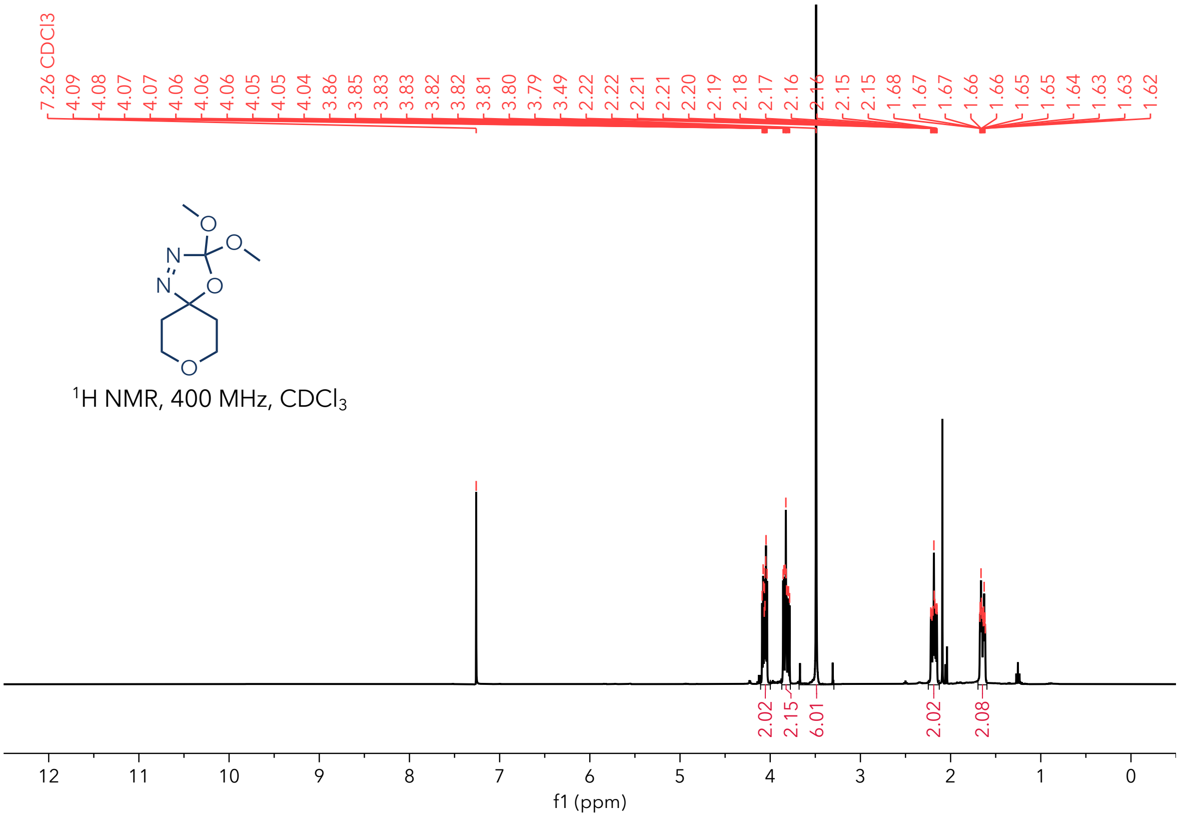
7.26 CDCl3
4.09
4.08
4.07
4.07
4.06
4.06
4.06
4.05
4.05
4.04
3.86
3.85
3.83
3.83
3.82
3.82
3.81
3.80
3.79
3.49
2.22
2.22
2.21
2.21
2.20
2.19
2.18
2.17
2.16
2.16
2.15
2.15
1.68
1.67
1.67
1.66
1.66
1.65
1.65
1.64
1.63
1.63
1.62
N
N
O
O
O
O
1H NMR, 400 MHz, CDCl3
2.02
2.15
6.01
2.02
2.08
12
11
10
9
8
7
6
5
4
3
2
1
0
f1 (ppm)

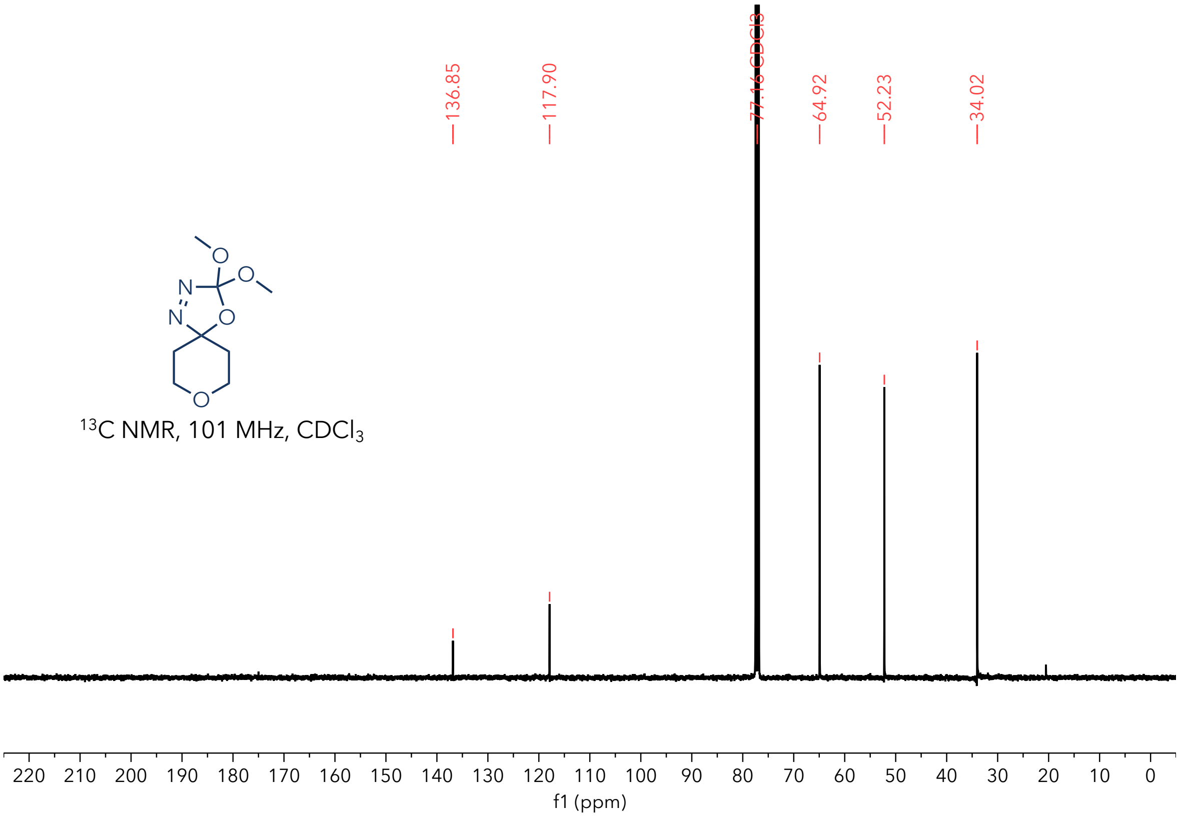
136.85
117.90
77.16 CDCl3
64.92
52.23
34.02
N
N
O
O
O
O
13C NMR, 101 MHz, CDCl3
220
210
200
190
180
170
160
150
140
130
120
110
100
90
80
70
60
50
40
30
20
10
0
f1 (ppm)

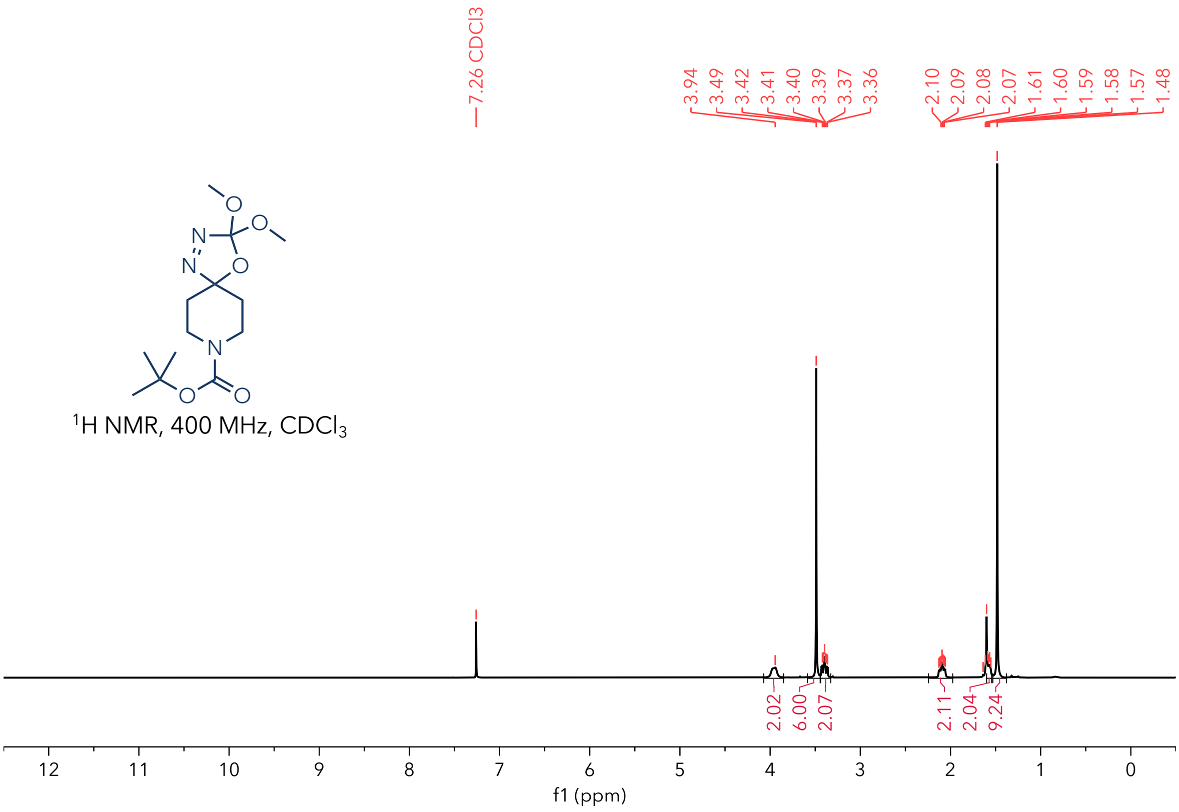

^{1}H NMR, 400 MHz, $CDCl_3$

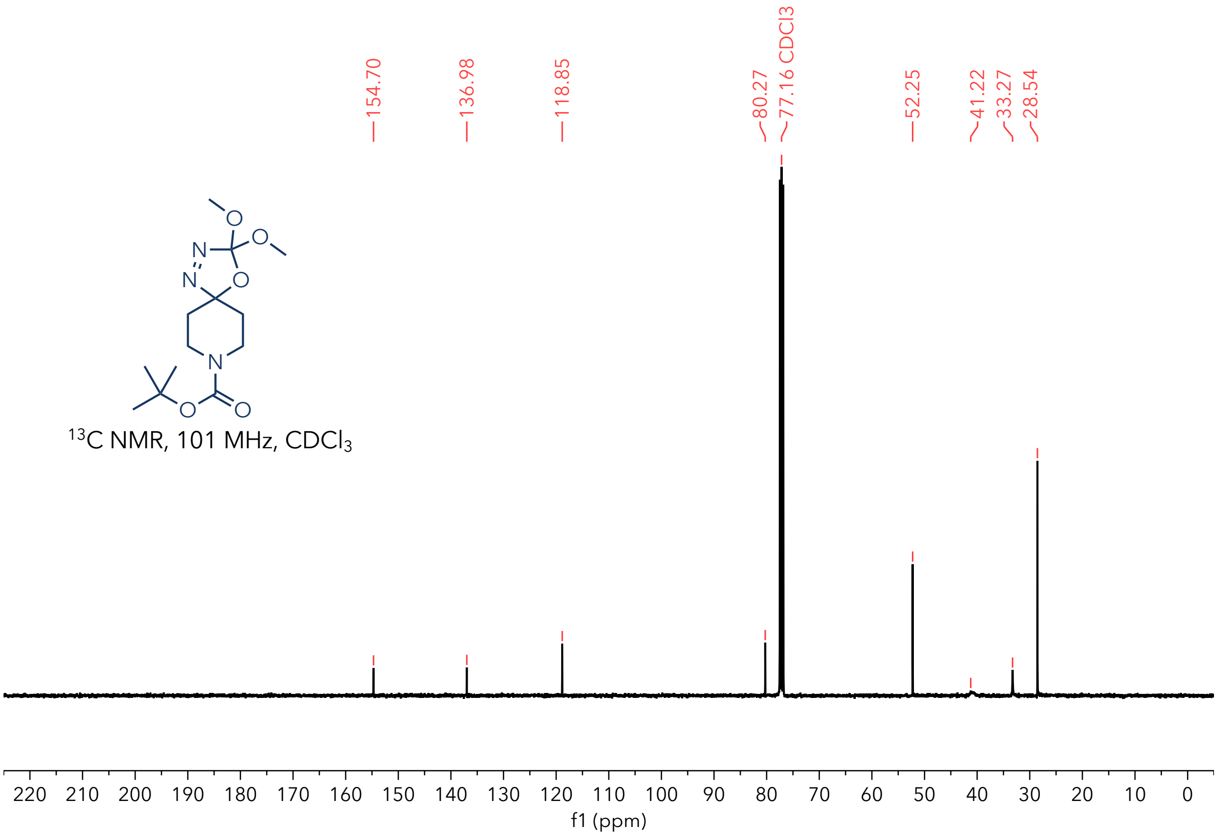

^{13}C NMR, 101 MHz, $CDCl_3$

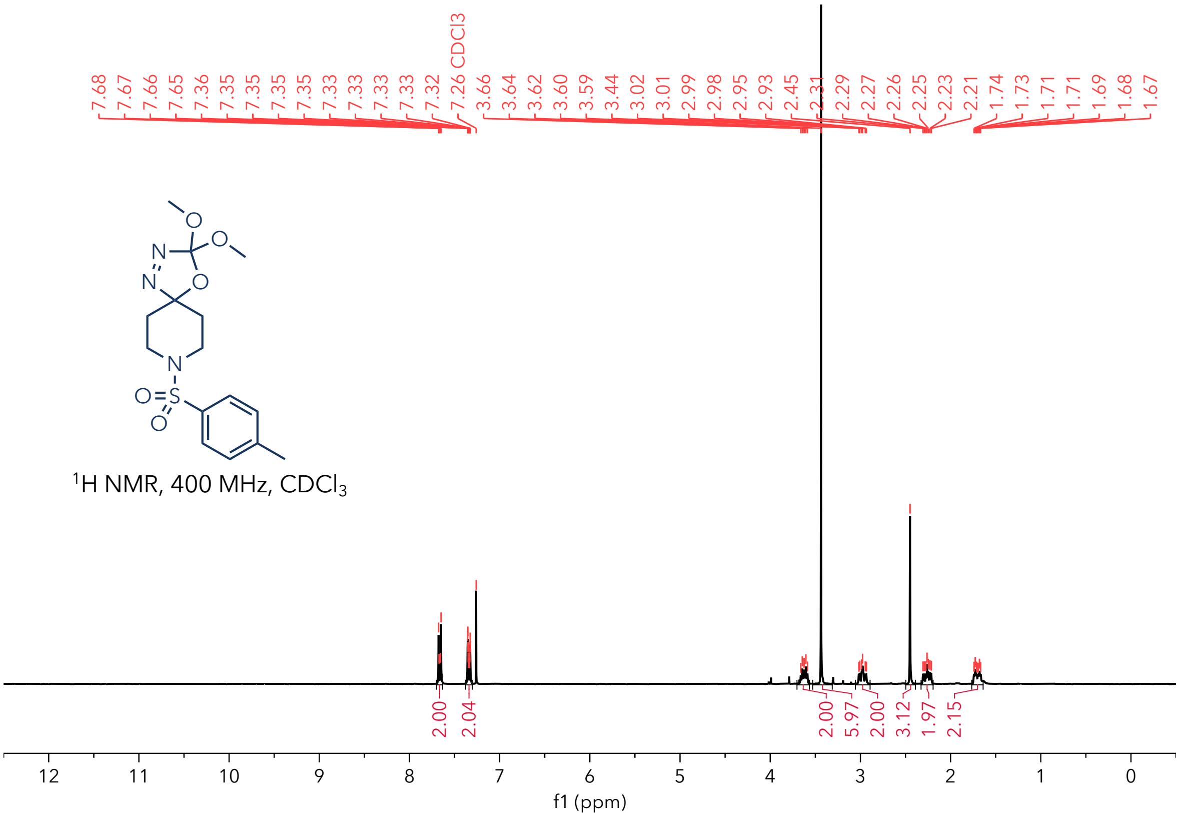
7.68 7.67 7.66 7.65 7.36 7.35 7.35 7.35 7.35 7.33 7.33 7.33 7.33 7.32 7.26 CDCl3 3.66 3.64 3.62 3.60 3.59 3.44 3.02 3.01 2.99 2.98 2.95 2.93 2.45 2.31 2.29 2.27 2.26 2.25 2.23 2.21 1.74 1.73 1.71 1.71 1.69 1.68 1.67
^{1}H NMR, 400 MHz, $CDCl_3$
2.00 2.04 2.00 5.97 2.00 3.12 1.97 2.15
f1 (ppm)

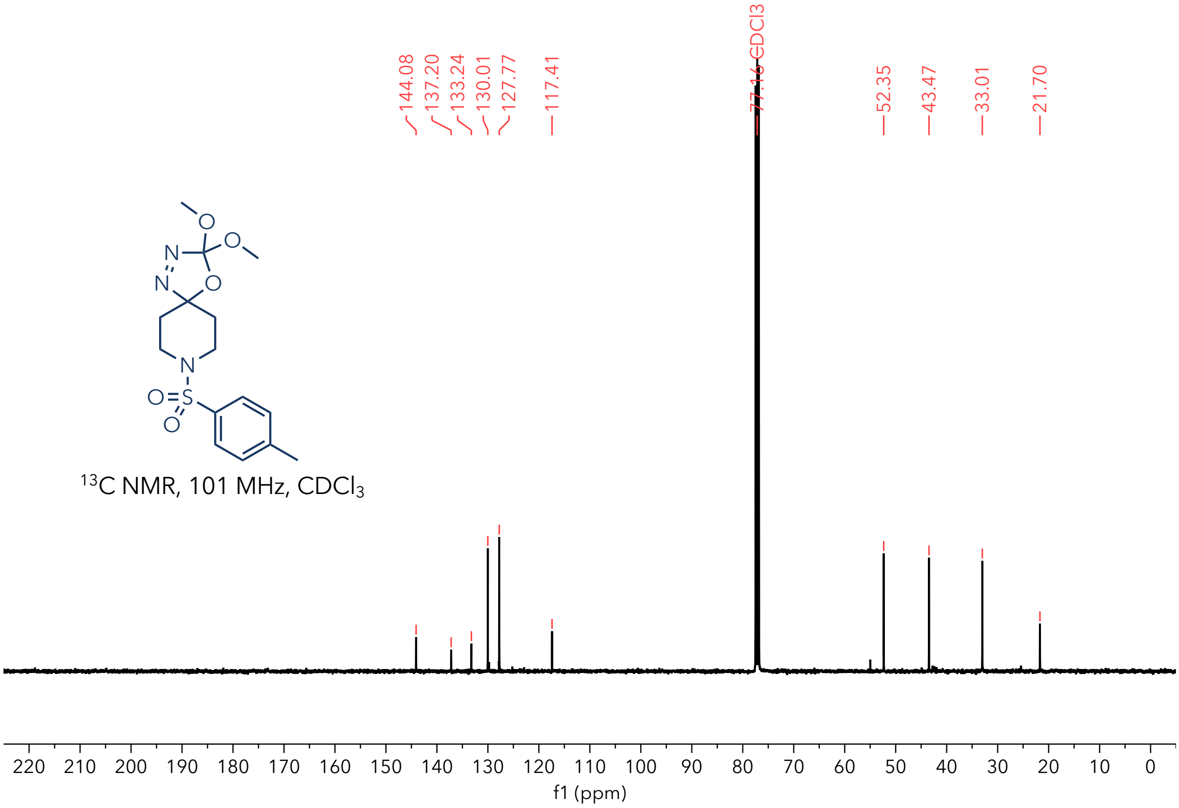
144.08 137.20 133.24 130.01 127.77 117.41 77.16 CDCl3 52.35 43.47 33.01 21.70
^{13}C NMR, 101 MHz, $CDCl_3$
f1 (ppm)

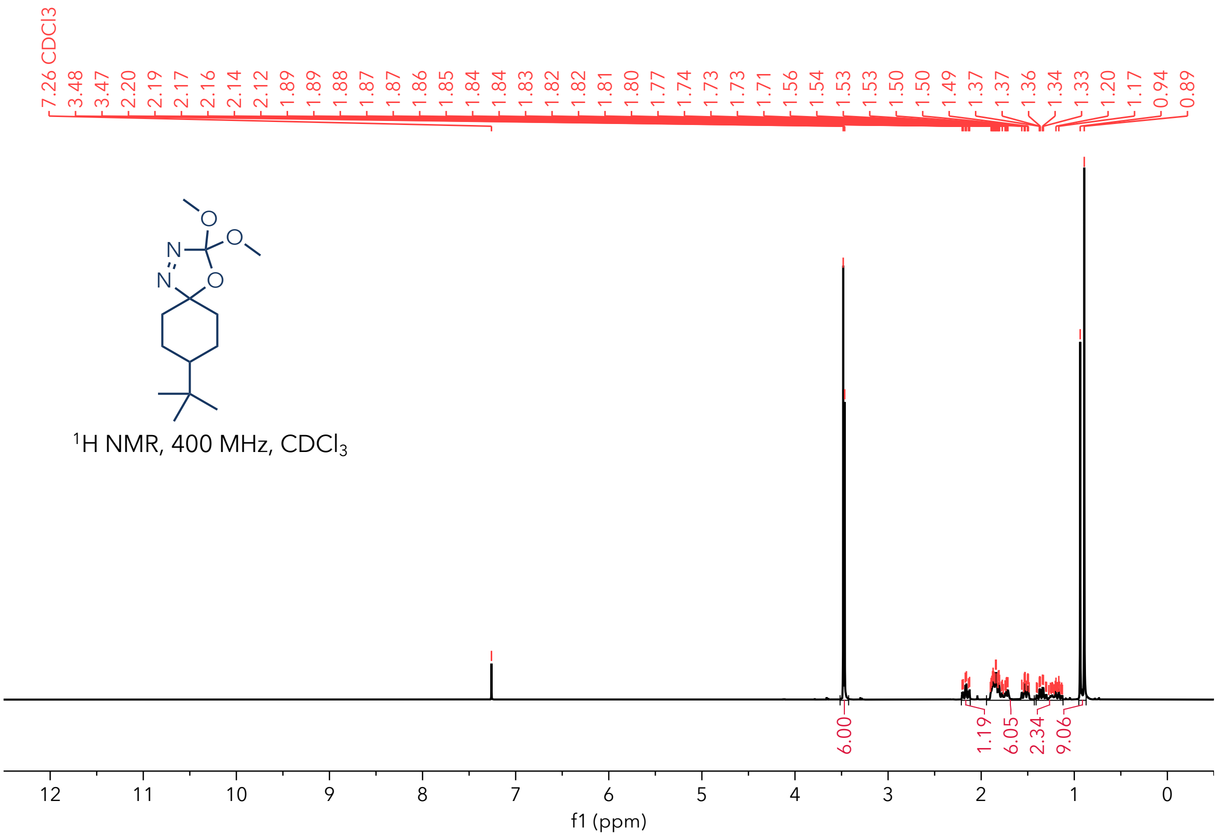
7.26 CDCl3
3.48
3.47
2.20
2.19
2.17
2.16
2.14
2.12
1.89
1.89
1.88
1.87
1.87
1.86
1.85
1.84
1.84
1.83
1.82
1.82
1.81
1.80
1.77
1.74
1.73
1.73
1.71
1.56
1.54
1.53
1.53
1.50
1.50
1.49
1.37
1.37
1.36
1.34
1.33
1.20
1.17
0.94
0.89
N
N
O
O
O
^{1}H NMR, 400 MHz, $CDCl_3$
6.00
1.19
6.05
2.34
9.06
12
11
10
9
8
7
6
5
4
3
2
1
0
f1 (ppm)

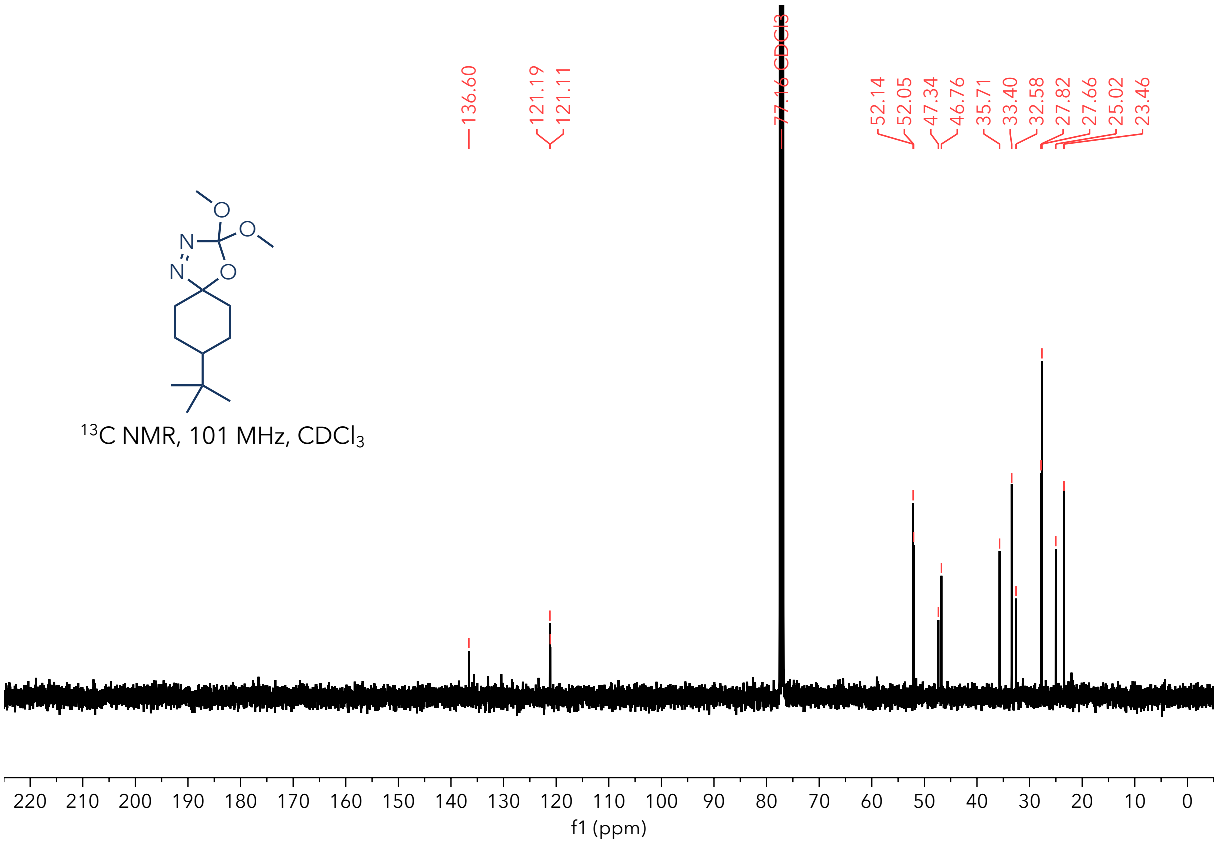
136.60
121.19
121.11
77.16 CDCl3
52.14
52.05
47.34
46.76
35.71
33.40
32.58
27.82
27.66
25.02
23.46
N
N
O
O
O
^{13}C NMR, 101 MHz, $CDCl_3$
220
210
200
190
180
170
160
150
140
130
120
110
100
90
80
70
60
50
40
30
20
10
0
f1 (ppm)

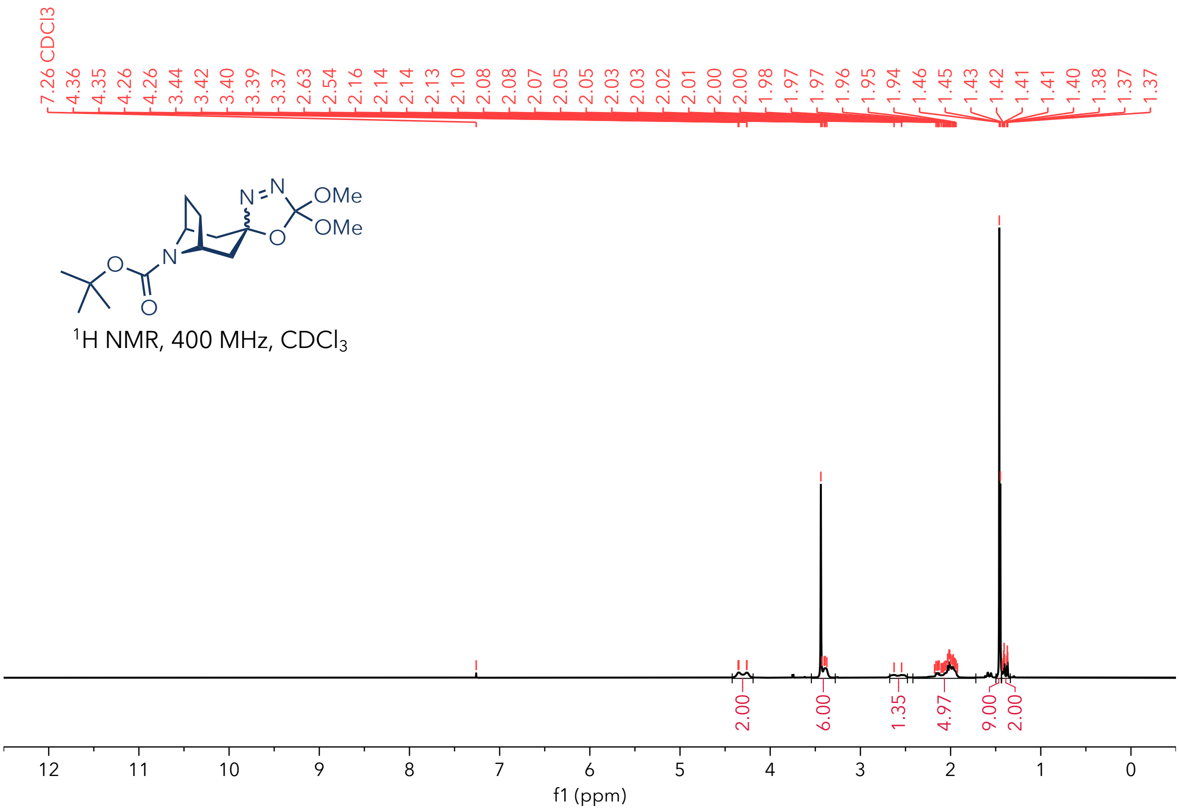

7.26 CDCl3
4.36
4.35
4.26
4.26
3.44
3.42
3.40
3.39
3.37
2.63
2.54
2.16
2.14
2.14
2.13
2.10
2.08
2.08
2.07
2.05
2.05
2.03
2.03
2.02
2.01
2.00
2.00
1.98
1.97
1.97
1.96
1.95
1.94
1.46
1.45
1.43
1.42
1.41
1.41
1.40
1.38
1.37
1.37
OMe
OMe
N
O
O
O
^{1}H NMR, 400 MHz, $CDCl_3$
2.00
6.00
1.35
4.97
9.00
2.00
12
11
10
9
8
7
6
5
4
3
2
1
0
f1 (ppm)

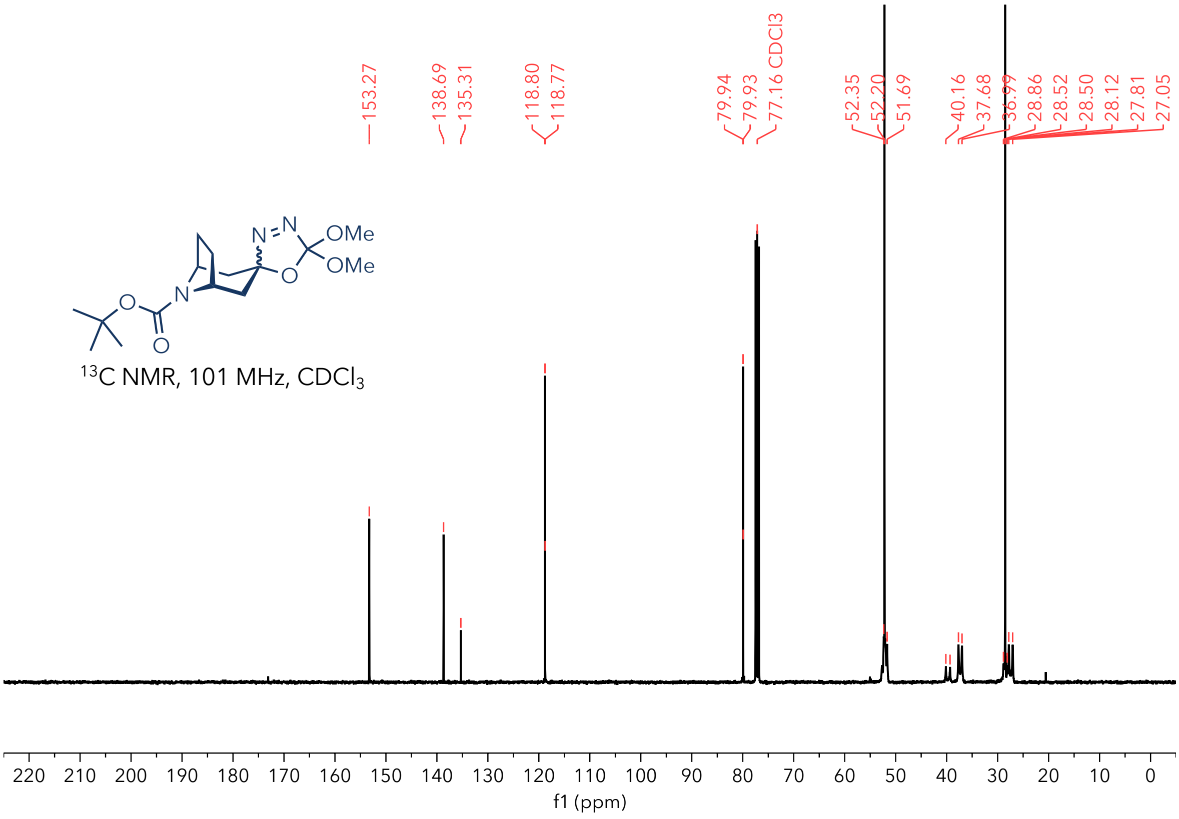

153.27
138.69
135.31
118.80
118.77
79.94
79.93
77.16 CDCl3
52.35
52.20
51.69
40.16
37.68
28.86
28.52
28.50
28.12
27.81
27.05
OMe
OMe
N
O
O
O
^{13}C NMR, 101 MHz, $CDCl_3$
220
210
200
190
180
170
160
150
140
130
120
110
100
90
80
70
60
50
40
30
20
10
0
f1 (ppm)

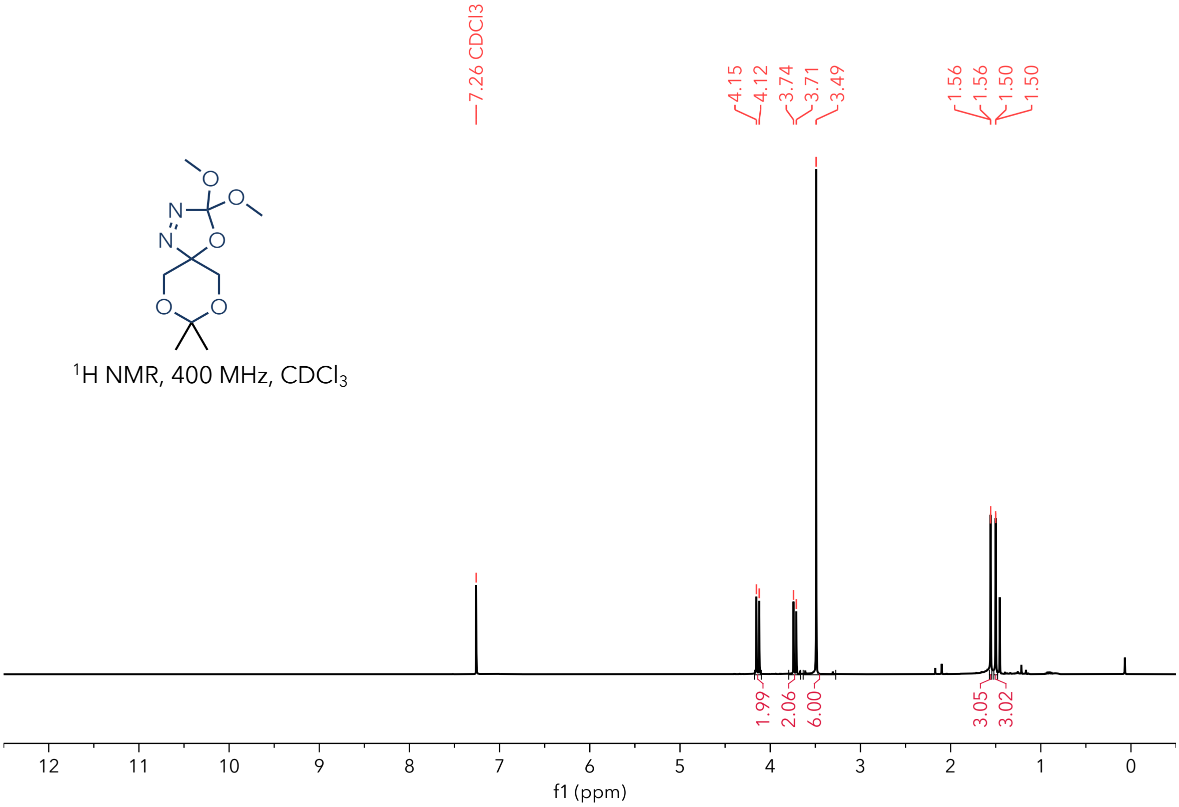

7.26 CDCl3
4.15
4.12
3.74
3.71
3.49
1.56
1.56
1.50
1.50
1H NMR, 400 MHz, CDCl3
1.99
2.06
6.00
3.05
3.02
12 11 10 9 8 7 6 5 4 3 2 1 0
f1 (ppm)

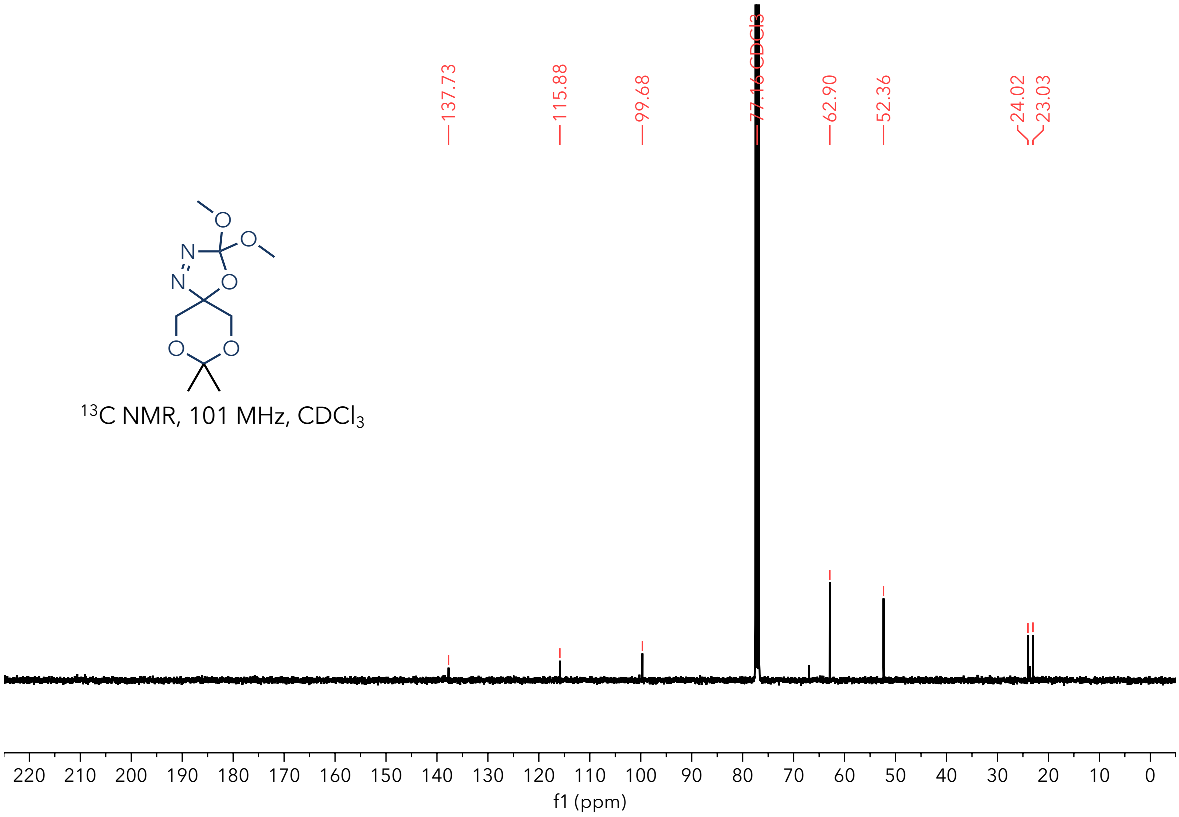

137.73
115.88
99.68
77.16 CDCl3
62.90
52.36
24.02
23.03
13C NMR, 101 MHz, CDCl3
220 210 200 190 180 170 160 150 140 130 120 110 100 90 80 70 60 50 40 30 20 10 0
f1 (ppm)

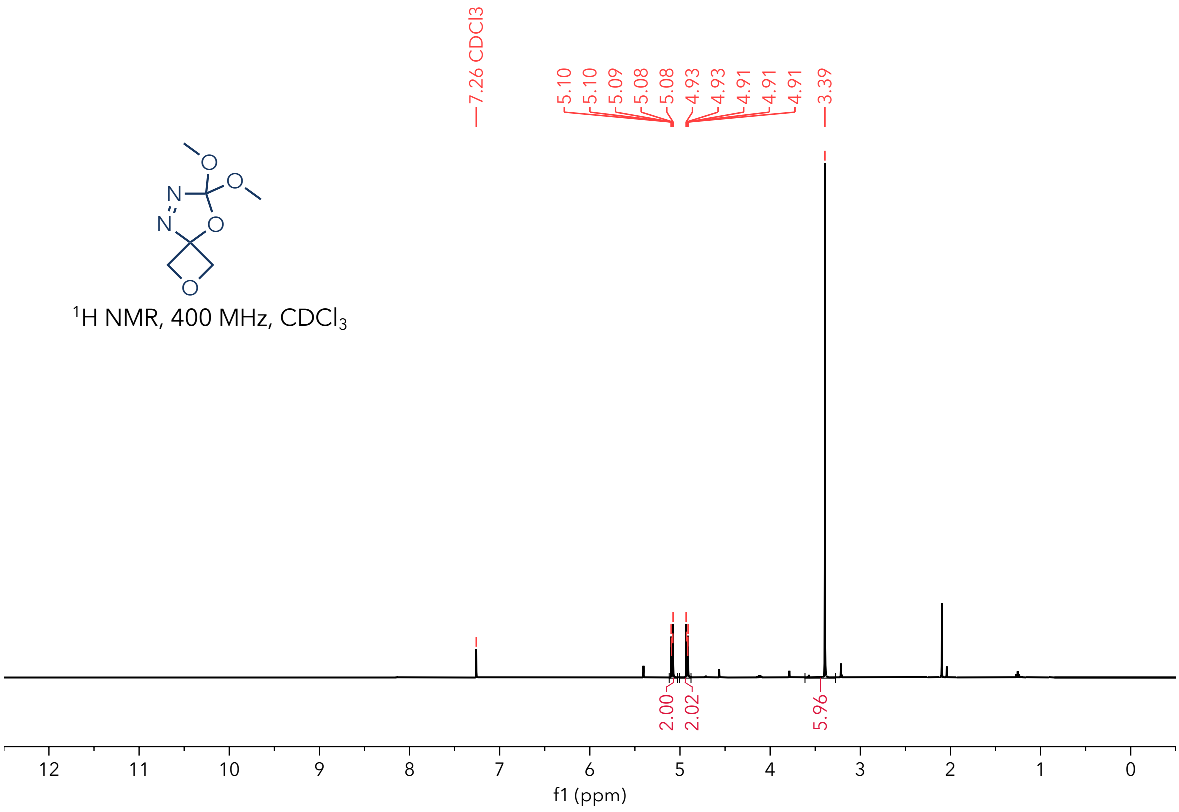

^{1}H NMR, 400 MHz, $CDCl_3$

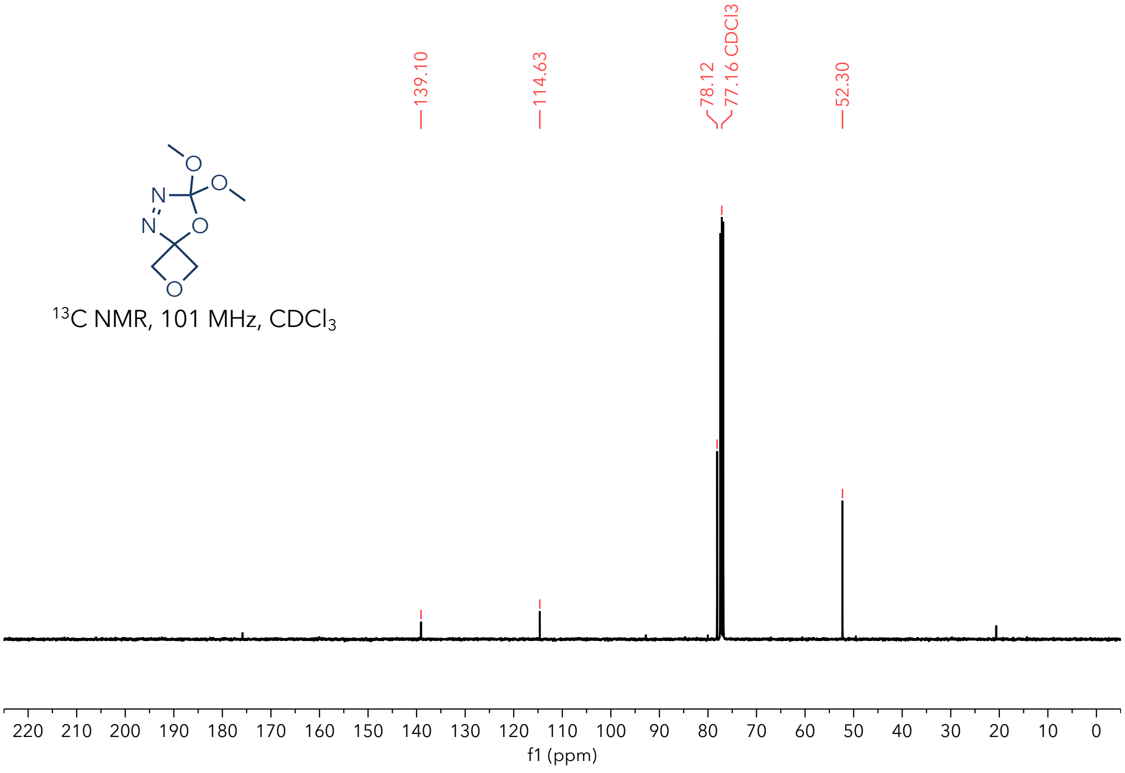

^{13}C NMR, 101 MHz, $CDCl_3$

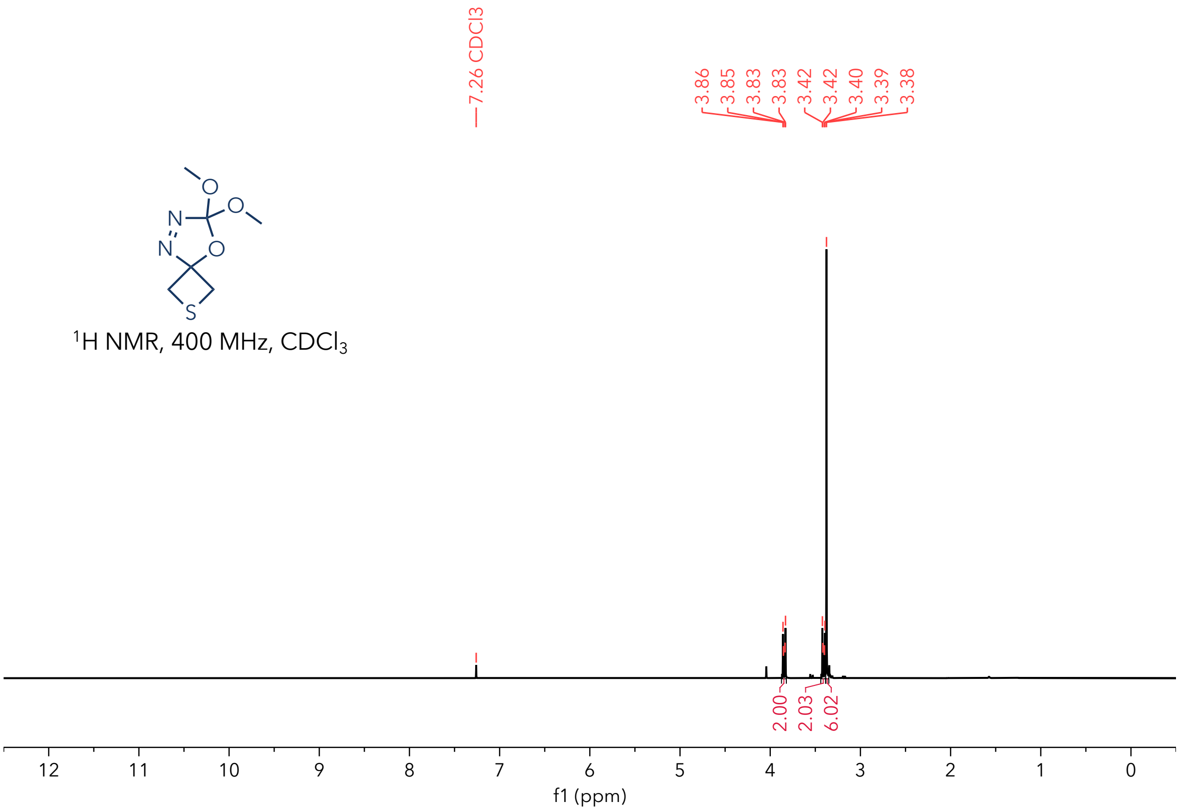

^{1}H NMR, 400 MHz, $CDCl_3$

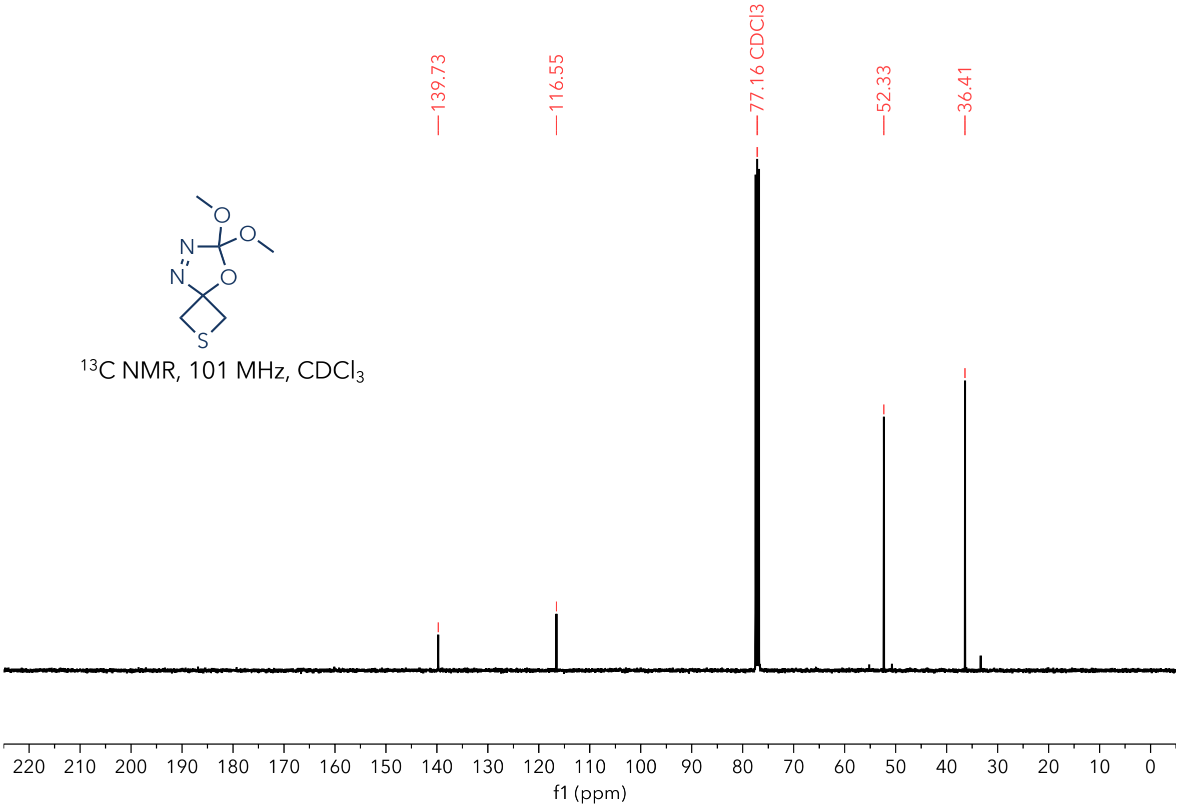

^{13}C NMR, 101 MHz, $CDCl_3$

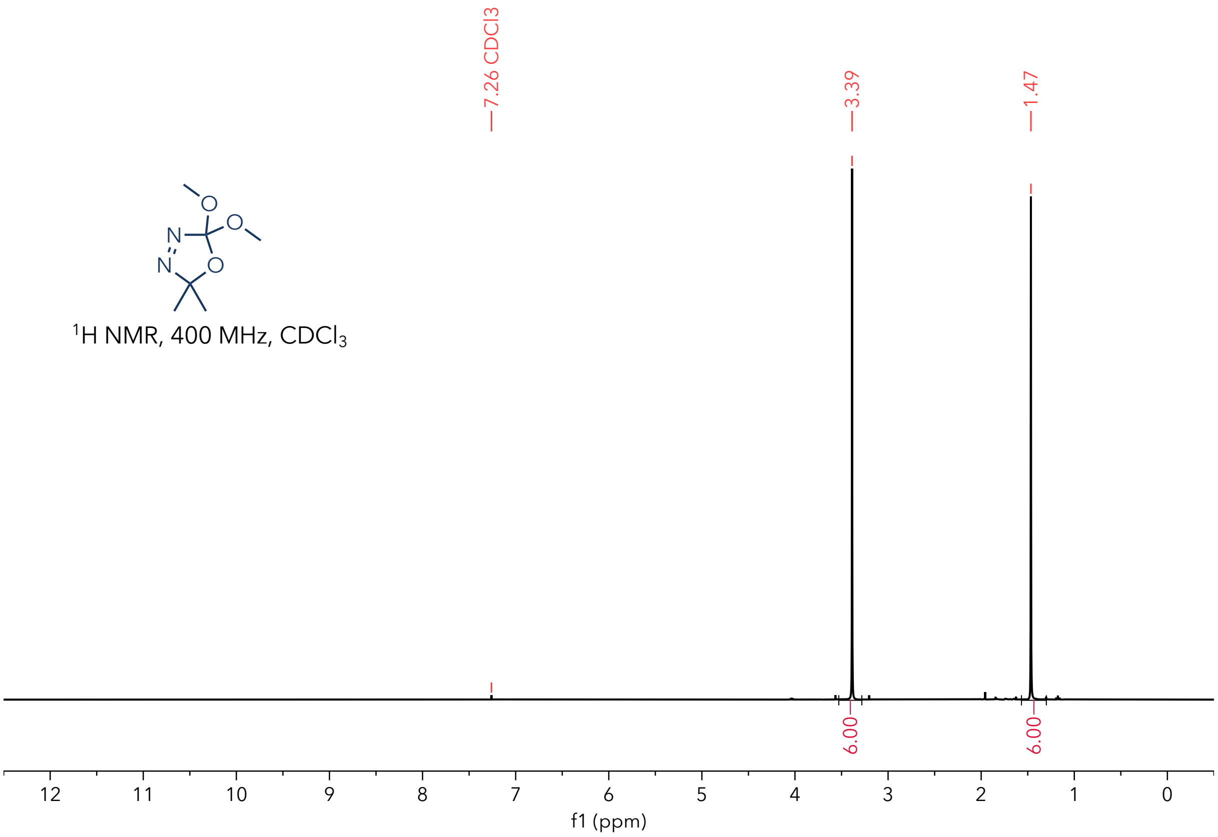

^{1}H NMR, 400 MHz, $CDCl_3$

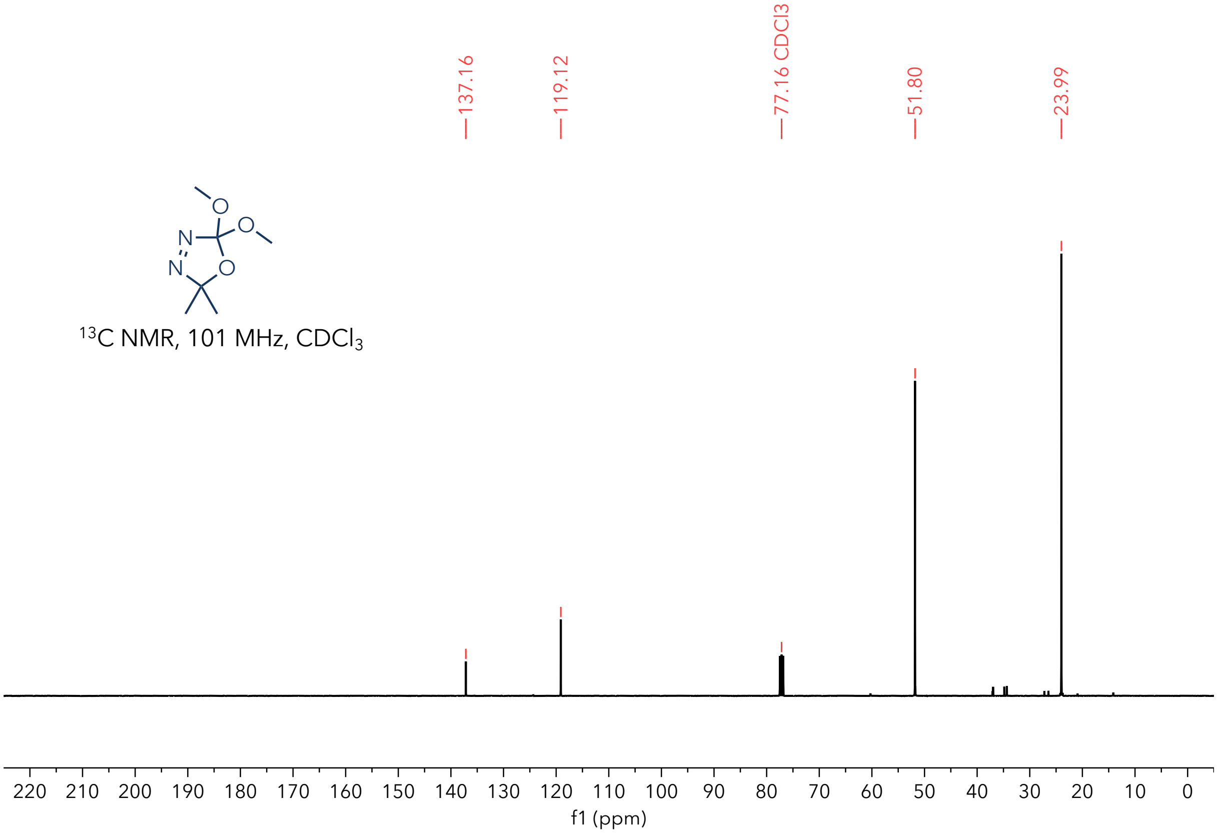

^{13}C NMR, 101 MHz, $CDCl_3$

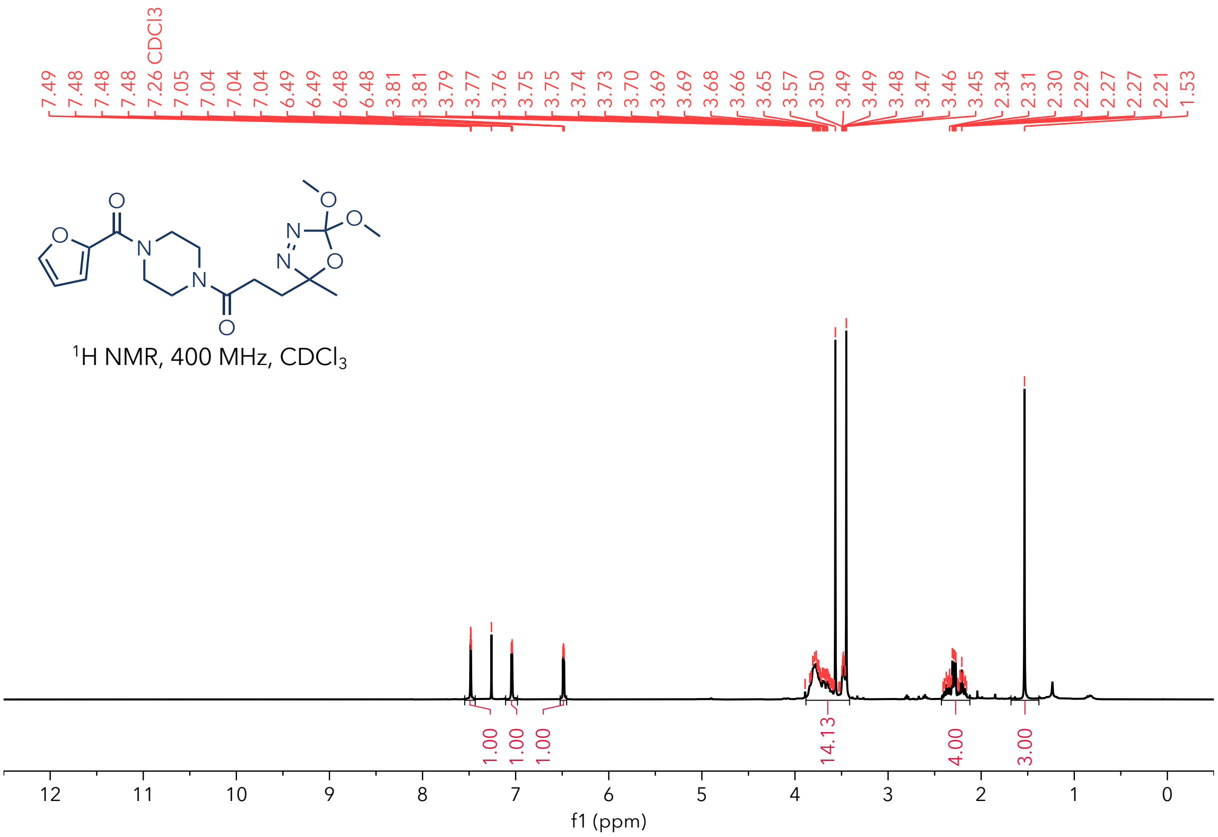
7.49 7.48 7.48 7.48 7.26 CDCl3 7.05 7.04 7.04 7.04 6.49 6.49 6.48 6.48 3.81 3.81 3.79 3.77 3.76 3.75 3.75 3.74 3.73 3.70 3.69 3.69 3.68 3.66 3.65 3.57 3.50 3.49 3.49 3.48 3.47 3.46 3.45 2.34 2.31 2.30 2.29 2.27 2.27 2.21 1.53
O
N
N
O
N
N
O
O
O
O
^{1}H NMR, 400 MHz, $CDCl_3$
1.00 1.00 1.00 14.13 4.00 3.00
12 11 10 9 8 7 6 5 4 3 2 1 0
f1 (ppm)

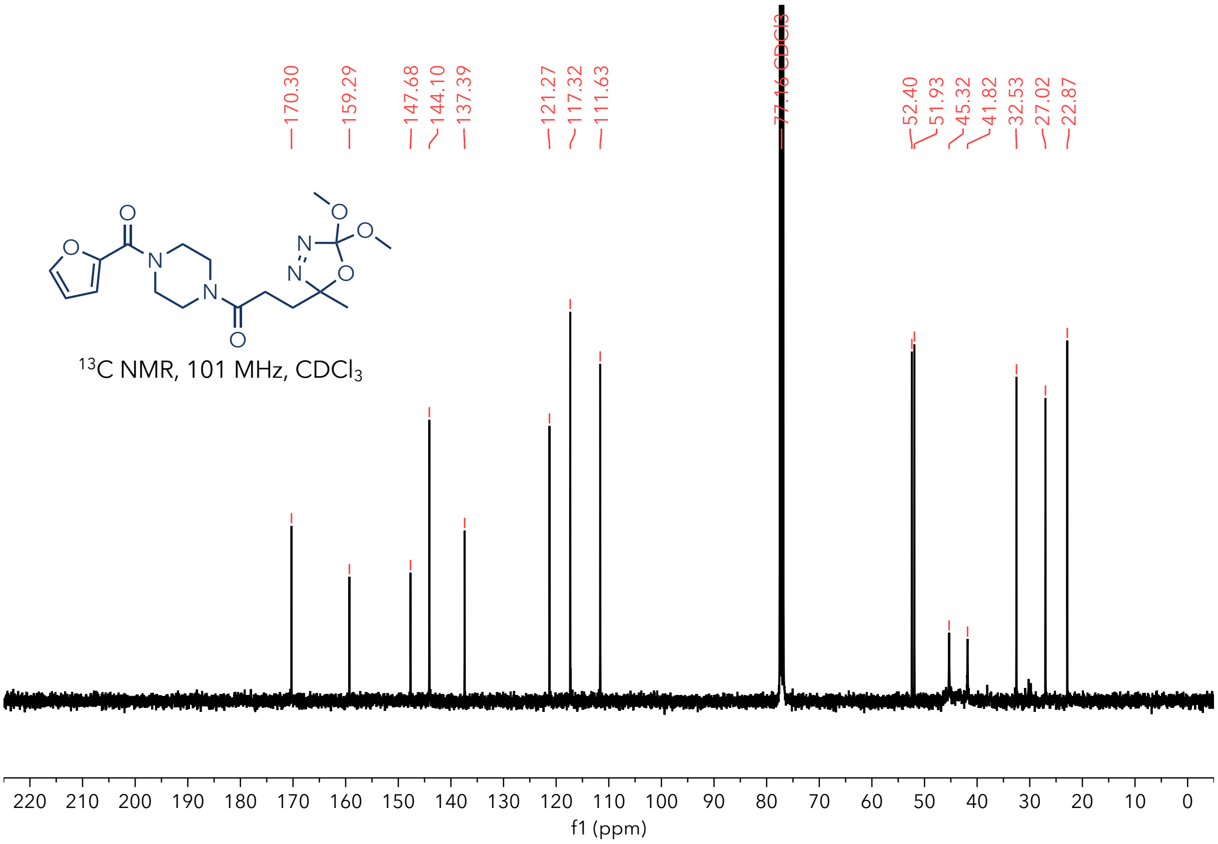
170.30 159.29 147.68 144.10 137.39 121.27 117.32 111.63 77.16 CDCl3 52.40 51.93 45.32 41.82 32.53 27.02 22.87
O
N
N
O
N
N
O
O
O
O
^{13}C NMR, 101 MHz, $CDCl_3$
220 210 200 190 180 170 160 150 140 130 120 110 100 90 80 70 60 50 40 30 20 10 0
f1 (ppm)

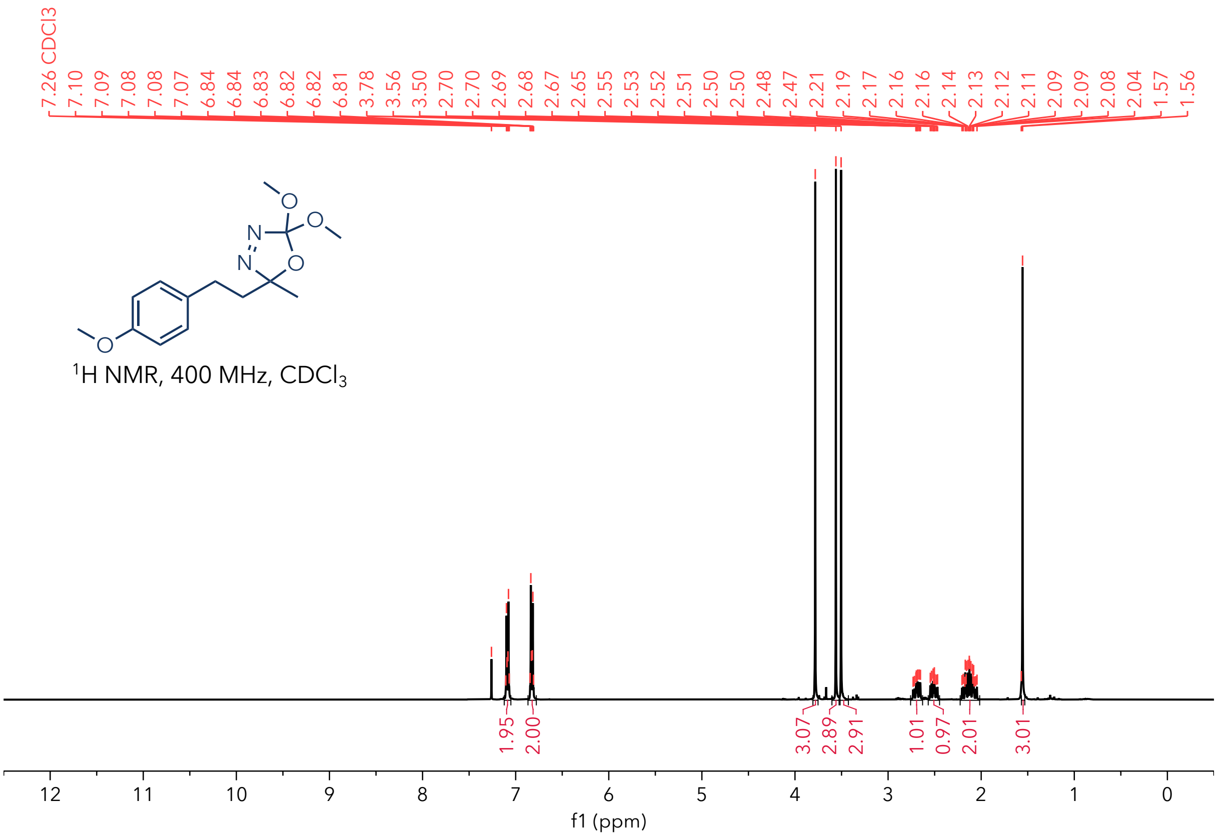

^{1}H NMR, 400 MHz, $CDCl_3$

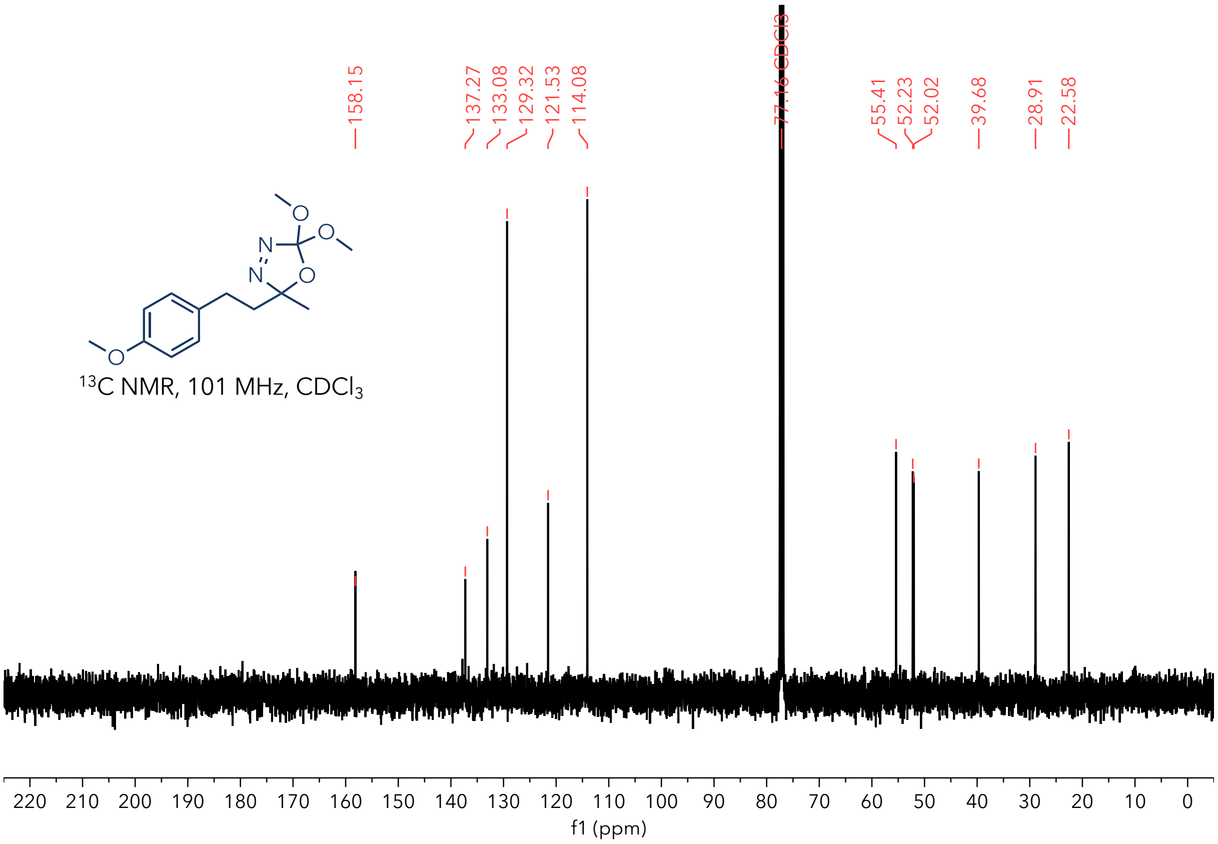

^{13}C NMR, 101 MHz, $CDCl_3$

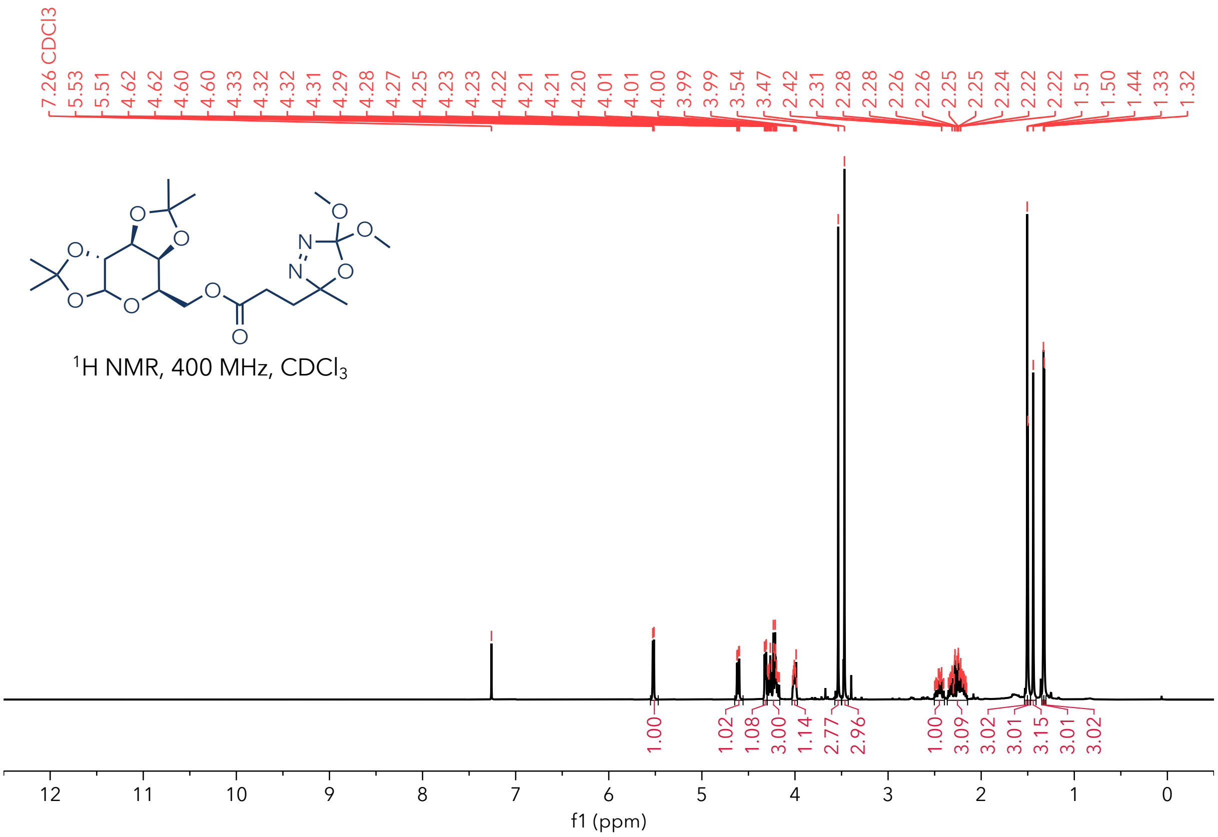
1H NMR, 400 MHz, CDCl3
f1 (ppm)

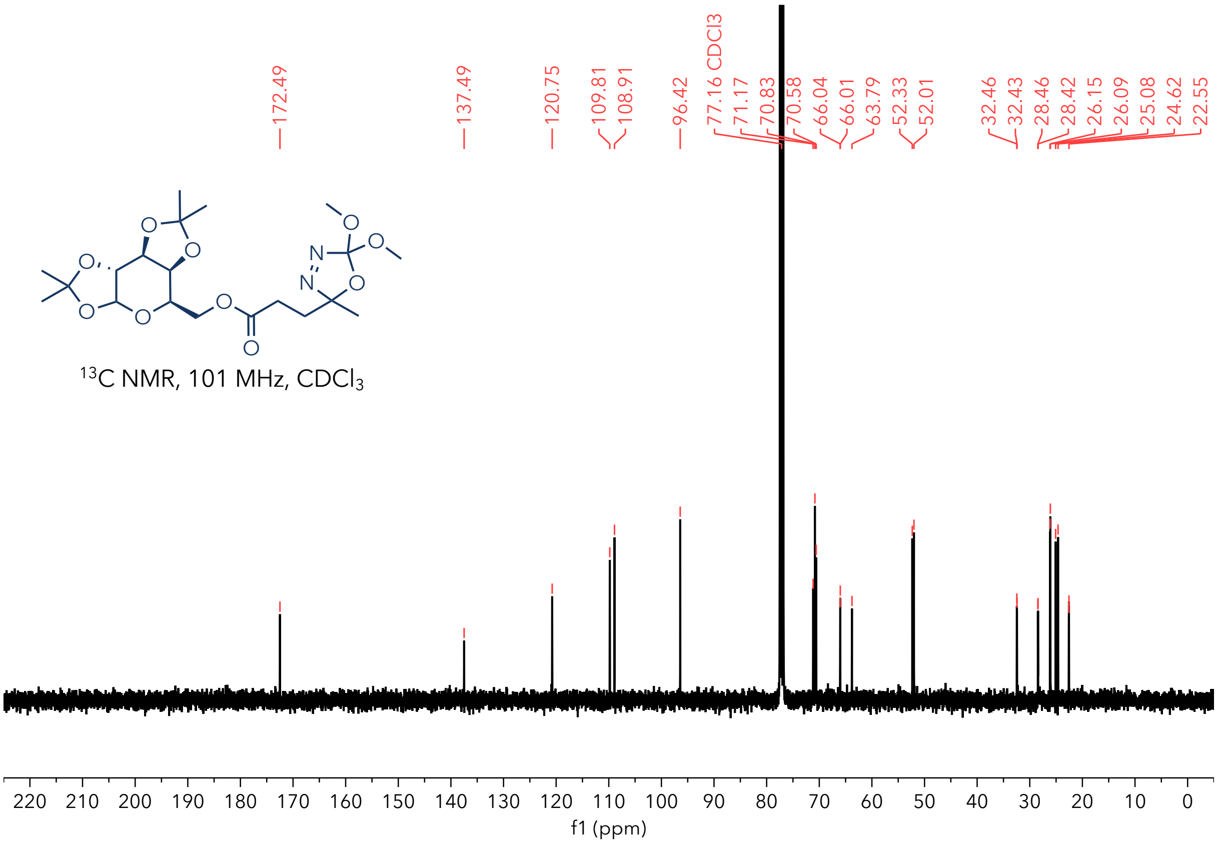
13C NMR, 101 MHz, CDCl3
f1 (ppm)

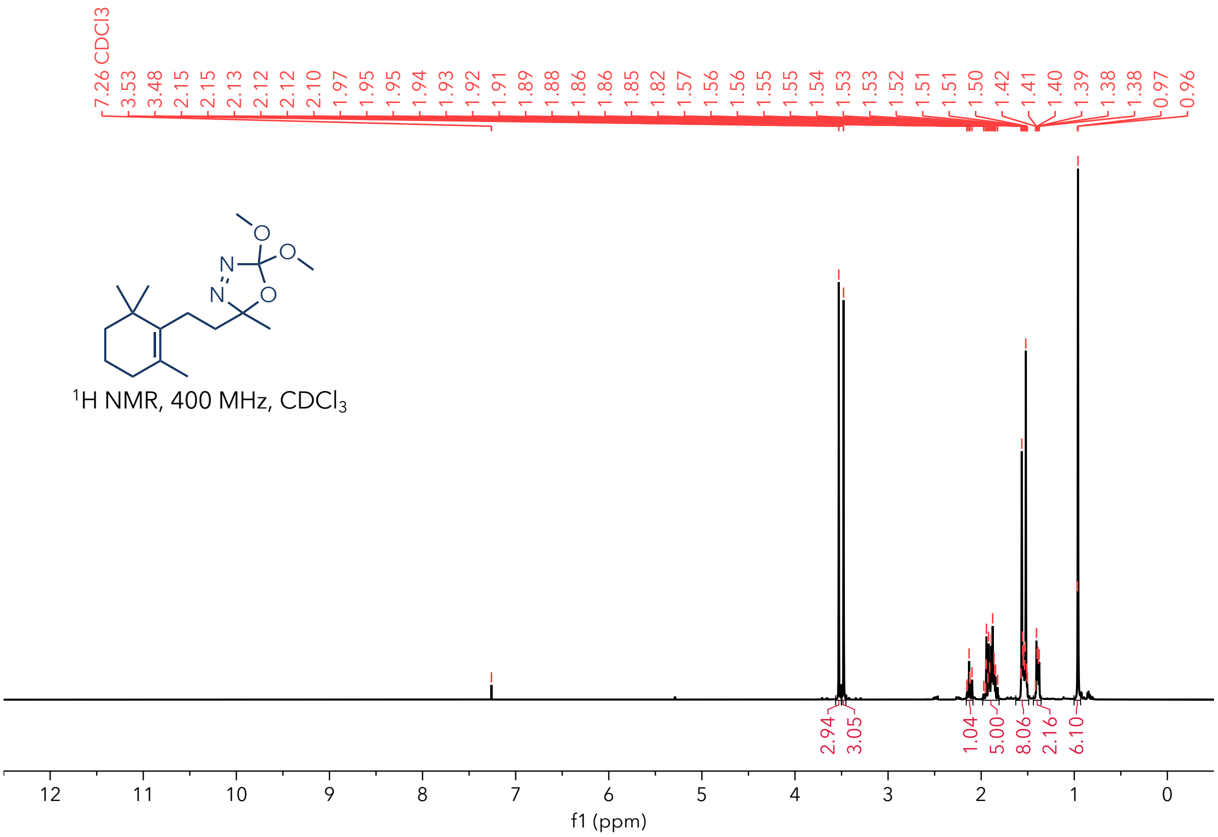

^{1}H NMR, 400 MHz, $CDCl_3$

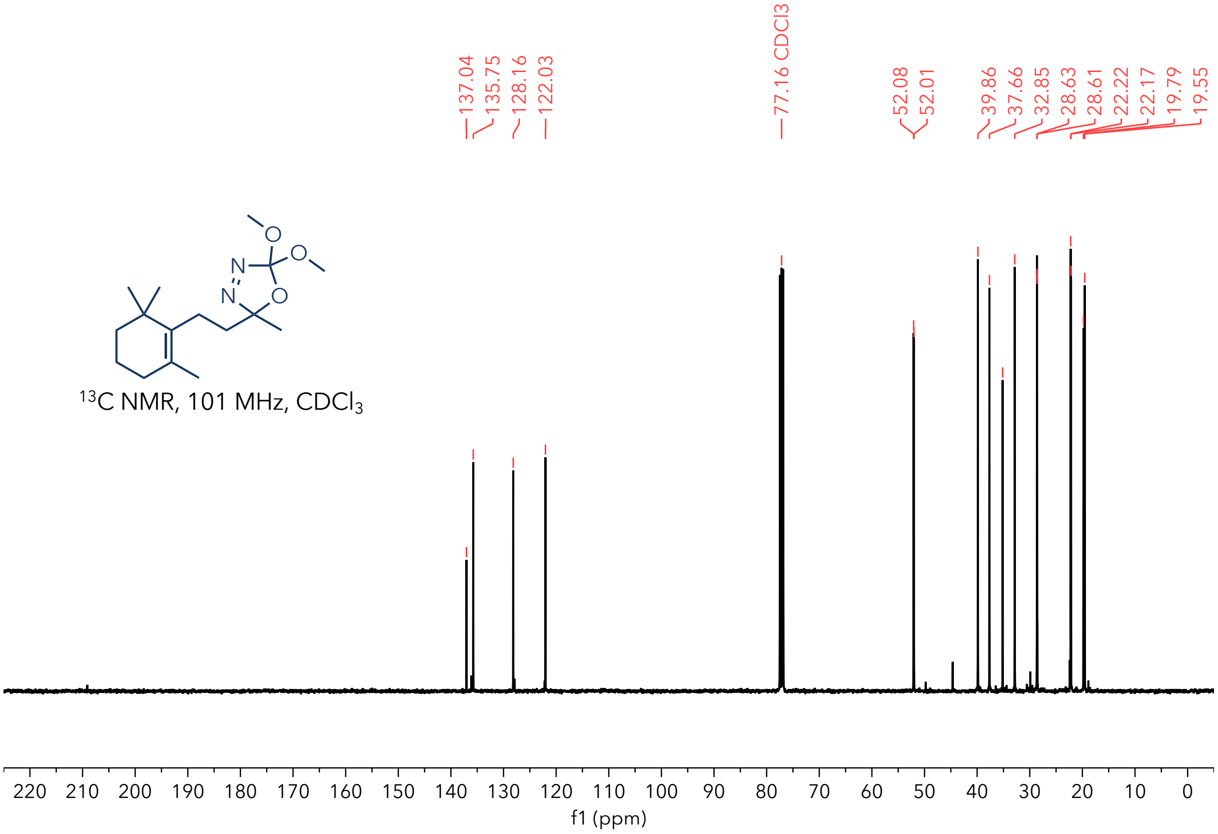

^{13}C NMR, 101 MHz, $CDCl_3$

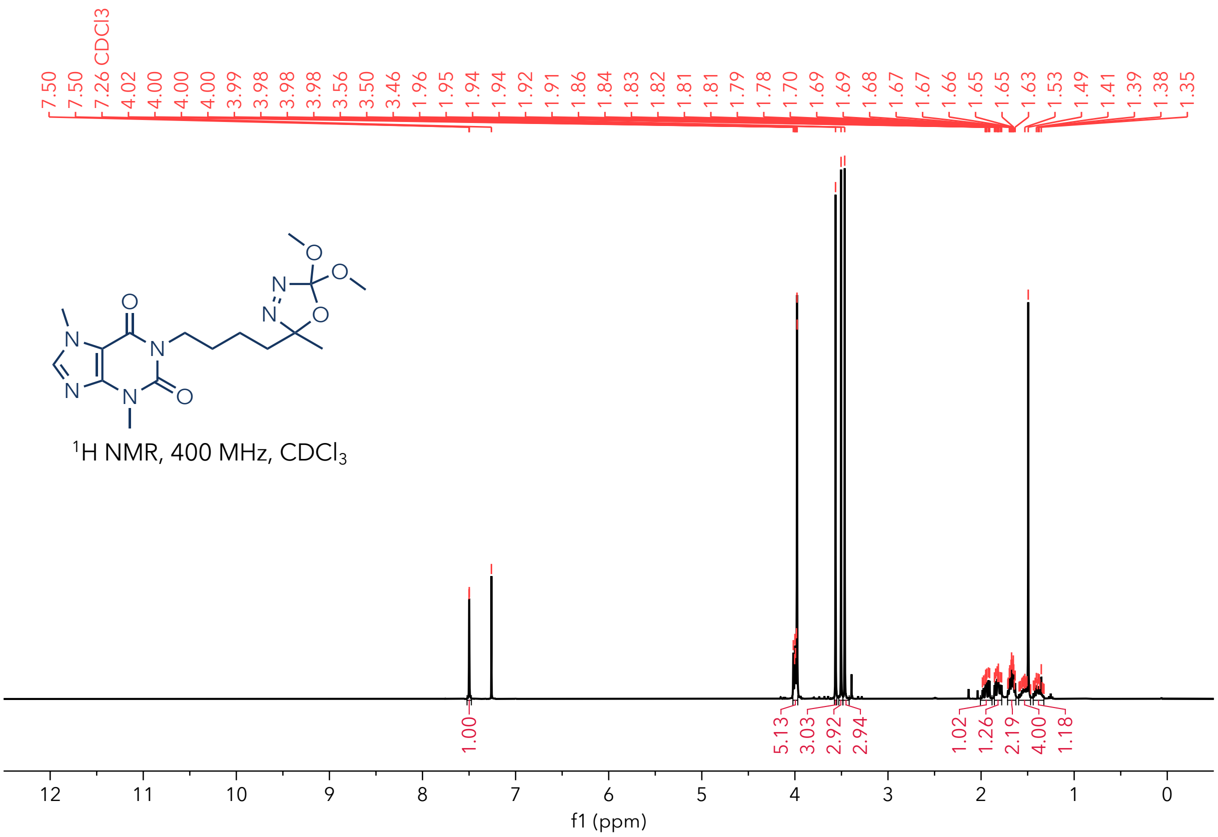
7.50
7.50
7.26 CDCl3
4.02
4.00
4.00
4.00
3.99
3.98
3.98
3.98
3.56
3.50
3.46
1.96
1.95
1.94
1.94
1.92
1.91
1.86
1.84
1.83
1.82
1.81
1.81
1.79
1.78
1.70
1.69
1.69
1.68
1.67
1.67
1.66
1.65
1.65
1.63
1.53
1.49
1.41
1.39
1.38
1.35
^{1}H NMR, 400 MHz, $CDCl_3$
1.00
5.13
3.03
2.92
2.94
1.02
1.26
2.19
4.00
1.18
12
11
10
9
8
7
6
5
4
3
2
1
0
f1 (ppm)

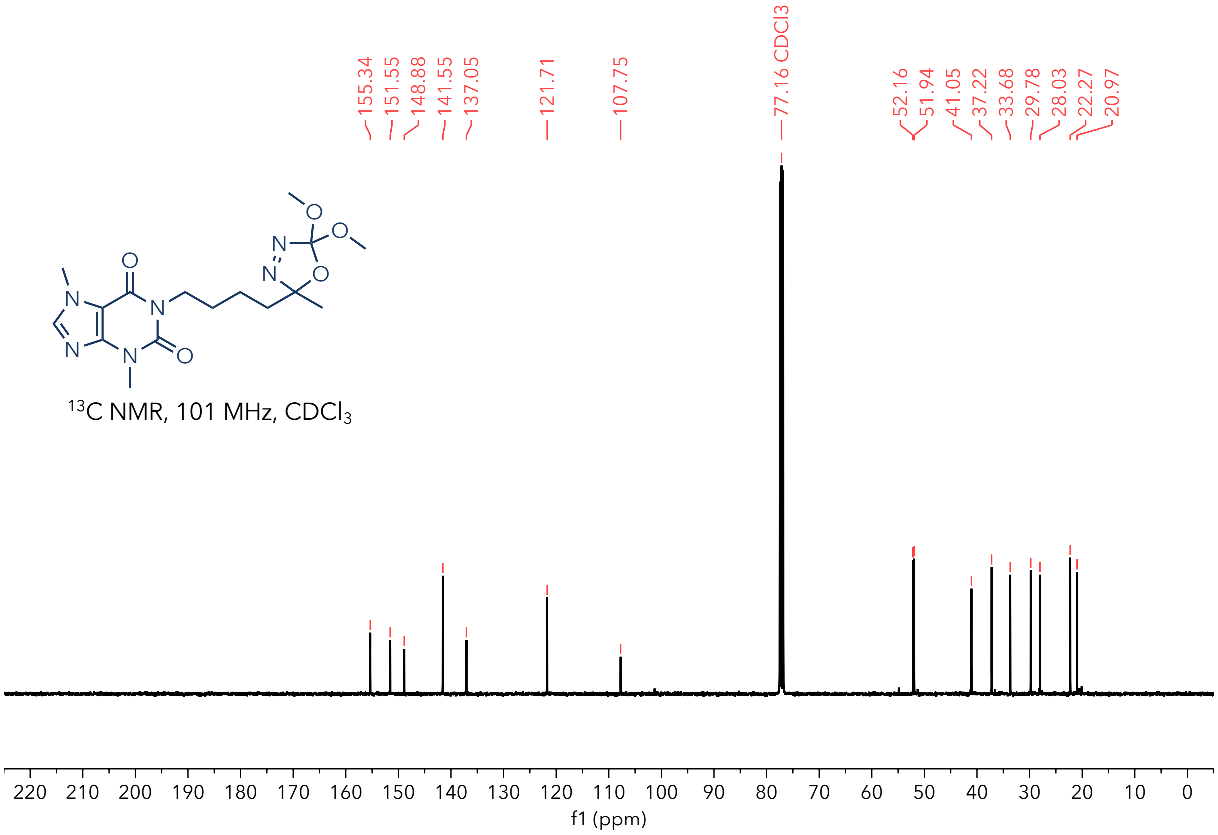
155.34
151.55
148.88
141.55
137.05
121.71
107.75
77.16 CDCl3
52.16
51.94
41.05
37.22
33.68
29.78
28.03
22.27
20.97
^{13}C NMR, 101 MHz, $CDCl_3$
220
210
200
190
180
170
160
150
140
130
120
110
100
90
80
70
60
50
40
30
20
10
0
f1 (ppm)

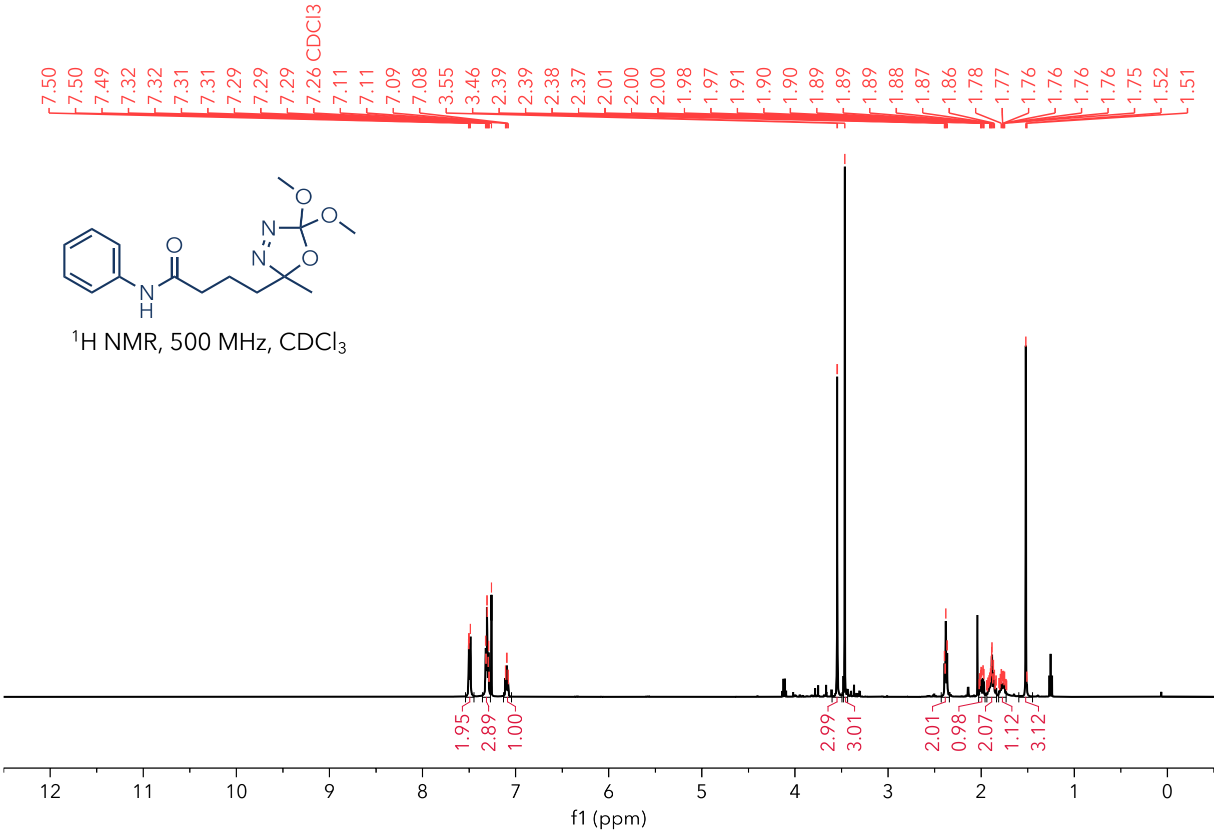

^{1}H NMR, 500 MHz, CDCl$_3$
f1 (ppm)

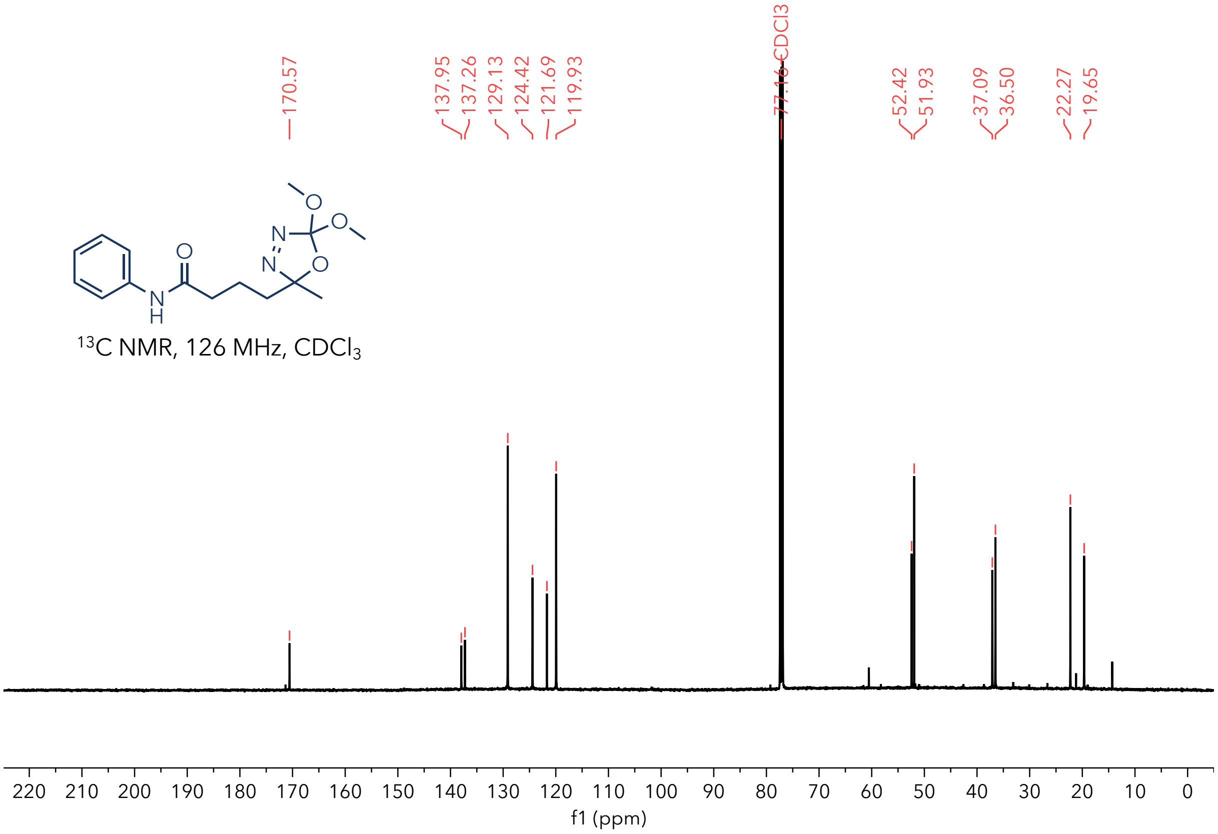

170.57
137.95
137.26
129.13
124.42
121.69
119.93
77.16 CDCl3
52.42
51.93
37.09
36.50
22.27
19.65
^{13}C NMR, 126 MHz, CDCl$_3$
f1 (ppm)

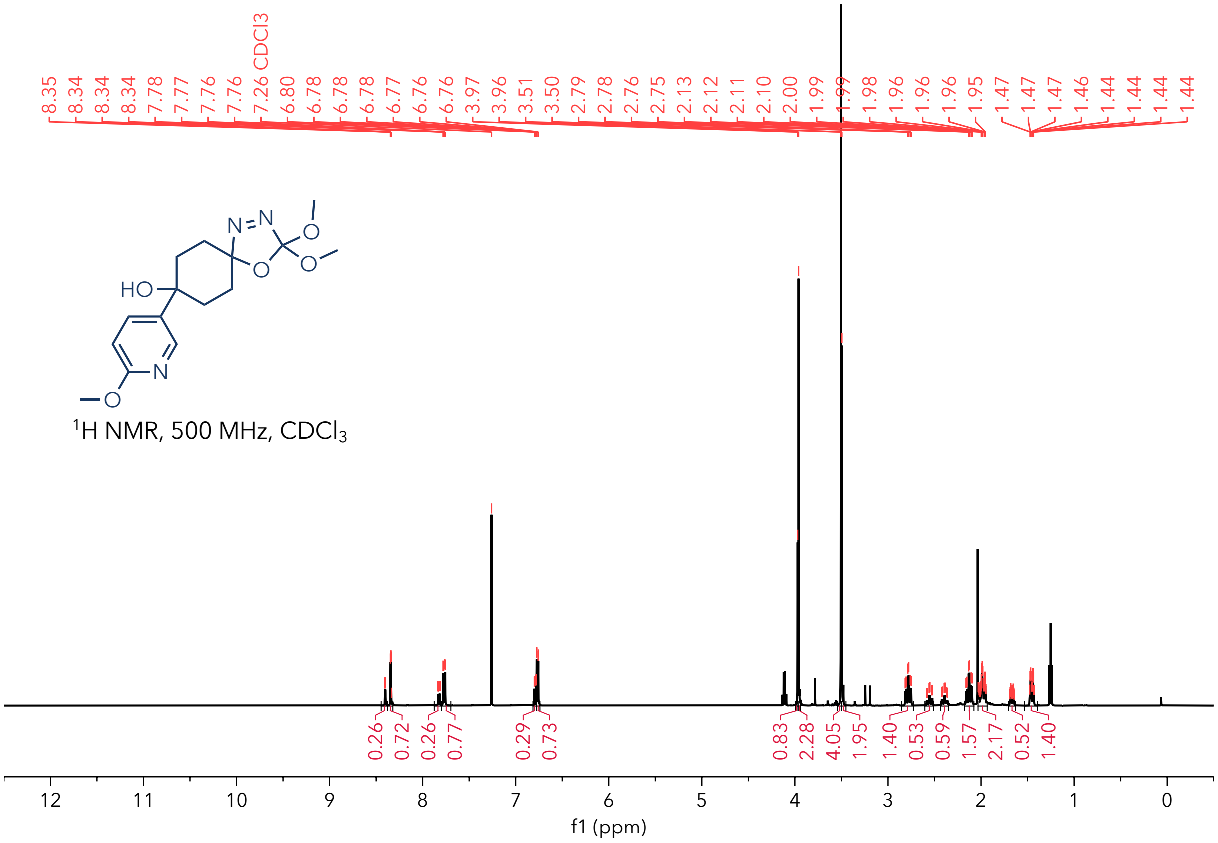

8.35 8.34 8.34 8.34 7.78 7.77 7.76 7.76 7.26 CDCl3 6.80 6.78 6.78 6.78 6.77 6.76 6.76 3.97 3.96 3.51 3.50 2.79 2.78 2.76 2.75 2.13 2.12 2.11 2.10 2.00 1.99 1.99 1.98 1.96 1.96 1.96 1.95 1.47 1.47 1.47 1.46 1.44 1.44 1.44 1.44
HO
N=N
O
N
¹H NMR, 500 MHz, CDCl₃
0.26 0.72 0.26 0.77 0.29 0.73 0.83 2.28 4.05 1.95 1.40 0.53 0.59 1.57 2.17 0.52 1.40
f1 (ppm)

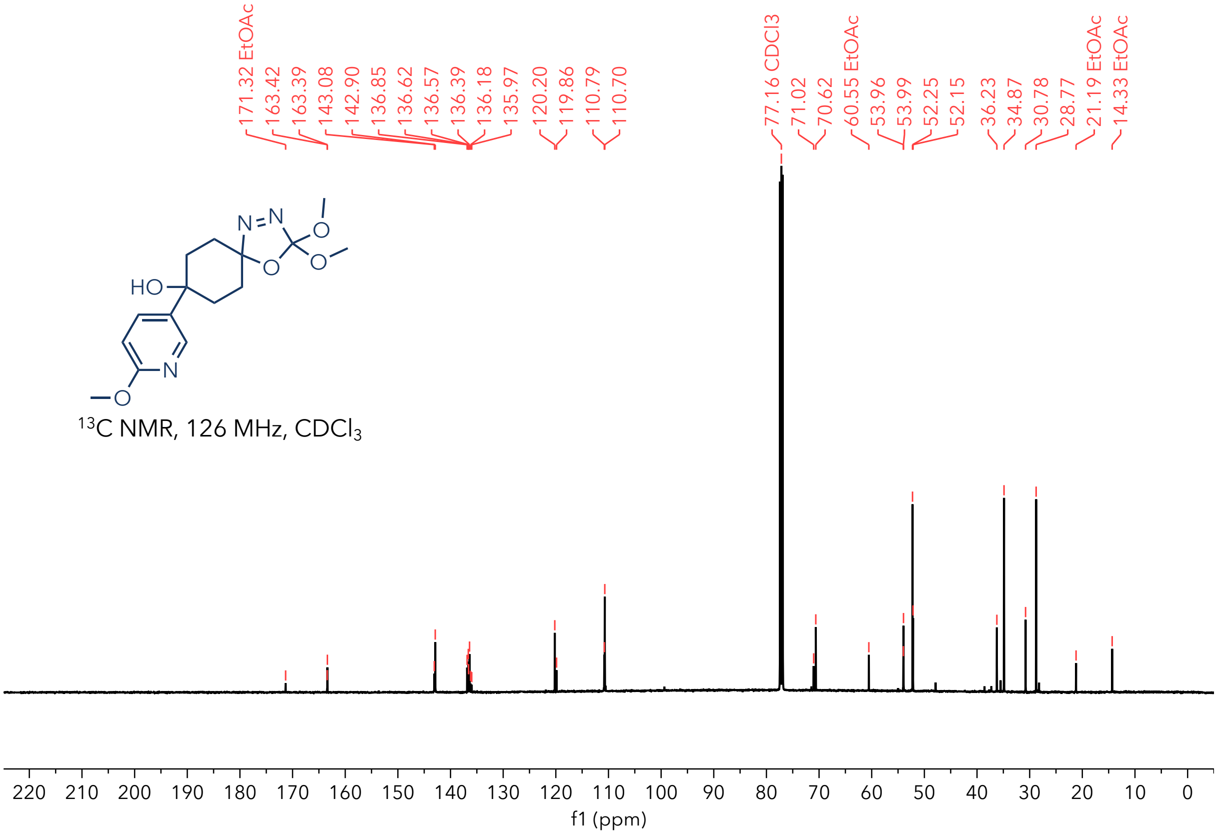

171.32 EtOAc 163.42 163.39 143.08 142.90 136.85 136.62 136.57 136.39 136.18 135.97 120.20 119.86 110.79 110.70 77.16 CDCl3 71.02 70.62 60.55 EtOAc 53.96 53.99 52.25 52.15 36.23 34.87 30.78 28.77 21.19 EtOAc 14.33 EtOAc
HO
N=N
O
N
¹³C NMR, 126 MHz, CDCl₃
f1 (ppm)

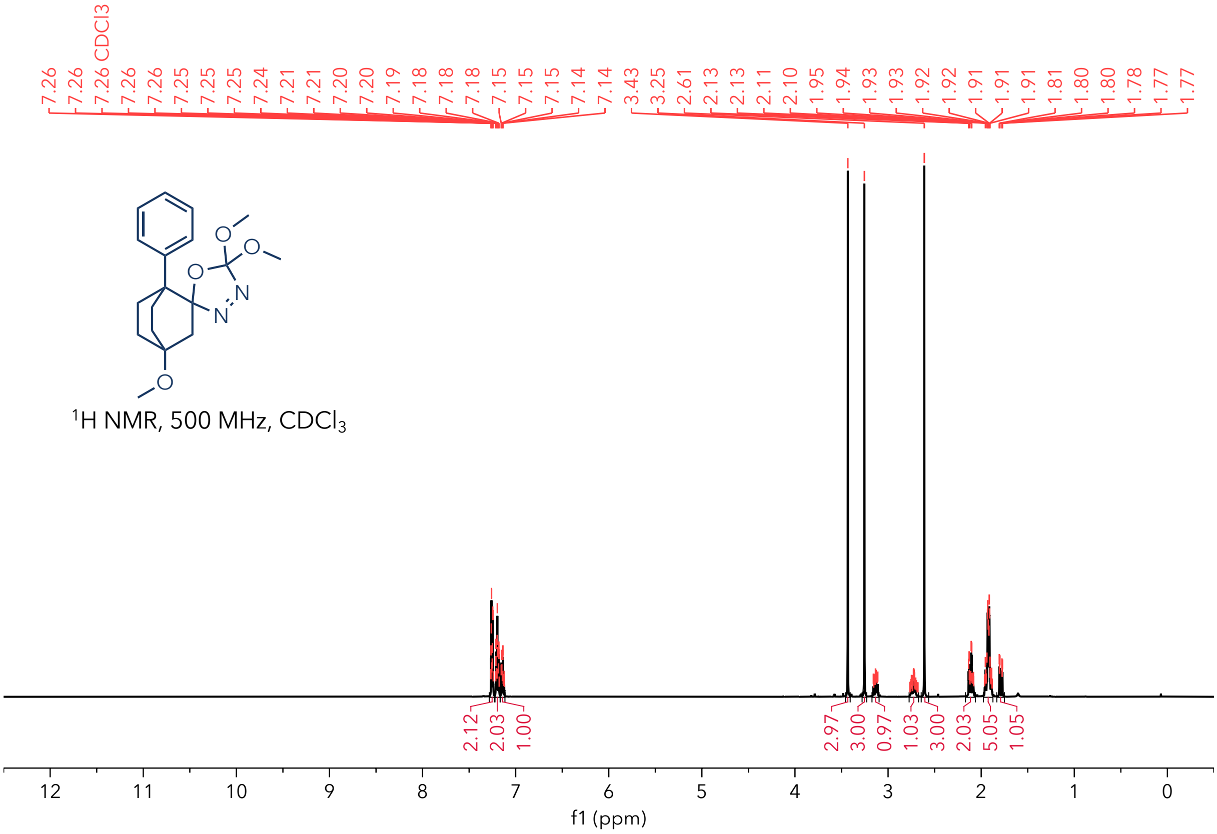
1H NMR, 500 MHz, CDCl3
7.26 7.26 7.26 CDCl3 7.26 7.26 7.25 7.25 7.25 7.24 7.21 7.21 7.20 7.20 7.19 7.18 7.18 7.18 7.15 7.15 7.15 7.14 7.14
3.43 3.25 2.61 2.13 2.13 2.11 2.10 1.95 1.94 1.93 1.93 1.92 1.92 1.91 1.91 1.91 1.81 1.80 1.80 1.78 1.77 1.77
2.12 2.03 1.00
2.97 3.00 0.97 1.03 3.00 2.03 5.05 1.05
12 11 10 9 8 7 6 5 4 3 2 1 0
f1 (ppm)

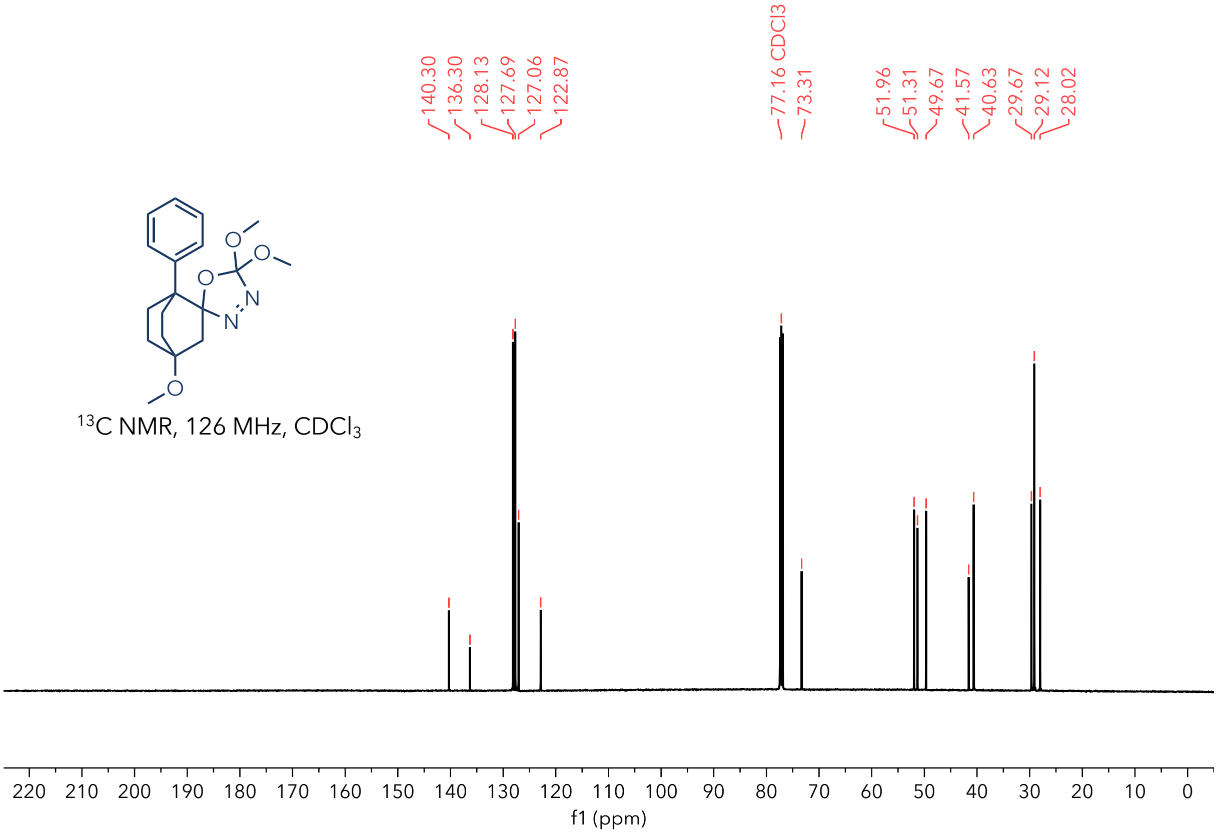
13C NMR, 126 MHz, CDCl3
140.30 136.30 128.13 127.69 127.06 122.87
77.16 CDCl3 73.31
51.96 51.31 49.67 41.57 40.63 29.67 29.12 28.02
220 210 200 190 180 170 160 150 140 130 120 110 100 90 80 70 60 50 40 30 20 10 0
f1 (ppm)

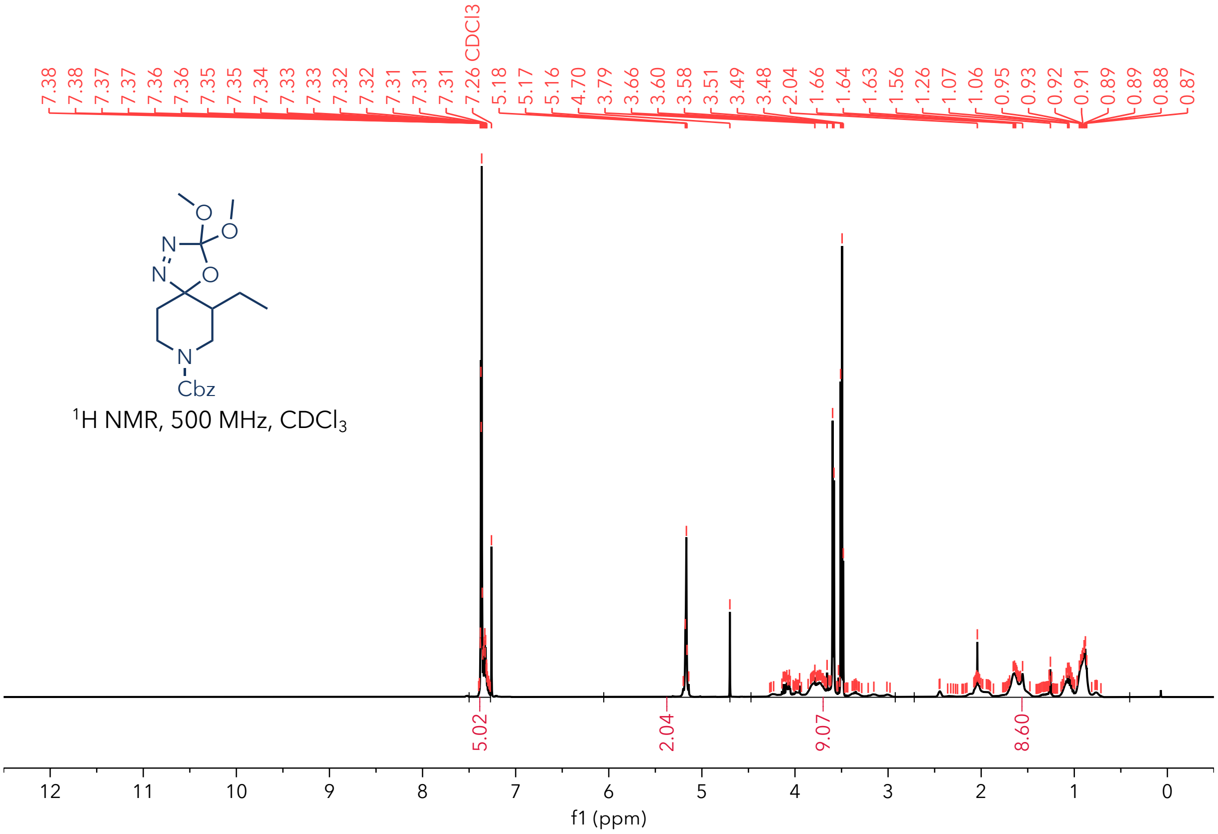
7.38
7.38
7.37
7.37
7.36
7.36
7.35
7.35
7.34
7.33
7.33
7.32
7.32
7.31
7.31
7.31
7.26 CDCl3
5.18
5.17
5.16
4.70
3.79
3.66
3.60
3.58
3.51
3.49
3.48
2.04
1.66
1.64
1.63
1.56
1.26
1.07
1.06
0.95
0.93
0.92
0.91
0.89
0.89
0.88
0.87
N
N
O
O
O
N
Cbz
¹H NMR, 500 MHz, CDCl₃
5.02
2.04
9.07
8.60
12
11
10
9
8
7
6
5
4
3
2
1
0
f1 (ppm)

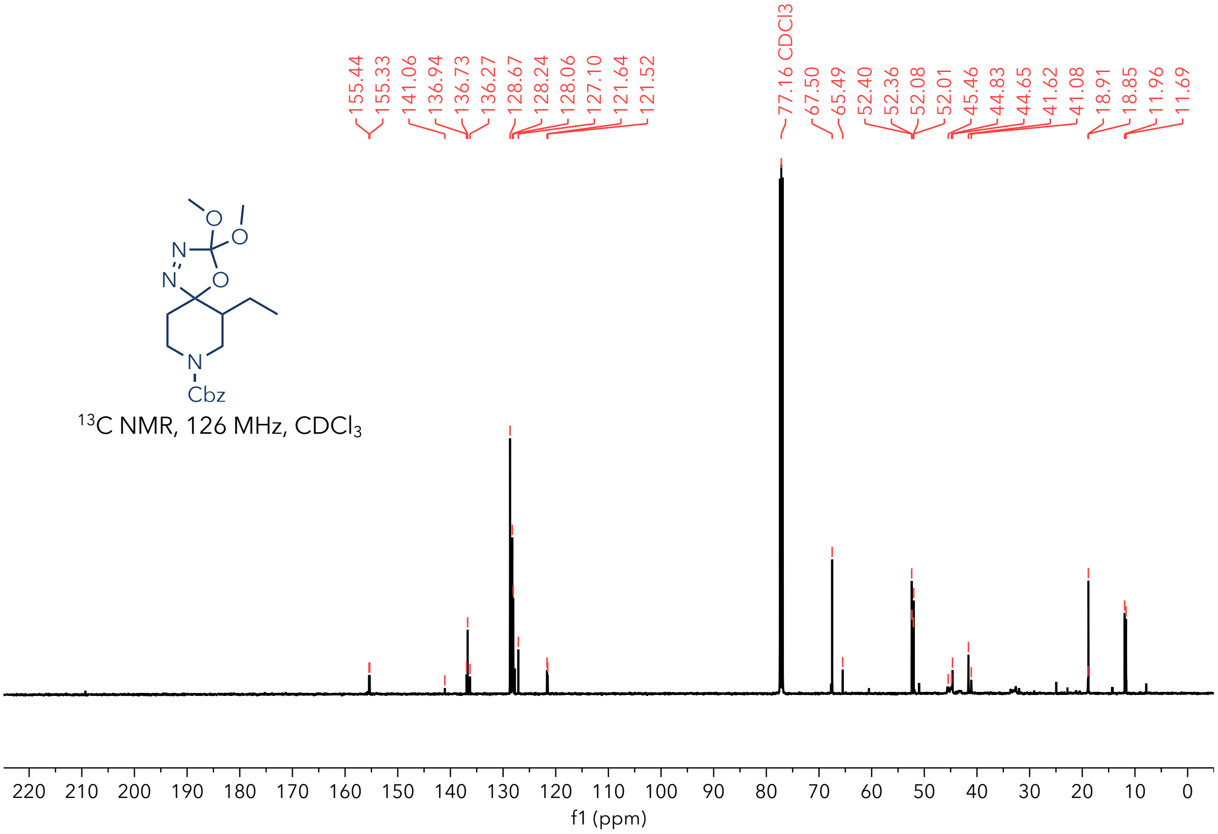
155.44
155.33
141.06
136.94
136.73
136.27
128.67
128.24
128.06
127.10
121.64
121.52
77.16 CDCl3
67.50
65.49
52.40
52.36
52.08
52.01
45.46
44.83
44.65
41.62
41.08
18.91
18.85
11.96
11.69
N
N
O
O
O
N
Cbz
¹³C NMR, 126 MHz, CDCl₃
220
210
200
190
180
170
160
150
140
130
120
110
100
90
80
70
60
50
40
30
20
10
0
f1 (ppm)

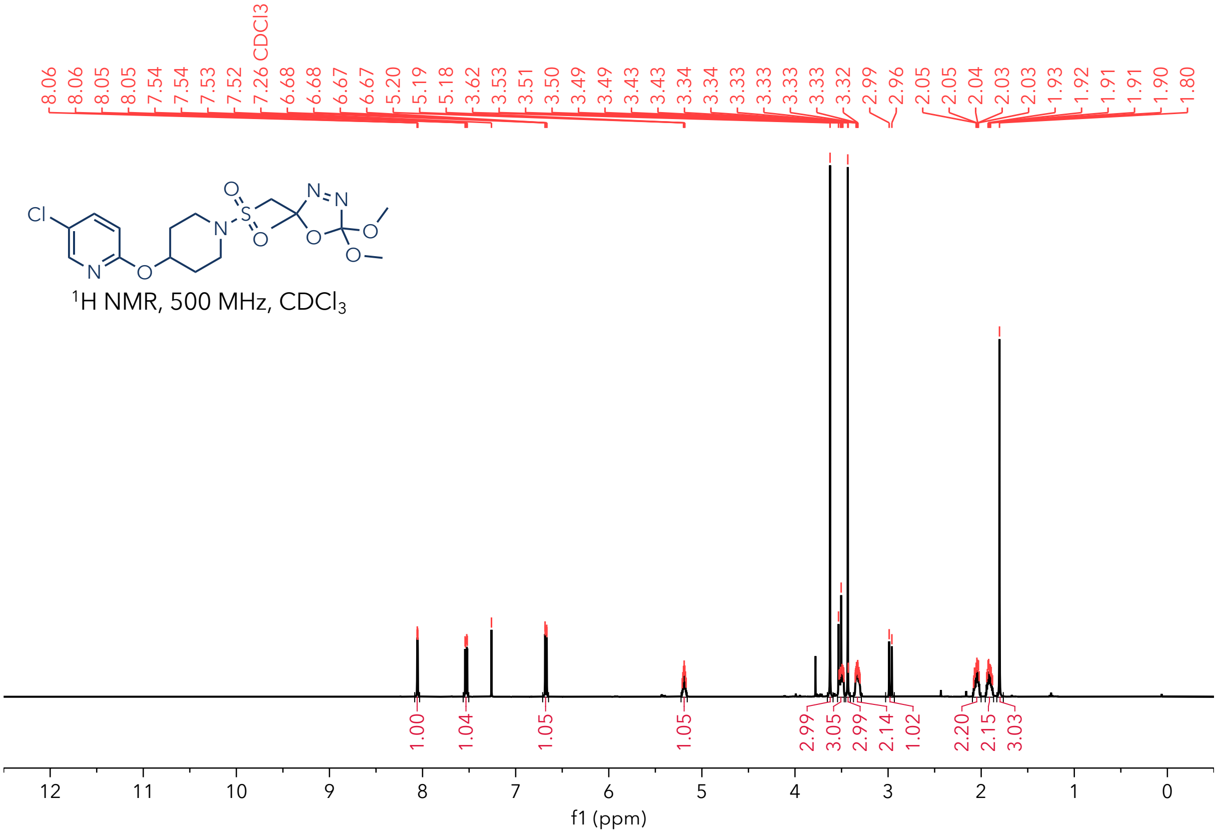
8.06 8.06 8.05 8.05 7.54 7.54 7.53 7.52 7.26 CDCl3 6.68 6.68 6.67 6.67 5.20 5.19 5.18 3.62 3.53 3.51 3.50 3.49 3.49 3.43 3.43 3.34 3.34 3.33 3.33 3.33 3.33 3.32 2.99 2.96 2.05 2.05 2.04 2.03 2.03 1.93 1.92 1.91 1.91 1.90 1.80
Cl
N
O
N
S
O
O
N
N
O
O
O
1H NMR, 500 MHz, CDCl3
1.00 1.04 1.05 1.05 2.99 3.05 2.99 2.14 1.02 2.20 2.15 3.03
12 11 10 9 8 7 6 5 4 3 2 1 0
f1 (ppm)
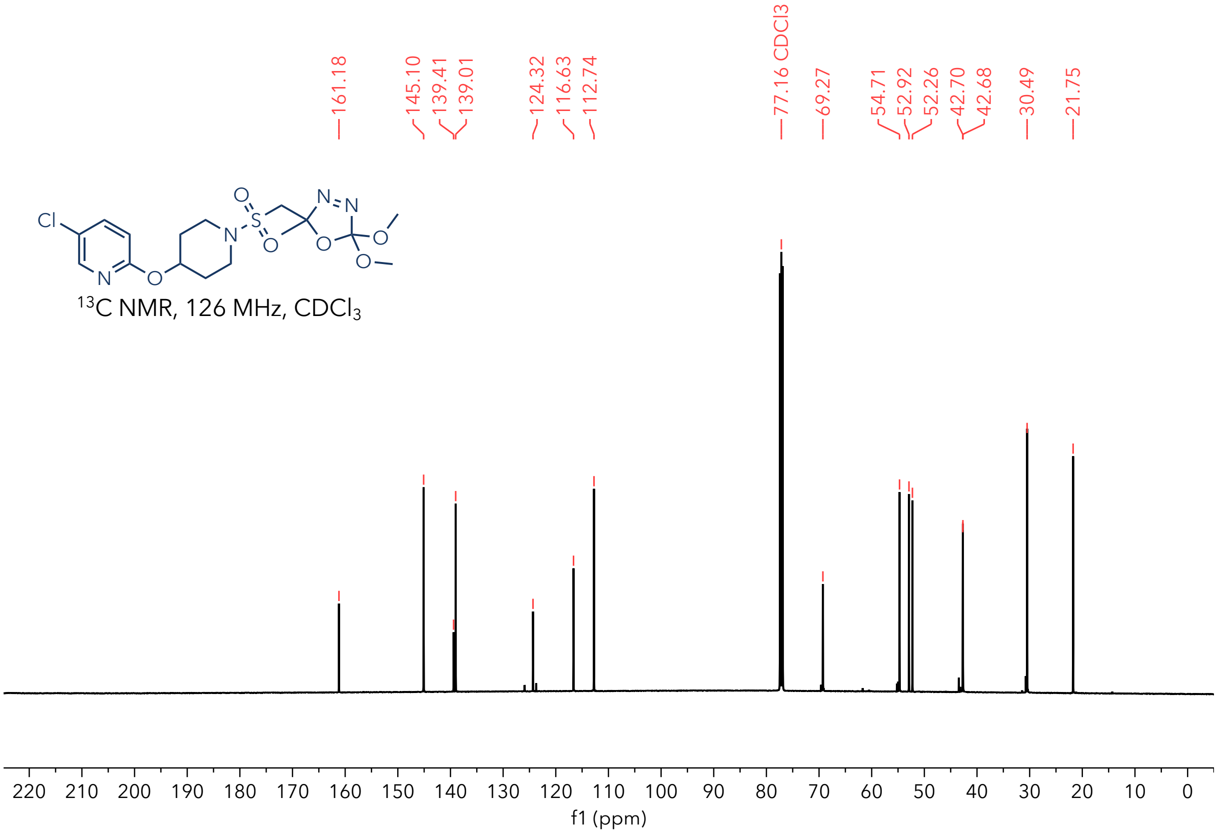
161.18 145.10 139.41 139.01 124.32 116.63 112.74 77.16 CDCl3 69.27 54.71 52.92 52.26 42.70 42.68 30.49 21.75
Cl
N
O
N
S
O
O
N
N
O
O
O
13C NMR, 126 MHz, CDCl3
220 210 200 190 180 170 160 150 140 130 120 110 100 90 80 70 60 50 40 30 20 10 0
f1 (ppm)

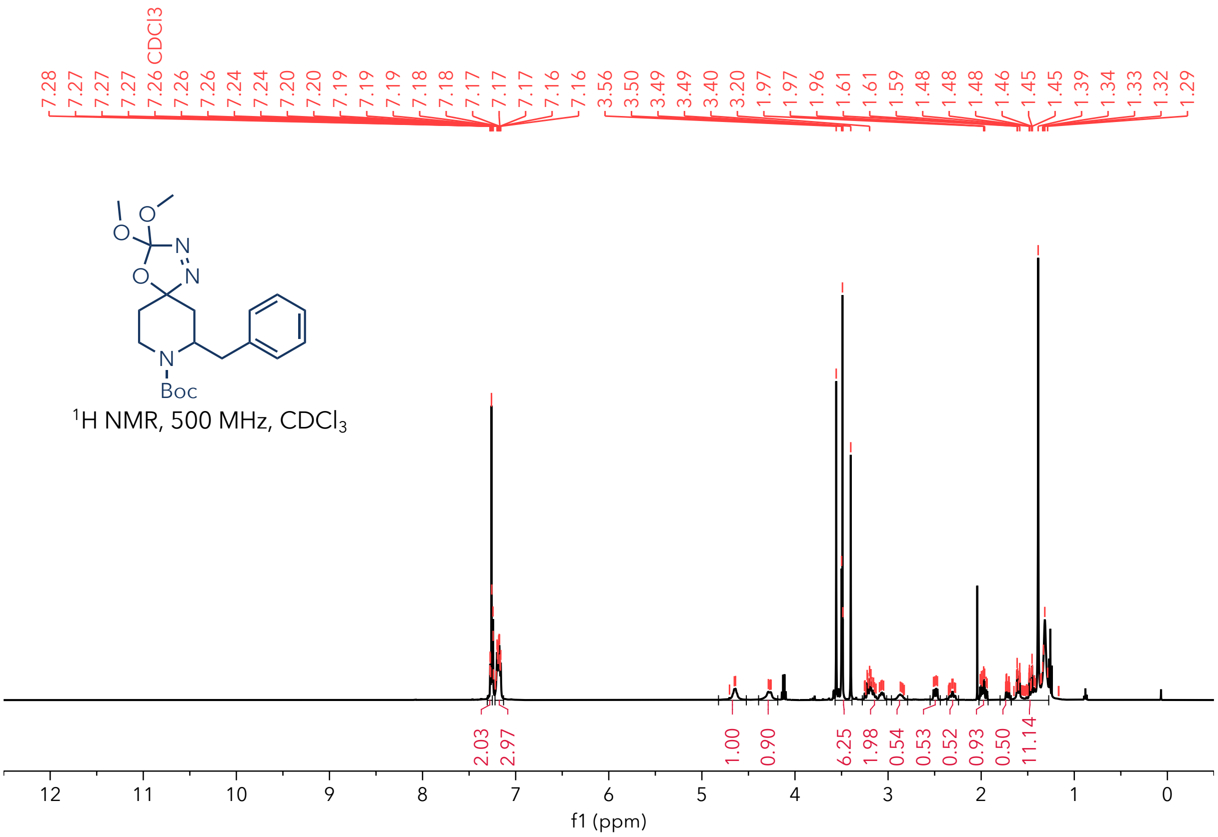
7.28 7.27 7.27 7.27 7.26 CDCl3 7.26 7.26 7.24 7.24 7.20 7.20 7.19 7.19 7.19 7.18 7.18 7.17 7.17 7.17 7.16 7.16
3.56 3.50 3.49 3.49 3.40 3.20 1.97 1.97 1.96 1.61 1.61 1.59 1.48 1.48 1.48 1.46 1.45 1.45 1.39 1.34 1.33 1.32 1.29
O
O
N
N
O
N
Boc
^{1}H NMR, 500 MHz, $CDCl_3$
2.03 2.97 1.00 0.90 6.25 1.98 0.54 0.53 0.52 0.93 0.50 11.14
12 11 10 9 8 7 6 5 4 3 2 1 0
f1 (ppm)

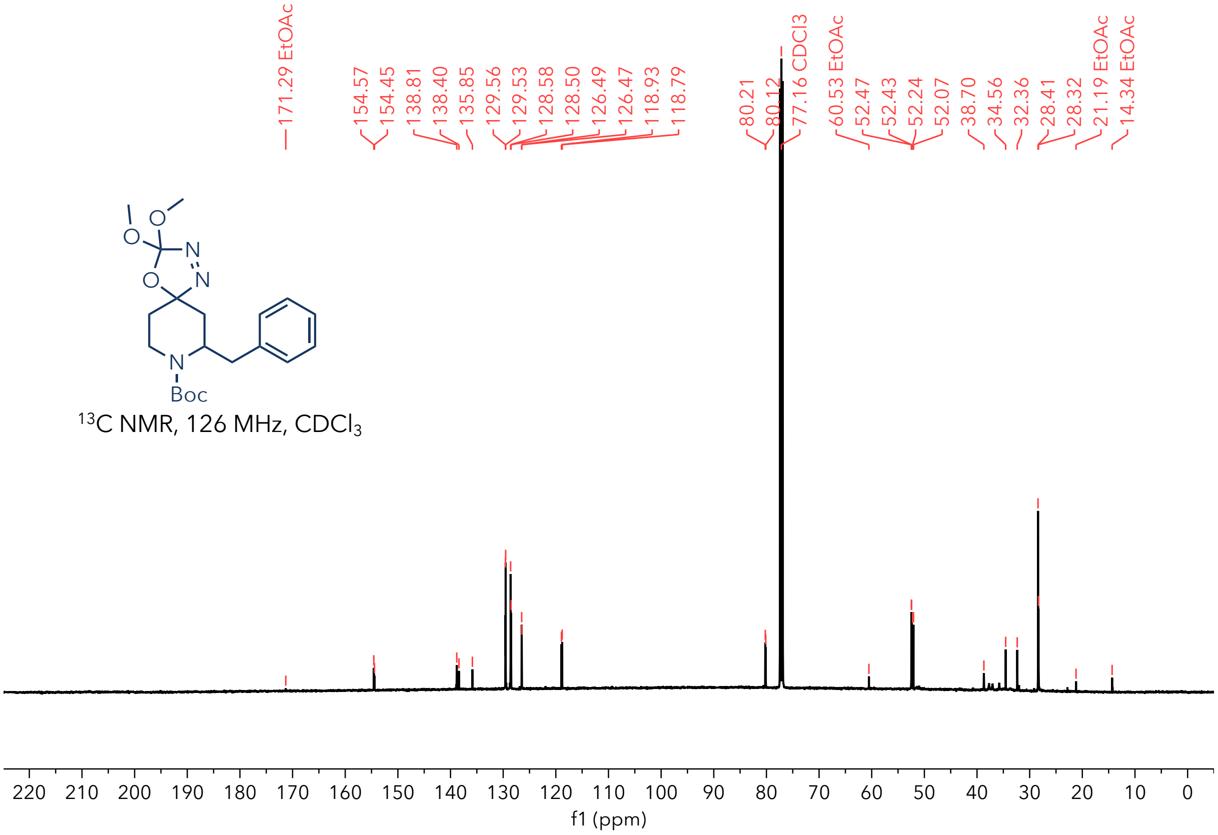
171.29 EtOAc
154.57 154.45 138.81 138.40 135.85 129.56 129.53 128.58 128.50 126.49 126.47 118.93 118.79
80.21 80.12 77.16 CDCl3 60.53 EtOAc 52.47 52.43 52.24 52.07 38.70 34.56 32.36 28.41 28.32 21.19 EtOAc 14.34 EtOAc
O
O
N
N
O
N
Boc
^{13}C NMR, 126 MHz, $CDCl_3$
220 210 200 190 180 170 160 150 140 130 120 110 100 90 80 70 60 50 40 30 20 10 0
f1 (ppm)

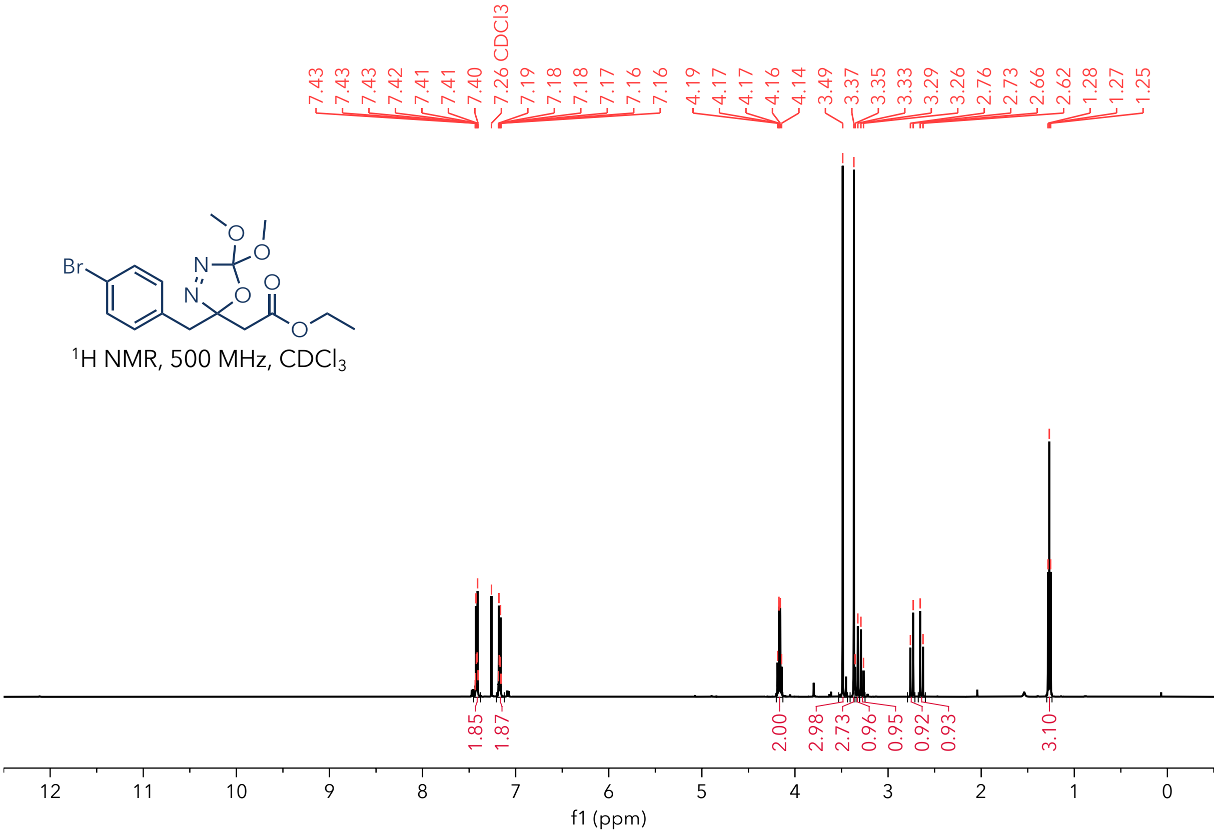

Br
N
N
O
O
O
O
O
¹H NMR, 500 MHz, CDCl₃
7.43
7.43
7.43
7.42
7.41
7.41
7.40
7.26 CDCl3
7.19
7.18
7.18
7.17
7.16
7.16
4.19
4.17
4.17
4.16
4.14
3.49
3.37
3.35
3.33
3.29
3.26
2.76
2.73
2.66
2.62
1.28
1.27
1.25
1.85
1.87
2.00
2.98
2.73
0.96
0.95
0.92
0.93
3.10
12
11
10
9
8
7
6
5
4
3
2
1
0
f1 (ppm)

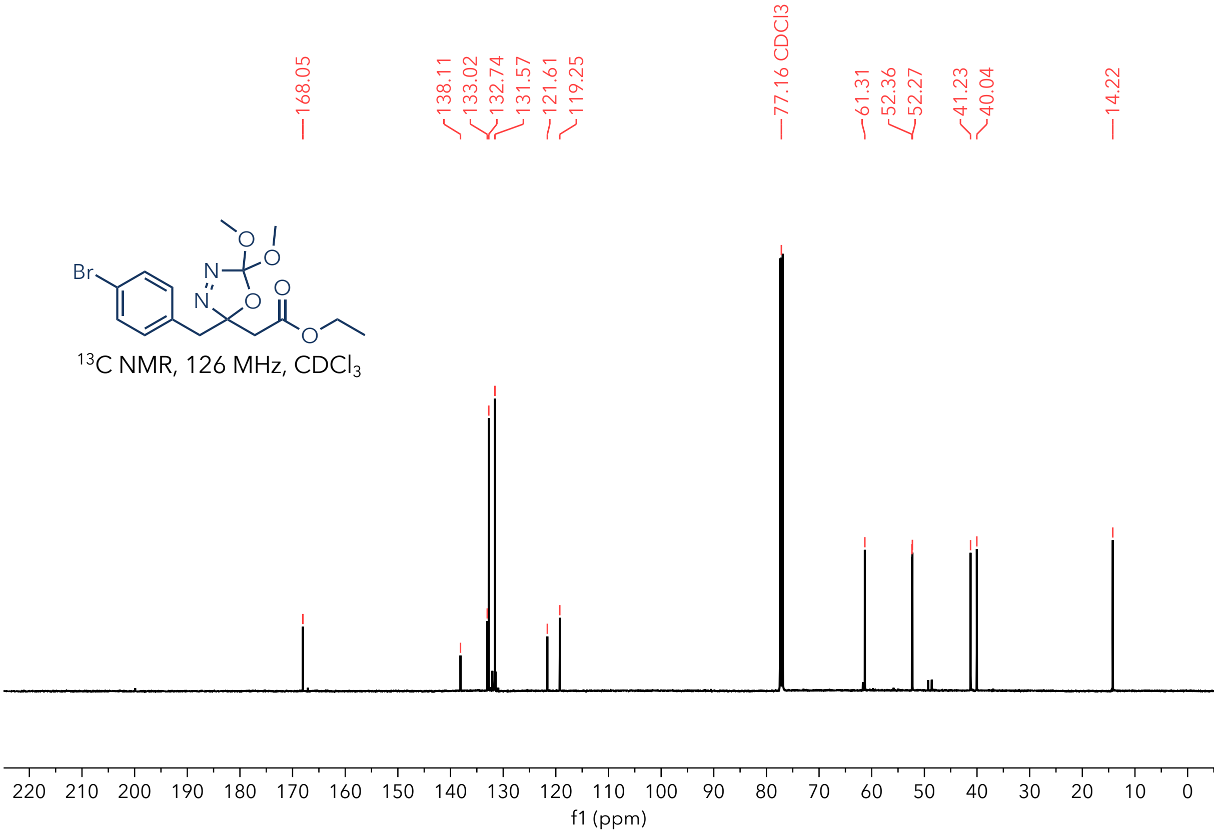

Br
N
N
O
O
O
O
O
¹³C NMR, 126 MHz, CDCl₃
168.05
138.11
133.02
132.74
131.57
121.61
119.25
77.16 CDCl3
61.31
52.36
52.27
41.23
40.04
14.22
220
210
200
190
180
170
160
150
140
130
120
110
100
90
80
70
60
50
40
30
20
10
0
f1 (ppm)

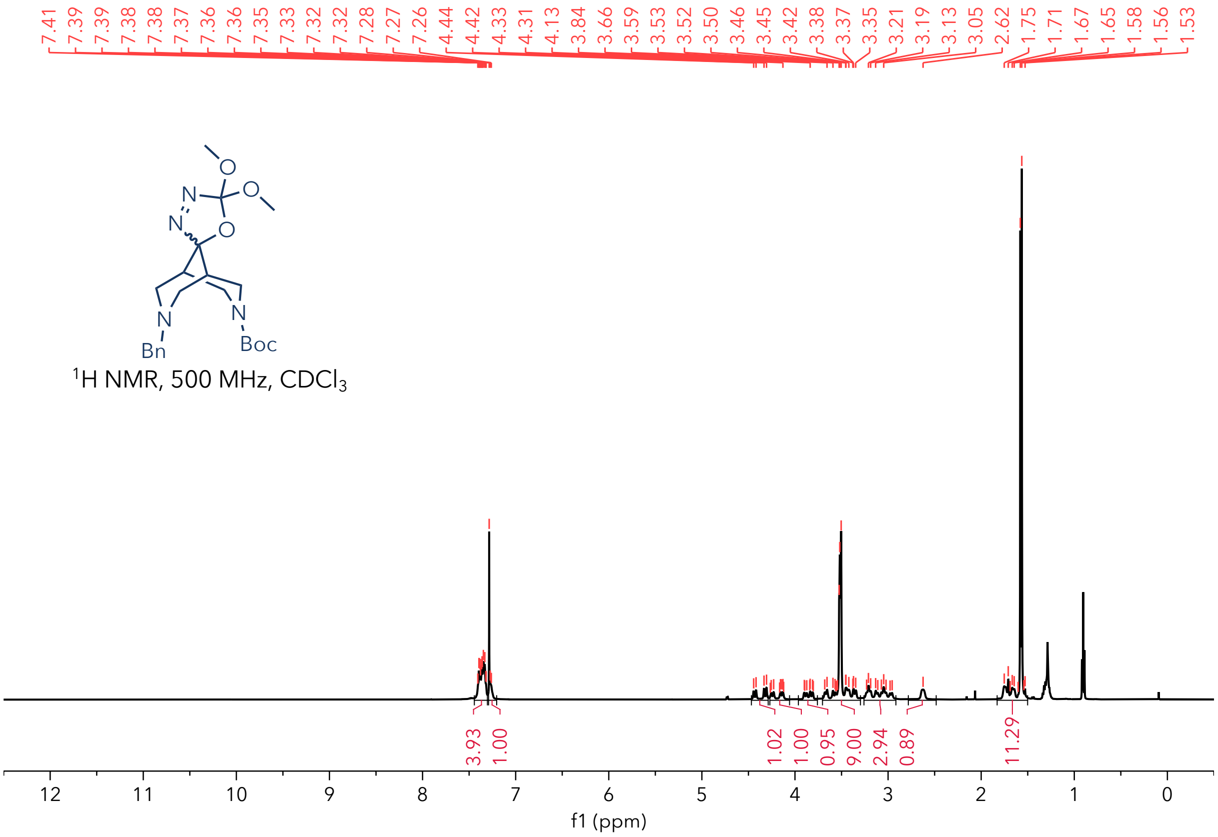
7.41 7.39 7.39 7.38 7.38 7.37 7.36 7.36 7.35 7.33 7.32 7.32 7.28 7.27 7.26 4.44 4.42 4.33 4.31 4.13 3.84 3.66 3.59 3.53 3.52 3.50 3.46 3.45 3.42 3.38 3.37 3.35 3.21 3.19 3.13 3.05 2.62 1.75 1.71 1.67 1.65 1.58 1.56 1.53
N
N
O
O
O
N
N
Bn
Boc
^{1}H NMR, 500 MHz, $CDCl_3$
3.93 1.00 1.02 1.00 0.95 9.00 2.94 0.89 11.29
12 11 10 9 8 7 6 5 4 3 2 1 0
f1 (ppm)

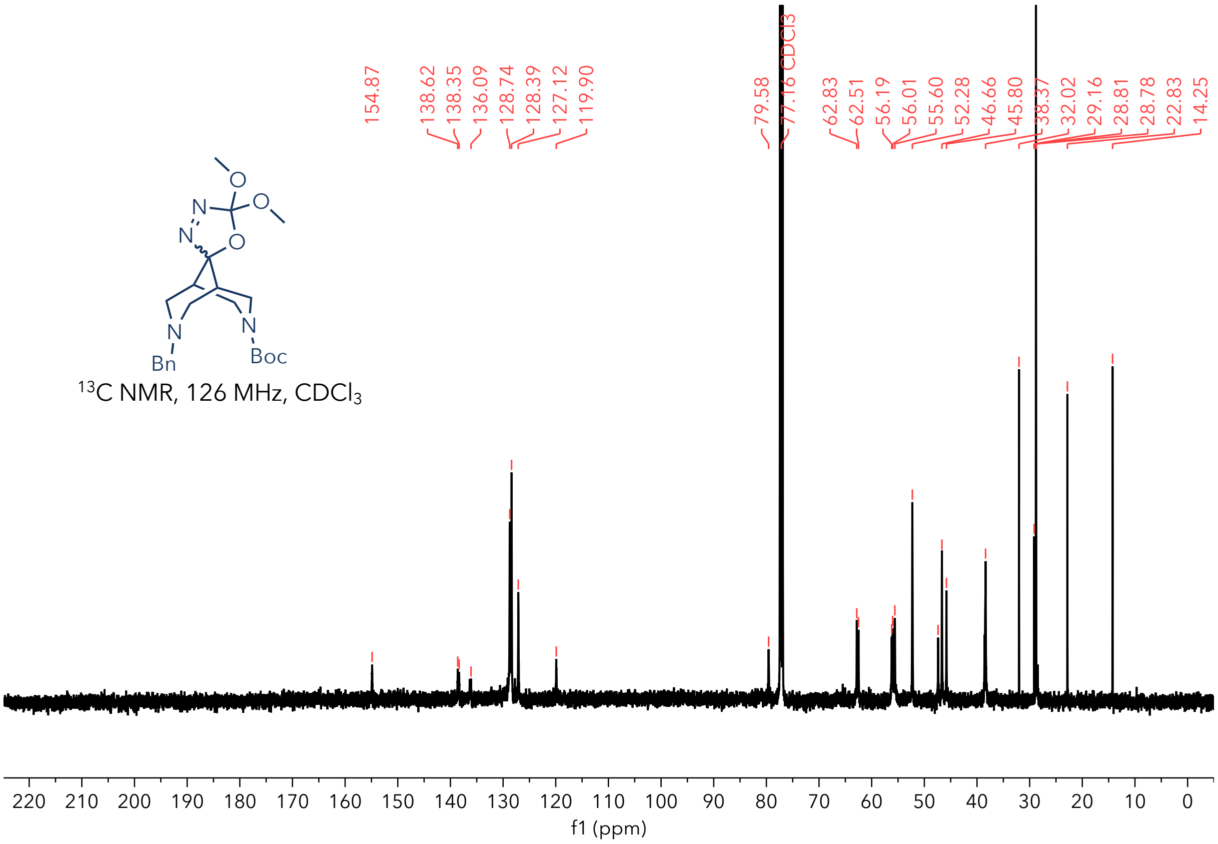
154.87
138.62 138.35 136.09 128.74 128.39 127.12 119.90
79.58 77.16 CDCl3
62.83 62.51 56.19 56.01 55.60 52.28 46.66 45.80 38.37 32.02 29.16 28.81 28.78 22.83 14.25
N
N
O
O
O
N
N
Bn
Boc
^{13}C NMR, 126 MHz, $CDCl_3$
220 210 200 190 180 170 160 150 140 130 120 110 100 90 80 70 60 50 40 30 20 10 0
f1 (ppm)

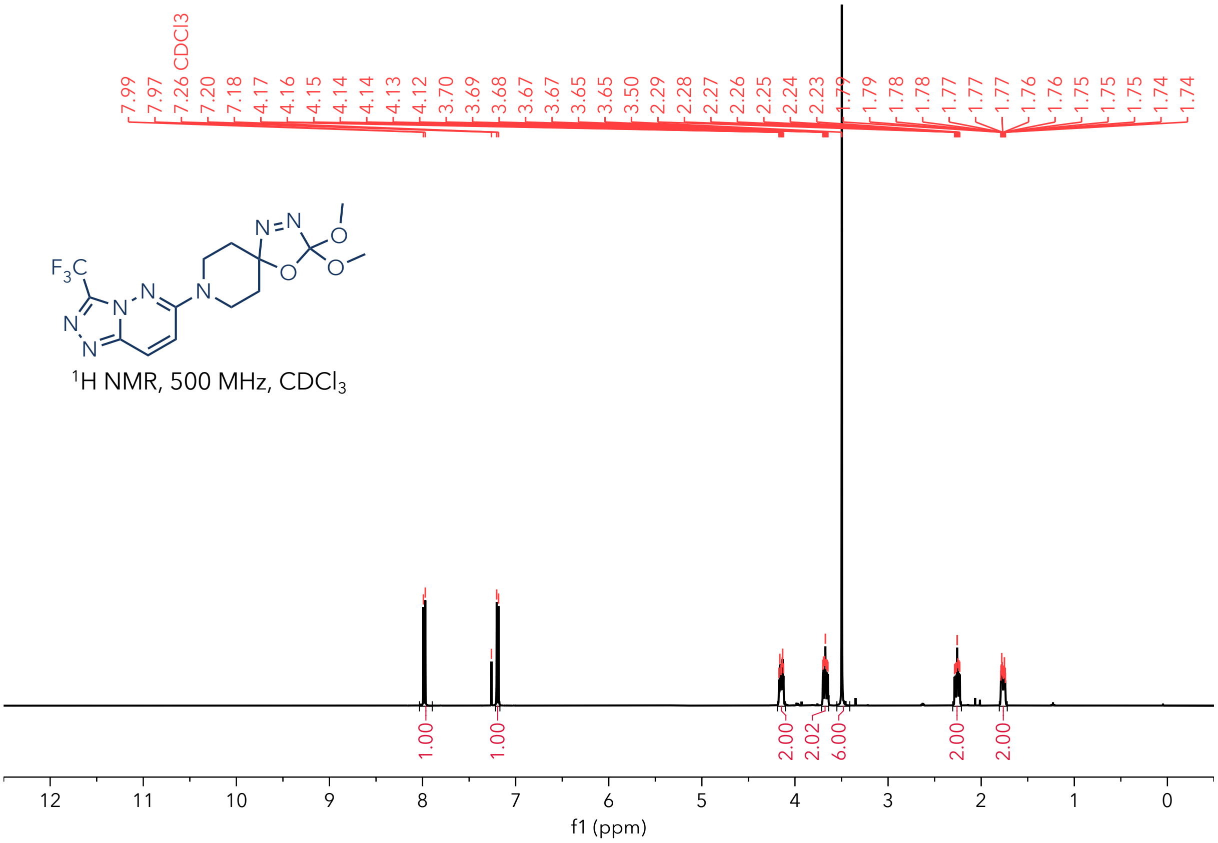

^{1}H NMR, 500 MHz, $CDCl_3$
f1 (ppm)

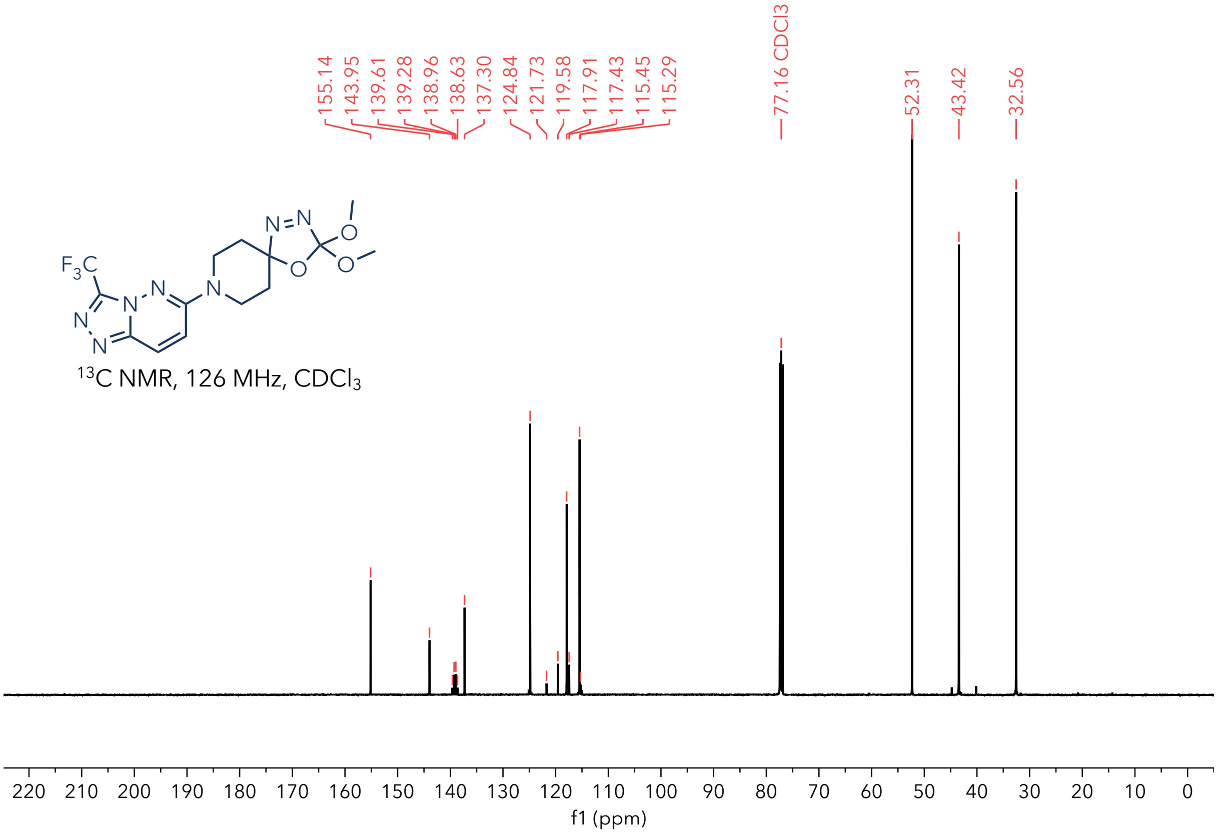

^{13}C NMR, 126 MHz, $CDCl_3$
f1 (ppm)

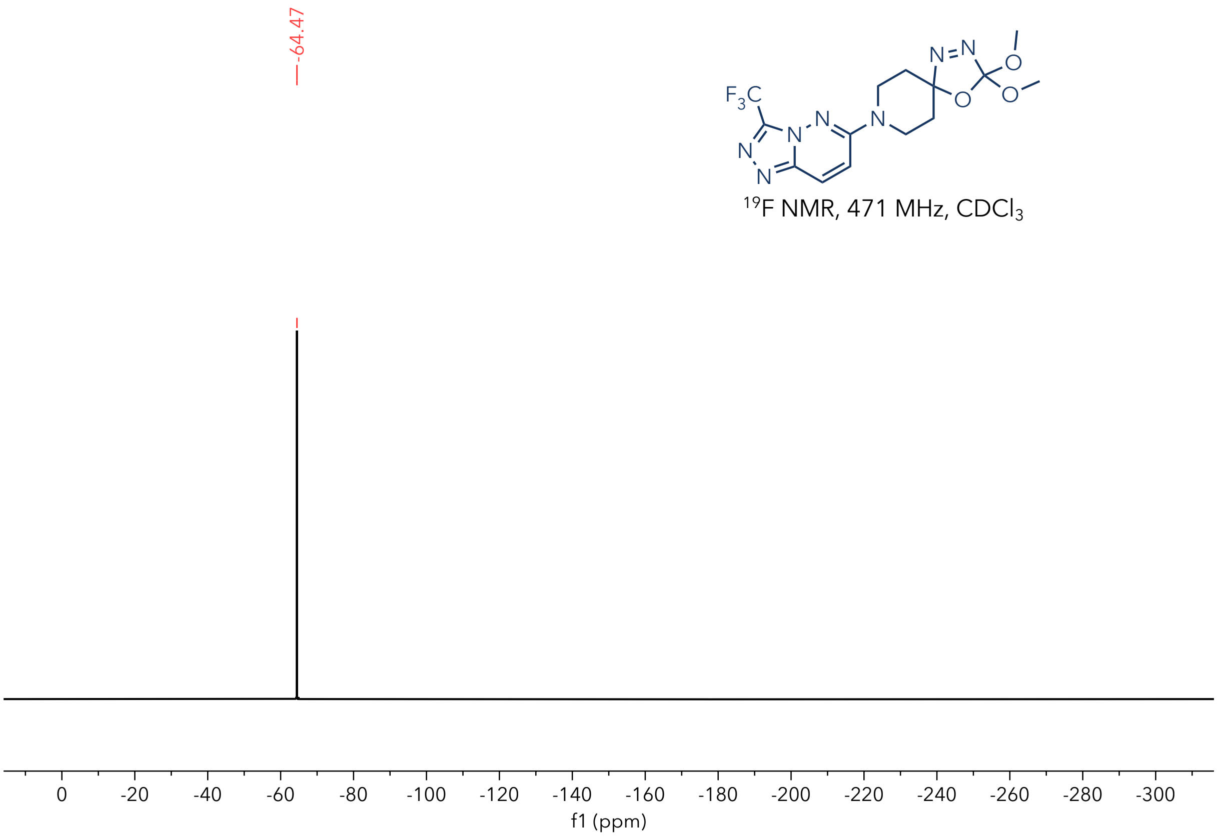
-64.47
F3C
N
N
N
N
N
N
N=N
O
O
O
19F NMR, 471 MHz, CDCl3
0
-20
-40
-60
-80
-100
-120
-140
-160
-180
-200
-220
-240
-260
-280
-300
f1 (ppm)

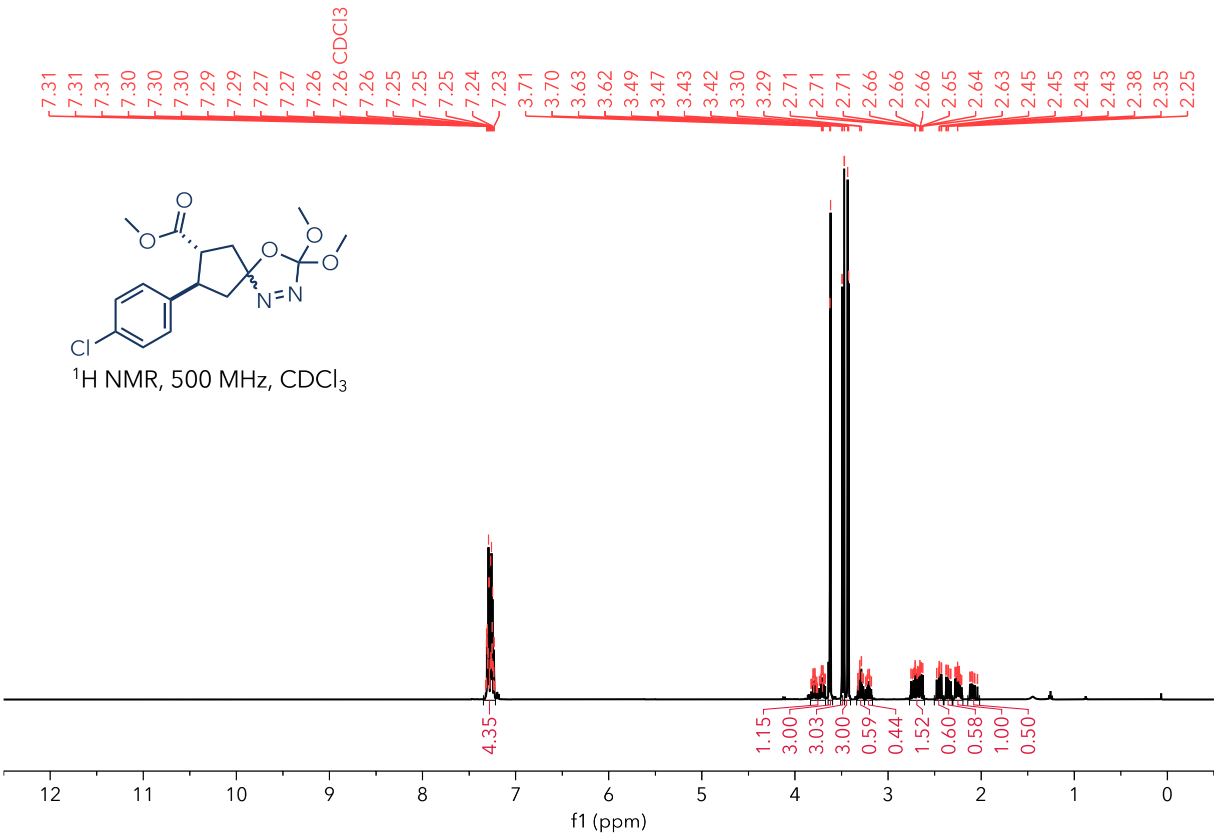
7.31 7.31 7.31 7.30 7.30 7.30 7.29 7.29 7.27 7.27 7.26 7.26 CDCl3 7.26 7.25 7.25 7.25 7.24 7.23
3.71 3.70 3.63 3.62 3.49 3.47 3.43 3.42 3.30 3.29 2.71 2.71 2.71 2.66 2.66 2.66 2.65 2.64 2.63 2.45 2.45 2.43 2.43 2.38 2.35 2.25
O
O
O
O
N=N
Cl
^{1}H NMR, 500 MHz, CDCl$_3$
f1 (ppm)

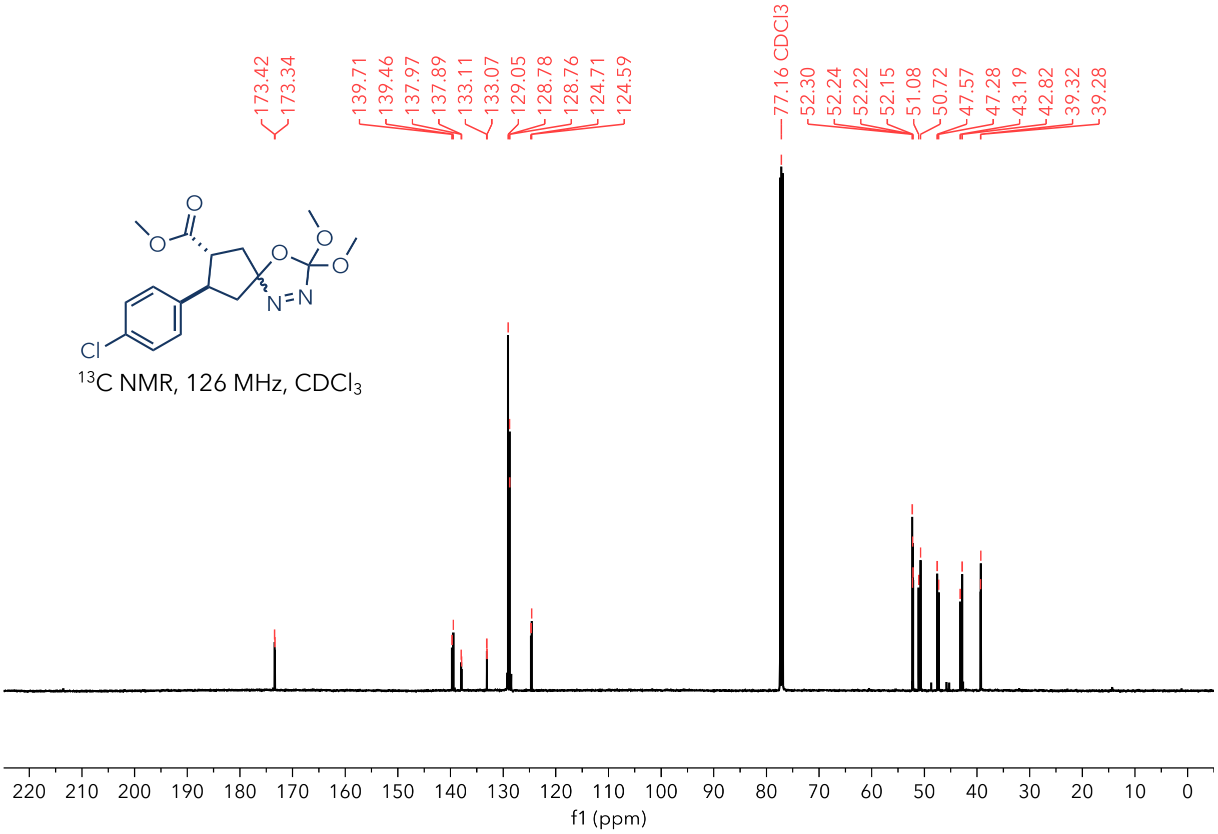
173.42 173.34
139.71 139.46 137.97 137.89 133.11 133.07 129.05 128.78 128.76 124.71 124.59
77.16 CDCl3
52.30 52.24 52.22 52.15 51.08 50.72 47.57 47.28 43.19 42.82 39.32 39.28
O
O
O
O
N=N
Cl
^{13}C NMR, 126 MHz, CDCl$_3$
f1 (ppm)

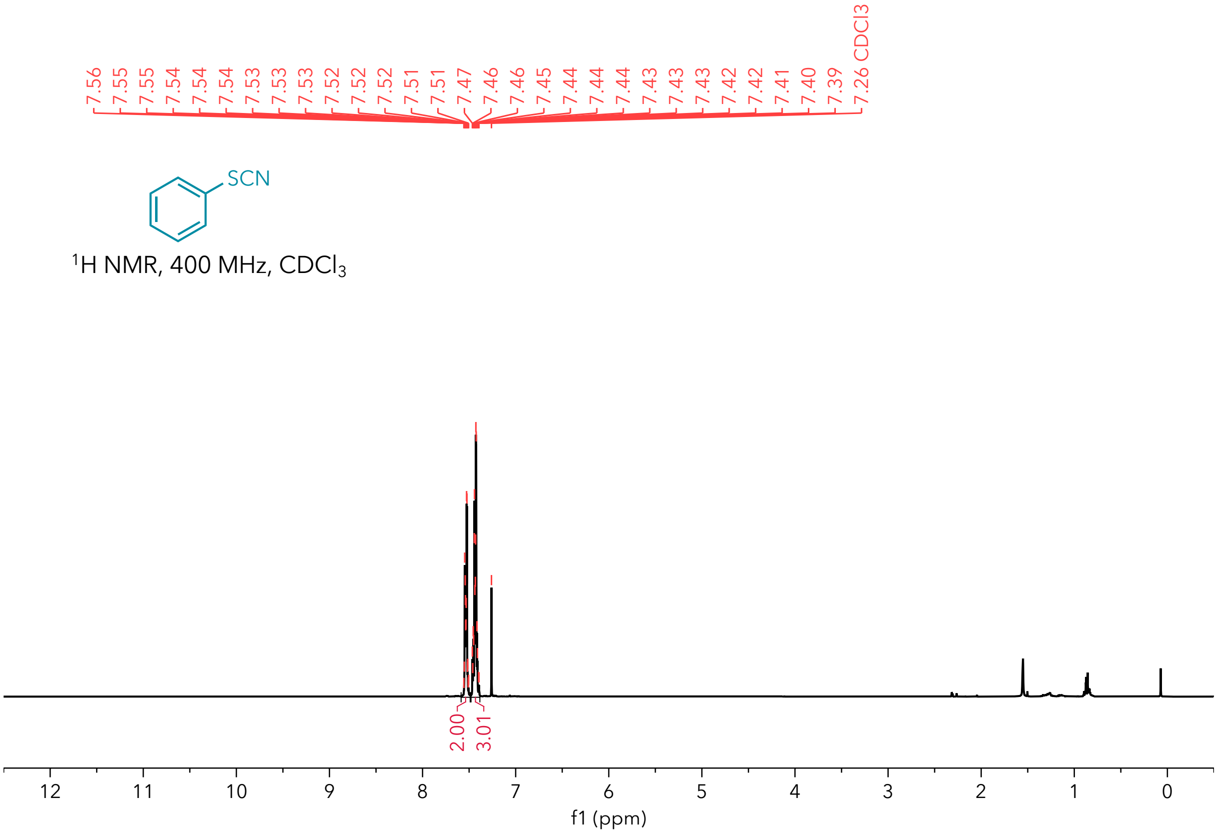
7.56 7.55 7.55 7.54 7.54 7.54 7.53 7.53 7.53 7.52 7.52 7.52 7.51 7.51 7.47 7.46 7.46 7.45 7.44 7.44 7.44 7.43 7.43 7.43 7.42 7.42 7.41 7.40 7.39 7.26 CDCl3
SCN
^1H NMR, 400 MHz, $CDCl_3$
2.00
3.01
f1 (ppm)

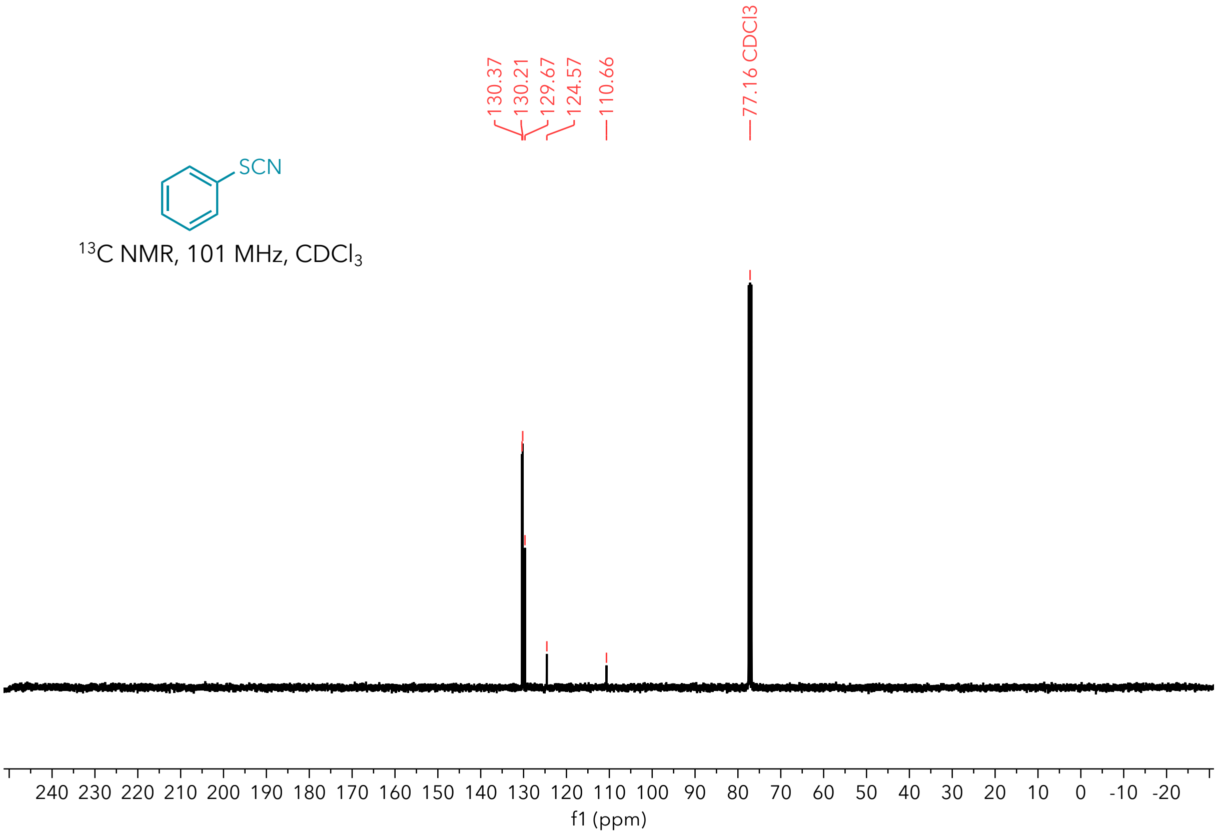
130.37
130.21
129.67
124.57
110.66
77.16 CDCl3
SCN
^{13}C NMR, 101 MHz, $CDCl_3$
f1 (ppm)

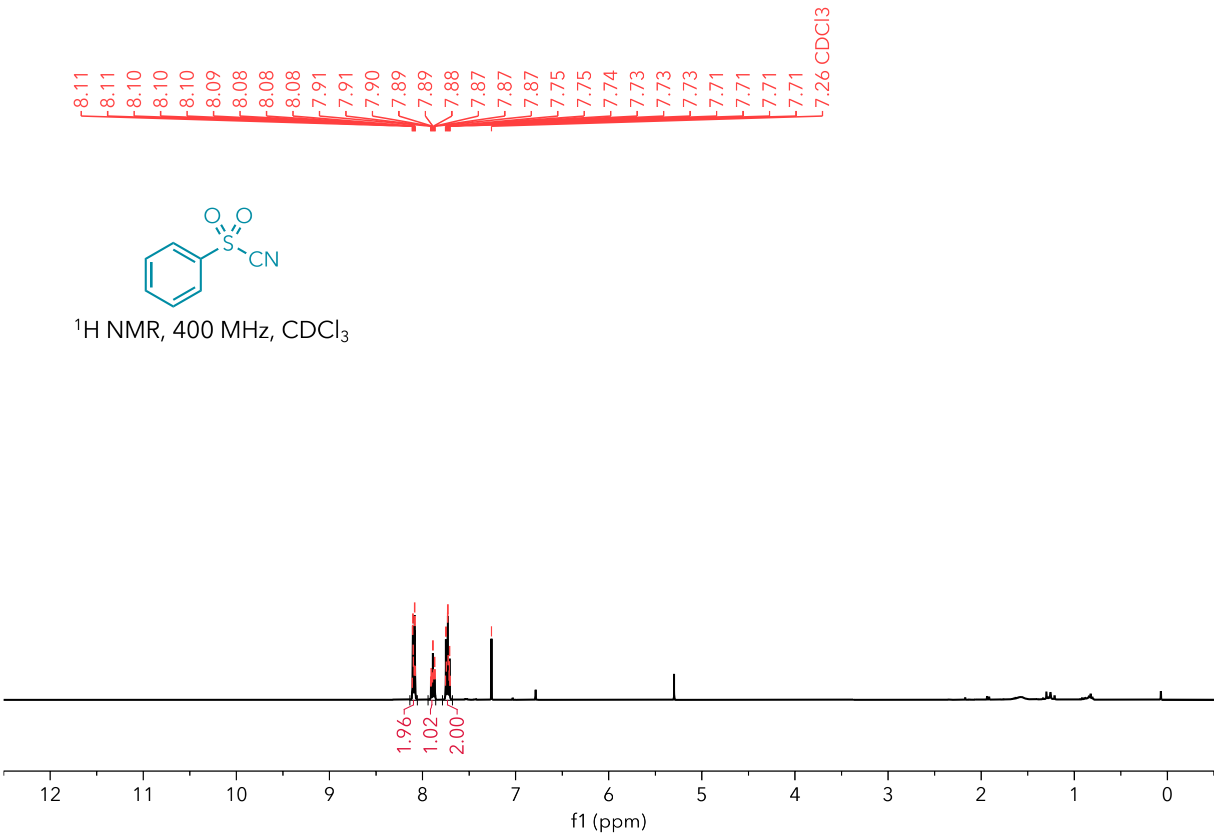

8.11 8.11 8.10 8.10 8.10 8.09 8.08 8.08 8.08 7.91 7.91 7.90 7.89 7.89 7.88 7.87 7.87 7.87 7.75 7.75 7.74 7.73 7.73 7.73 7.71 7.71 7.71 7.71 7.26 CDCl3
CN
1H NMR, 400 MHz, CDCl3
1.96
1.02
2.00
f1 (ppm)

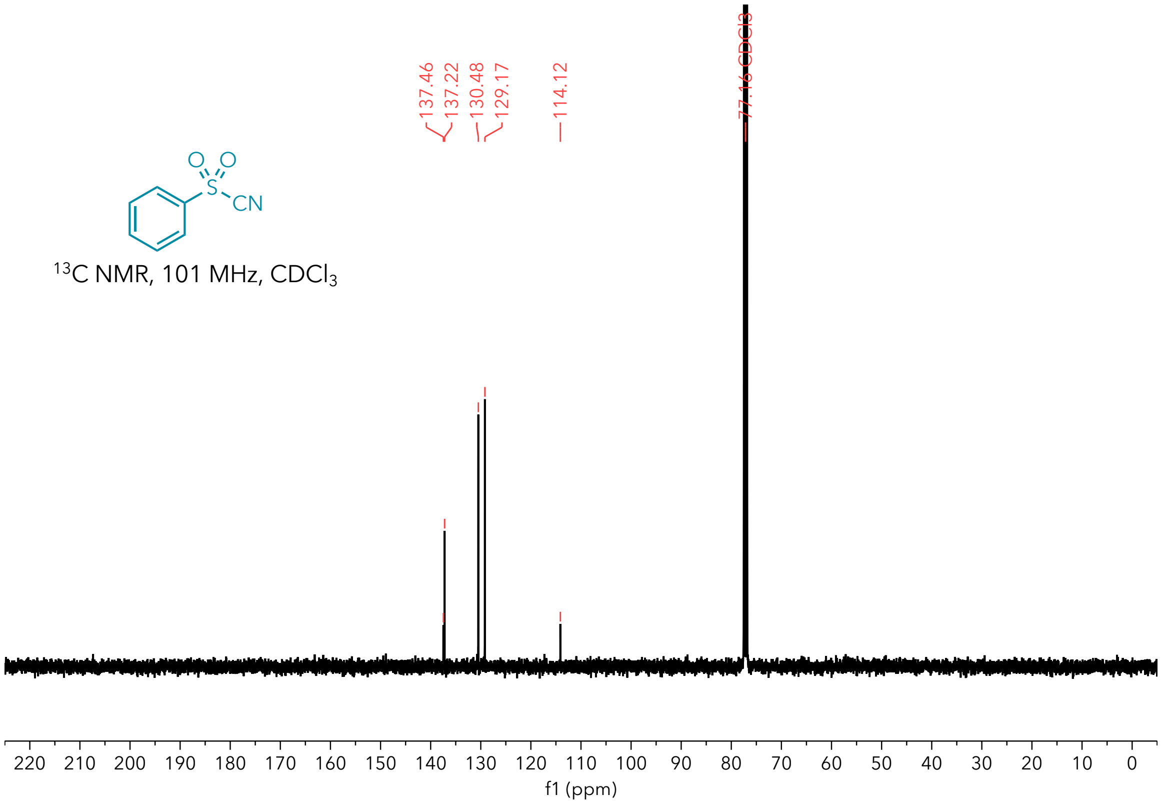

137.46
137.22
130.48
129.17
114.12
77.16 CDCl3
CN
13C NMR, 101 MHz, CDCl3
f1 (ppm)

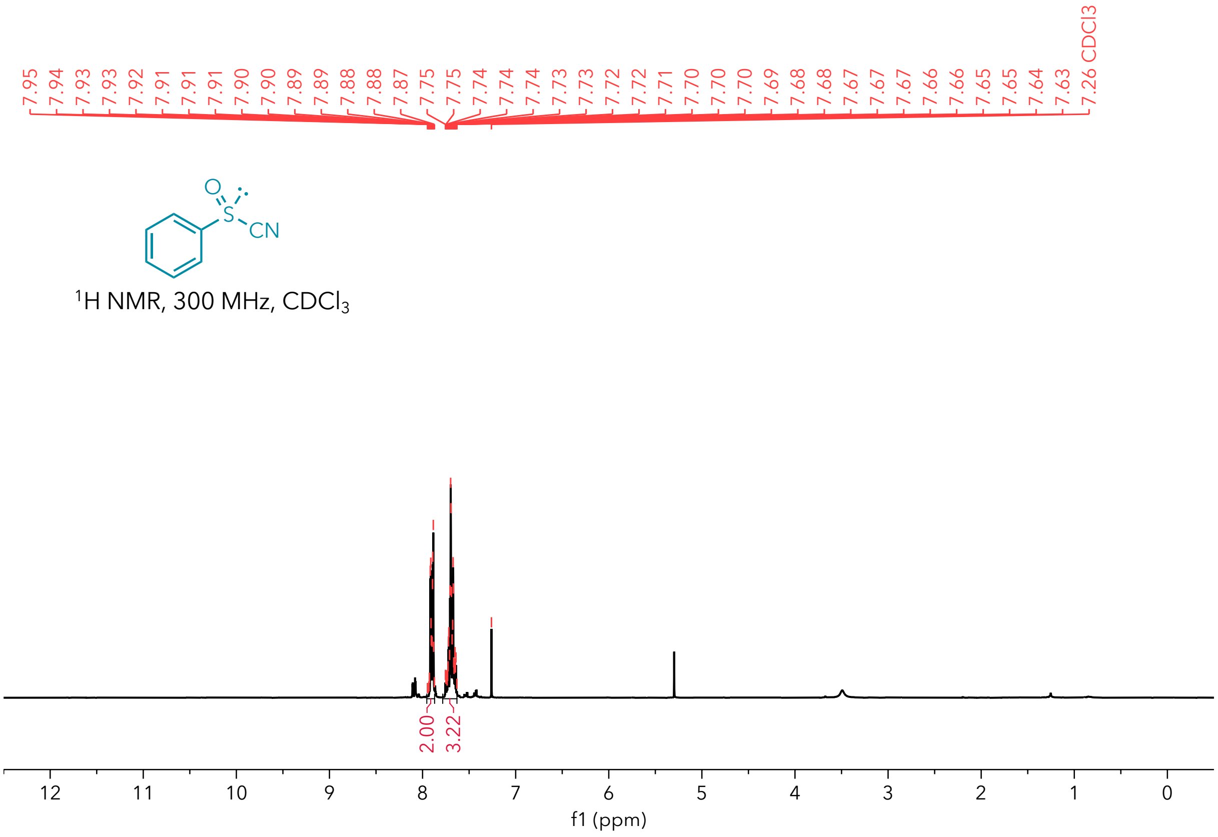
7.95 7.94 7.93 7.93 7.92 7.91 7.91 7.91 7.90 7.90 7.89 7.89 7.88 7.88 7.87 7.75 7.75 7.74 7.74 7.74 7.73 7.73 7.72 7.72 7.71 7.70 7.70 7.70 7.69 7.68 7.68 7.67 7.67 7.67 7.66 7.66 7.65 7.65 7.64 7.63 7.26 CDCl3
CN
^{1}H NMR, 300 MHz, $CDCl_3$
2.00
3.22
f1 (ppm)

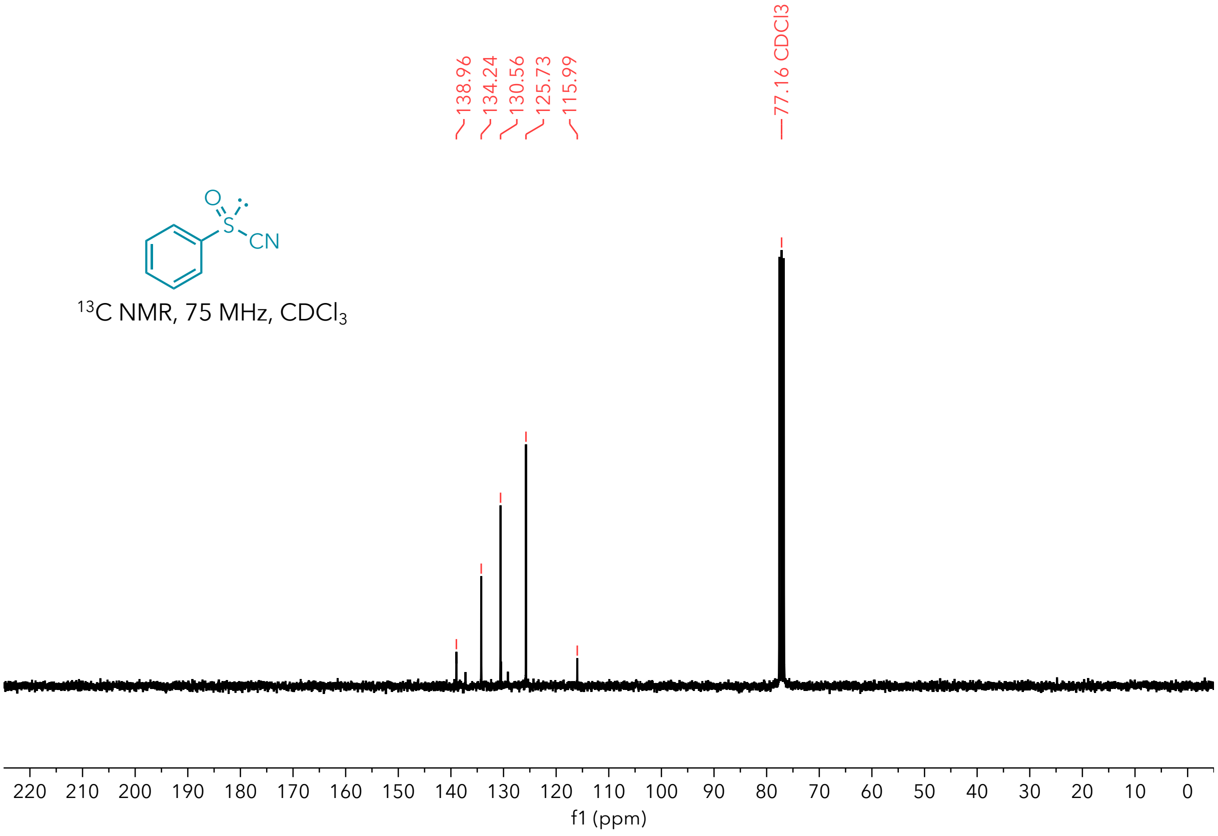
138.96
134.24
130.56
125.73
115.99
77.16 CDCl3
CN
^{13}C NMR, 75 MHz, $CDCl_3$
f1 (ppm)

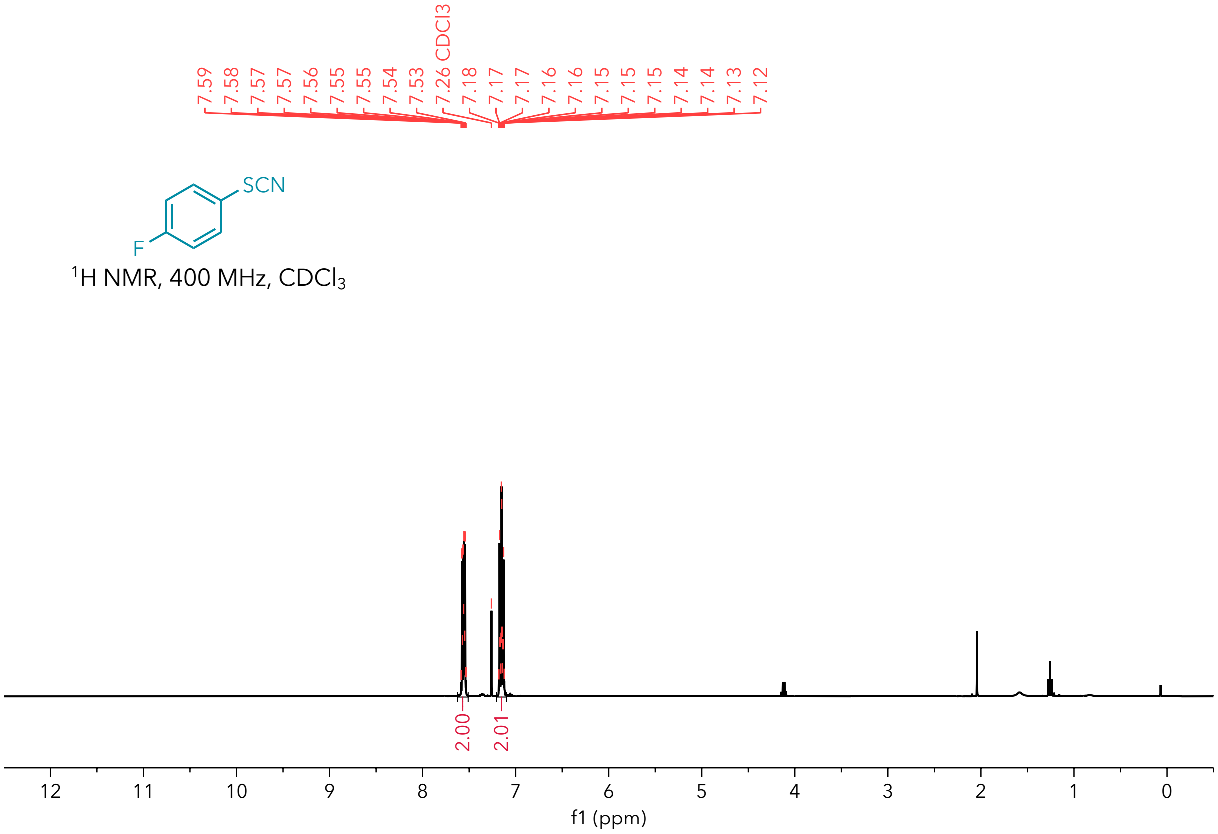
7.59
7.58
7.57
7.57
7.56
7.55
7.55
7.54
7.53
7.26 CDCl3
7.18
7.17
7.17
7.16
7.16
7.15
7.15
7.15
7.14
7.14
7.13
7.12
SCN
F
^{1}H NMR, 400 MHz, CDCl3
2.00
2.01
12
11
10
9
8
7
6
5
4
3
2
1
0
f1 (ppm)

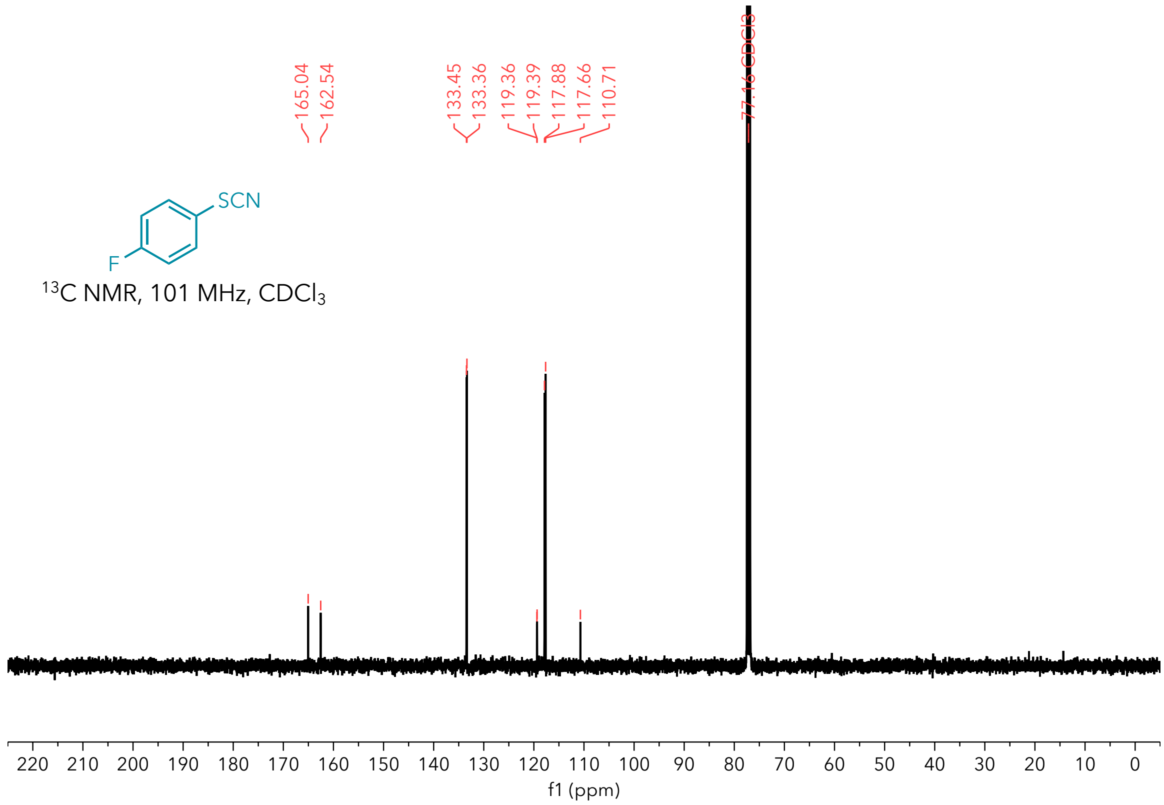
165.04
162.54
133.45
133.36
119.36
119.39
117.88
117.66
110.71
77.16 CDCl3
SCN
F
^{13}C NMR, 101 MHz, CDCl3
220
210
200
190
180
170
160
150
140
130
120
110
100
90
80
70
60
50
40
30
20
10
0
f1 (ppm)

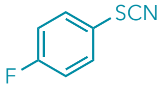

^{19}F NMR, 376 MHz, $CDCl_3$

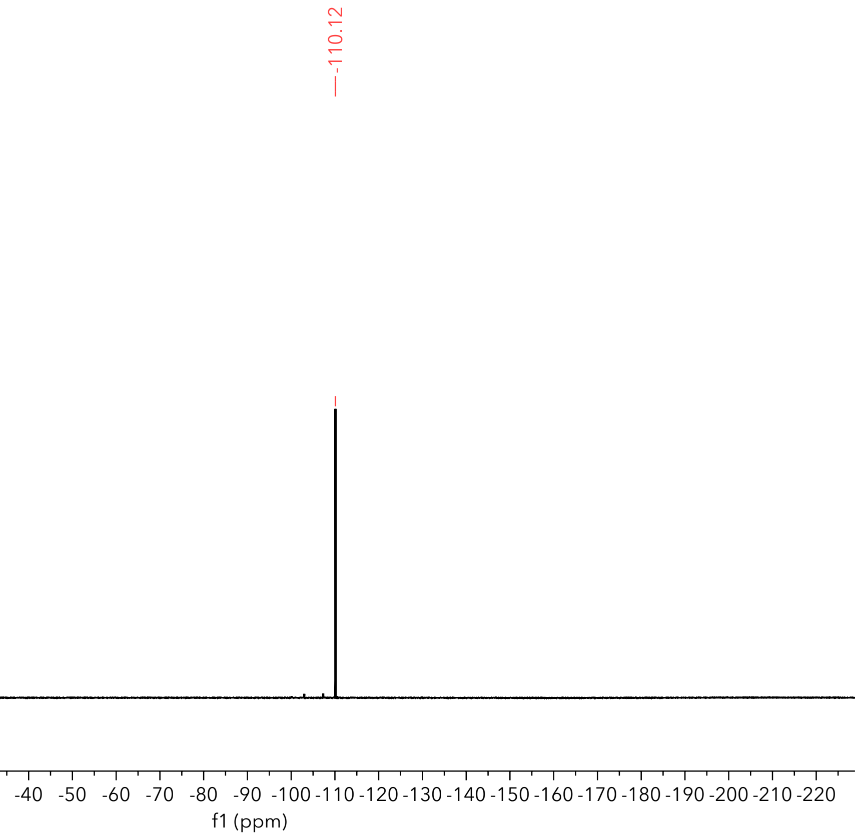

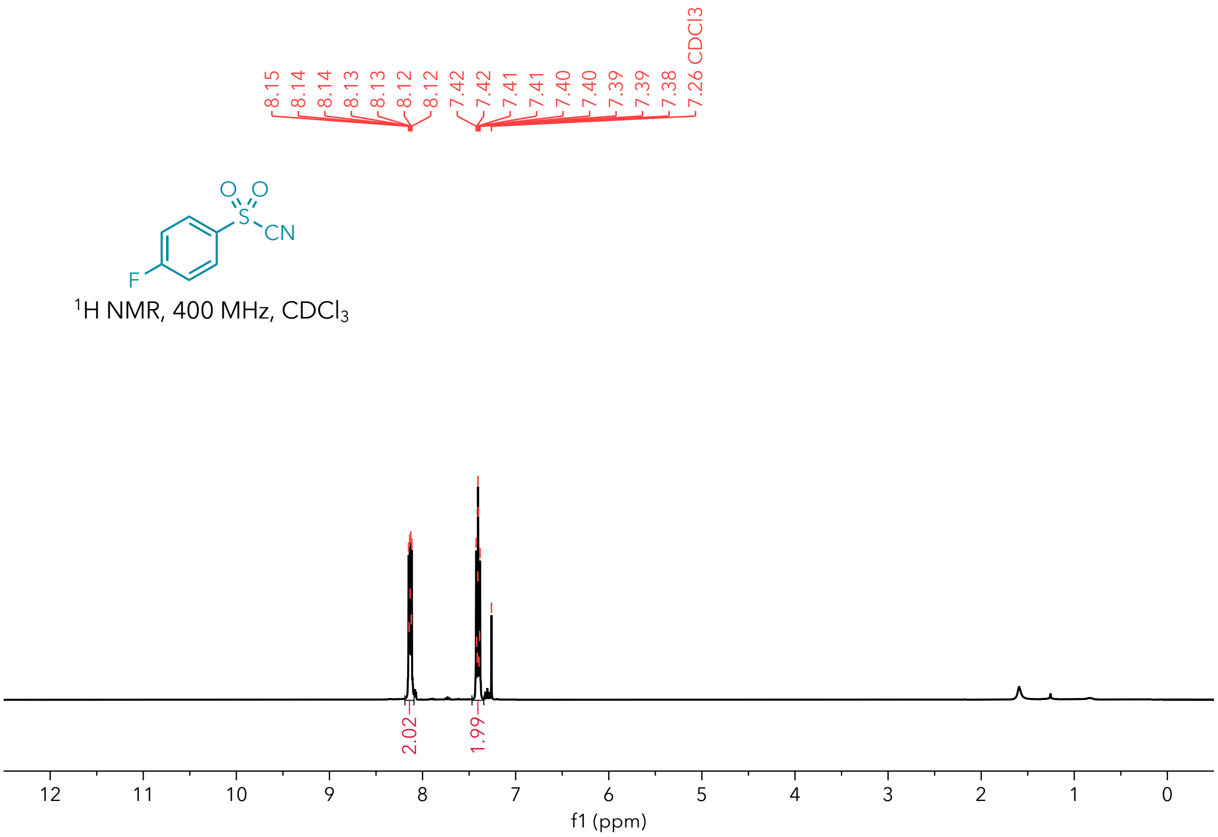

^{1}H NMR, 400 MHz, $CDCl_3$

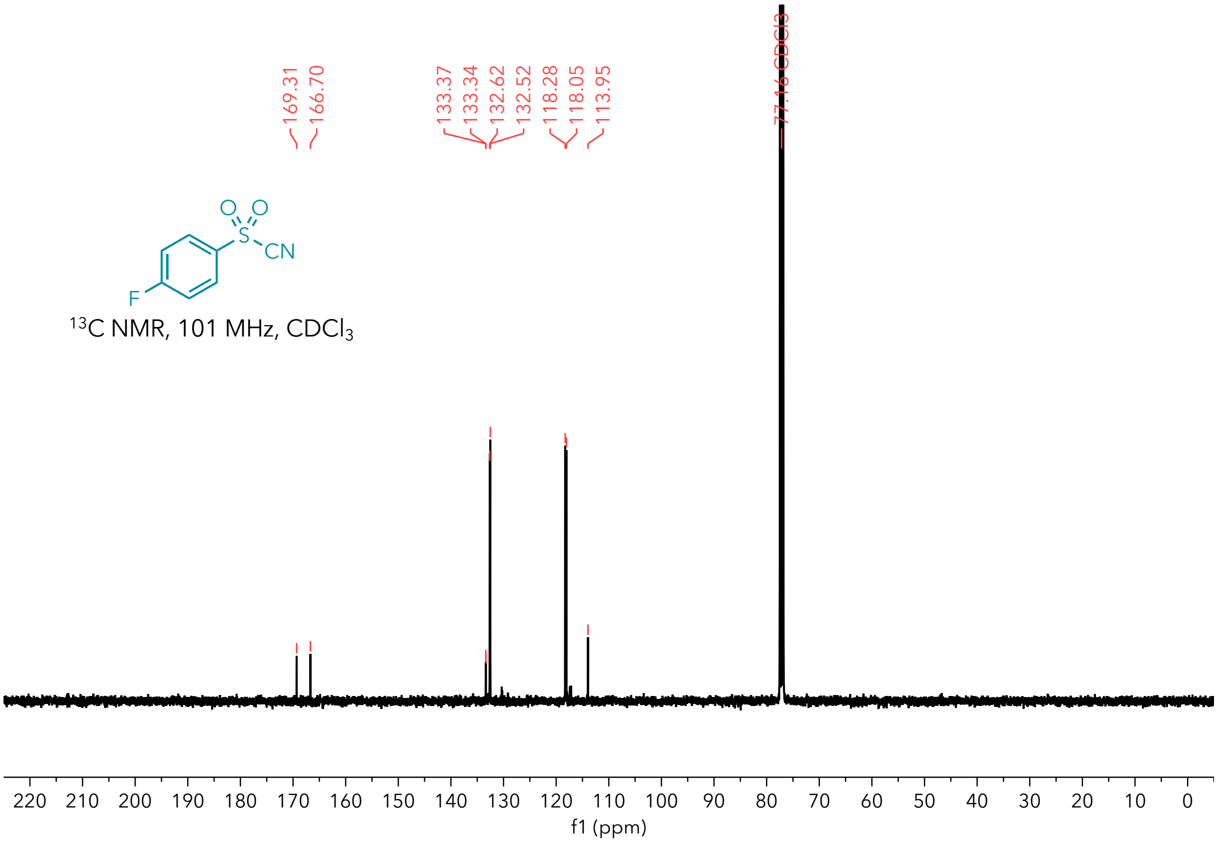

^{13}C NMR, 101 MHz, $CDCl_3$

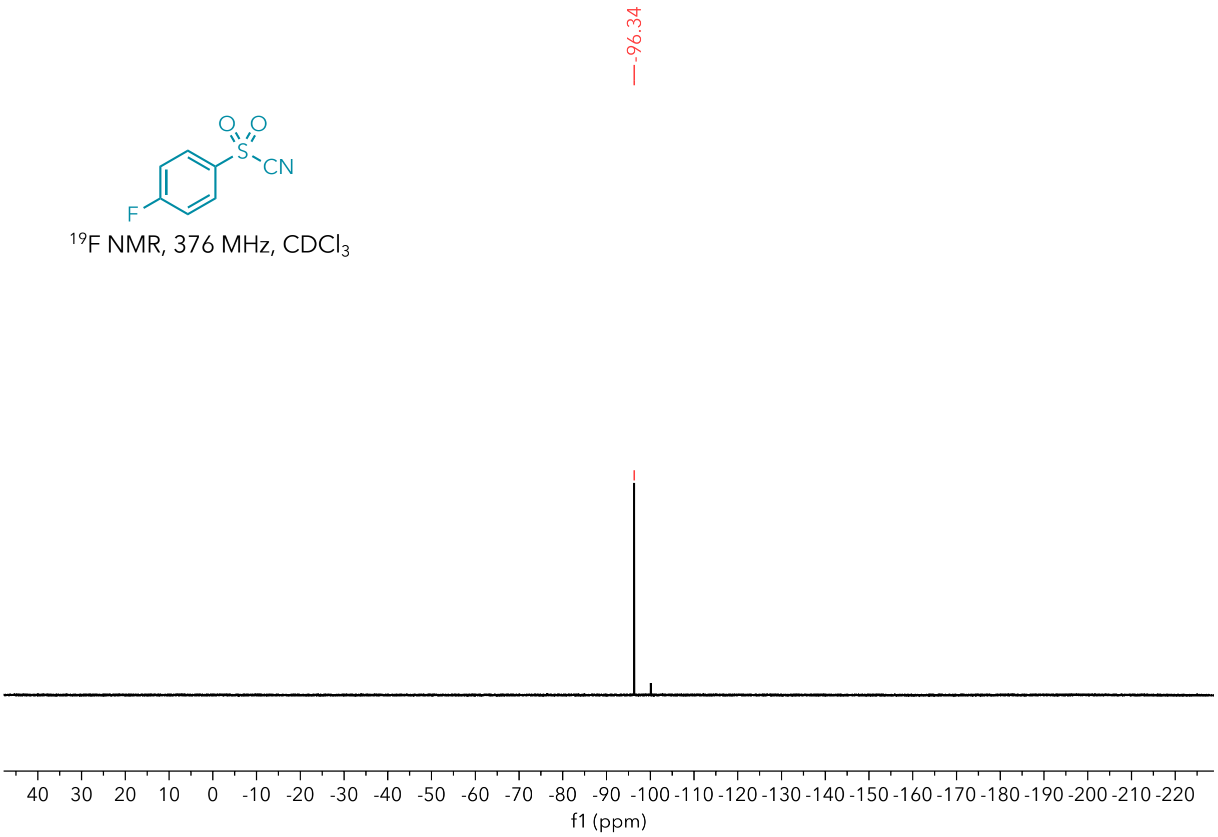
-96.34
F
O
O
S
CN
^{19}F NMR, 376 MHz, $CDCl_3$
40 30 20 10 0 -10 -20 -30 -40 -50 -60 -70 -80 -90 -100 -110 -120 -130 -140 -150 -160 -170 -180 -190 -200 -210 -220
f1 (ppm)

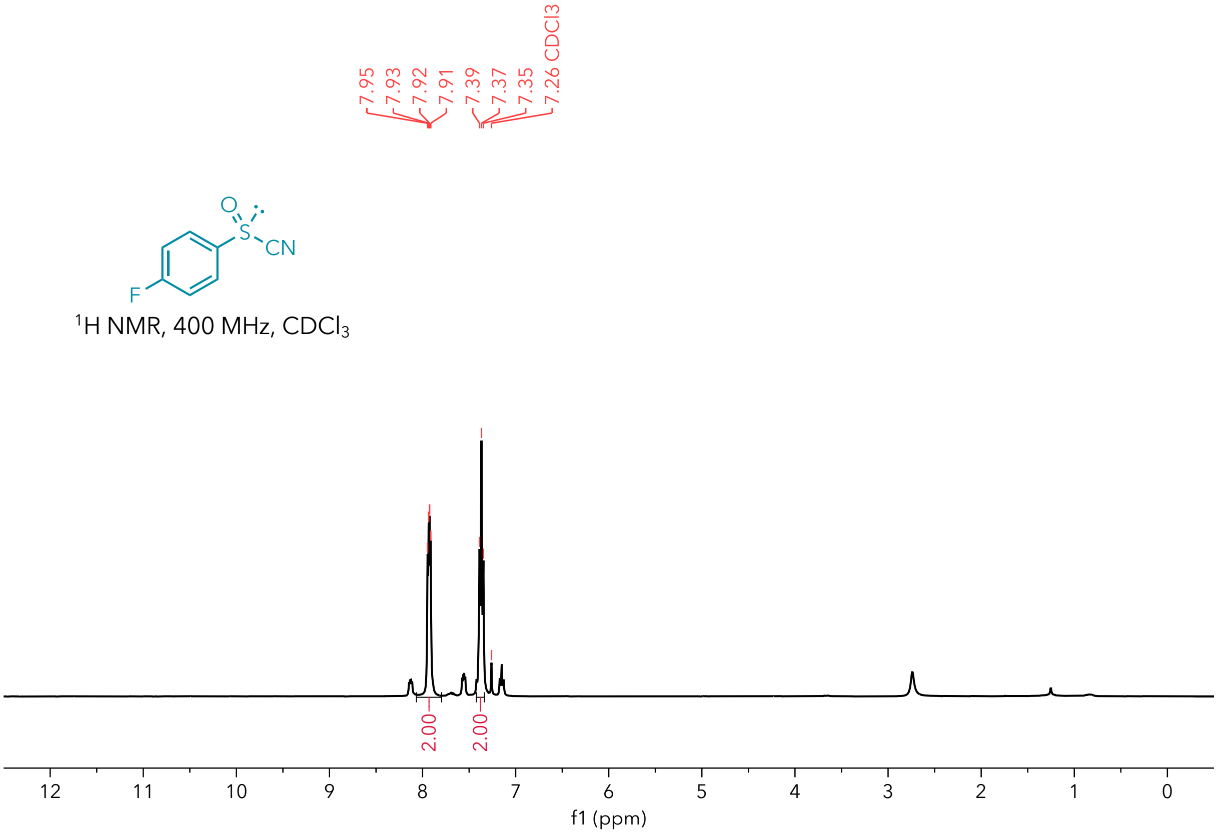

^{1}H NMR, 400 MHz, $CDCl_3$

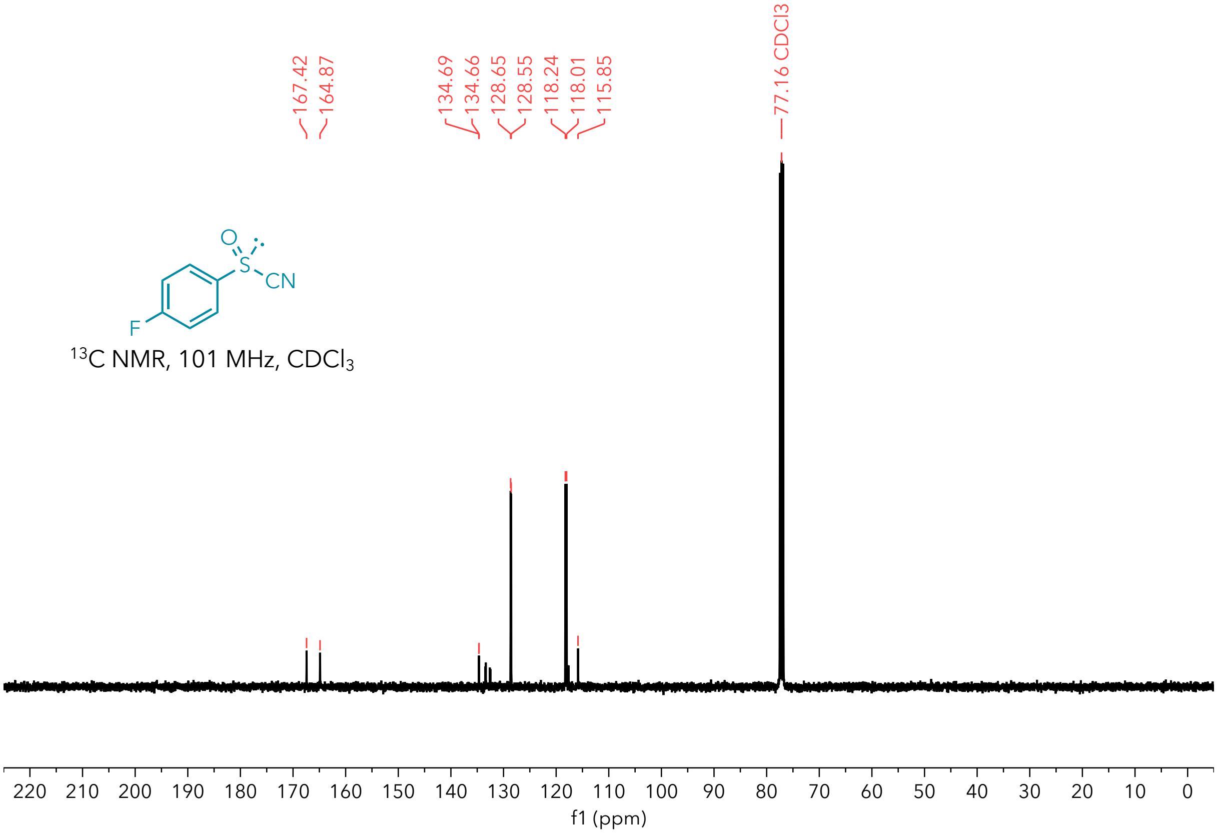

^{13}C NMR, 101 MHz, $CDCl_3$

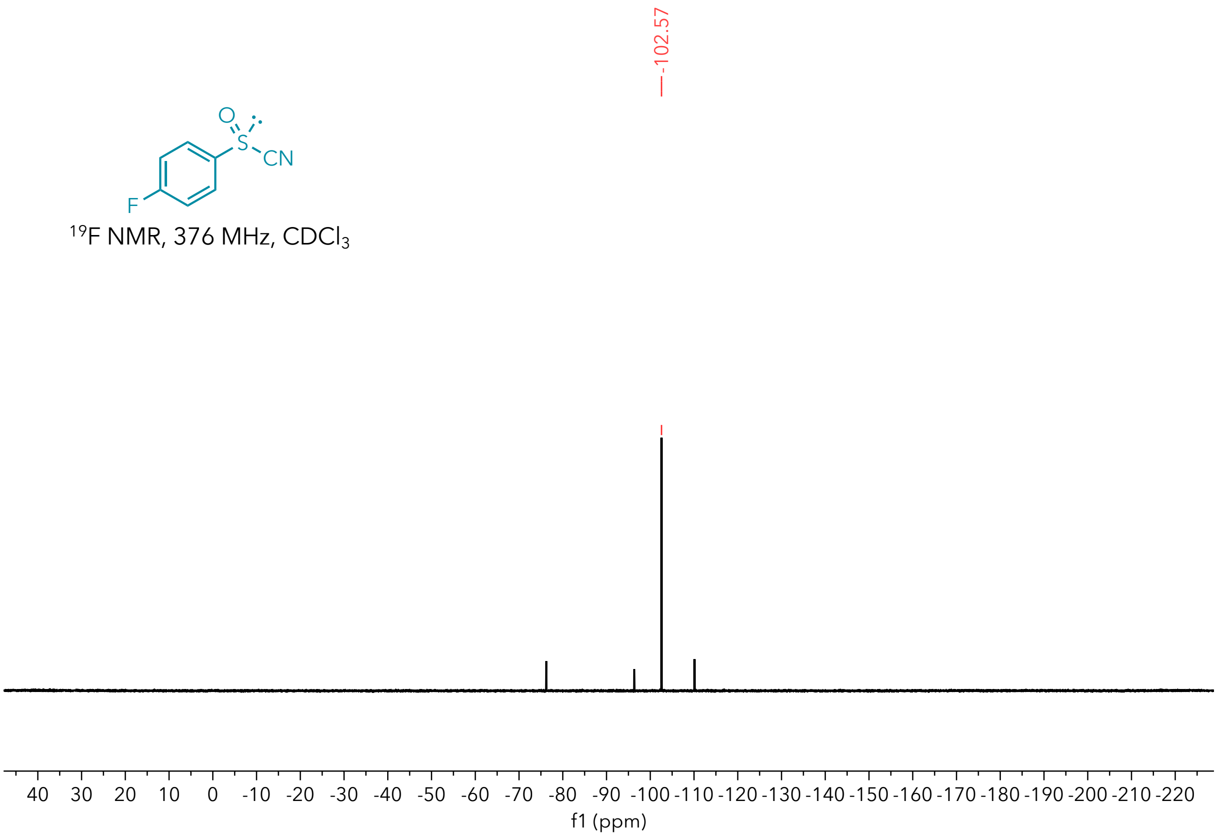

^{19}F NMR, 376 MHz, $CDCl_3$

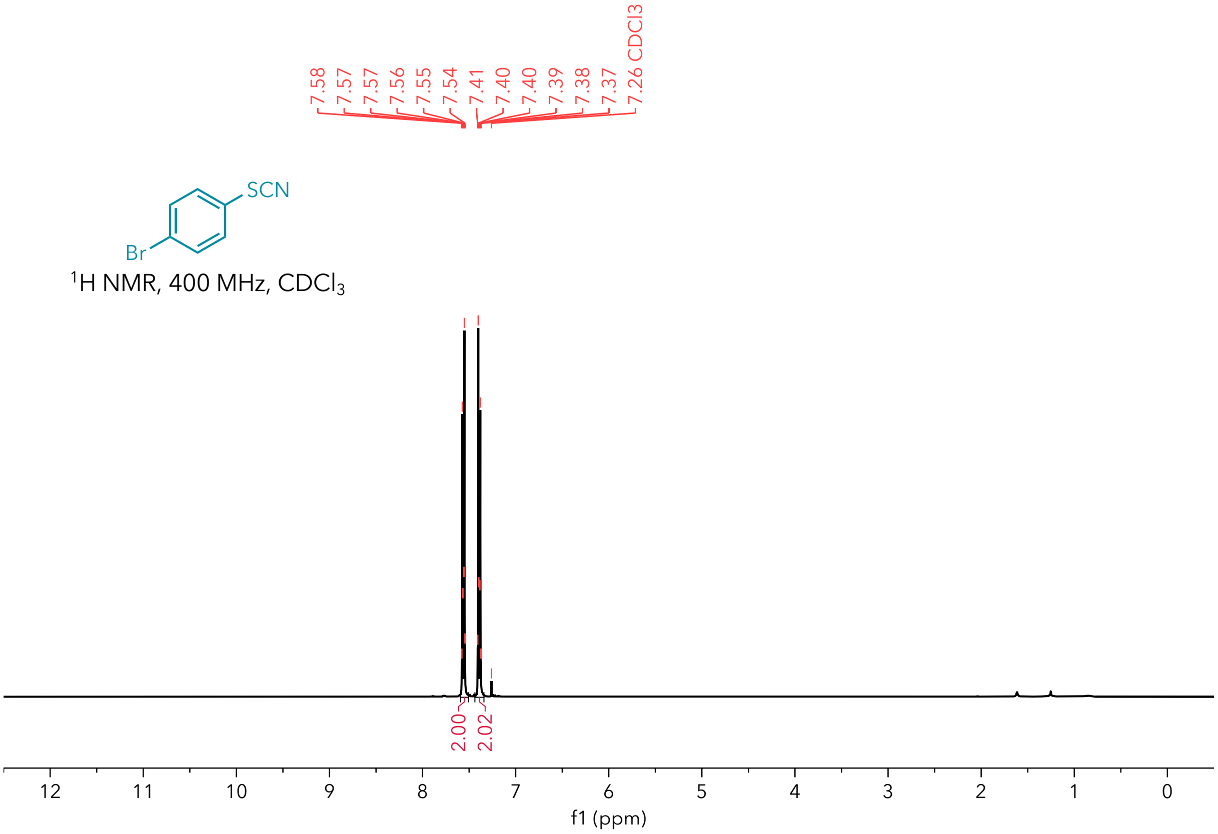

7.58
7.57
7.57
7.56
7.55
7.54
7.41
7.40
7.40
7.39
7.38
7.37
7.26 CDCl3
SCN
Br
^{1}H NMR, 400 MHz, $CDCl_3$
2.00
2.02
12 11 10 9 8 7 6 5 4 3 2 1 0
f1 (ppm)

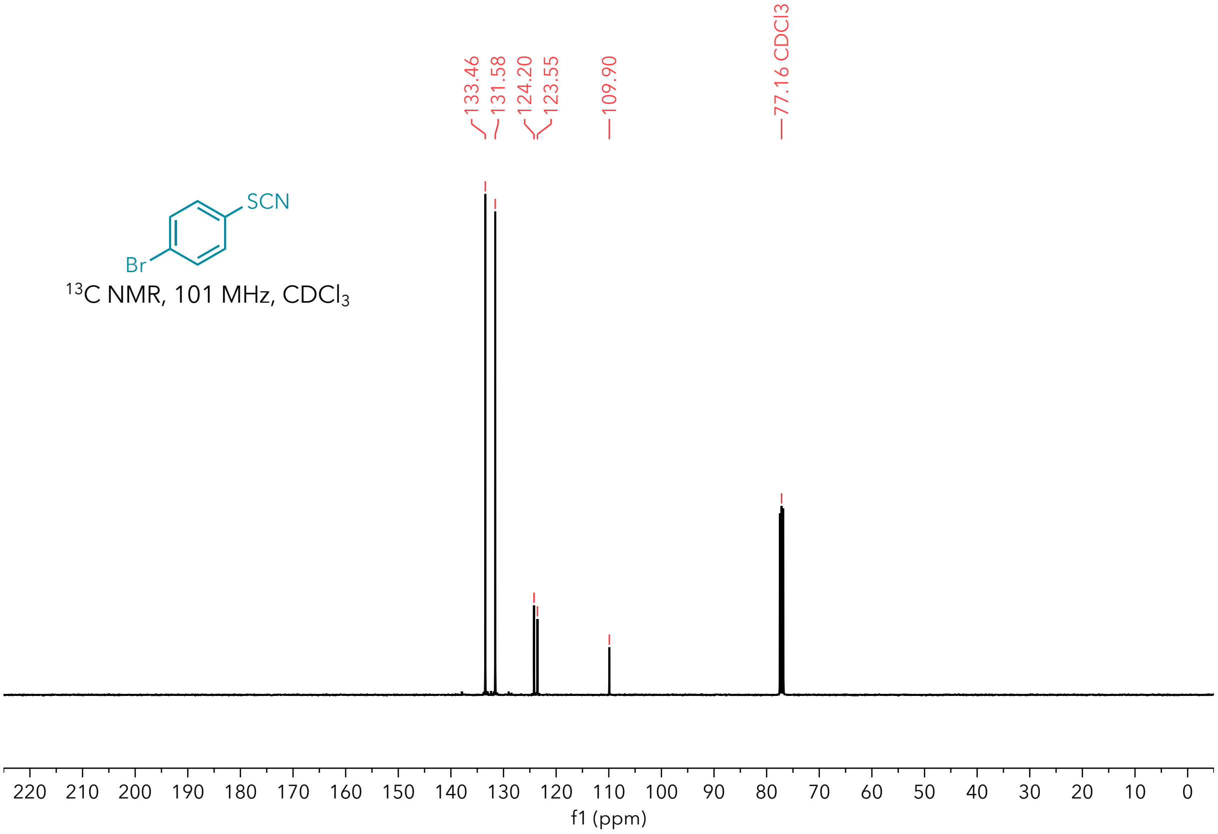

133.46
131.58
124.20
123.55
109.90
77.16 CDCl3
SCN
Br
^{13}C NMR, 101 MHz, $CDCl_3$
220 210 200 190 180 170 160 150 140 130 120 110 100 90 80 70 60 50 40 30 20 10 0
f1 (ppm)

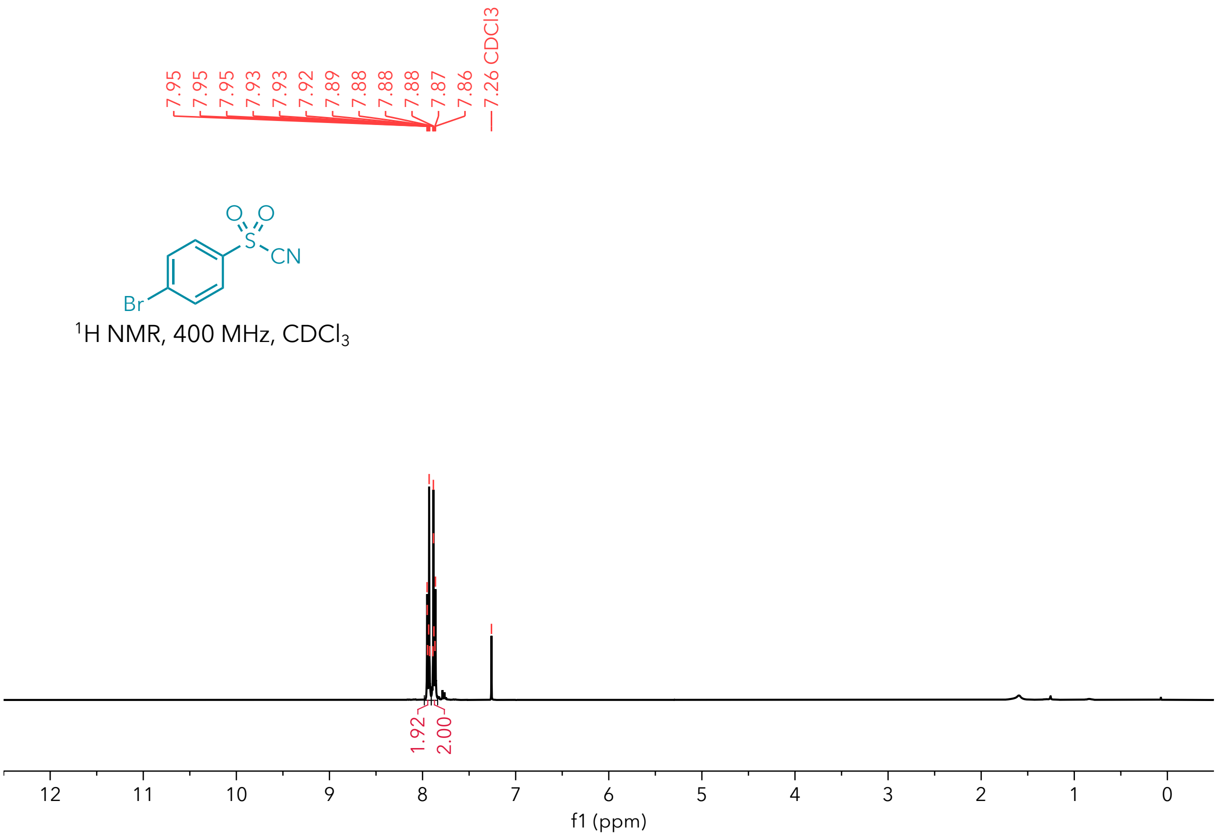
7.95
7.95
7.95
7.93
7.93
7.92
7.89
7.88
7.88
7.88
7.87
7.86
7.26 CDCl3
O O
S
CN
Br
^{1}H NMR, 400 MHz, $CDCl_3$
1.92
2.00
12 11 10 9 8 7 6 5 4 3 2 1 0
f1 (ppm)

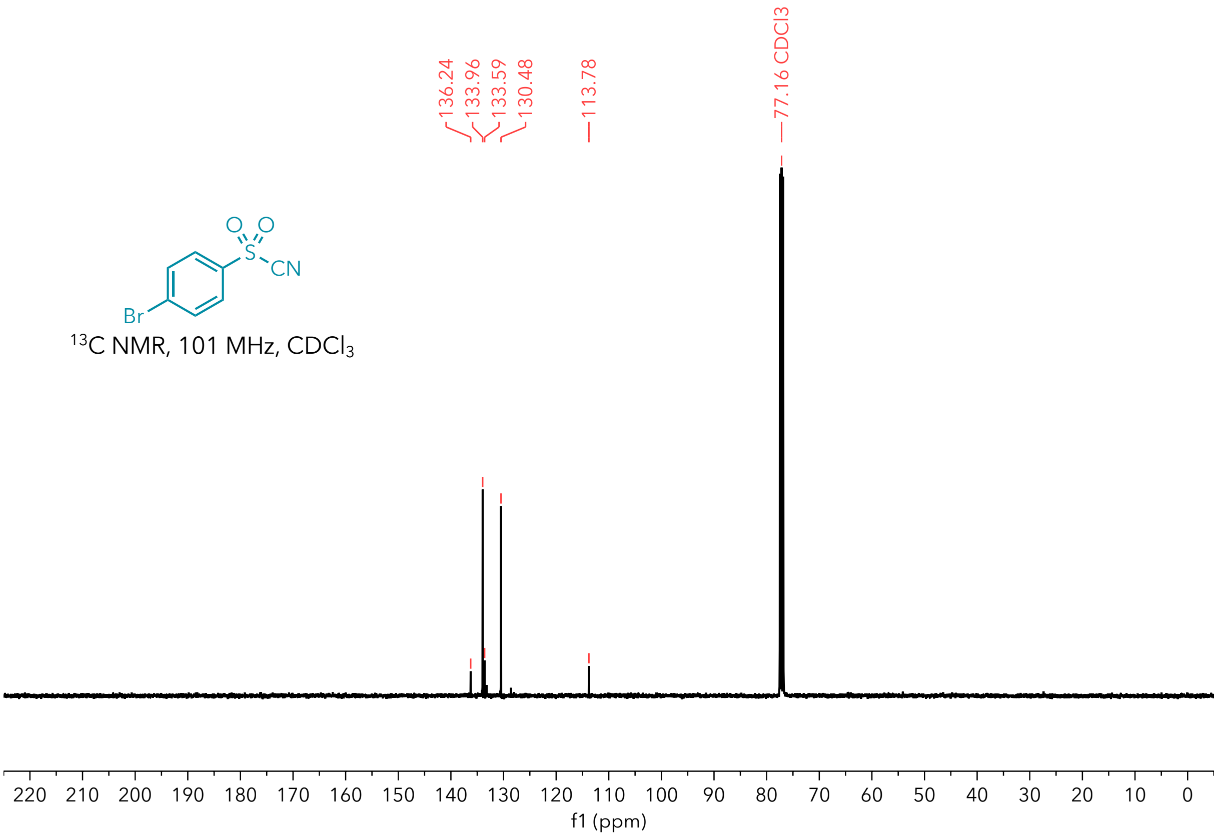
136.24
133.96
133.59
130.48
113.78
77.16 CDCl3
O O
S
CN
Br
^{13}C NMR, 101 MHz, $CDCl_3$
220 210 200 190 180 170 160 150 140 130 120 110 100 90 80 70 60 50 40 30 20 10 0
f1 (ppm)

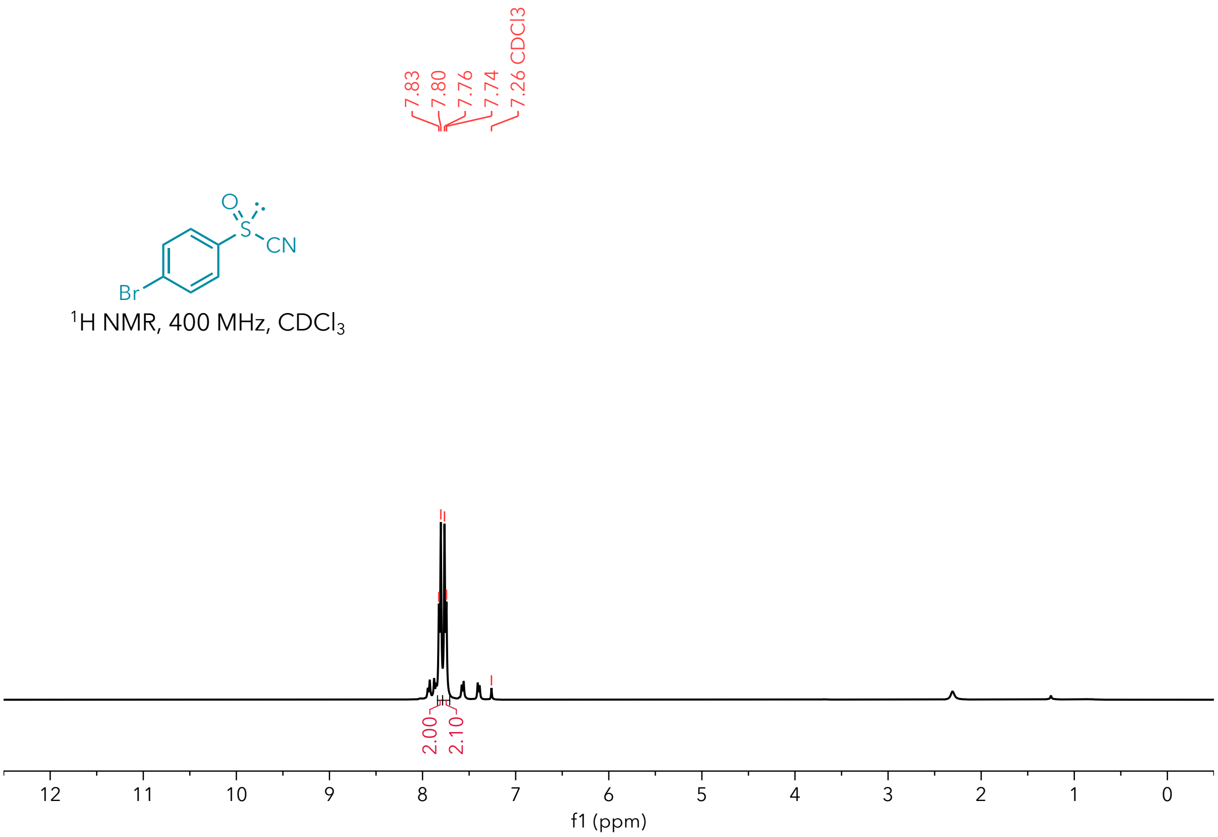
7.83
7.80
7.76
7.74
7.26 CDCl3
O
S
CN
Br
1H NMR, 400 MHz, CDCl3
2.00
2.10
12 11 10 9 8 7 6 5 4 3 2 1 0
f1 (ppm)

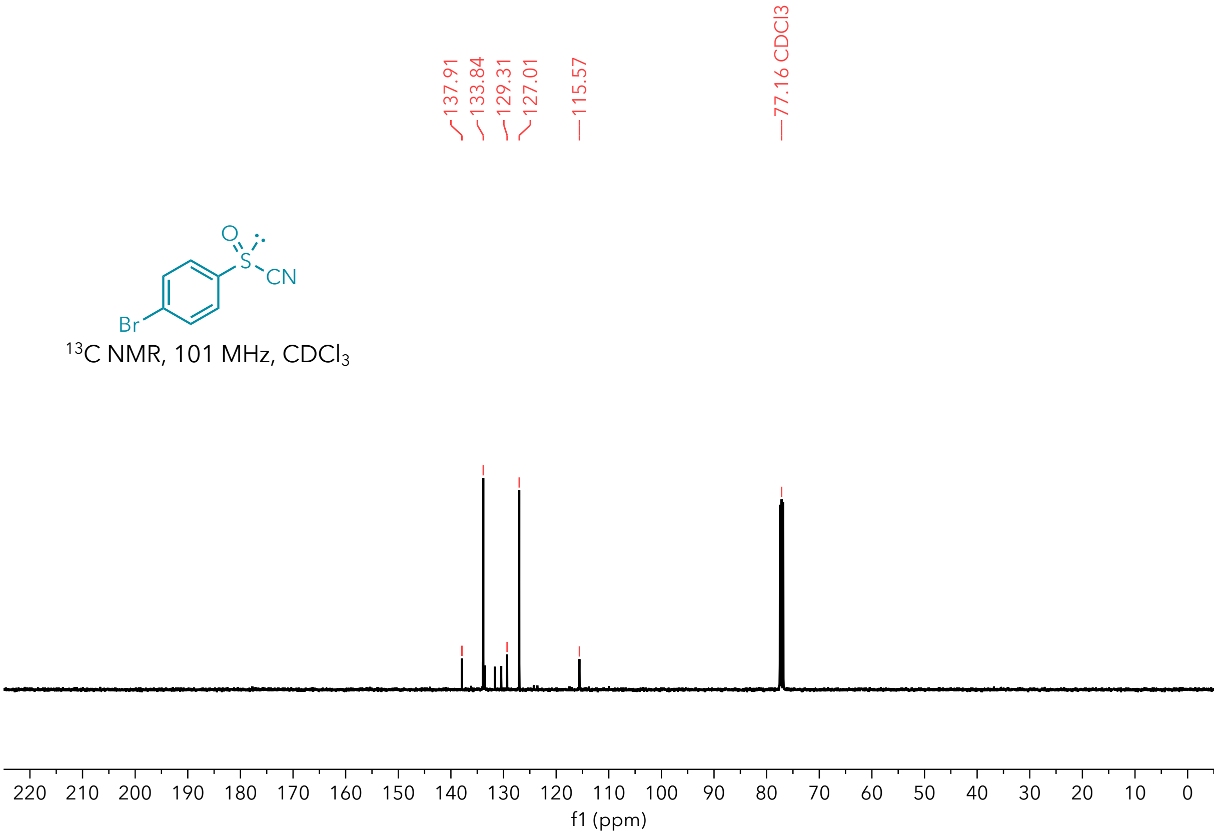
137.91
133.84
129.31
127.01
115.57
77.16 CDCl3
O
S
CN
Br
13C NMR, 101 MHz, CDCl3
220 210 200 190 180 170 160 150 140 130 120 110 100 90 80 70 60 50 40 30 20 10 0
f1 (ppm)

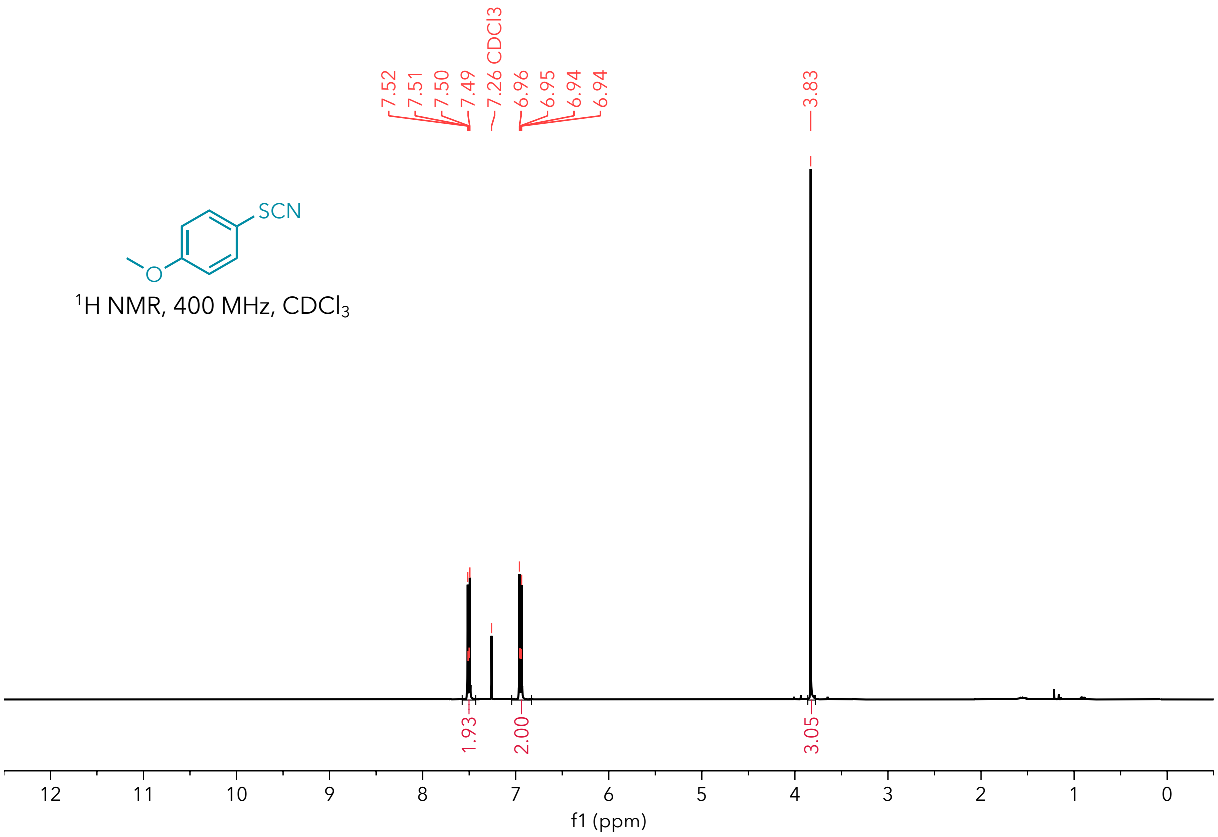
7.52
7.51
7.50
7.49
7.26 CDCl3
6.96
6.95
6.94
6.94
3.83
SCN
O
1H NMR, 400 MHz, CDCl3
1.93
2.00
3.05
12
11
10
9
8
7
6
5
4
3
2
1
0
f1 (ppm)

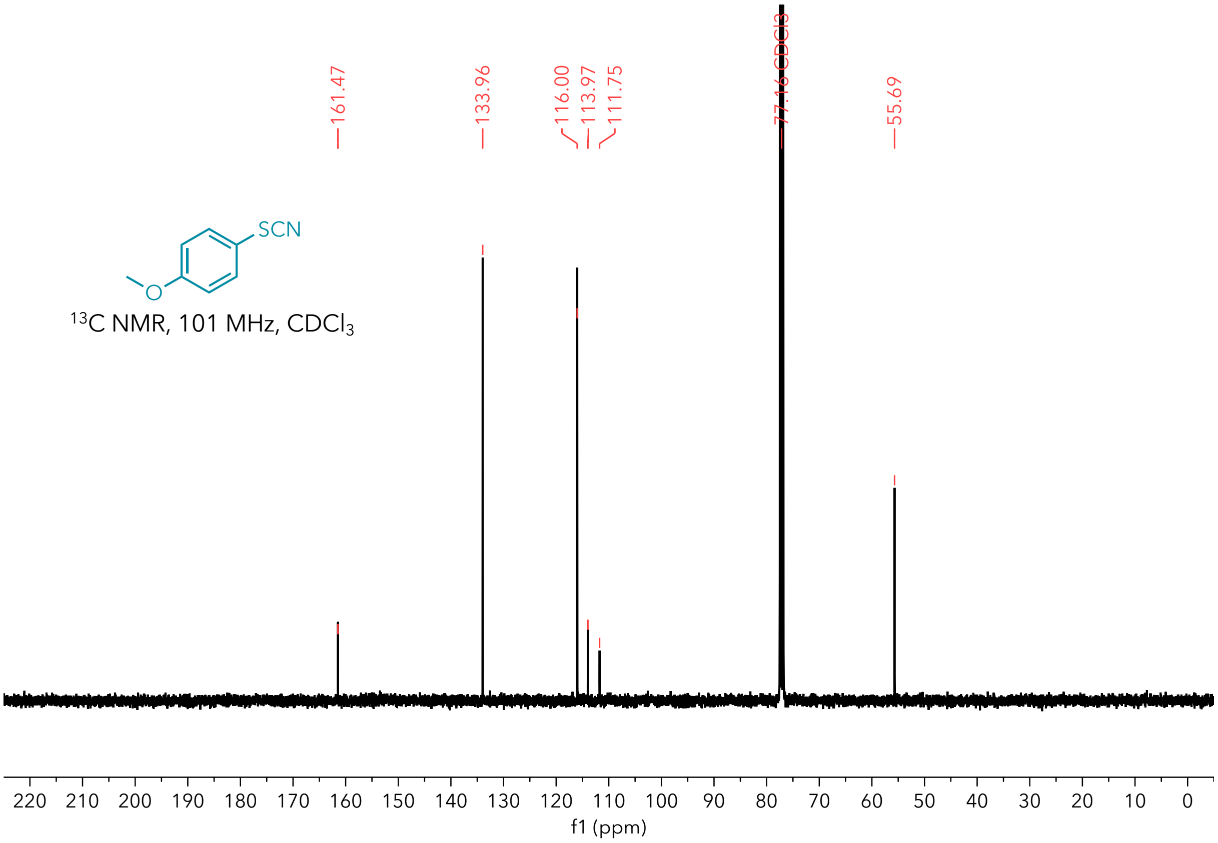
161.47
133.96
116.00
113.97
111.75
77.16 CDCl3
55.69
SCN
O
13C NMR, 101 MHz, CDCl3
220
210
200
190
180
170
160
150
140
130
120
110
100
90
80
70
60
50
40
30
20
10
0
f1 (ppm)

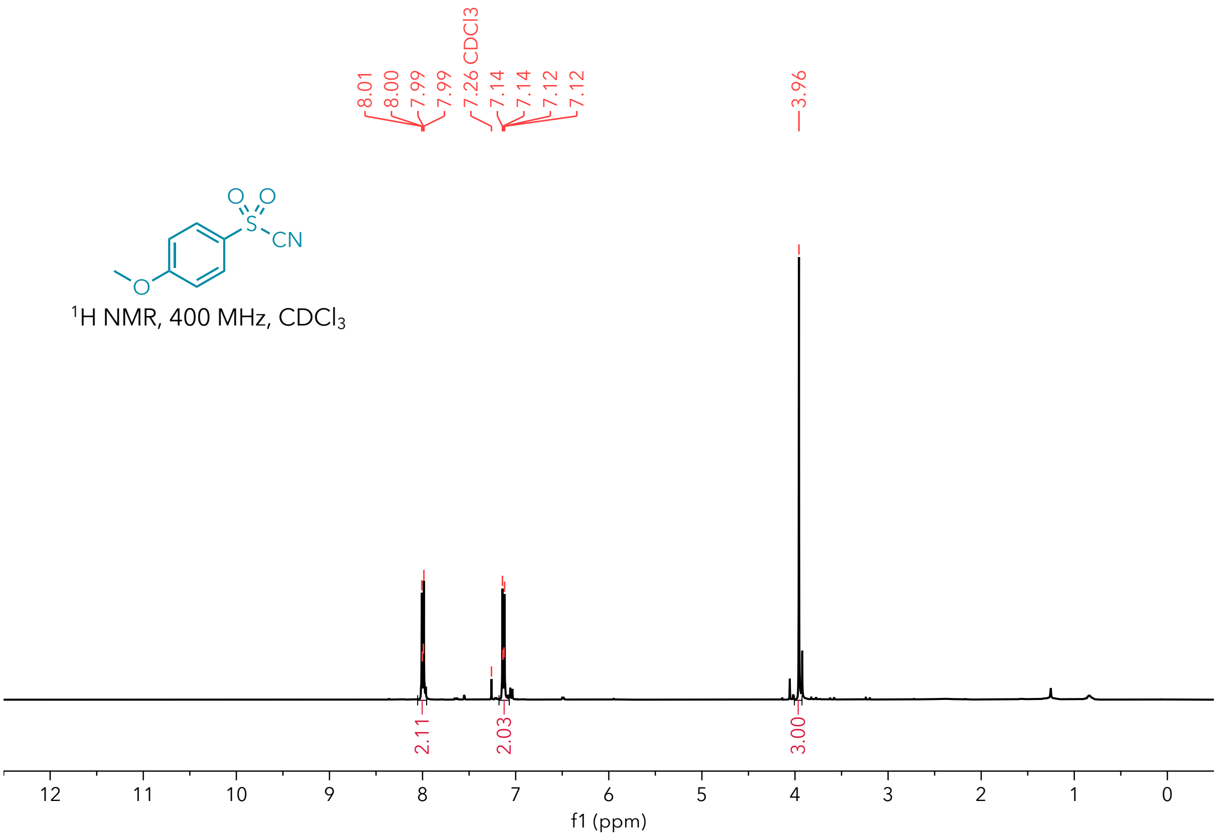
8.01
8.00
7.99
7.99
7.26 CDCl3
7.14
7.14
7.12
7.12
3.96
O O
S
CN
O
1H NMR, 400 MHz, CDCl3
2.11
2.03
3.00
12 11 10 9 8 7 6 5 4 3 2 1 0
f1 (ppm)

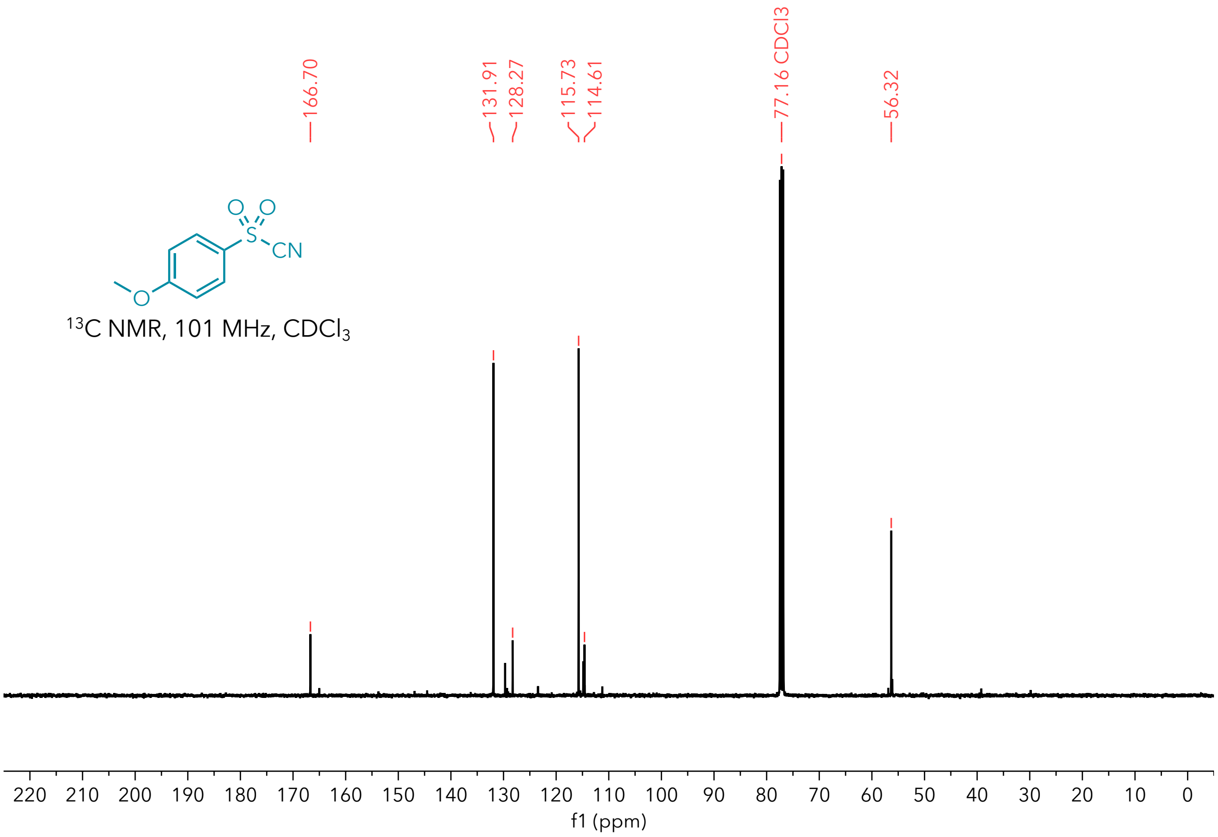
166.70
131.91
128.27
115.73
114.61
77.16 CDCl3
56.32
O O
S
CN
O
13C NMR, 101 MHz, CDCl3
220 210 200 190 180 170 160 150 140 130 120 110 100 90 80 70 60 50 40 30 20 10 0
f1 (ppm)

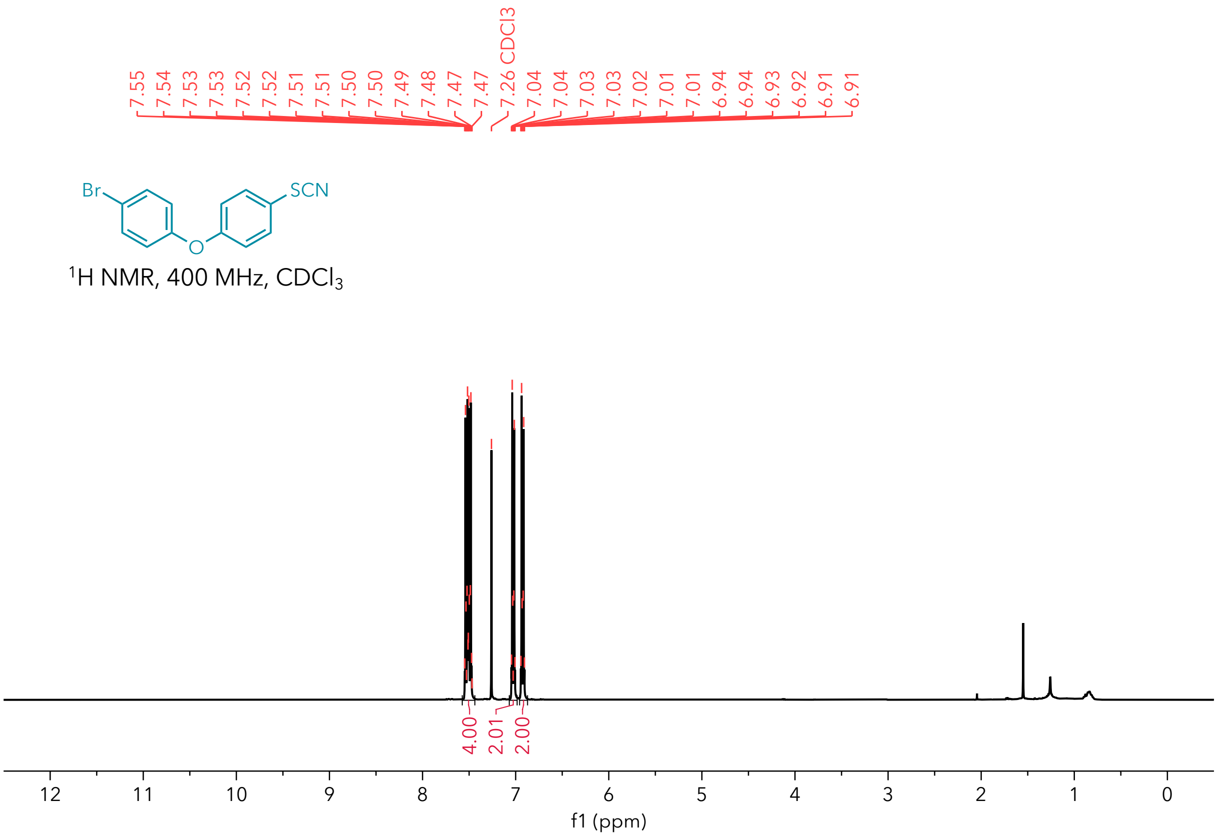

^{1}H NMR, 400 MHz, $CDCl_3$

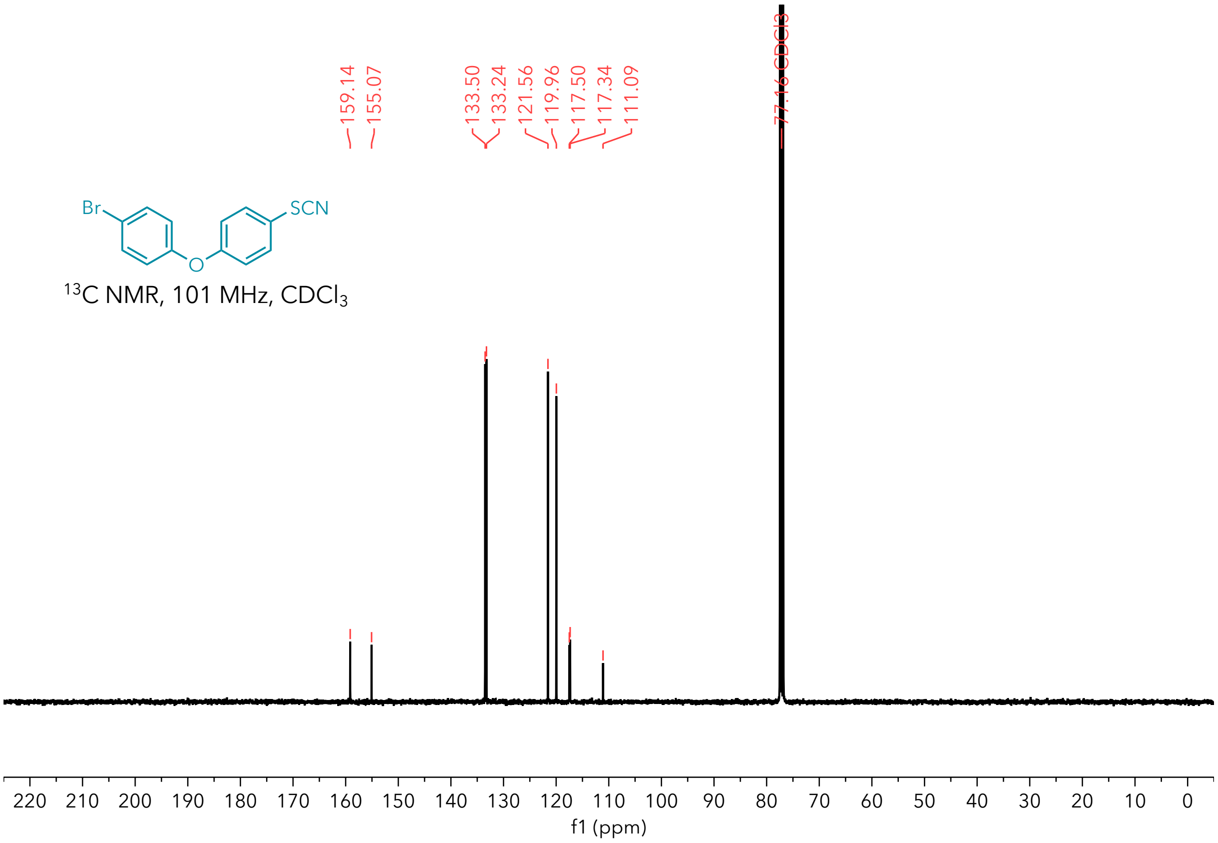

^{13}C NMR, 101 MHz, $CDCl_3$

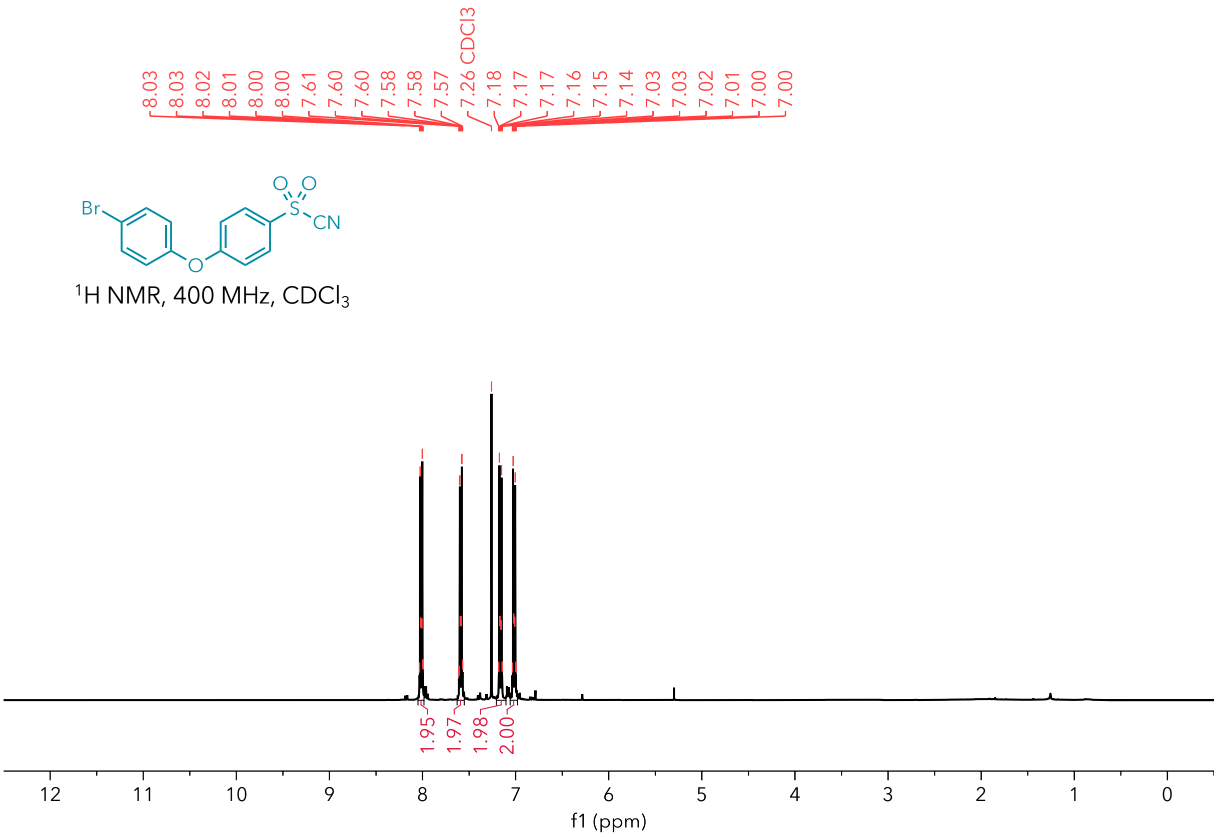
8.03 8.03 8.02 8.01 8.00 8.00 7.61 7.60 7.60 7.58 7.58 7.57 7.26 CDCl3 7.18 7.17 7.17 7.16 7.15 7.14 7.03 7.03 7.02 7.01 7.00 7.00
Br
O O
S
CN
O
1H NMR, 400 MHz, CDCl3
1.95 1.97 1.98 2.00
12 11 10 9 8 7 6 5 4 3 2 1 0
f1 (ppm)

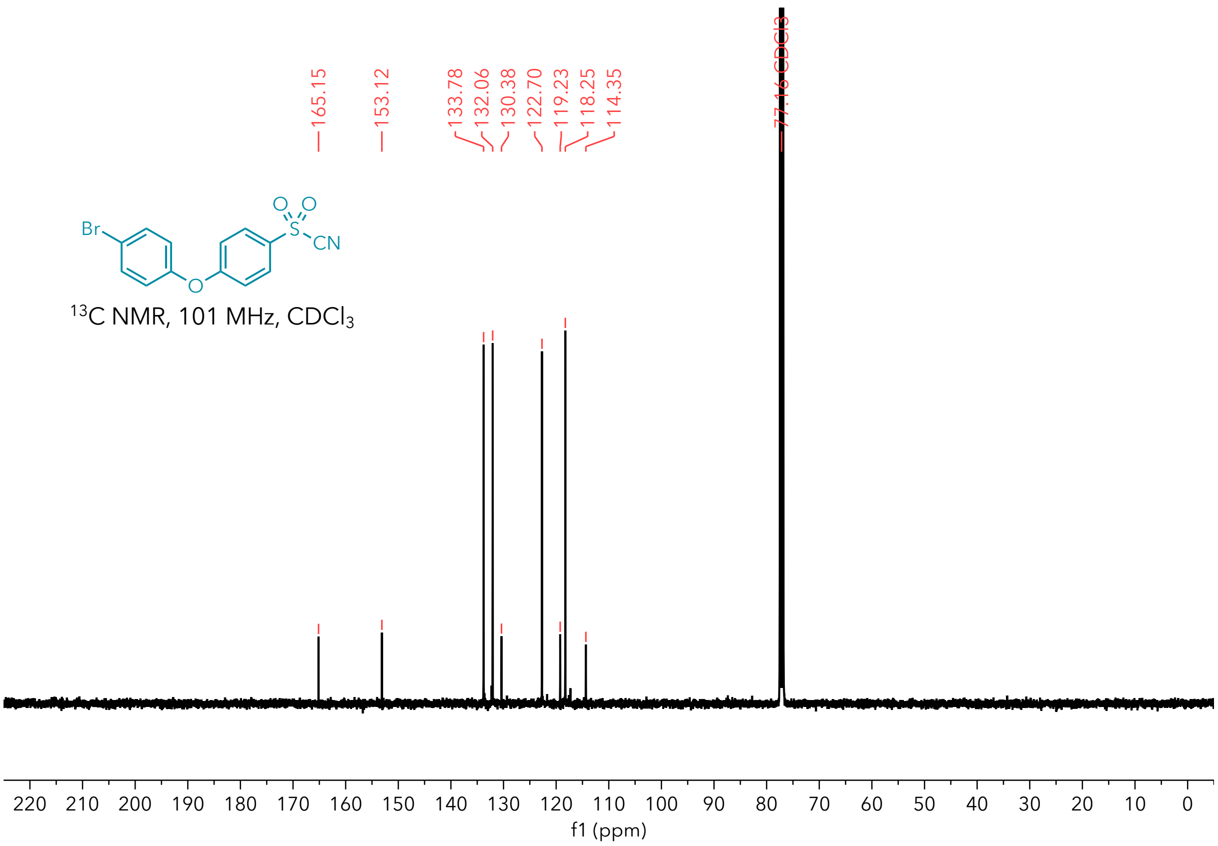
165.15
153.12
133.78 132.06 130.38 122.70 119.23 118.25 114.35
77.16 CDCl3
Br
O O
S
CN
O
13C NMR, 101 MHz, CDCl3
220 210 200 190 180 170 160 150 140 130 120 110 100 90 80 70 60 50 40 30 20 10 0
f1 (ppm)

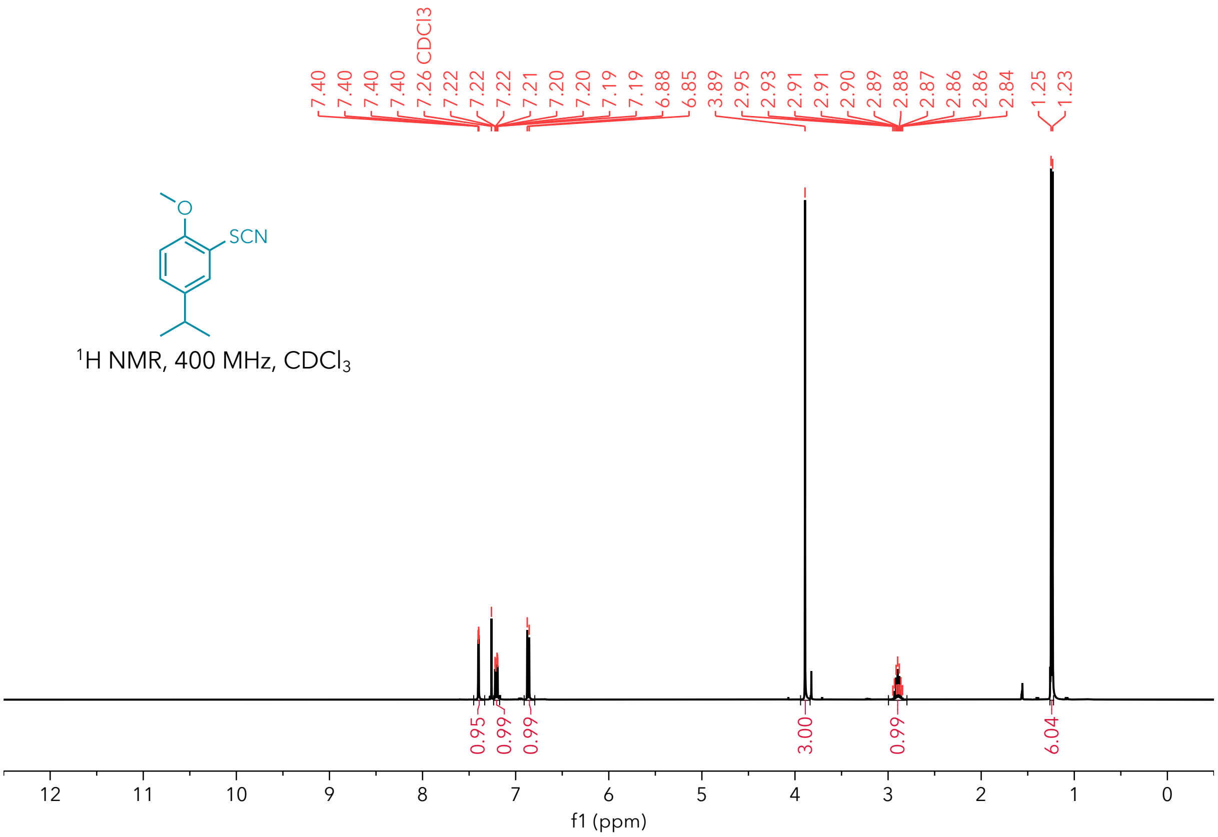
7.40
7.40
7.40
7.40
7.26 CDCl3
7.22
7.22
7.22
7.21
7.20
7.20
7.19
7.19
6.88
6.85
3.89
2.95
2.93
2.91
2.91
2.90
2.89
2.88
2.87
2.86
2.86
2.84
1.25
1.23
SCN
O
1H NMR, 400 MHz, CDCl3
0.95
0.99
0.99
3.00
0.99
6.04
12
11
10
9
8
7
6
5
4
3
2
1
0
f1 (ppm)

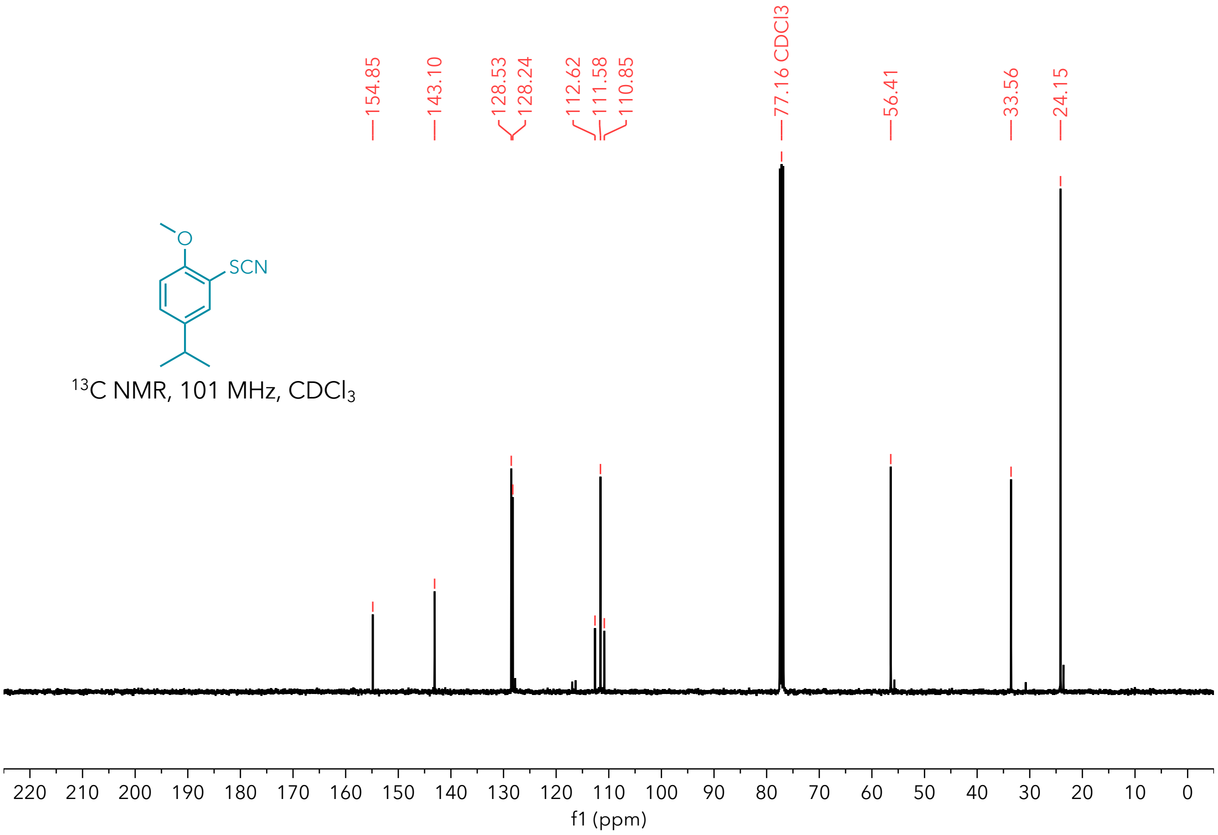
154.85
143.10
128.53
128.24
112.62
111.58
110.85
77.16 CDCl3
56.41
33.56
24.15
SCN
O
13C NMR, 101 MHz, CDCl3
220
210
200
190
180
170
160
150
140
130
120
110
100
90
80
70
60
50
40
30
20
10
0
f1 (ppm)

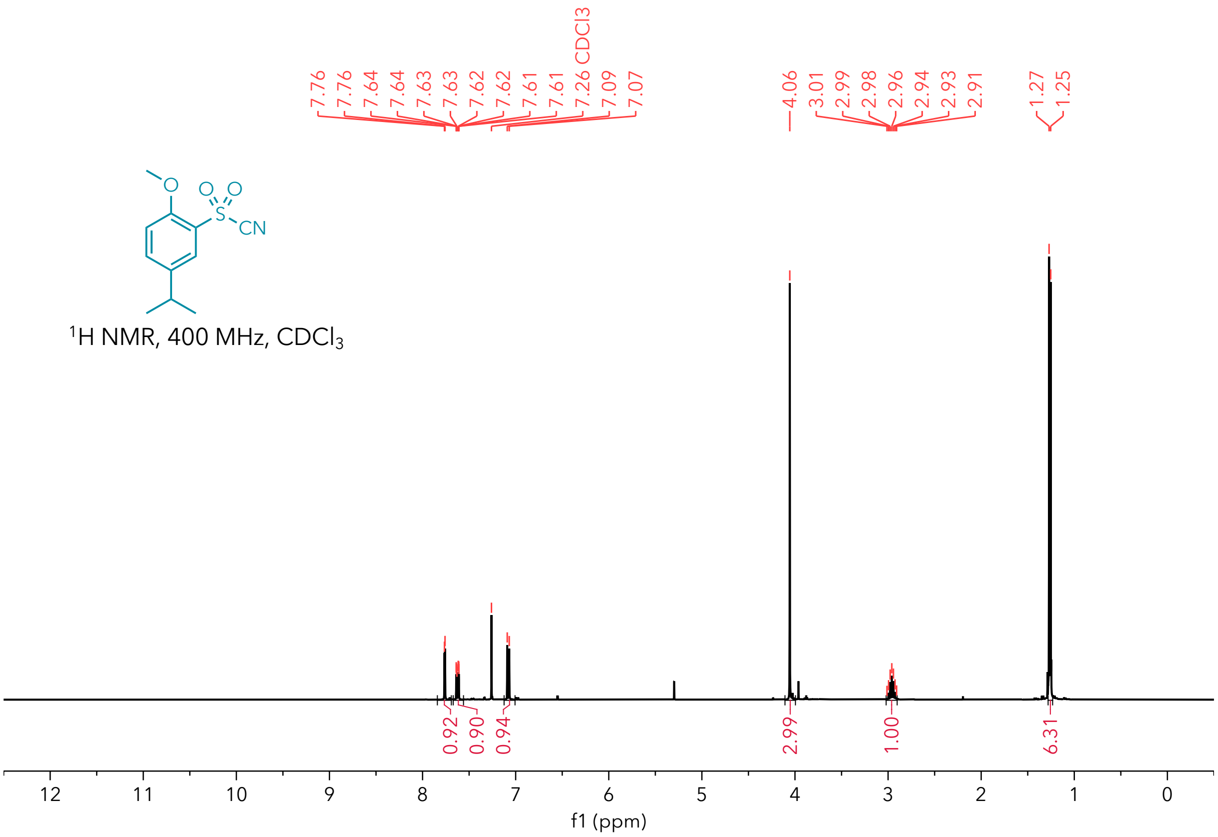
1H NMR, 400 MHz, CDCl3
7.76 7.76 7.64 7.64 7.63 7.63 7.62 7.62 7.61 7.61 7.26 CDCl3 7.09 7.07
4.06 3.01 2.99 2.98 2.96 2.94 2.93 2.91 1.27 1.25
0.92 0.90 0.94 2.99 1.00 6.31
f1 (ppm)

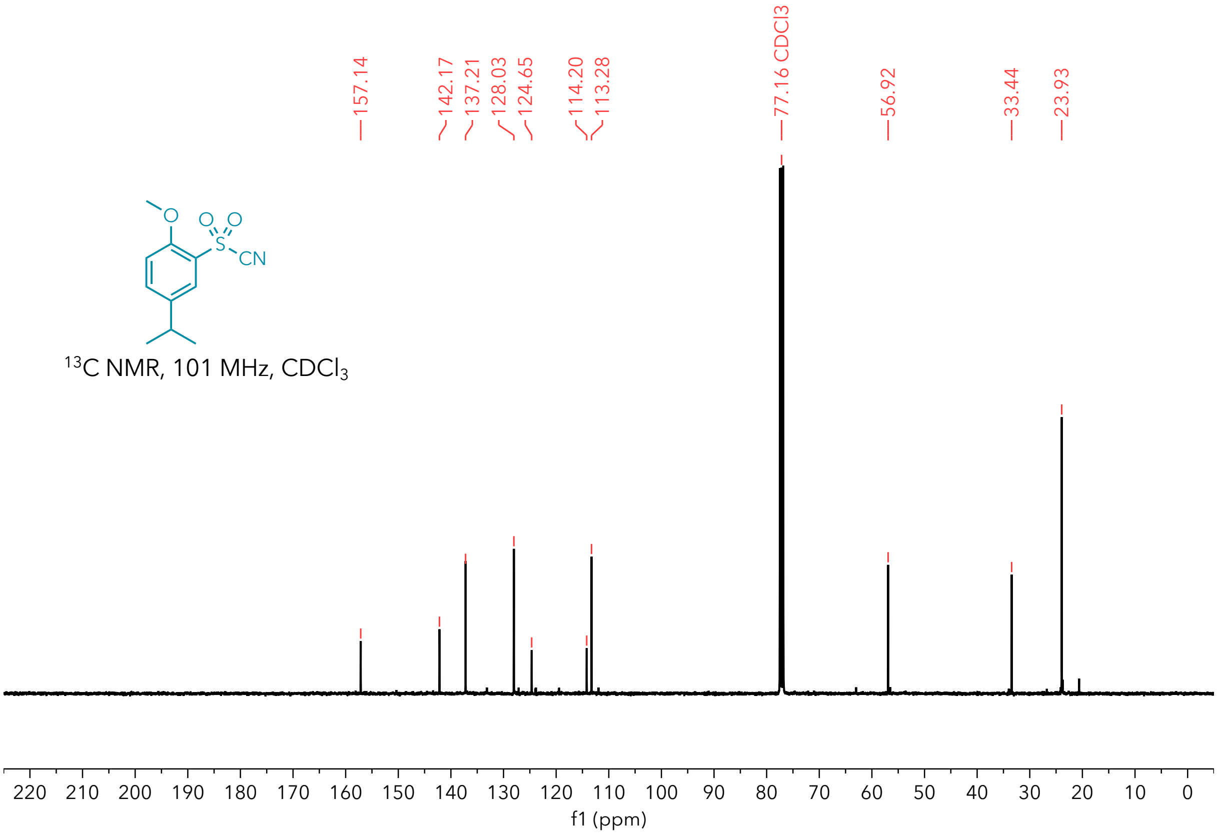
13C NMR, 101 MHz, CDCl3
157.14 142.17 137.21 128.03 124.65 114.20 113.28 77.16 CDCl3 56.92 33.44 23.93
f1 (ppm)

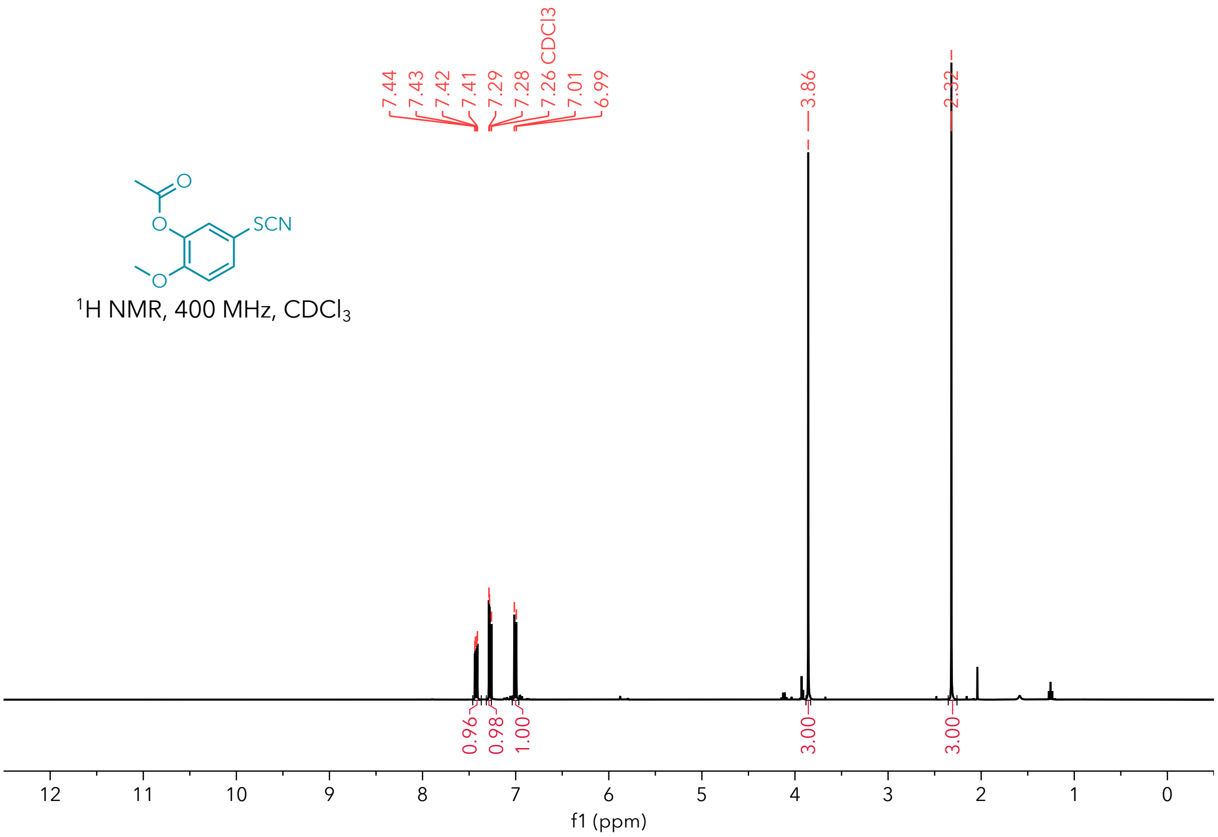
7.44
7.43
7.42
7.41
7.29
7.28
7.26 CDCl3
7.01
6.99
3.86
2.32
SCN
O
O
O
1H NMR, 400 MHz, CDCl3
0.96
0.98
1.00
3.00
3.00
12
11
10
9
8
7
6
5
4
3
2
1
0
f1 (ppm)

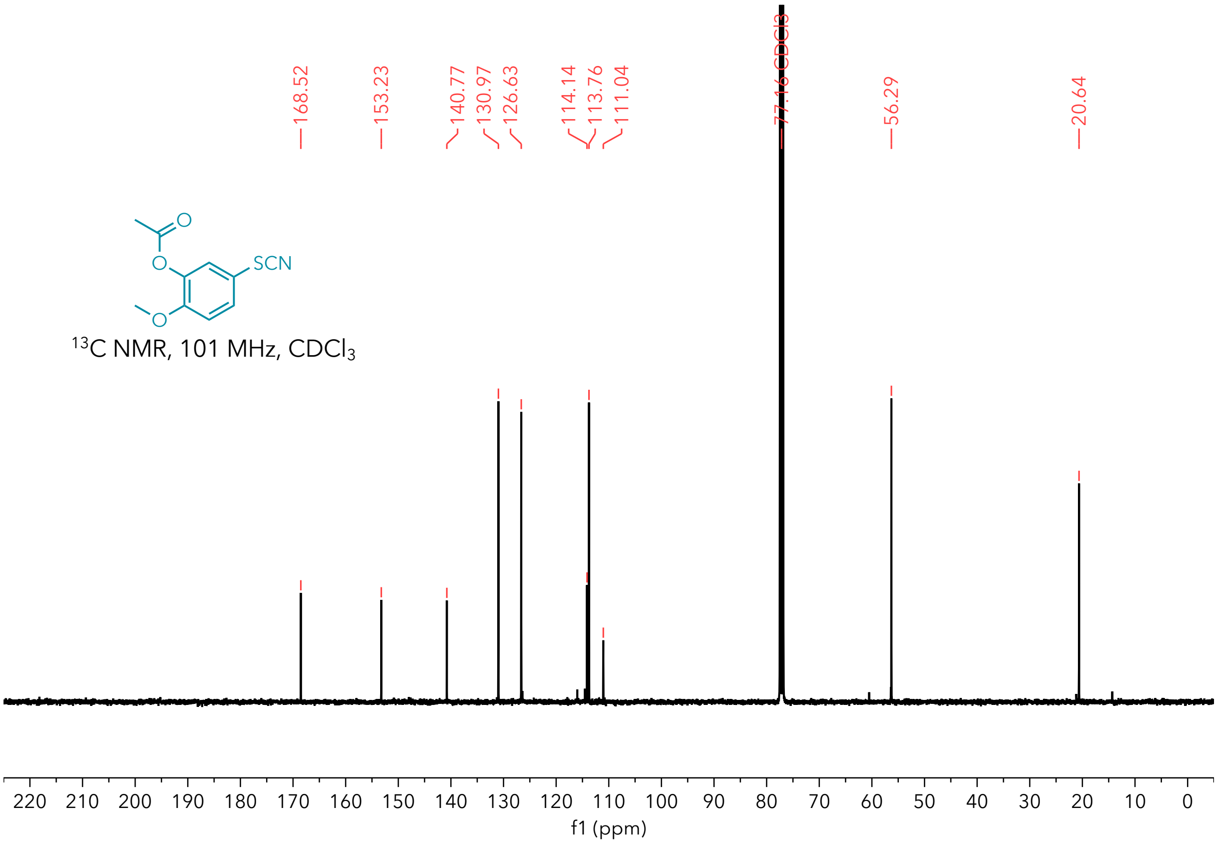
168.52
153.23
140.77
130.97
126.63
114.14
113.76
111.04
77.16 CDCl3
56.29
20.64
SCN
O
O
O
13C NMR, 101 MHz, CDCl3
220
210
200
190
180
170
160
150
140
130
120
110
100
90
80
70
60
50
40
30
20
10
0
f1 (ppm)

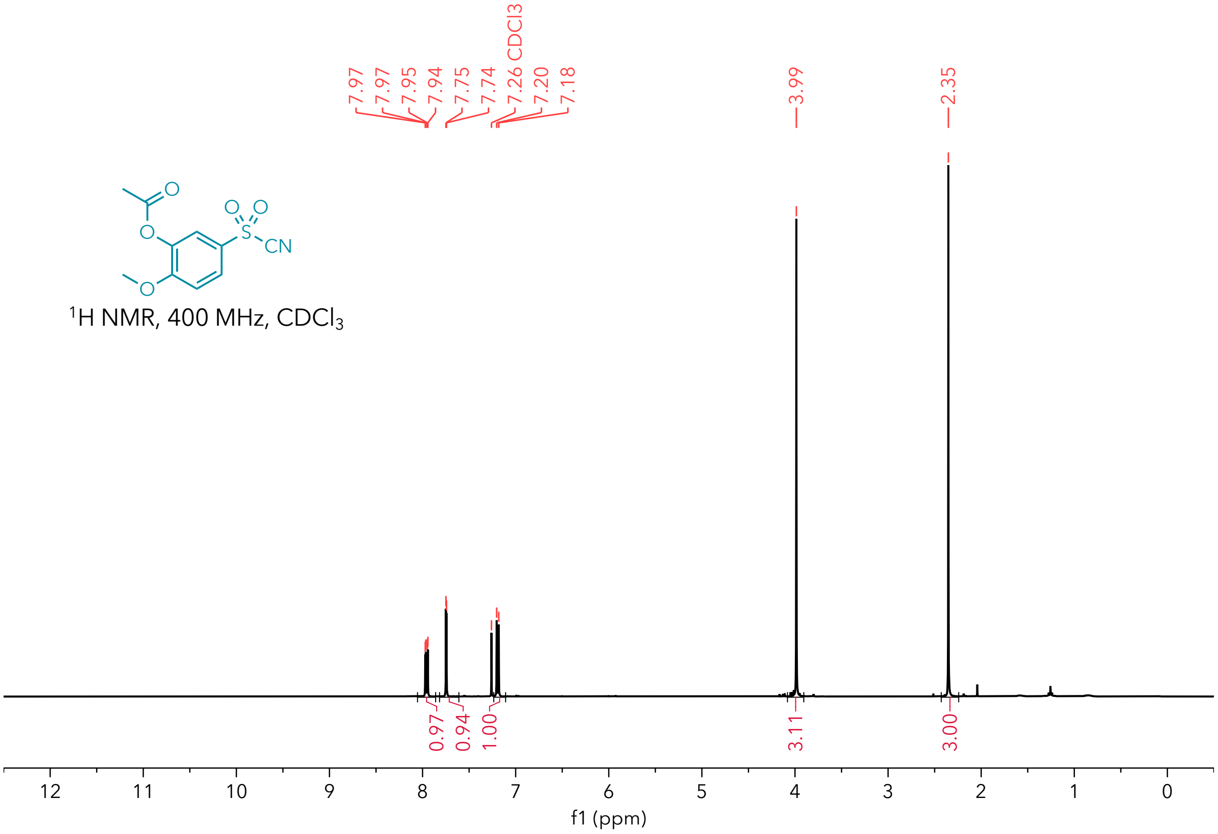
7.97
7.97
7.95
7.94
7.75
7.74
7.26 CDCl3
7.20
7.18
3.99
2.35
O
O
O
S
O
O
CN
O
^{1}H NMR, 400 MHz, CDCl$_3$
0.97
0.94
1.00
3.11
3.00
12
11
10
9
8
7
6
5
4
3
2
1
0
f1 (ppm)

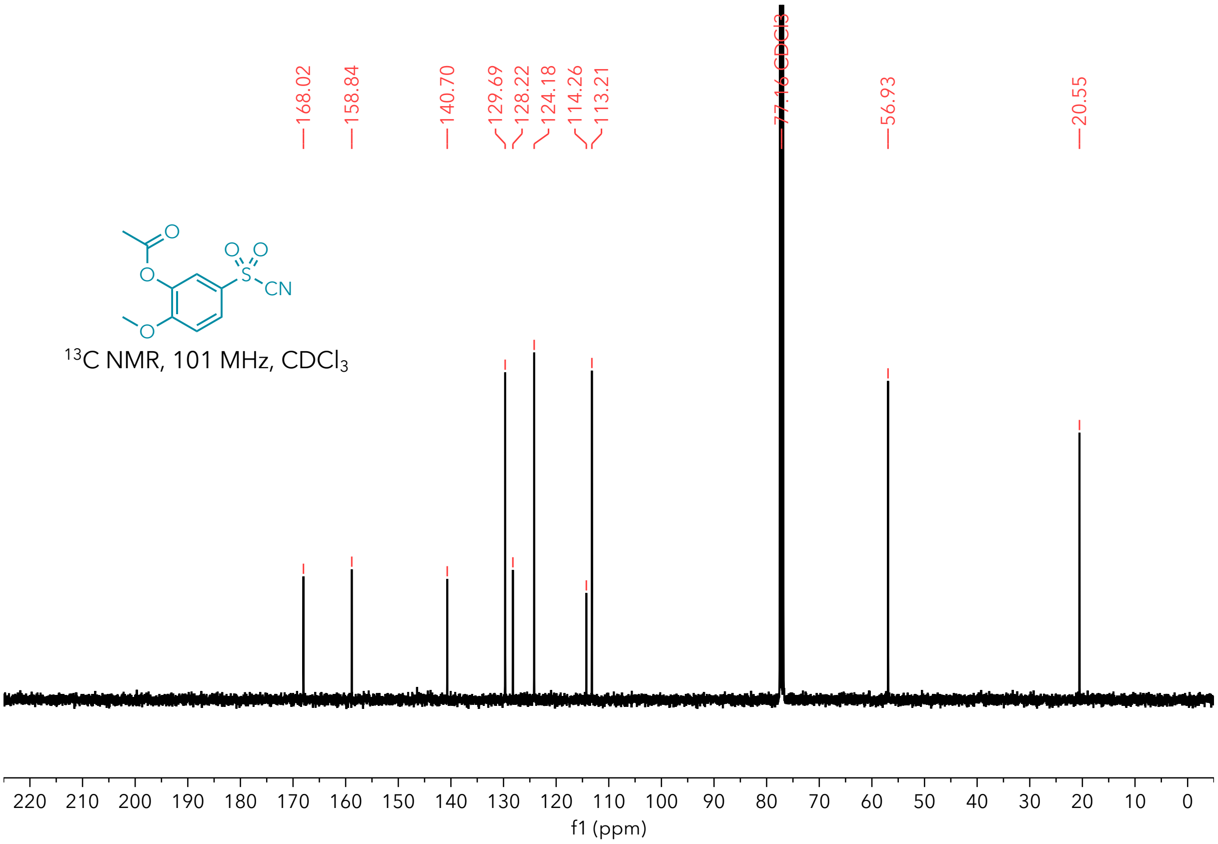
168.02
158.84
140.70
129.69
128.22
124.18
114.26
113.21
77.16 CDCl3
56.93
20.55
O
O
O
S
O
O
CN
O
^{13}C NMR, 101 MHz, CDCl$_3$
220
210
200
190
180
170
160
150
140
130
120
110
100
90
80
70
60
50
40
30
20
10
0
f1 (ppm)

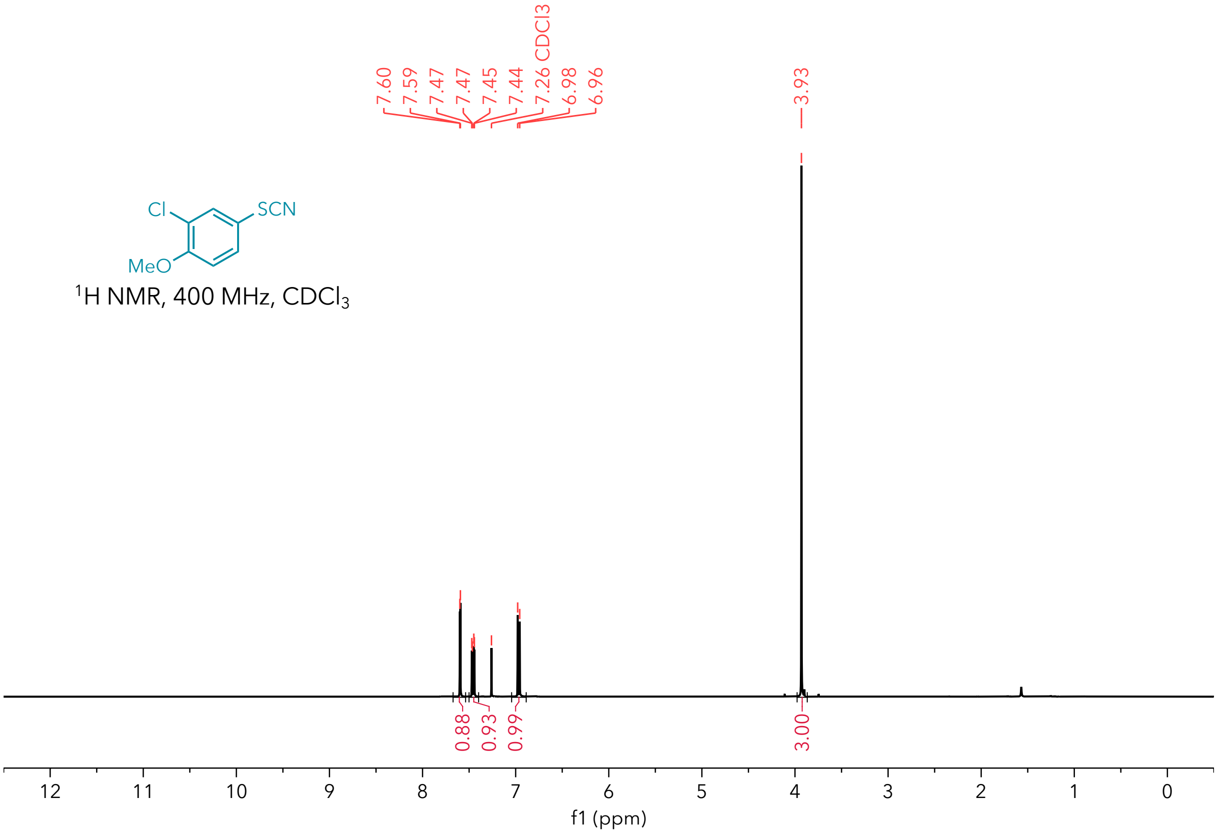

^1H NMR, 400 MHz, $CDCl_3$

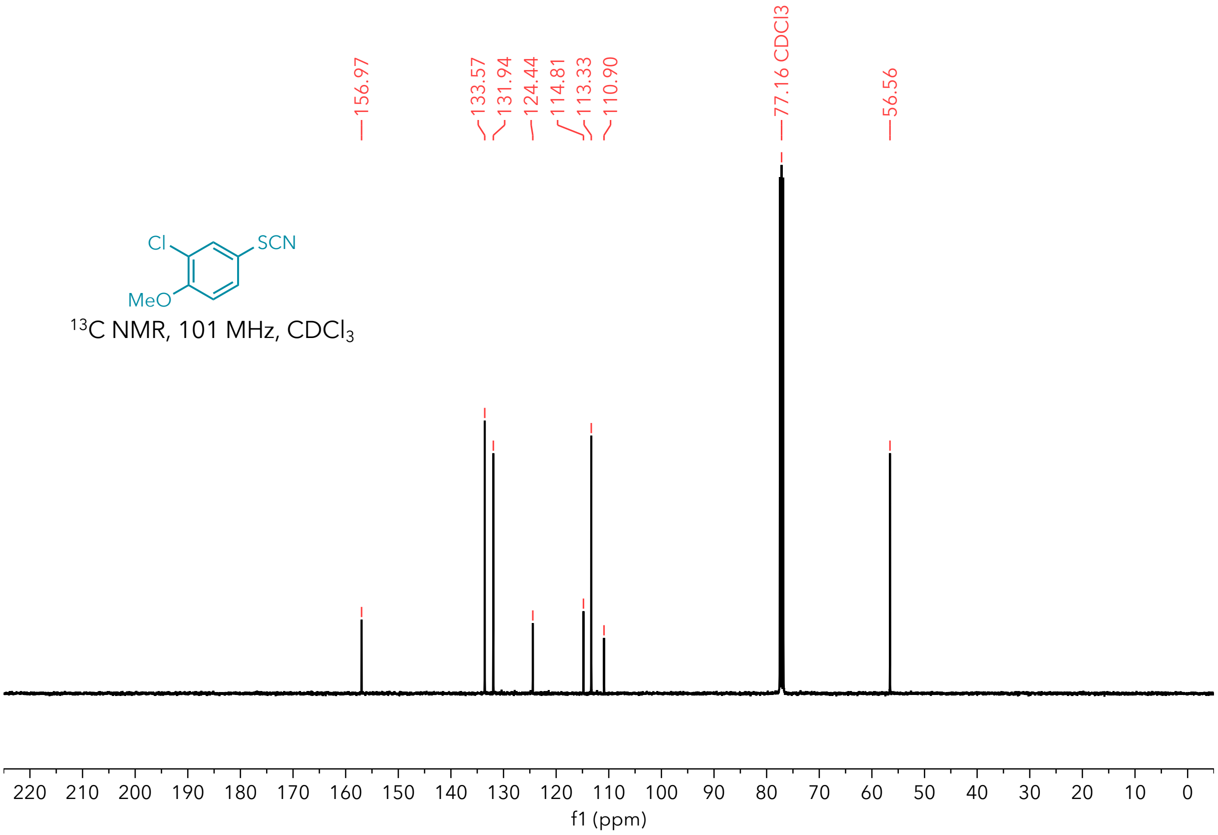

^{13}C NMR, 101 MHz, $CDCl_3$

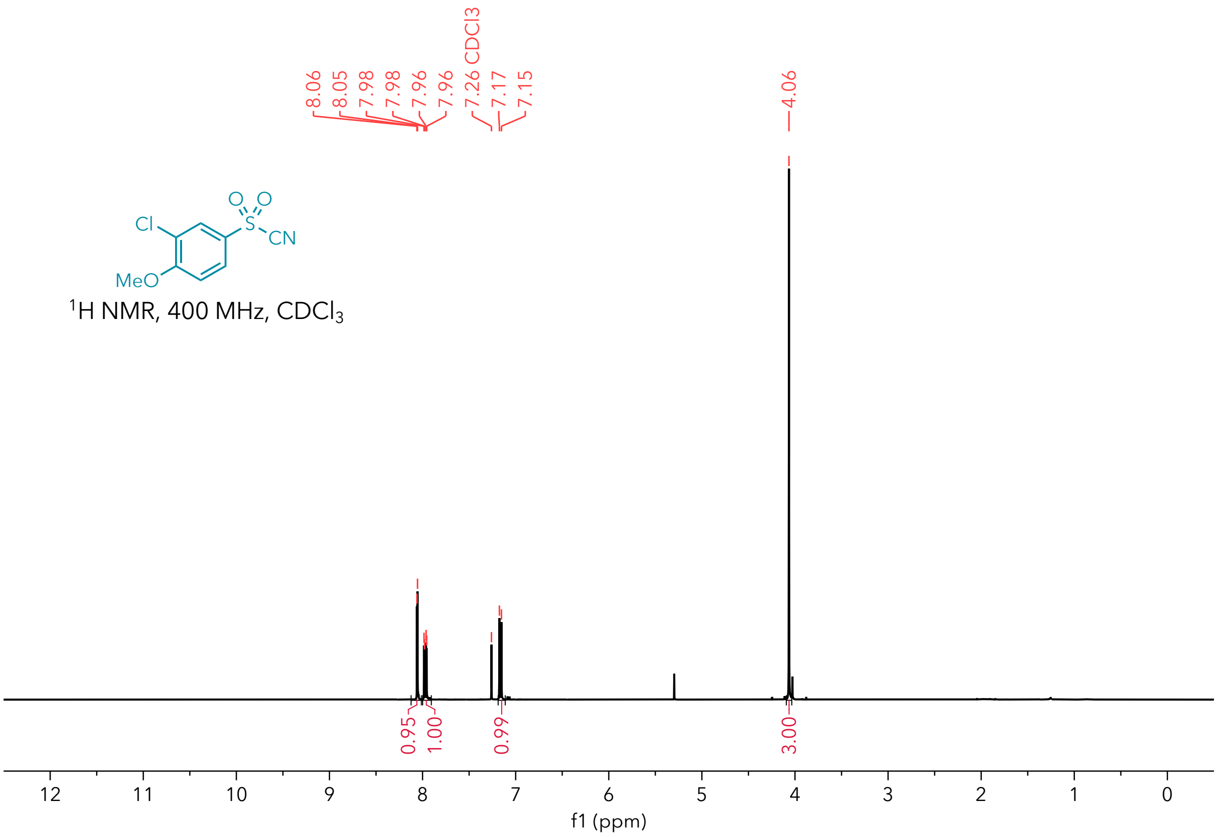
8.06
8.05
7.98
7.98
7.96
7.96
7.26 CDCl3
7.17
7.15
4.06
Cl
MeO
O
O
S
CN
1H NMR, 400 MHz, CDCl3
0.95
1.00
0.99
3.00
12
11
10
9
8
7
6
5
4
3
2
1
0
f1 (ppm)

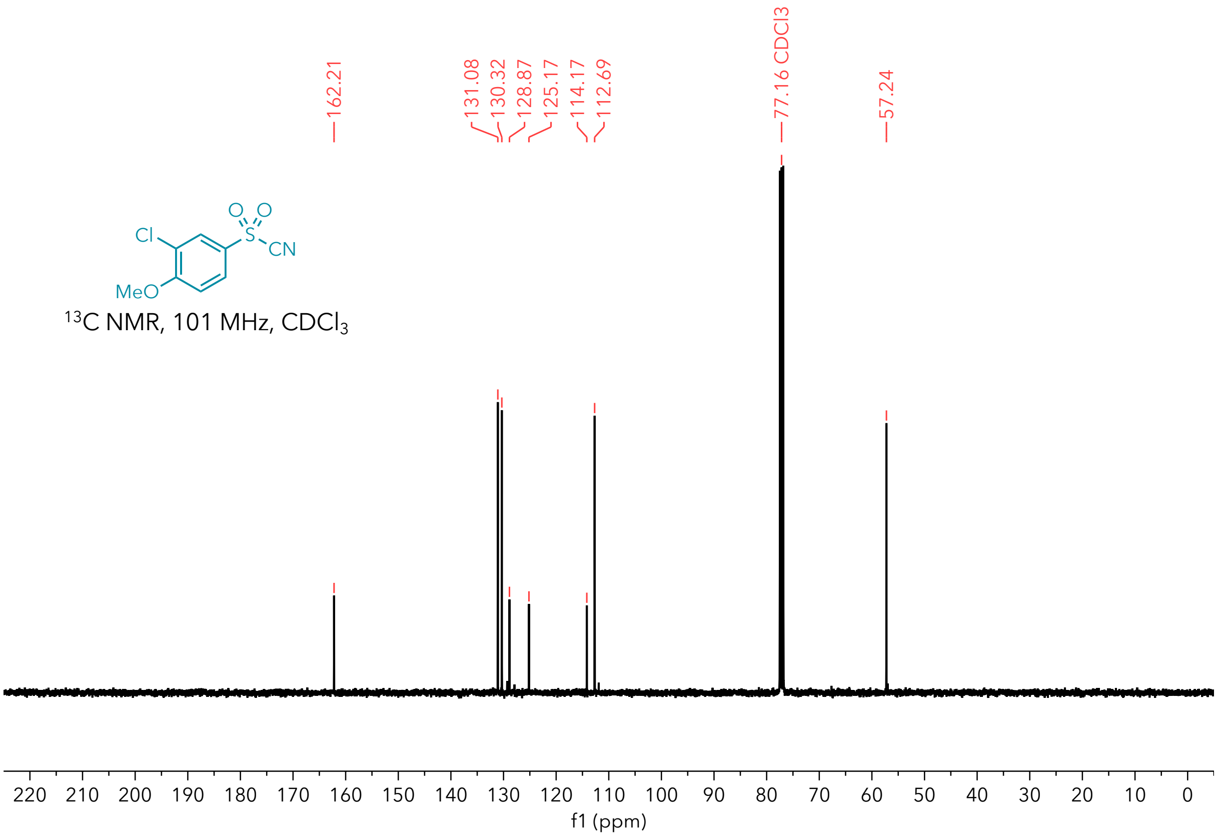
162.21
131.08
130.32
128.87
125.17
114.17
112.69
77.16 CDCl3
57.24
Cl
MeO
O
O
S
CN
13C NMR, 101 MHz, CDCl3
220
210
200
190
180
170
160
150
140
130
120
110
100
90
80
70
60
50
40
30
20
10
0
f1 (ppm)

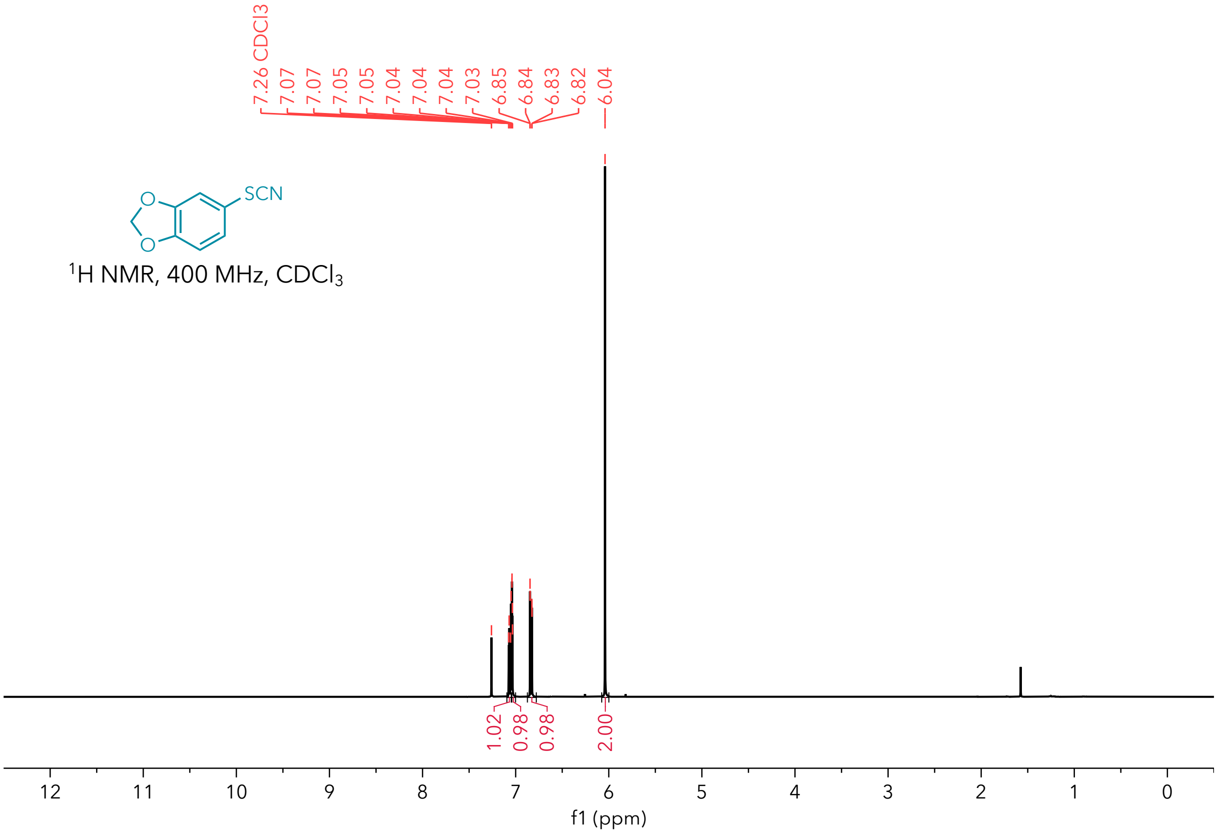
7.26 CDCl3
7.07
7.07
7.05
7.05
7.04
7.04
7.04
7.03
6.85
6.84
6.83
6.82
6.04
SCN
O
O
1H NMR, 400 MHz, CDCl3
1.02
0.98
0.98
2.00
12
11
10
9
8
7
6
5
4
3
2
1
0
f1 (ppm)

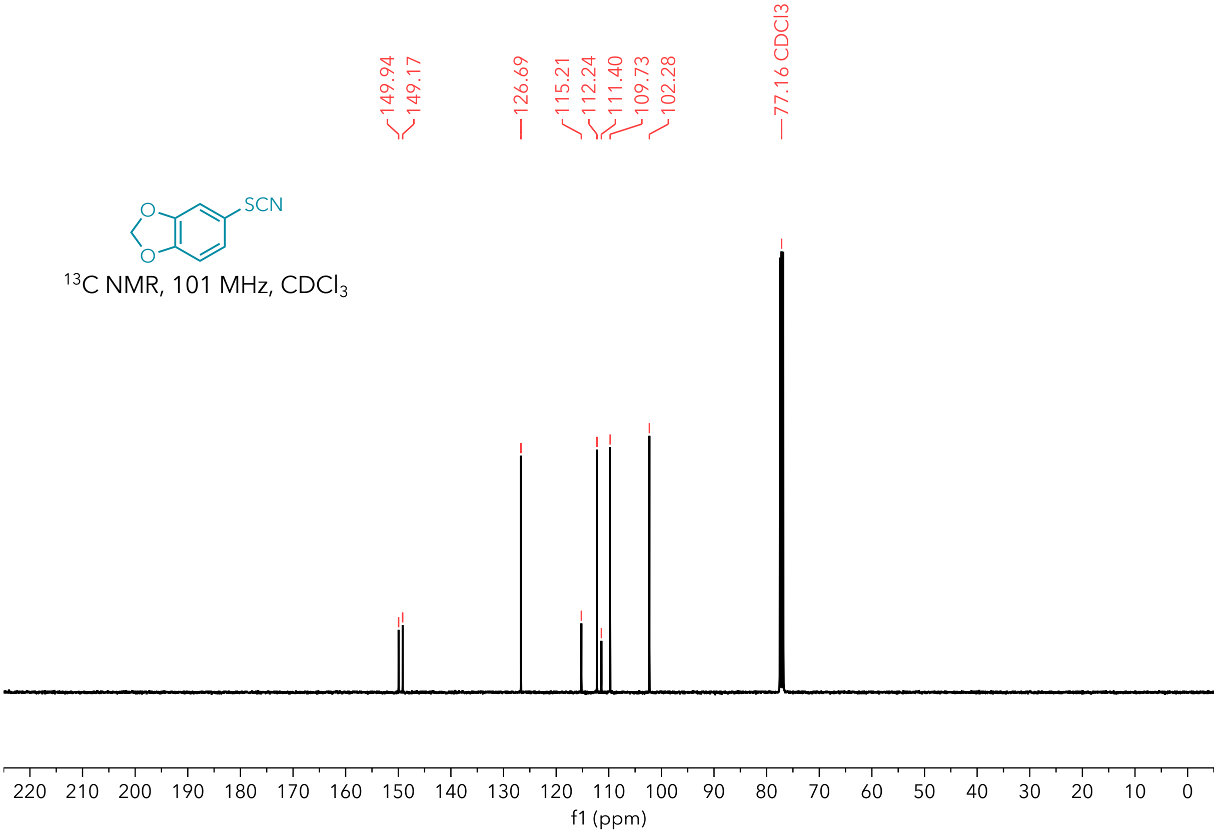
149.94
149.17
126.69
115.21
112.24
111.40
109.73
102.28
77.16 CDCl3
SCN
O
O
13C NMR, 101 MHz, CDCl3
220
210
200
190
180
170
160
150
140
130
120
110
100
90
80
70
60
50
40
30
20
10
0
f1 (ppm)

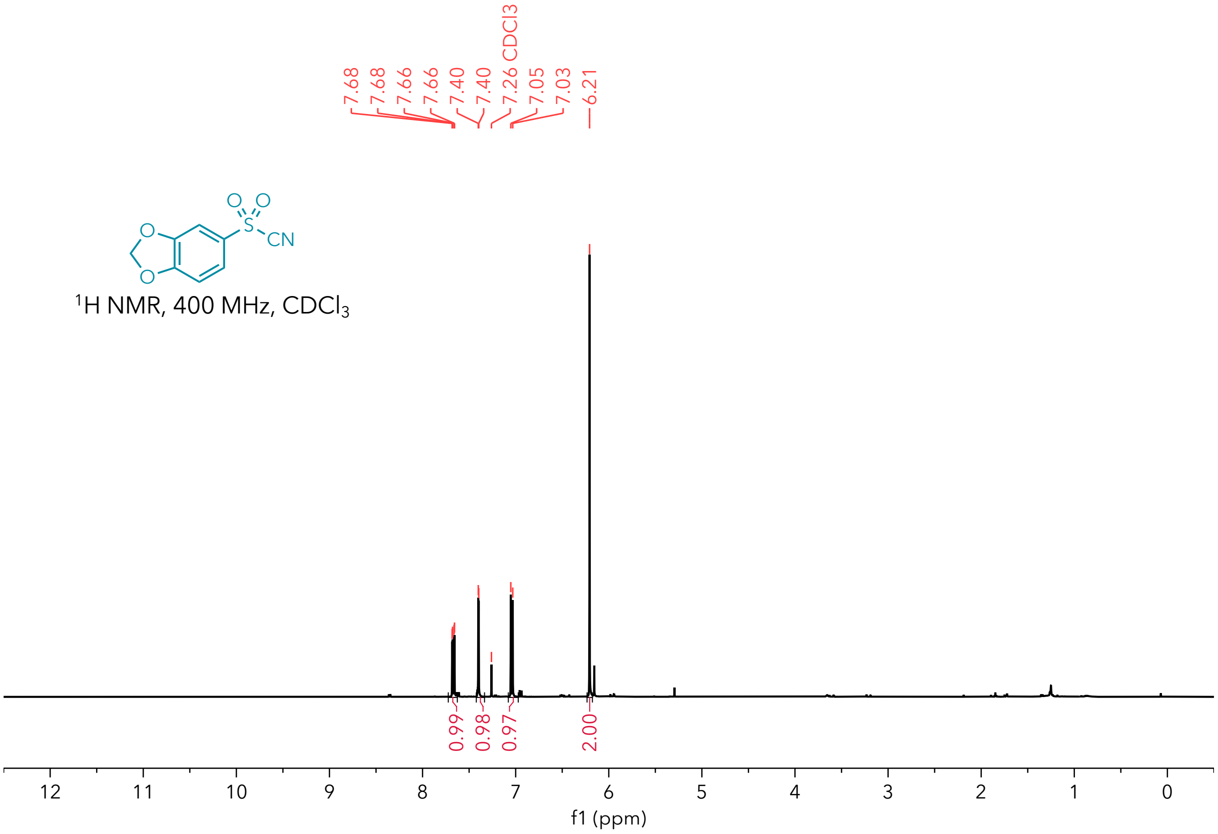

^{1}H NMR, 400 MHz, $CDCl_3$

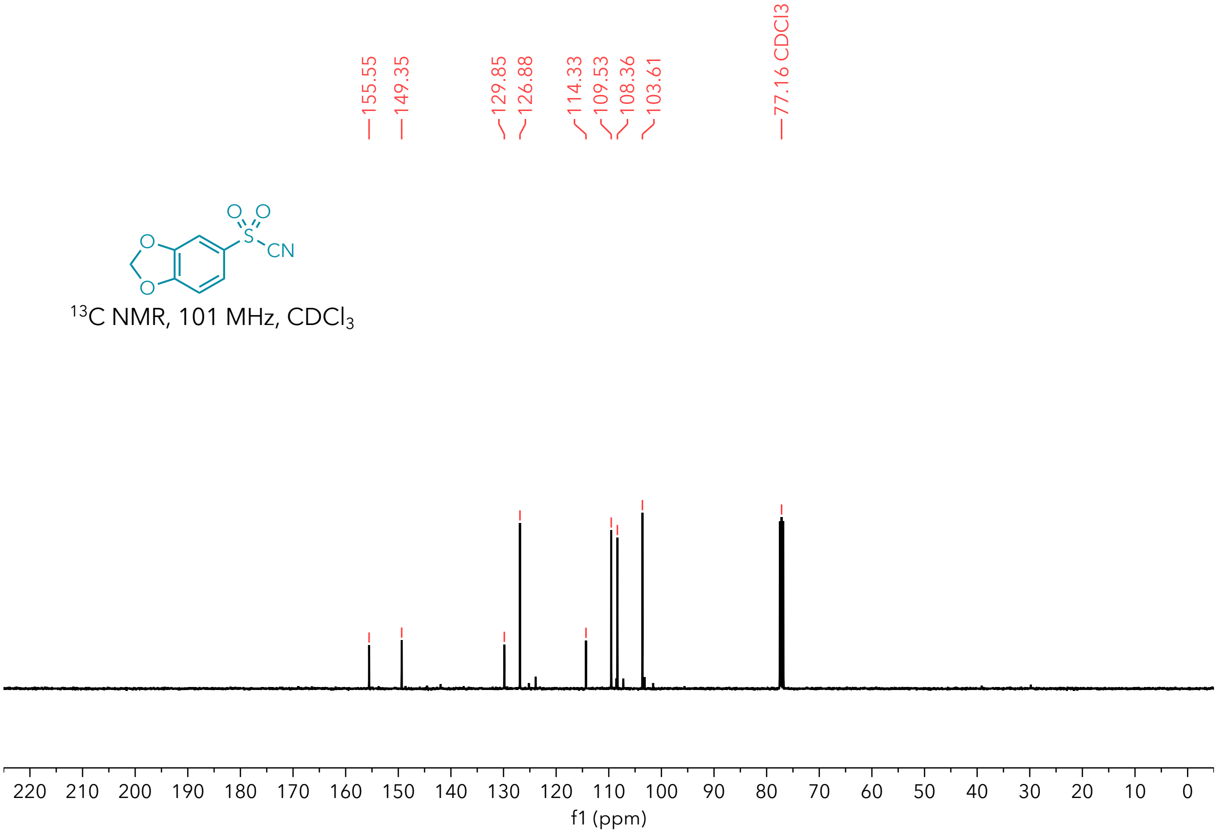

^{13}C NMR, 101 MHz, $CDCl_3$

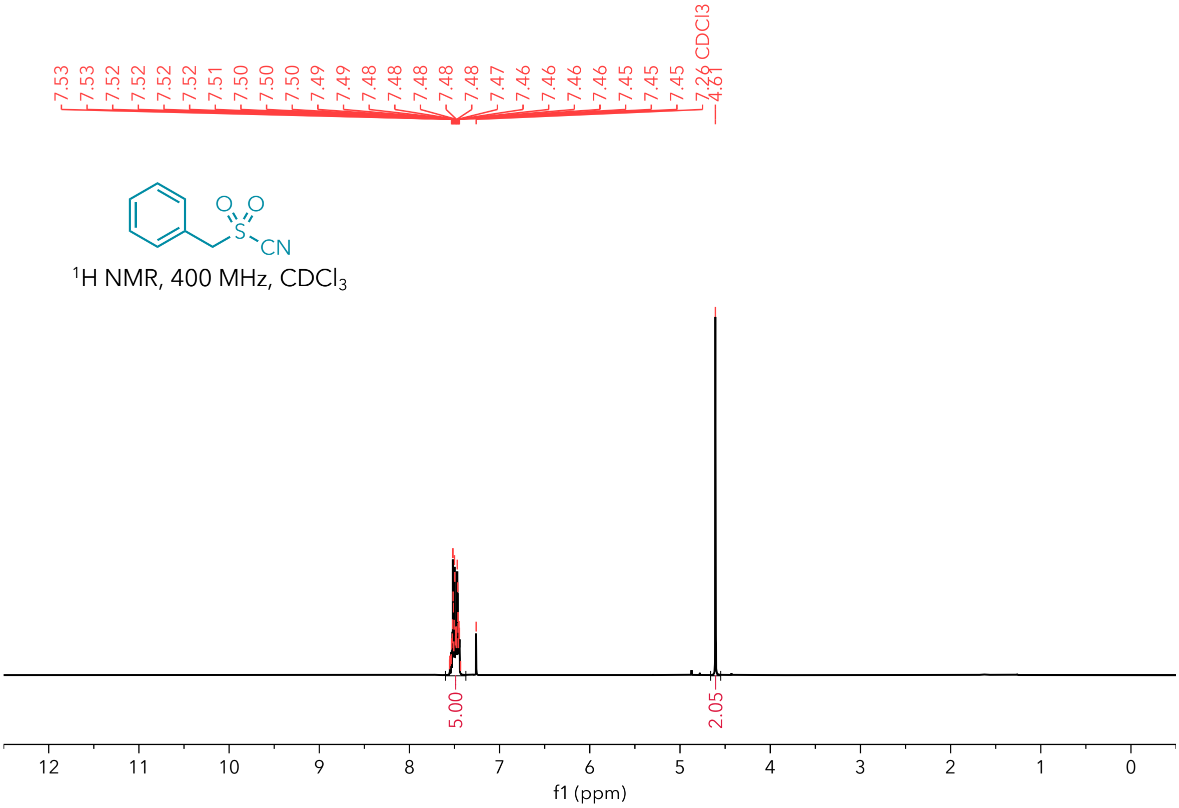
7.53
7.53
7.52
7.52
7.52
7.52
7.51
7.50
7.50
7.50
7.49
7.49
7.48
7.48
7.48
7.48
7.48
7.47
7.46
7.46
7.46
7.46
7.45
7.45
7.45
7.26 CDCl3
4.61
O O
S
CN
1H NMR, 400 MHz, CDCl3
5.00
2.05
12 11 10 9 8 7 6 5 4 3 2 1 0
f1 (ppm)

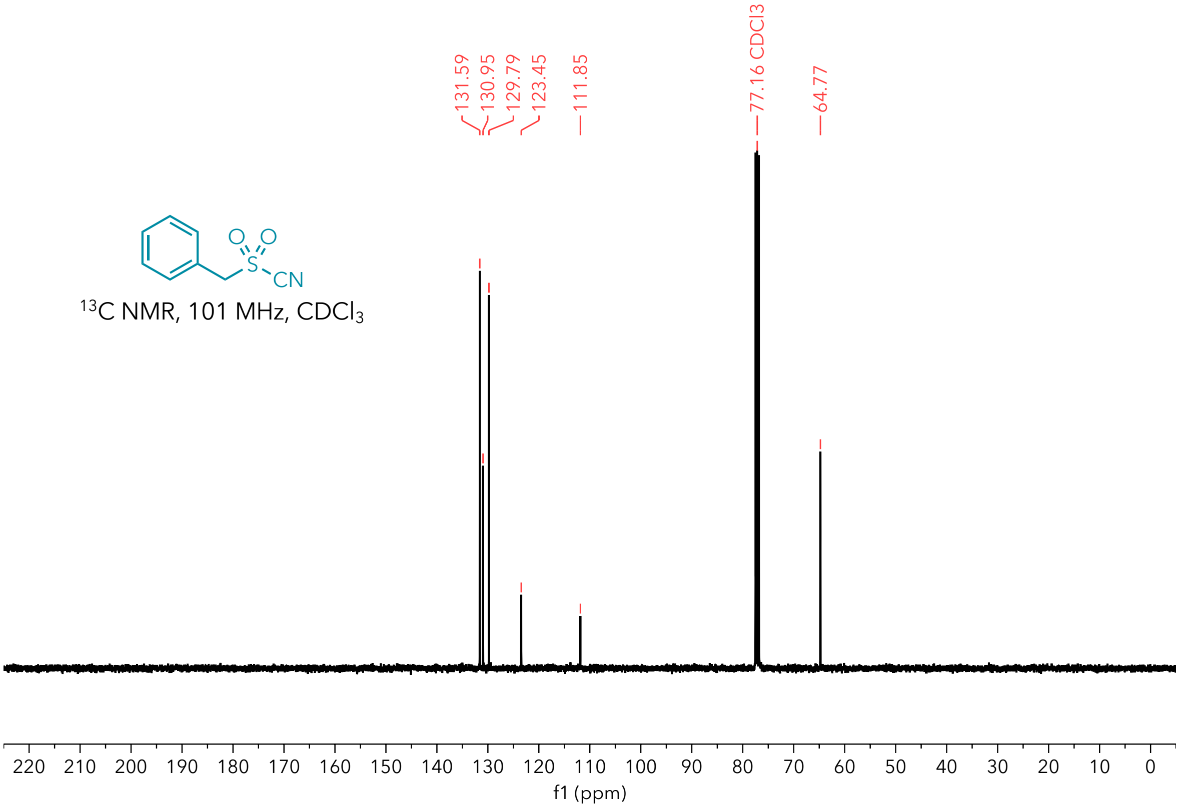
131.59
130.95
129.79
123.45
111.85
77.16 CDCl3
64.77
O O
S
CN
13C NMR, 101 MHz, CDCl3
220 210 200 190 180 170 160 150 140 130 120 110 100 90 80 70 60 50 40 30 20 10 0
f1 (ppm)

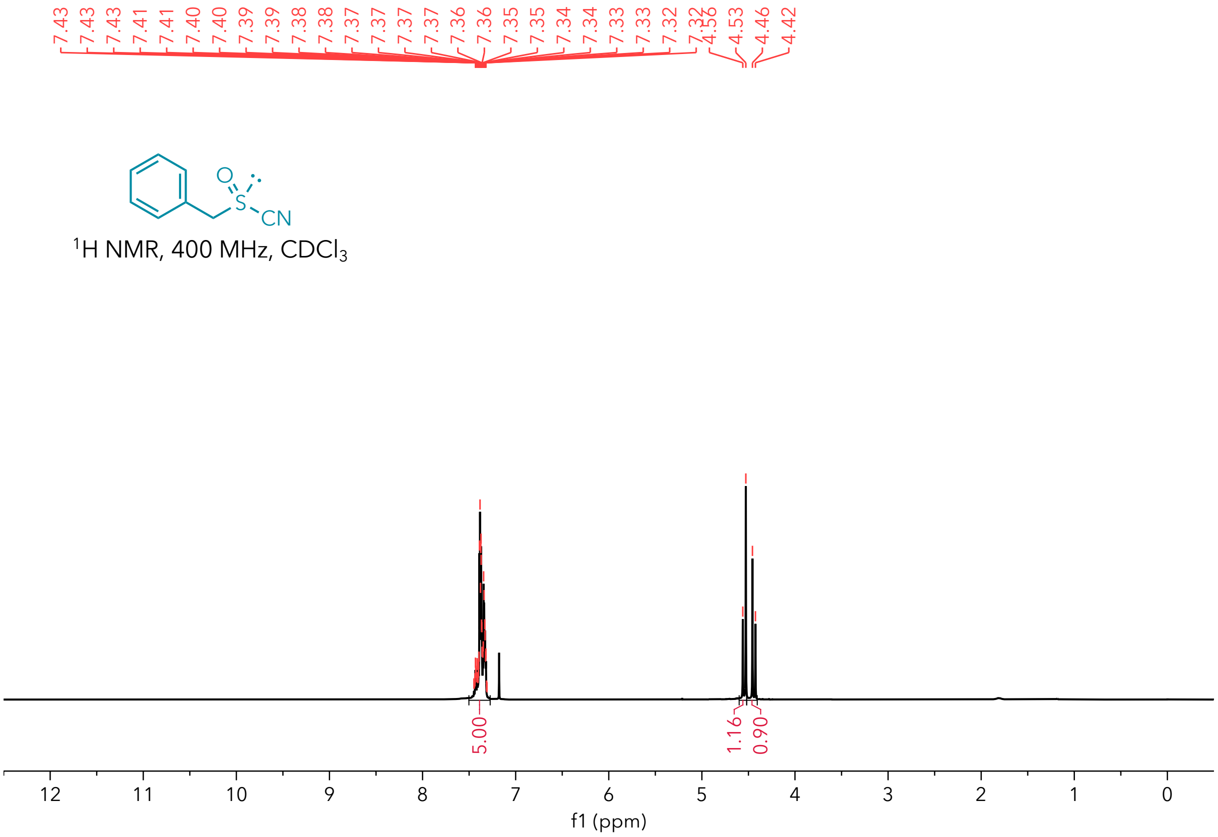
7.43 7.43 7.43 7.41 7.41 7.40 7.40 7.39 7.39 7.38 7.38 7.37 7.37 7.37 7.37 7.36 7.36 7.35 7.35 7.34 7.34 7.33 7.33 7.32 7.32 4.56 4.53 4.46 4.42
O
S
CN
1H NMR, 400 MHz, CDCl3
5.00
1.16
0.90
12 11 10 9 8 7 6 5 4 3 2 1 0
f1 (ppm)

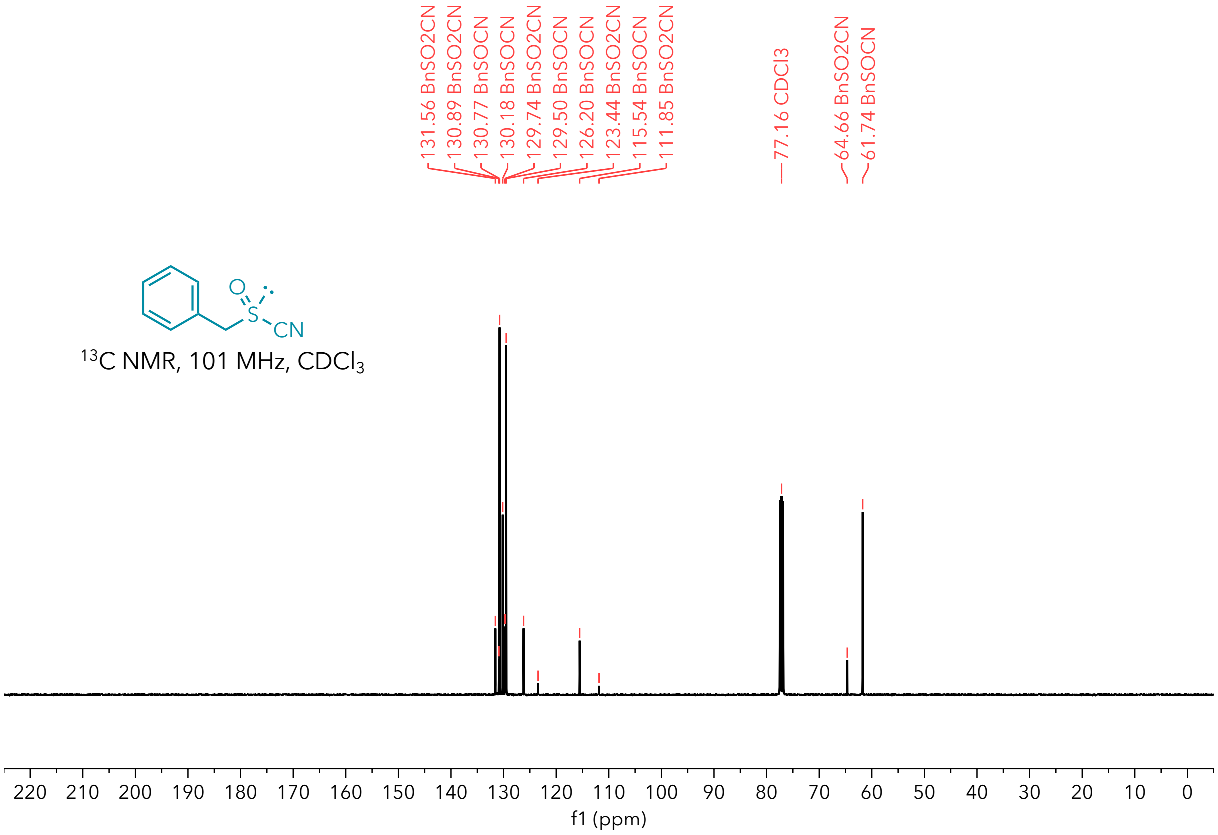
131.56 BnSO2CN
130.89 BnSO2CN
130.77 BnSOCN
130.18 BnSOCN
129.74 BnSO2CN
129.50 BnSOCN
126.20 BnSOCN
123.44 BnSO2CN
115.54 BnSOCN
111.85 BnSO2CN
77.16 CDCl3
64.66 BnSO2CN
61.74 BnSOCN
O
S
CN
13C NMR, 101 MHz, CDCl3
220 210 200 190 180 170 160 150 140 130 120 110 100 90 80 70 60 50 40 30 20 10 0
f1 (ppm)

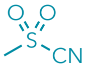

^{1}H NMR, 400 MHz, $CDCl_3$

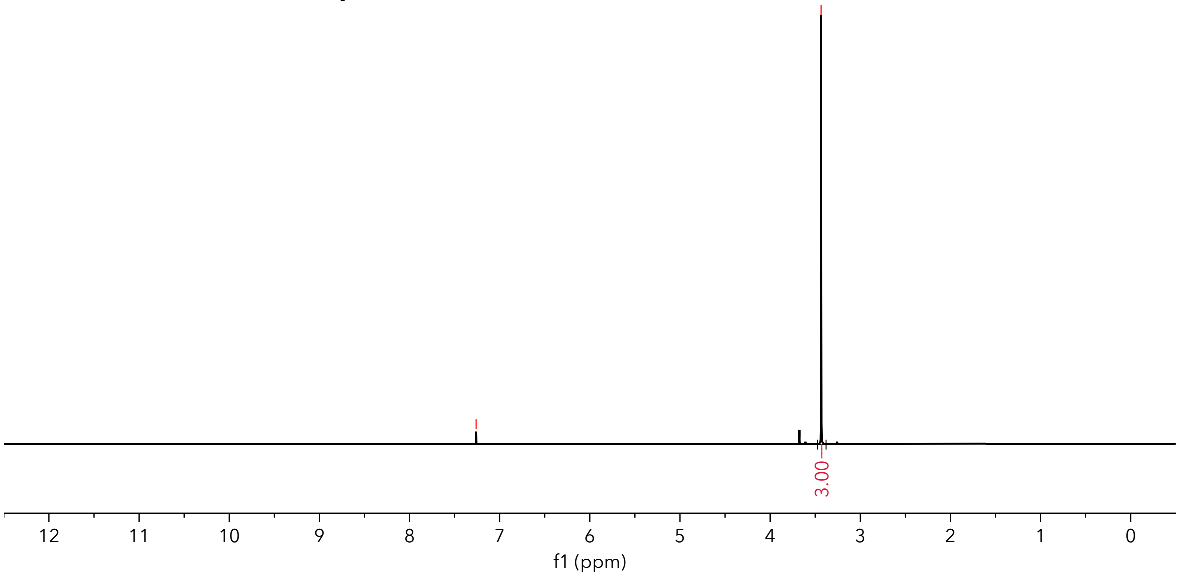

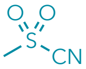

^{13}C NMR, 101 MHz, $CDCl_3$

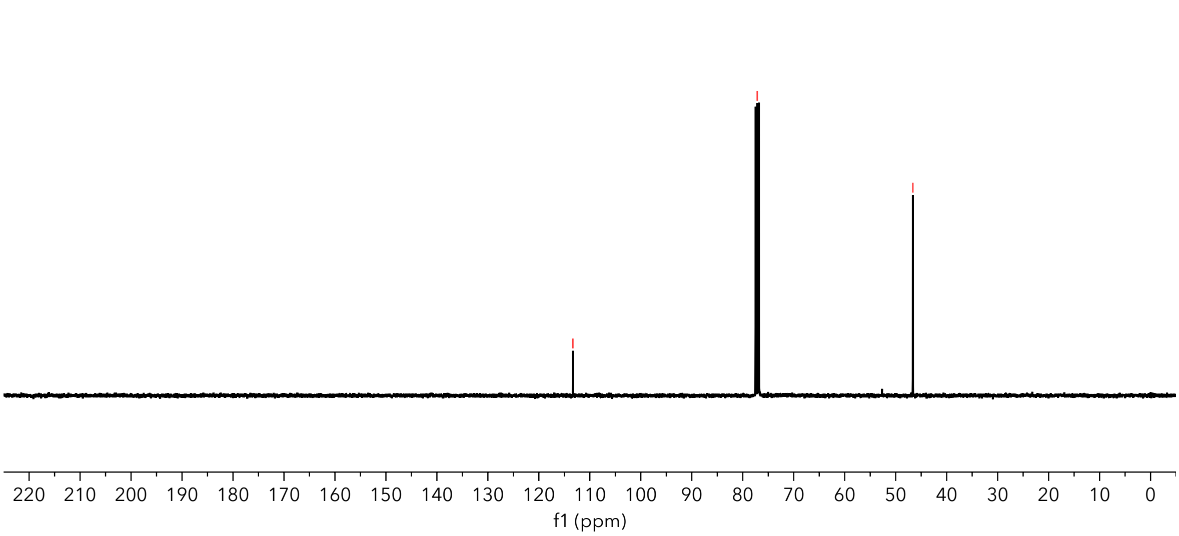

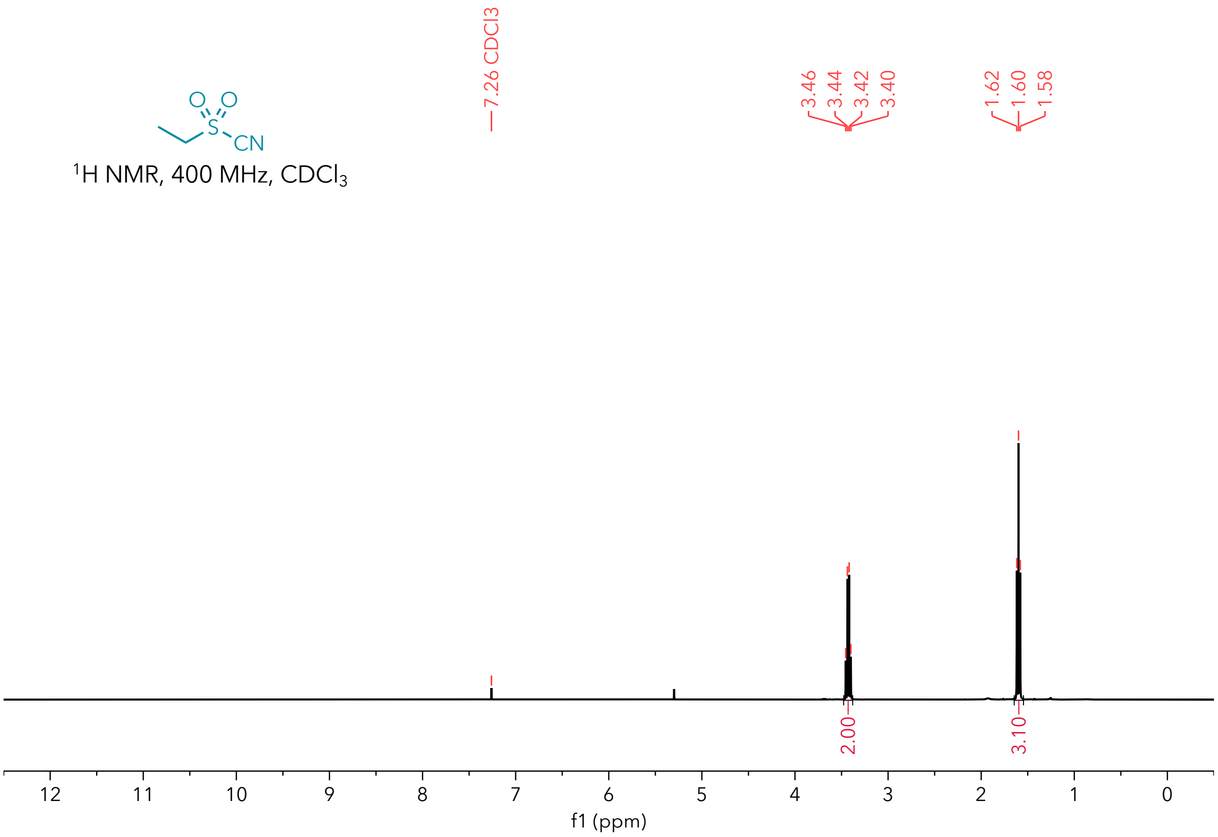
O O
S
CN
1H NMR, 400 MHz, CDCl3
7.26 CDCl3
3.46
3.44
3.42
3.40
1.62
1.60
1.58
2.00
3.10
12 11 10 9 8 7 6 5 4 3 2 1 0
f1 (ppm)

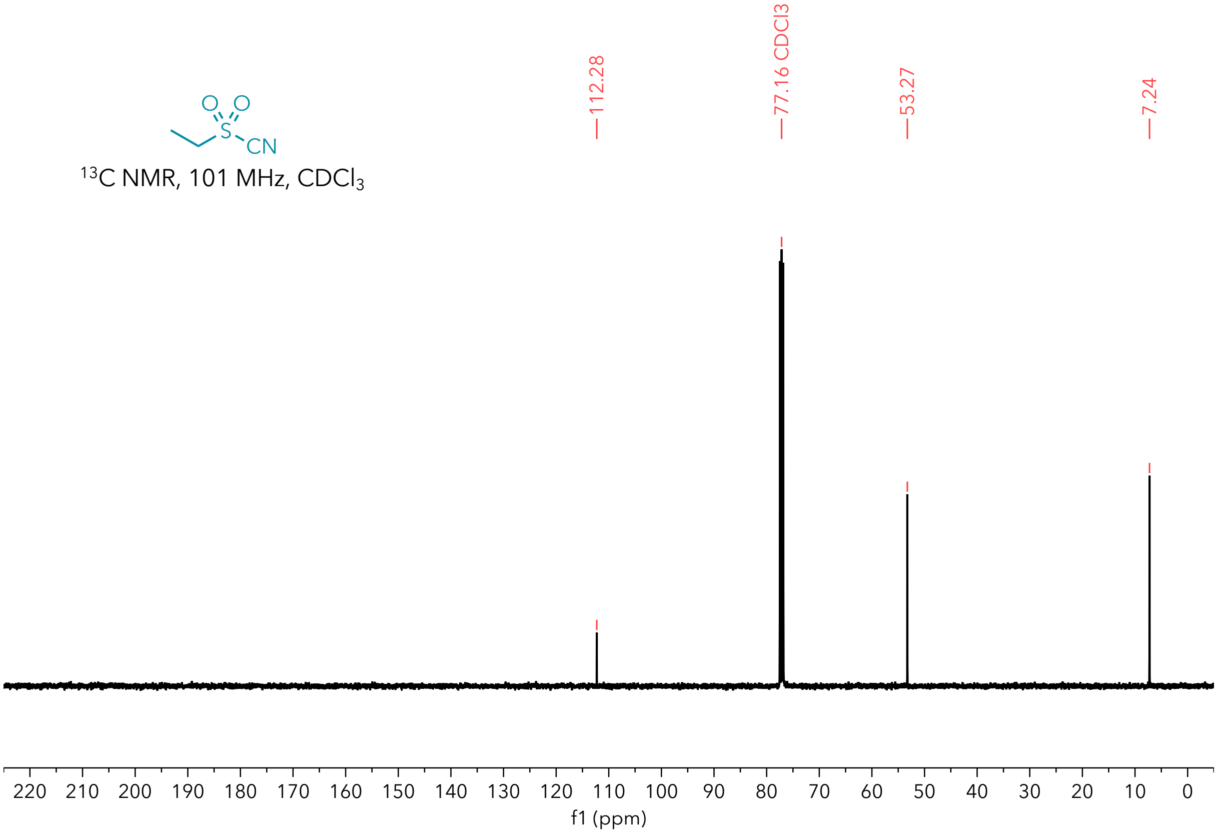
O O
S
CN
13C NMR, 101 MHz, CDCl3
112.28
77.16 CDCl3
53.27
7.24
220 210 200 190 180 170 160 150 140 130 120 110 100 90 80 70 60 50 40 30 20 10 0
f1 (ppm)

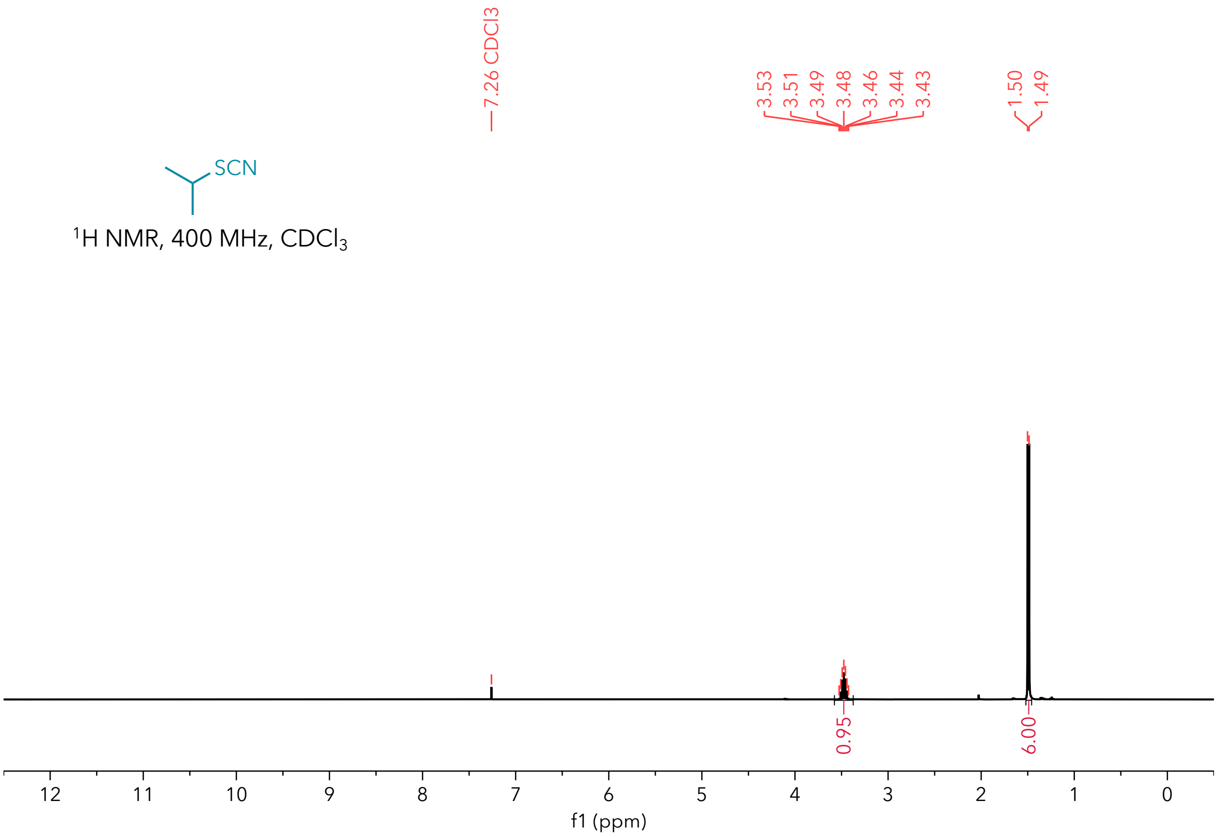
7.26 CDCl3
3.53
3.51
3.49
3.48
3.46
3.44
3.43
1.50
1.49
SCN
^{1}H NMR, 400 MHz, $CDCl_3$
0.95
6.00
12 11 10 9 8 7 6 5 4 3 2 1 0
f1 (ppm)

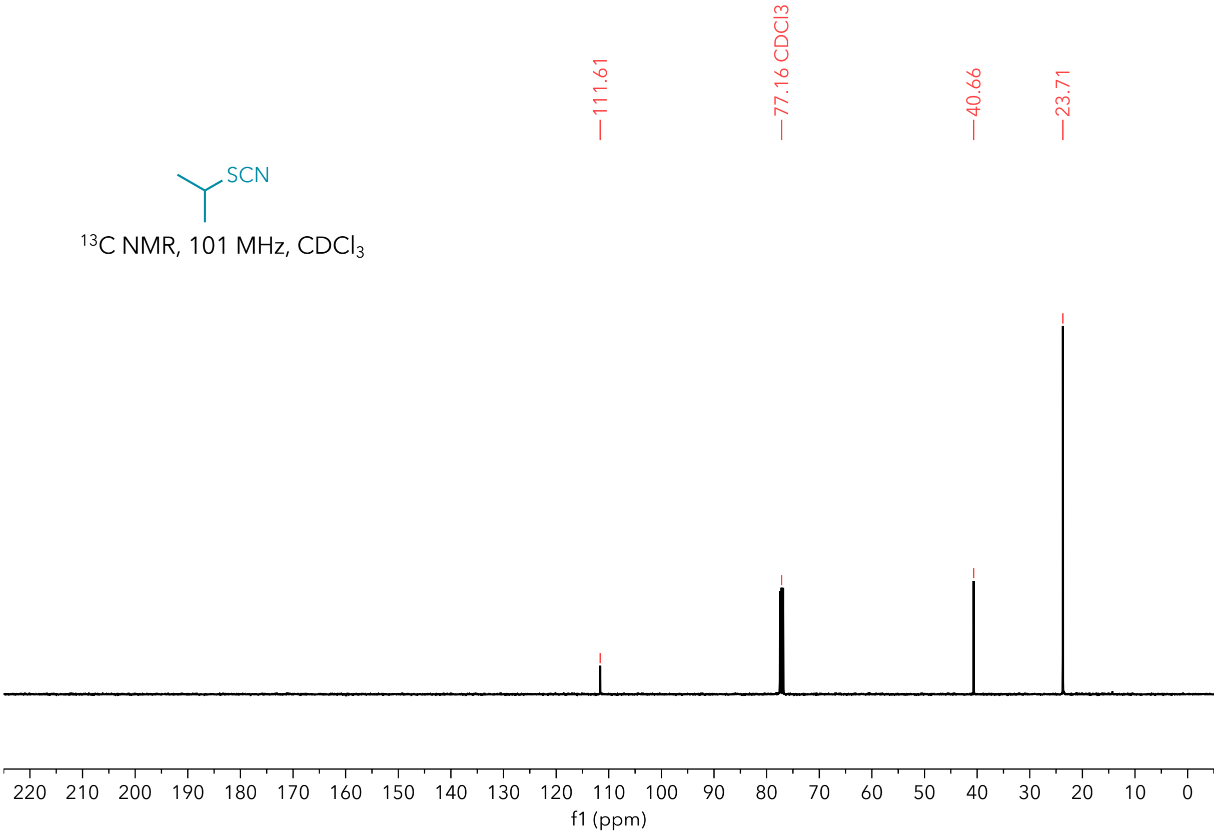
111.61
77.16 CDCl3
40.66
23.71
SCN
^{13}C NMR, 101 MHz, $CDCl_3$
220 210 200 190 180 170 160 150 140 130 120 110 100 90 80 70 60 50 40 30 20 10 0
f1 (ppm)

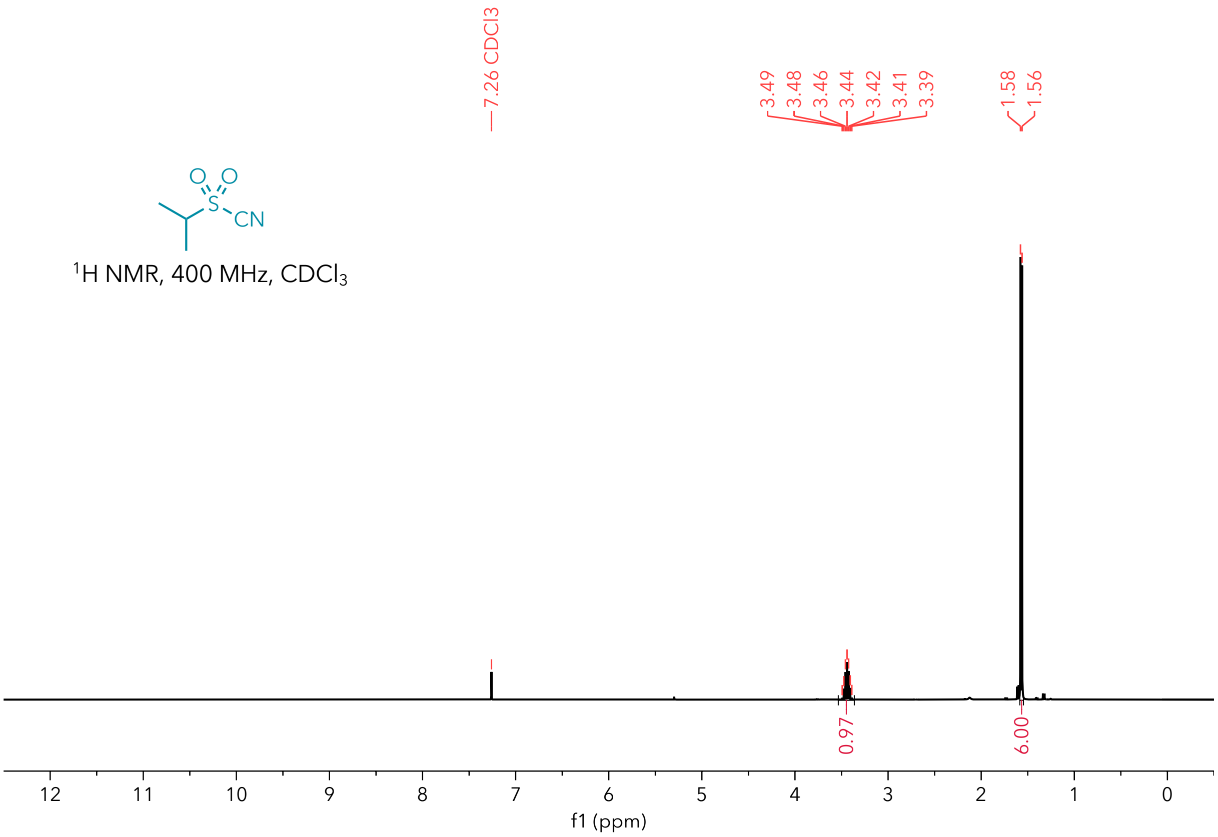

O O
S
CN
^{1}H NMR, 400 MHz, $CDCl_3$
7.26 CDCl3
3.49
3.48
3.46
3.44
3.42
3.41
3.39
1.58
1.56
0.97
6.00
12 11 10 9 8 7 6 5 4 3 2 1 0
f1 (ppm)

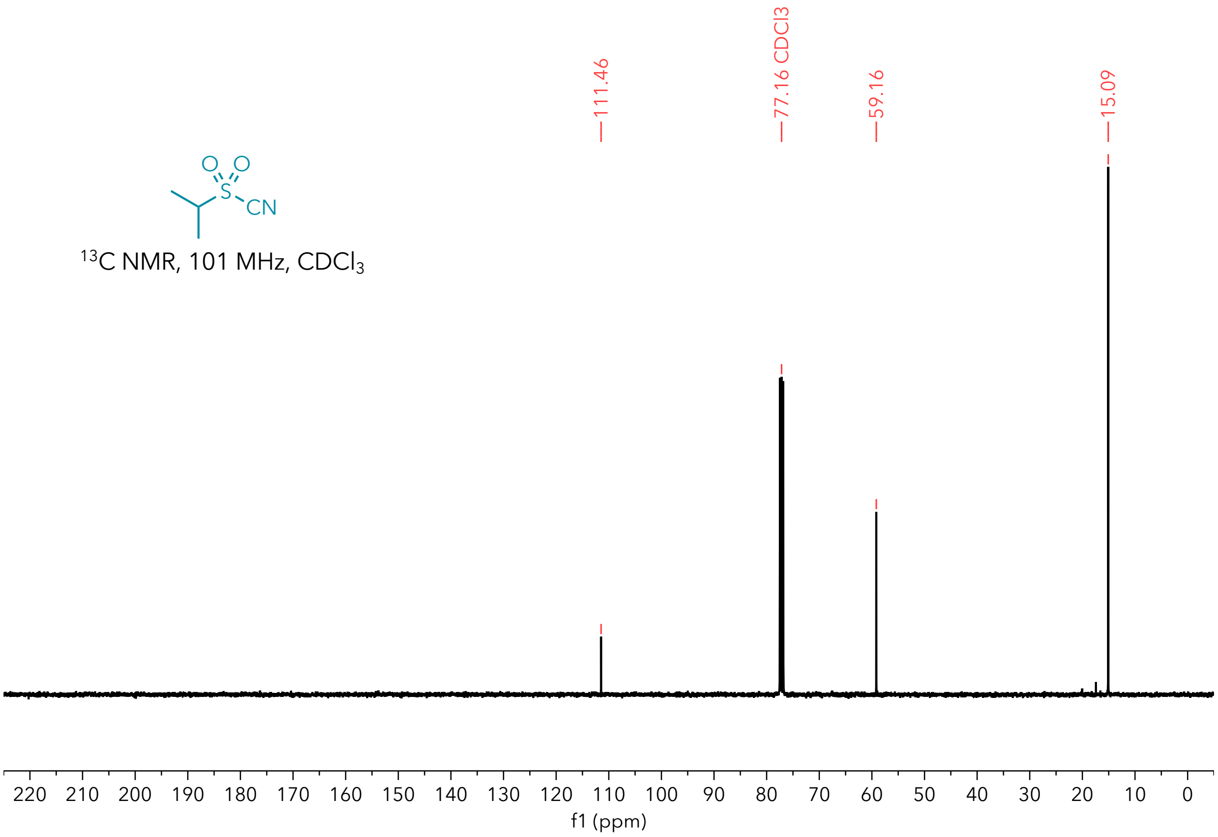

O O
S
CN
^{13}C NMR, 101 MHz, $CDCl_3$
111.46
77.16 CDCl3
59.16
15.09
220 210 200 190 180 170 160 150 140 130 120 110 100 90 80 70 60 50 40 30 20 10 0
f1 (ppm)

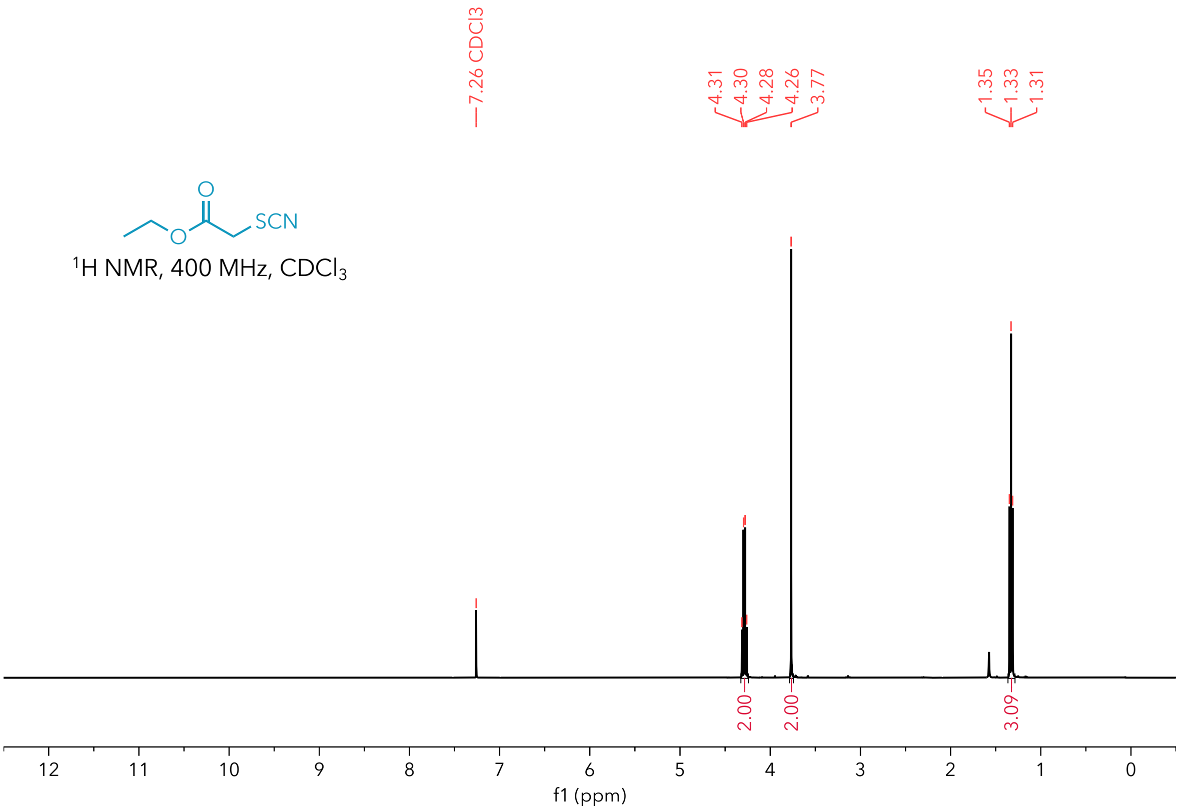

7.26 CDCl3
4.31
4.30
4.28
4.26
3.77
1.35
1.33
1.31
SCN
O
O
¹H NMR, 400 MHz, CDCl₃
2.00
2.00
3.09
f1 (ppm)

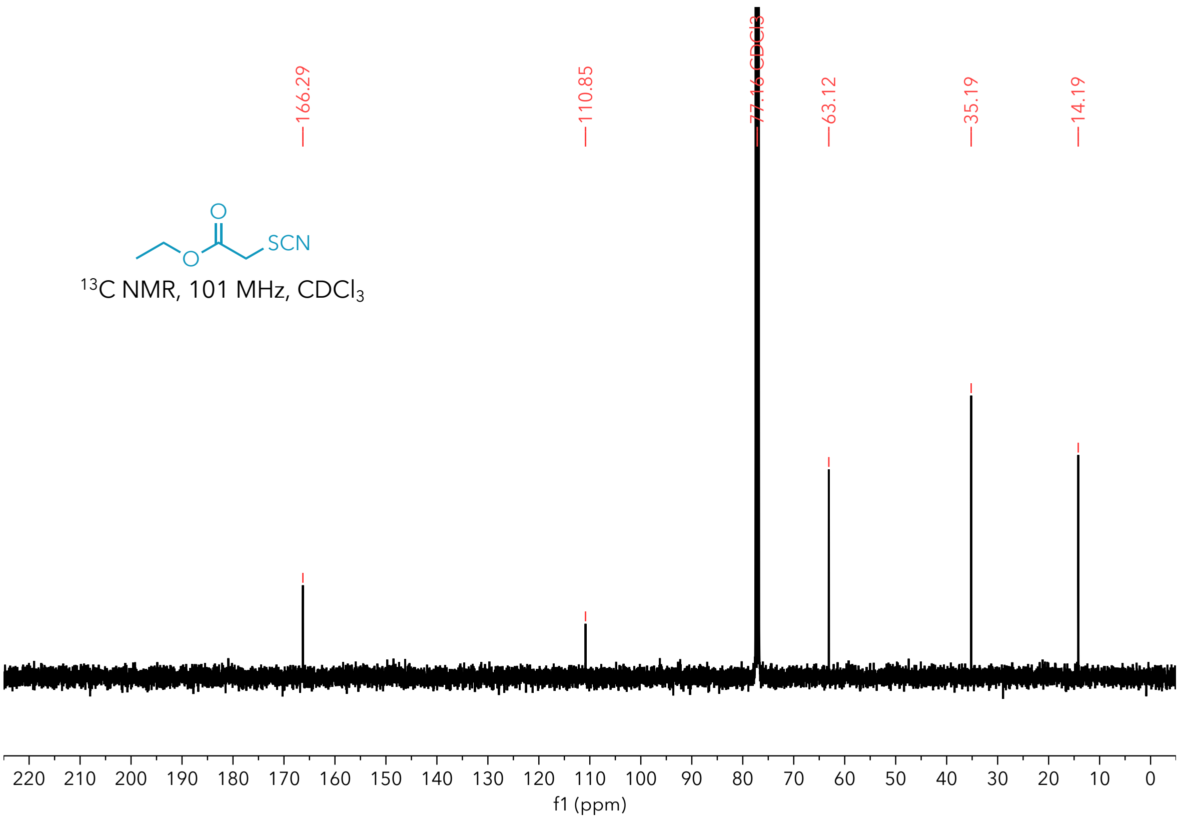

166.29
110.85
77.16 CDCl3
63.12
35.19
14.19
SCN
O
O
¹³C NMR, 101 MHz, CDCl₃
f1 (ppm)

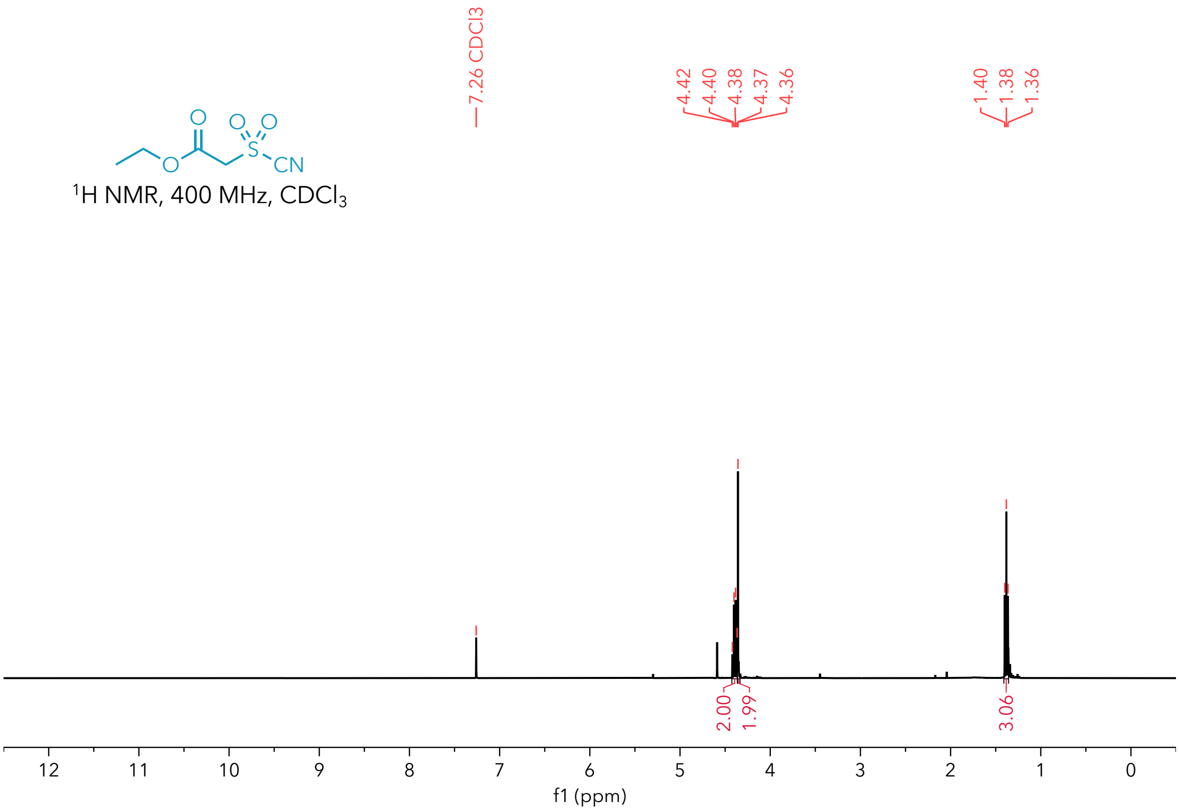
7.26 CDCl3
4.42
4.40
4.38
4.37
4.36
1.40
1.38
1.36
CN
1H NMR, 400 MHz, CDCl3
2.00
1.99
3.06
12 11 10 9 8 7 6 5 4 3 2 1 0
f1 (ppm)

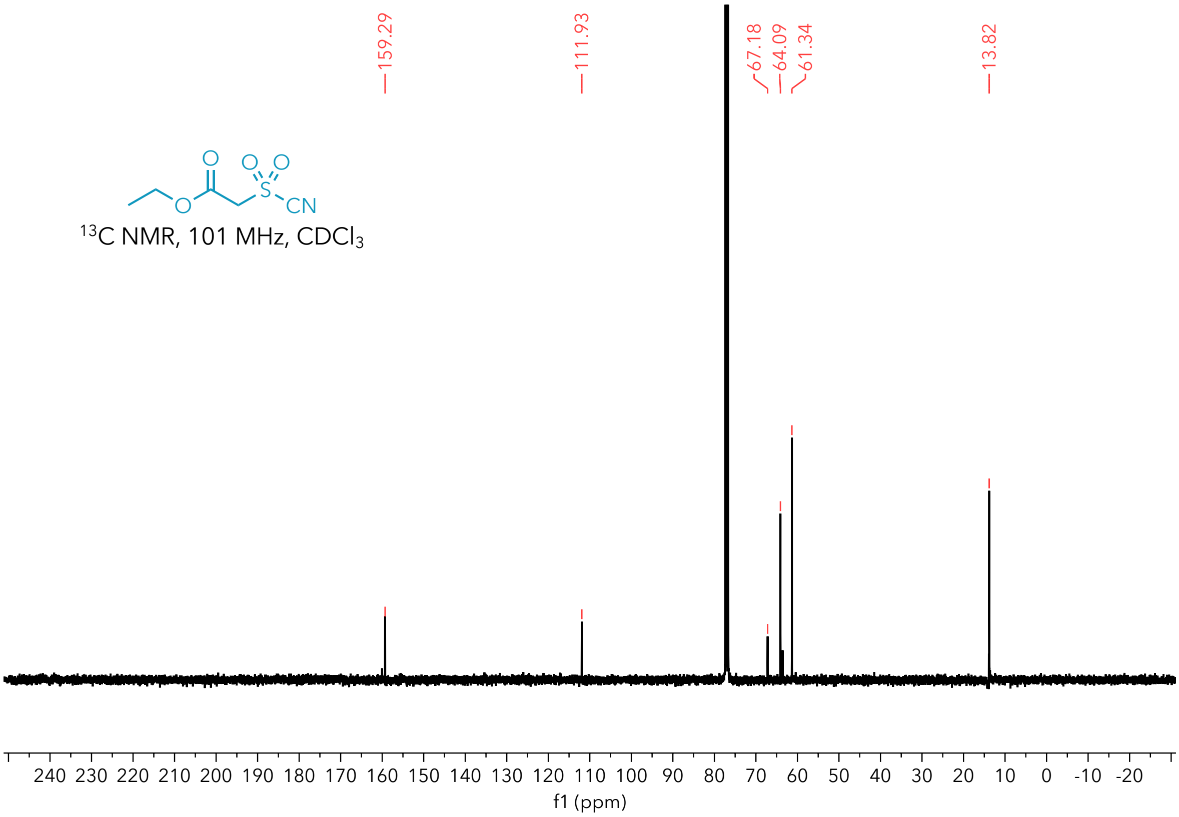
159.29
111.93
67.18
64.09
61.34
13.82
CN
13C NMR, 101 MHz, CDCl3
240 230 220 210 200 190 180 170 160 150 140 130 120 110 100 90 80 70 60 50 40 30 20 10 0 -10 -20
f1 (ppm)

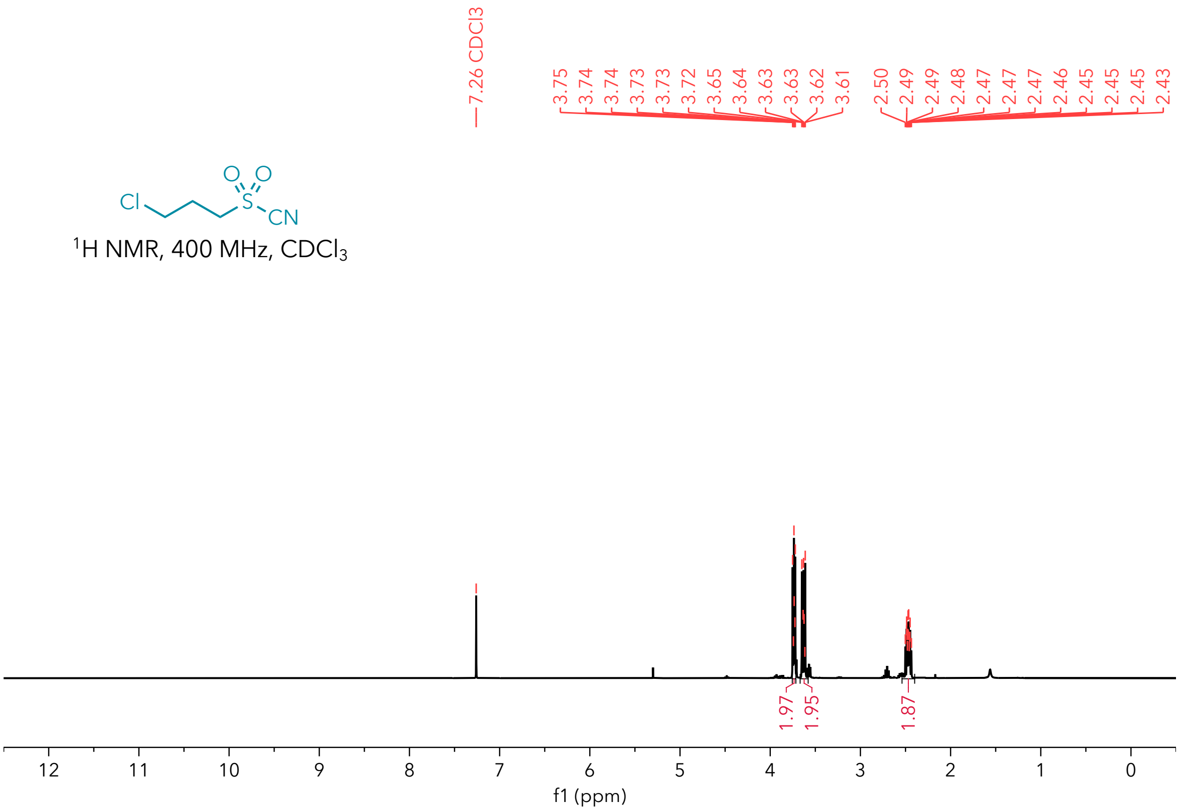
Cl
O O
S
CN
¹H NMR, 400 MHz, CDCl₃
7.26 CDCl3
3.75
3.74
3.74
3.73
3.73
3.72
3.65
3.64
3.63
3.63
3.62
3.61
2.50
2.49
2.49
2.48
2.47
2.47
2.47
2.46
2.45
2.45
2.45
2.43
1.97
1.95
1.87
12 11 10 9 8 7 6 5 4 3 2 1 0
f1 (ppm)

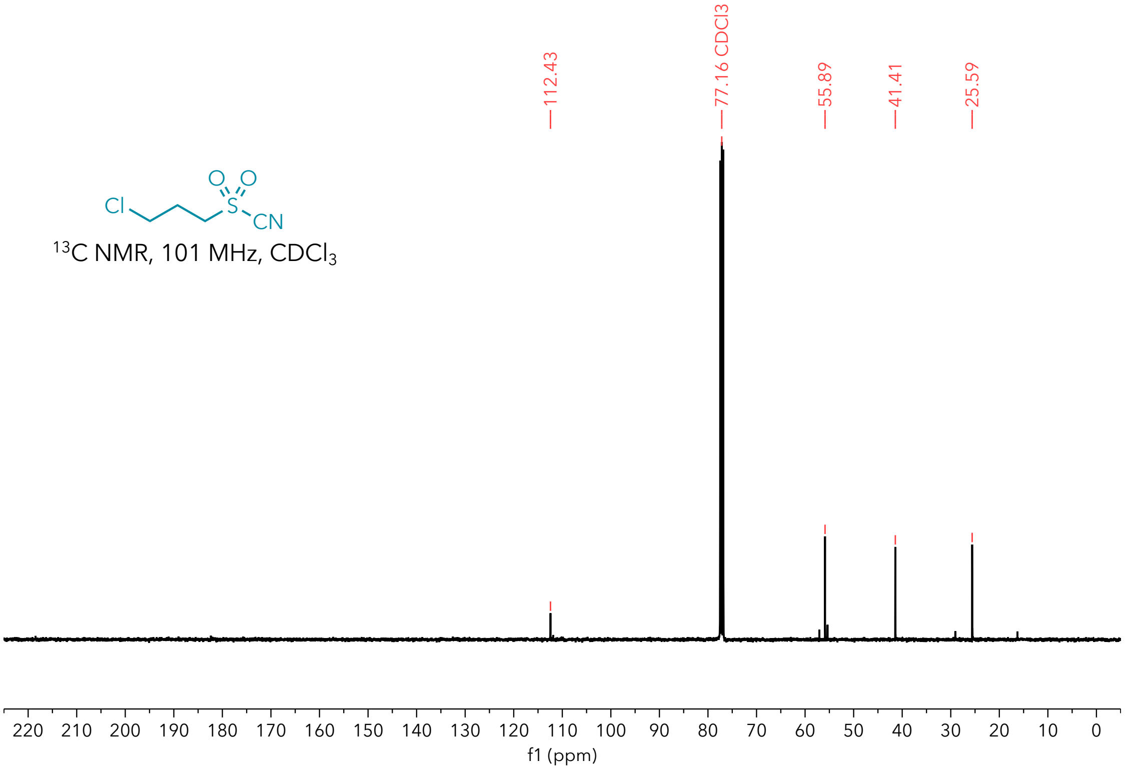
Cl
O O
S
CN
¹³C NMR, 101 MHz, CDCl₃
112.43
77.16 CDCl3
55.89
41.41
25.59
220 210 200 190 180 170 160 150 140 130 120 110 100 90 80 70 60 50 40 30 20 10 0
f1 (ppm)

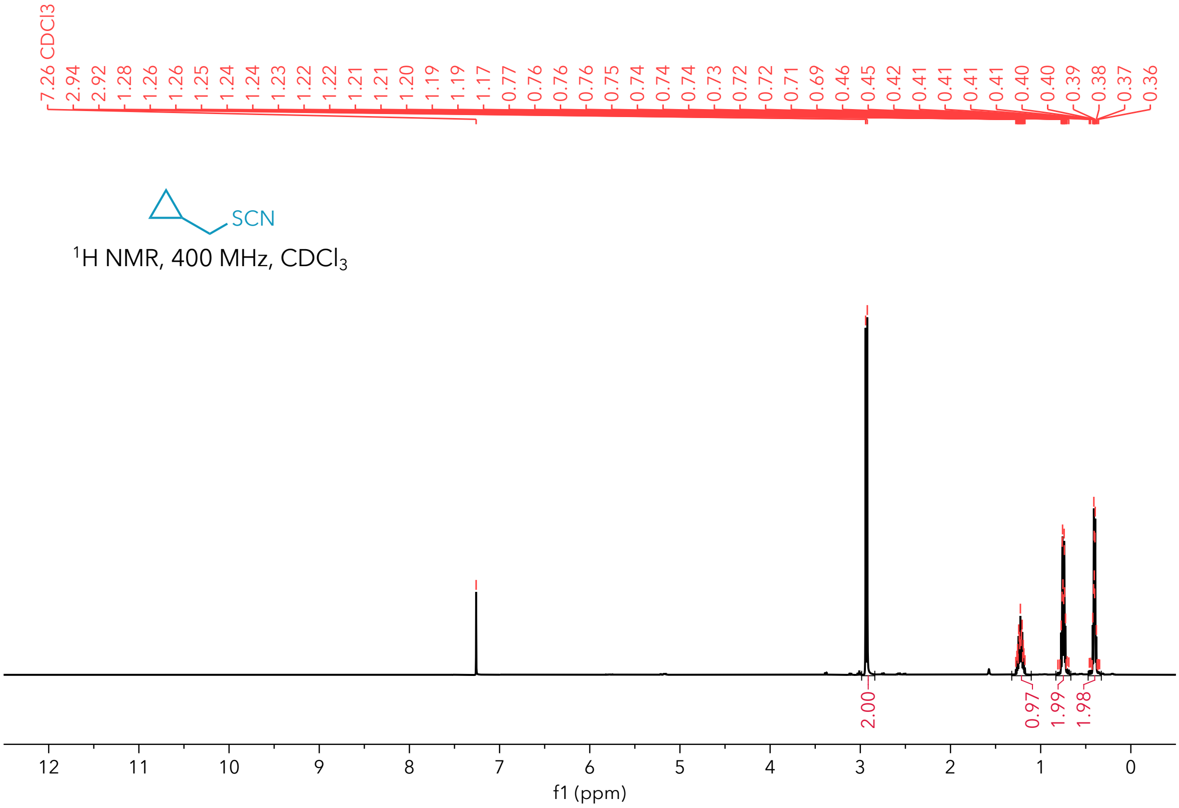

7.26 CDCl3
2.94
2.92
1.28
1.26
1.26
1.25
1.24
1.24
1.23
1.22
1.22
1.21
1.21
1.20
1.19
1.19
1.17
0.77
0.76
0.76
0.76
0.75
0.74
0.74
0.74
0.73
0.72
0.72
0.71
0.69
0.46
0.45
0.42
0.41
0.41
0.41
0.41
0.40
0.40
0.39
0.38
0.37
0.36
SCN
^{1}H NMR, 400 MHz, $CDCl_3$
2.00
0.97
1.99
1.98
12
11
10
9
8
7
6
5
4
3
2
1
0
f1 (ppm)

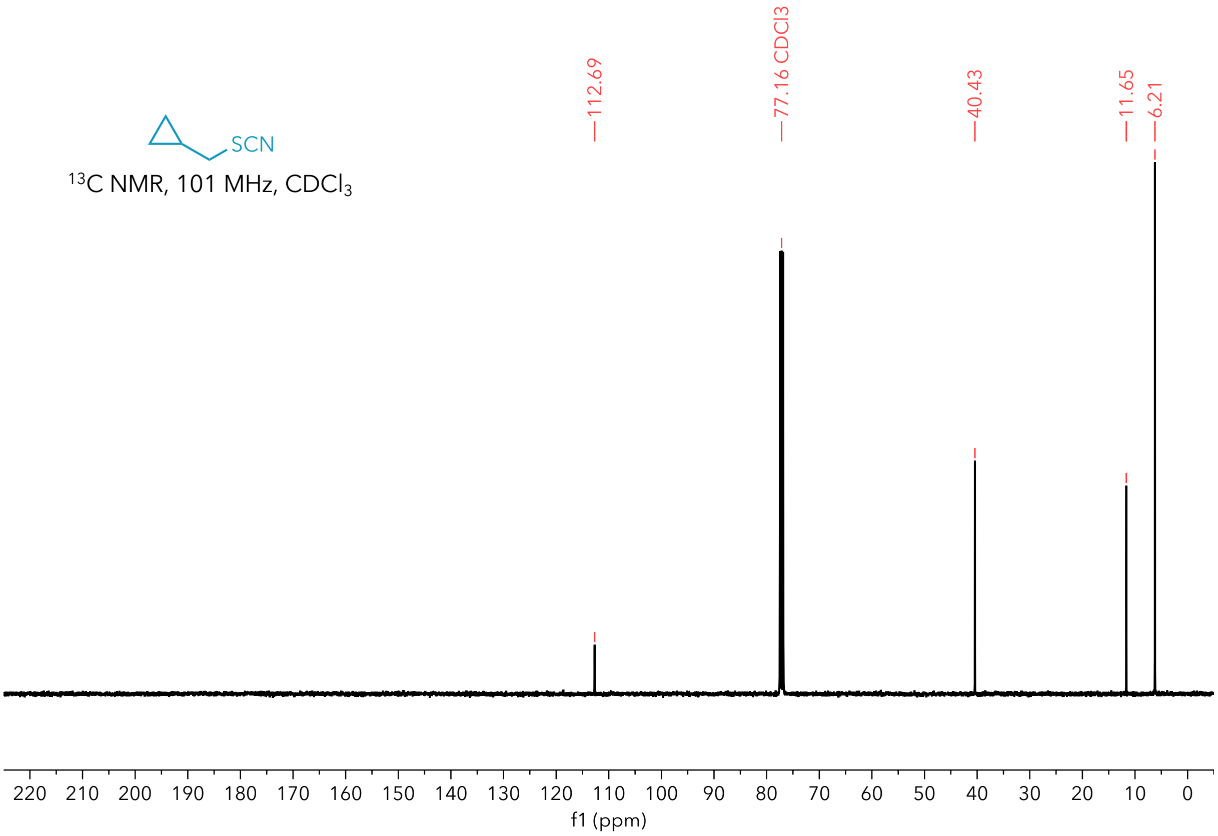

112.69
77.16 CDCl3
40.43
11.65
6.21
SCN
^{13}C NMR, 101 MHz, $CDCl_3$
220
210
200
190
180
170
160
150
140
130
120
110
100
90
80
70
60
50
40
30
20
10
0
f1 (ppm)

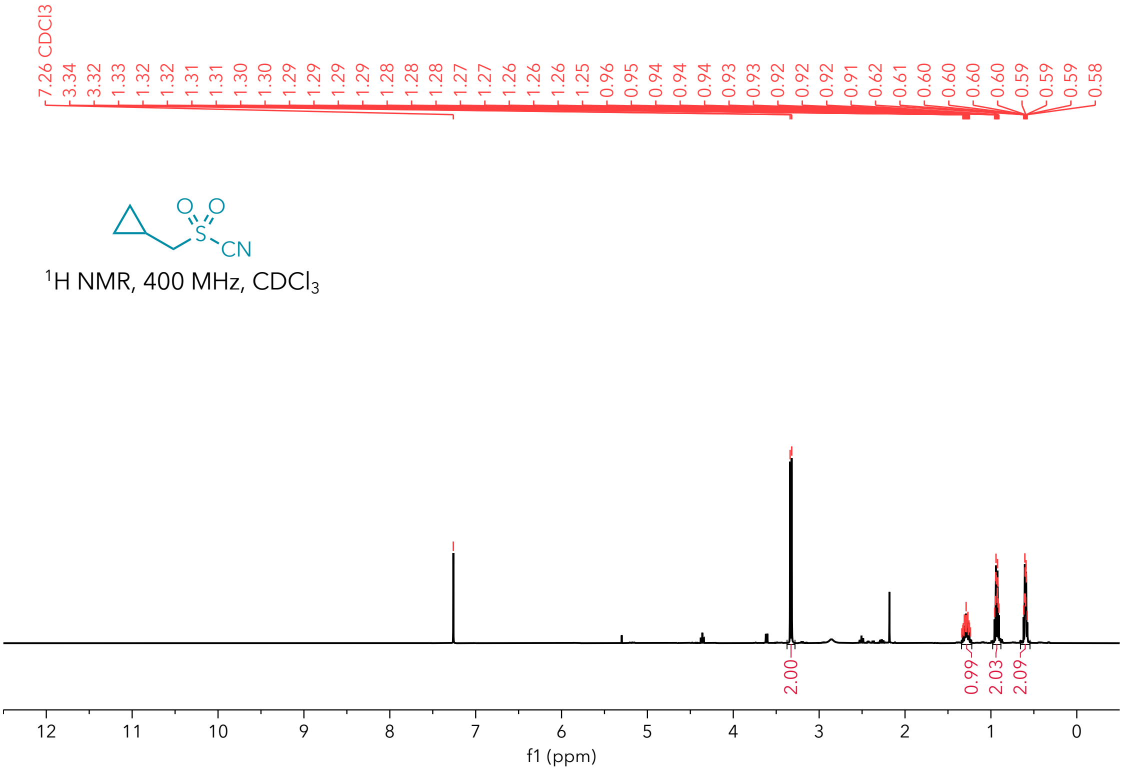
7.26 CDCl3
3.34
3.32
1.33
1.32
1.32
1.32
1.31
1.31
1.30
1.30
1.29
1.29
1.29
1.29
1.28
1.28
1.28
1.27
1.27
1.26
1.26
1.26
1.25
0.96
0.95
0.94
0.94
0.94
0.93
0.93
0.92
0.92
0.92
0.91
0.62
0.61
0.60
0.60
0.60
0.60
0.59
0.59
0.59
0.58
CN
S
O O
1H NMR, 400 MHz, CDCl3
2.00
0.99
2.03
2.09
f1 (ppm)

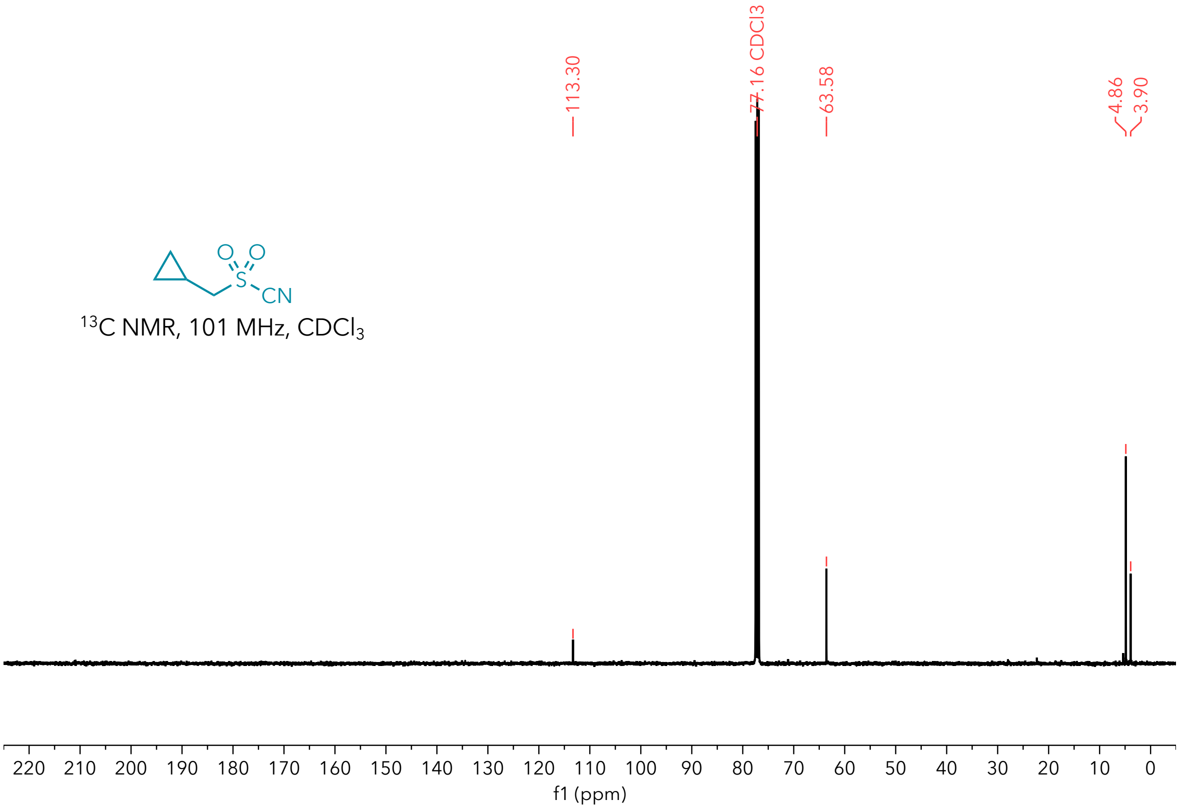
113.30
77.16 CDCl3
63.58
4.86
3.90
CN
S
O O
13C NMR, 101 MHz, CDCl3
f1 (ppm)

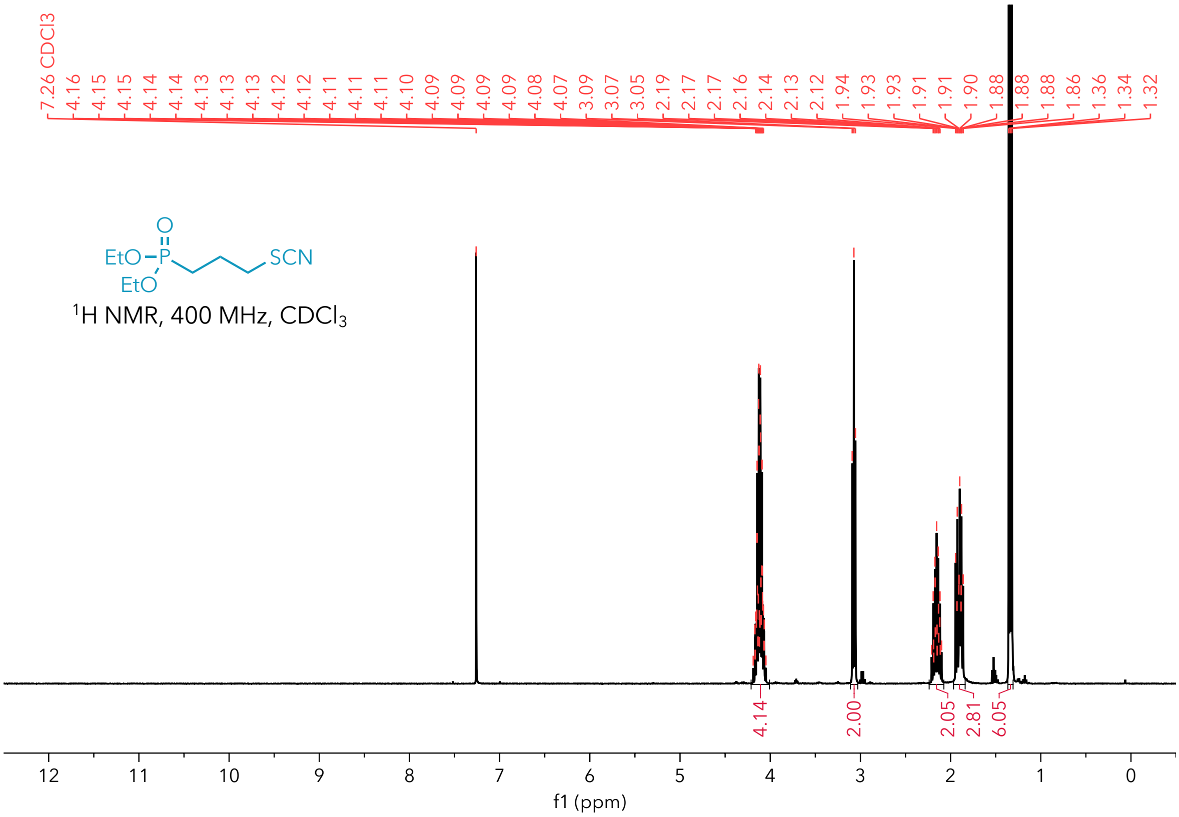
7.26 CDCl3
4.16 4.15 4.15 4.14 4.14 4.13 4.13 4.13 4.12 4.12 4.11 4.11 4.11 4.10 4.09 4.09 4.09 4.09 4.08 4.07 3.09 3.07 3.05 2.19 2.17 2.17 2.16 2.14 2.13 2.12 1.94 1.93 1.93 1.91 1.91 1.90 1.88 1.88 1.88 1.86 1.36 1.34 1.32
O
EtO
P
SCN
EtO
1H NMR, 400 MHz, CDCl3
4.14
2.00
2.05
2.81
6.05
12 11 10 9 8 7 6 5 4 3 2 1 0
f1 (ppm)

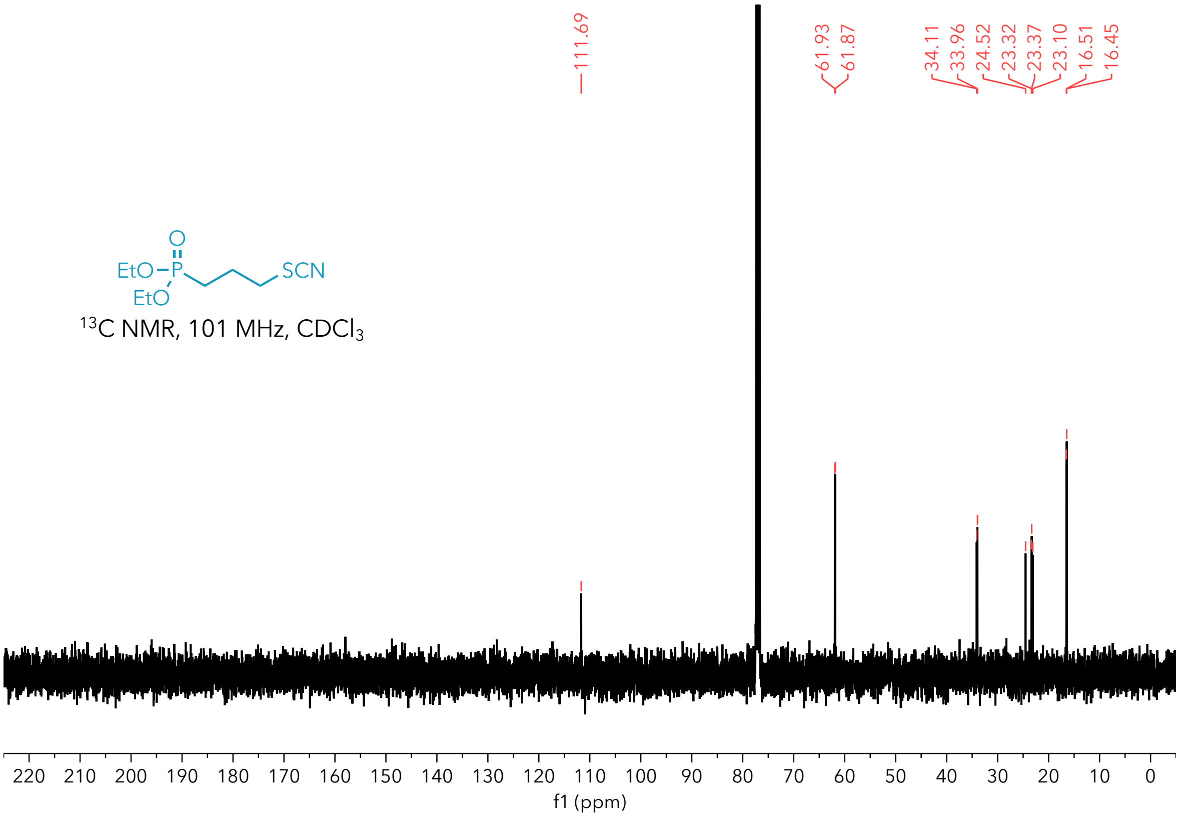
111.69
61.93
61.87
34.11 33.96 24.52 23.32 23.37 23.10 16.51 16.45
O
EtO
P
SCN
EtO
13C NMR, 101 MHz, CDCl3
220 210 200 190 180 170 160 150 140 130 120 110 100 90 80 70 60 50 40 30 20 10 0
f1 (ppm)

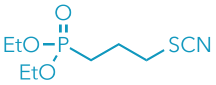

^{31}P NMR, 162 MHz, $CDCl_3$

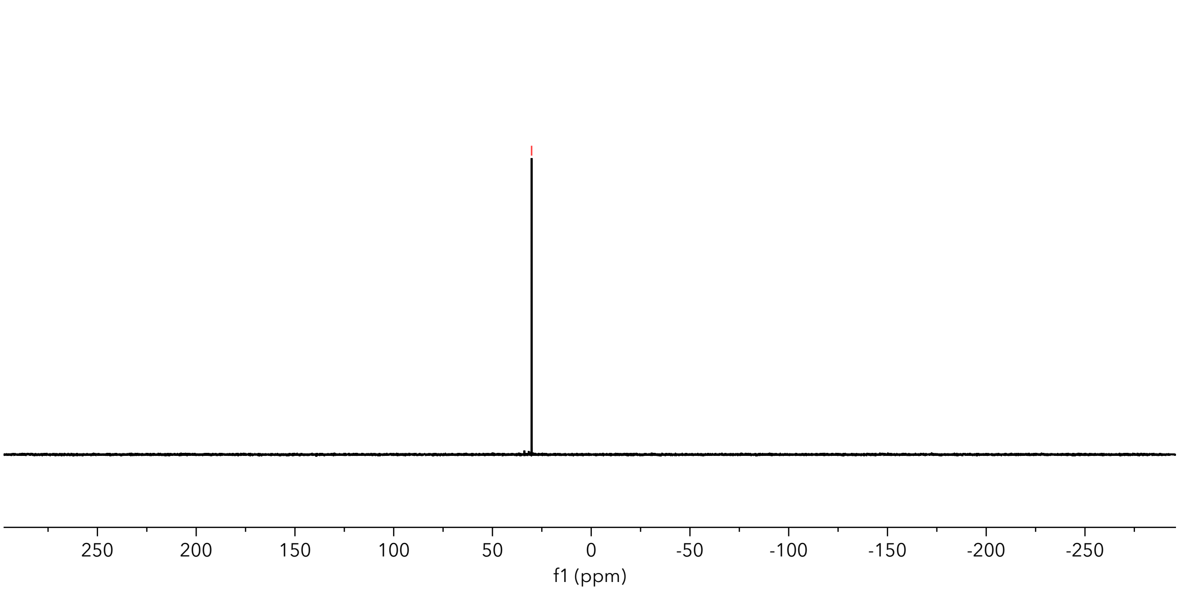

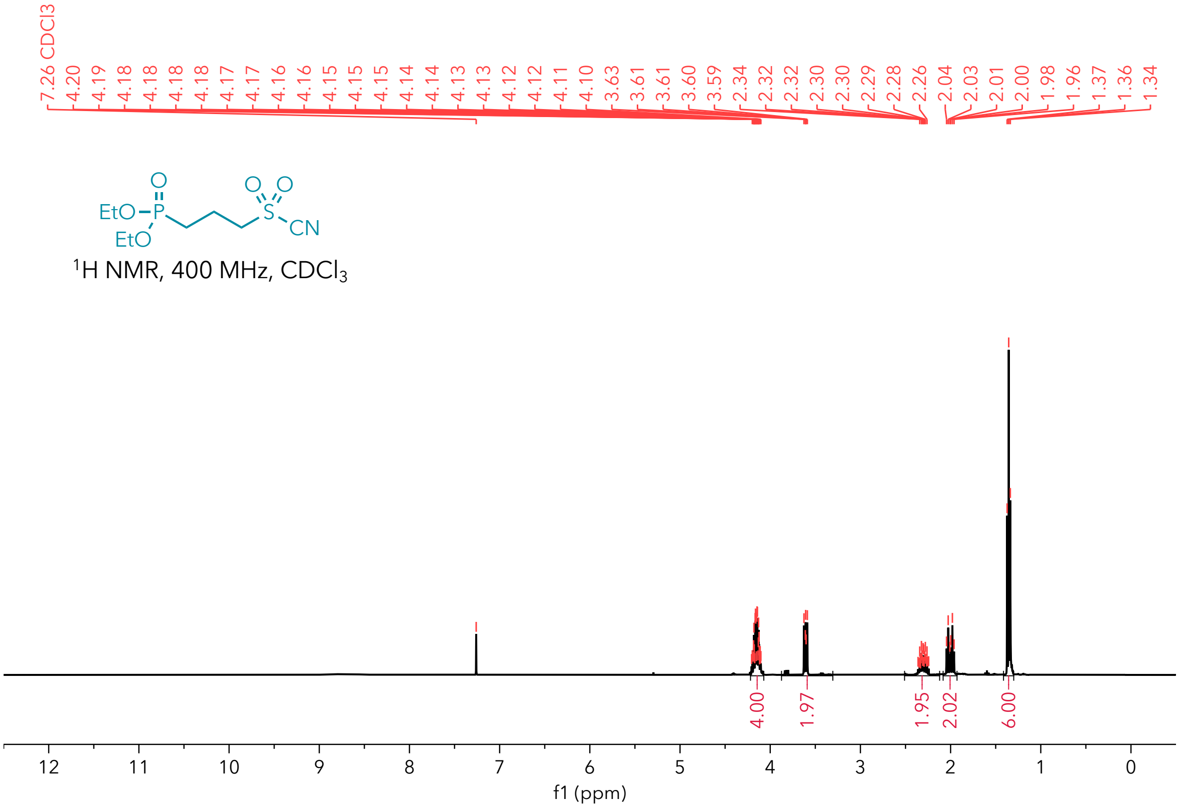
7.26 CDCl3
4.20
4.19
4.18
4.18
4.18
4.18
4.17
4.17
4.16
4.16
4.15
4.15
4.15
4.14
4.14
4.13
4.13
4.12
4.12
4.11
4.10
3.63
3.61
3.61
3.60
3.59
2.34
2.32
2.32
2.30
2.30
2.29
2.28
2.26
2.04
2.03
2.01
2.00
1.98
1.96
1.37
1.36
1.34
EtO
EtO
P
O
S
O O
CN
^{1}H NMR, 400 MHz, $CDCl_3$
4.00
1.97
1.95
2.02
6.00
f1 (ppm)

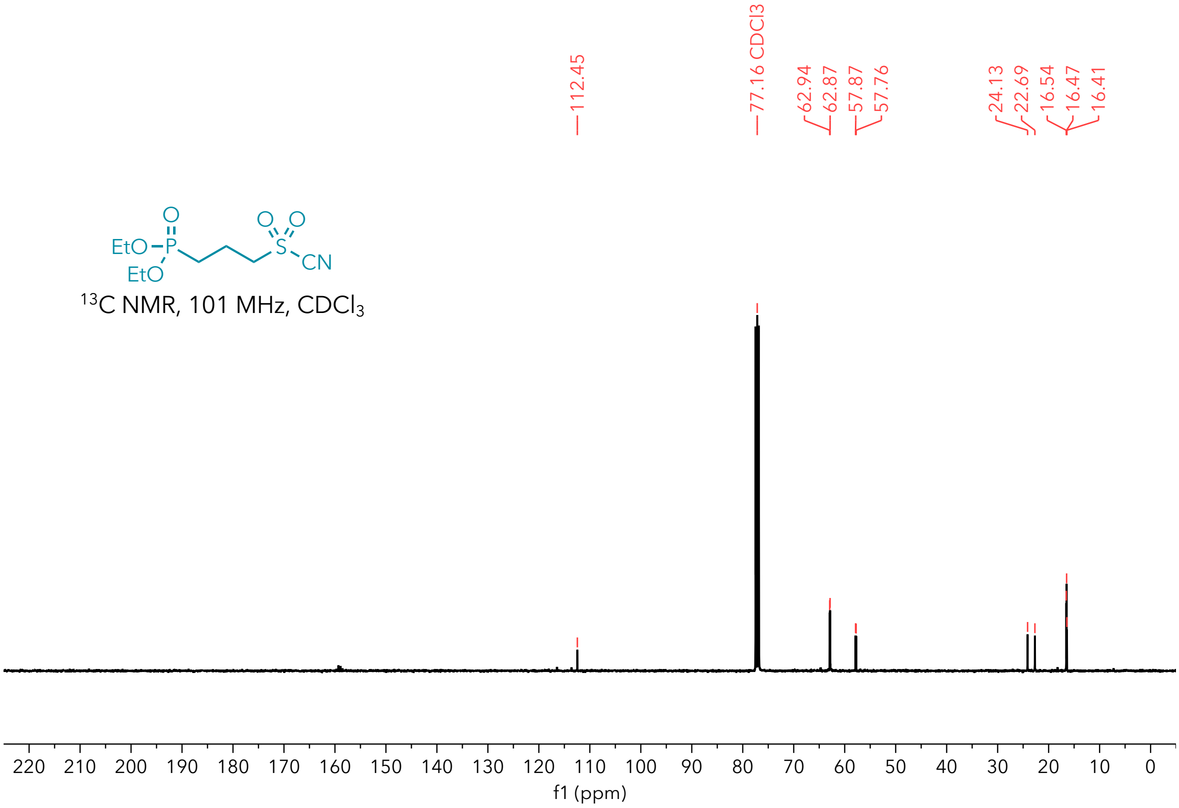
112.45
77.16 CDCl3
62.94
62.87
57.87
57.76
24.13
22.69
16.54
16.47
16.41
EtO
EtO
P
O
S
O O
CN
^{13}C NMR, 101 MHz, $CDCl_3$
f1 (ppm)

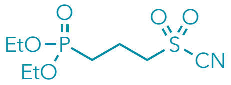

^{31}P NMR, 162 MHz, $CDCl_3$

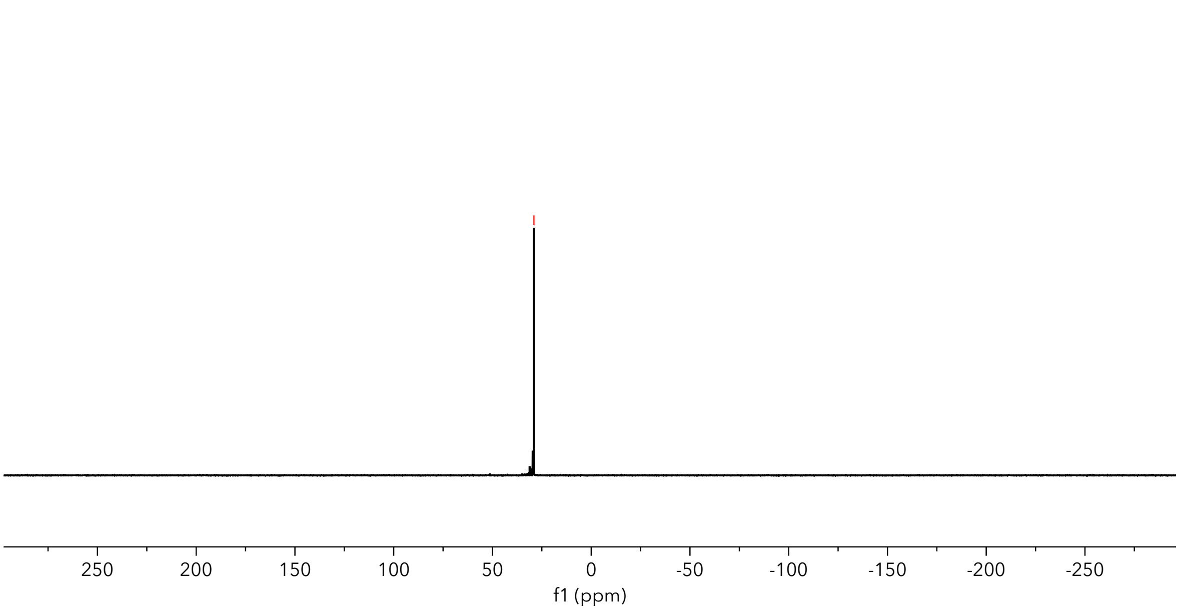

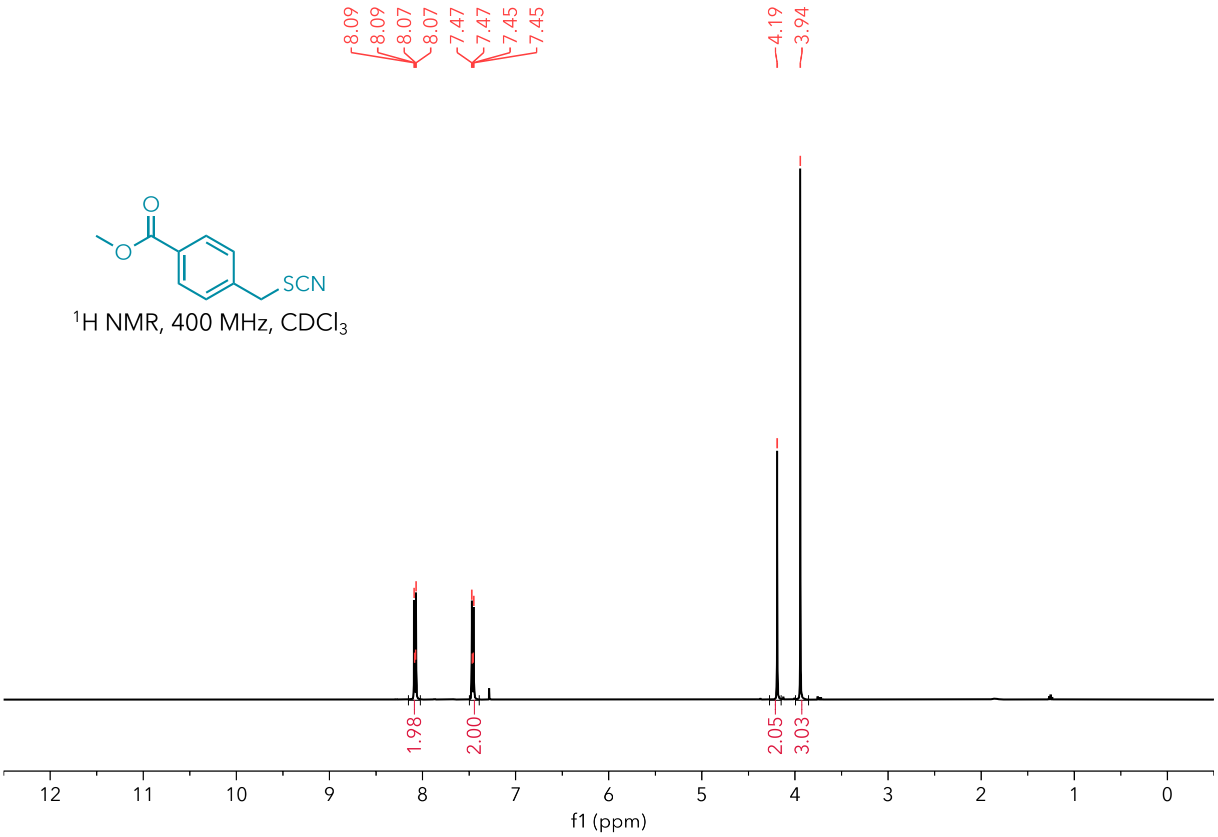

8.09
8.09
8.07
8.07
7.47
7.47
7.45
7.45
4.19
3.94
SCN
^{1}H NMR, 400 MHz, $CDCl_3$
1.98
2.00
2.05
3.03
12 11 10 9 8 7 6 5 4 3 2 1 0
f1 (ppm)

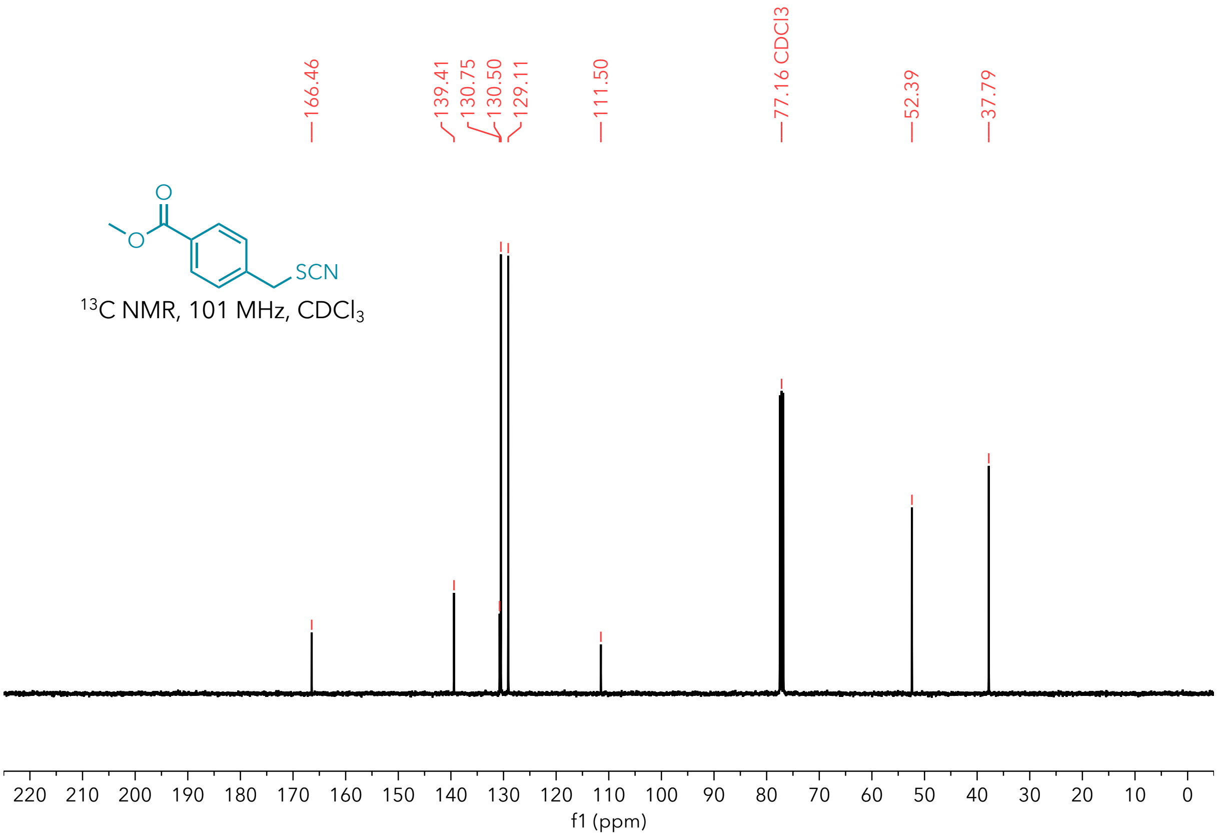

166.46
139.41
130.75
130.50
129.11
111.50
77.16 CDCl3
52.39
37.79
SCN
^{13}C NMR, 101 MHz, $CDCl_3$
220 210 200 190 180 170 160 150 140 130 120 110 100 90 80 70 60 50 40 30 20 10 0
f1 (ppm)

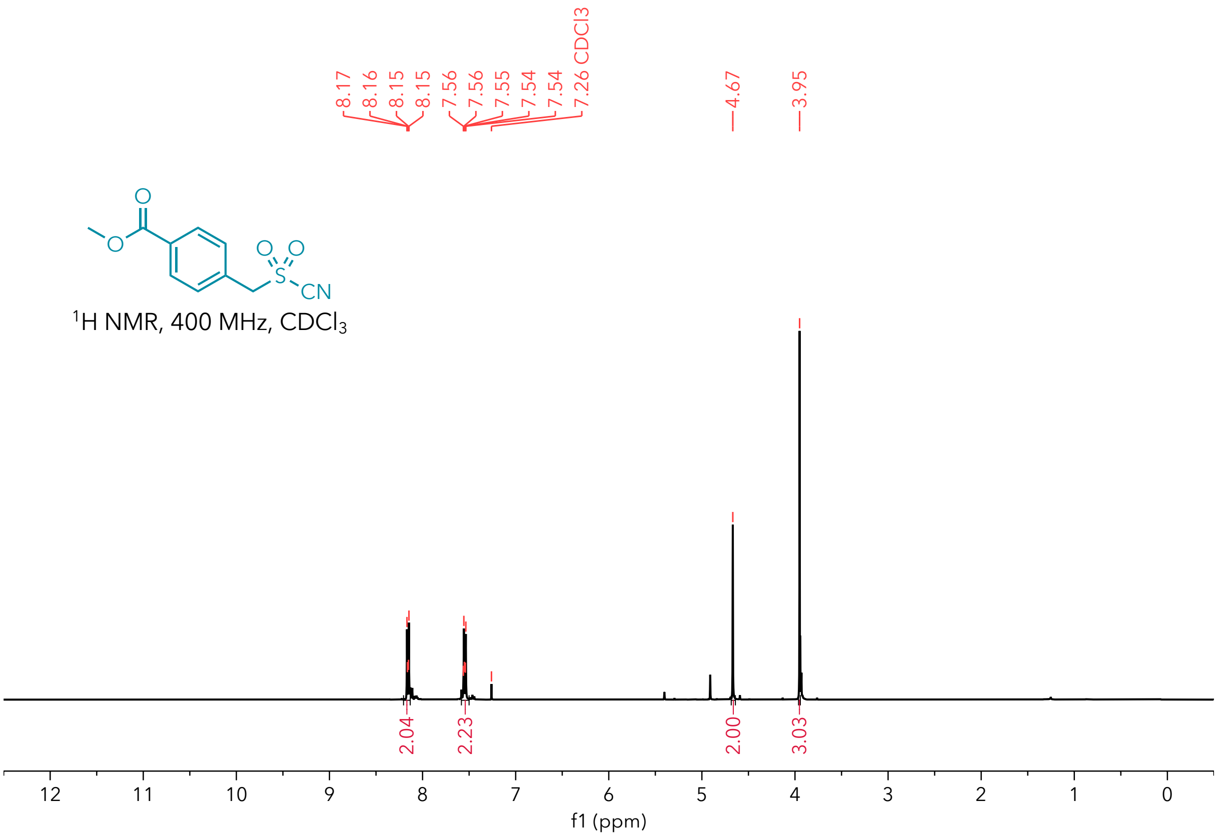

8.17
8.16
8.15
8.15
7.56
7.56
7.55
7.54
7.54
7.26 CDCl3
4.67
3.95
CN
^{1}H NMR, 400 MHz, $CDCl_3$
2.04
2.23
2.00
3.03
f1 (ppm)

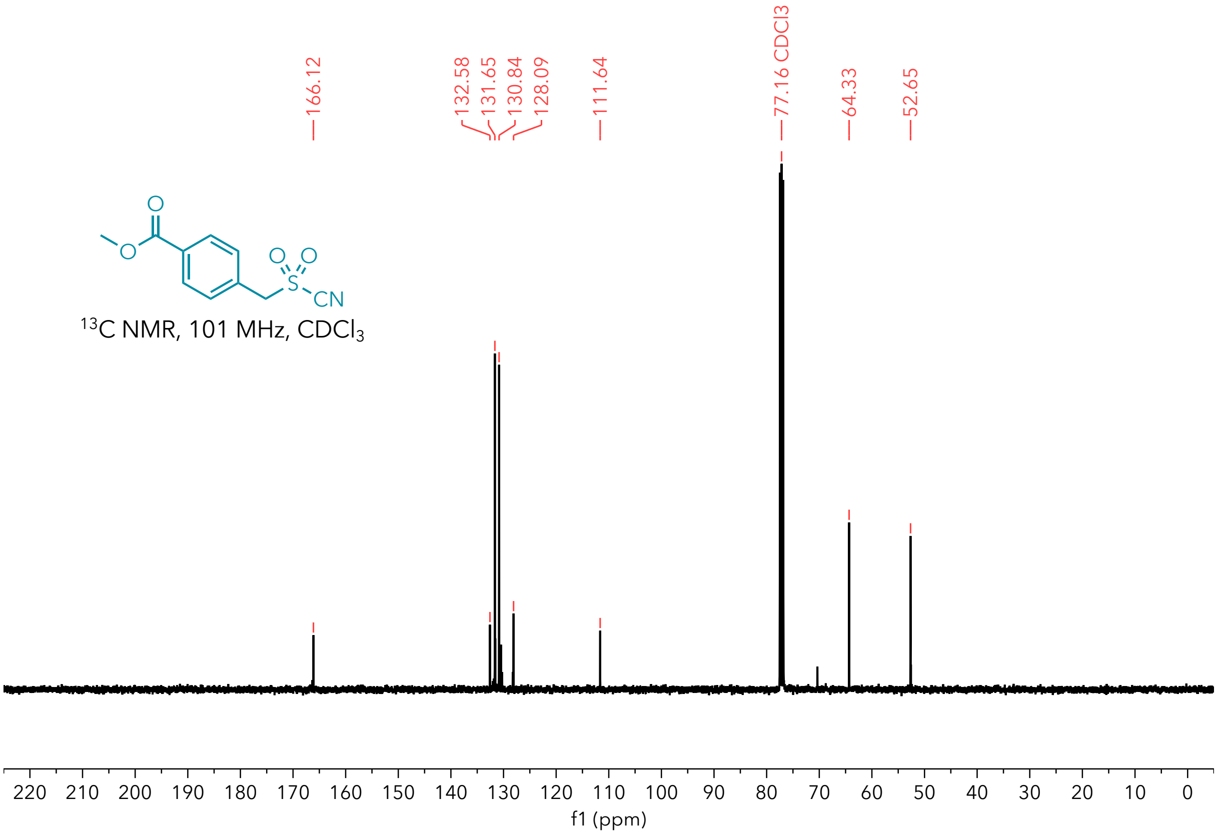

166.12
132.58
131.65
130.84
128.09
111.64
77.16 CDCl3
64.33
52.65
CN
^{13}C NMR, 101 MHz, $CDCl_3$
f1 (ppm)

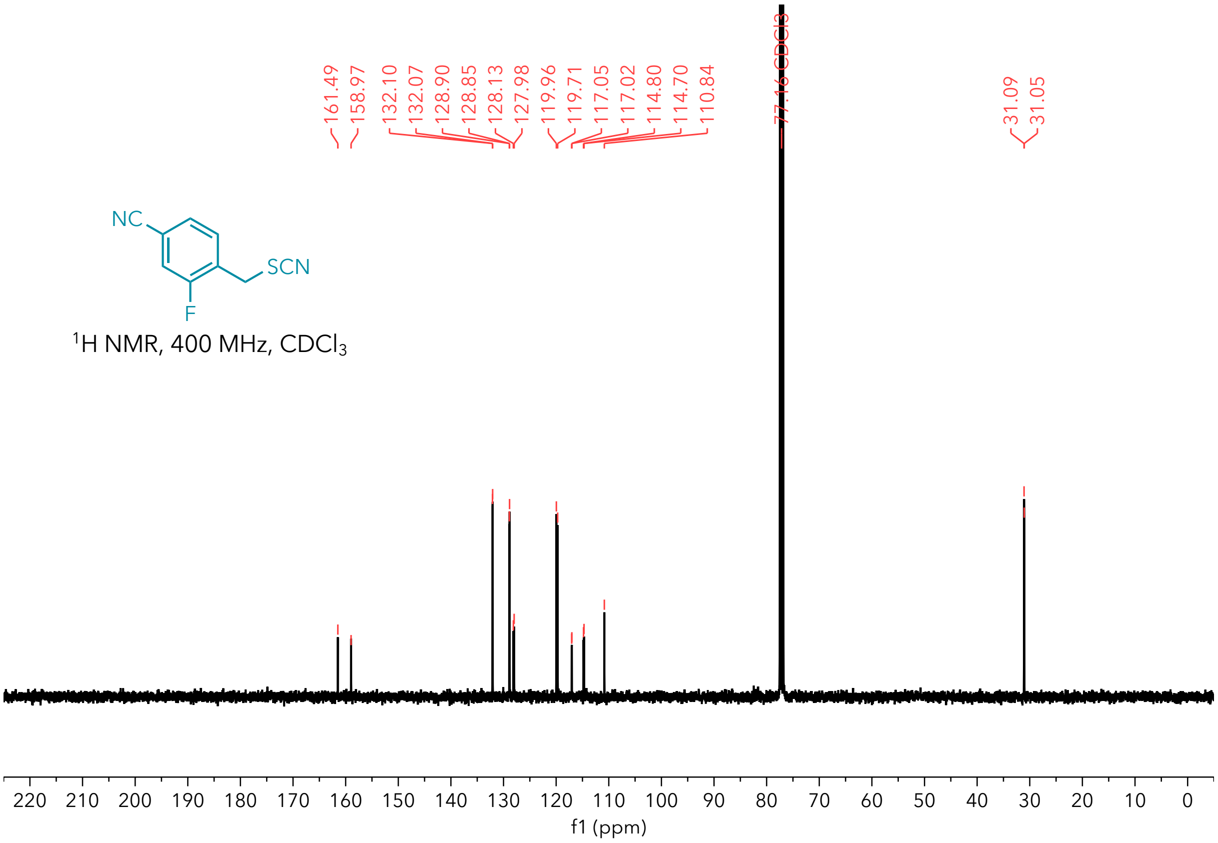

^{1}H NMR, 400 MHz, $CDCl_3$

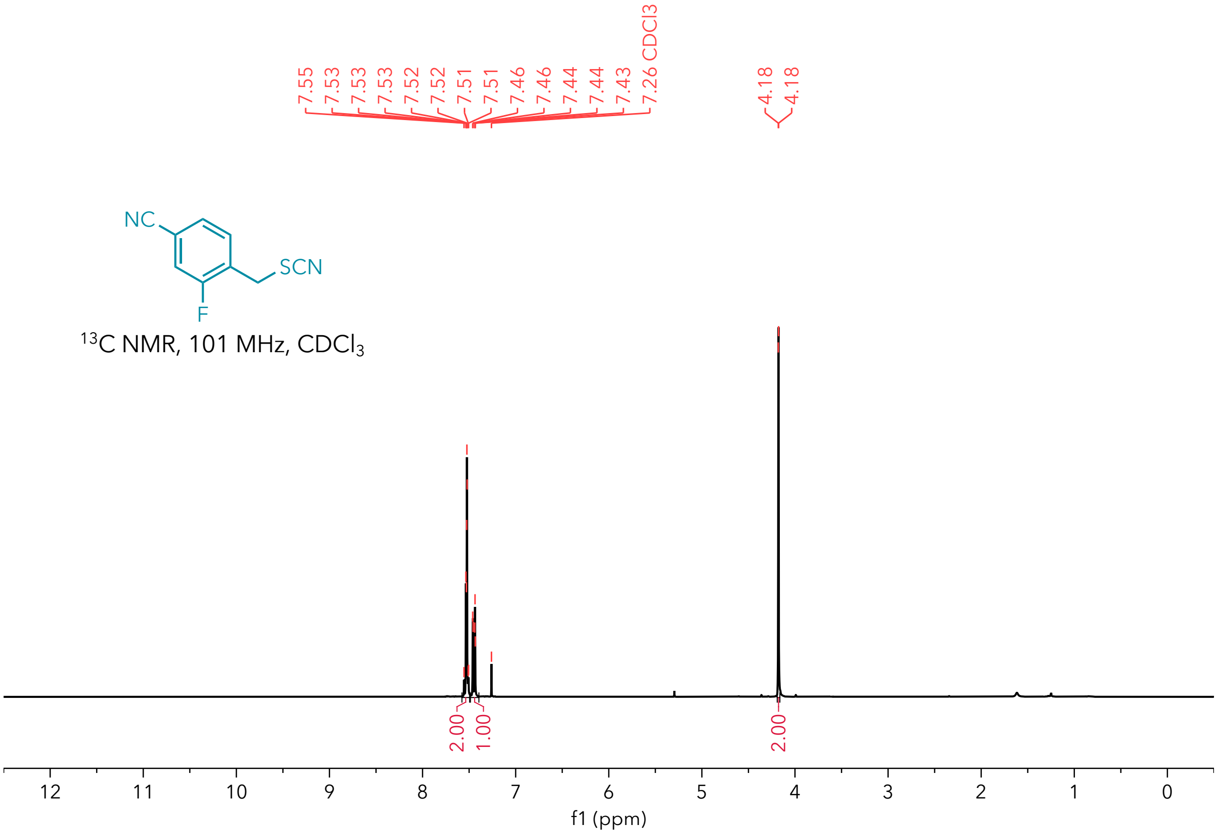

^{13}C NMR, 101 MHz, $CDCl_3$

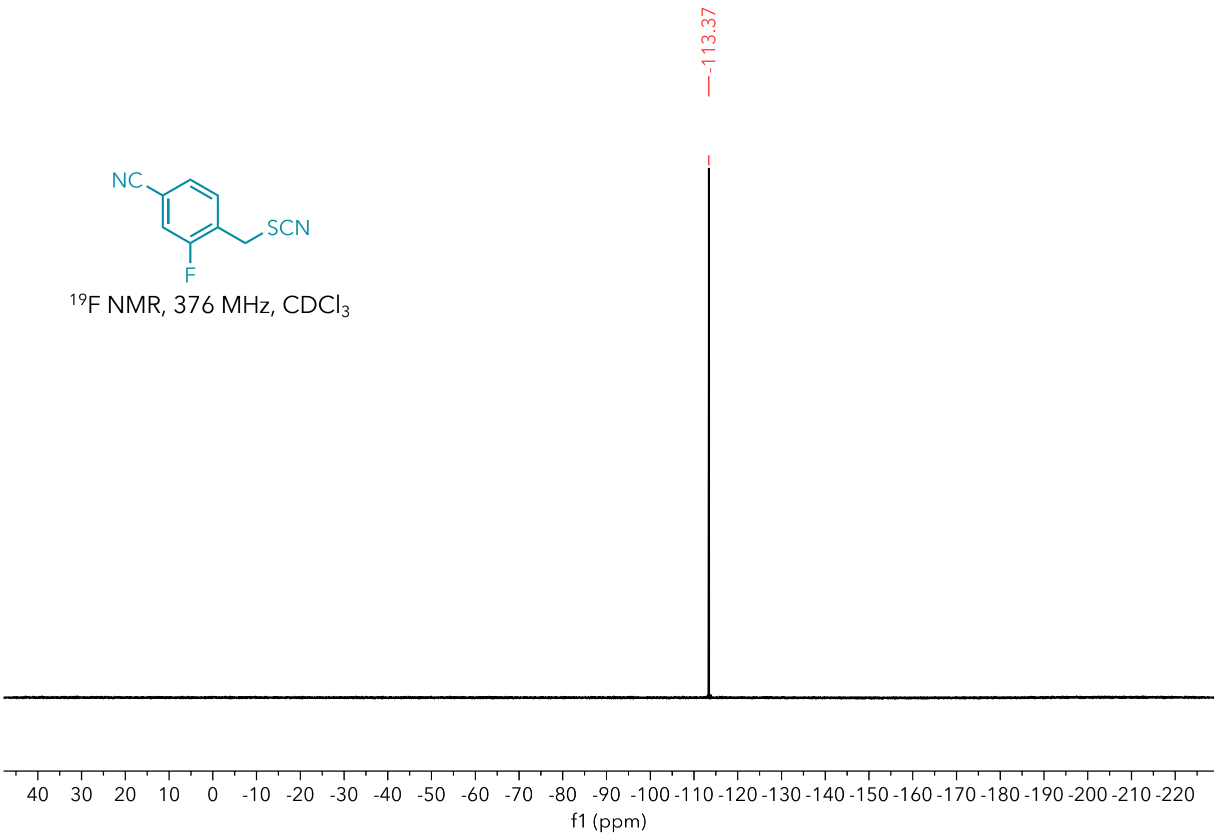

-113.37
NC
SCN
F
¹⁹F NMR, 376 MHz, CDCl₃
40 30 20 10 0 -10 -20 -30 -40 -50 -60 -70 -80 -90 -100 -110 -120 -130 -140 -150 -160 -170 -180 -190 -200 -210 -220
f1 (ppm)

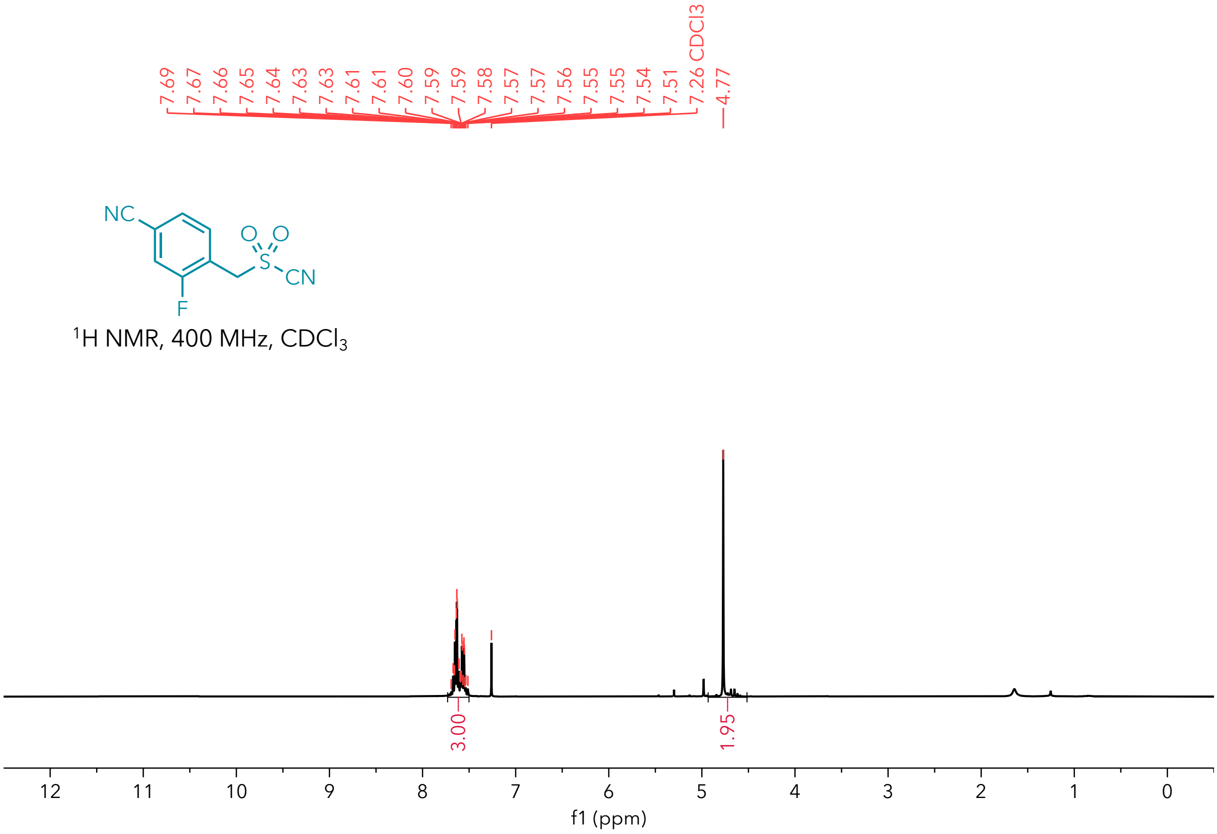

^{1}H NMR, 400 MHz, $CDCl_3$

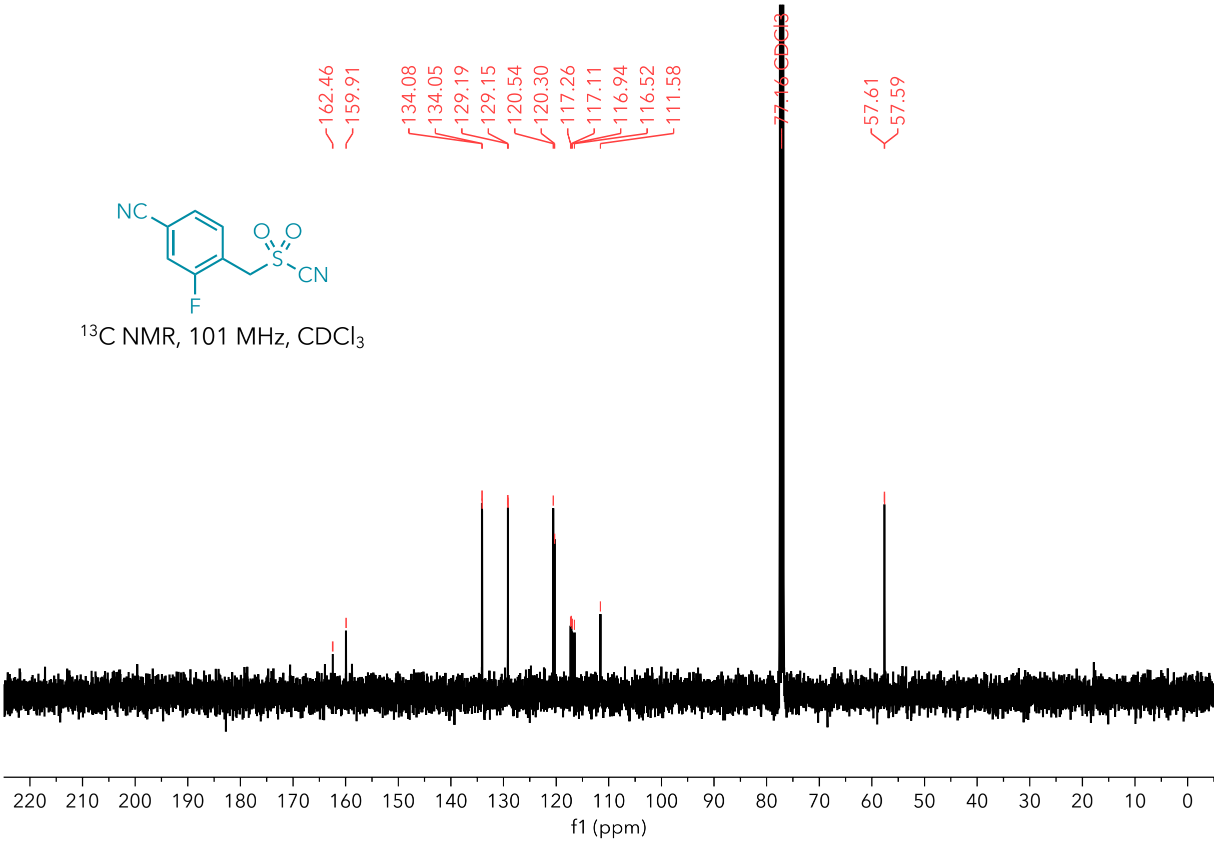

^{13}C NMR, 101 MHz, $CDCl_3$

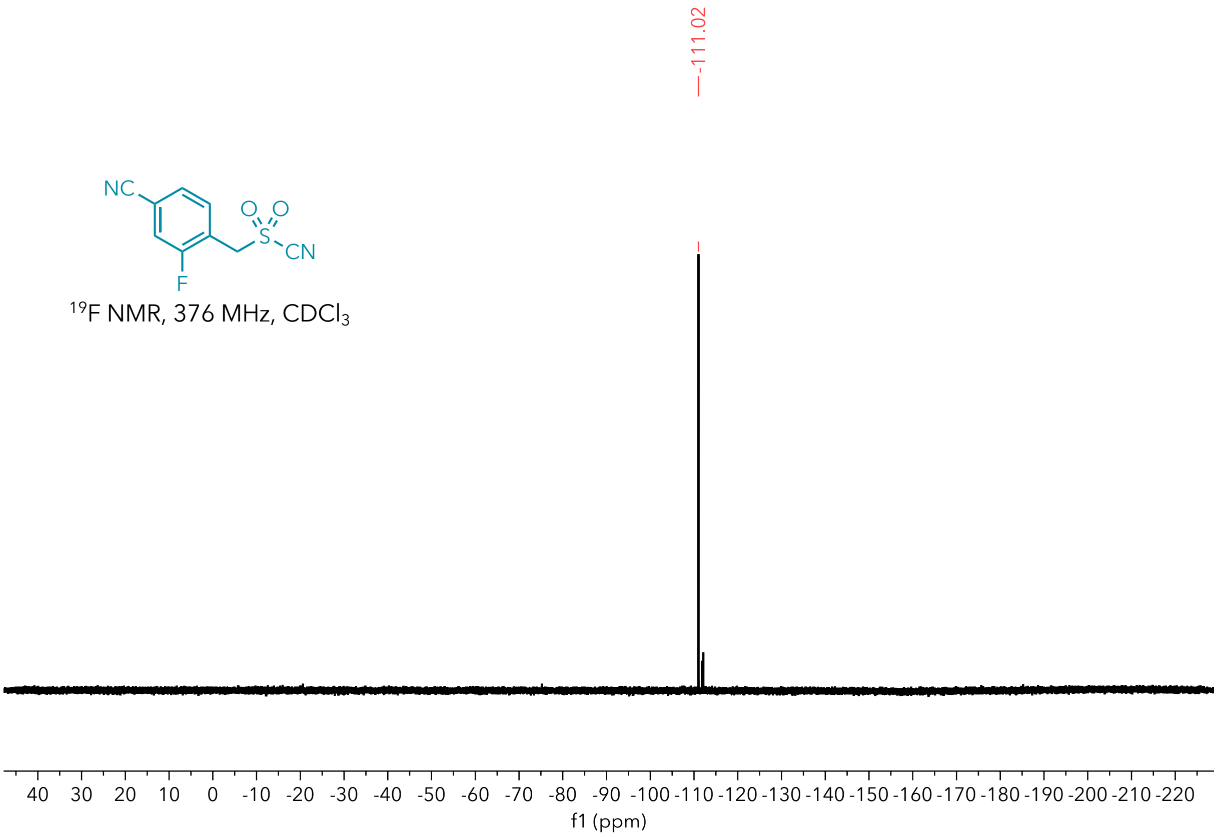
-111.02
NC
O O
S
CN
F
^{19}F NMR, 376 MHz, CDCl$_3$
40 30 20 10 0 -10 -20 -30 -40 -50 -60 -70 -80 -90 -100 -110 -120 -130 -140 -150 -160 -170 -180 -190 -200 -210 -220
f1 (ppm)

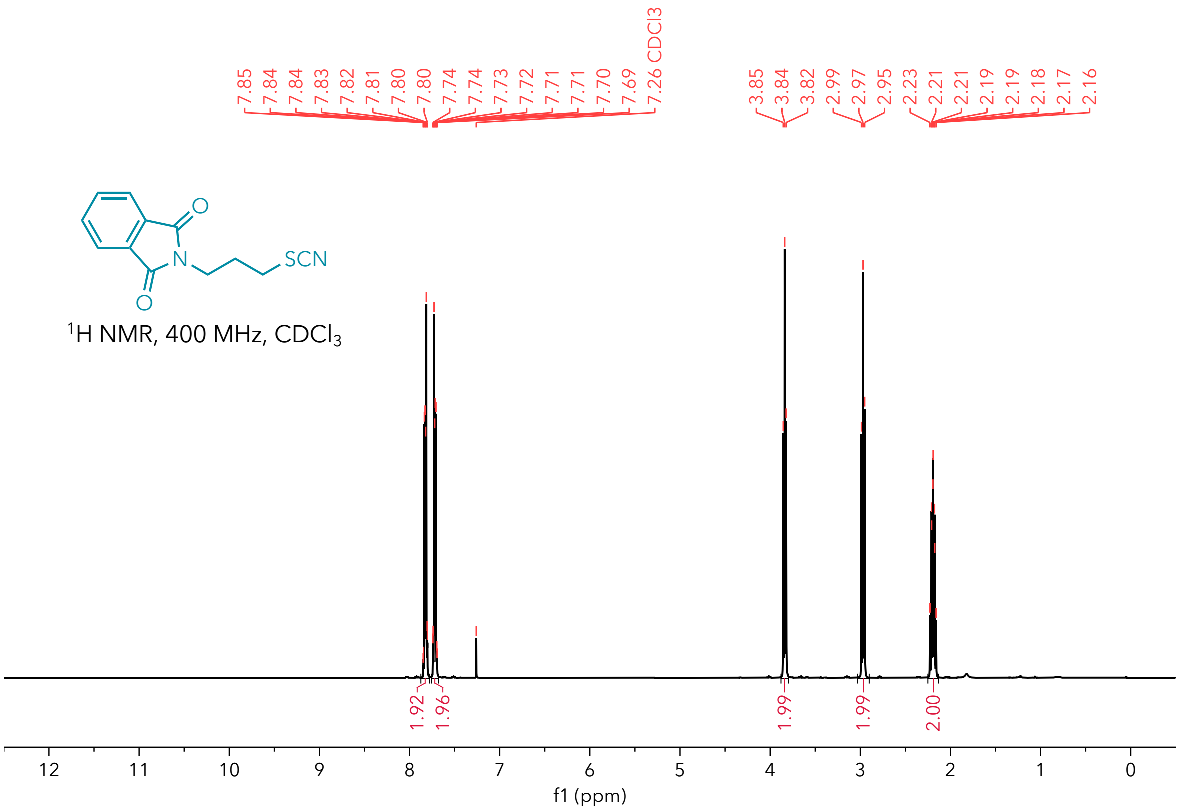
7.85 7.84 7.84 7.83 7.82 7.81 7.80 7.80 7.74 7.74 7.73 7.72 7.71 7.71 7.70 7.69 7.26 CDCl3
3.85 3.84 3.82 2.99 2.97 2.95 2.23 2.21 2.21 2.19 2.19 2.18 2.17 2.16
O
N
SCN
O
^{1}H NMR, 400 MHz, $CDCl_3$
1.92
1.96
1.99
1.99
2.00
12 11 10 9 8 7 6 5 4 3 2 1 0
f1 (ppm)

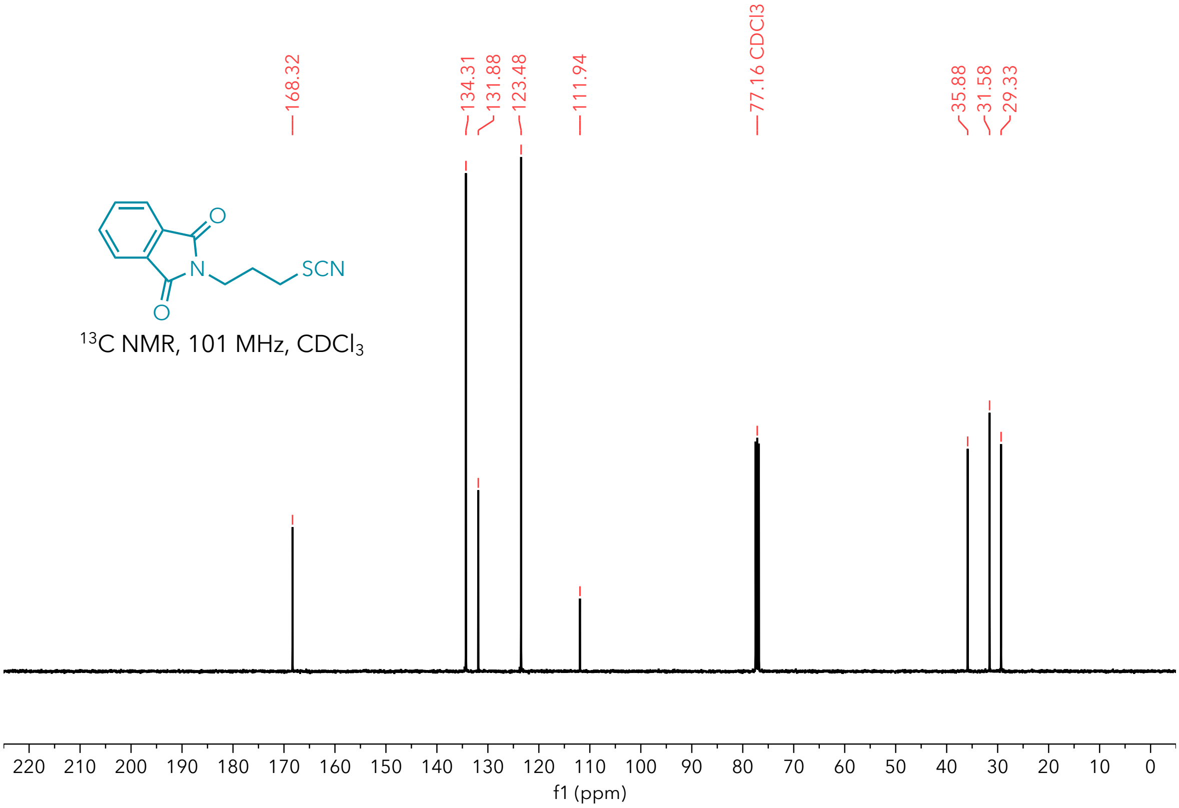
168.32
134.31 131.88 123.48
111.94
77.16 CDCl3
35.88 31.58 29.33
O
N
SCN
O
^{13}C NMR, 101 MHz, $CDCl_3$
220 210 200 190 180 170 160 150 140 130 120 110 100 90 80 70 60 50 40 30 20 10 0
f1 (ppm)

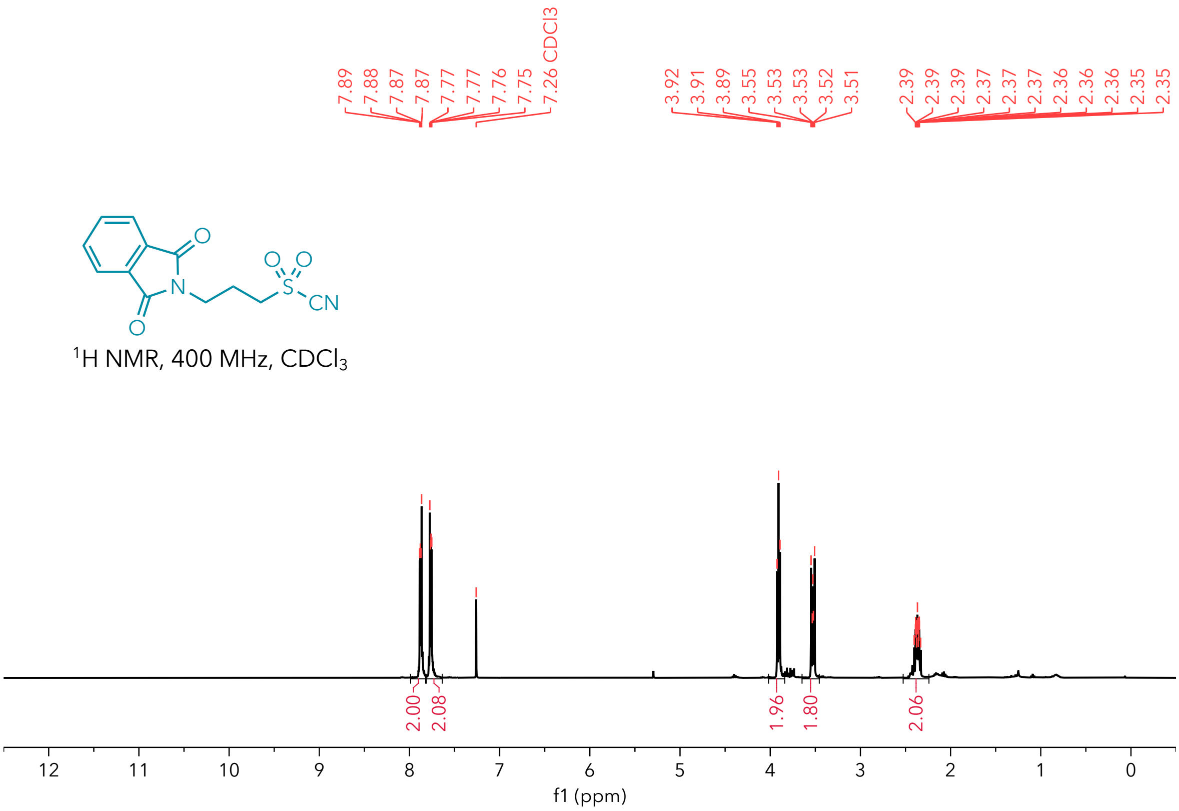

7.89
7.88
7.87
7.87
7.77
7.77
7.76
7.75
7.26 CDCl3
3.92
3.91
3.89
3.55
3.53
3.53
3.52
3.51
2.39
2.39
2.39
2.37
2.37
2.37
2.36
2.36
2.36
2.35
2.35
O
O
N
O
O
S
CN
1H NMR, 400 MHz, CDCl3
2.00
2.08
1.96
1.80
2.06
12 11 10 9 8 7 6 5 4 3 2 1 0
f1 (ppm)

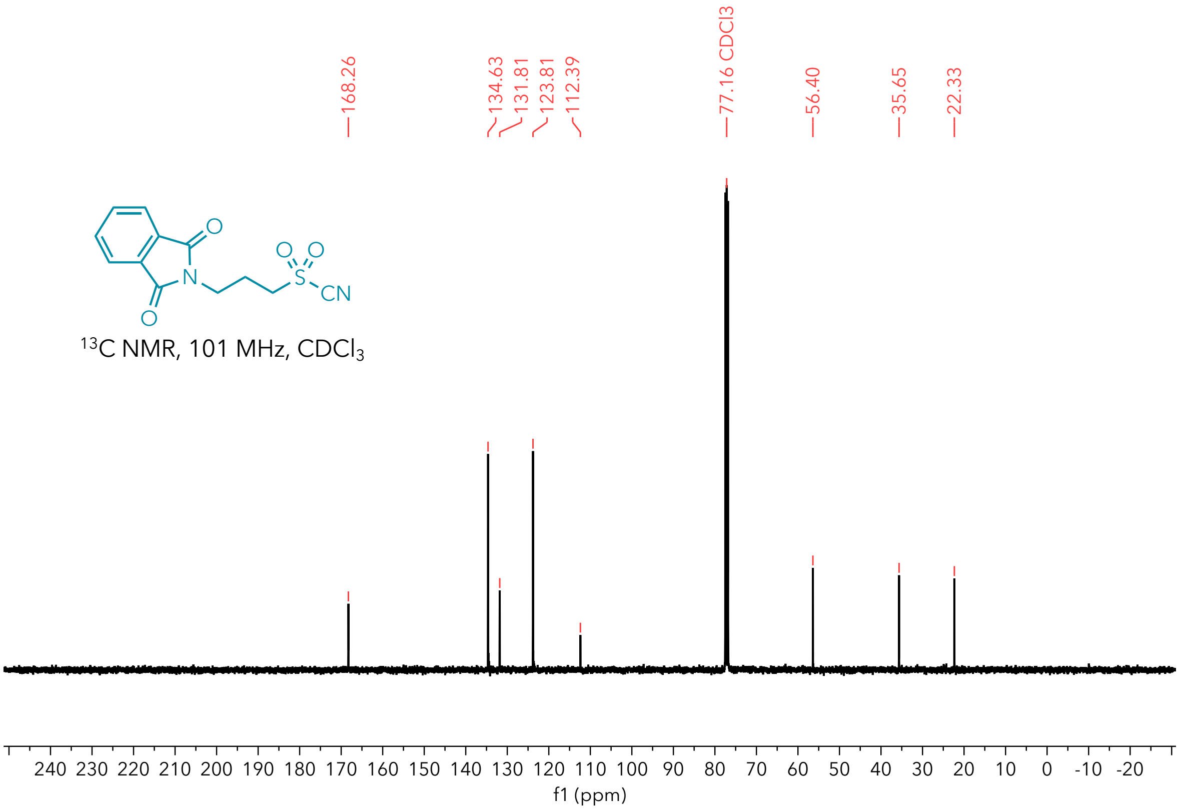

168.26
134.63
131.81
123.81
112.39
77.16 CDCl3
56.40
35.65
22.33
O
O
N
O
O
S
CN
13C NMR, 101 MHz, CDCl3
240 230 220 210 200 190 180 170 160 150 140 130 120 110 100 90 80 70 60 50 40 30 20 10 0 -10 -20
f1 (ppm)

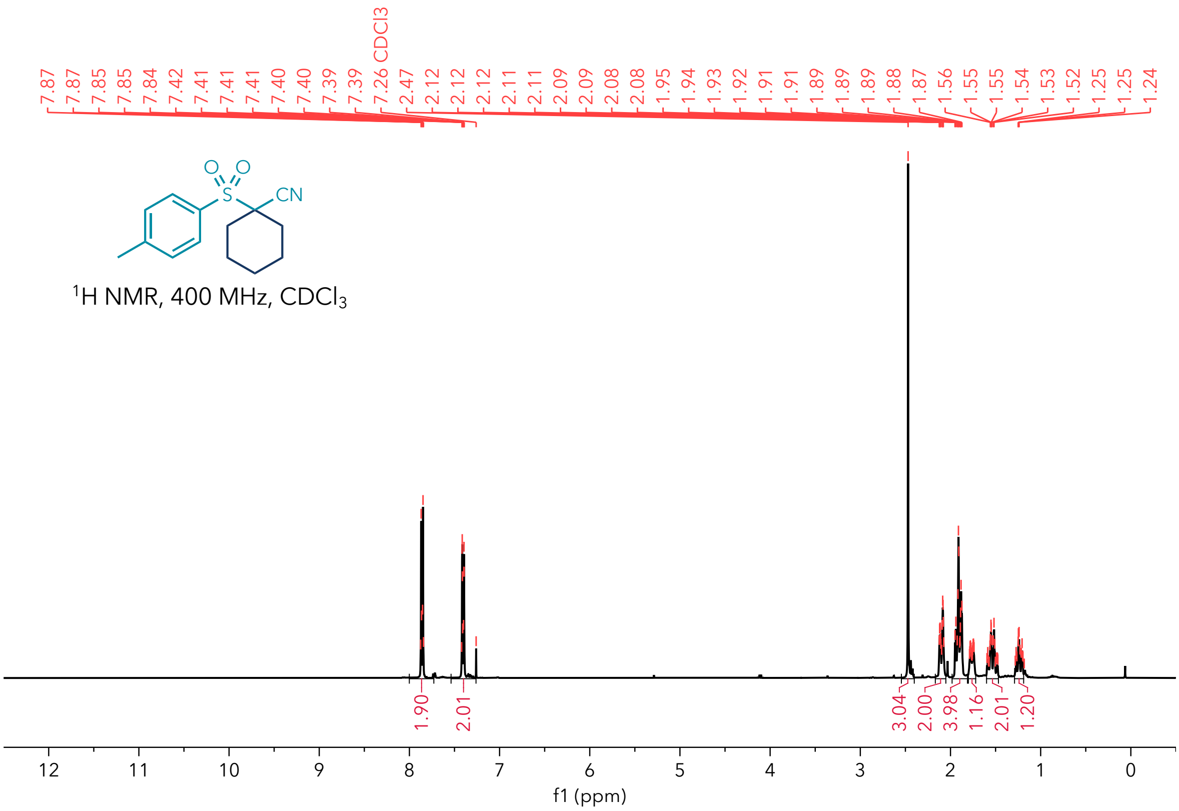
CN
1H NMR, 400 MHz, CDCl3
7.87 7.87 7.85 7.85 7.84 7.42 7.41 7.41 7.41 7.40 7.40 7.39 7.39 7.26 CDCl3 2.47 2.12 2.12 2.12 2.11 2.11 2.09 2.09 2.08 2.08 1.95 1.94 1.93 1.92 1.91 1.91 1.89 1.89 1.89 1.88 1.87 1.56 1.55 1.55 1.54 1.53 1.52 1.25 1.25 1.24
1.90 2.01 3.04 2.00 3.98 1.16 2.01 1.20
12 11 10 9 8 7 6 5 4 3 2 1 0
f1 (ppm)

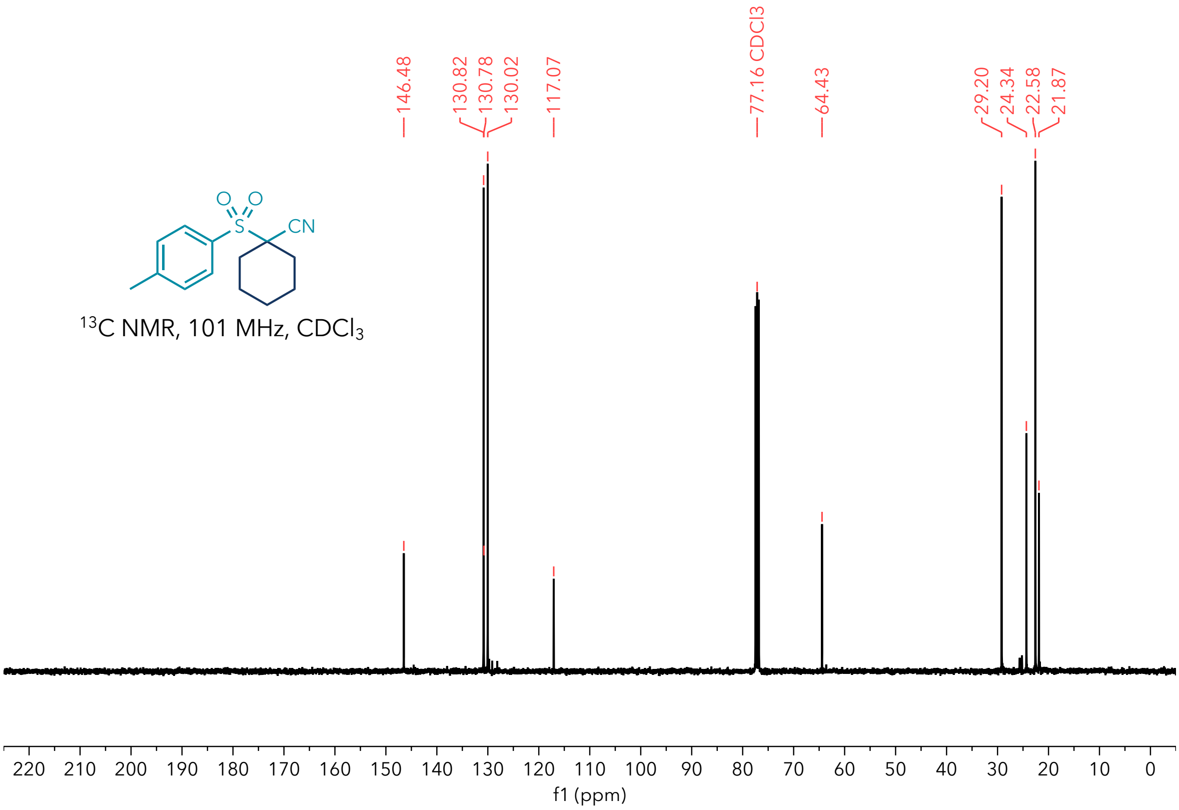
CN
13C NMR, 101 MHz, CDCl3
146.48 130.82 130.78 130.02 117.07 77.16 CDCl3 64.43 29.20 24.34 22.58 21.87
220 210 200 190 180 170 160 150 140 130 120 110 100 90 80 70 60 50 40 30 20 10 0
f1 (ppm)

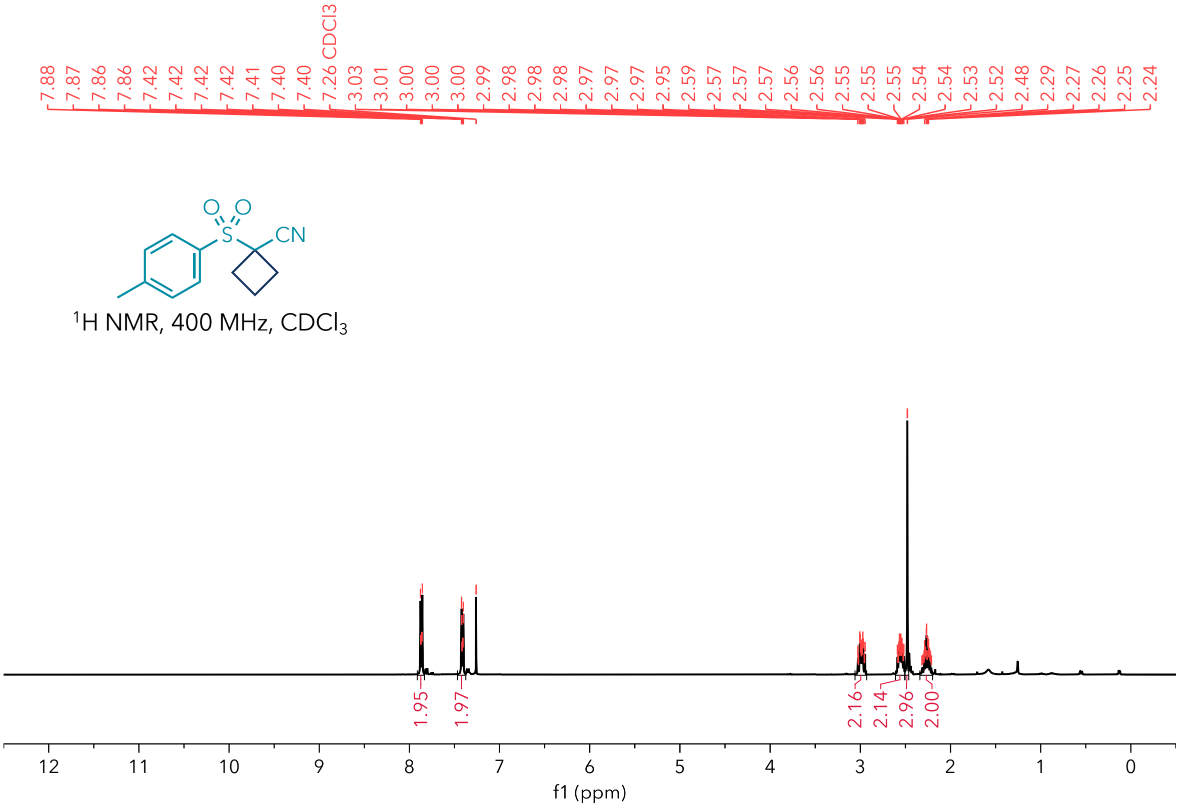
1H NMR, 400 MHz, CDCl3
f1 (ppm)

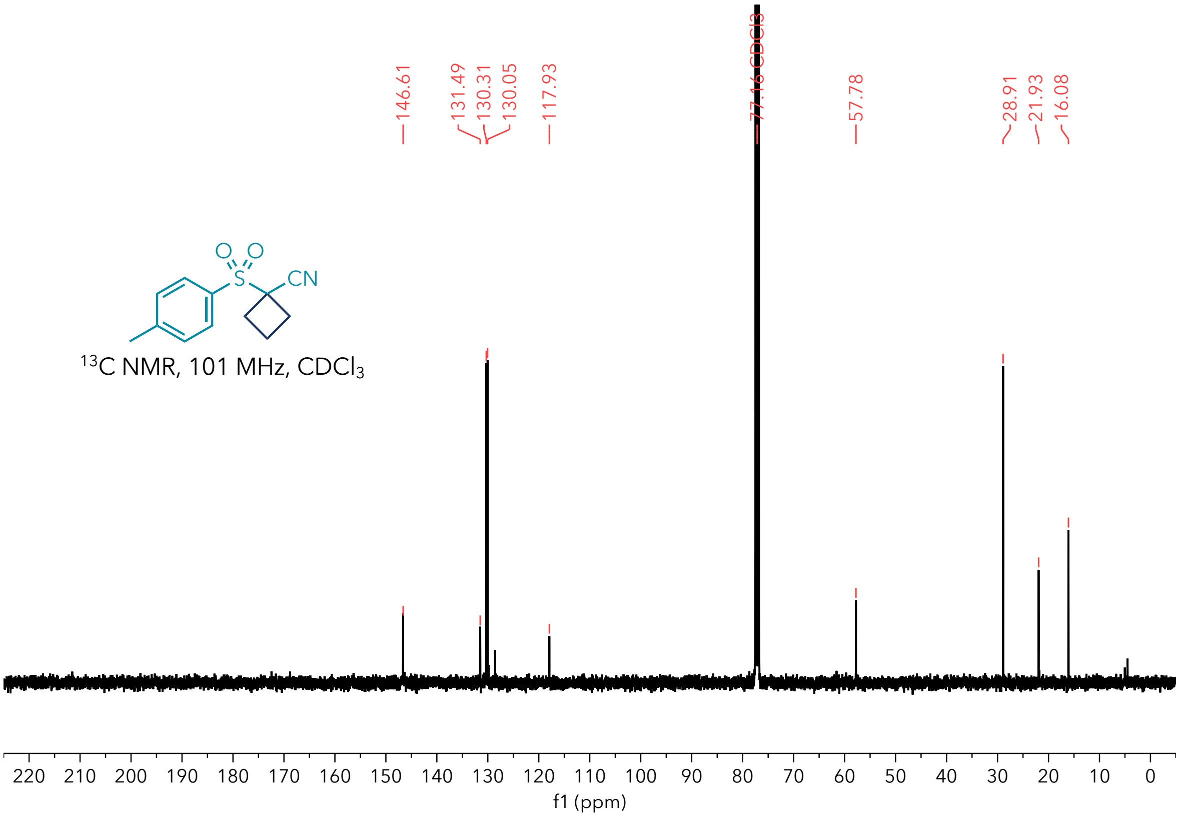
13C NMR, 101 MHz, CDCl3
f1 (ppm)

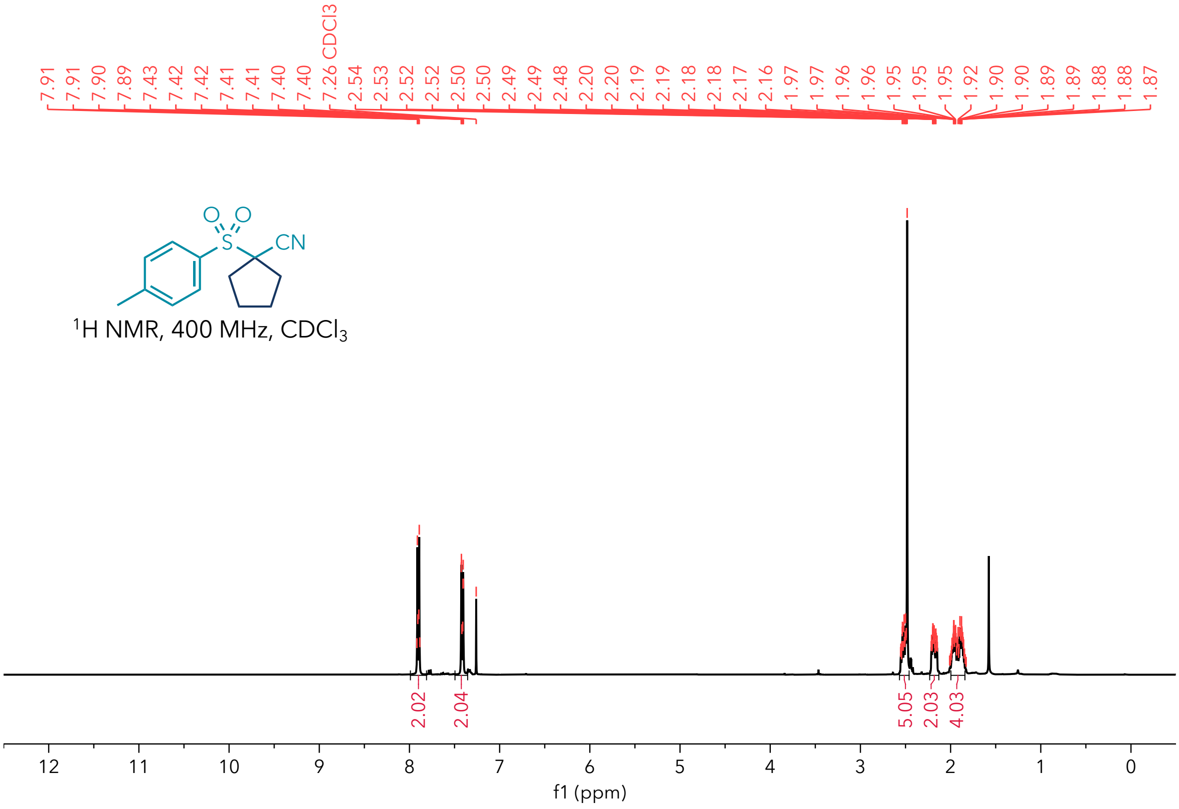

7.91
7.91
7.90
7.89
7.43
7.42
7.42
7.41
7.41
7.40
7.40
7.26 CDCl3
2.54
2.53
2.52
2.52
2.50
2.50
2.49
2.49
2.48
2.20
2.20
2.19
2.19
2.18
2.18
2.17
2.16
1.97
1.97
1.96
1.96
1.95
1.95
1.95
1.92
1.90
1.90
1.89
1.89
1.88
1.88
1.87
CN
^{1}H NMR, 400 MHz, $CDCl_3$
2.02
2.04
5.05
2.03
4.03
f1 (ppm)

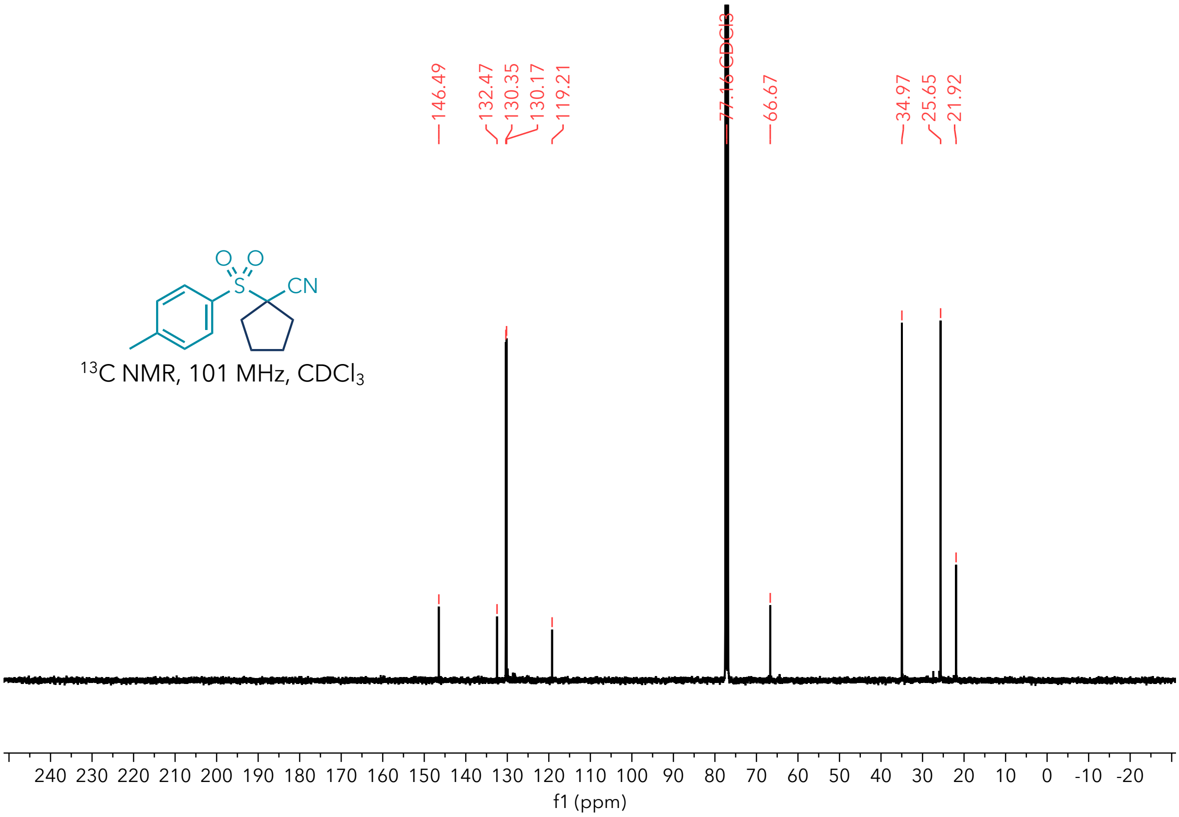

146.49
132.47
130.35
130.17
119.21
77.16 CDCl3
66.67
34.97
25.65
21.92
CN
^{13}C NMR, 101 MHz, $CDCl_3$
f1 (ppm)

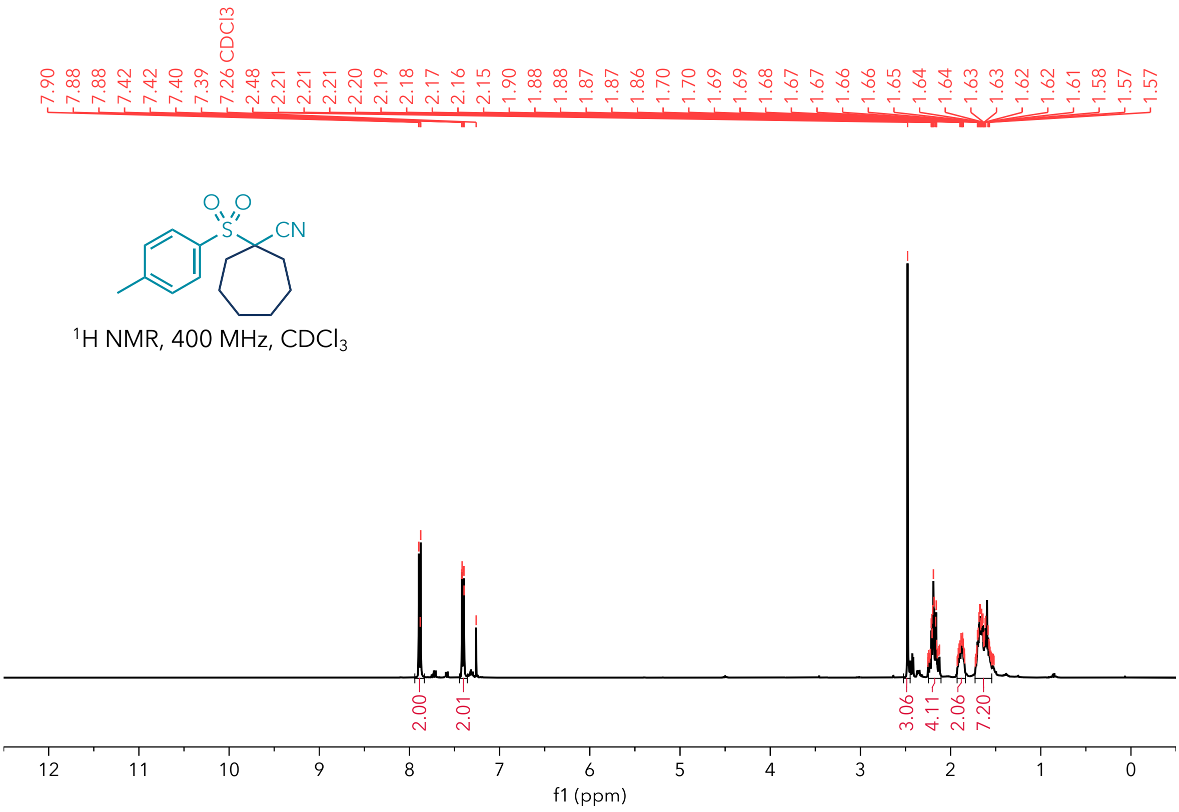
7.90
7.88
7.88
7.42
7.42
7.40
7.39
7.26 CDCl3
2.48
2.21
2.21
2.21
2.20
2.19
2.18
2.17
2.16
2.15
1.90
1.88
1.88
1.87
1.87
1.86
1.70
1.70
1.69
1.69
1.68
1.67
1.67
1.66
1.66
1.65
1.64
1.64
1.63
1.63
1.62
1.62
1.61
1.58
1.57
1.57
O
O
S
CN
^{1}H NMR, 400 MHz, $CDCl_3$
2.00
2.01
3.06
4.11
2.06
7.20
12
11
10
9
8
7
6
5
4
3
2
1
0
f1 (ppm)

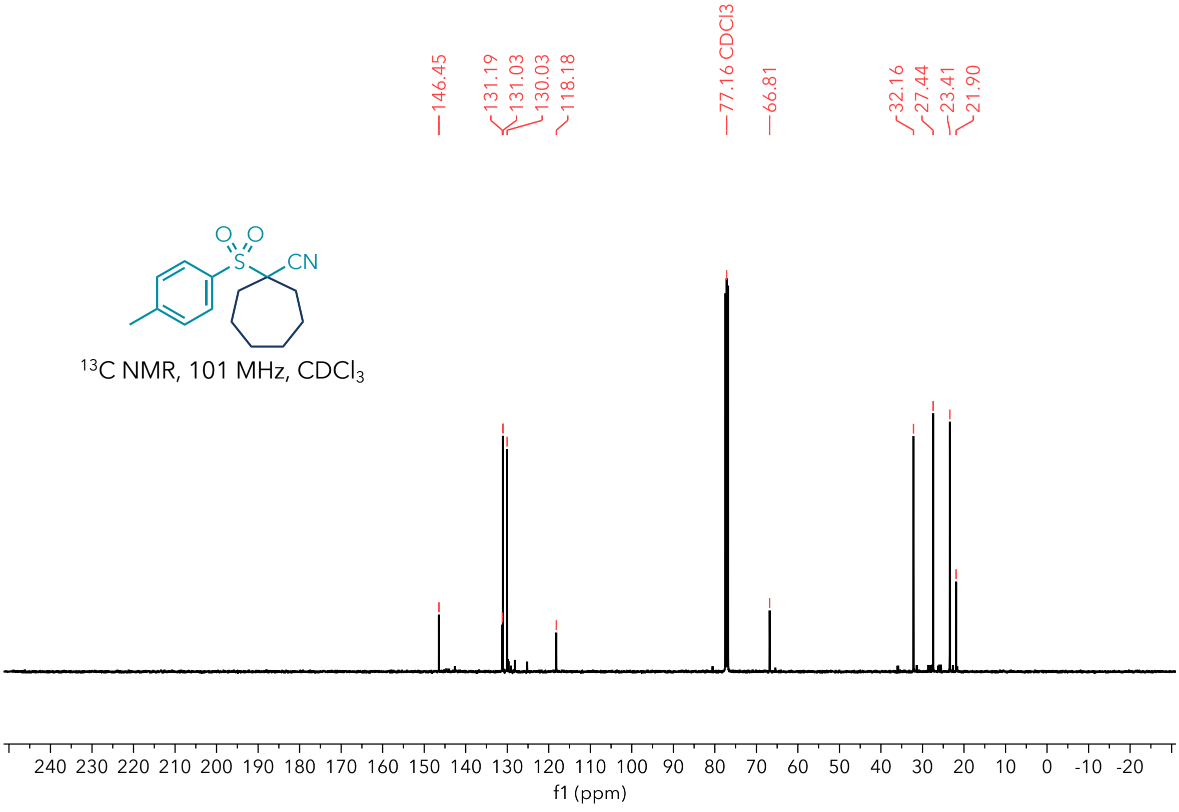
146.45
131.19
131.03
130.03
118.18
77.16 CDCl3
66.81
32.16
27.44
23.41
21.90
O
O
S
CN
^{13}C NMR, 101 MHz, $CDCl_3$
240 230 220 210 200 190 180 170 160 150 140 130 120 110 100 90 80 70 60 50 40 30 20 10 0 -10 -20
f1 (ppm)

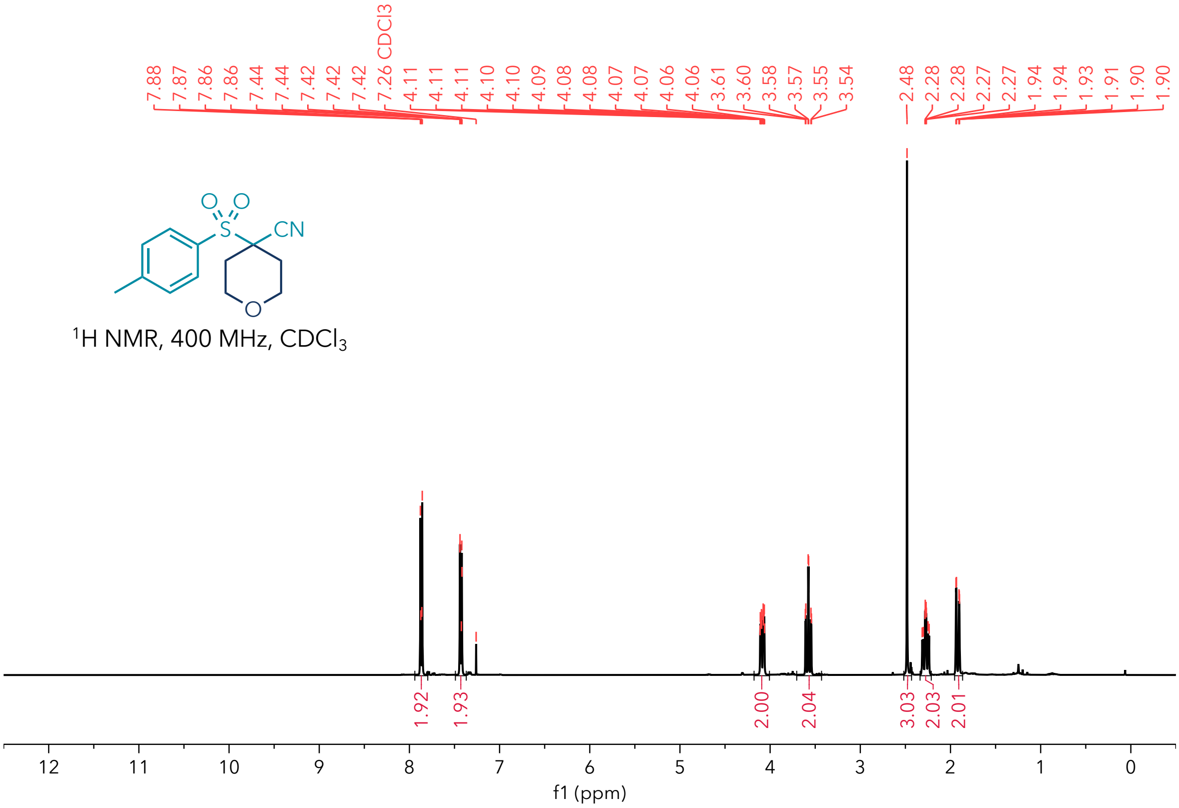

7.88 7.87 7.86 7.86 7.44 7.44 7.42 7.42 7.42 7.26 CDCl3 4.11 4.11 4.11 4.10 4.10 4.09 4.08 4.08 4.07 4.07 4.06 4.06 3.61 3.60 3.58 3.57 3.55 3.54 2.48 2.28 2.28 2.27 2.27 1.94 1.94 1.93 1.91 1.90 1.90
CN
O
S
O O
^{1}H NMR, 400 MHz, CDCl3
1.92 1.93 2.00 2.04 3.03 2.03 2.01
12 11 10 9 8 7 6 5 4 3 2 1 0
f1 (ppm)

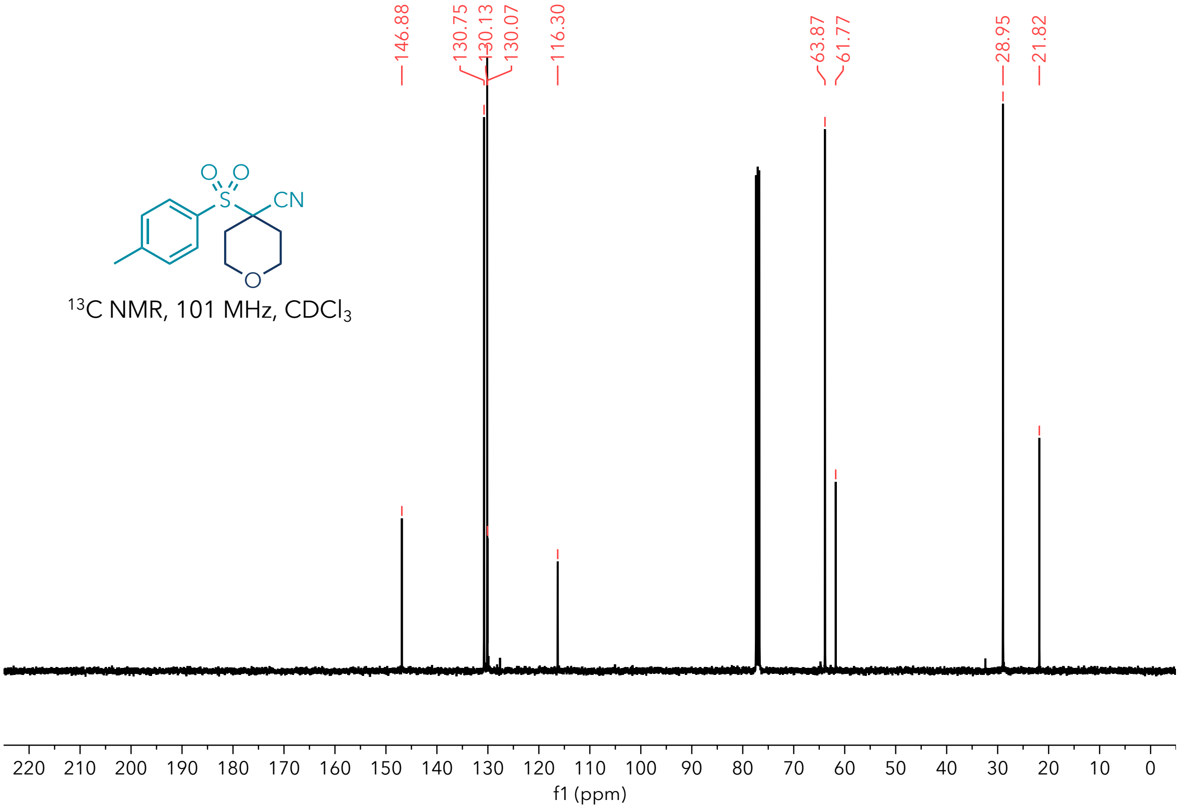

146.88 130.75 130.13 130.07 116.30 63.87 61.77 28.95 21.82
CN
O
S
O O
^{13}C NMR, 101 MHz, CDCl3
220 210 200 190 180 170 160 150 140 130 120 110 100 90 80 70 60 50 40 30 20 10 0
f1 (ppm)

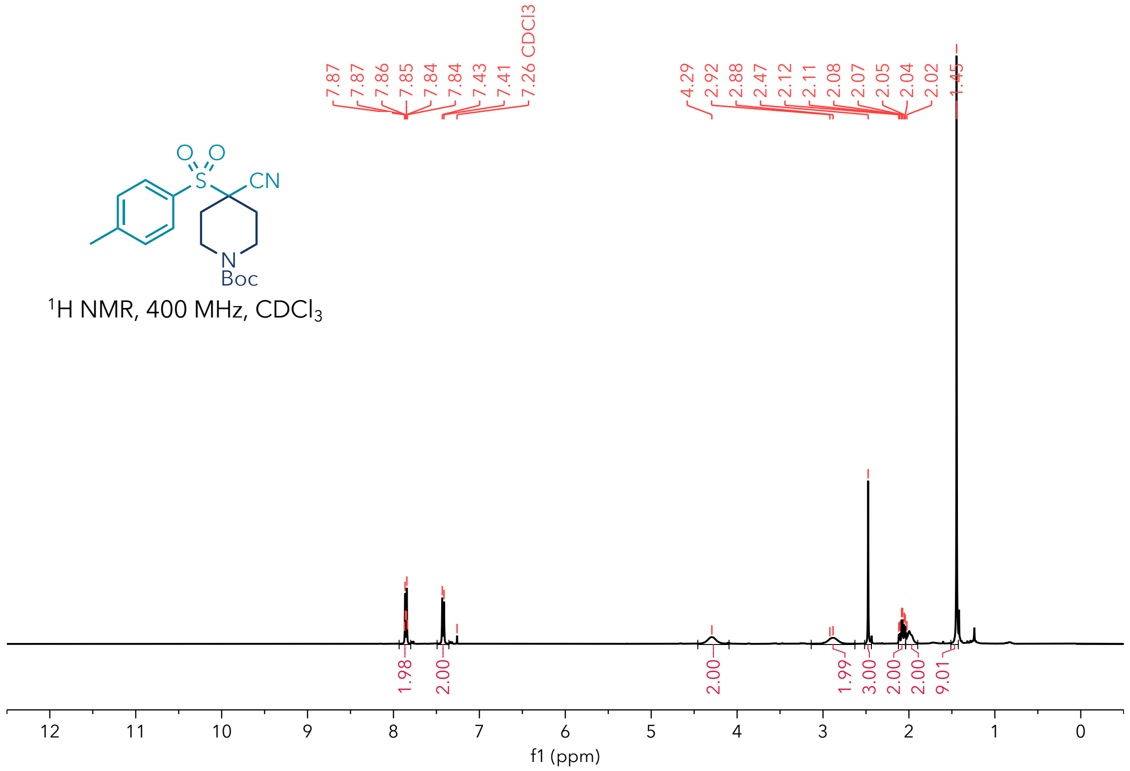

7.87
7.87
7.86
7.85
7.84
7.84
7.43
7.41
7.26 CDCl3
4.29
2.92
2.88
2.47
2.12
2.11
2.08
2.07
2.05
2.04
2.02
1.45
O
O
S
CN
N
Boc
1H NMR, 400 MHz, CDCl3
1.98
2.00
2.00
1.99
3.00
2.00
2.00
9.01
f1 (ppm)

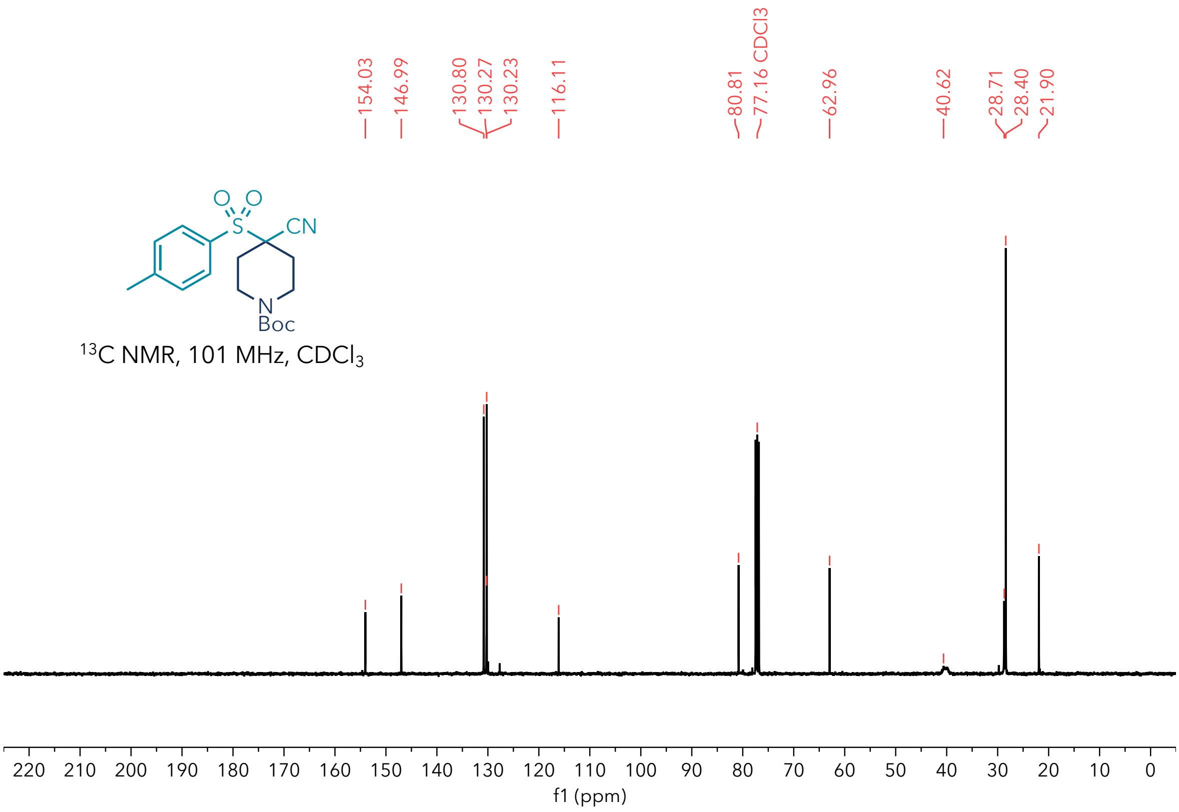

154.03
146.99
130.80
130.27
130.23
116.11
80.81
77.16 CDCl3
62.96
40.62
28.71
28.40
21.90
O
O
S
CN
N
Boc
13C NMR, 101 MHz, CDCl3
f1 (ppm)

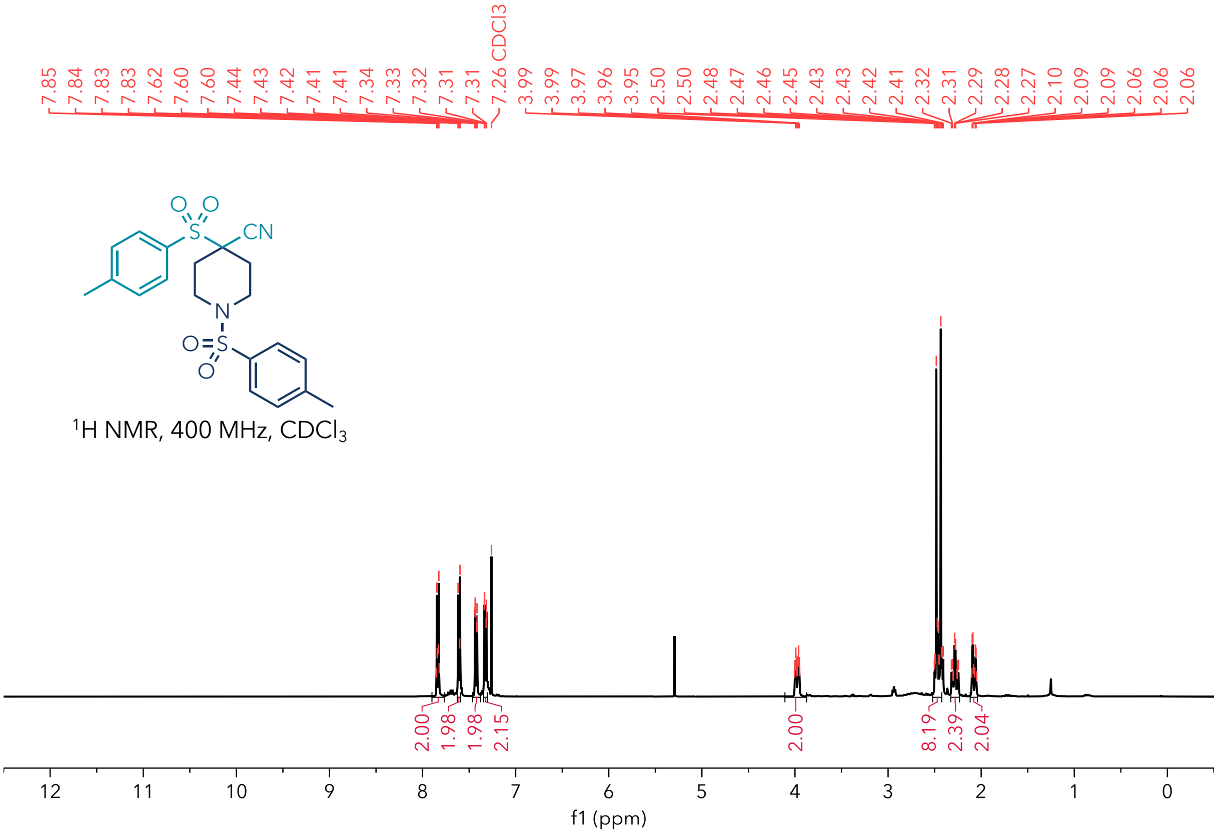
7.85 7.84 7.83 7.83 7.62 7.60 7.60 7.44 7.43 7.42 7.41 7.41 7.34 7.33 7.32 7.31 7.31 7.26 CDCl3 3.99 3.99 3.97 3.96 3.95 2.50 2.50 2.48 2.47 2.46 2.45 2.43 2.43 2.42 2.41 2.32 2.31 2.29 2.28 2.27 2.10 2.09 2.09 2.06 2.06 2.06
CN
N
^{1}H NMR, 400 MHz, $CDCl_3$
2.00 1.98 1.98 2.15 2.00 8.19 2.39 2.04
12 11 10 9 8 7 6 5 4 3 2 1 0
f1 (ppm)

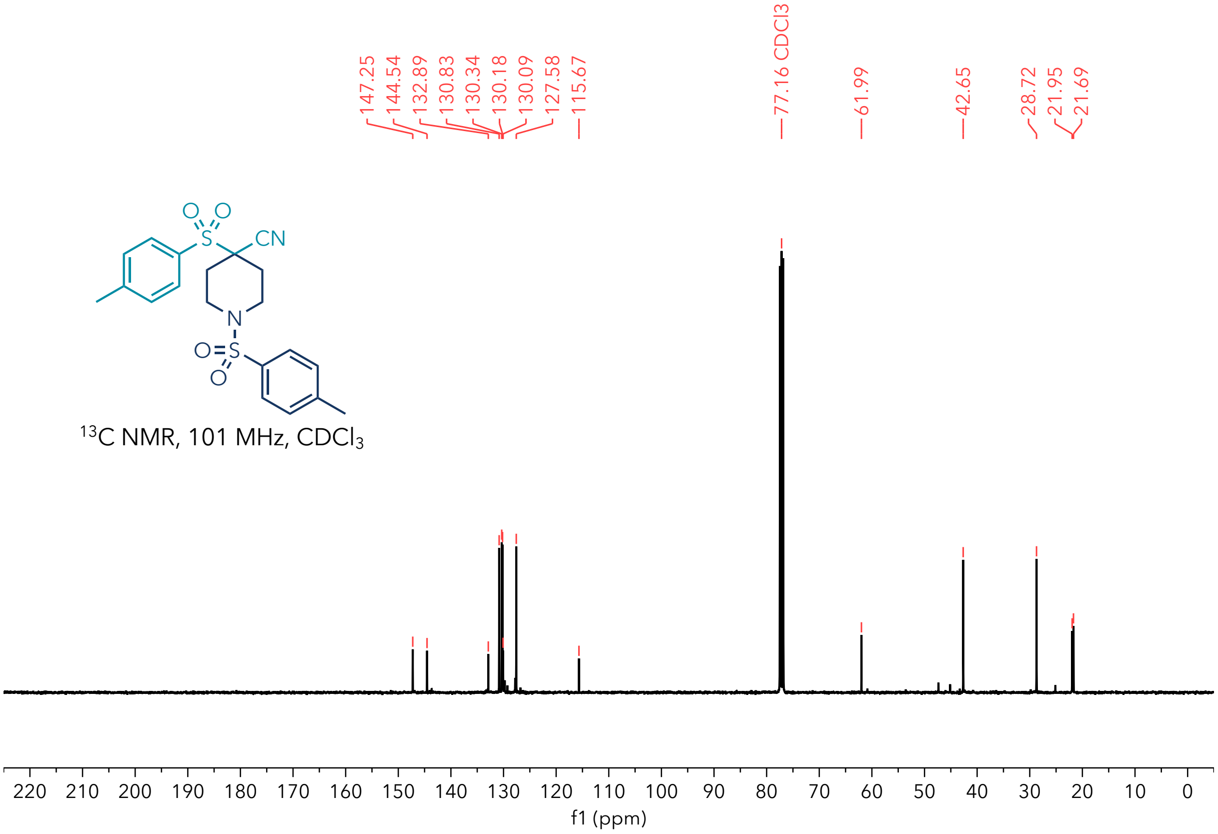
147.25 144.54 132.89 130.83 130.34 130.18 130.09 127.58 115.67 77.16 CDCl3 61.99 42.65 28.72 21.95 21.69
CN
N
^{13}C NMR, 101 MHz, $CDCl_3$
220 210 200 190 180 170 160 150 140 130 120 110 100 90 80 70 60 50 40 30 20 10 0
f1 (ppm)

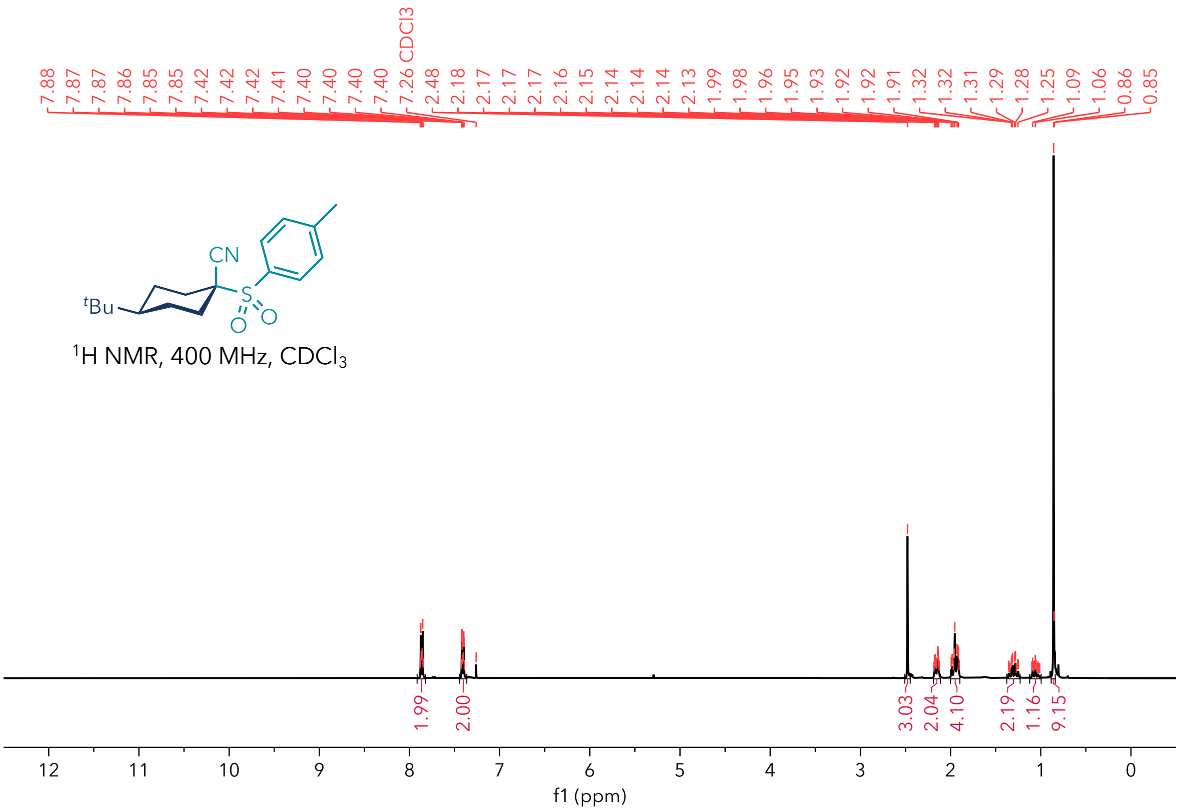
7.88
7.87
7.87
7.86
7.85
7.85
7.42
7.42
7.42
7.41
7.40
7.40
7.40
7.40
7.26 CDCl3
2.48
2.18
2.17
2.17
2.17
2.16
2.15
2.14
2.14
2.14
2.13
1.99
1.98
1.96
1.95
1.93
1.92
1.92
1.91
1.32
1.32
1.31
1.29
1.28
1.25
1.09
1.06
0.86
0.85
CN
S
O
O
tBu
1H NMR, 400 MHz, CDCl3
1.99
2.00
3.03
2.04
4.10
2.19
1.16
9.15
12
11
10
9
8
7
6
5
4
3
2
1
0
f1 (ppm)

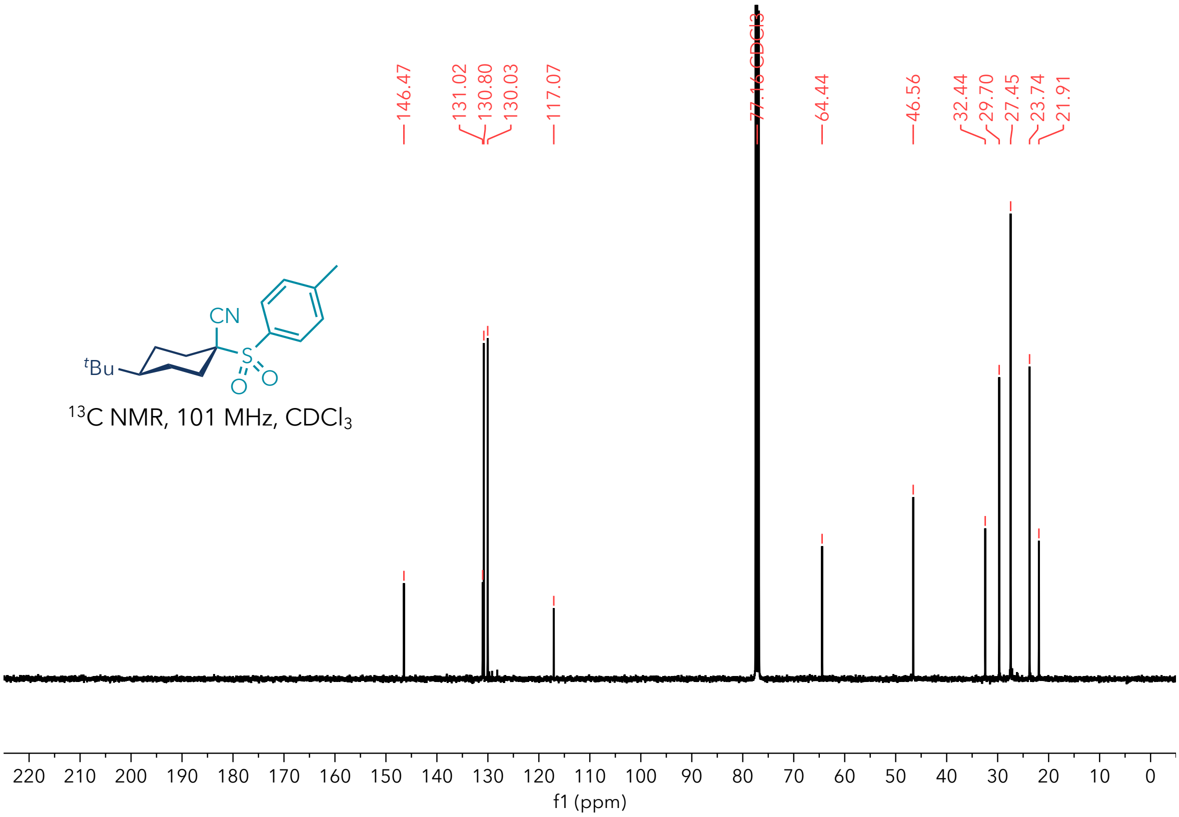
146.47
131.02
130.80
130.03
117.07
77.16 CDCl3
64.44
46.56
32.44
29.70
27.45
23.74
21.91
CN
S
O
O
tBu
13C NMR, 101 MHz, CDCl3
220
210
200
190
180
170
160
150
140
130
120
110
100
90
80
70
60
50
40
30
20
10
0
f1 (ppm)

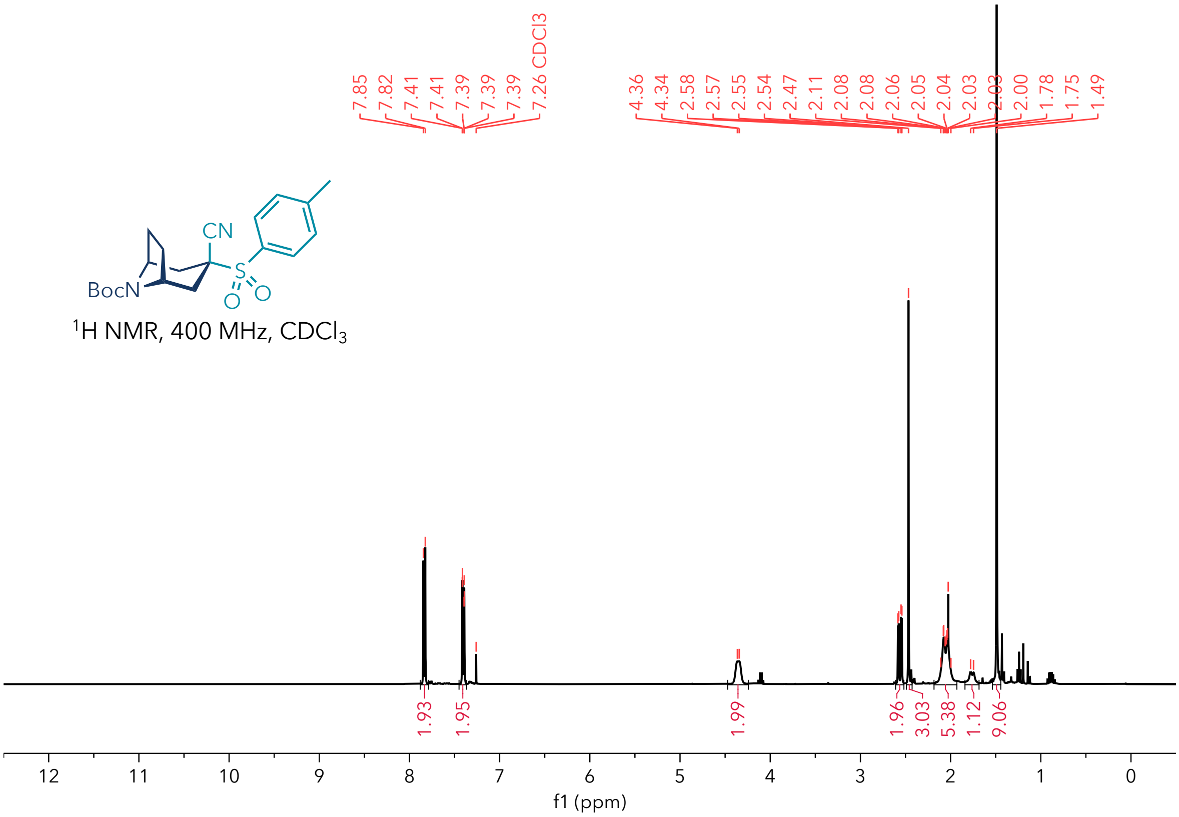
7.85
7.82
7.41
7.41
7.39
7.39
7.39
7.26 CDCl3
4.36
4.34
2.58
2.57
2.55
2.54
2.47
2.11
2.08
2.08
2.06
2.05
2.04
2.03
2.03
2.00
1.78
1.75
1.49
CN
BocN
S
O
O
^{1}H NMR, 400 MHz, $CDCl_3$
1.93
1.95
1.99
1.96
3.03
5.38
1.12
9.06
12
11
10
9
8
7
6
5
4
3
2
1
0
f1 (ppm)

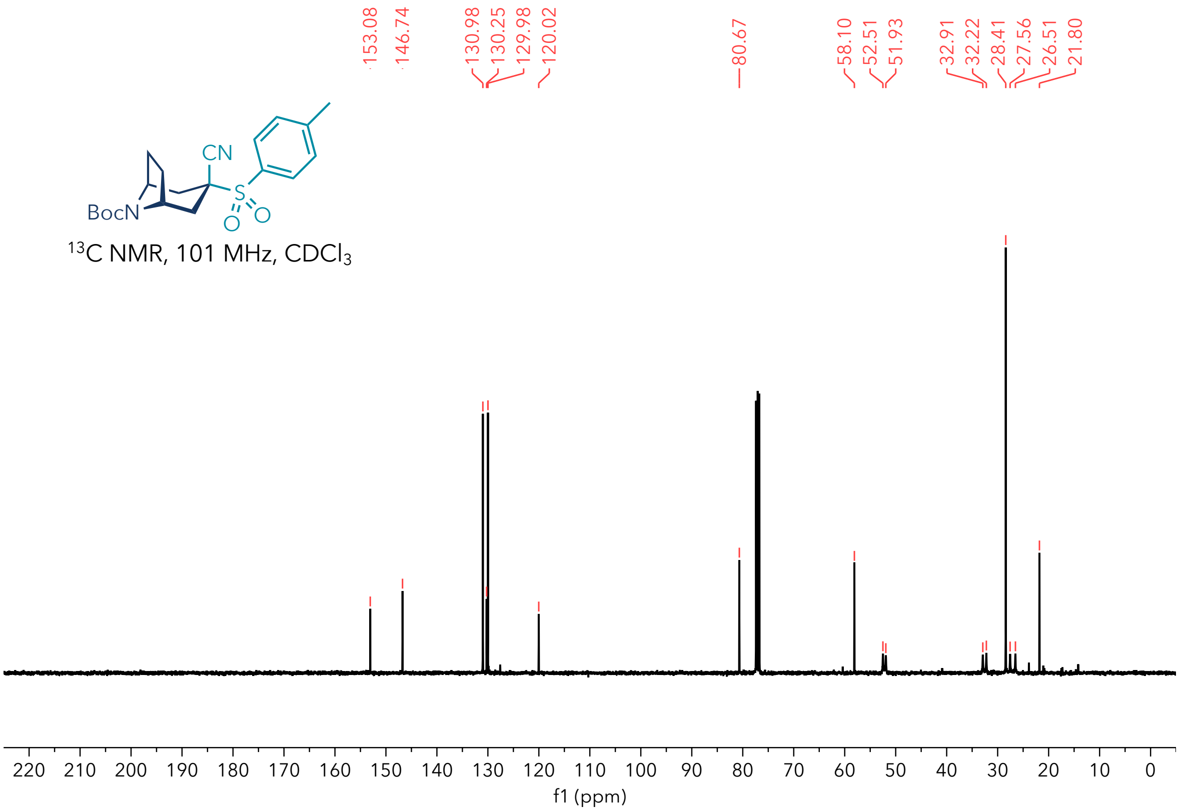
153.08
146.74
130.98
130.25
129.98
120.02
80.67
58.10
52.51
51.93
32.91
32.22
28.41
27.56
26.51
21.80
CN
BocN
S
O
O
^{13}C NMR, 101 MHz, $CDCl_3$
220 210 200 190 180 170 160 150 140 130 120 110 100 90 80 70 60 50 40 30 20 10 0
f1 (ppm)

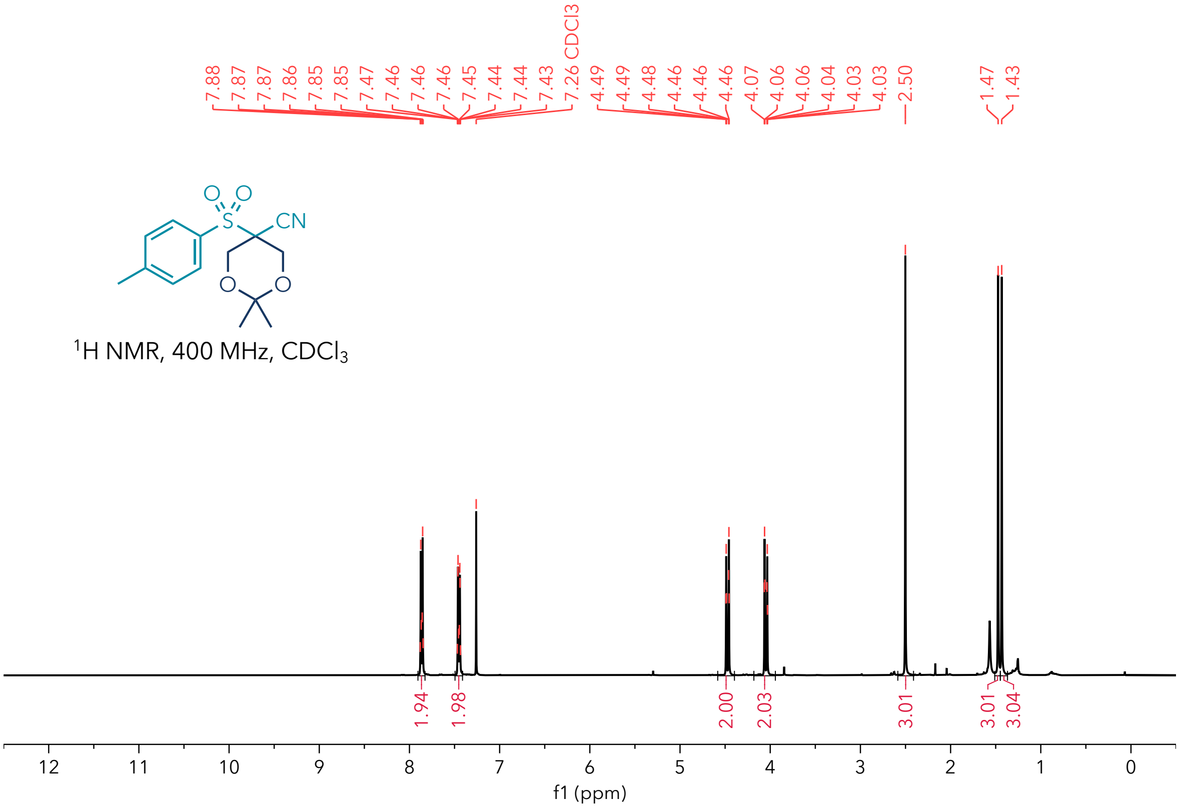
7.88
7.87
7.87
7.86
7.85
7.85
7.47
7.46
7.46
7.46
7.45
7.44
7.44
7.43
7.26 CDCl3
4.49
4.49
4.48
4.46
4.46
4.46
4.07
4.06
4.06
4.04
4.03
4.03
2.50
1.47
1.43
O O
S
CN
O O
1H NMR, 400 MHz, CDCl3
1.94
1.98
2.00
2.03
3.01
3.01
3.04
12 11 10 9 8 7 6 5 4 3 2 1 0
f1 (ppm)

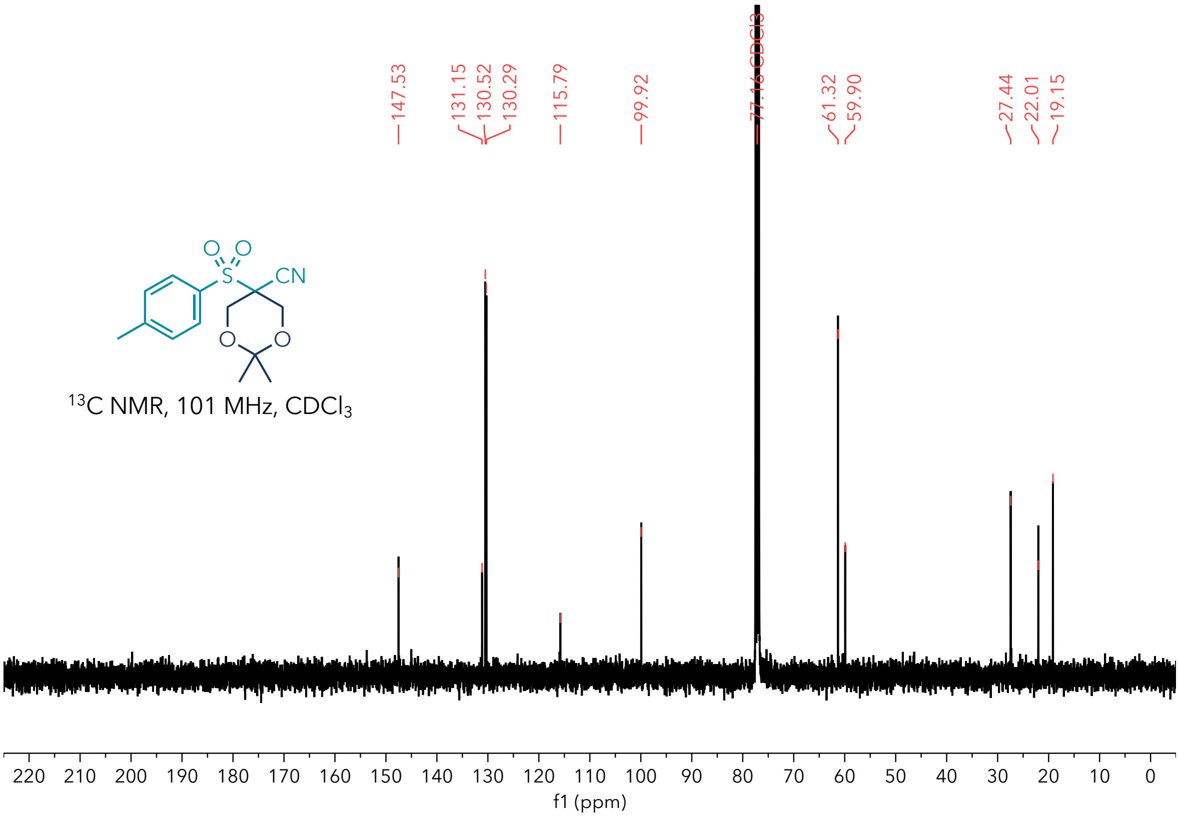
147.53
131.15
130.52
130.29
115.79
99.92
77.16 CDCl3
61.32
59.90
27.44
22.01
19.15
O O
S
CN
O O
13C NMR, 101 MHz, CDCl3
220 210 200 190 180 170 160 150 140 130 120 110 100 90 80 70 60 50 40 30 20 10 0
f1 (ppm)

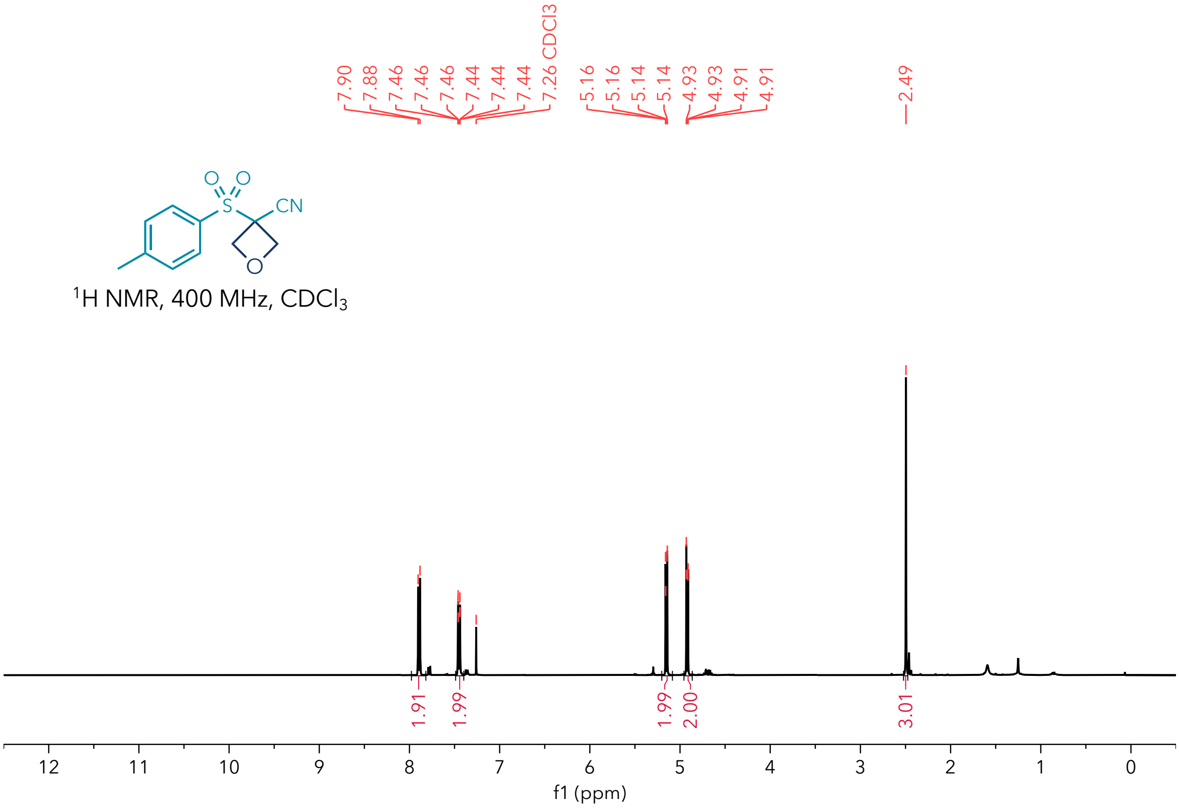

^{1}H NMR, 400 MHz, $CDCl_3$

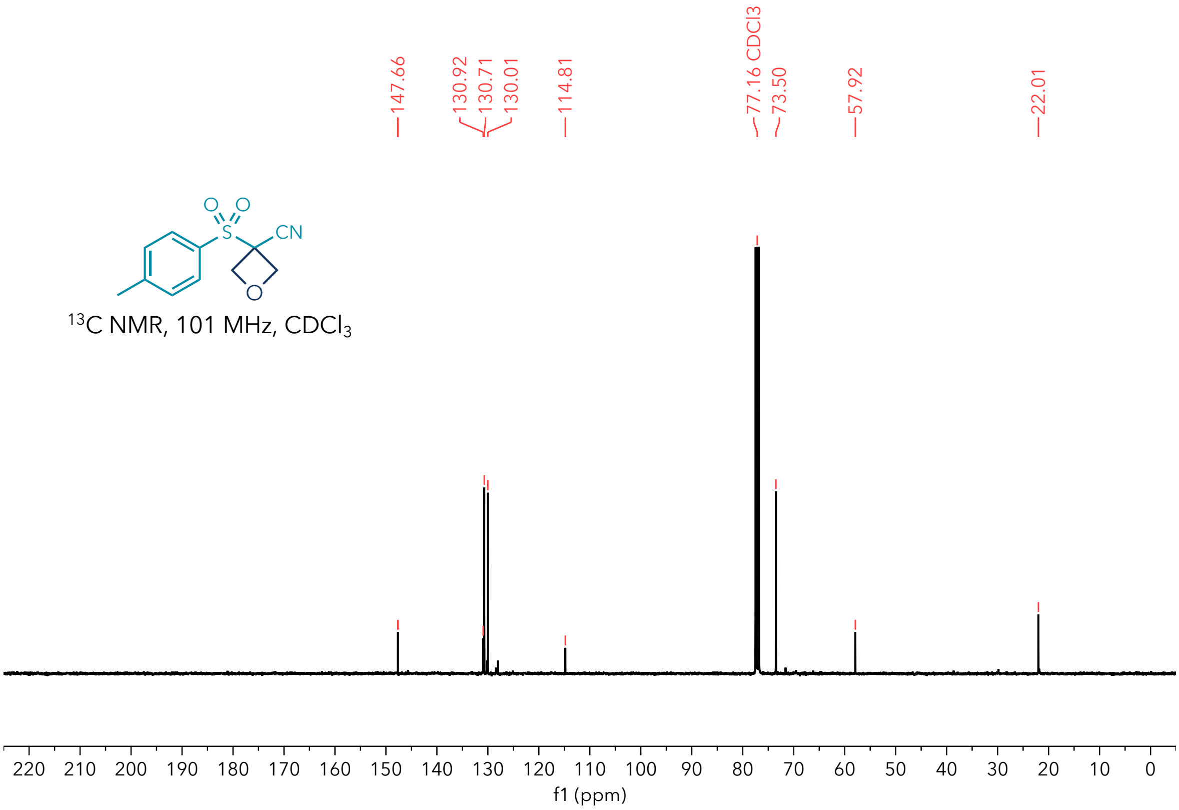

^{13}C NMR, 101 MHz, $CDCl_3$

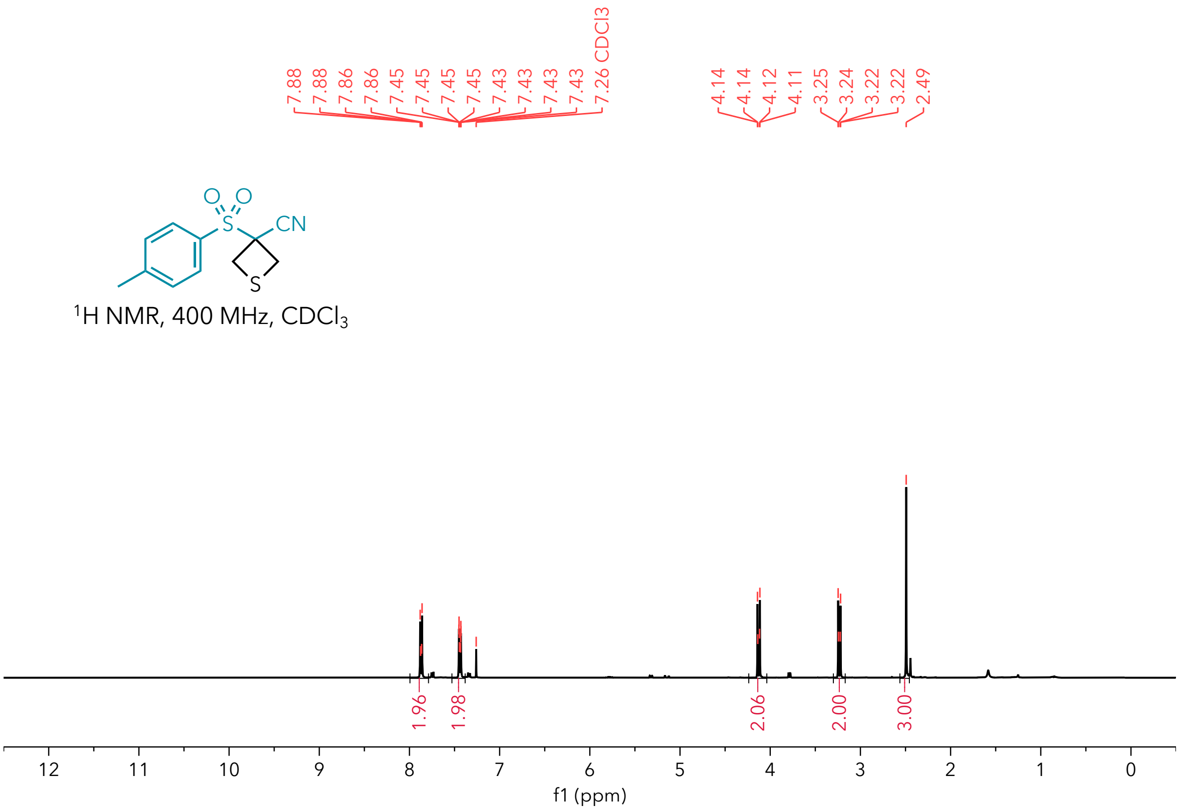

^{1}H NMR, 400 MHz, $CDCl_3$

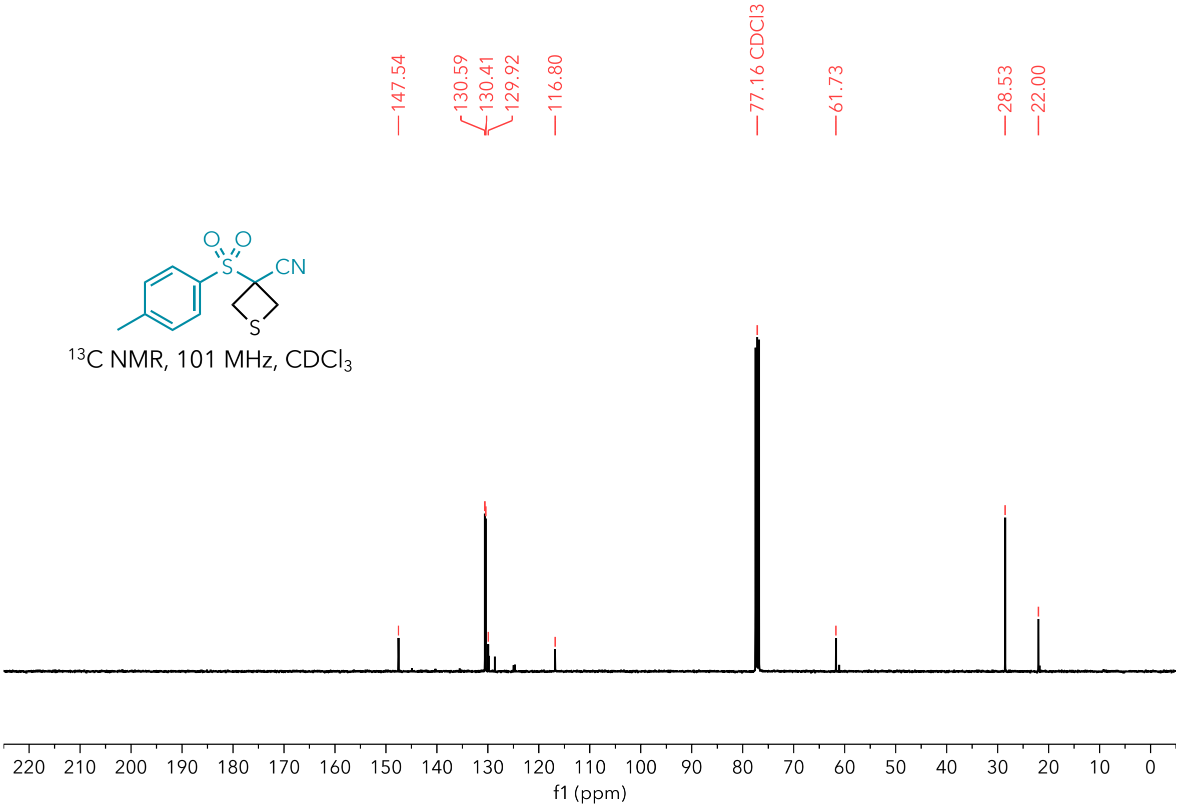

^{13}C NMR, 101 MHz, $CDCl_3$

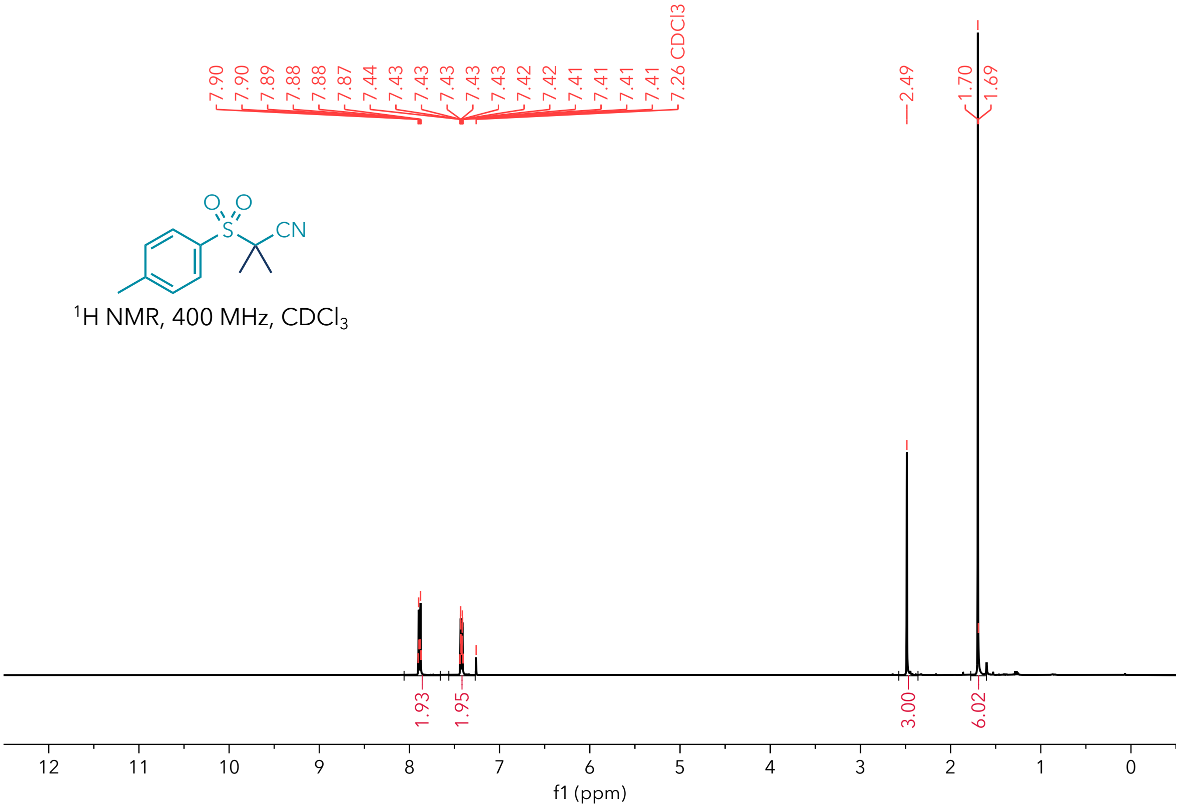

7.90
7.90
7.89
7.88
7.88
7.87
7.44
7.43
7.43
7.43
7.43
7.43
7.42
7.42
7.41
7.41
7.41
7.41
7.26 CDCl3
2.49
1.70
1.69
^{1}H NMR, 400 MHz, CDCl$_3$
1.93
1.95
3.00
6.02
f1 (ppm)

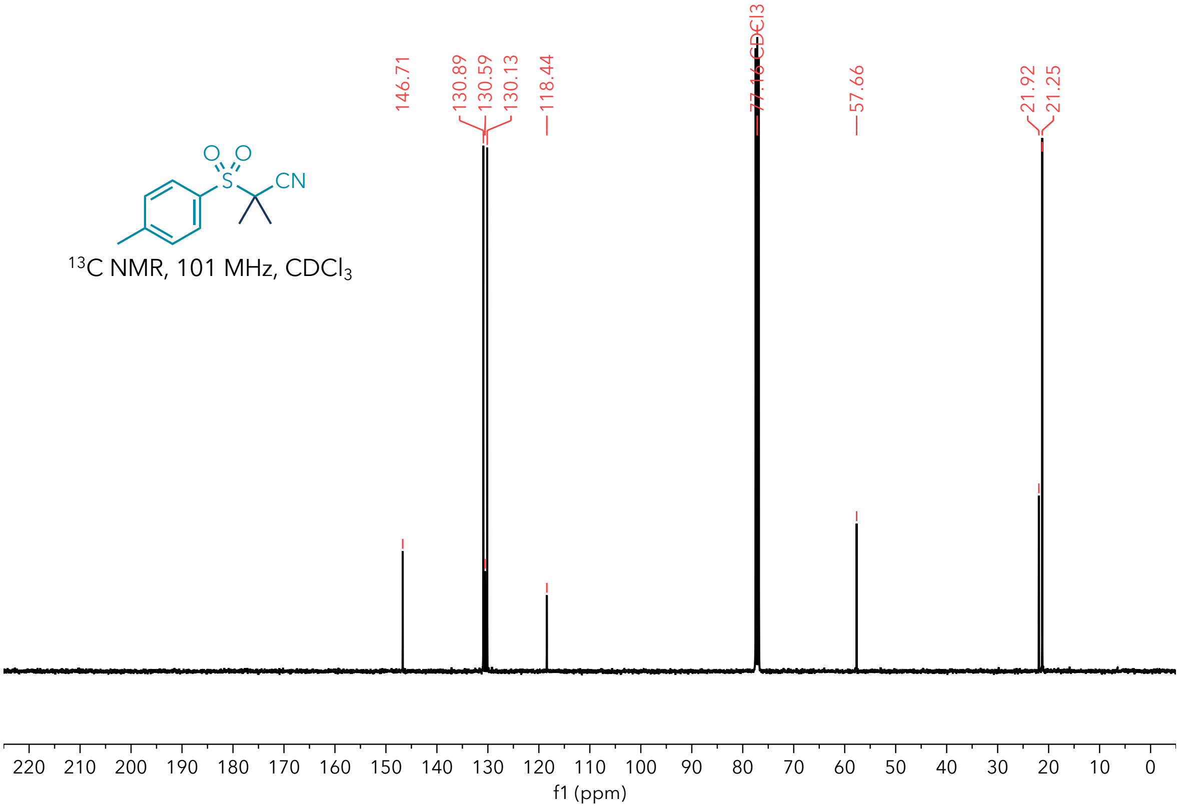

146.71
130.89
130.59
130.13
118.44
77.16 CDCl3
57.66
21.92
21.25
^{13}C NMR, 101 MHz, CDCl$_3$
f1 (ppm)

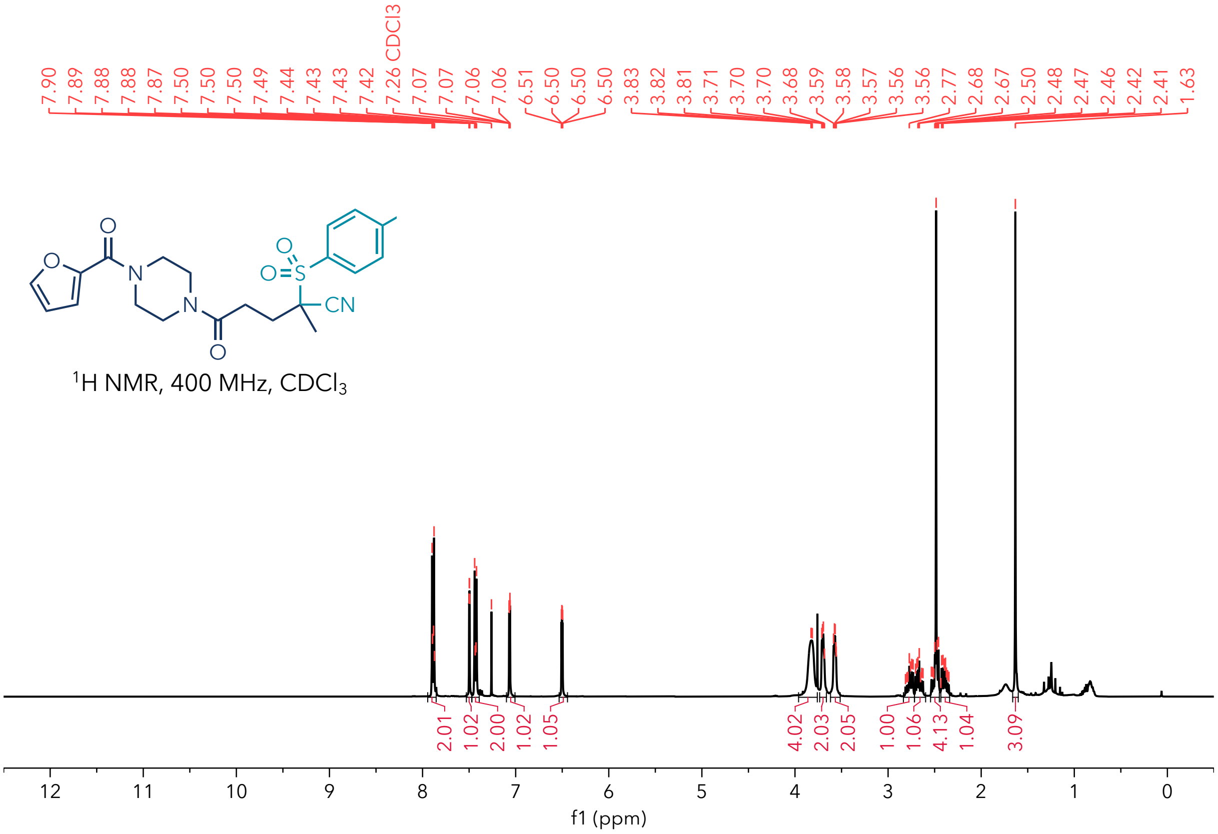

^{1}H NMR, 400 MHz, $CDCl_3$

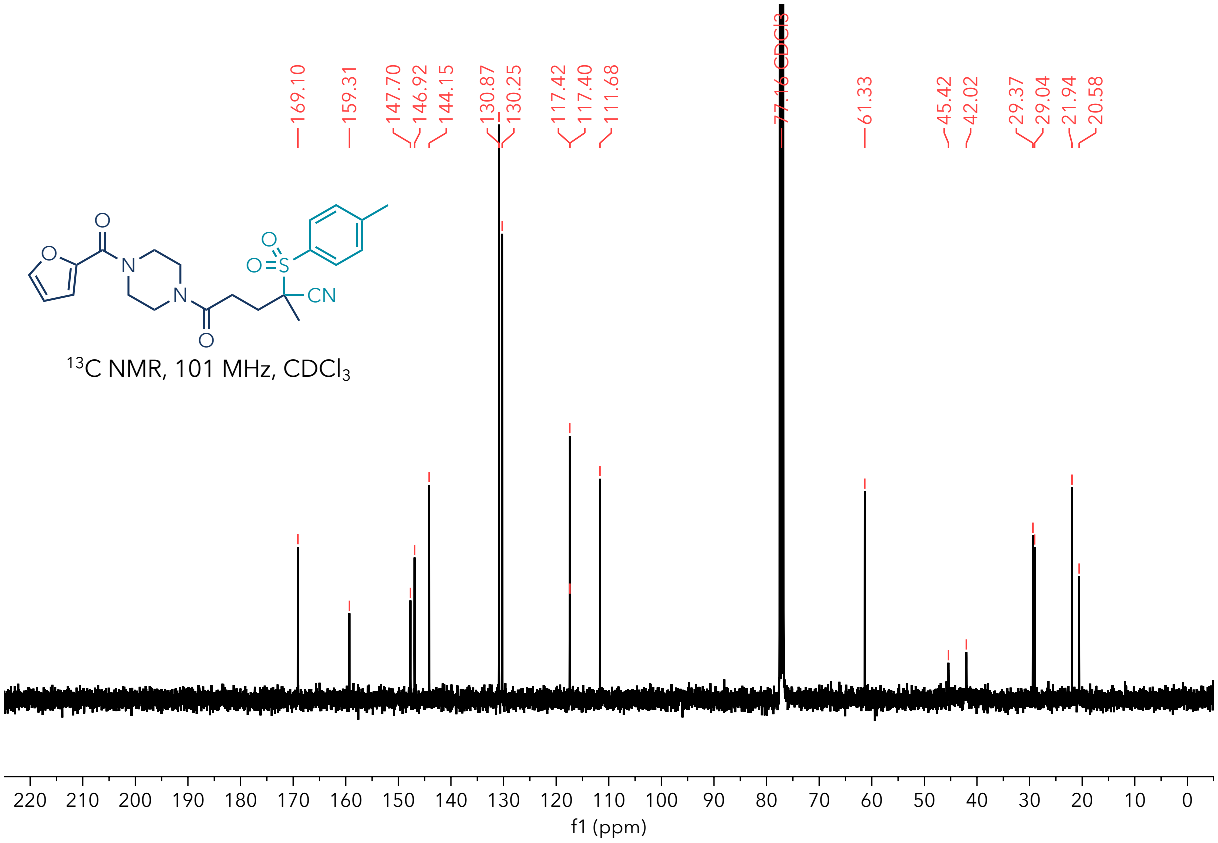

^{13}C NMR, 101 MHz, $CDCl_3$

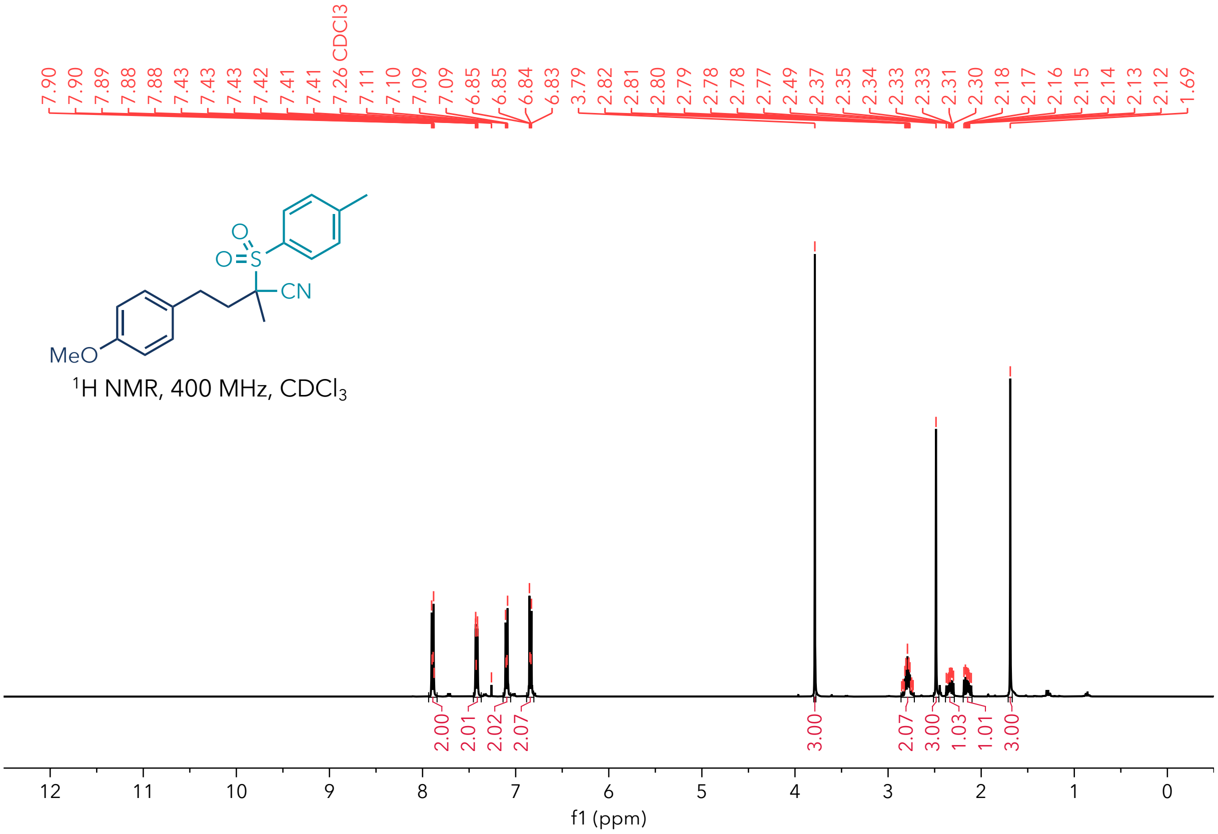

7.90
7.90
7.89
7.88
7.88
7.43
7.43
7.43
7.42
7.41
7.41
7.26 CDCl3
7.11
7.10
7.09
7.09
6.85
6.85
6.84
6.83
3.79
2.82
2.81
2.80
2.79
2.78
2.78
2.77
2.49
2.37
2.35
2.34
2.33
2.33
2.31
2.30
2.18
2.17
2.16
2.15
2.14
2.13
2.12
1.69
O
O
S
CN
MeO
^{1}H NMR, 400 MHz, $CDCl_3$
2.00
2.01
2.02
2.07
3.00
2.07
3.00
1.03
1.01
3.00
12
11
10
9
8
7
6
5
4
3
2
1
0
f1 (ppm)

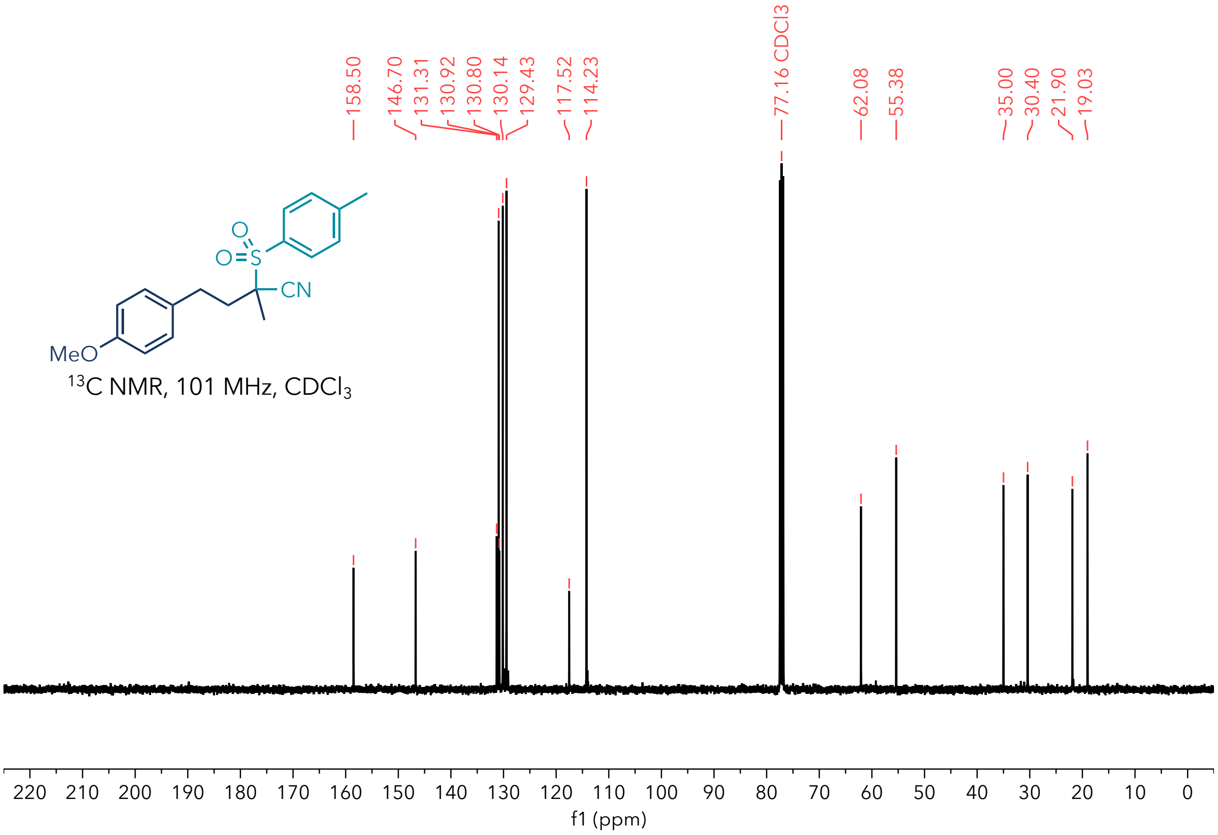

158.50
146.70
131.31
130.92
130.80
130.14
129.43
117.52
114.23
77.16 CDCl3
62.08
55.38
35.00
30.40
21.90
19.03
O
O
S
CN
MeO
^{13}C NMR, 101 MHz, $CDCl_3$
220
210
200
190
180
170
160
150
140
130
120
110
100
90
80
70
60
50
40
30
20
10
0
f1 (ppm)

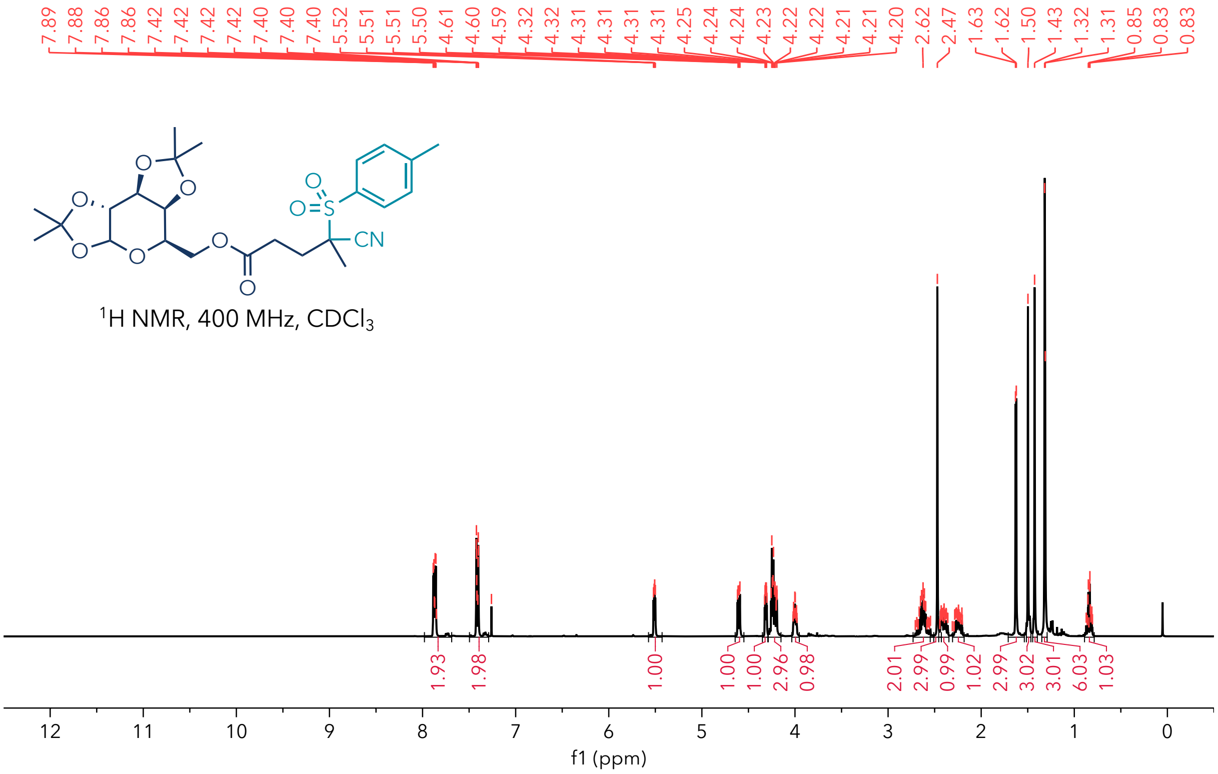
7.89 7.88 7.86 7.86 7.42 7.42 7.42 7.42 7.40 7.40 7.40 5.52 5.51 5.51 5.50 4.61 4.60 4.59 4.32 4.32 4.31 4.31 4.31 4.31 4.25 4.24 4.24 4.23 4.22 4.22 4.21 4.21 4.20 2.62 2.47 1.63 1.62 1.50 1.43 1.32 1.31 0.85 0.83 0.83
O
O=S
CN
O
^{1}H NMR, 400 MHz, $CDCl_3$
1.93 1.98 1.00 1.00 1.00 2.96 0.98 2.01 2.99 0.99 1.02 2.99 3.02 3.01 6.03 1.03
12 11 10 9 8 7 6 5 4 3 2 1 0
f1 (ppm)

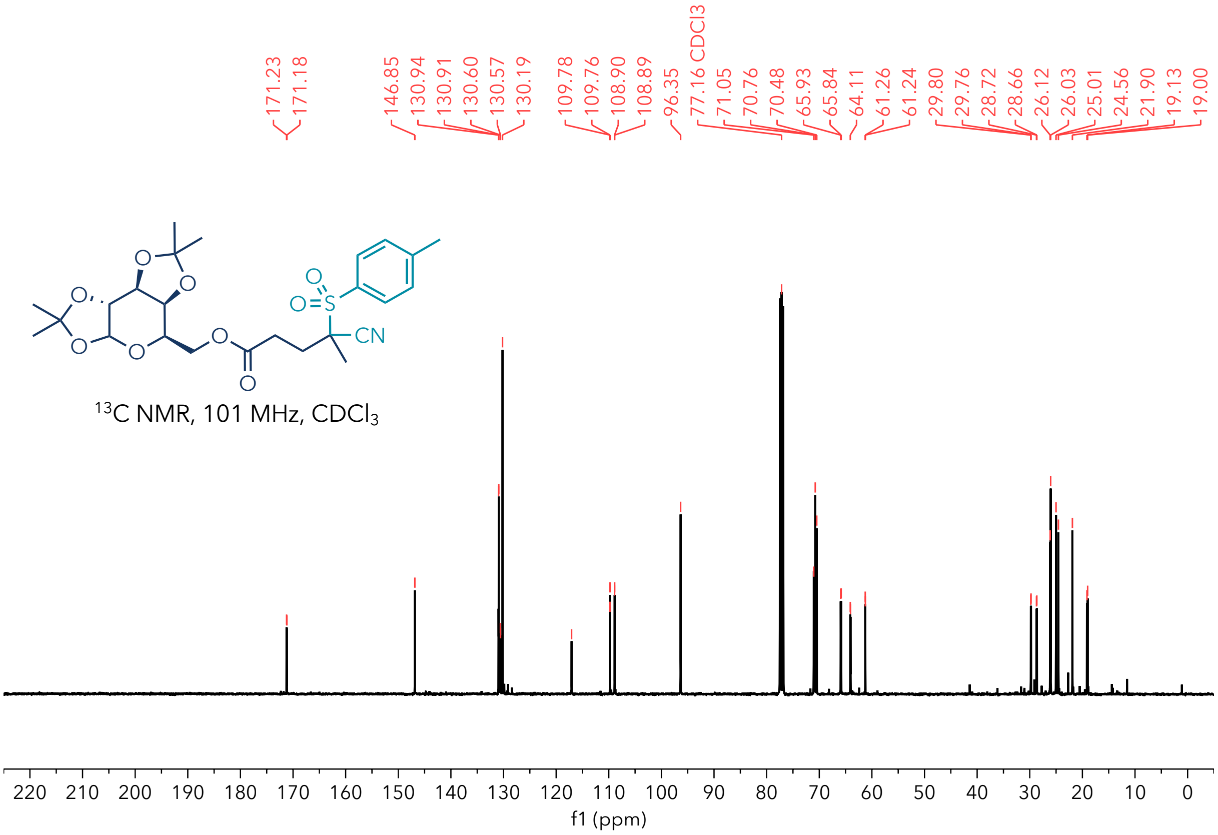
171.23 171.18 146.85 130.94 130.91 130.60 130.57 130.19 109.78 109.76 108.90 108.89 96.35 77.16 CDCl3 71.05 70.76 70.48 65.93 65.84 64.11 61.26 61.24 29.80 29.76 28.72 28.66 26.12 26.03 25.01 24.56 21.90 19.13 19.00
O
O=S
CN
O
^{13}C NMR, 101 MHz, $CDCl_3$
220 210 200 190 180 170 160 150 140 130 120 110 100 90 80 70 60 50 40 30 20 10 0
f1 (ppm)

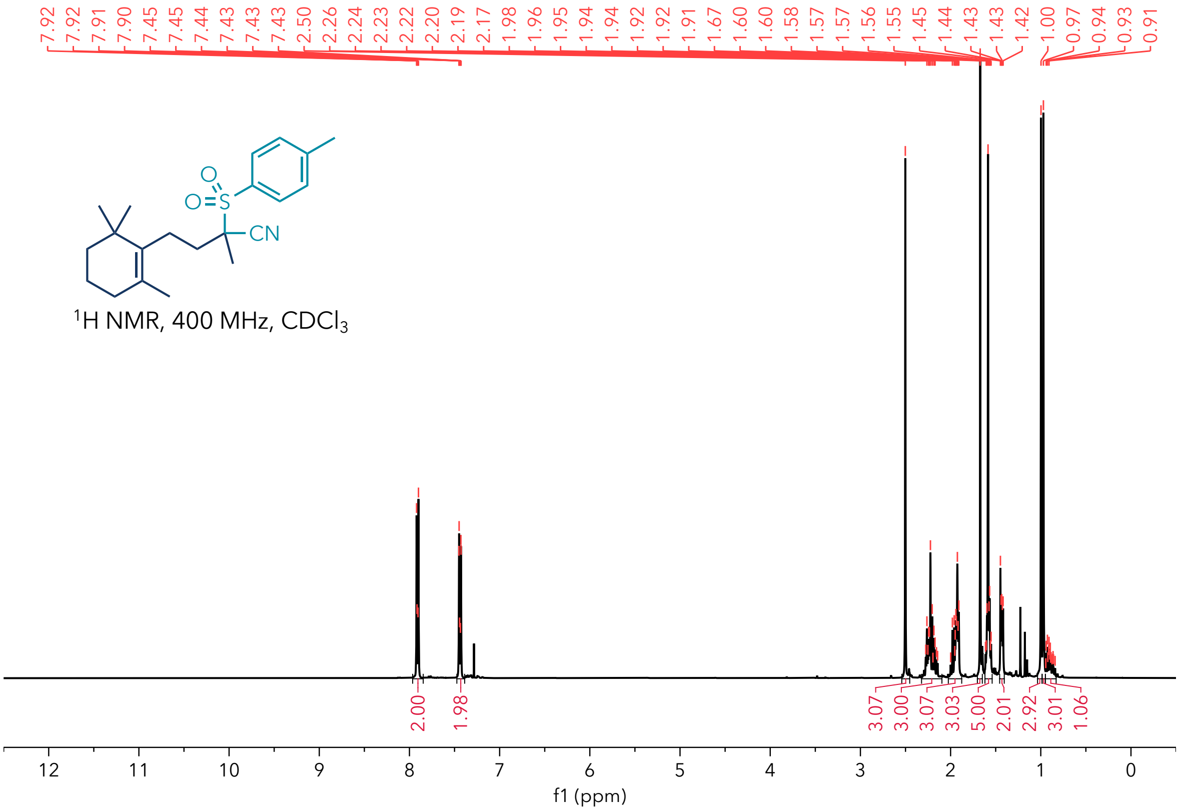
7.92 7.92 7.91 7.90 7.45 7.45 7.44 7.43 7.43 7.43 2.50 2.26 2.24 2.23 2.22 2.20 2.19 2.17 1.98 1.96 1.95 1.94 1.94 1.92 1.92 1.91 1.67 1.60 1.60 1.58 1.57 1.57 1.56 1.55 1.45 1.44 1.43 1.43 1.42 1.00 0.97 0.94 0.93 0.91
O
O=S
CN
^{1}H NMR, 400 MHz, $CDCl_3$
2.00 1.98 3.07 3.00 3.07 3.03 5.00 2.01 2.92 3.01 1.06
12 11 10 9 8 7 6 5 4 3 2 1 0
f1 (ppm)

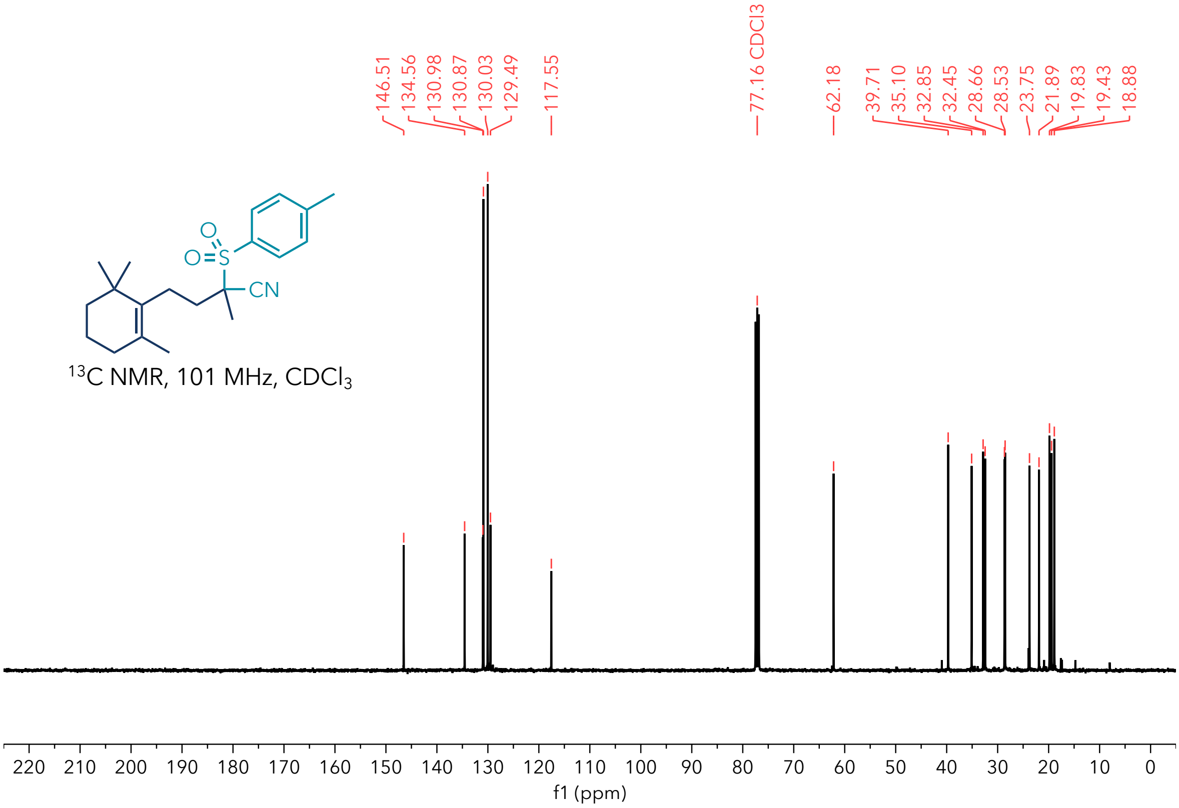
146.51 134.56 130.98 130.87 130.03 129.49 117.55 77.16 CDCl3 62.18 39.71 35.10 32.85 32.45 28.66 28.53 23.75 21.89 19.83 19.43 18.88
O
O=S
CN
^{13}C NMR, 101 MHz, $CDCl_3$
220 210 200 190 180 170 160 150 140 130 120 110 100 90 80 70 60 50 40 30 20 10 0
f1 (ppm)

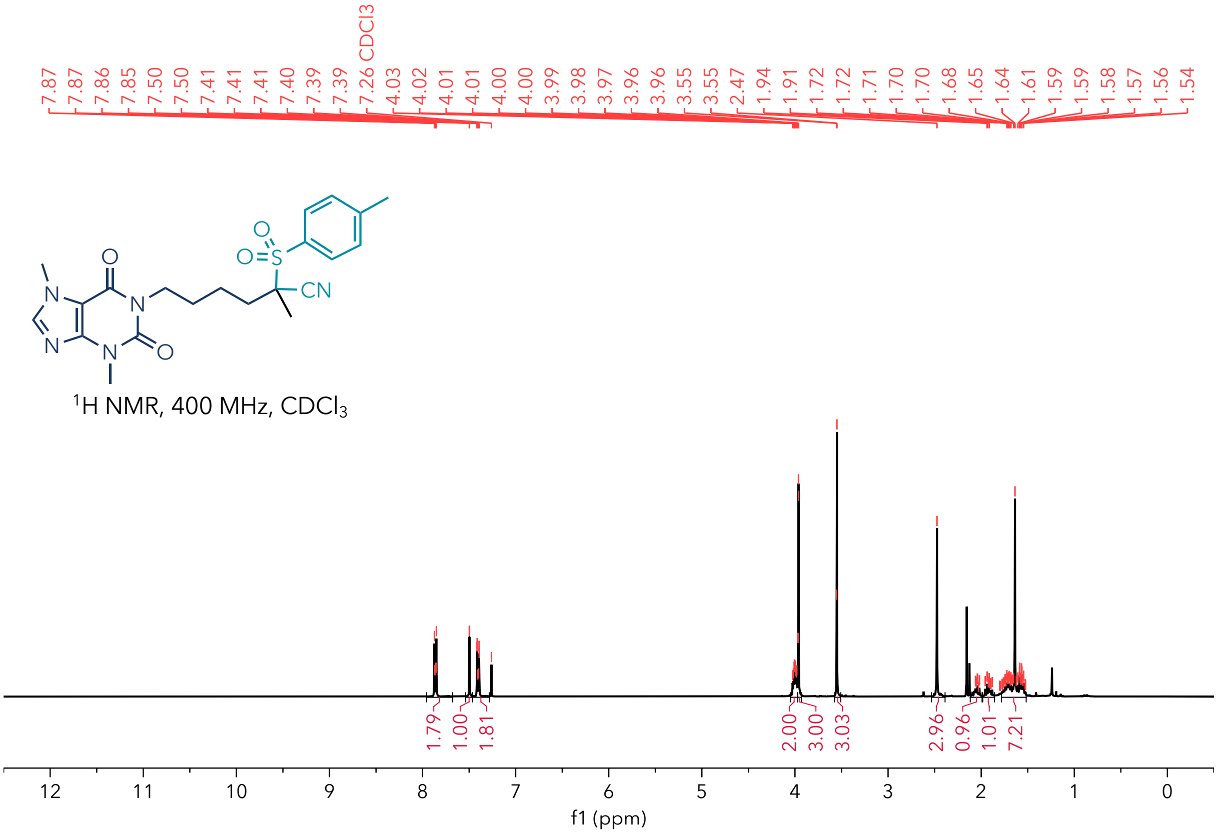

^{1}H NMR, 400 MHz, $CDCl_3$

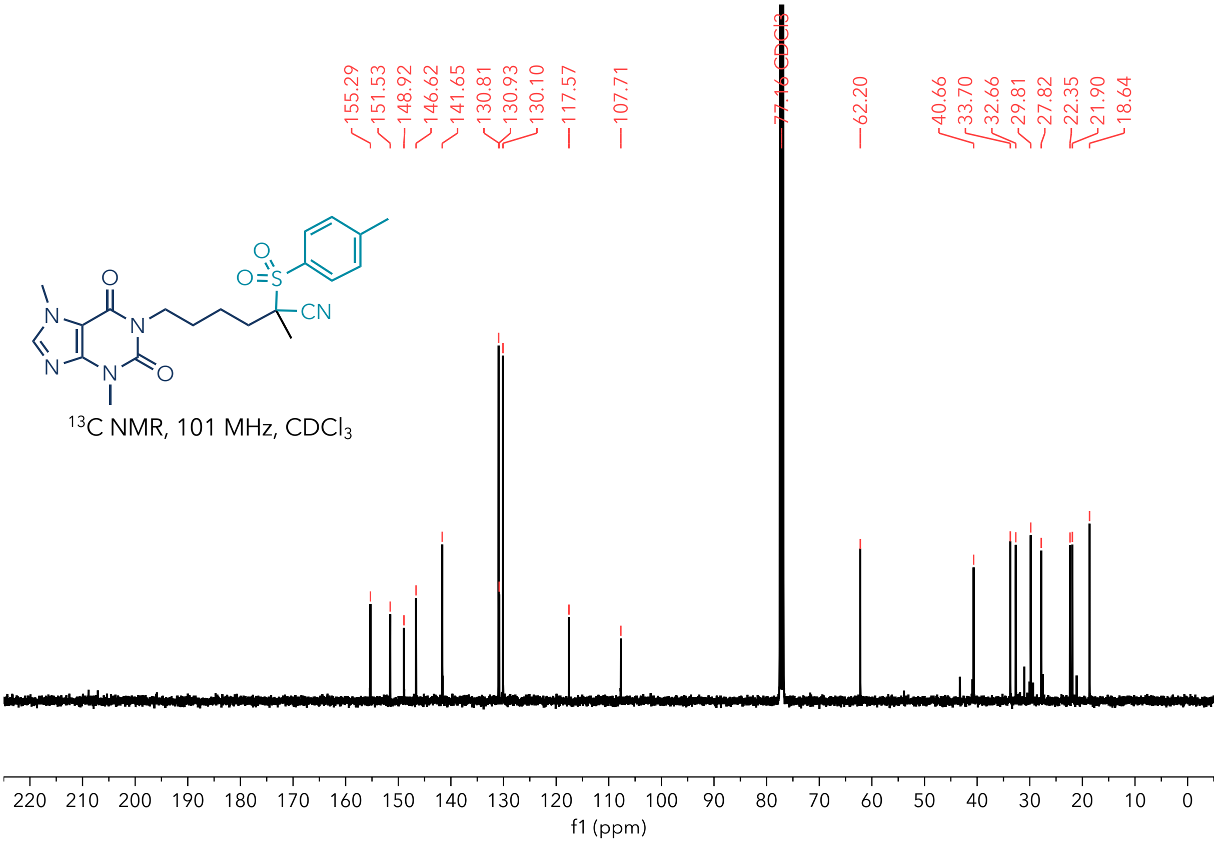

^{13}C NMR, 101 MHz, $CDCl_3$

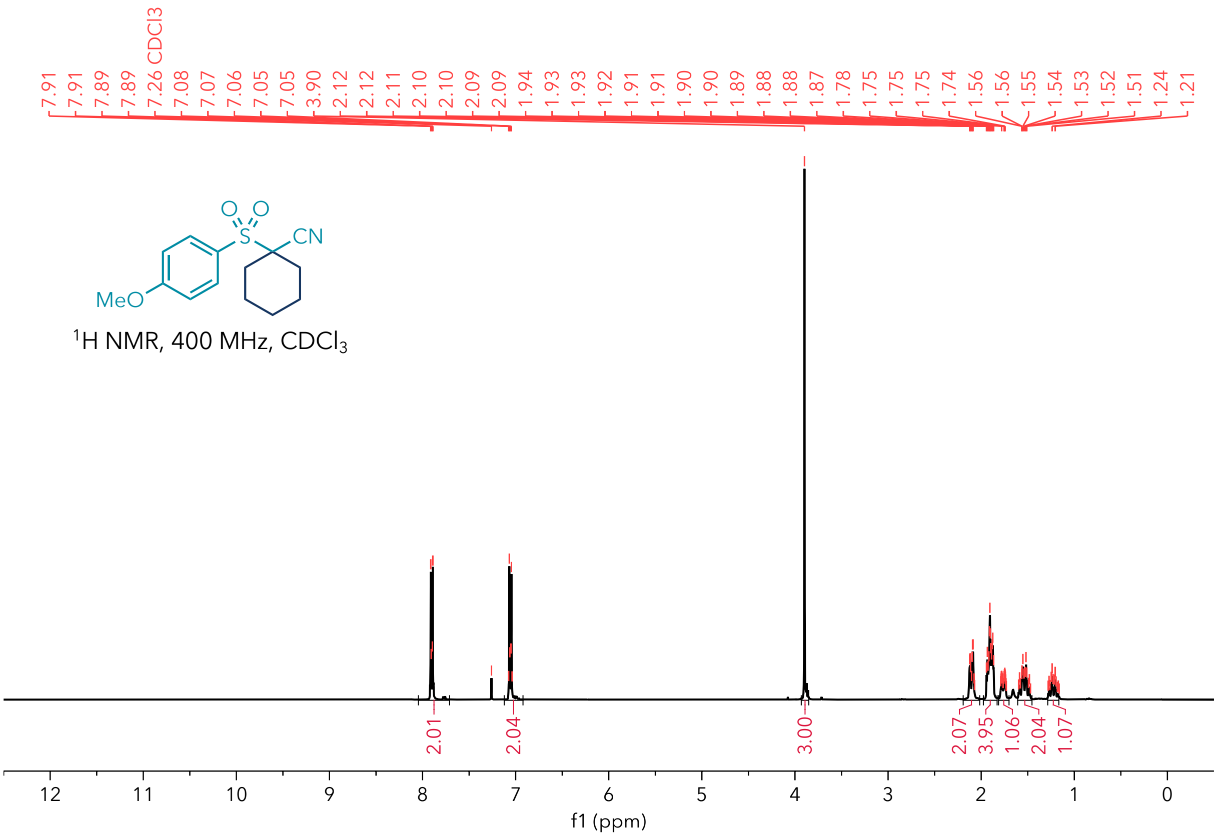

^{1}H NMR, 400 MHz, $CDCl_3$

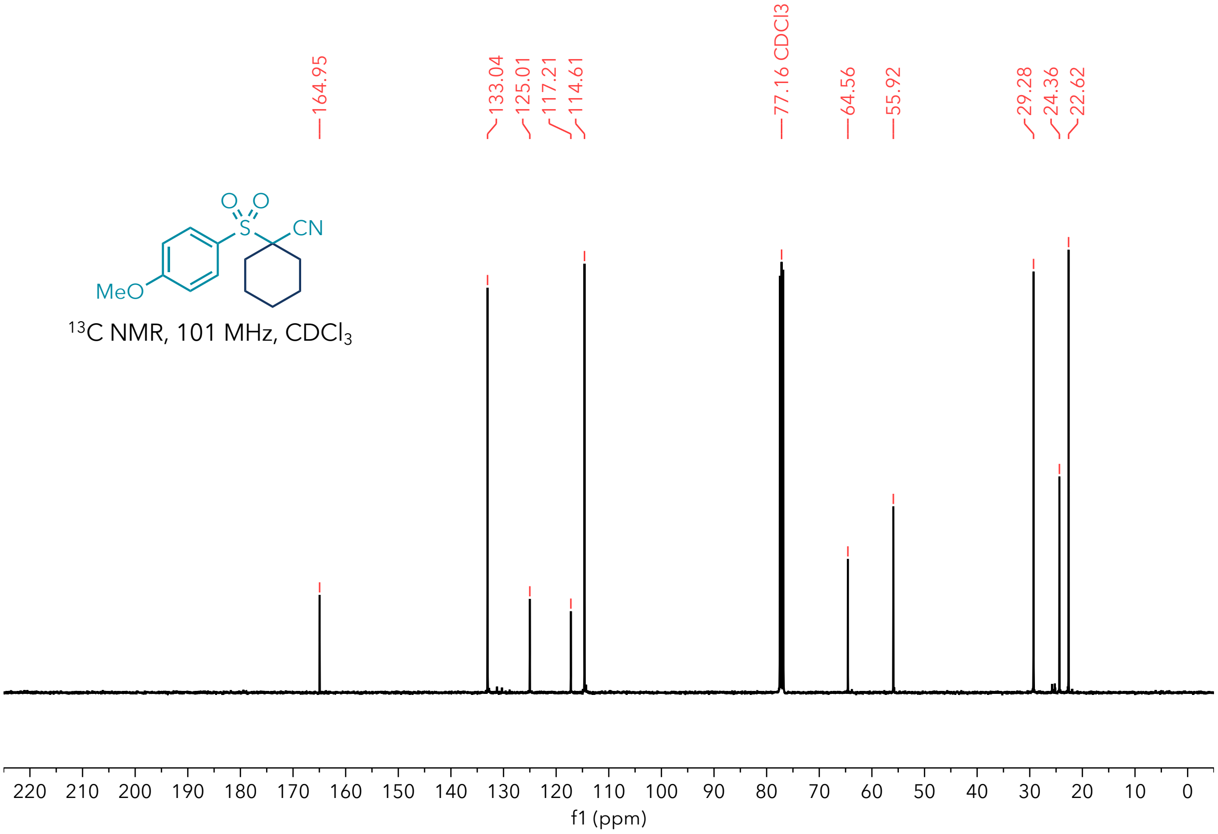

^{13}C NMR, 101 MHz, $CDCl_3$

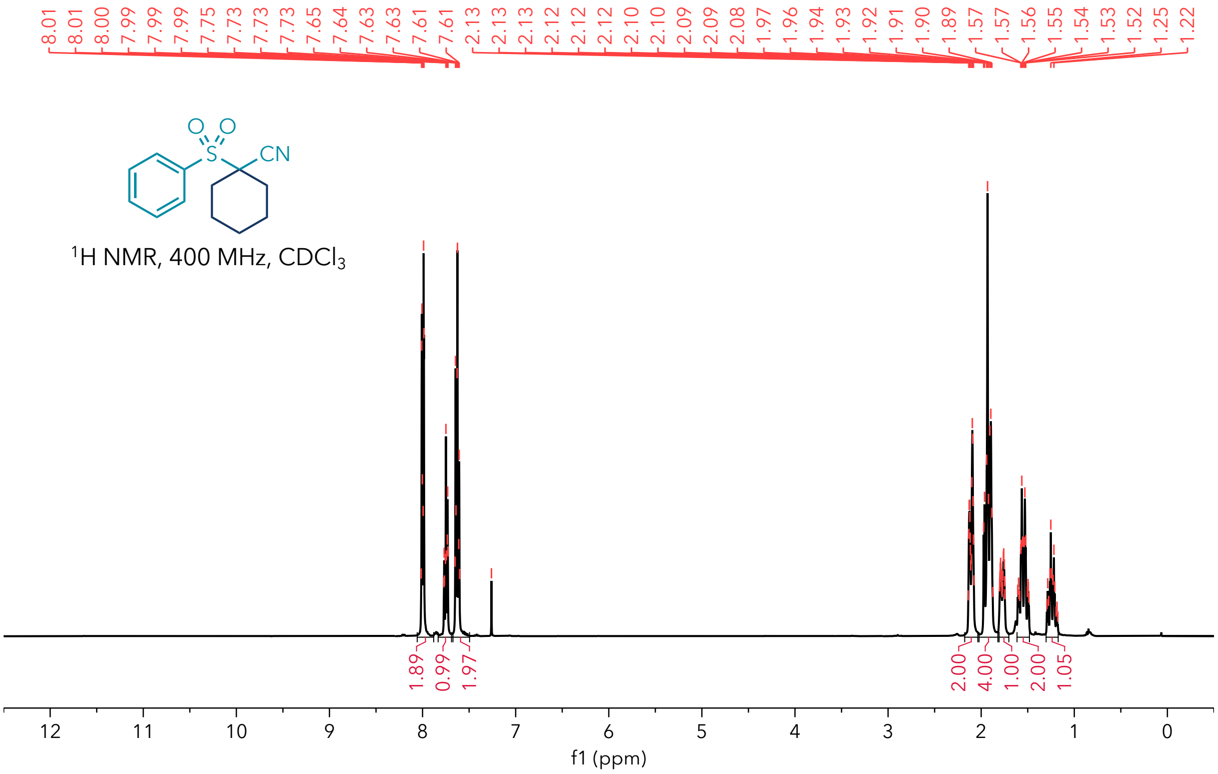
8.01
8.01
8.00
7.99
7.99
7.99
7.75
7.73
7.73
7.73
7.65
7.64
7.63
7.63
7.61
7.61
2.13
2.13
2.13
2.12
2.12
2.12
2.10
2.10
2.09
2.09
2.08
1.97
1.96
1.94
1.93
1.92
1.91
1.90
1.89
1.57
1.57
1.56
1.55
1.54
1.53
1.52
1.25
1.22
O
O
S
CN
^{1}H NMR, 400 MHz, $CDCl_3$
1.89
0.99
1.97
2.00
4.00
1.00
2.00
1.05
12
11
10
9
8
7
6
5
4
3
2
1
0
f1 (ppm)

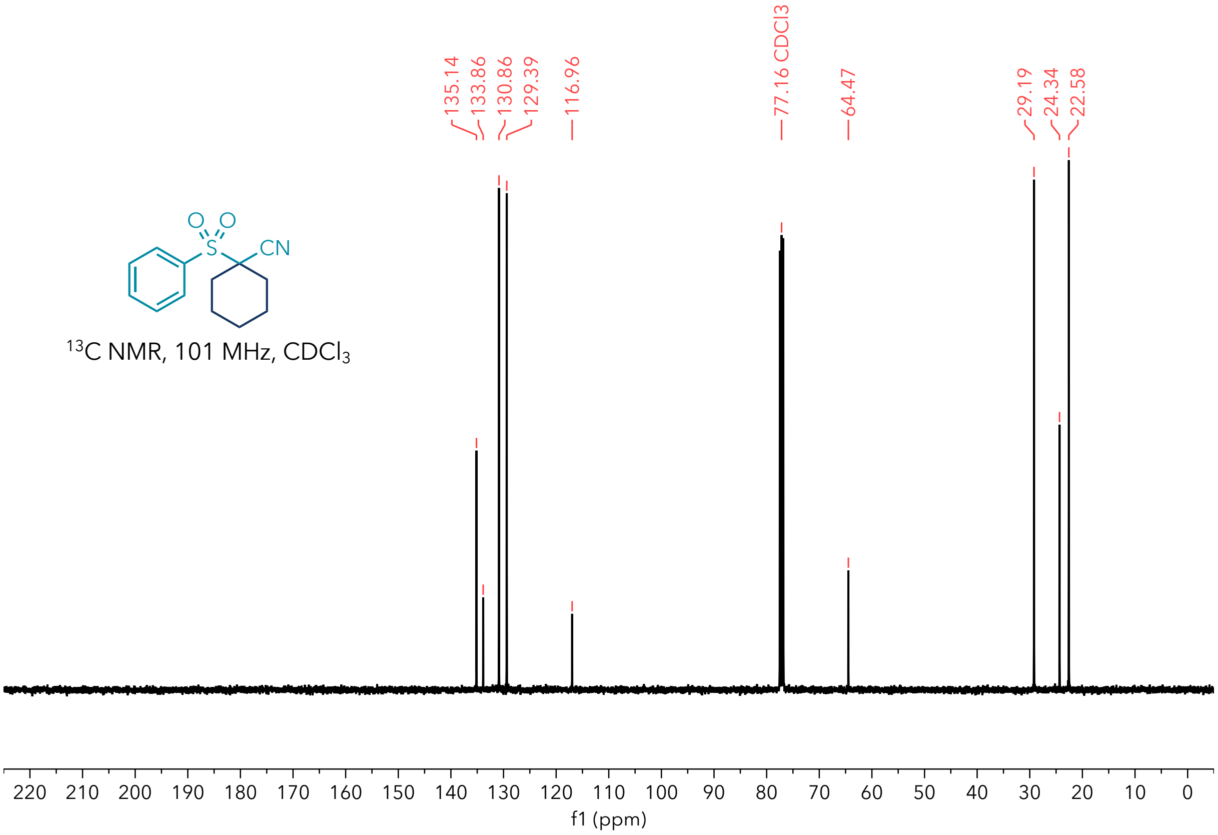
135.14
133.86
130.86
129.39
116.96
77.16 CDCl3
64.47
29.19
24.34
22.58
O
O
S
CN
^{13}C NMR, 101 MHz, $CDCl_3$
220
210
200
190
180
170
160
150
140
130
120
110
100
90
80
70
60
50
40
30
20
10
0
f1 (ppm)

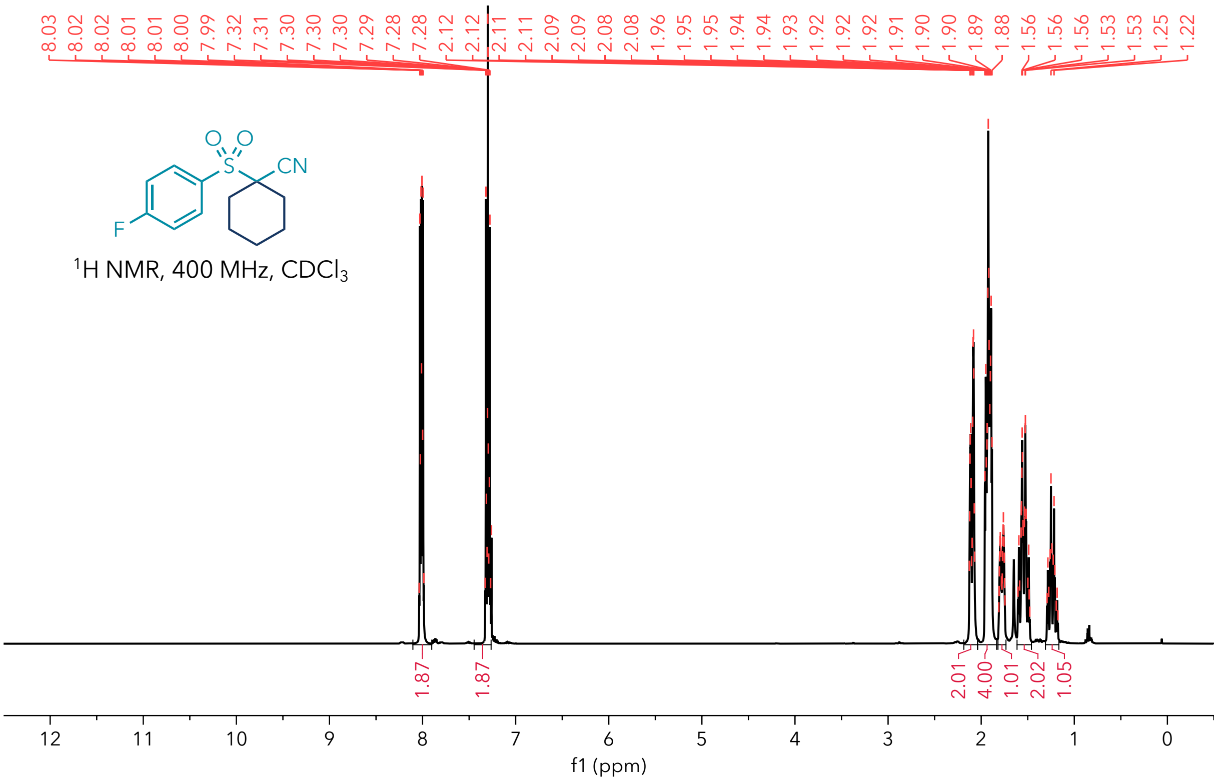

^{1}H NMR, 400 MHz, $CDCl_3$

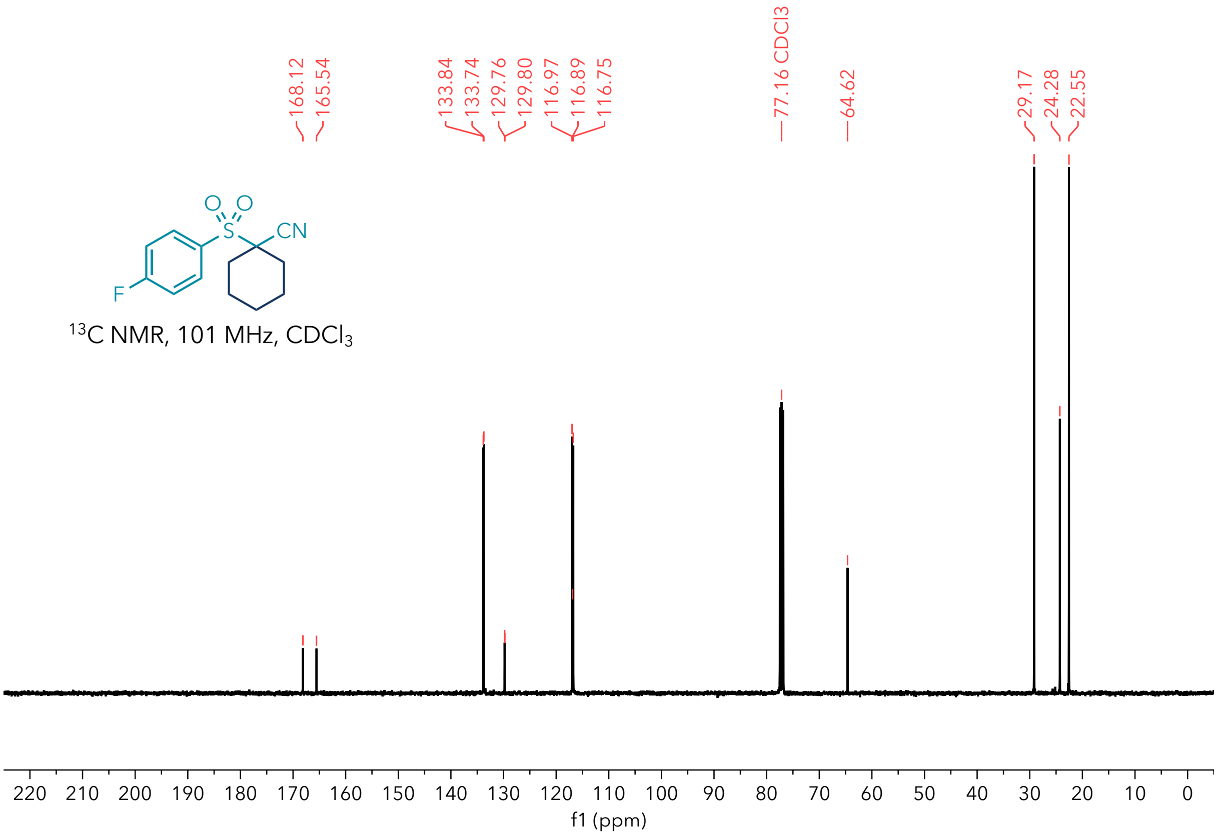

^{13}C NMR, 101 MHz, $CDCl_3$

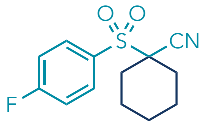

^{19}F NMR, 376 MHz, $CDCl_3$

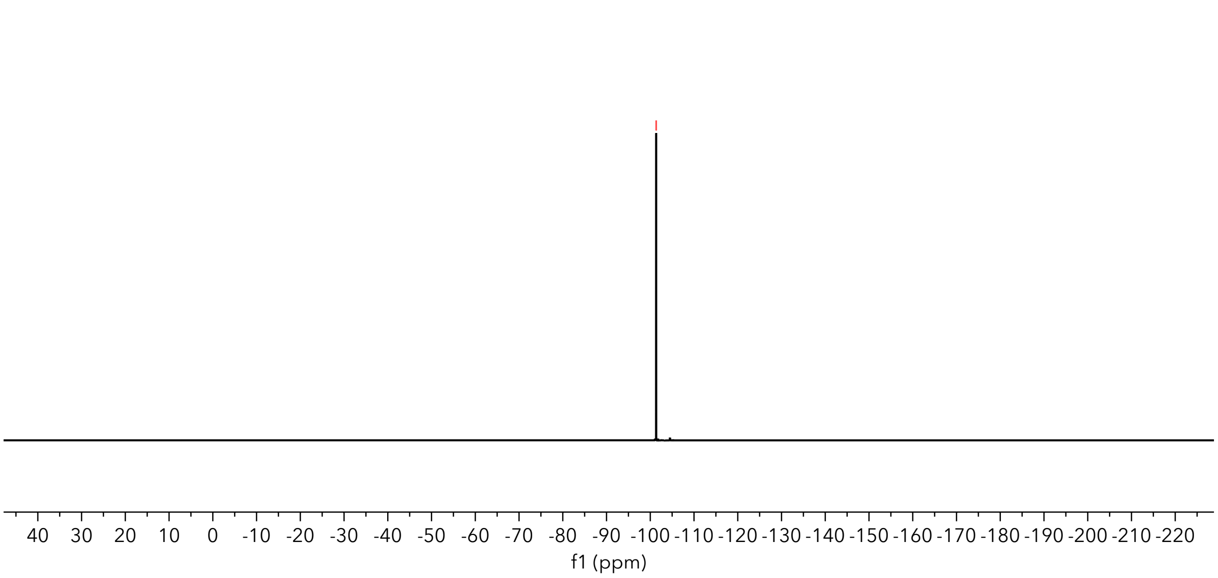

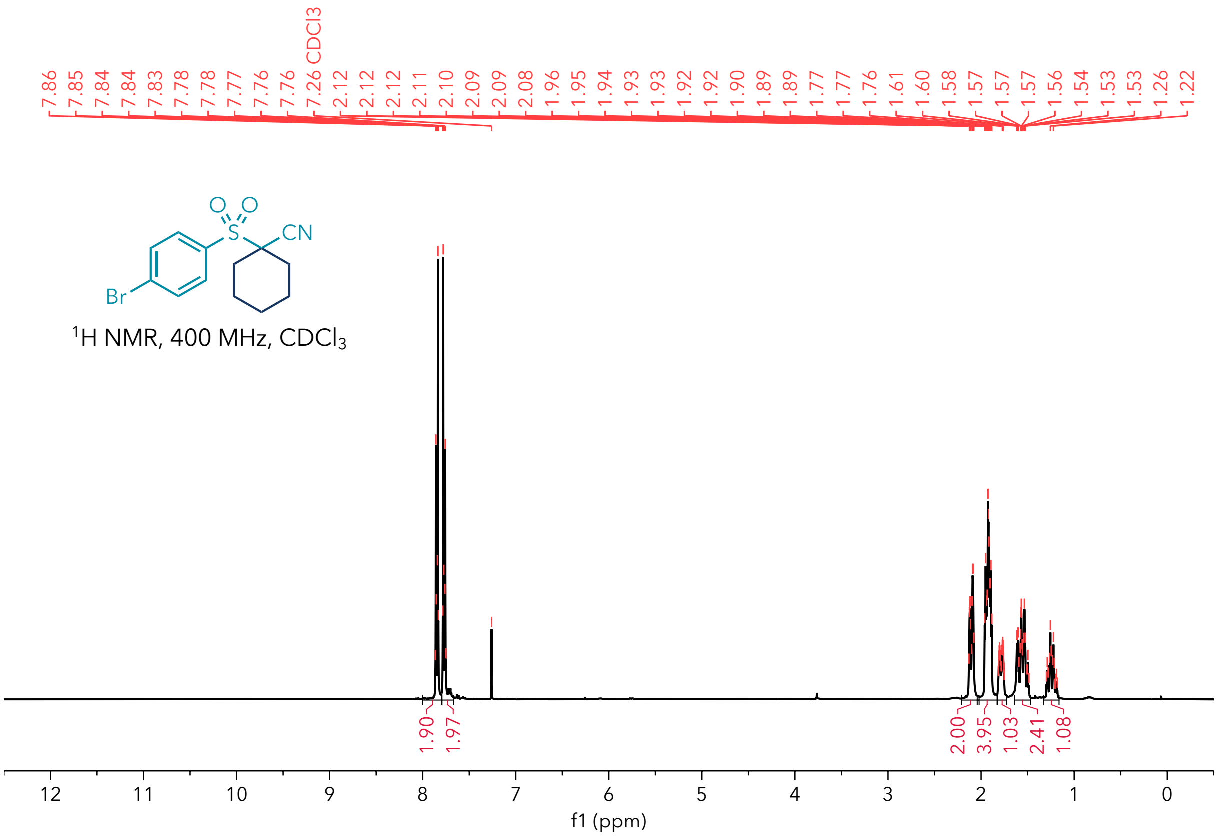
7.86
7.85
7.84
7.84
7.83
7.78
7.78
7.77
7.76
7.76
7.26 CDCl3
2.12
2.12
2.12
2.11
2.10
2.09
2.09
2.08
1.96
1.95
1.94
1.93
1.93
1.92
1.92
1.90
1.89
1.89
1.77
1.77
1.76
1.61
1.60
1.58
1.57
1.57
1.57
1.56
1.54
1.53
1.53
1.26
1.22
Br
O
O
S
CN
1H NMR, 400 MHz, CDCl3
1.90
1.97
2.00
3.95
1.03
2.41
1.08
12
11
10
9
8
7
6
5
4
3
2
1
0
f1 (ppm)

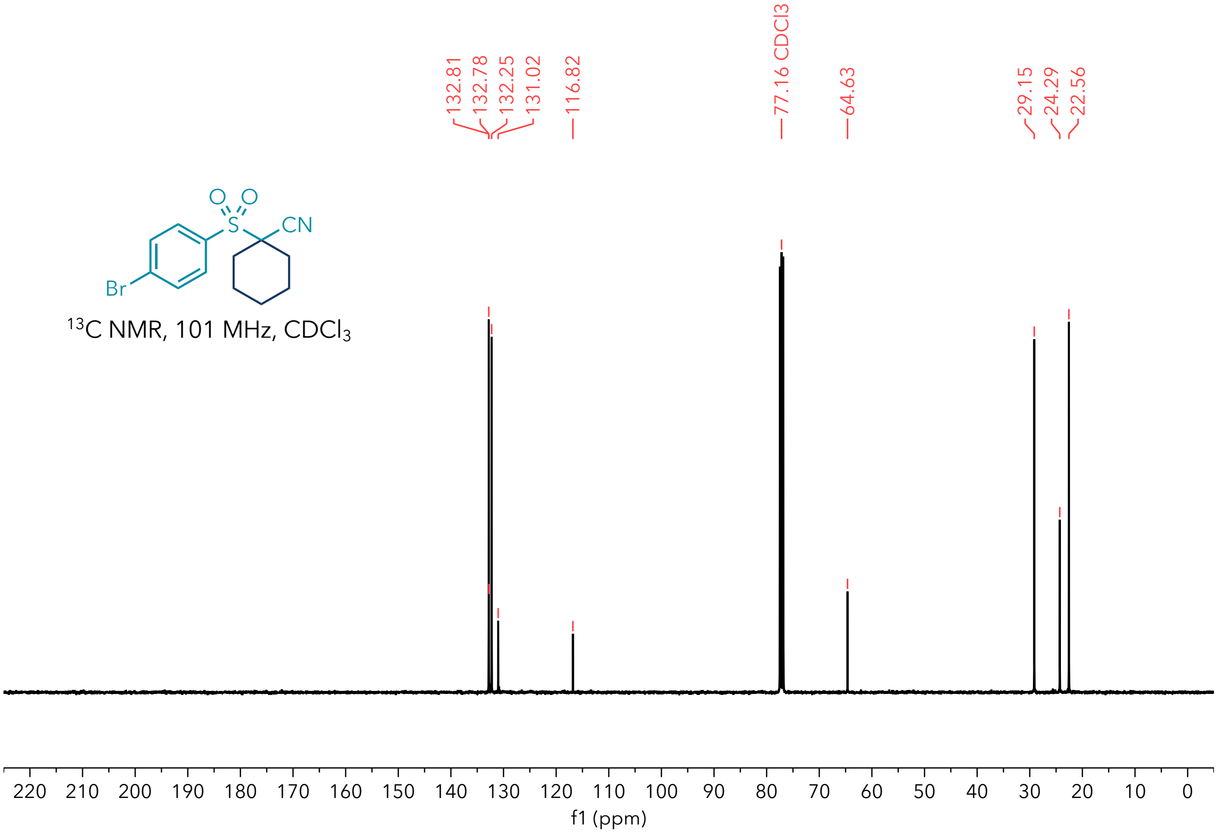
132.81
132.78
132.25
131.02
116.82
77.16 CDCl3
64.63
29.15
24.29
22.56
Br
O
O
S
CN
13C NMR, 101 MHz, CDCl3
220
210
200
190
180
170
160
150
140
130
120
110
100
90
80
70
60
50
40
30
20
10
0
f1 (ppm)

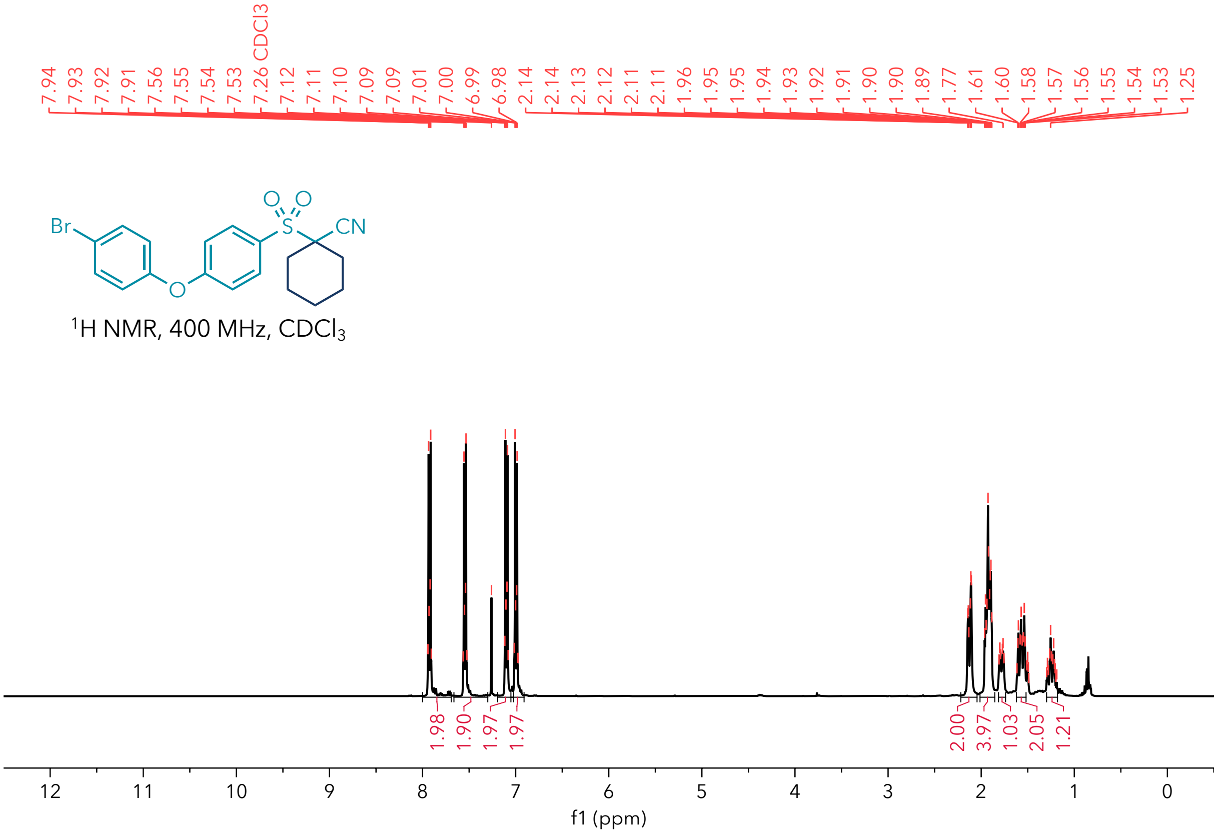

^{1}H NMR, 400 MHz, $CDCl_3$

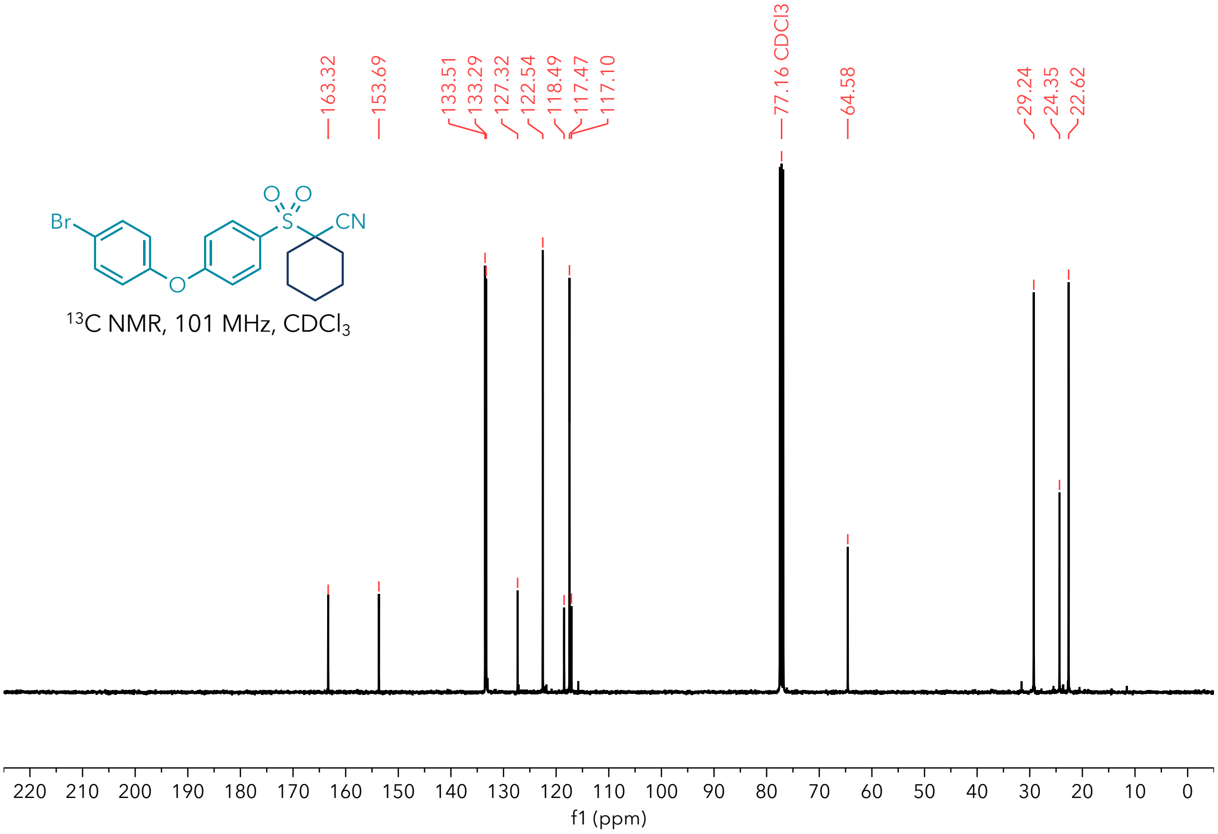

^{13}C NMR, 101 MHz, $CDCl_3$

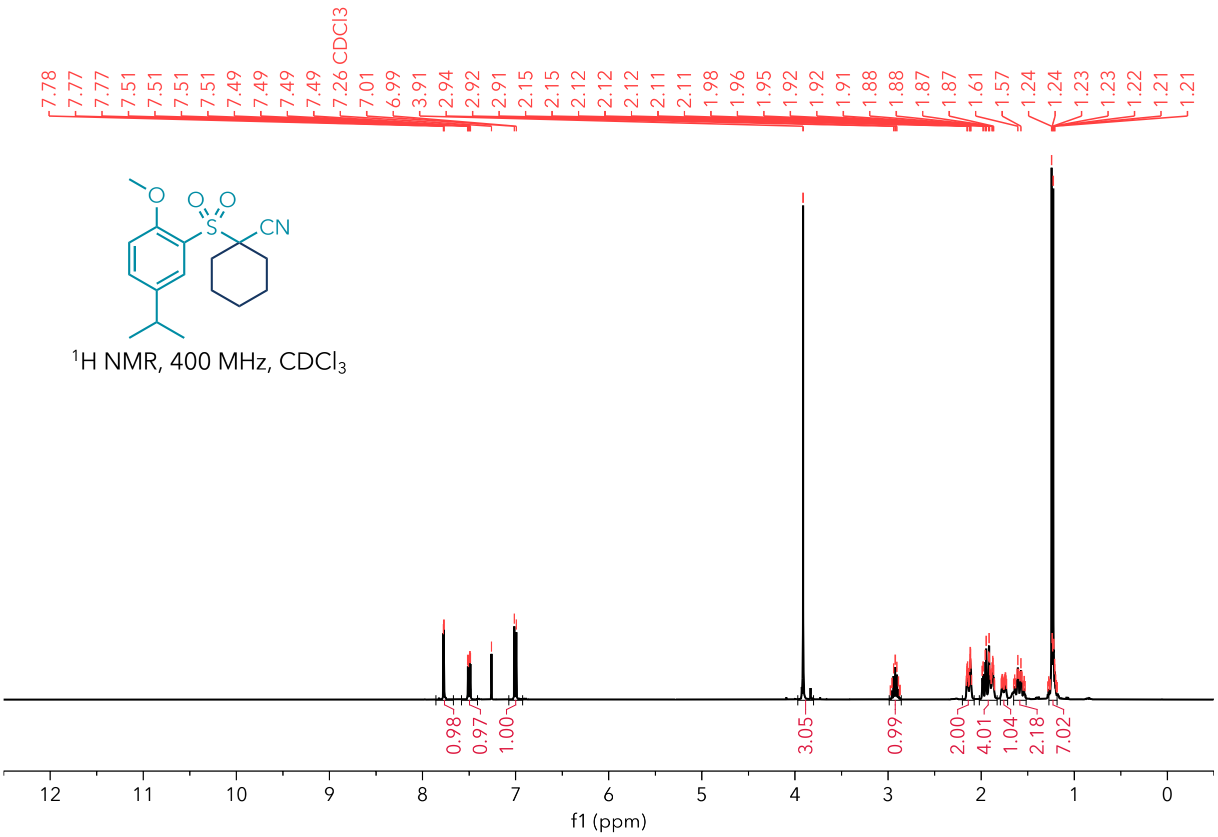
7.78 7.77 7.77 7.51 7.51 7.51 7.51 7.49 7.49 7.49 7.49 7.26 CDCl3 7.01 6.99 3.91 2.94 2.92 2.91 2.15 2.15 2.12 2.12 2.12 2.11 2.11 1.98 1.96 1.95 1.92 1.92 1.91 1.88 1.88 1.87 1.87 1.61 1.57 1.24 1.24 1.23 1.23 1.22 1.21 1.21
O
O
O
S
CN
^{1}H NMR, 400 MHz, $CDCl_3$
0.98
0.97
1.00
3.05
0.99
2.00
4.01
1.04
2.18
7.02
12 11 10 9 8 7 6 5 4 3 2 1 0
f1 (ppm)

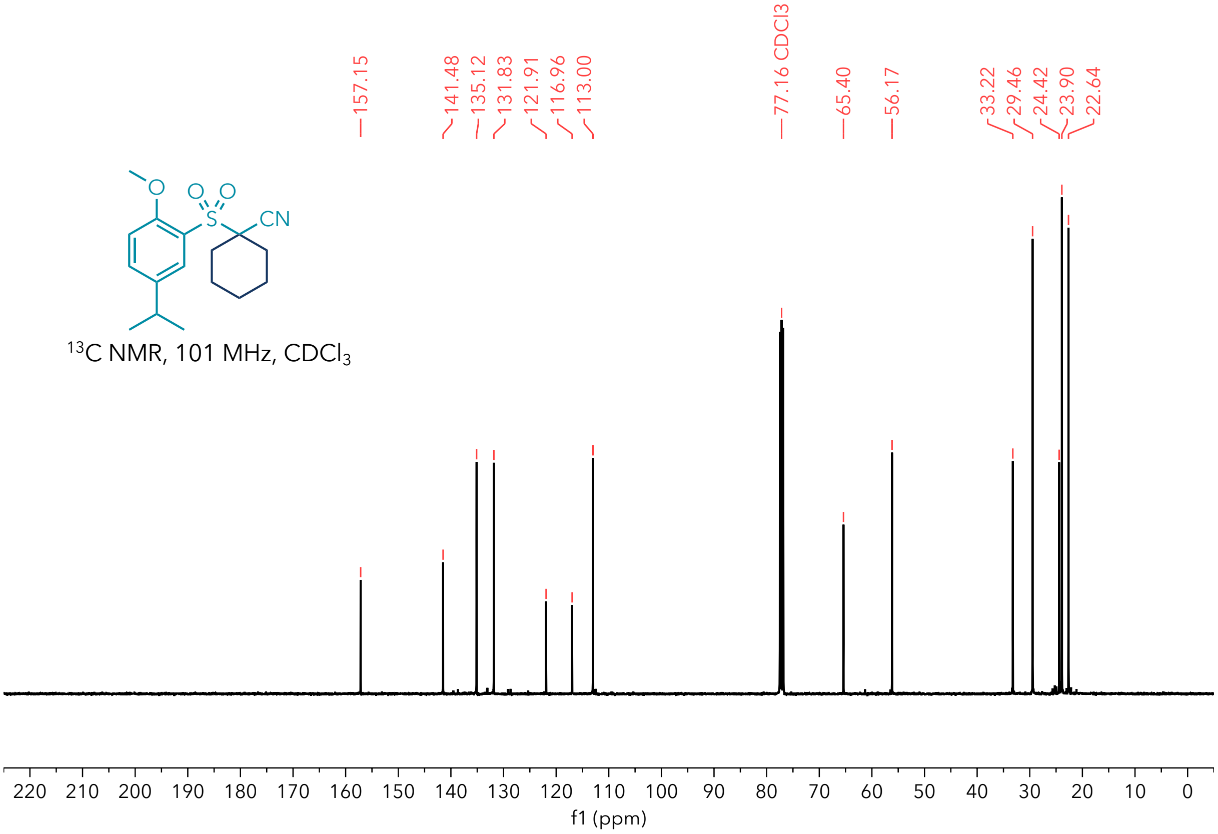
157.15
141.48 135.12 131.83 121.91 116.96 113.00
77.16 CDCl3
65.40
56.17
33.22 29.46 24.42 23.90 22.64
O
O
O
S
CN
^{13}C NMR, 101 MHz, $CDCl_3$
220 210 200 190 180 170 160 150 140 130 120 110 100 90 80 70 60 50 40 30 20 10 0
f1 (ppm)

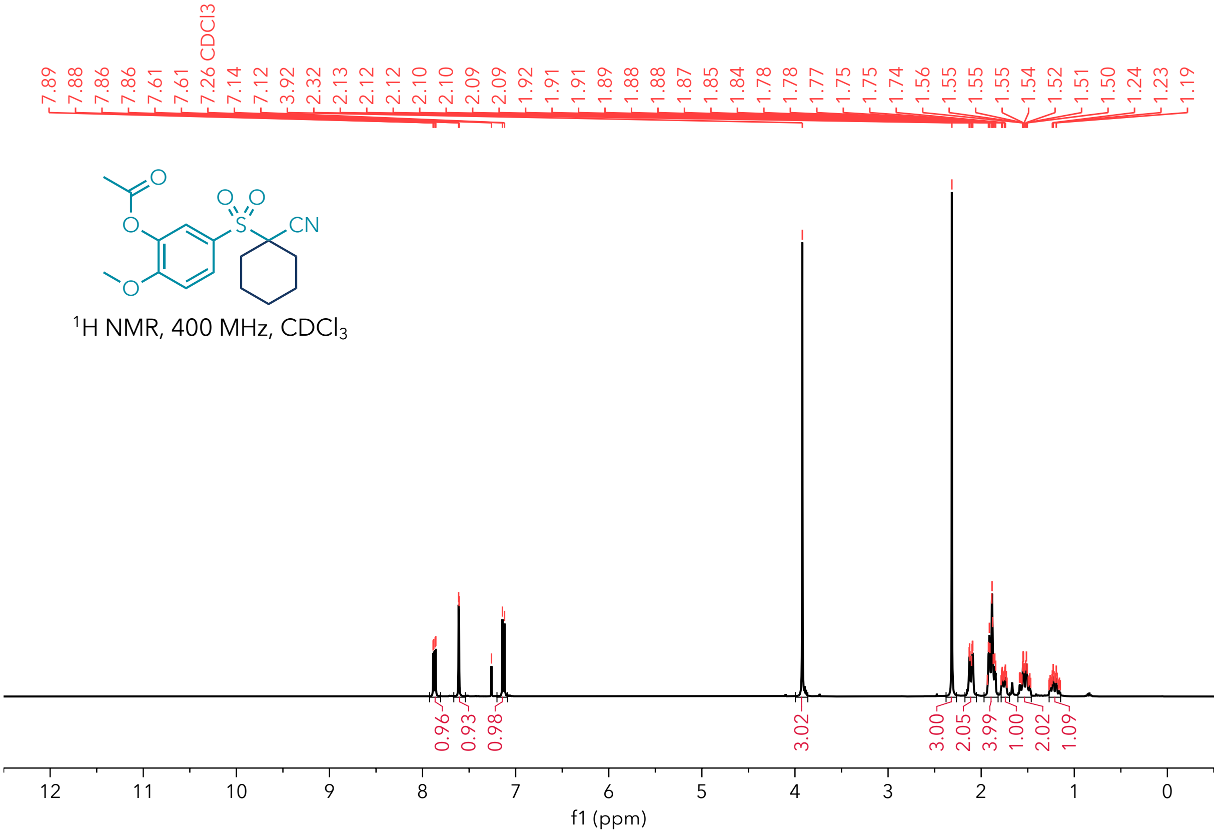

7.89
7.88
7.86
7.86
7.61
7.61
7.26 CDCl3
7.14
7.12
3.92
2.32
2.13
2.12
2.12
2.10
2.10
2.09
2.09
1.92
1.91
1.91
1.89
1.88
1.88
1.87
1.85
1.84
1.78
1.78
1.77
1.75
1.75
1.74
1.56
1.55
1.55
1.55
1.54
1.52
1.51
1.50
1.24
1.23
1.19
CN
^{1}H NMR, 400 MHz, CDCl$_3$
0.96
0.93
0.98
3.02
3.00
2.05
3.99
1.00
2.02
1.09
12
11
10
9
8
7
6
5
4
3
2
1
0
f1 (ppm)

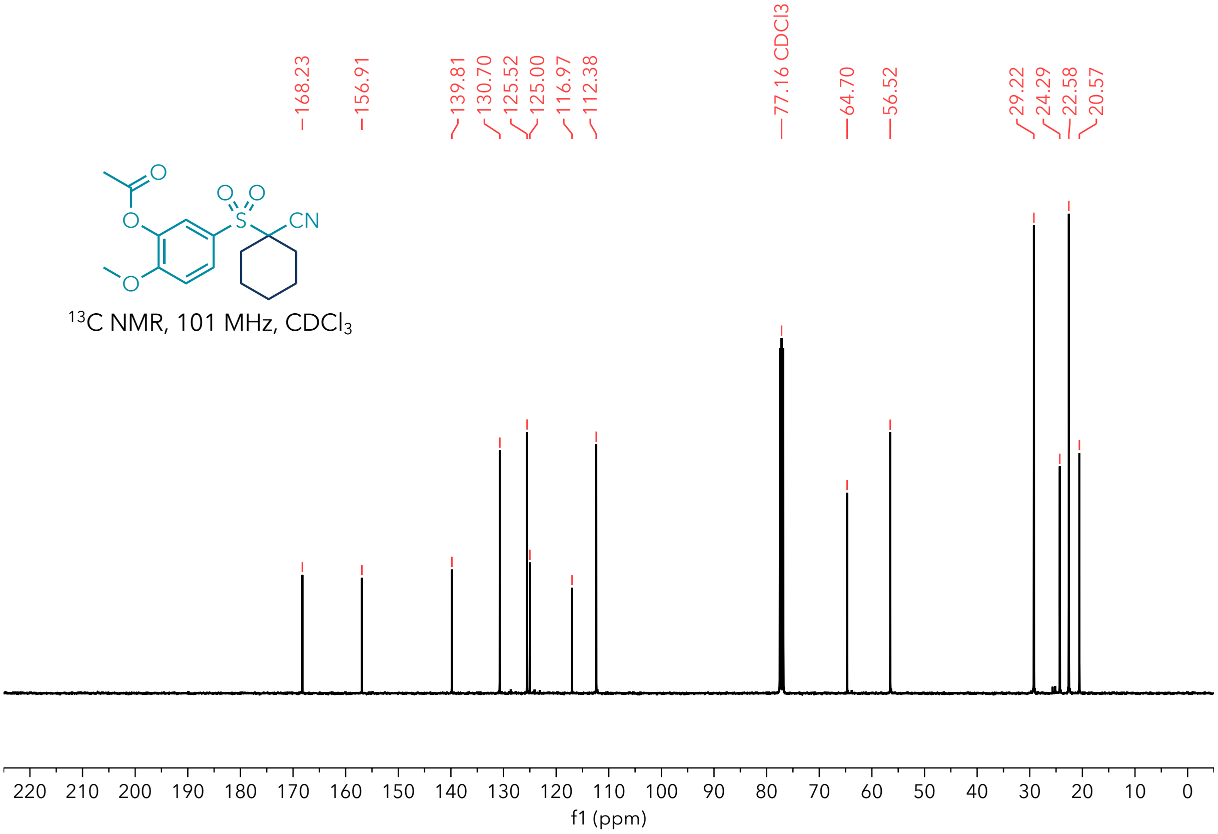

168.23
156.91
139.81
130.70
125.52
125.00
116.97
112.38
77.16 CDCl3
64.70
56.52
29.22
24.29
22.58
20.57
CN
^{13}C NMR, 101 MHz, CDCl$_3$
220
210
200
190
180
170
160
150
140
130
120
110
100
90
80
70
60
50
40
30
20
10
0
f1 (ppm)

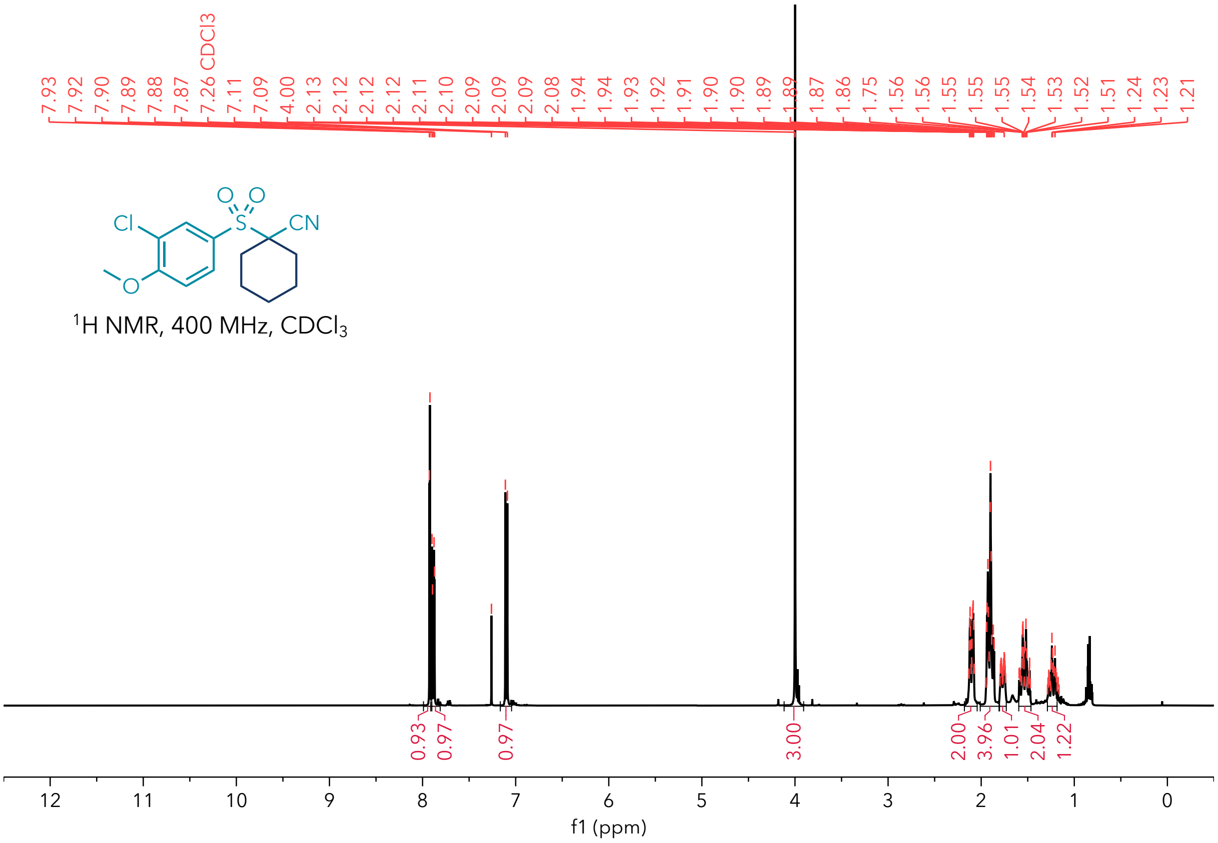
7.93 7.92 7.90 7.89 7.88 7.87 7.26 CDCl3 7.11 7.09 4.00 2.13 2.12 2.12 2.12 2.11 2.10 2.09 2.09 2.09 2.08 1.94 1.94 1.93 1.92 1.91 1.90 1.90 1.89 1.89 1.87 1.86 1.75 1.56 1.56 1.55 1.55 1.55 1.54 1.53 1.52 1.51 1.24 1.23 1.21
Cl
O
S
CN
^{1}H NMR, 400 MHz, $CDCl_3$
0.93 0.97 0.97 3.00 2.00 3.96 1.01 2.04 1.22
12 11 10 9 8 7 6 5 4 3 2 1 0
f1 (ppm)

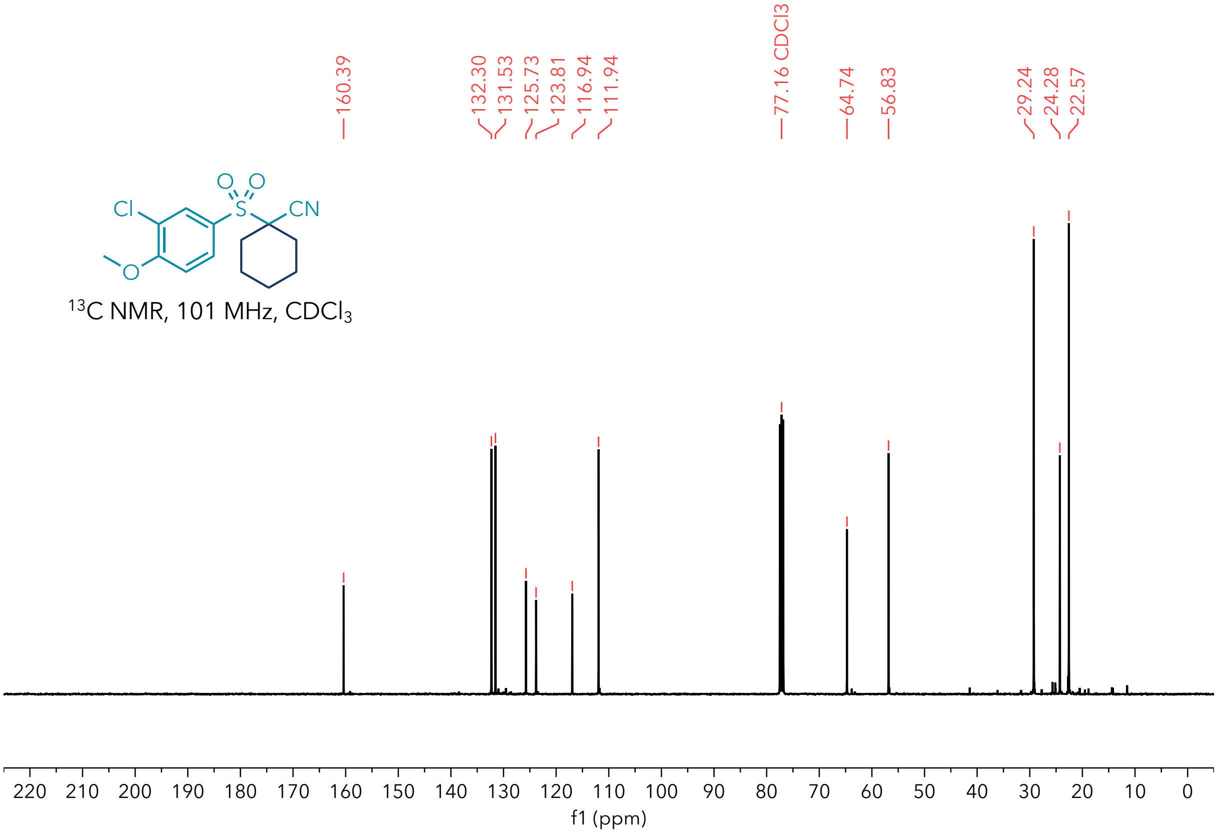
160.39 132.30 131.53 125.73 123.81 116.94 111.94 77.16 CDCl3 64.74 56.83 29.24 24.28 22.57
Cl
O
S
CN
^{13}C NMR, 101 MHz, $CDCl_3$
220 210 200 190 180 170 160 150 140 130 120 110 100 90 80 70 60 50 40 30 20 10 0
f1 (ppm)

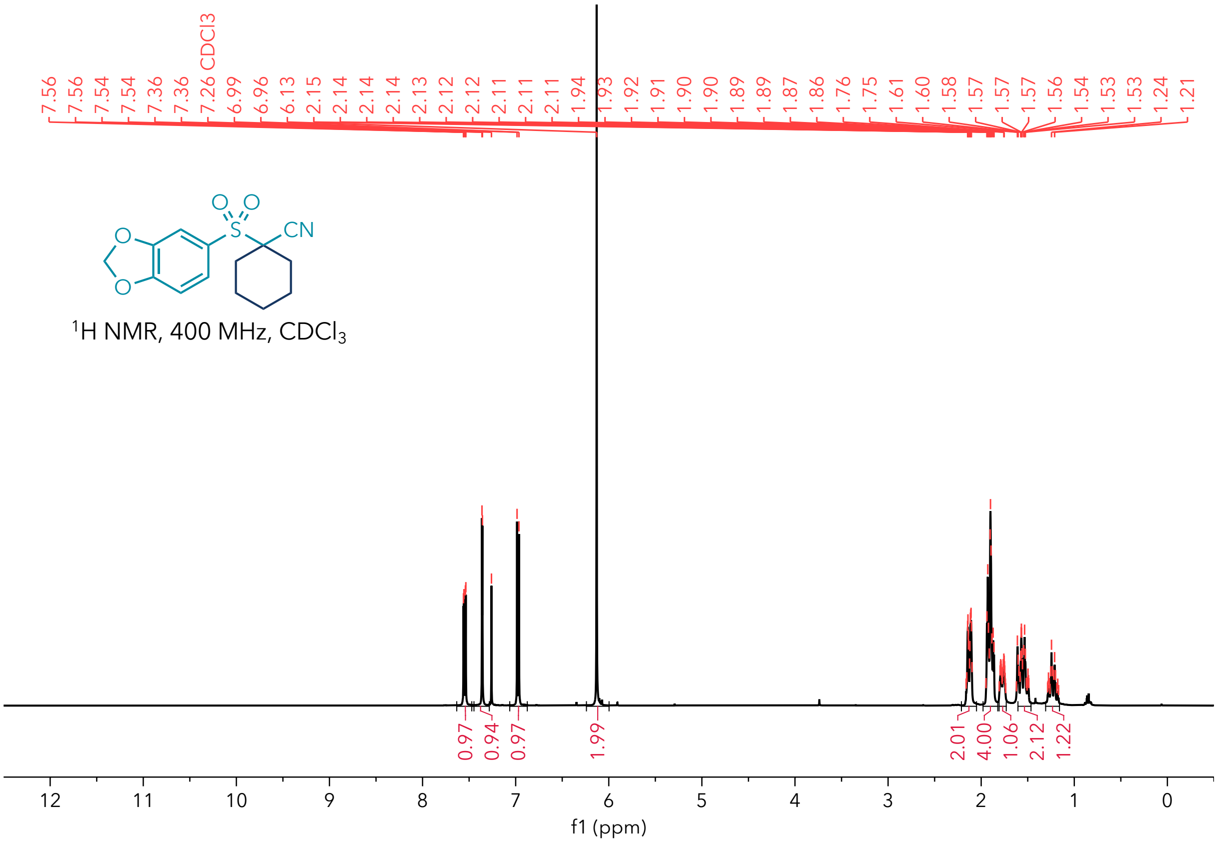
7.56 7.56 7.54 7.54 7.36 7.36 7.26 CDCl3 6.99 6.96 6.13 2.15 2.14 2.14 2.14 2.13 2.12 2.12 2.11 2.11 2.11 1.94 1.93 1.92 1.91 1.90 1.90 1.89 1.89 1.87 1.86 1.76 1.75 1.61 1.60 1.58 1.57 1.57 1.57 1.56 1.54 1.53 1.53 1.24 1.21
O
O
S
CN
O
O
^{1}H NMR, 400 MHz, $CDCl_3$
0.97 0.94 0.97 1.99 2.01 4.00 1.06 2.12 1.22
12 11 10 9 8 7 6 5 4 3 2 1 0
f1 (ppm)

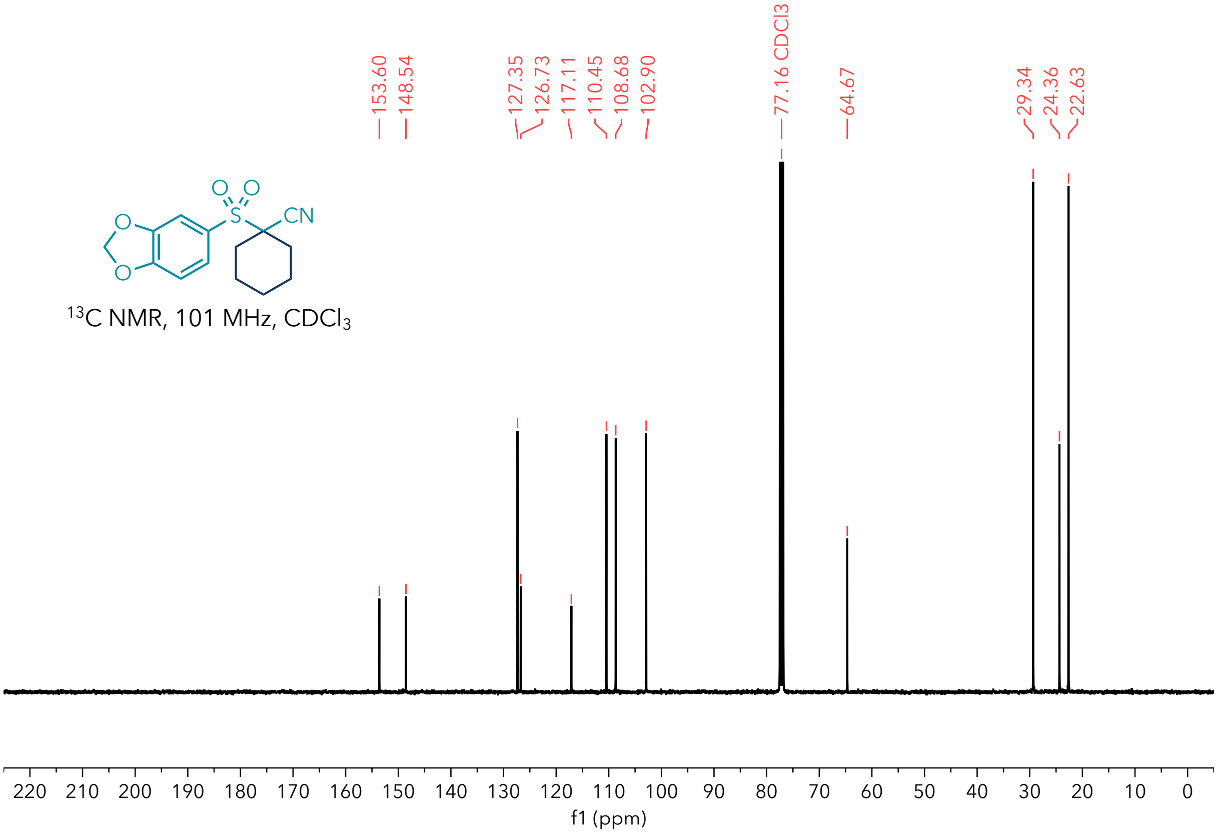
153.60 148.54 127.35 126.73 117.11 110.45 108.68 102.90 77.16 CDCl3 64.67 29.34 24.36 22.63
O
O
S
CN
O
O
^{13}C NMR, 101 MHz, $CDCl_3$
220 210 200 190 180 170 160 150 140 130 120 110 100 90 80 70 60 50 40 30 20 10 0
f1 (ppm)

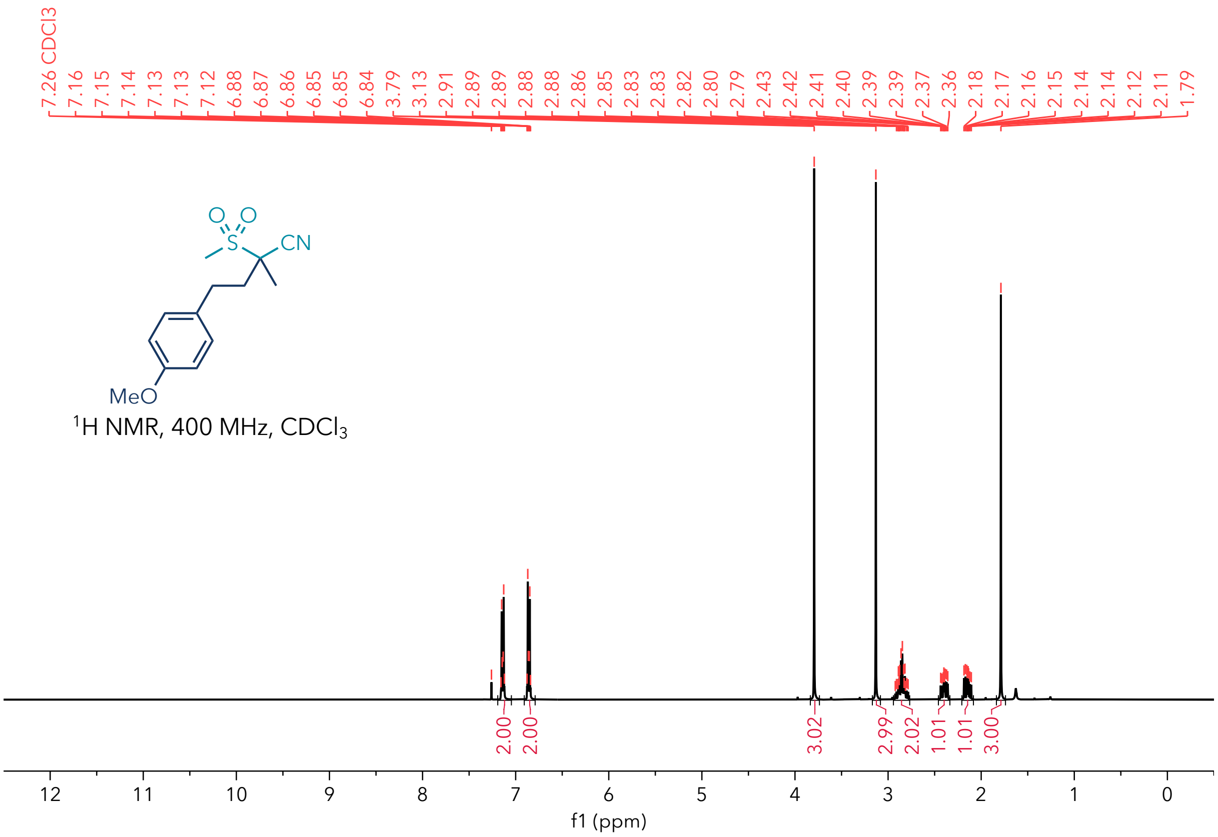
7.26 CDCl3
7.16 7.15 7.14 7.13 7.13 7.12 6.88 6.87 6.86 6.85 6.85 6.84 3.79 3.13 2.91 2.89 2.89 2.88 2.88 2.86 2.85 2.83 2.83 2.82 2.80 2.79 2.43 2.42 2.41 2.40 2.39 2.39 2.37 2.36 2.18 2.17 2.16 2.15 2.14 2.14 2.12 2.11 1.79
O O
S
CN
MeO
1H NMR, 400 MHz, CDCl3
2.00 2.00 3.02 2.99 2.02 1.01 1.01 3.00
12 11 10 9 8 7 6 5 4 3 2 1 0
f1 (ppm)

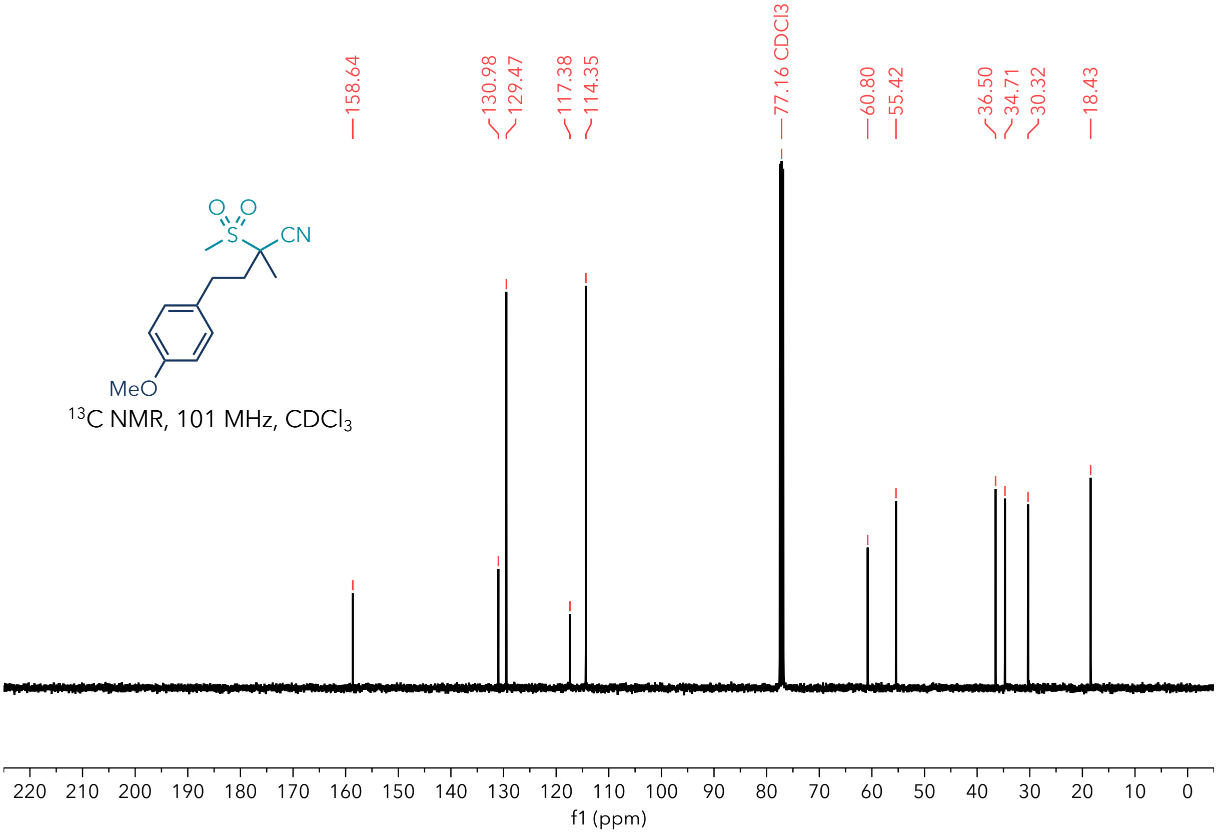
158.64
130.98 129.47
117.38 114.35
77.16 CDCl3
60.80 55.42
36.50 34.71 30.32
18.43
O O
S
CN
MeO
13C NMR, 101 MHz, CDCl3
220 210 200 190 180 170 160 150 140 130 120 110 100 90 80 70 60 50 40 30 20 10 0
f1 (ppm)

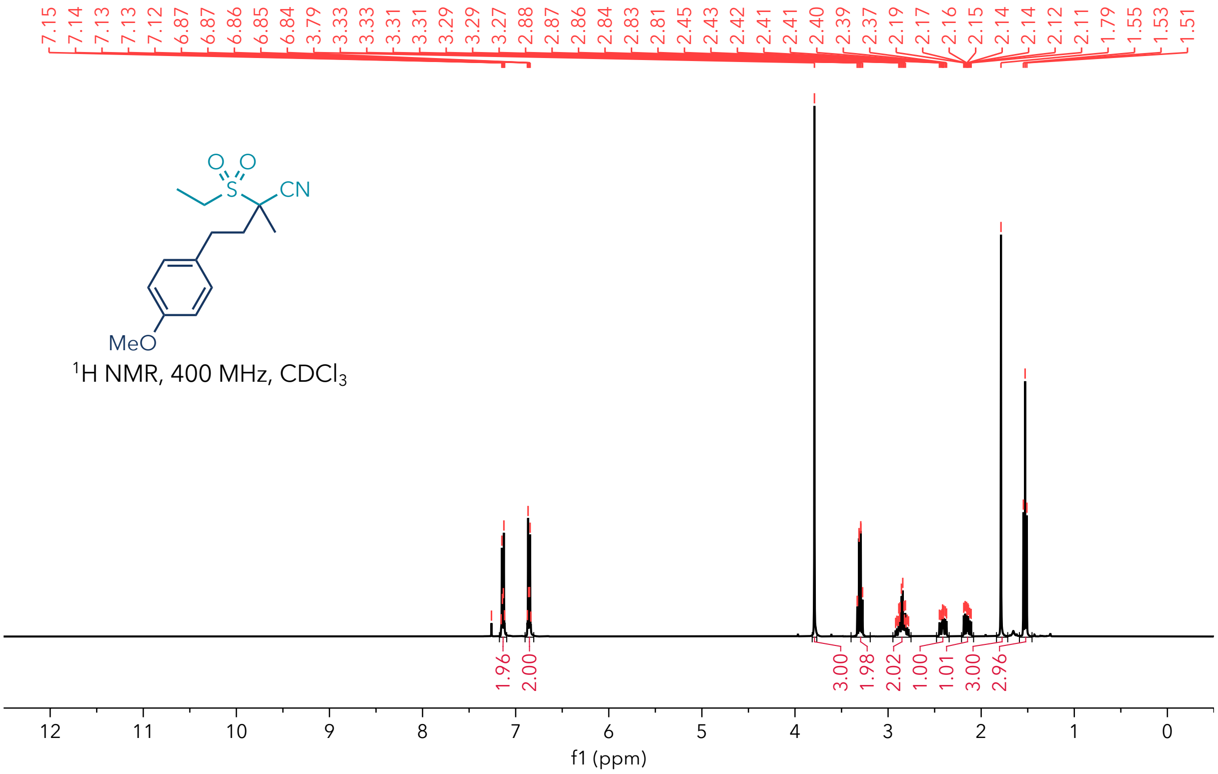
7.15 7.14 7.13 7.13 7.12 6.87 6.87 6.86 6.85 6.84 3.79 3.33 3.33 3.31 3.31 3.29 3.29 3.27 2.88 2.87 2.86 2.84 2.83 2.81 2.45 2.43 2.42 2.41 2.41 2.40 2.39 2.37 2.19 2.17 2.16 2.15 2.14 2.14 2.12 2.11 1.79 1.55 1.53 1.51
O O
S
CN
MeO
^{1}H NMR, 400 MHz, $CDCl_3$
1.96 2.00 3.00 1.98 2.02 1.00 1.01 3.00 2.96
12 11 10 9 8 7 6 5 4 3 2 1 0
f1 (ppm)

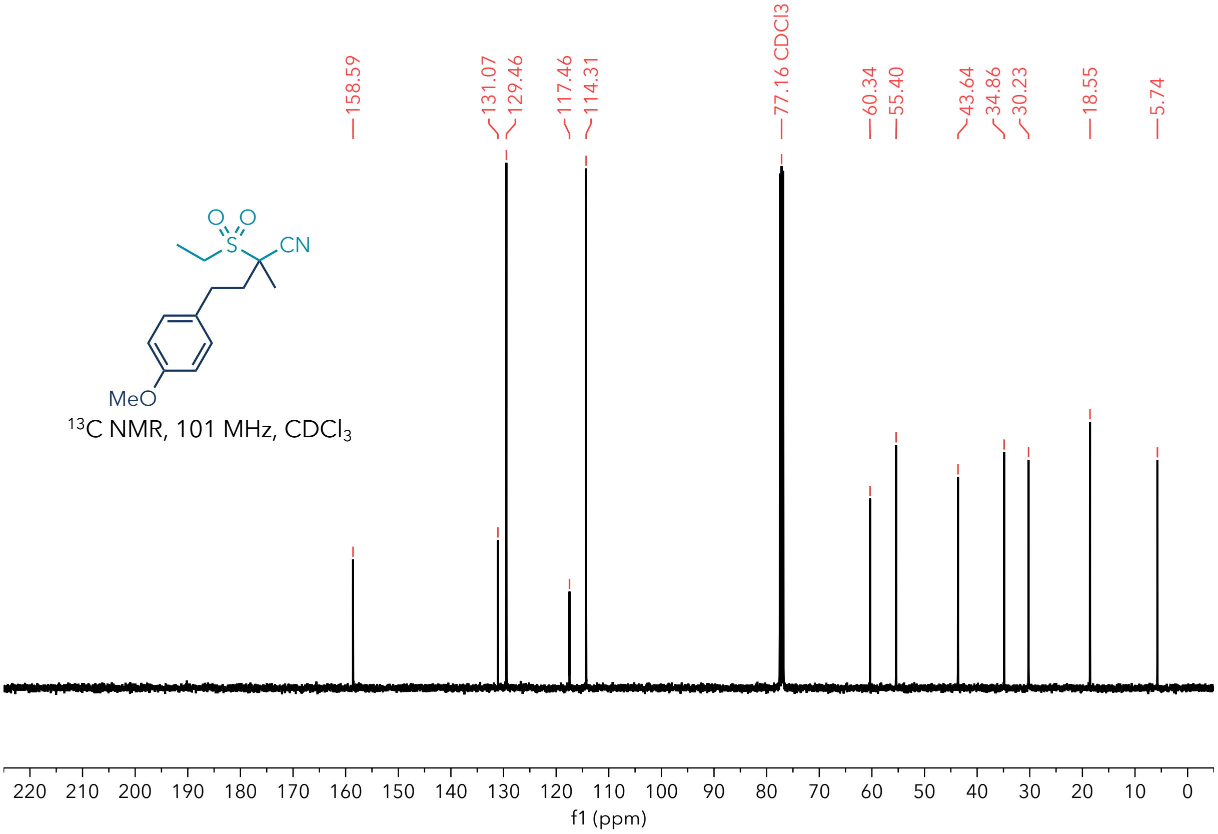
158.59
131.07 129.46
117.46 114.31
77.16 CDCl3
60.34 55.40
43.64 34.86 30.23
18.55
5.74
O O
S
CN
MeO
^{13}C NMR, 101 MHz, $CDCl_3$
220 210 200 190 180 170 160 150 140 130 120 110 100 90 80 70 60 50 40 30 20 10 0
f1 (ppm)

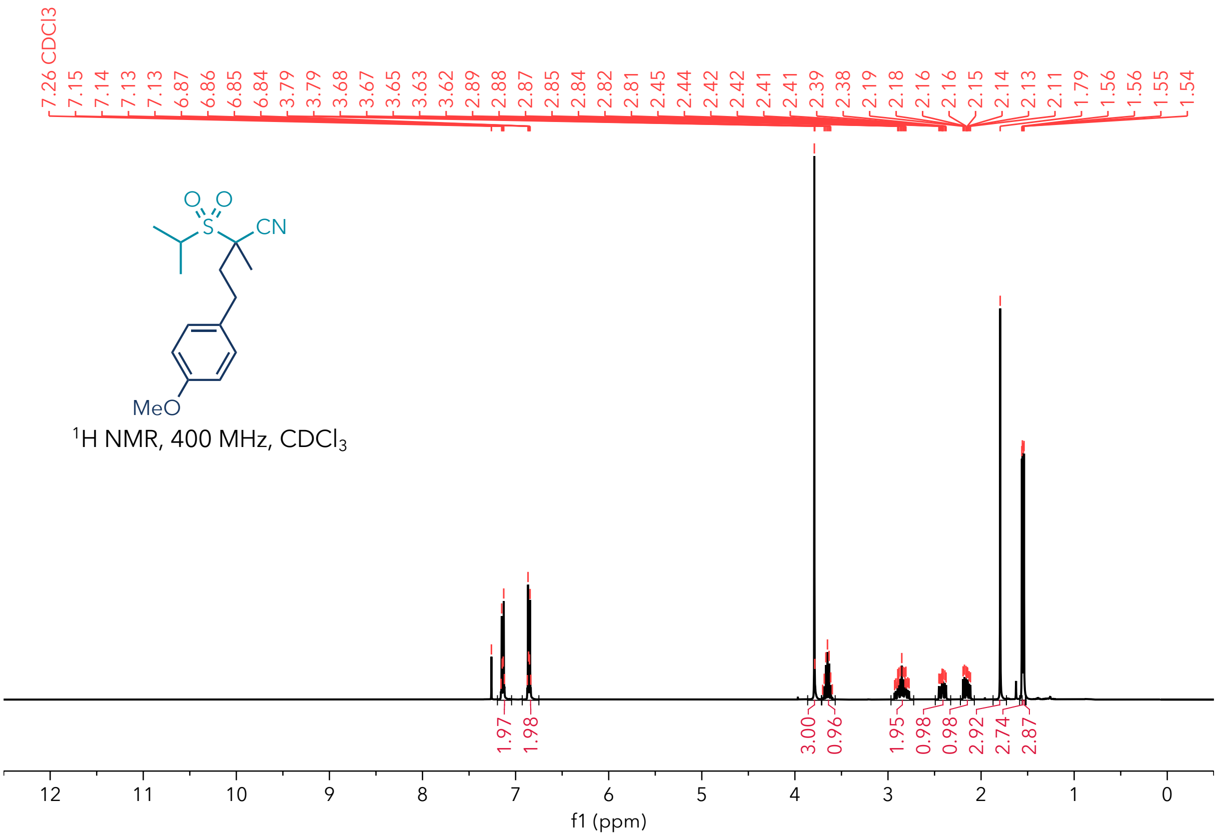

7.26 CDCl3
7.15
7.14
7.13
7.13
6.87
6.86
6.85
6.84
3.79
3.79
3.68
3.67
3.65
3.63
3.62
2.89
2.88
2.87
2.85
2.84
2.82
2.81
2.45
2.44
2.42
2.42
2.41
2.41
2.39
2.38
2.19
2.18
2.16
2.16
2.15
2.14
2.13
2.11
1.79
1.56
1.56
1.55
1.54
O
O
S
CN
MeO
^{1}H NMR, 400 MHz, $CDCl_3$
1.97
1.98
3.00
0.96
1.95
0.98
0.98
2.92
2.74
2.87
12
11
10
9
8
7
6
5
4
3
2
1
0
f1 (ppm)

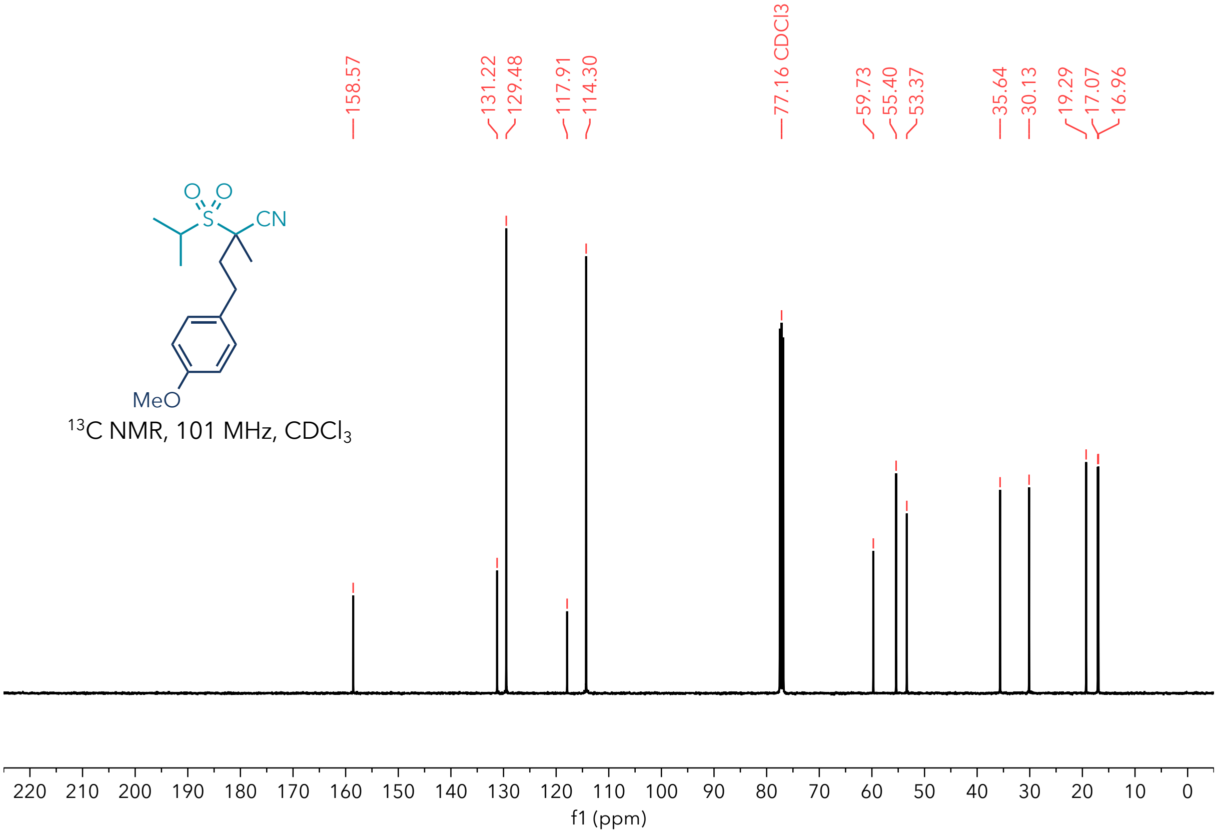

158.57
131.22
129.48
117.91
114.30
77.16 CDCl3
59.73
55.40
53.37
35.64
30.13
19.29
17.07
16.96
O
O
S
CN
MeO
^{13}C NMR, 101 MHz, $CDCl_3$
220
210
200
190
180
170
160
150
140
130
120
110
100
90
80
70
60
50
40
30
20
10
0
f1 (ppm)

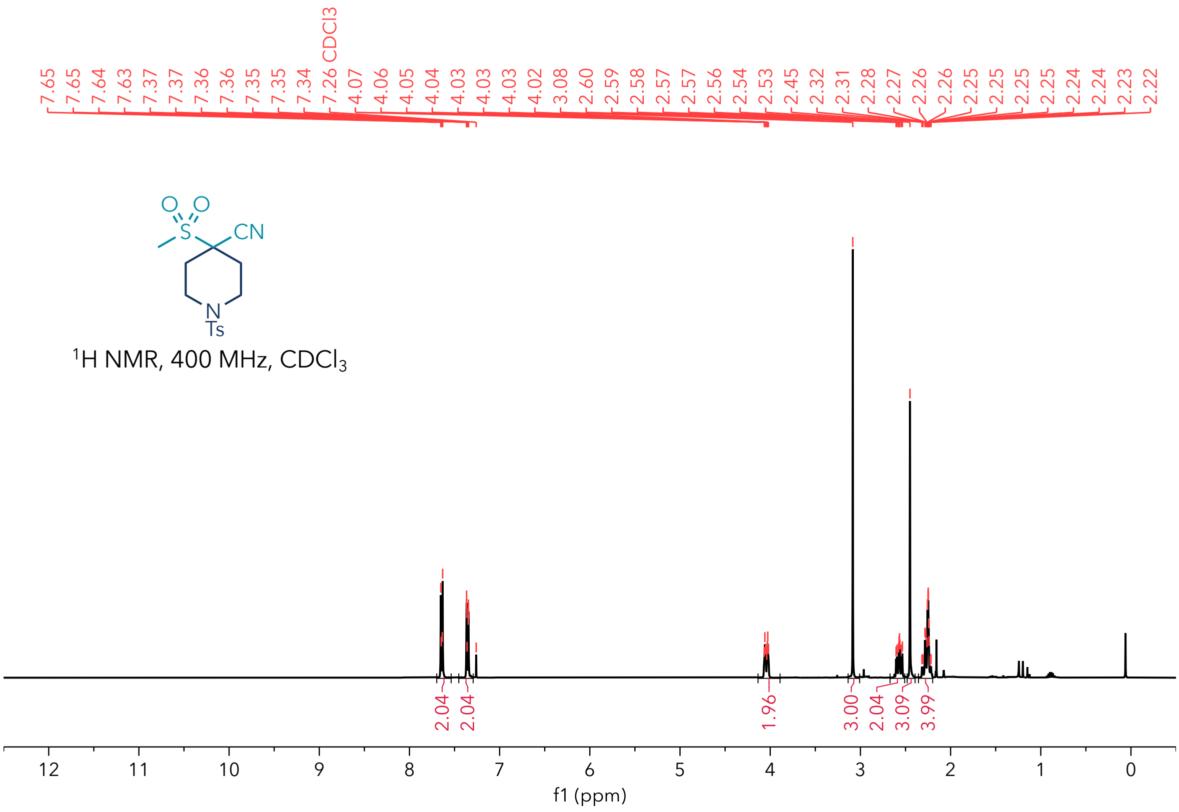
7.65 7.65 7.64 7.63 7.37 7.37 7.36 7.36 7.35 7.35 7.34 7.26 CDCl3 4.07 4.06 4.05 4.04 4.03 4.03 4.03 4.02 3.08 2.60 2.59 2.58 2.57 2.57 2.56 2.54 2.53 2.45 2.32 2.31 2.28 2.27 2.26 2.26 2.25 2.25 2.25 2.25 2.24 2.24 2.23 2.22
O O
S
CN
N
Ts
1H NMR, 400 MHz, CDCl3
2.04 2.04 1.96 3.00 2.04 3.09 3.99
f1 (ppm)

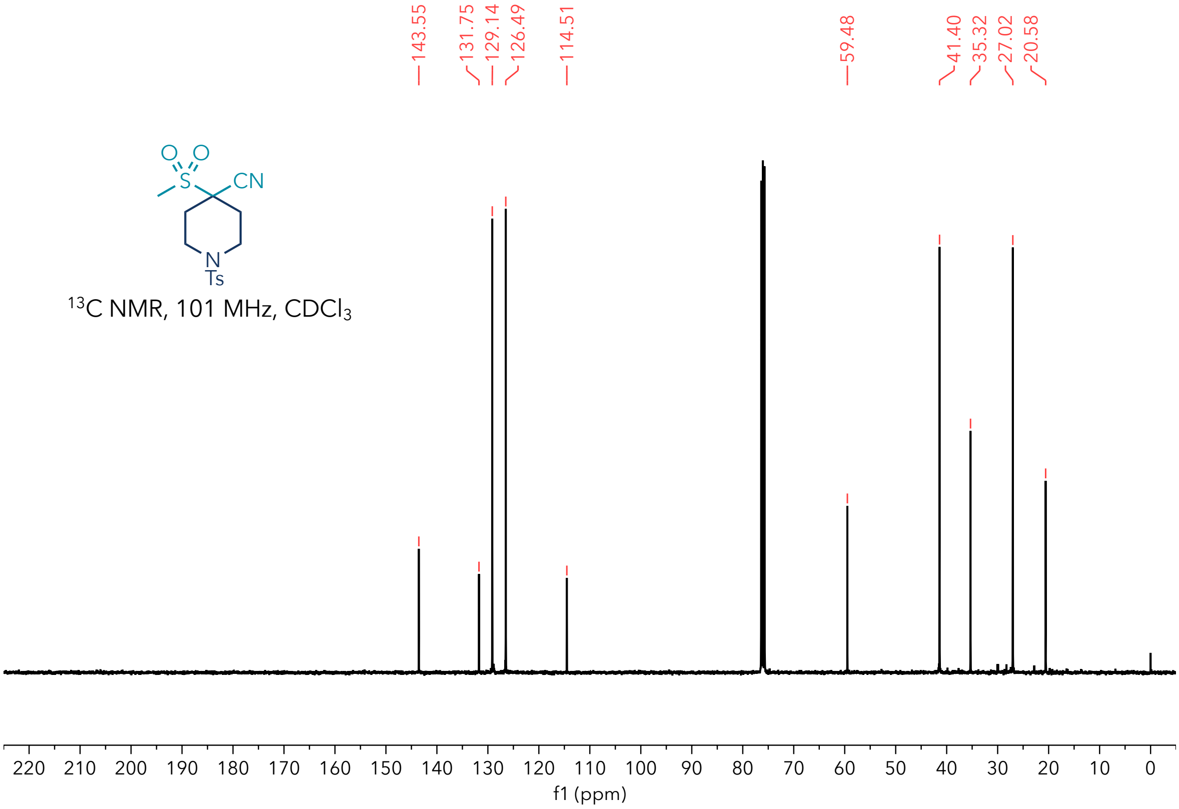
143.55 131.75 129.14 126.49 114.51 59.48 41.40 35.32 27.02 20.58
O O
S
CN
N
Ts
13C NMR, 101 MHz, CDCl3
f1 (ppm)

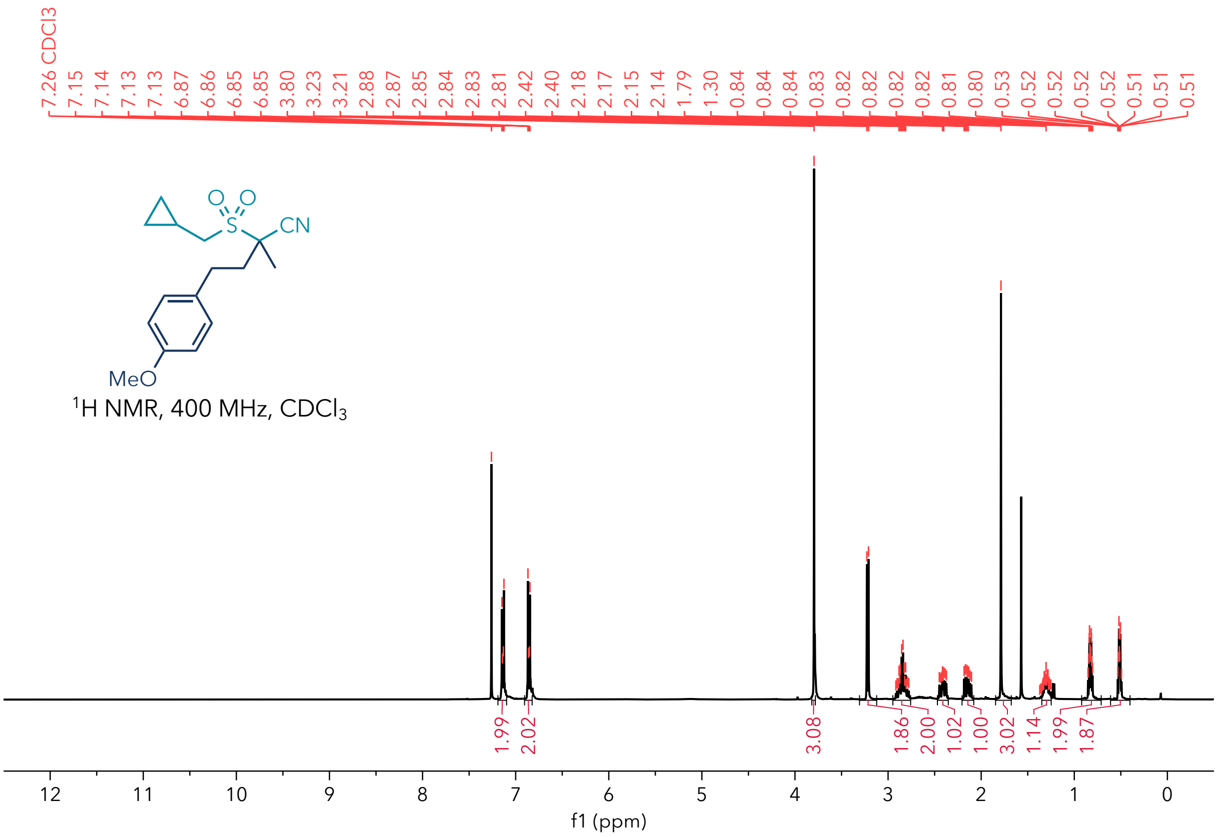
7.26 CDCl3
7.15
7.14
7.13
7.13
6.87
6.86
6.85
6.85
3.80
3.23
3.21
2.88
2.87
2.85
2.84
2.83
2.81
2.42
2.40
2.18
2.17
2.15
2.14
1.79
1.30
0.84
0.84
0.84
0.83
0.82
0.82
0.82
0.82
0.81
0.80
0.53
0.52
0.52
0.52
0.52
0.51
0.51
0.51
CN
MeO
^{1}H NMR, 400 MHz, $CDCl_3$
1.99
2.02
3.08
1.86
2.00
1.02
1.00
3.02
1.14
1.99
1.87
f1 (ppm)

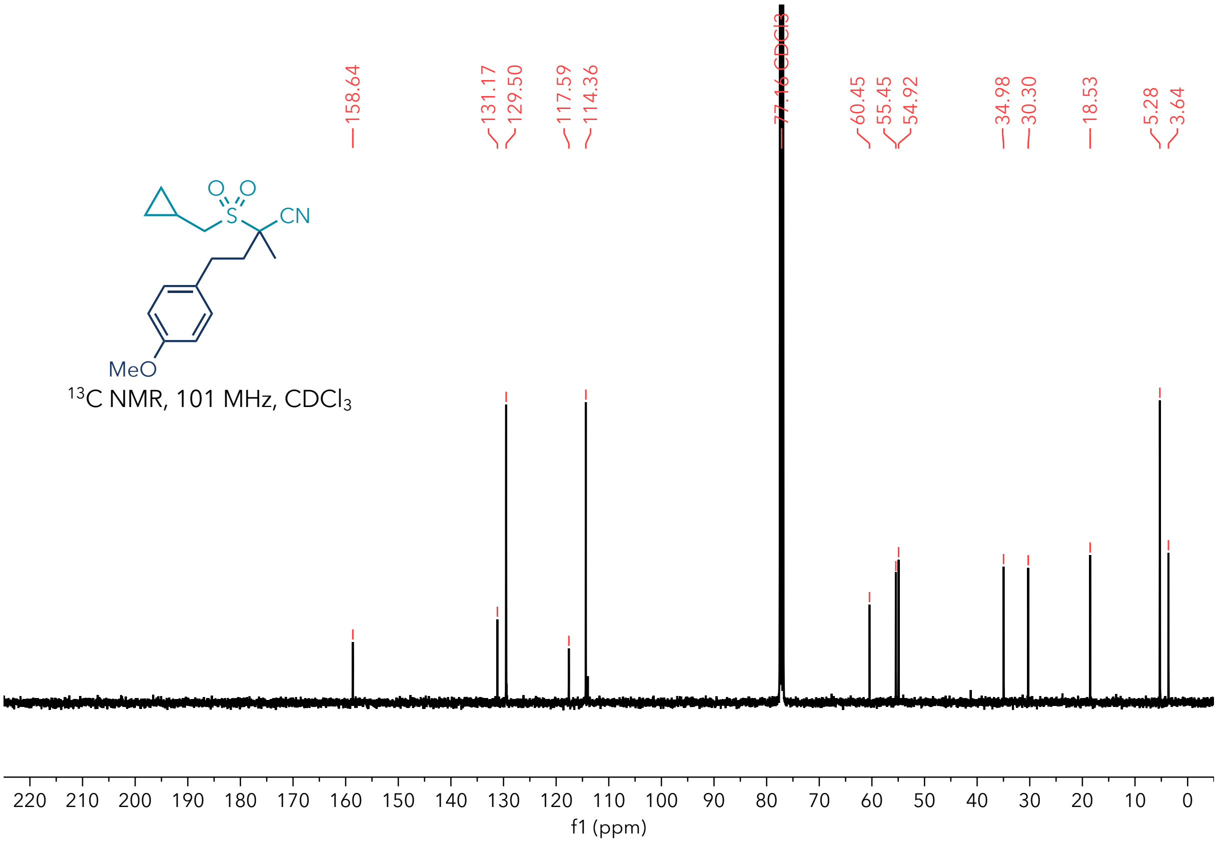
158.64
131.17
129.50
117.59
114.36
77.16 CDCl3
60.45
55.45
54.92
34.98
30.30
18.53
5.28
3.64
CN
MeO
^{13}C NMR, 101 MHz, $CDCl_3$
f1 (ppm)

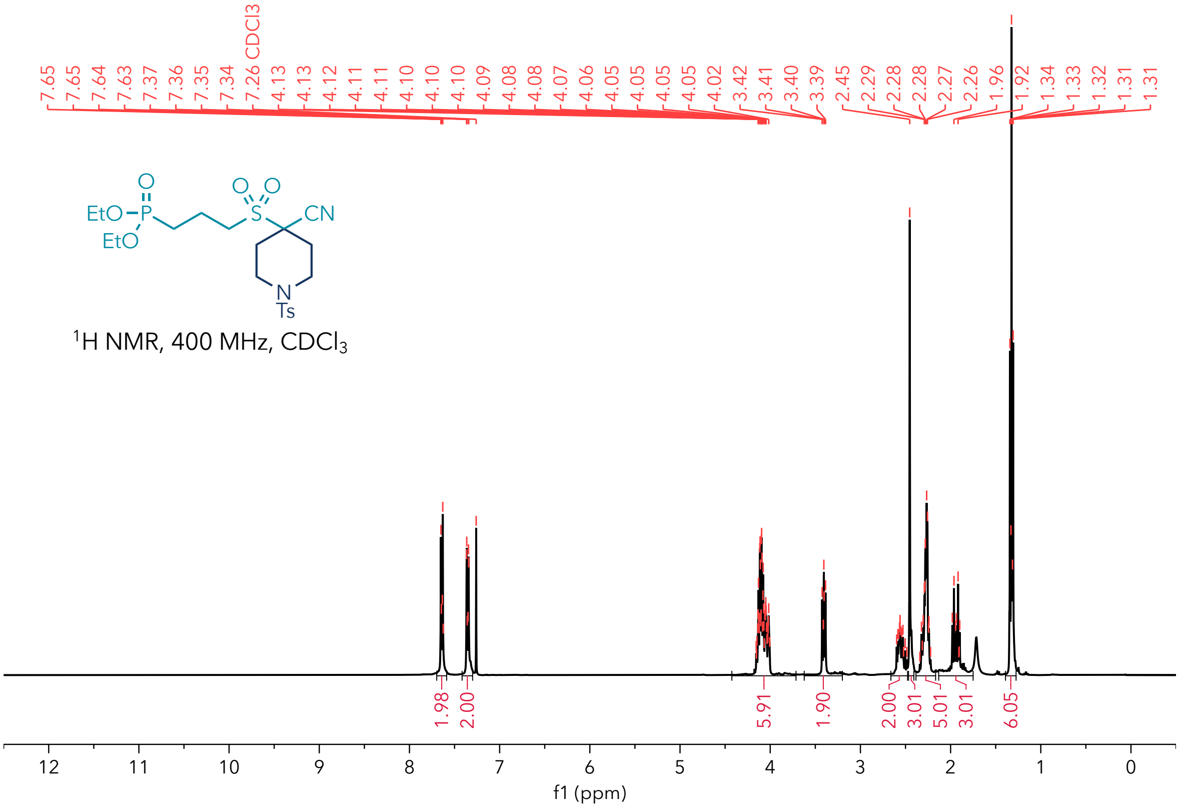
7.65 7.65 7.64 7.63 7.37 7.36 7.35 7.34 7.26 CDCl3 4.13 4.13 4.12 4.11 4.11 4.10 4.10 4.10 4.09 4.08 4.08 4.07 4.06 4.05 4.05 4.05 4.05 4.02 3.42 3.41 3.40 3.39 2.45 2.29 2.28 2.28 2.27 2.26 1.96 1.92 1.34 1.33 1.32 1.31 1.31
EtO
EtO
P
O
S
O O
CN
N
Ts
^{1}H NMR, 400 MHz, CDCl$_3$
1.98 2.00 5.91 1.90 2.00 3.01 5.01 3.01 6.05
12 11 10 9 8 7 6 5 4 3 2 1 0
f1 (ppm)

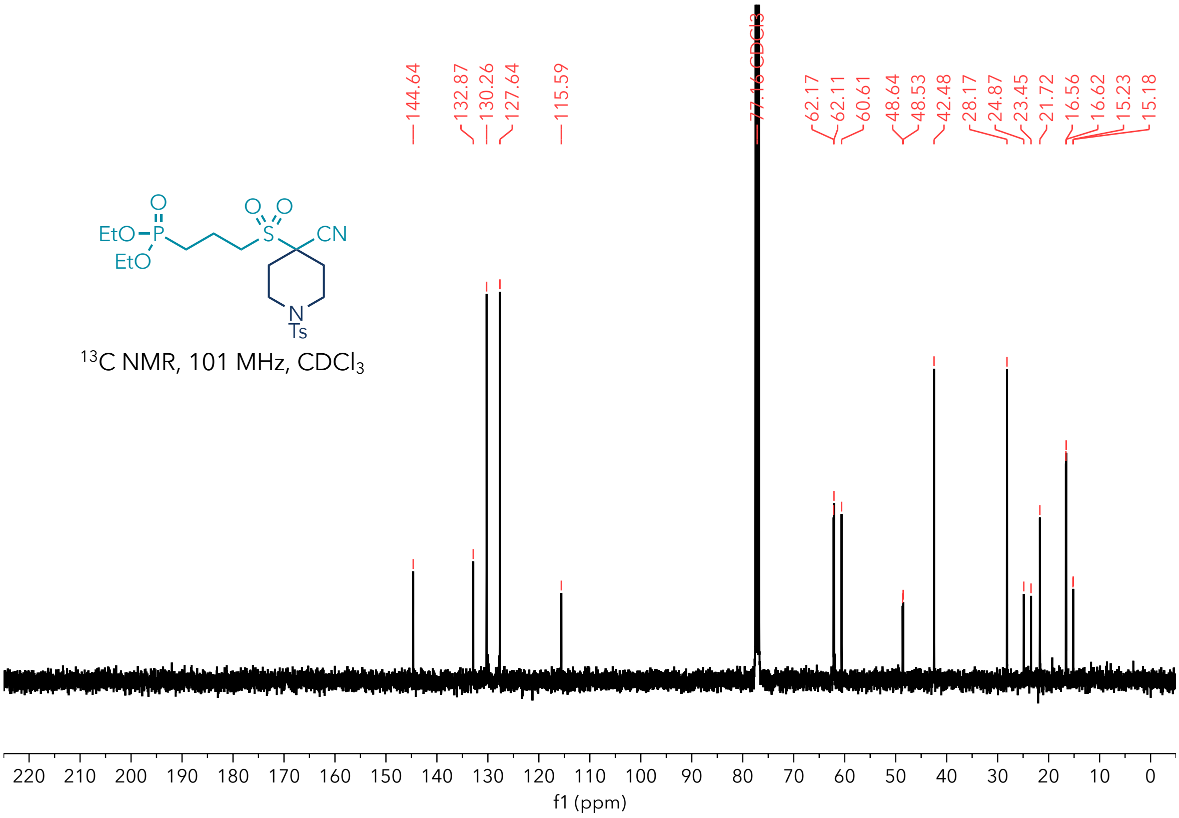
144.64 132.87 130.26 127.64 115.59 77.16 CDCl3 62.17 62.11 60.61 48.64 48.53 42.48 28.17 24.87 23.45 21.72 16.56 16.62 15.23 15.18
EtO
EtO
P
O
S
O O
CN
N
Ts
^{13}C NMR, 101 MHz, CDCl$_3$
220 210 200 190 180 170 160 150 140 130 120 110 100 90 80 70 60 50 40 30 20 10 0
f1 (ppm)

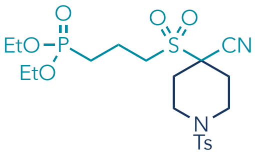

^{31}P NMR, 162 MHz, $CDCl_3$

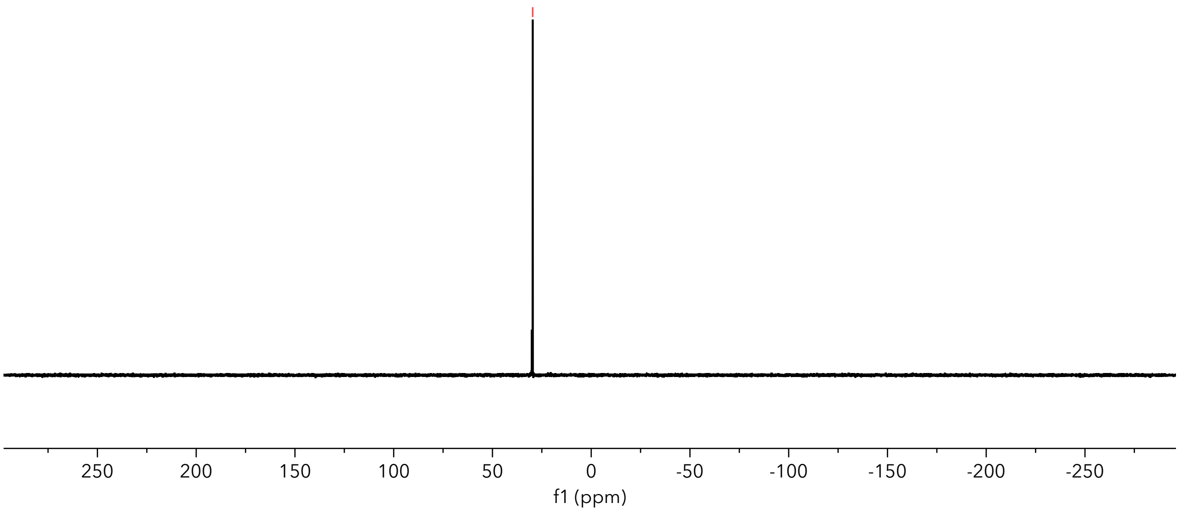

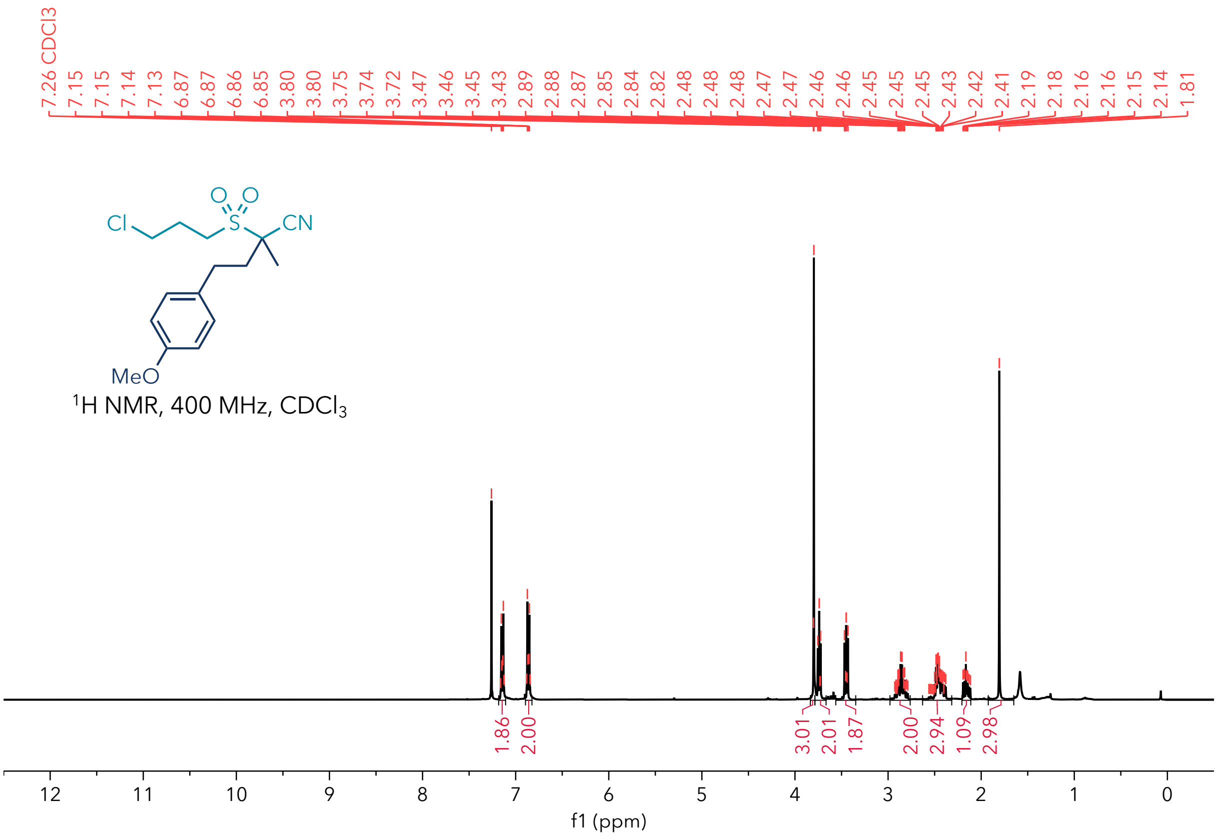

^{1}H NMR, 400 MHz, $CDCl_3$

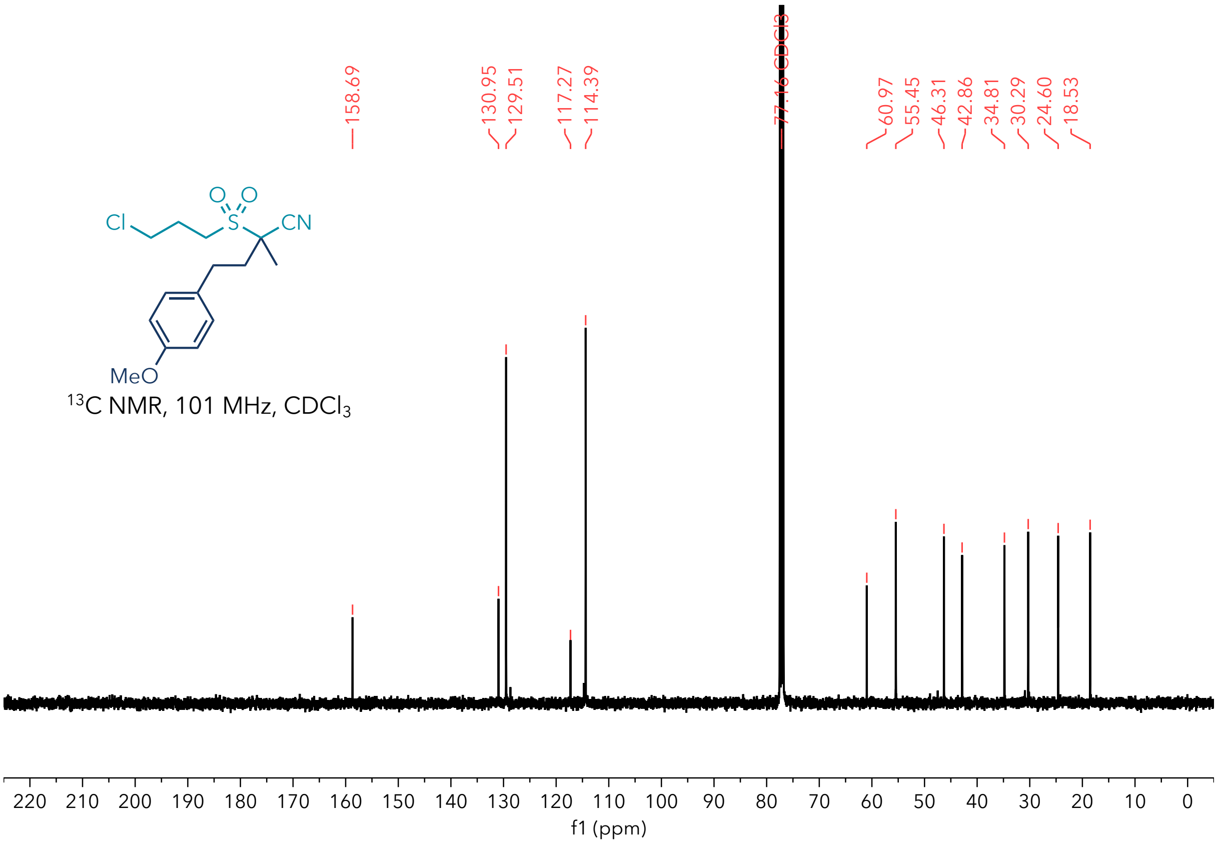

^{13}C NMR, 101 MHz, $CDCl_3$

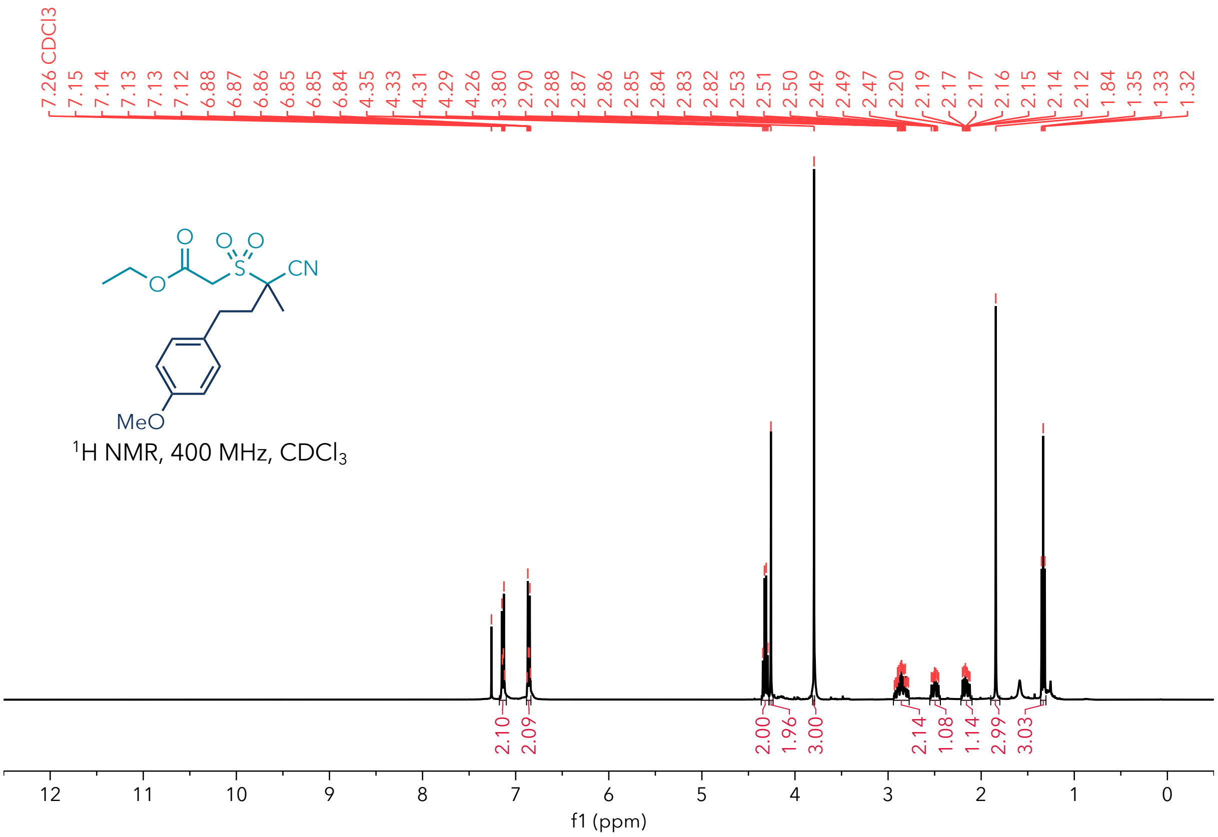

MeO
^{1}H NMR, 400 MHz, CDCl$_3$
f1 (ppm)

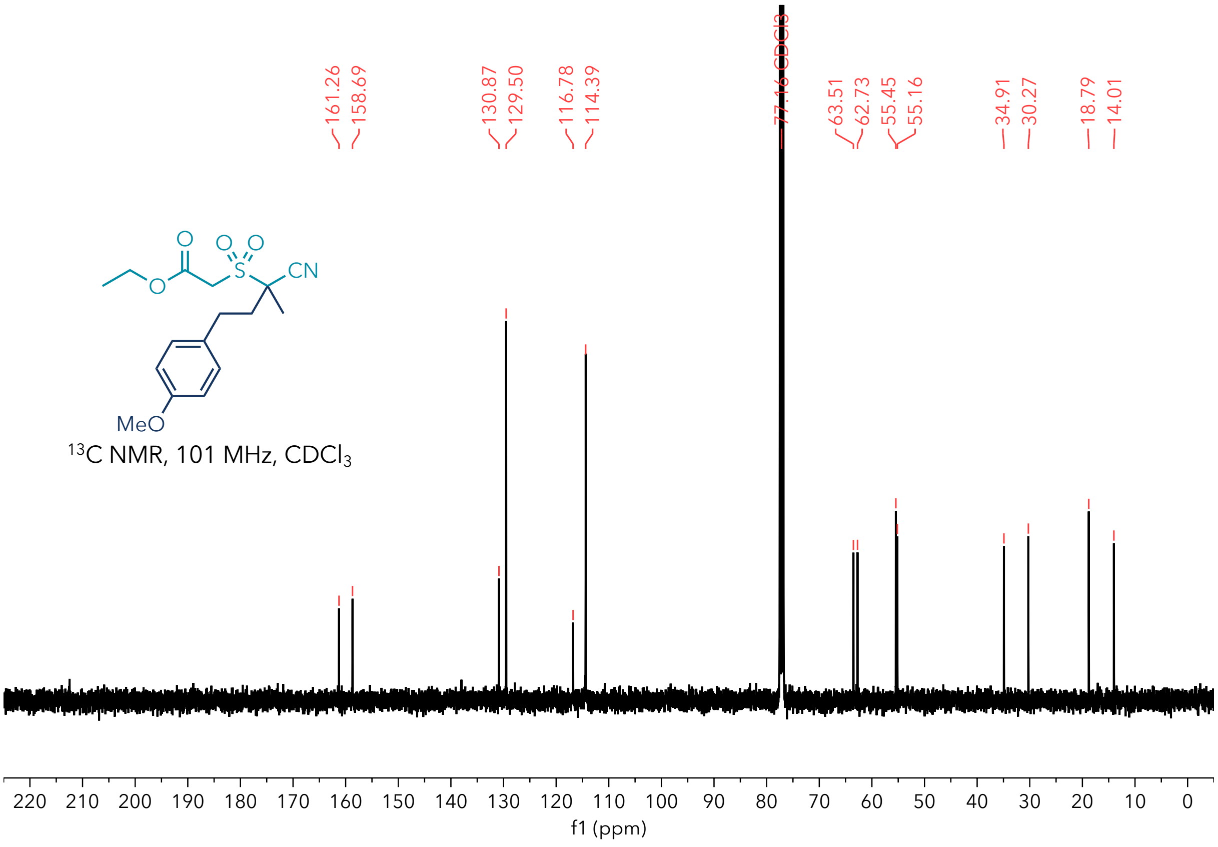

MeO
^{13}C NMR, 101 MHz, CDCl$_3$
f1 (ppm)

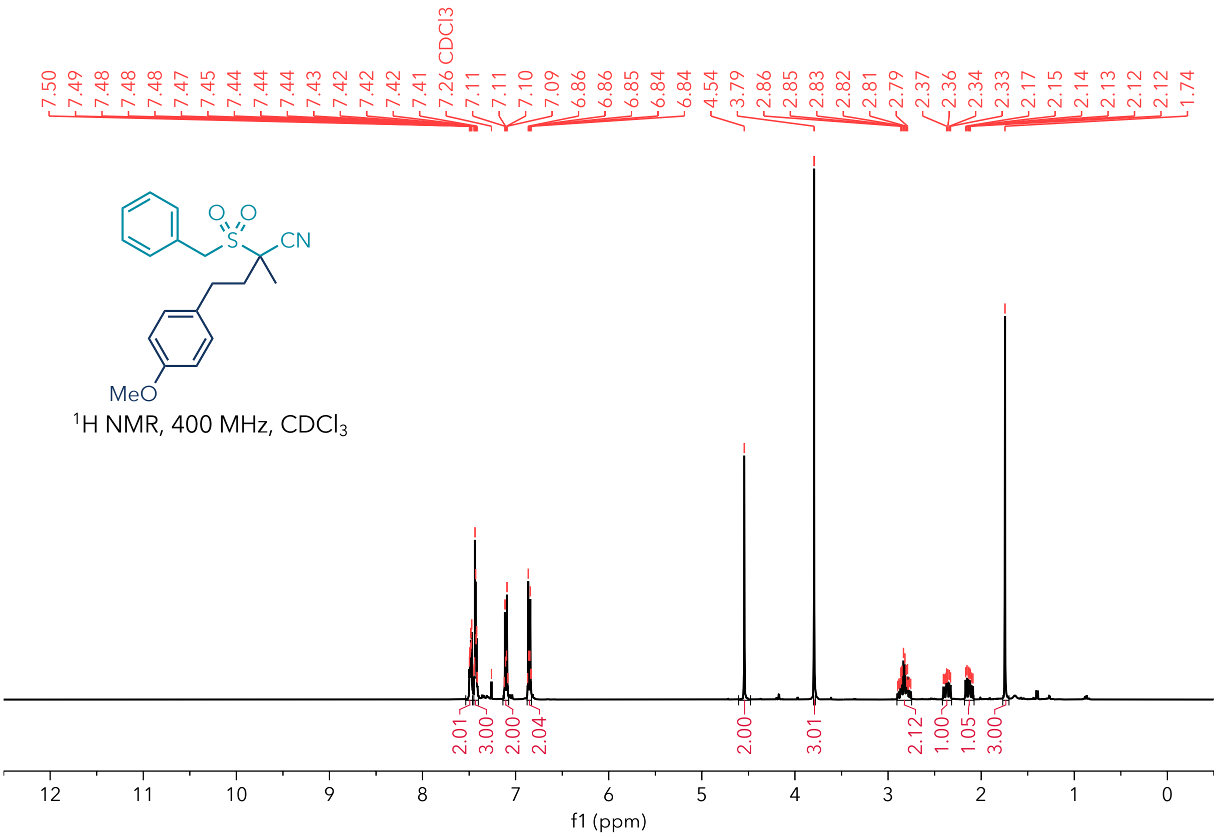
7.50
7.49
7.48
7.48
7.48
7.47
7.45
7.44
7.44
7.44
7.43
7.42
7.42
7.42
7.41
7.26 CDCl3
7.11
7.11
7.10
7.09
6.86
6.86
6.85
6.84
6.84
4.54
3.79
2.86
2.85
2.83
2.82
2.81
2.79
2.37
2.36
2.34
2.33
2.17
2.15
2.14
2.13
2.12
2.12
1.74
CN
MeO
^{1}H NMR, 400 MHz, $CDCl_3$
2.01
3.00
2.00
2.04
2.00
3.01
2.12
1.00
1.05
3.00
f1 (ppm)

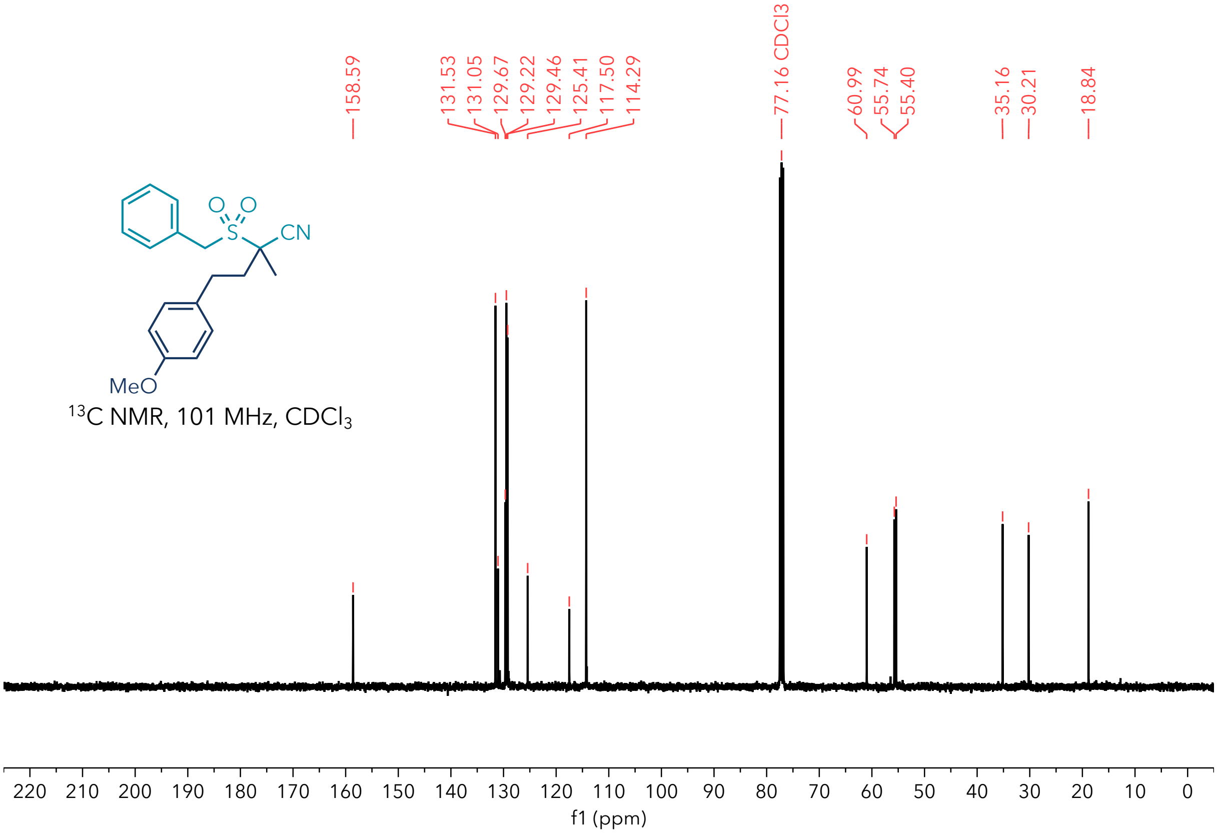
158.59
131.53
131.05
129.67
129.22
129.46
125.41
117.50
114.29
77.16 CDCl3
60.99
55.74
55.40
35.16
30.21
18.84
CN
MeO
^{13}C NMR, 101 MHz, $CDCl_3$
f1 (ppm)

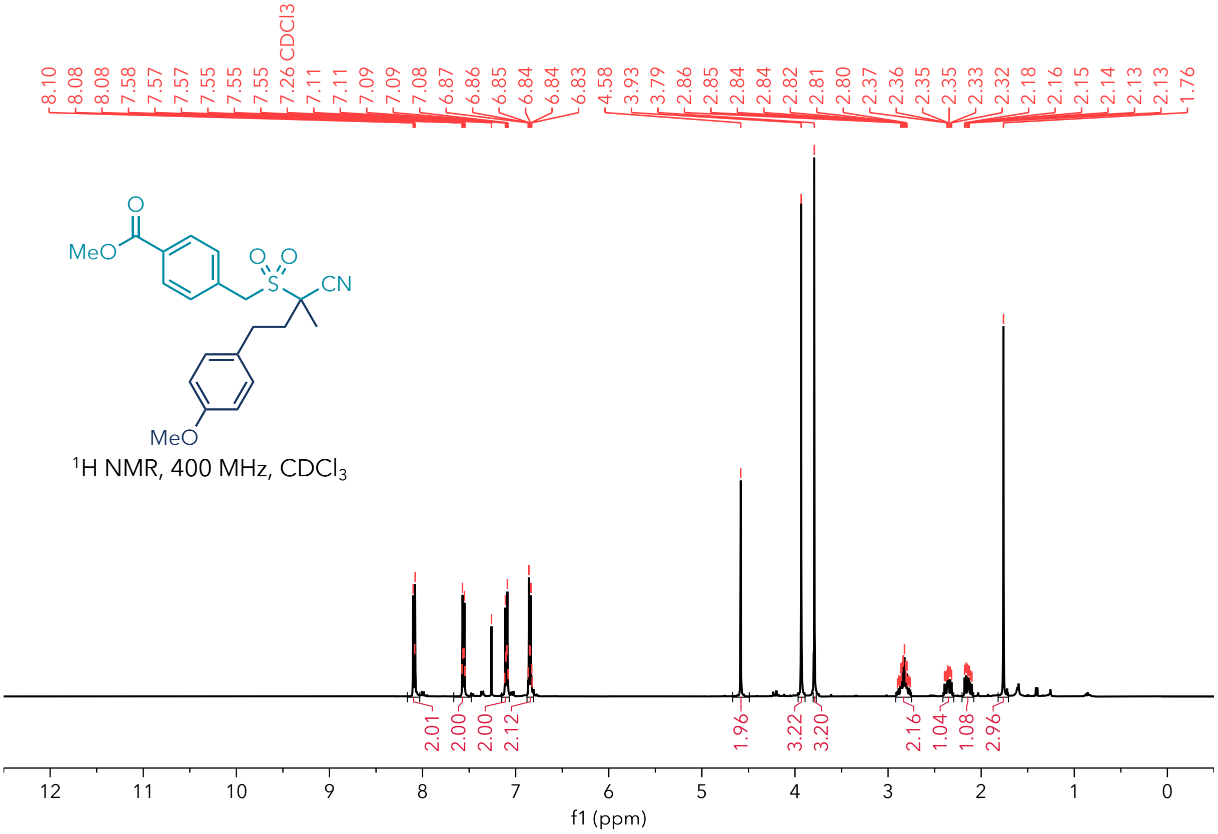
MeO
O
O
O
S
CN
MeO
^{1}H NMR, 400 MHz, CDCl$_3$
8.10 8.08 8.08 7.58 7.57 7.57 7.55 7.55 7.55 7.26 CDCl3 7.11 7.11 7.09 7.09 7.08 6.87 6.86 6.85 6.84 6.84 6.83 4.58 3.93 3.79 2.86 2.85 2.84 2.84 2.82 2.81 2.80 2.37 2.36 2.35 2.35 2.33 2.32 2.18 2.16 2.15 2.14 2.13 2.13 1.76
2.01 2.00 2.00 2.12 1.96 3.22 3.20 2.16 1.04 1.08 2.96
f1 (ppm)

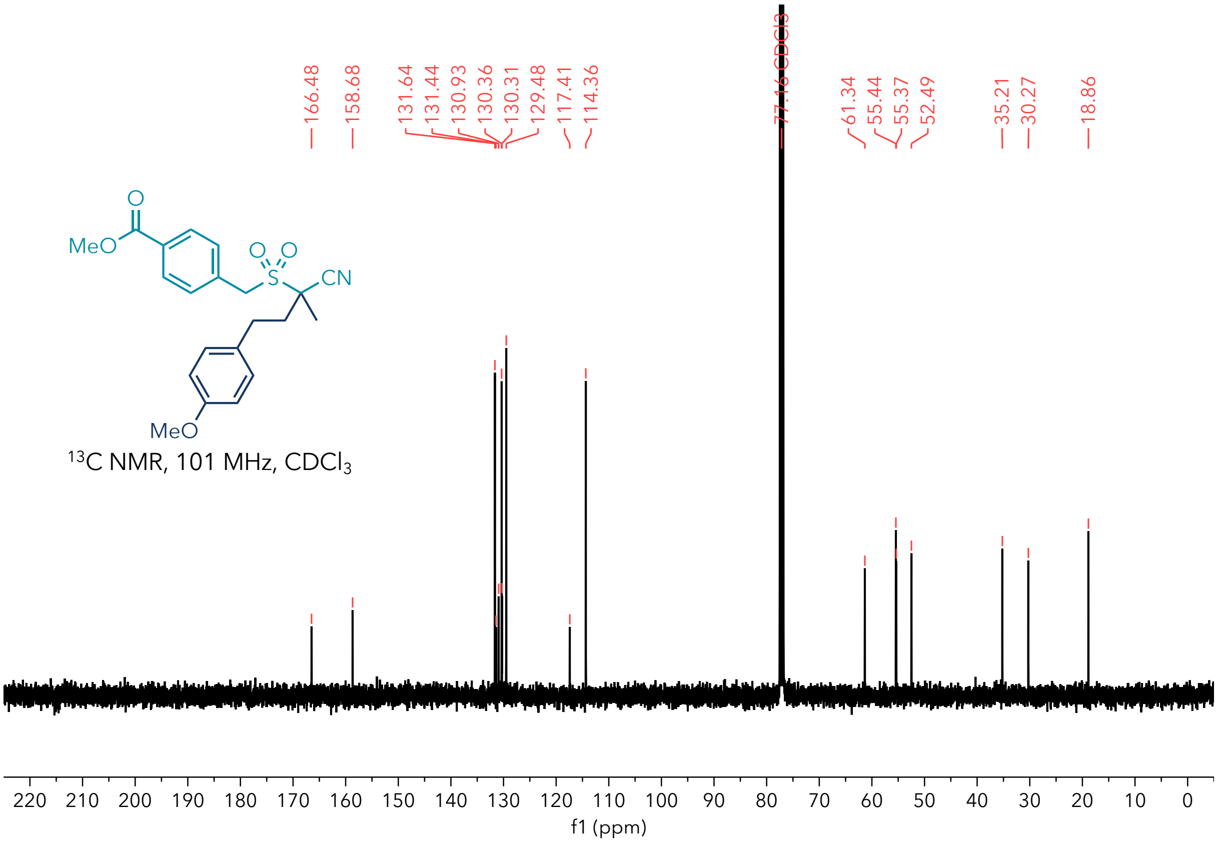
166.48 158.68 131.64 131.44 130.93 130.36 130.31 129.48 117.41 114.36 77.16 CDCl3 61.34 55.44 55.37 52.49 35.21 30.27 18.86
MeO
O
O
O
S
CN
MeO
^{13}C NMR, 101 MHz, CDCl$_3$
f1 (ppm)

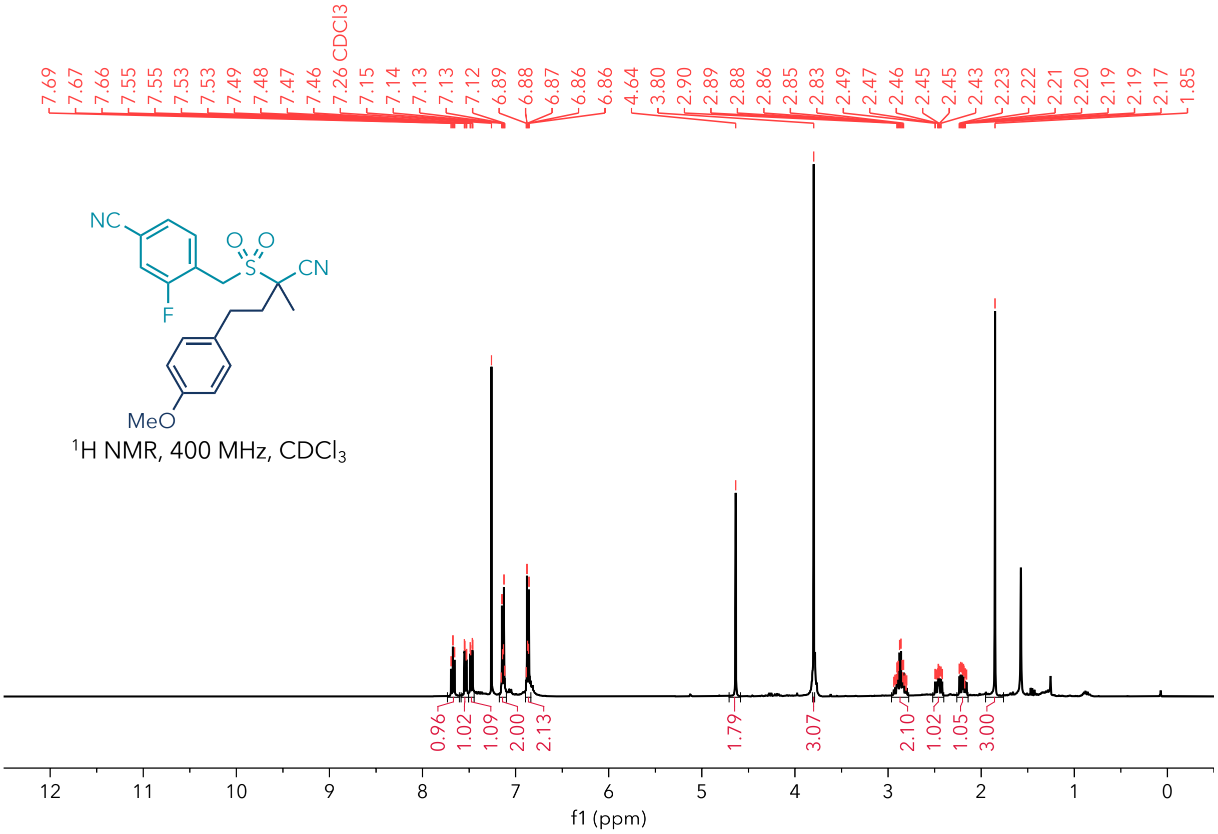

^{1}H NMR, 400 MHz, $CDCl_3$

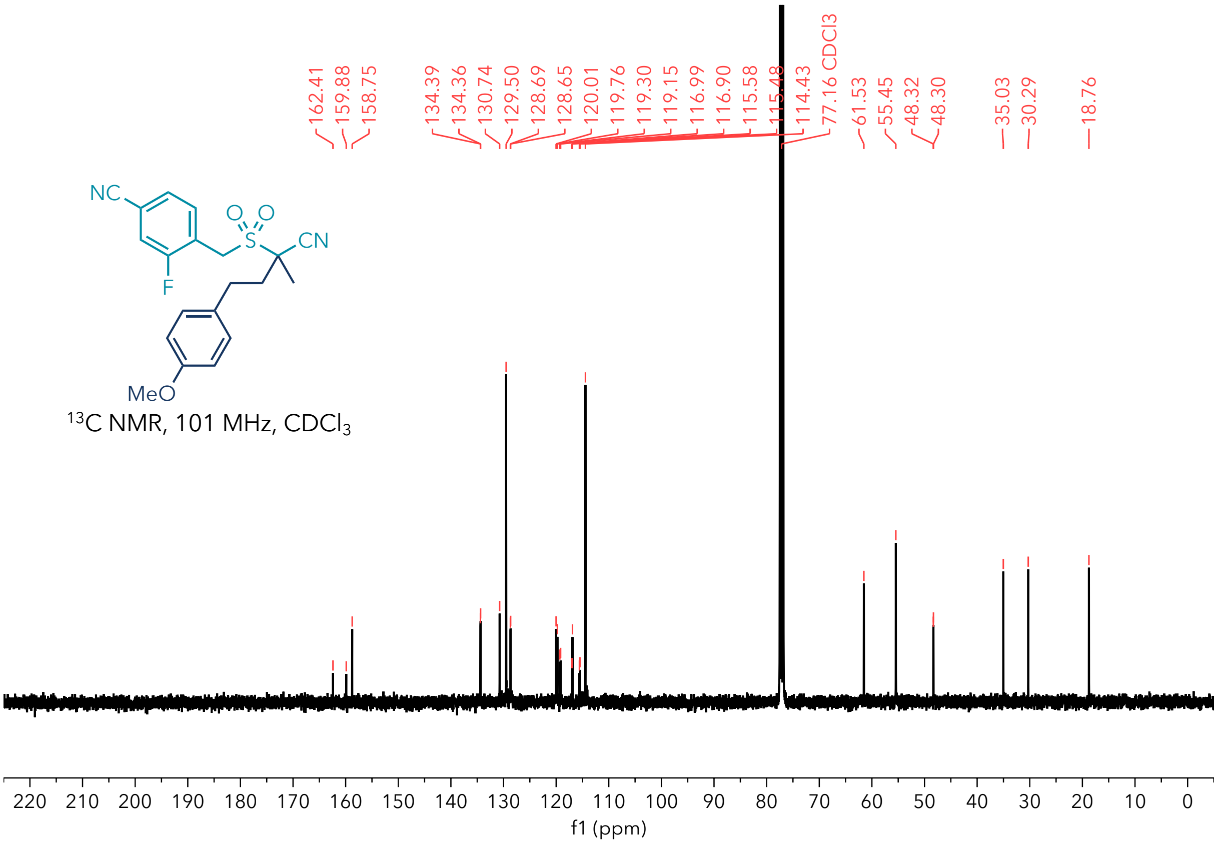

^{13}C NMR, 101 MHz, $CDCl_3$

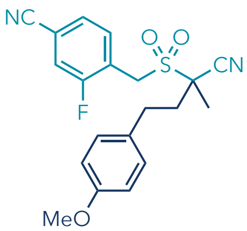

^{19}F NMR, 376 MHz, $CDCl_3$

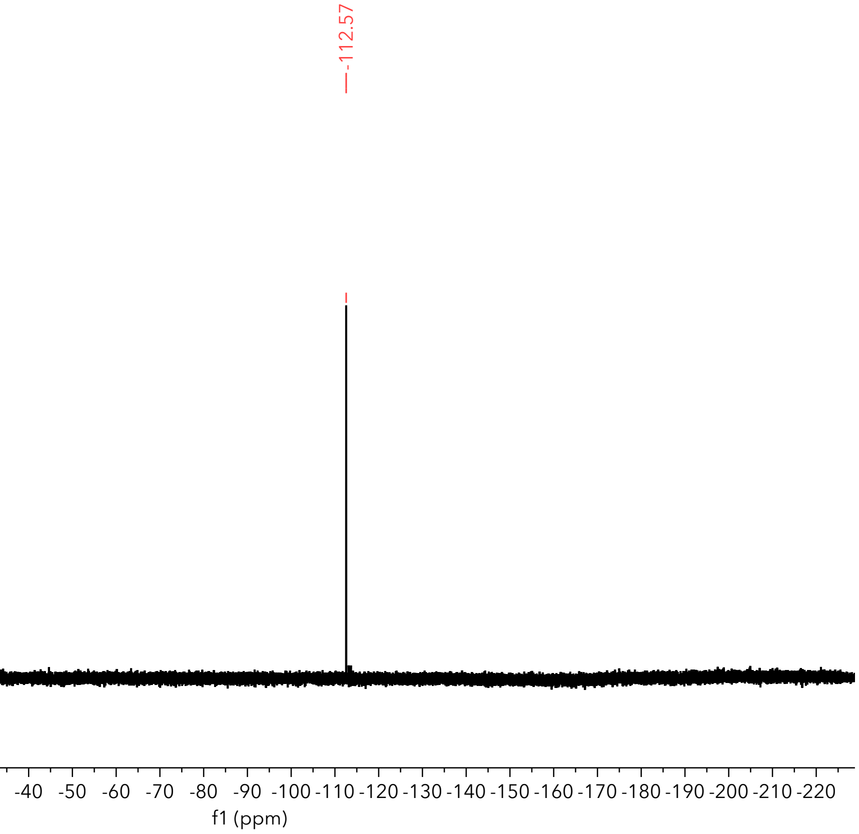

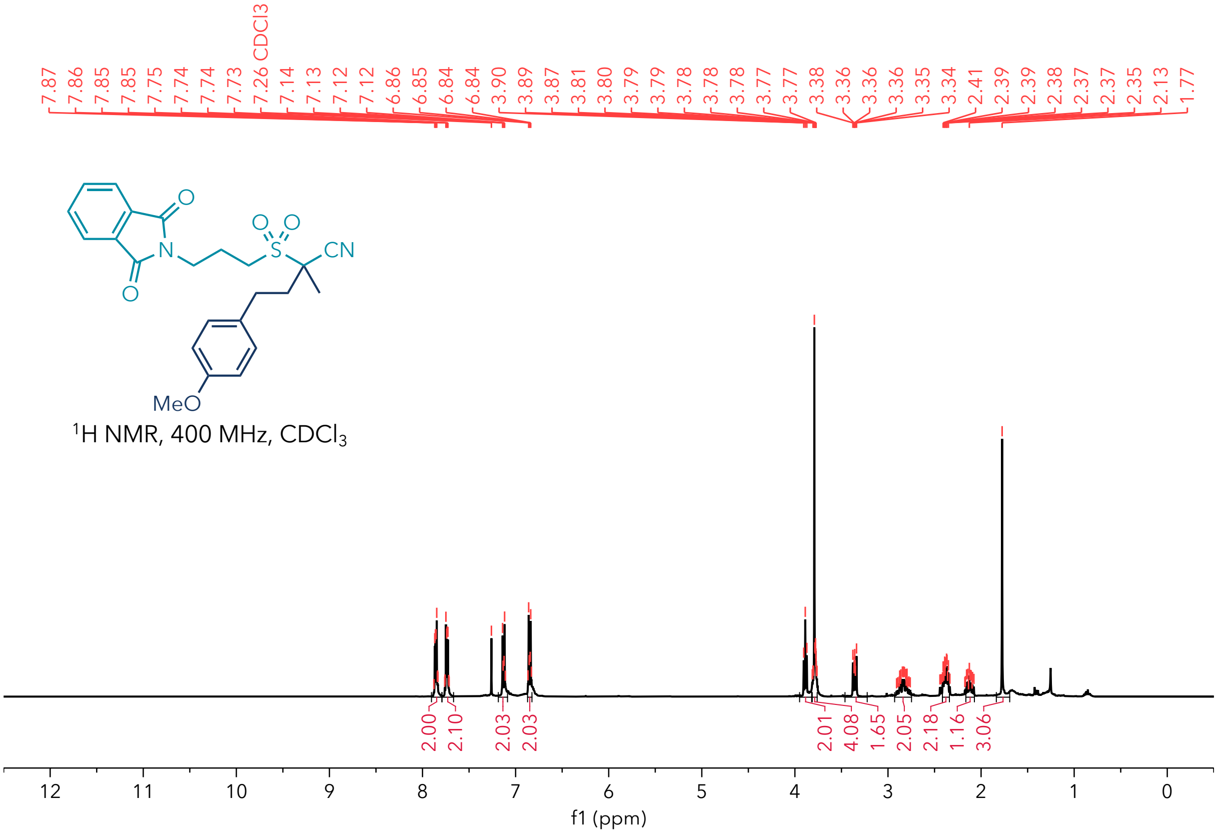
7.87 7.86 7.85 7.85 7.75 7.74 7.74 7.73 7.26 CDCl3 7.14 7.13 7.12 7.12 6.86 6.85 6.84 6.84 3.90 3.89 3.87 3.81 3.80 3.79 3.79 3.78 3.78 3.78 3.77 3.77 3.38 3.36 3.36 3.36 3.35 3.34 2.41 2.39 2.39 2.38 2.37 2.37 2.35 2.13 1.77
O
N
S
CN
MeO
^{1}H NMR, 400 MHz, $CDCl_3$
2.00 2.10 2.03 2.03 2.01 4.08 1.65 2.05 2.18 1.16 3.06
12 11 10 9 8 7 6 5 4 3 2 1 0
f1 (ppm)

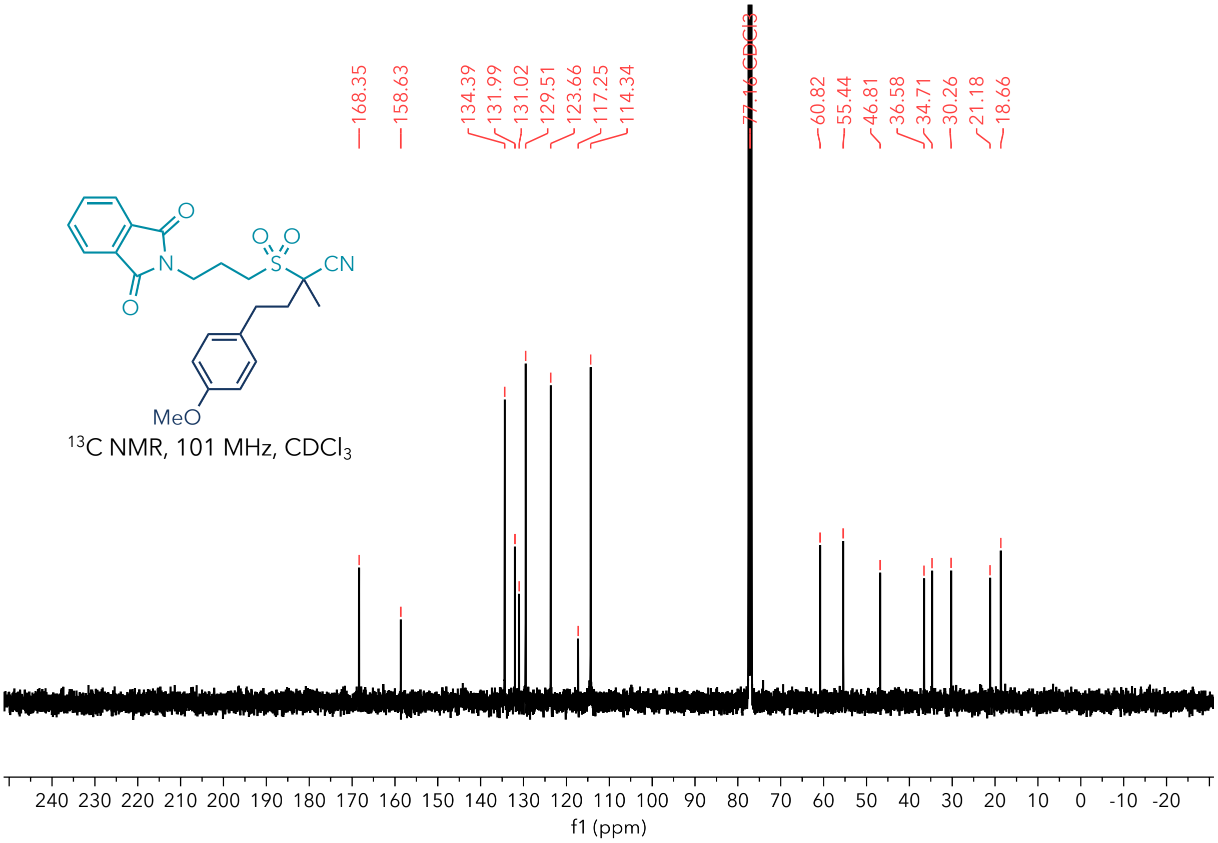
168.35 158.63 134.39 131.99 131.02 129.51 123.66 117.25 114.34 77.16 CDCl3 60.82 55.44 46.81 36.58 34.71 30.26 21.18 18.66
O
N
S
CN
MeO
^{13}C NMR, 101 MHz, $CDCl_3$
240 230 220 210 200 190 180 170 160 150 140 130 120 110 100 90 80 70 60 50 40 30 20 10 0 -10 -20
f1 (ppm)

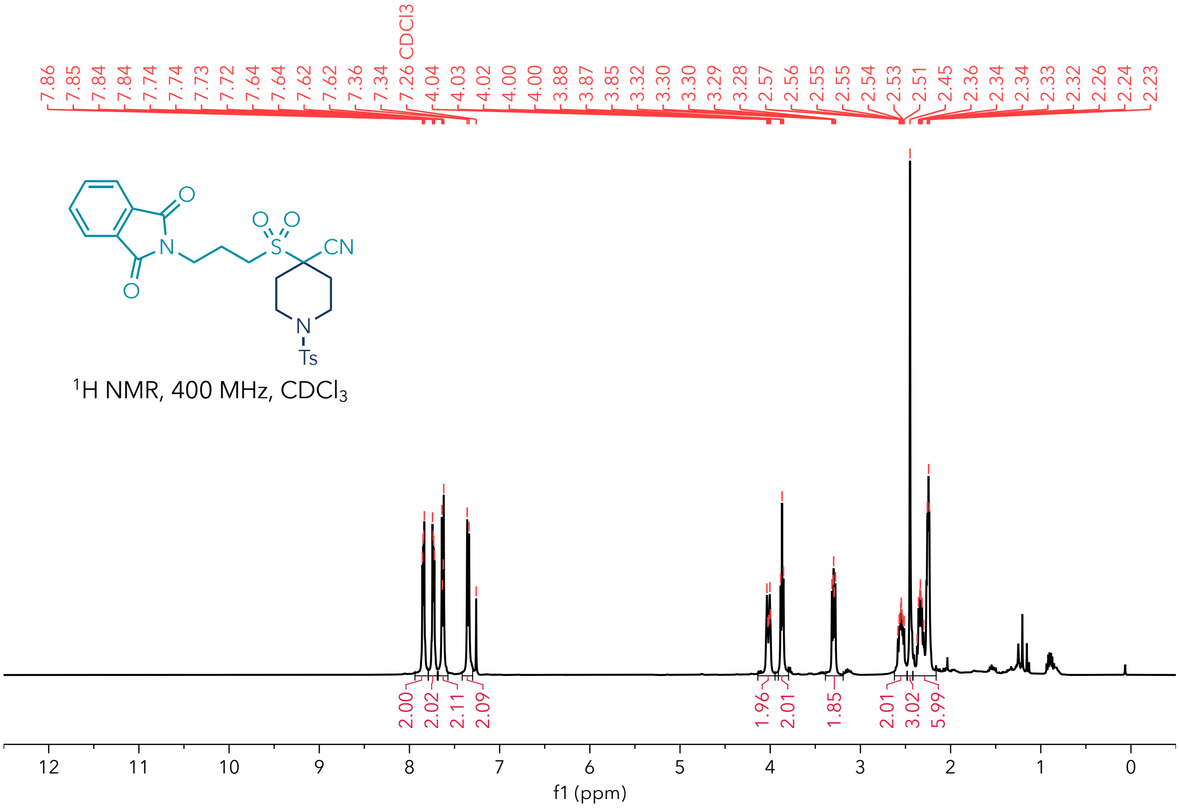
1H NMR, 400 MHz, CDCl3
f1 (ppm)

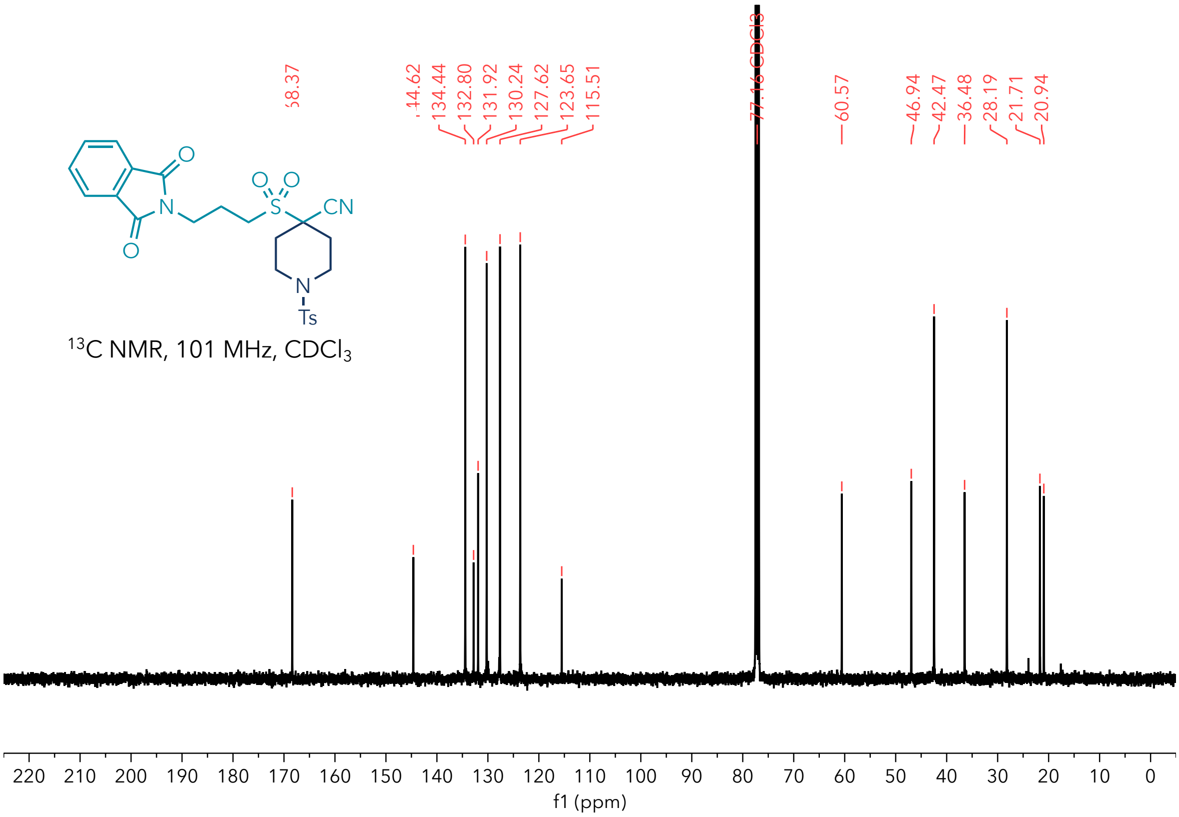
13C NMR, 101 MHz, CDCl3
f1 (ppm)

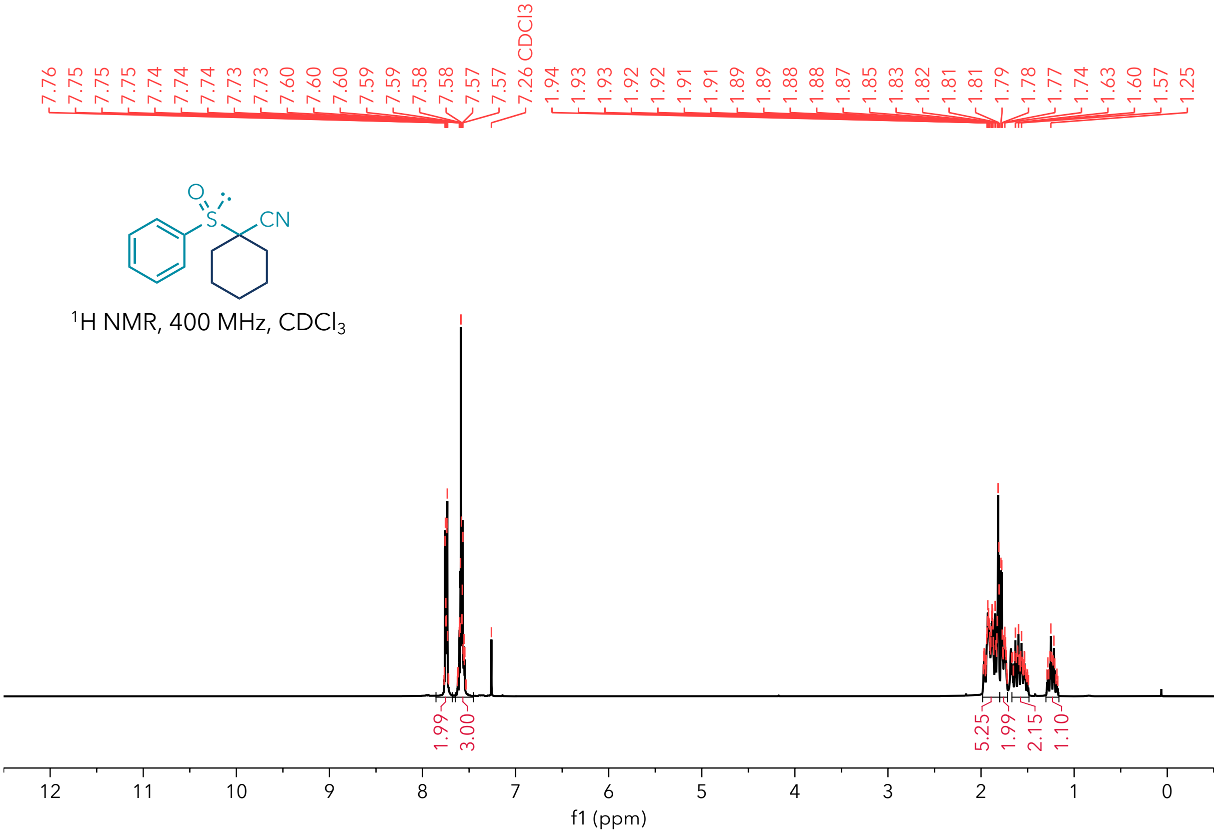
7.76
7.75
7.75
7.75
7.74
7.74
7.74
7.73
7.73
7.60
7.60
7.60
7.59
7.59
7.58
7.58
7.57
7.57
7.26 CDCl3
1.94
1.93
1.93
1.92
1.92
1.91
1.91
1.89
1.89
1.88
1.88
1.87
1.85
1.83
1.82
1.81
1.81
1.79
1.78
1.77
1.74
1.63
1.60
1.57
1.25
CN
O
S
1H NMR, 400 MHz, CDCl3
1.99
3.00
5.25
1.99
2.15
1.10
f1 (ppm)

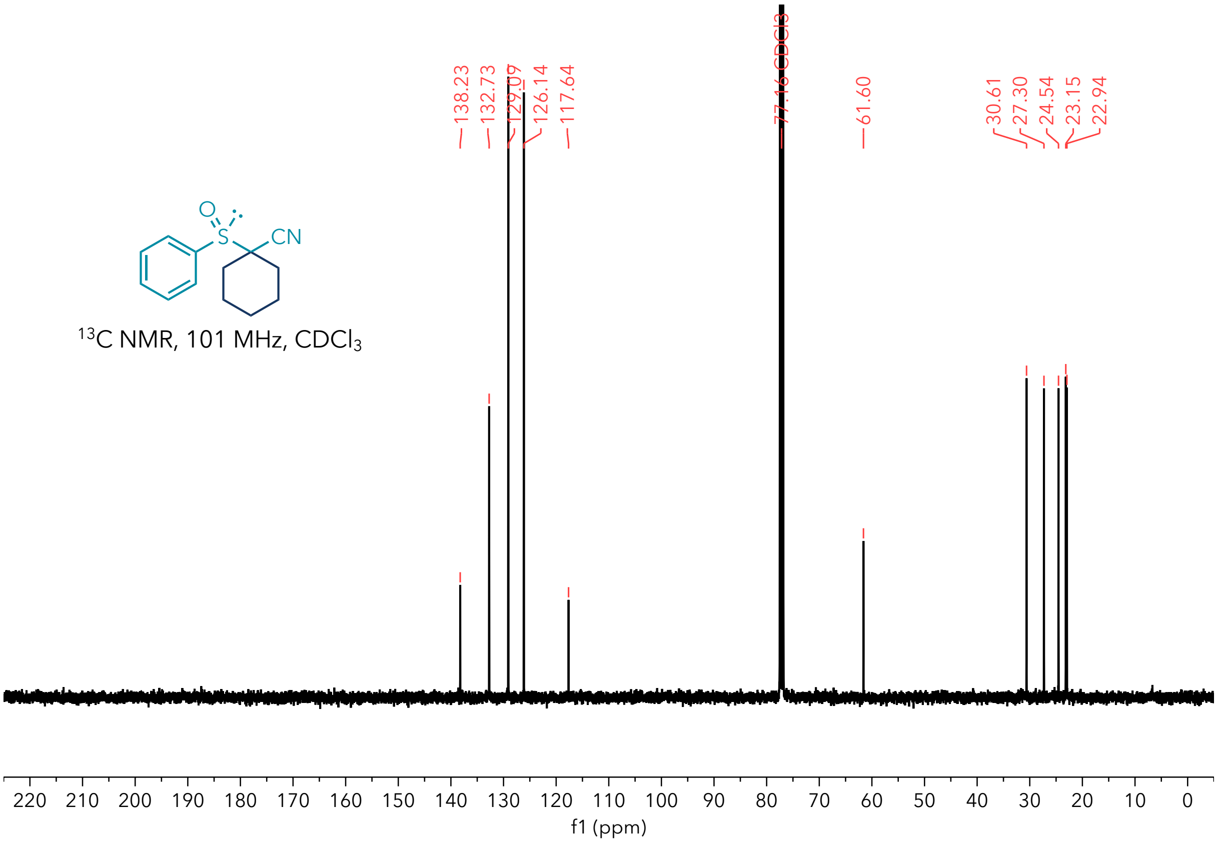
138.23
132.73
129.07
126.14
117.64
77.16 CDCl3
61.60
30.61
27.30
24.54
23.15
22.94
CN
O
S
13C NMR, 101 MHz, CDCl3
f1 (ppm)

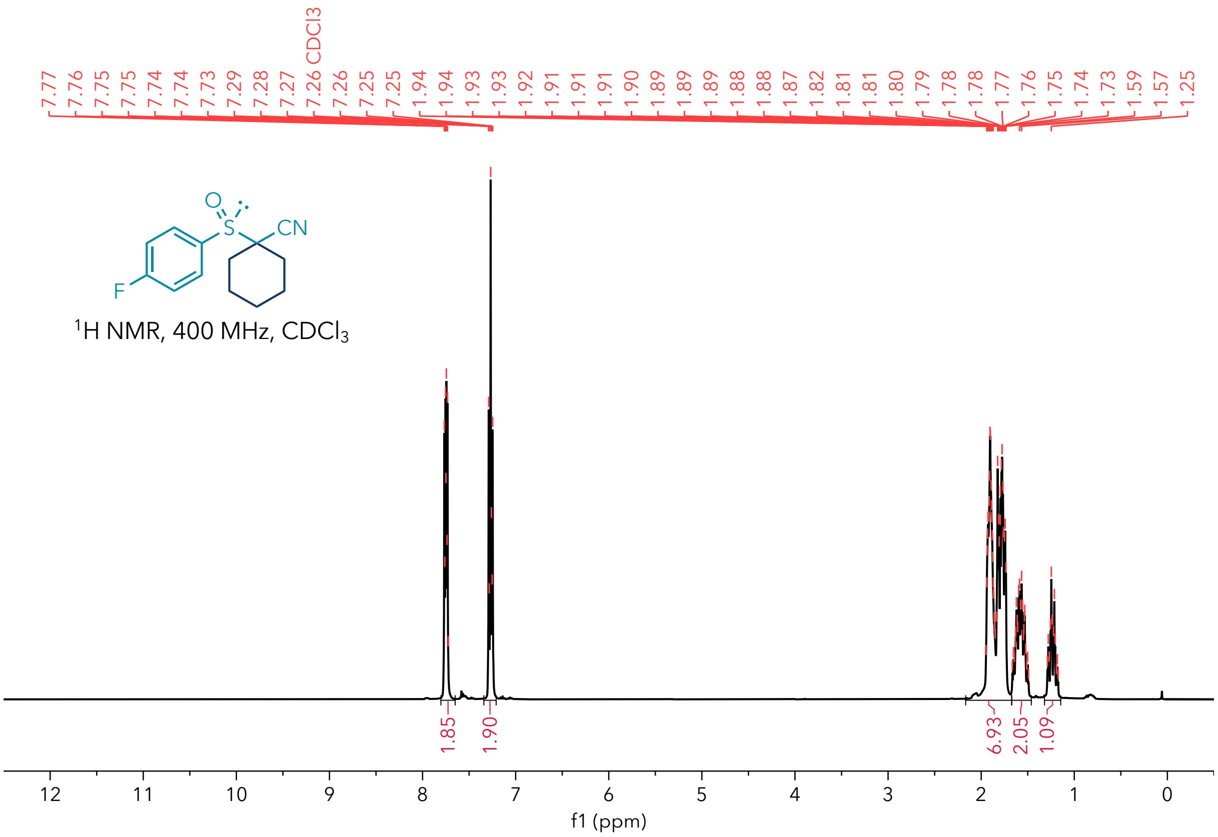

7.77
7.76
7.75
7.75
7.74
7.74
7.73
7.29
7.28
7.27
7.26 CDCl3
7.26
7.25
7.25
1.94
1.94
1.93
1.93
1.92
1.91
1.91
1.91
1.90
1.89
1.89
1.89
1.88
1.88
1.87
1.82
1.81
1.81
1.80
1.79
1.78
1.78
1.77
1.76
1.75
1.74
1.73
1.59
1.57
1.25
O
S
CN
F
1H NMR, 400 MHz, CDCl3
1.85
1.90
6.93
2.05
1.09
12
11
10
9
8
7
6
5
4
3
2
1
0
f1 (ppm)

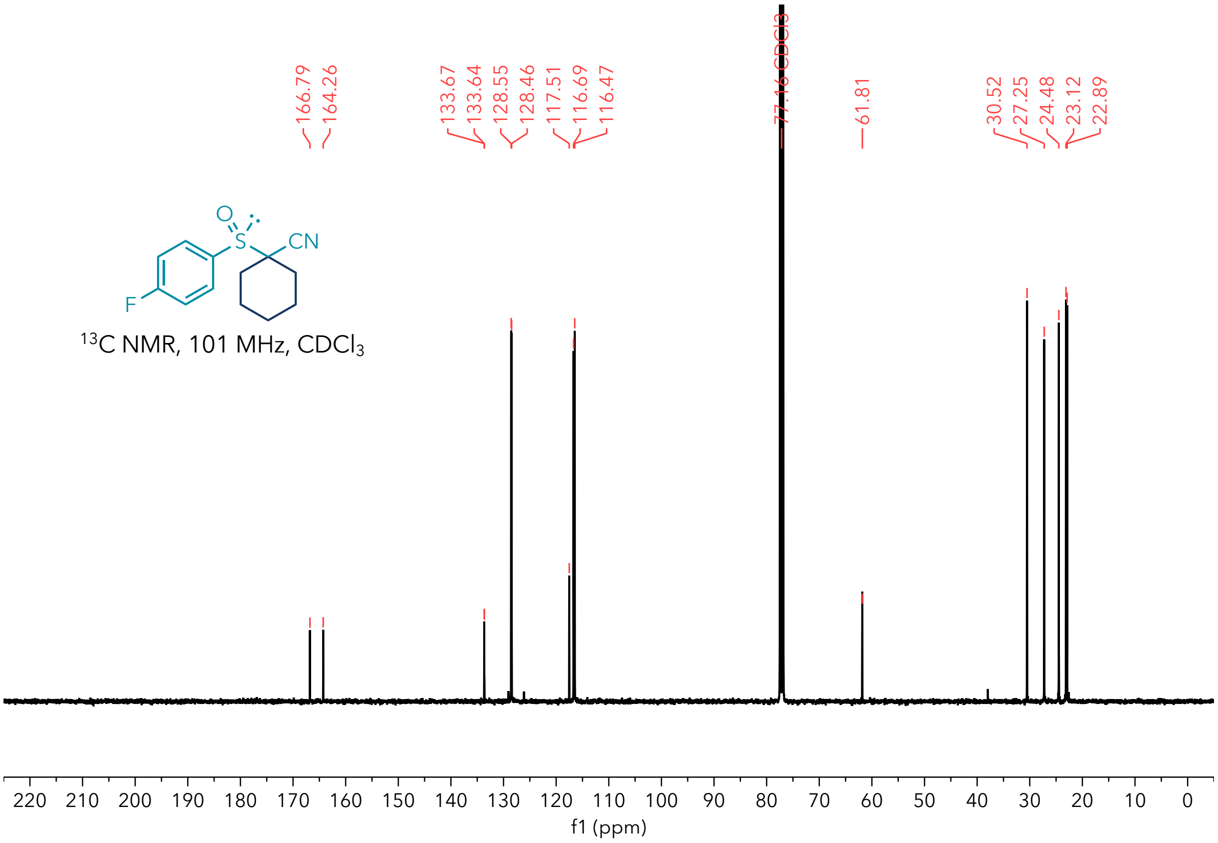

166.79
164.26
133.67
133.64
128.55
128.46
117.51
116.69
116.47
77.16 CDCl3
61.81
30.52
27.25
24.48
23.12
22.89
O
S
CN
F
13C NMR, 101 MHz, CDCl3
220
210
200
190
180
170
160
150
140
130
120
110
100
90
80
70
60
50
40
30
20
10
0
f1 (ppm)

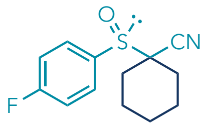

^{19}F NMR, 376 MHz, $CDCl_3$

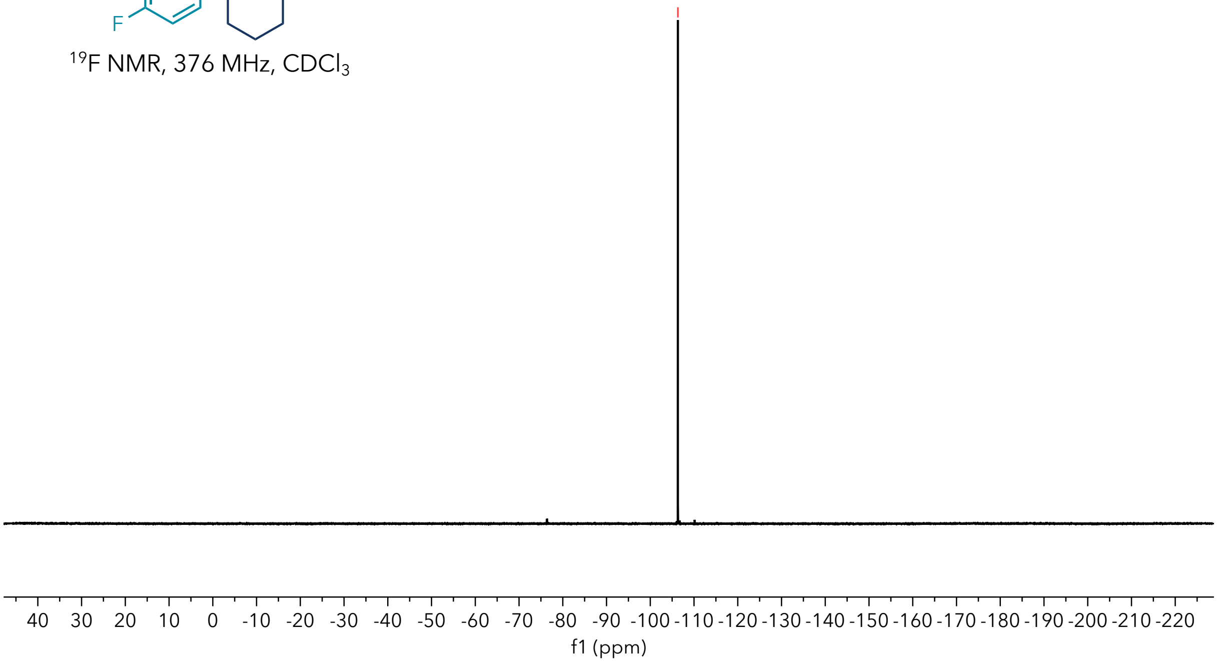

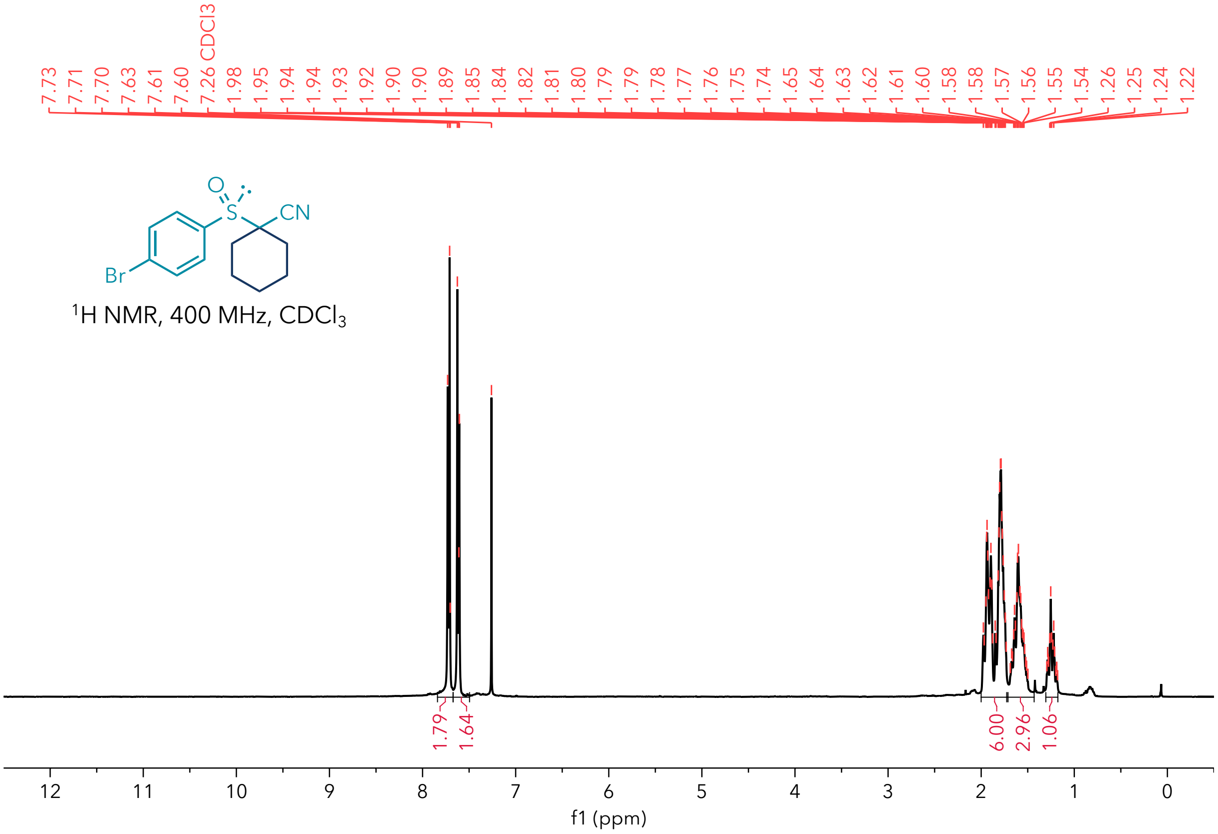
7.73
7.71
7.70
7.63
7.61
7.60
7.26 CDCl3
1.98
1.95
1.94
1.94
1.93
1.92
1.90
1.90
1.89
1.85
1.84
1.82
1.81
1.80
1.79
1.79
1.78
1.77
1.76
1.75
1.74
1.65
1.64
1.63
1.62
1.61
1.60
1.58
1.58
1.57
1.56
1.55
1.54
1.26
1.25
1.24
1.22
Br
O
S
CN
^{1}H NMR, 400 MHz, $CDCl_3$
1.79
1.64
6.00
2.96
1.06
12
11
10
9
8
7
6
5
4
3
2
1
0
f1 (ppm)

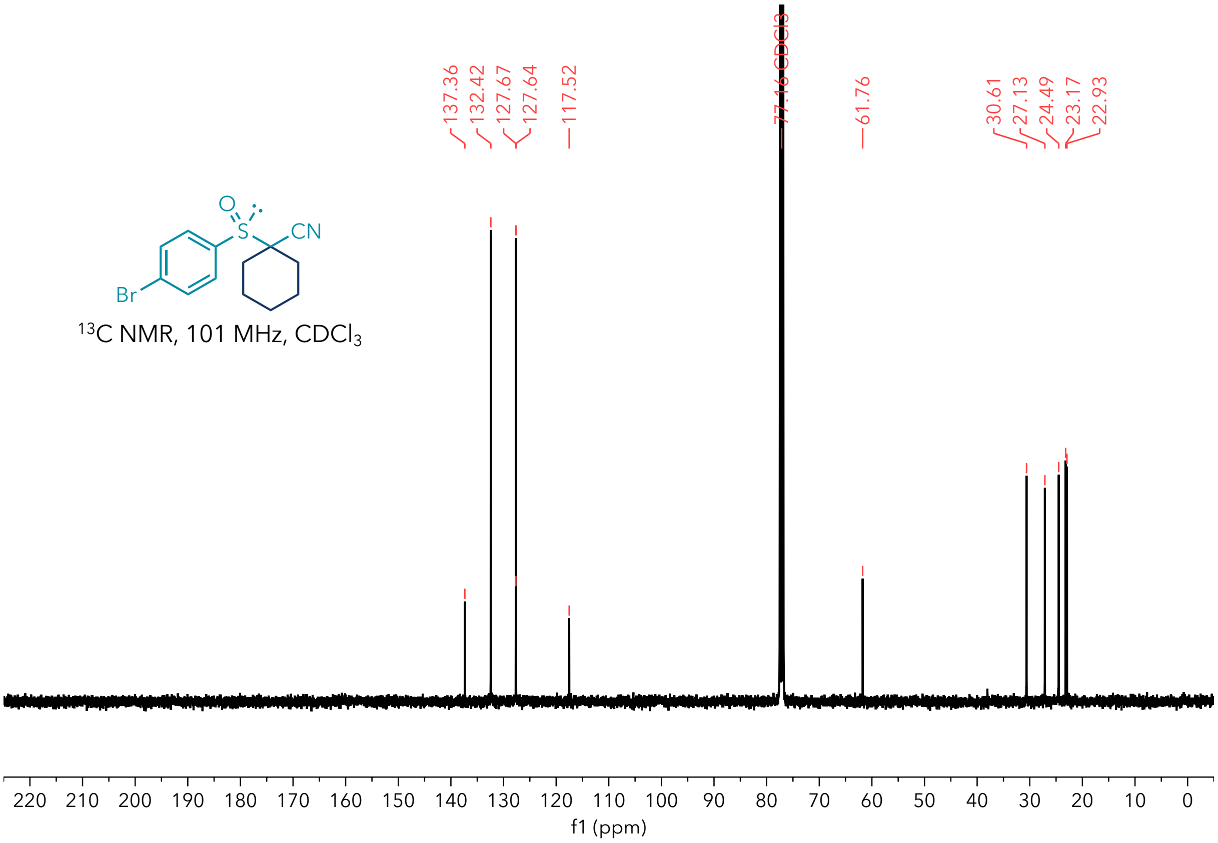
137.36
132.42
127.67
127.64
117.52
77.16 CDCl3
61.76
30.61
27.13
24.49
23.17
22.93
Br
O
S
CN
^{13}C NMR, 101 MHz, $CDCl_3$
220
210
200
190
180
170
160
150
140
130
120
110
100
90
80
70
60
50
40
30
20
10
0
f1 (ppm)

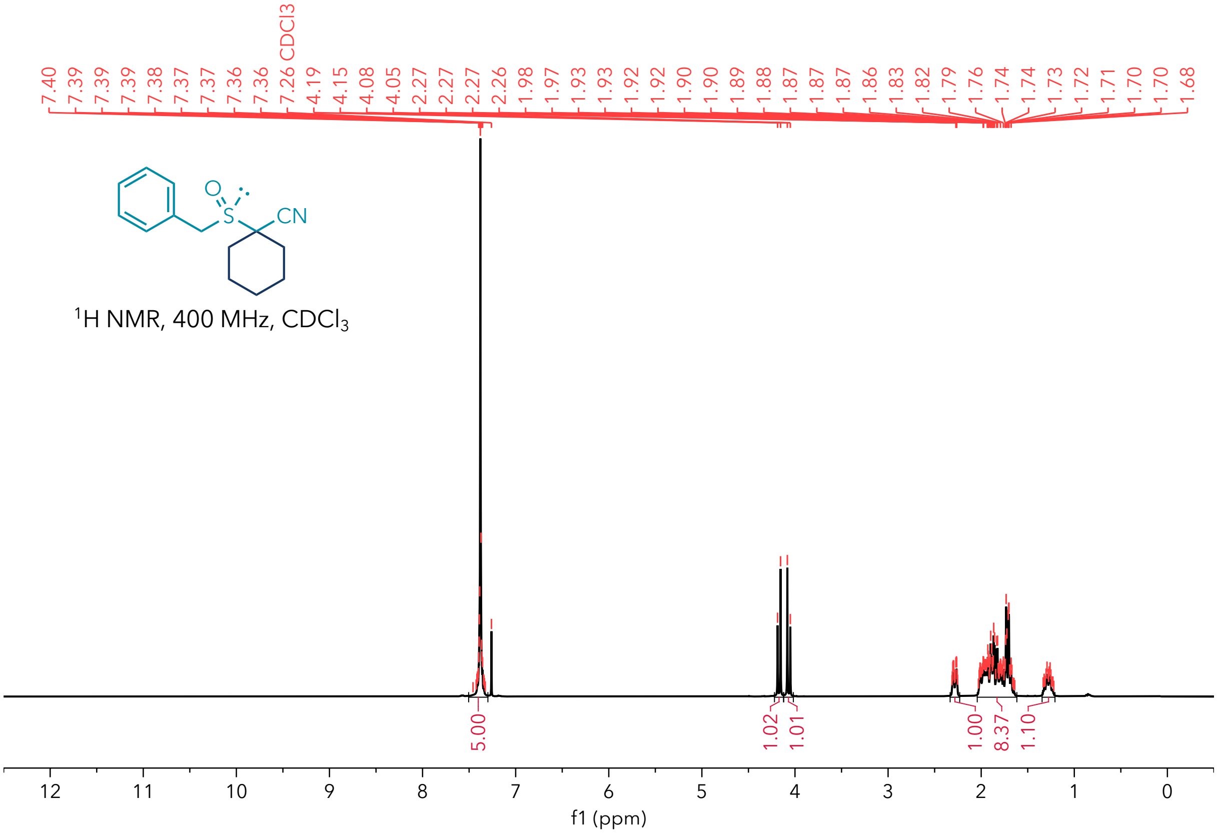

7.40 7.39 7.39 7.39 7.38 7.37 7.37 7.36 7.36 7.26 CDCl3 4.19 4.15 4.08 4.05 2.27 2.27 2.27 2.26 1.98 1.97 1.93 1.93 1.92 1.92 1.90 1.90 1.89 1.88 1.87 1.87 1.87 1.86 1.83 1.82 1.79 1.76 1.74 1.74 1.73 1.72 1.71 1.70 1.70 1.68
O
S
CN
1H NMR, 400 MHz, CDCl3
5.00
1.02
1.01
1.00
8.37
1.10
12 11 10 9 8 7 6 5 4 3 2 1 0
f1 (ppm)

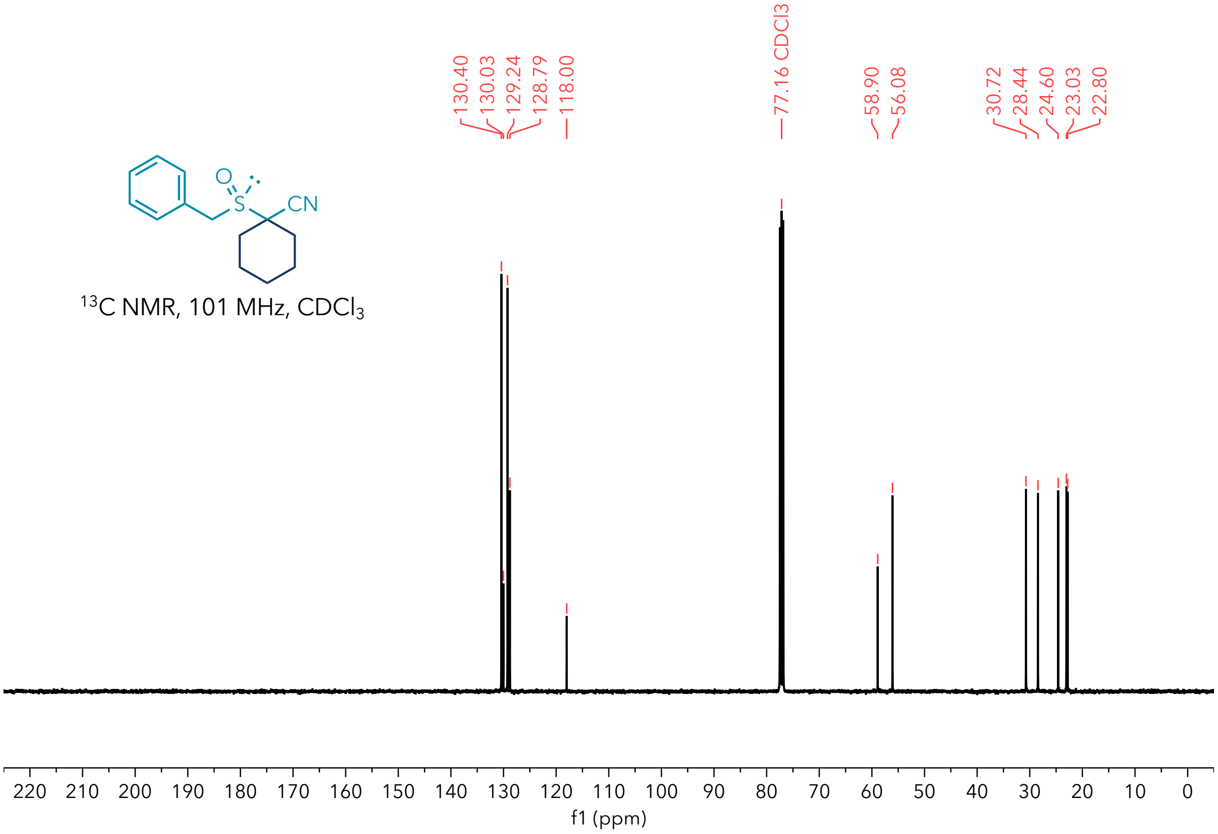

130.40 130.03 129.24 128.79 118.00
77.16 CDCl3
58.90 56.08
30.72 28.44 24.60 23.03 22.80
O
S
CN
13C NMR, 101 MHz, CDCl3
220 210 200 190 180 170 160 150 140 130 120 110 100 90 80 70 60 50 40 30 20 10 0
f1 (ppm)

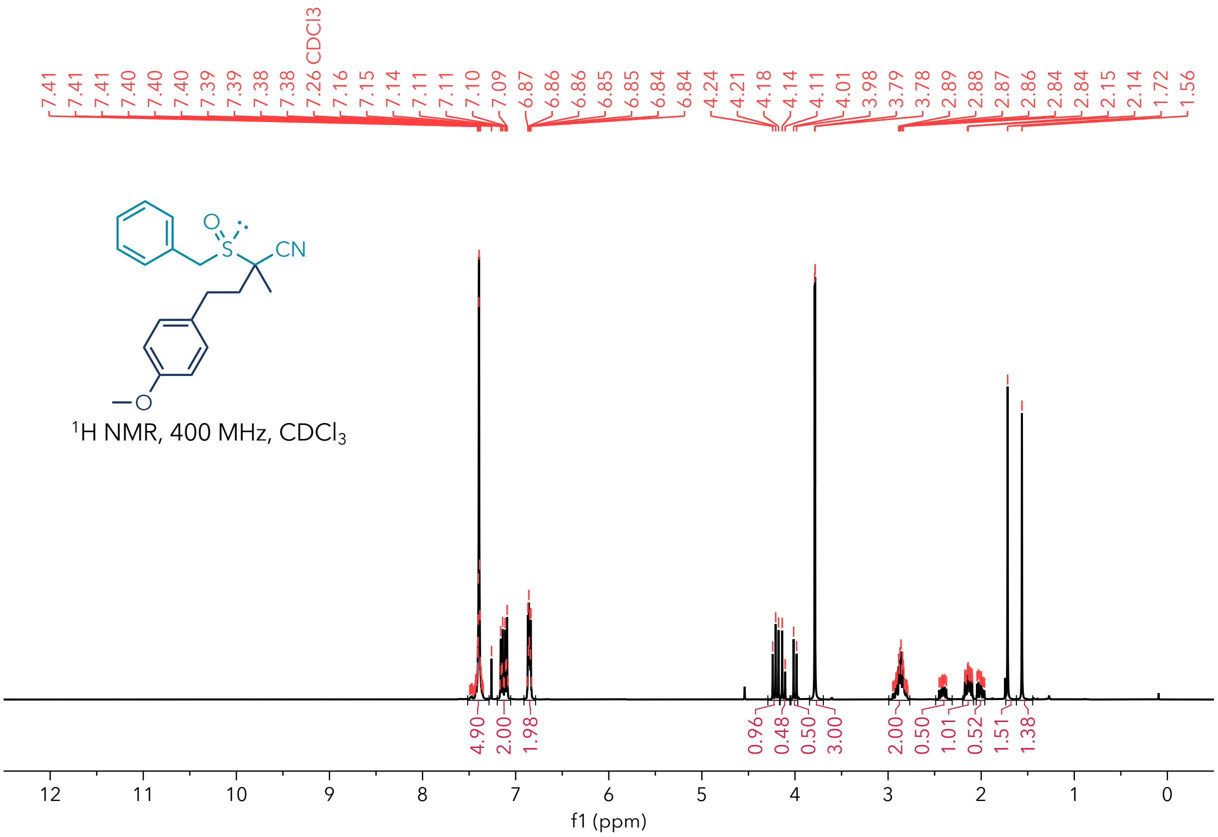

7.41
7.41
7.41
7.40
7.40
7.40
7.39
7.39
7.38
7.38
7.26 CDCl3
7.16
7.15
7.14
7.11
7.11
7.10
7.09
6.87
6.86
6.86
6.85
6.85
6.84
6.84
4.24
4.21
4.18
4.14
4.11
4.01
3.98
3.79
3.78
2.89
2.88
2.87
2.86
2.84
2.84
2.15
2.14
1.72
1.56
CN
O
S
O
^{1}H NMR, 400 MHz, $CDCl_3$
4.90
2.00
1.98
0.96
0.48
0.50
3.00
2.00
0.50
1.01
0.52
1.51
1.38
12
11
10
9
8
7
6
5
4
3
2
1
0
f1 (ppm)

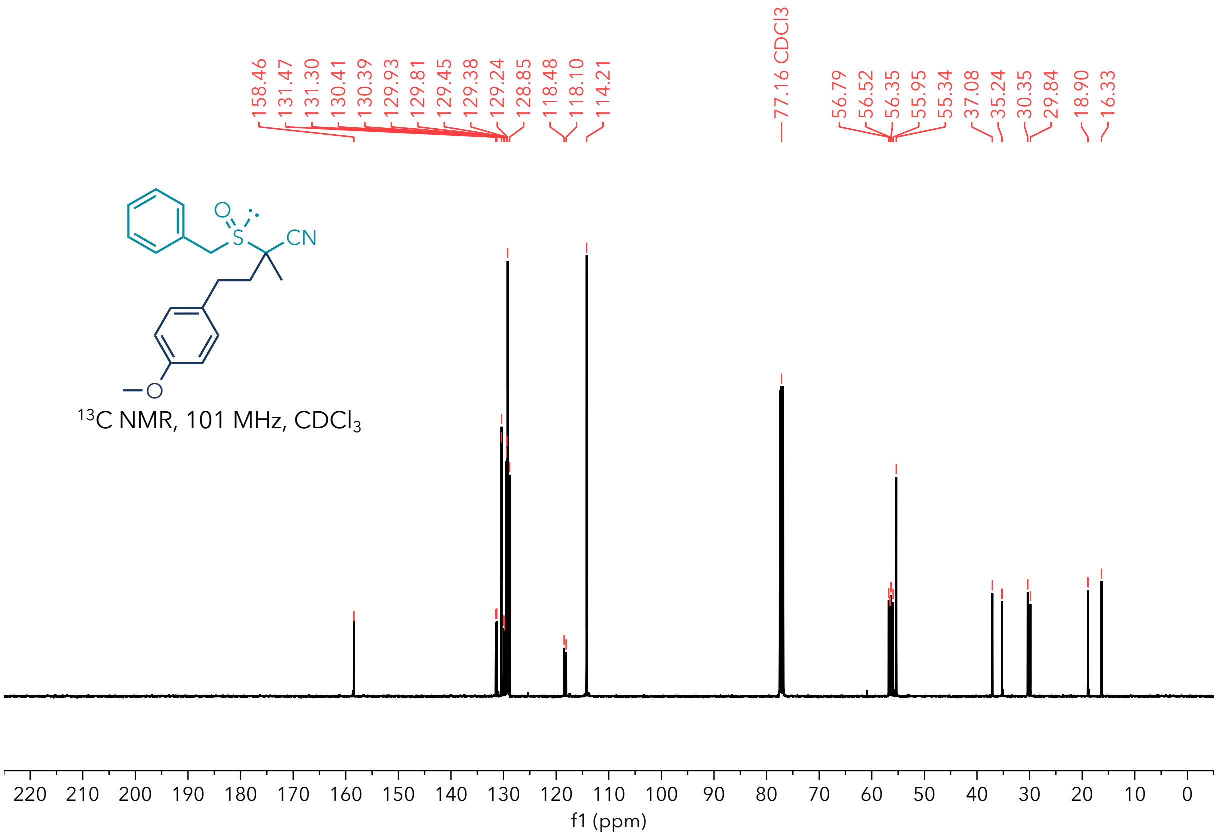

158.46
131.47
131.30
130.41
130.39
129.93
129.81
129.45
129.38
129.24
128.85
118.48
118.10
114.21
77.16 CDCl3
56.79
56.52
56.35
55.95
55.34
37.08
35.24
30.35
29.84
18.90
16.33
CN
O
S
O
^{13}C NMR, 101 MHz, $CDCl_3$
220
210
200
190
180
170
160
150
140
130
120
110
100
90
80
70
60
50
40
30
20
10
0
f1 (ppm)

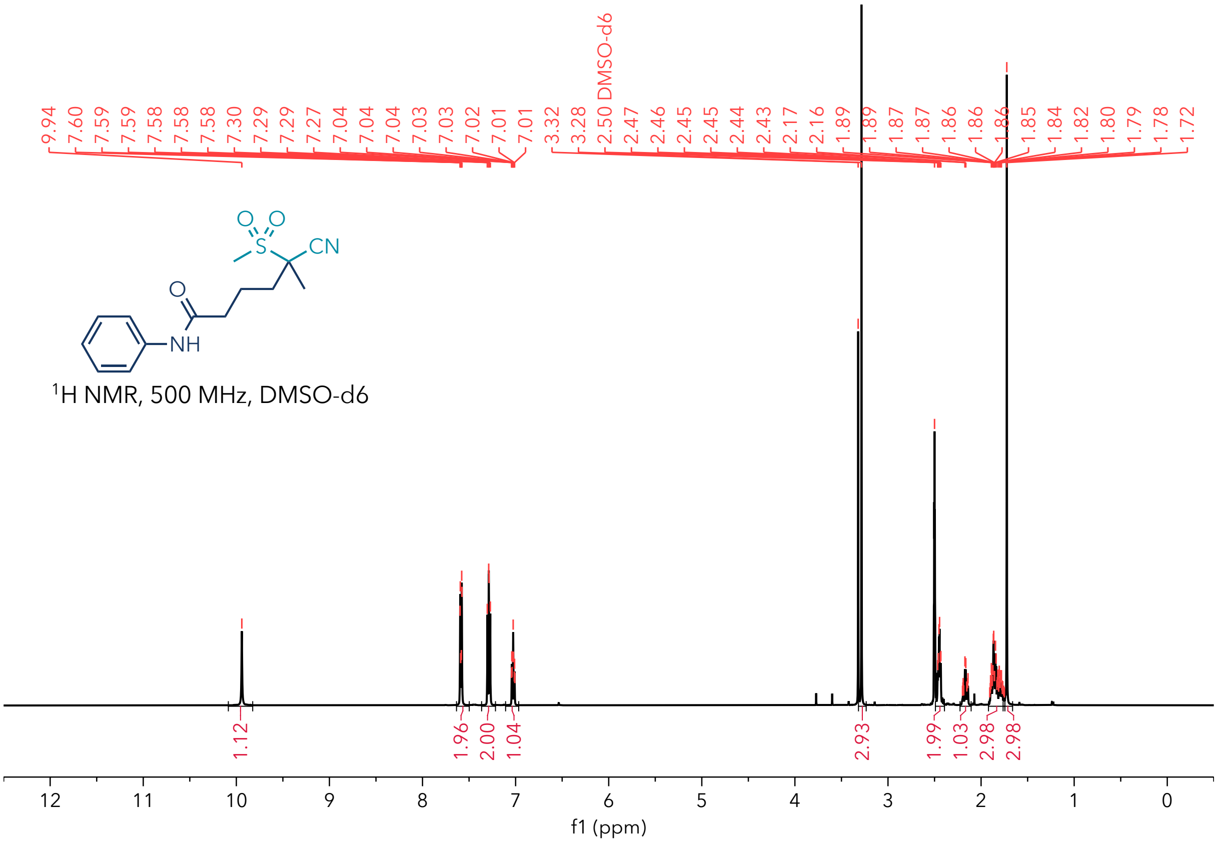
^{1}H NMR, 500 MHz, DMSO-d6
CN
NH
f1 (ppm)

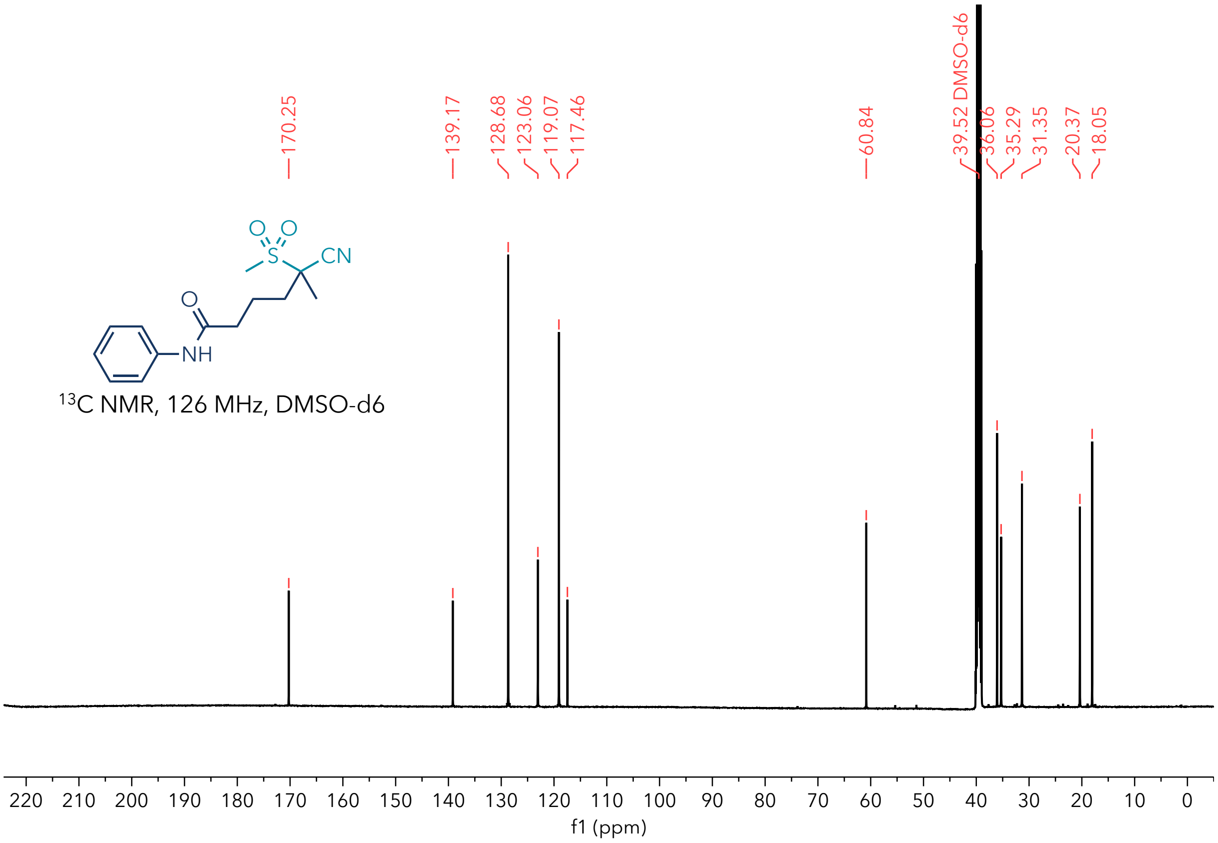
^{13}C NMR, 126 MHz, DMSO-d6
CN
NH
f1 (ppm)

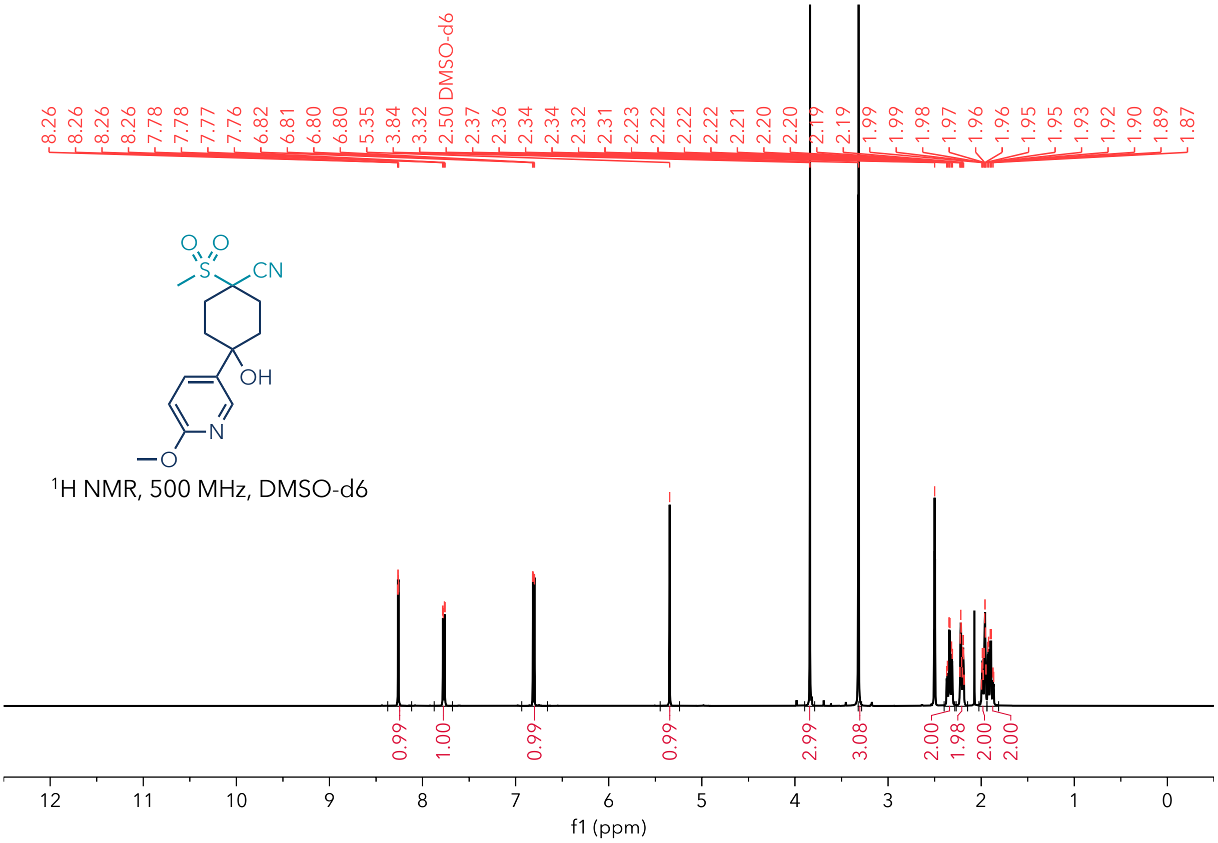

^{1}H NMR, 500 MHz, DMSO-d6

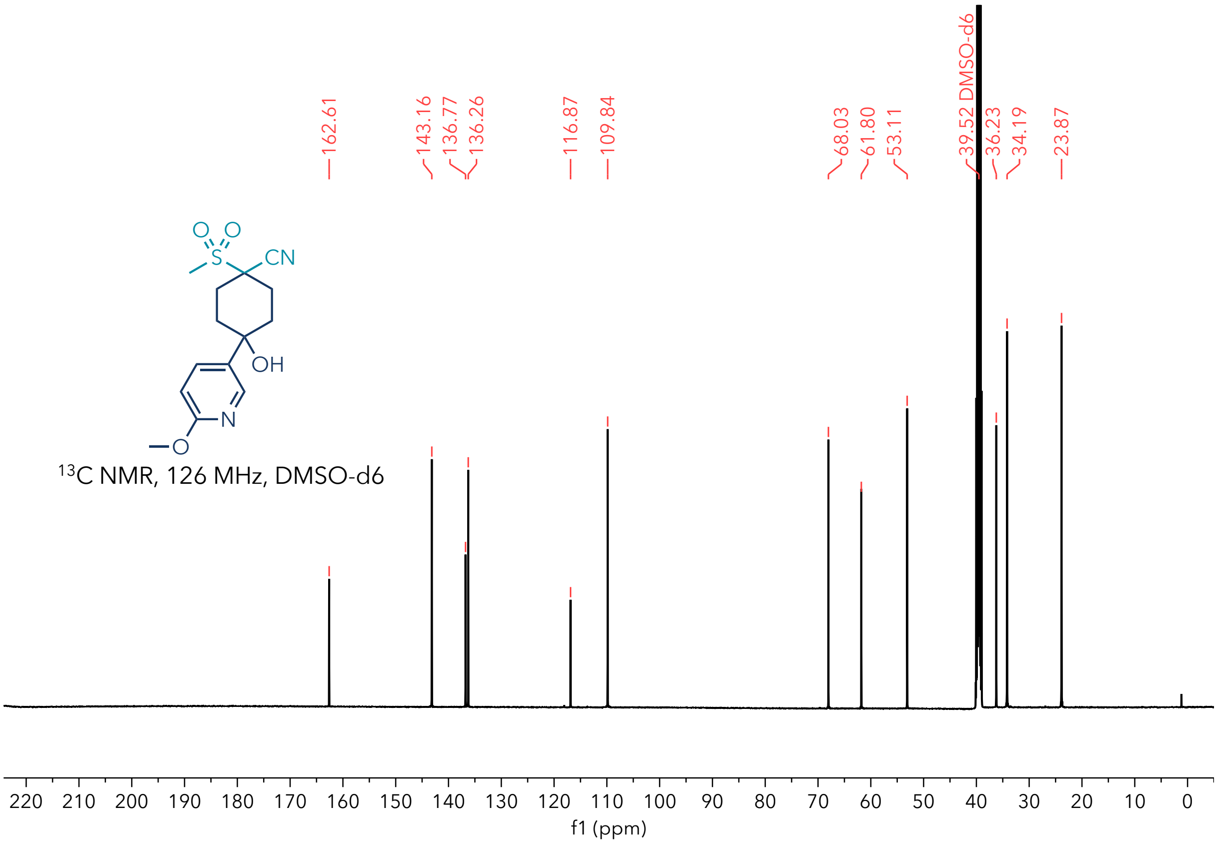

^{13}C NMR, 126 MHz, DMSO-d6

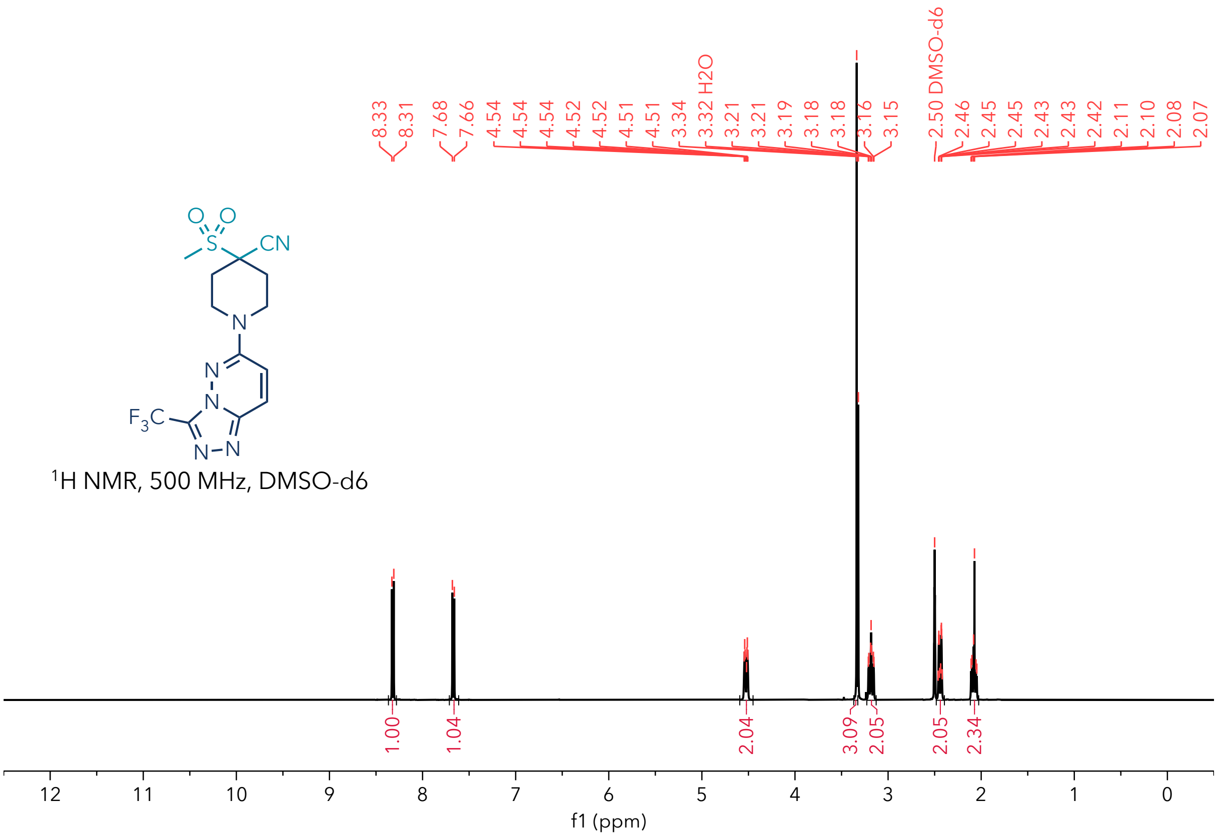

¹H NMR, 500 MHz, DMSO-d6

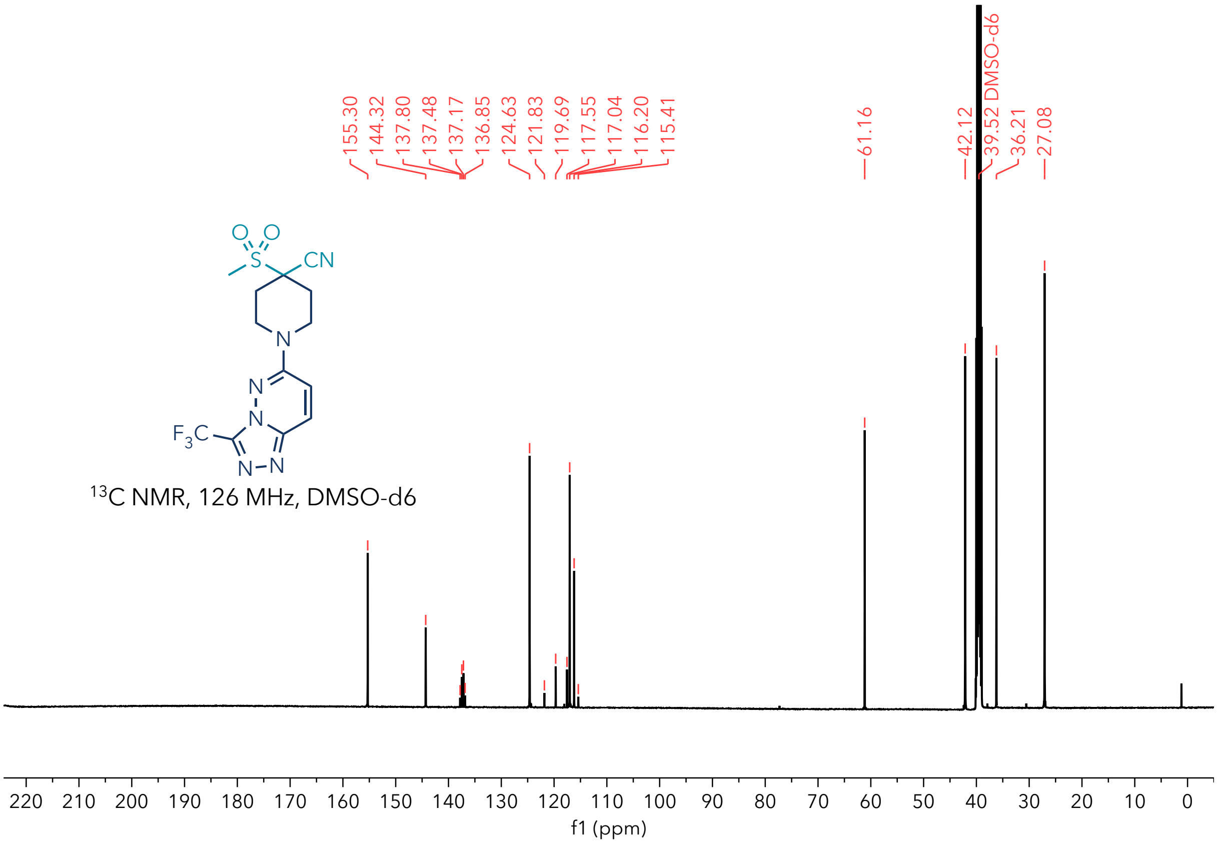

¹³C NMR, 126 MHz, DMSO-d6

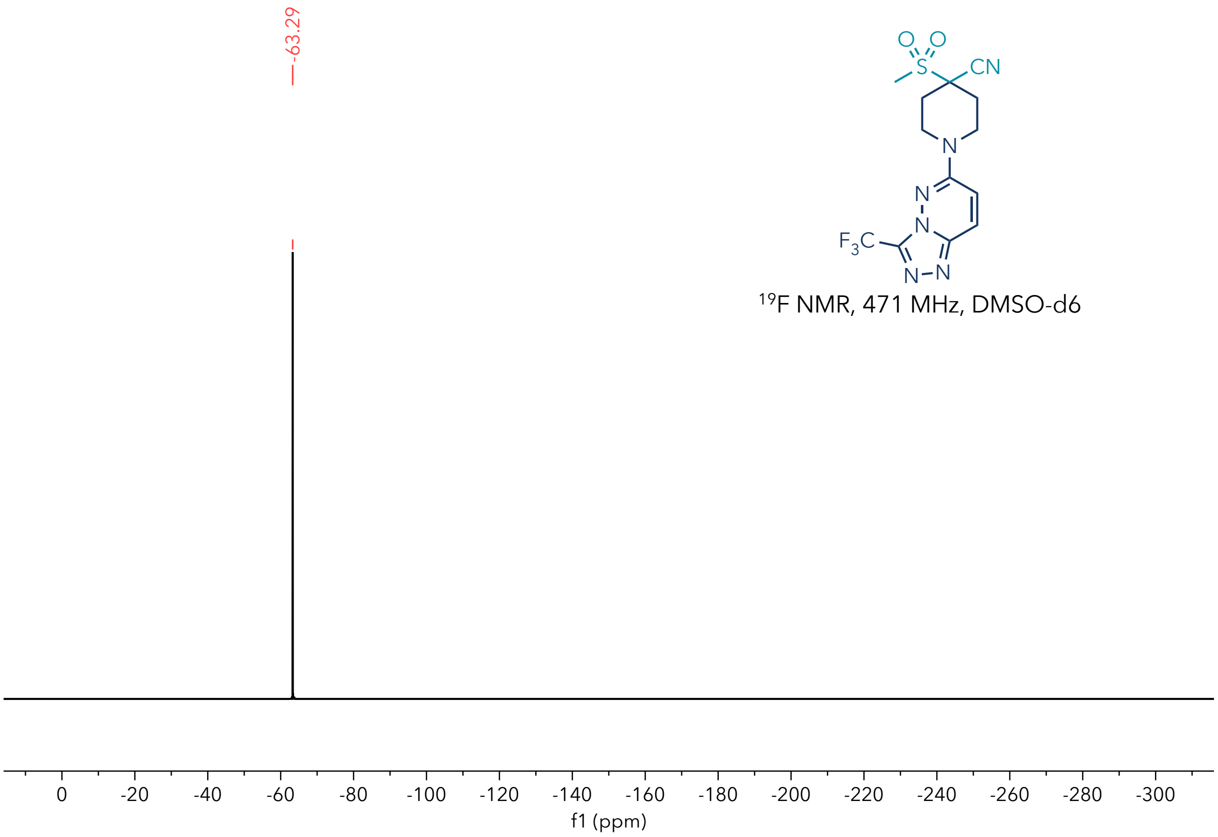

^{19}F NMR, 471 MHz, DMSO-d6

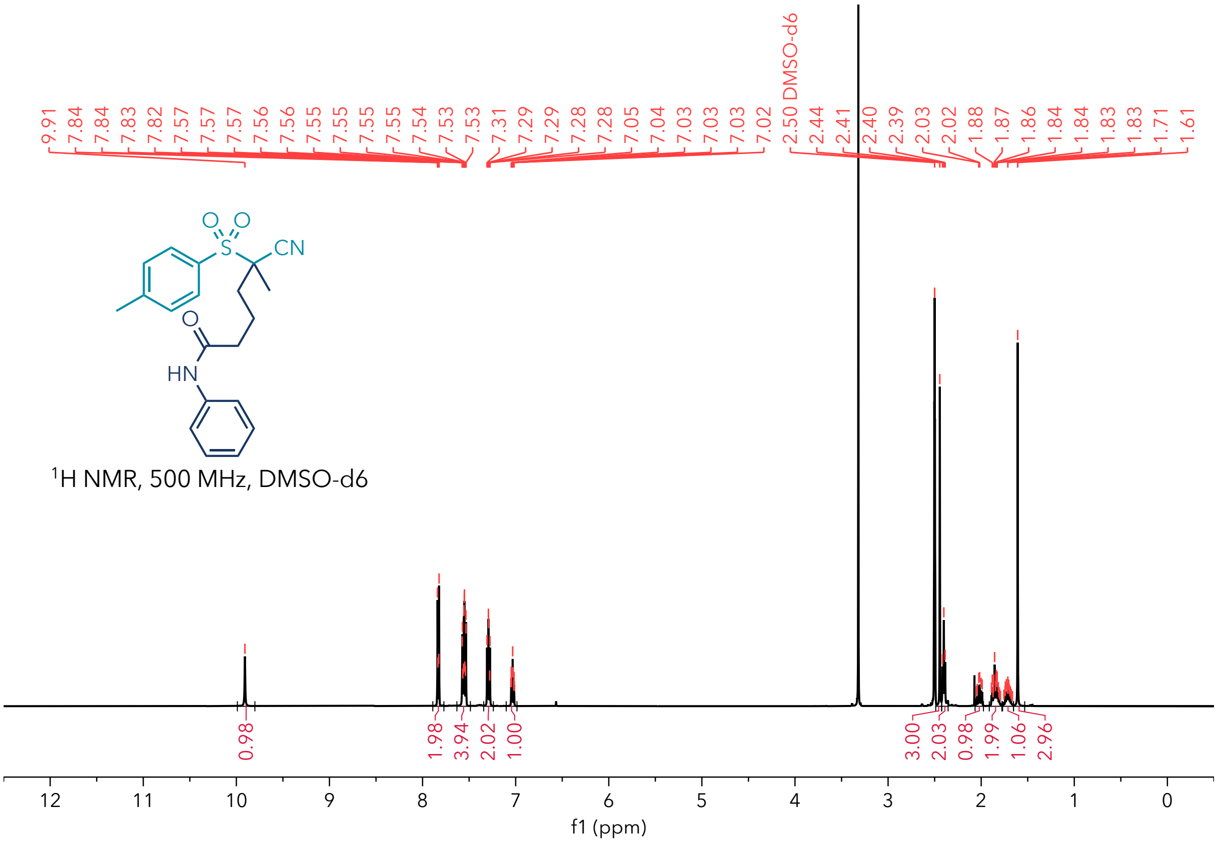
9.91 7.84 7.84 7.83 7.82 7.57 7.57 7.57 7.56 7.56 7.55 7.55 7.55 7.54 7.53 7.53 7.31 7.29 7.29 7.28 7.28 7.05 7.04 7.03 7.03 7.03 7.02 2.50 DMSO-d6 2.44 2.41 2.40 2.39 2.03 2.02 1.88 1.87 1.86 1.84 1.84 1.83 1.83 1.71 1.61
O O S CN O HN
^{1}H NMR, 500 MHz, DMSO-d6
0.98 1.98 3.94 2.02 1.00 3.00 2.03 0.98 1.99 1.06 2.96
12 11 10 9 8 7 6 5 4 3 2 1 0
f1 (ppm)

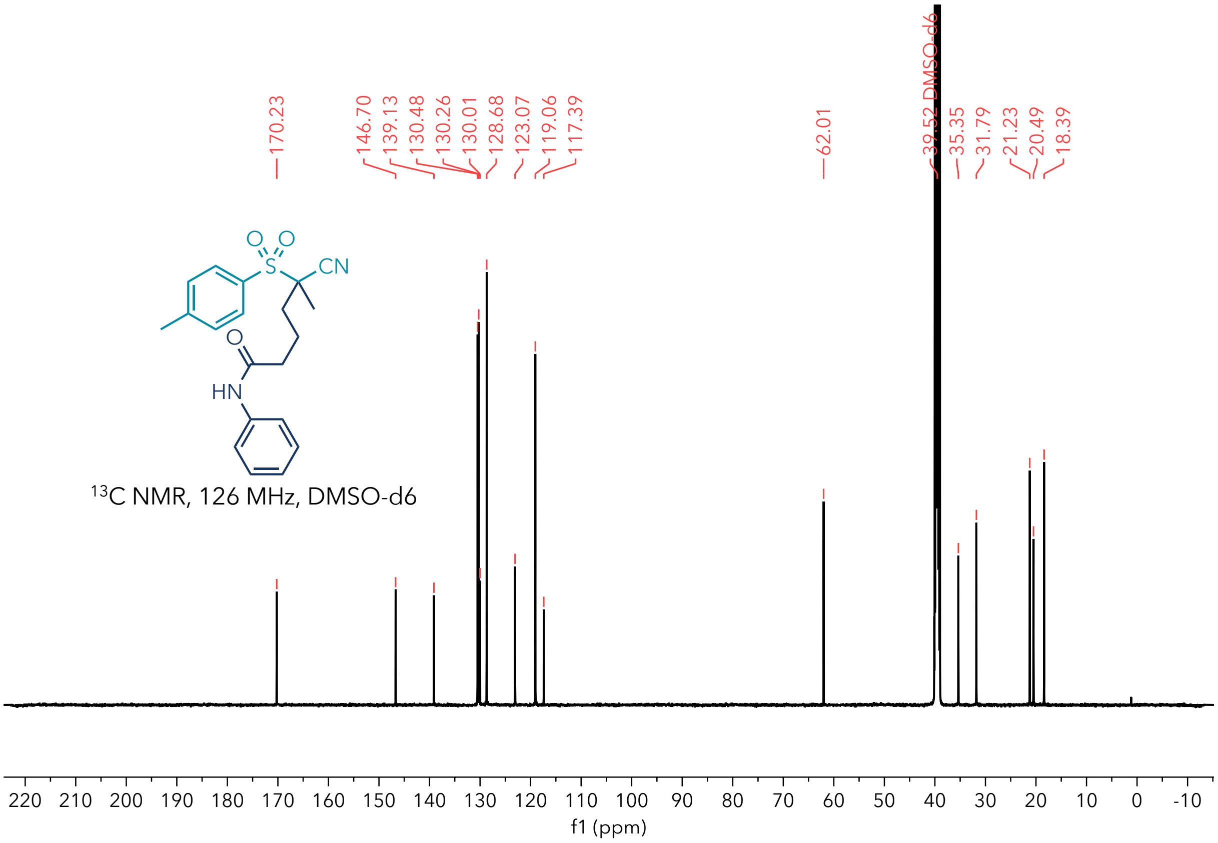
170.23 146.70 139.13 130.48 130.26 130.01 128.68 123.07 119.06 117.39 62.01 39.52 DMSO-d6 35.35 31.79 21.23 20.49 18.39
O O S CN O HN
^{13}C NMR, 126 MHz, DMSO-d6
220 210 200 190 180 170 160 150 140 130 120 110 100 90 80 70 60 50 40 30 20 10 0 -10
f1 (ppm)

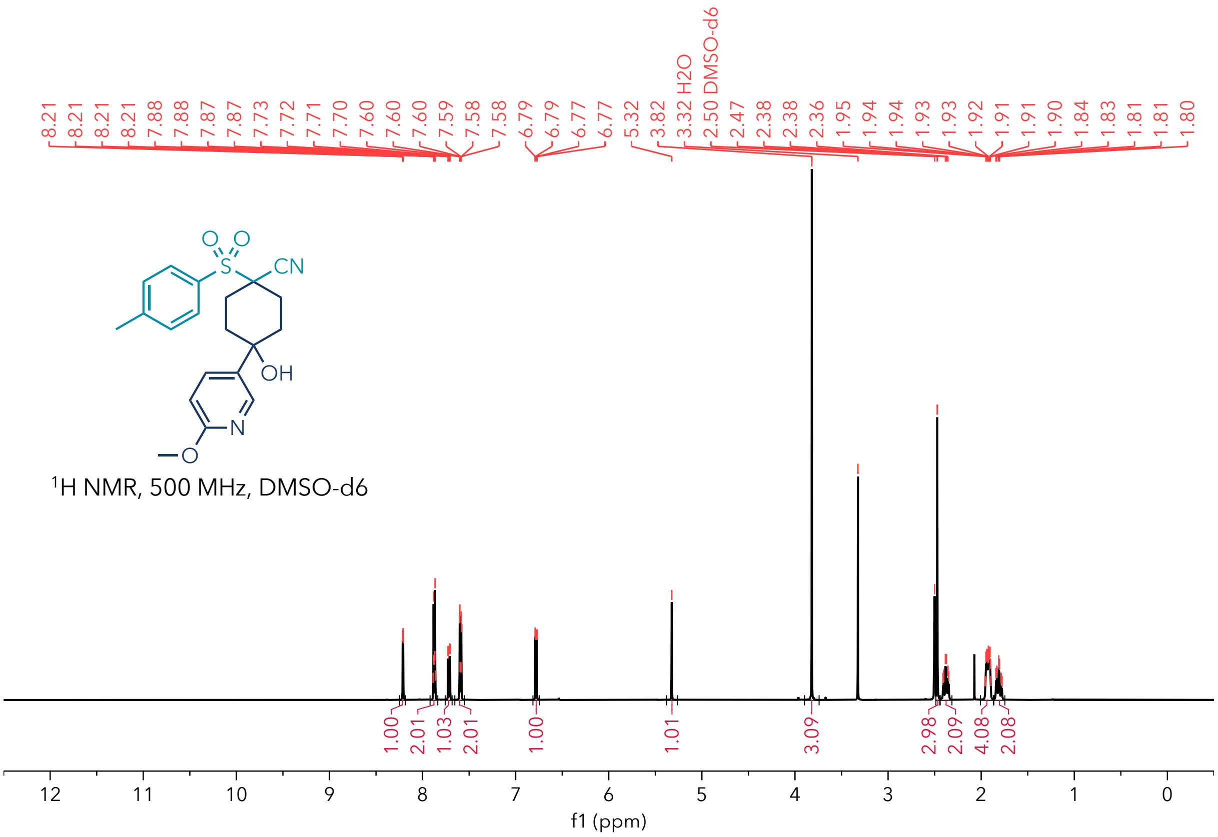
8.21
8.21
8.21
8.21
7.88
7.88
7.87
7.87
7.73
7.72
7.71
7.70
7.60
7.60
7.60
7.59
7.58
7.58
6.79
6.79
6.77
6.77
5.32
3.82
3.32 H2O
2.50 DMSO-d6
2.47
2.38
2.38
2.36
1.95
1.94
1.94
1.93
1.93
1.92
1.91
1.91
1.90
1.84
1.83
1.81
1.81
1.80
O
O
S
CN
OH
N
O
^{1}H NMR, 500 MHz, DMSO-d6
1.00
2.01
1.03
2.01
1.00
1.01
3.09
2.98
2.09
4.08
2.08
12
11
10
9
8
7
6
5
4
3
2
1
0
f1 (ppm)

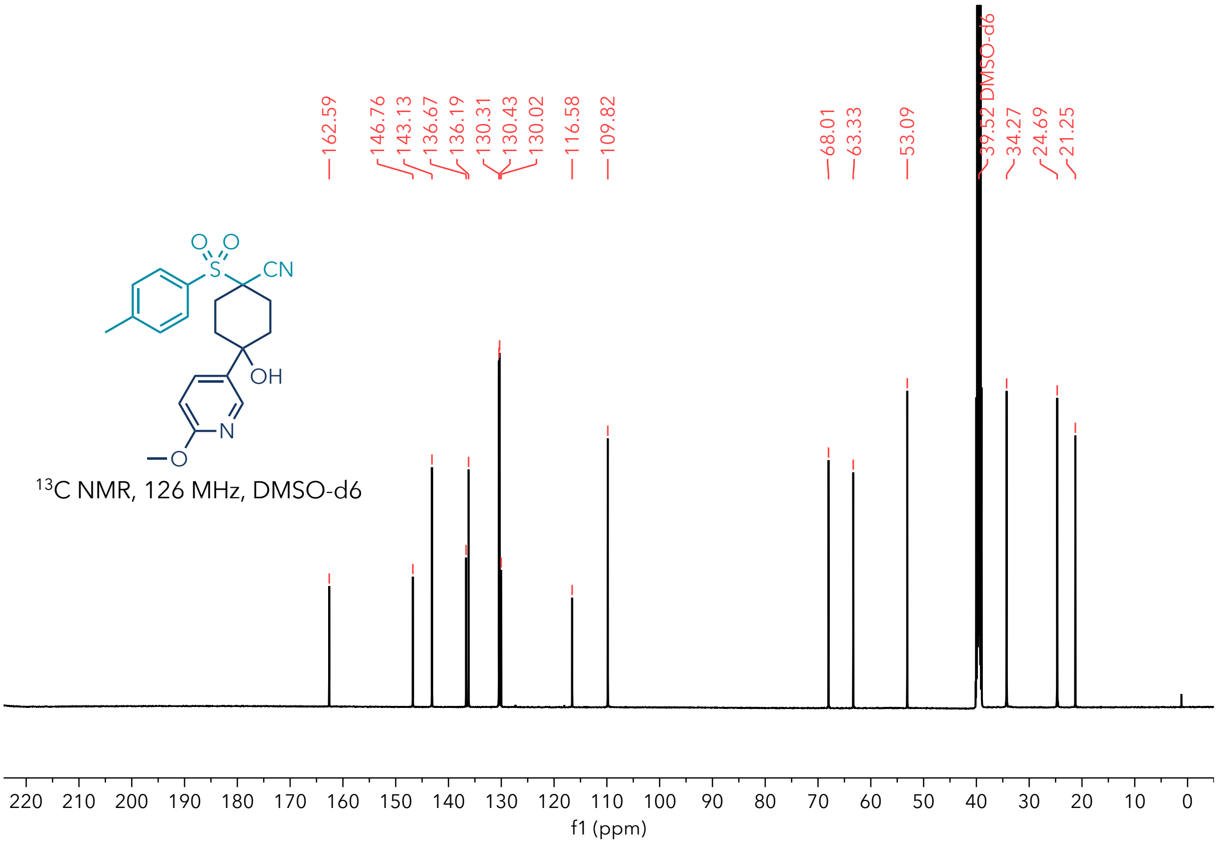
162.59
146.76
143.13
136.67
136.19
130.31
130.43
130.02
116.58
109.82
68.01
63.33
53.09
39.52 DMSO-d6
34.27
24.69
21.25
O
O
S
CN
OH
N
O
^{13}C NMR, 126 MHz, DMSO-d6
220
210
200
190
180
170
160
150
140
130
120
110
100
90
80
70
60
50
40
30
20
10
0
f1 (ppm)

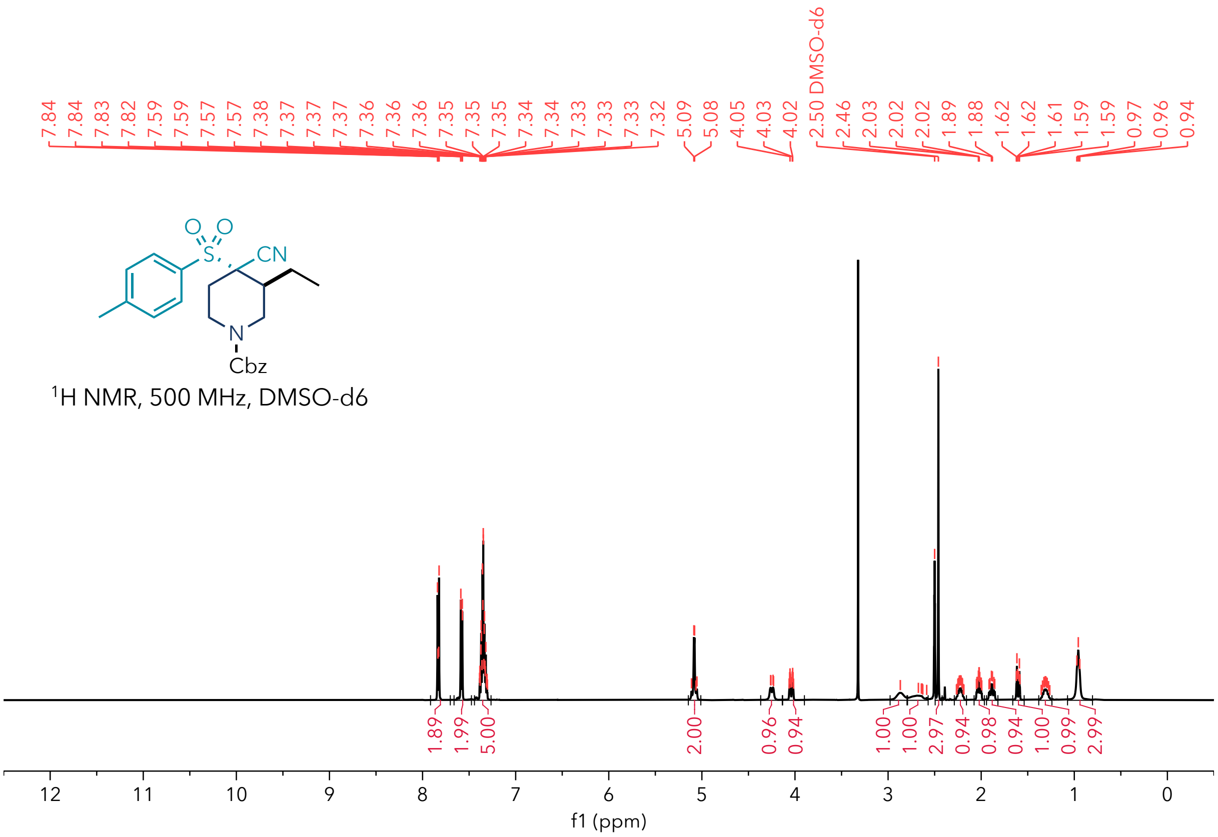
7.84 7.84 7.83 7.82 7.59 7.59 7.57 7.57 7.38 7.37 7.37 7.37 7.36 7.36 7.36 7.35 7.35 7.35 7.34 7.34 7.33 7.33 7.33 7.32 5.09 5.08 4.05 4.03 4.02 2.50 DMSO-d6 2.46 2.03 2.02 2.02 1.89 1.88 1.62 1.62 1.61 1.59 1.59 0.97 0.96 0.94
O O
S
CN
N
Cbz
^{1}H NMR, 500 MHz, DMSO-d6
1.89 1.99 5.00 2.00 0.96 0.94 1.00 1.00 2.97 0.94 0.98 0.94 1.00 0.99 2.99
12 11 10 9 8 7 6 5 4 3 2 1 0
f1 (ppm)

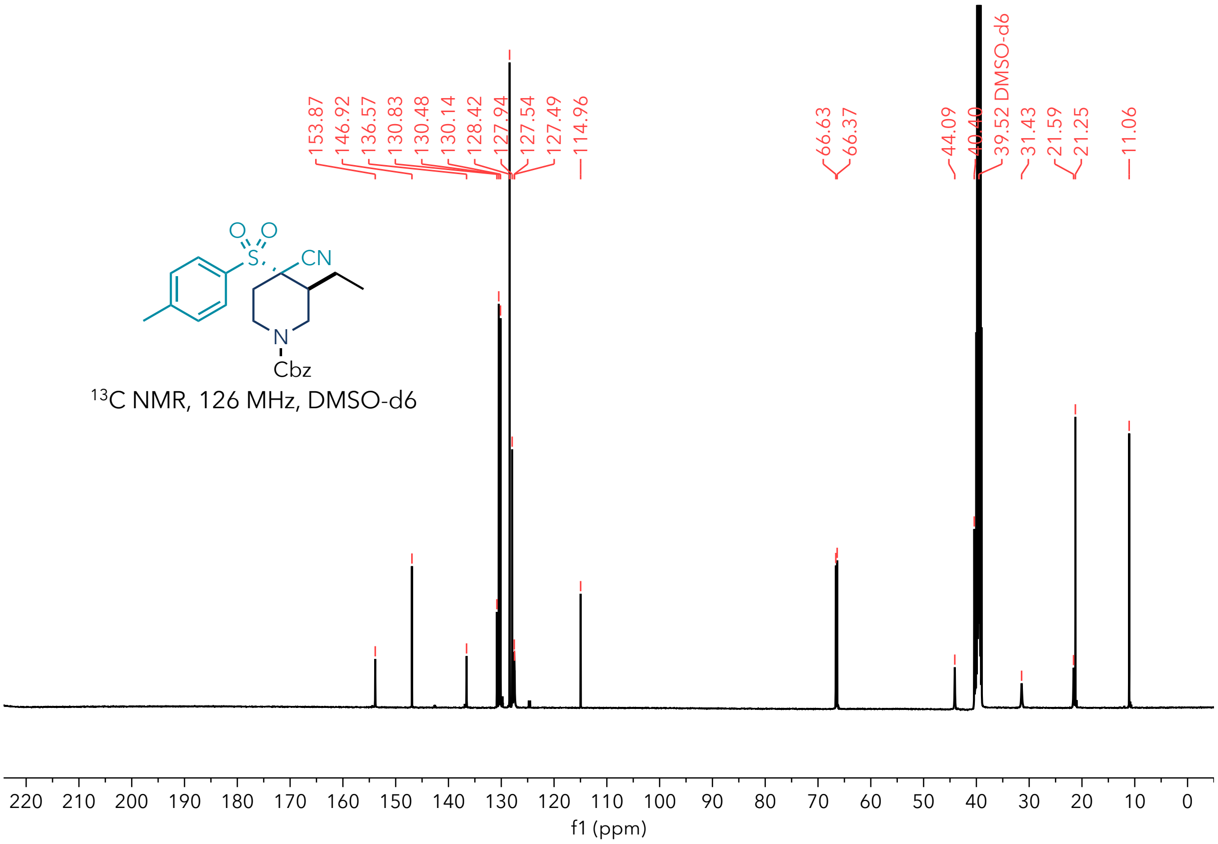
153.87 146.92 136.57 130.83 130.48 130.14 128.42 127.94 127.54 127.49 114.96
66.63 66.37
44.09 40.40 39.52 DMSO-d6 31.43 21.59 21.25 11.06
O O
S
CN
N
Cbz
^{13}C NMR, 126 MHz, DMSO-d6
220 210 200 190 180 170 160 150 140 130 120 110 100 90 80 70 60 50 40 30 20 10 0
f1 (ppm)

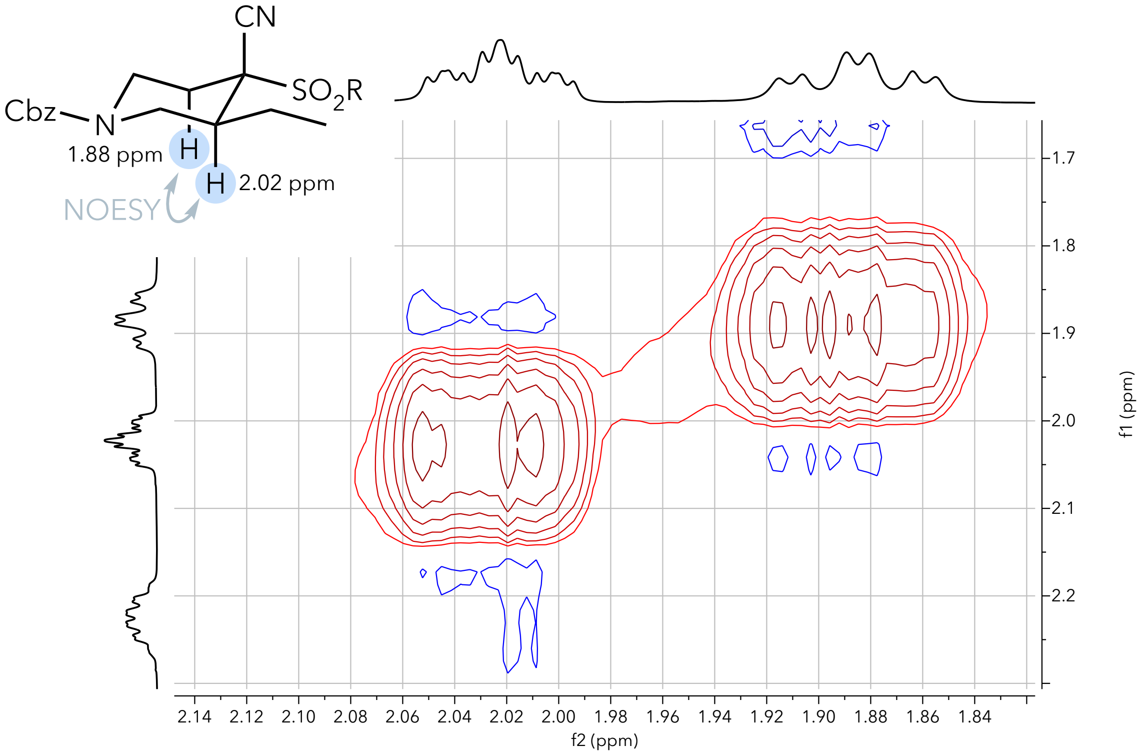
CN
Cbz
N
SO_2R
1.88 ppm
H
H
2.02 ppm
NOESY
f2 (ppm)
f1 (ppm)
2.14 2.12 2.10 2.08 2.06 2.04 2.02 2.00 1.98 1.96 1.94 1.92 1.90 1.88 1.86 1.84
1.7 1.8 1.9 2.0 2.1 2.2

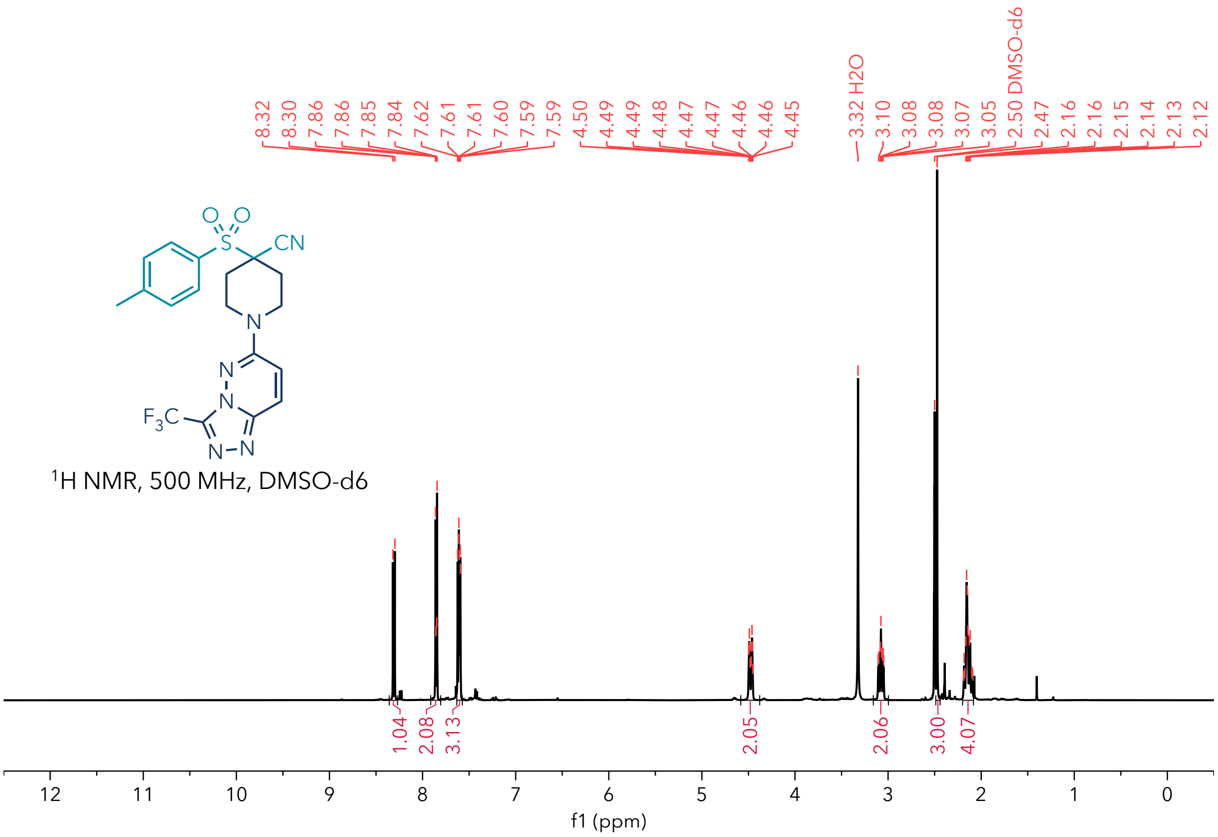

^{1}H NMR, 500 MHz, DMSO-d6

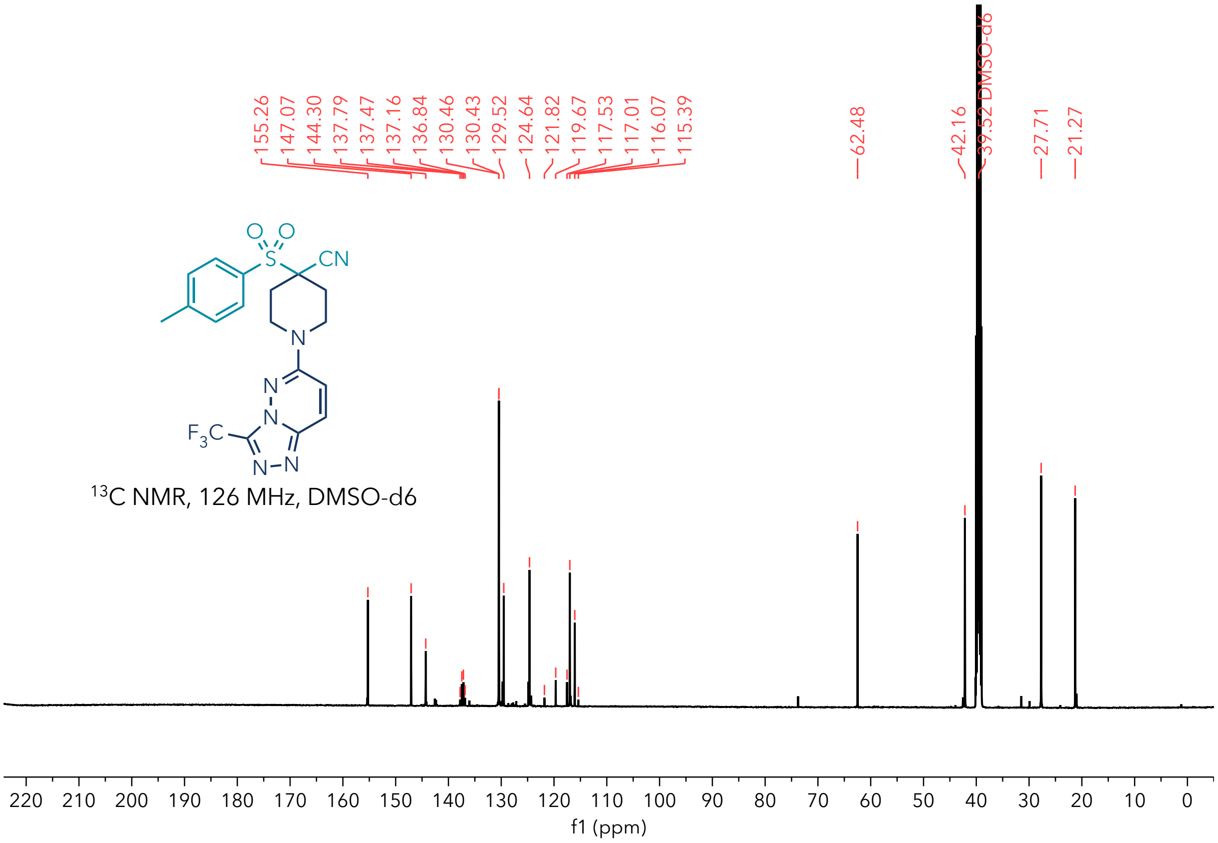

^{13}C NMR, 126 MHz, DMSO-d6

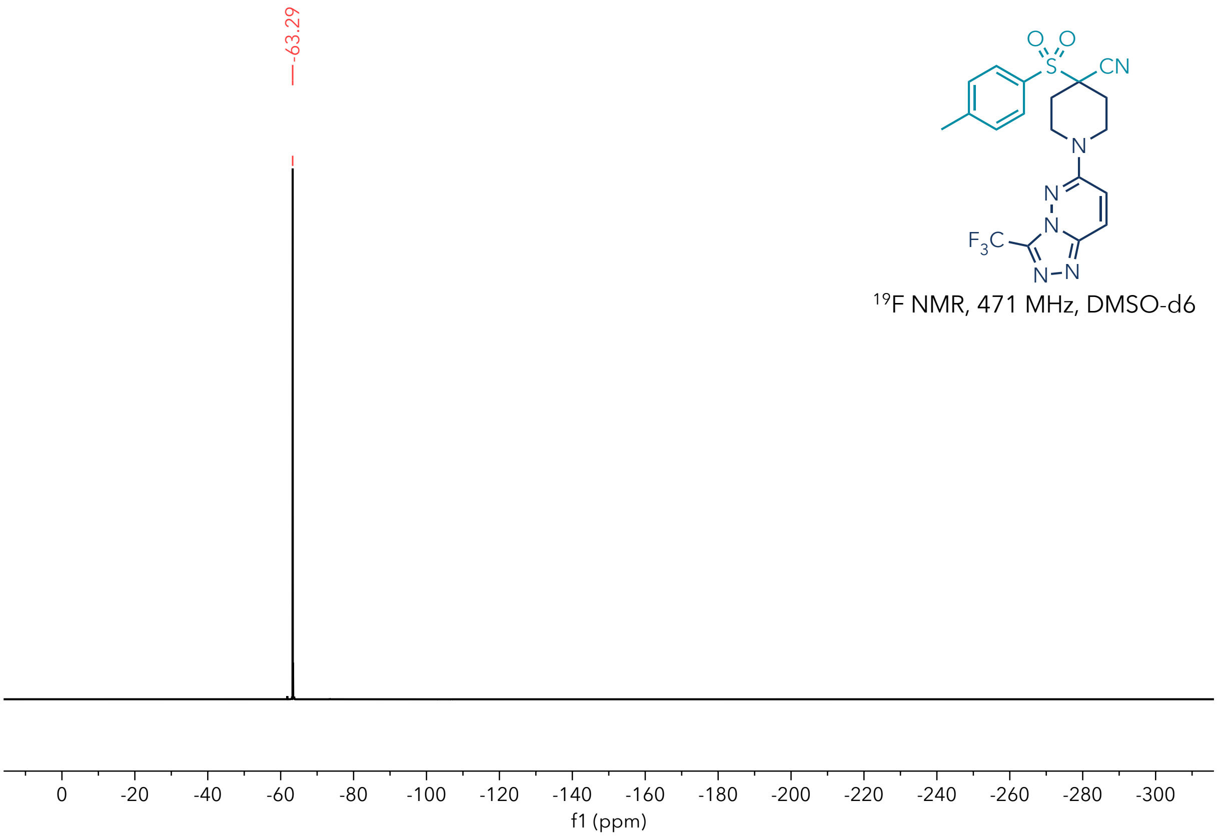

-63.29
CN
N
F3C
19F NMR, 471 MHz, DMSO-d6
0
-20
-40
-60
-80
-100
-120
-140
-160
-180
-200
-220
-240
-260
-280
-300
f1 (ppm)
